# Supplementary material for: Photocatalytic anti-Markovnikov hydro- and haloazidation of alkenes
Source: Nat Commun. 2025 Aug 25;16:7906. doi: 10.1038/s41467-025-63203-w (PMC12378208; doi:10.1038/s41467-025-63203-w)
Supplement: Supplementary file 1 — Supplementary Information [file 41467_2025_63203_MOESM1_ESM.pdf]

Supplementary Information

for

Photocatalytic Anti-Markovnikov Hydro- and Haloazidation of Alkenes

Kang-Jie Bian,† Shijin Yu,† Ying Chen, Qiming Liu, Xiaowei Chen, David Nemoto Jr, Shih-Chieh Kao, Angel A. Martí and Julian G. West*

Department of chemistry, Rice University, 6100 Main St MS 602, Houston, TX 77005.

Contents

I. Supplemental Methods 4

1.1 General Information 4

1.2 General Procedures for Substrate Synthesis 4

General Procedure 1 for the Synthesis of Unactivated Alkenes 4

General Procedure 2 for the Synthesis of Unactivated Alkenes 5

Procedure for the Synthesis of N-(but-3-en-1-yl)benzenesulfonamide 5

Procedure for the Synthesis of N-(but-3-en-1-yl)-N-methylbenzenesulfonamide 6

Procedure for the Synthesis of 2-(but-3-en-1-yl)isoindoline-1,3-dione 6

1.3 General Procedures for Photocatalytic Hydro- and Haloazidation of Alkenes 7

General Procedure A for hydroazidation of alkenes 7

General Procedure B for deuteroazidation of alkenes 8

General Procedure C for chloroazidation of alkenes 8

General Procedure D for bromoazidation of alkenes 9

II. Supplemental Discussion 10

2.1 Optimization of Hydroazidation 10

Supplementary Table 1. Initial testing of hydroazidation. 10

Supplementary Table 2. Bidentate ligand screening for hydroazidation. 10

Supplementary Table 3. Tridentate ligand screening for hydroazidation. 11

Supplementary Table 4. Solvent screening for hydroazidation. 11

Supplementary Table 5. HAT co-catalyst screening for hydroazidation. 12

Supplementary Table 6. Control experiments of hydroazidation. 12

2.2 Optimization of Chloroazidation 13

Supplementary Table 7. Initial testing of chloroazidation. 13

Supplementary Table 8. Ligand effect screening for chloroazidation. 13

Supplementary Table 9. Catalyst type/loading and azide amount screening for chloroazidation. 14

Supplementary Table 10. Control experiments of chloroazidation. 14

2.3 Optimization of Bromoazidation 14

Supplementary Table 11. Azide and NBS amount screening for bromoazidation. 14

Supplementary Table 12. Iron screening for bromoazidation. 15

Supplementary Table 13. Tridentate ligand screening for bromoazidation. 15

Supplementary Table 14. Control experiments of bromoazidation. 16

2.3 Characterization of Corresponding Products 17
2.4 Additional substrates for photocatalytic hydroazidation 55
Supplementary Figure 1. 1H NMR of Diene Hydroazidation 56
Supplementary Figure 2. 13C NMR of Diene Hydroazidation 57
2.5 Scale up reaction and late-stage application 58
Supplementary Figure 3. Scale Up of Hydroazidation Reaction 58
2.6 Kinetic Isotope Experiments (KIE) 60
Supplementary Figure 4. Kinetic Isotope Effect Reaction Experiments 60
Supplementary Table 15. Kinetic Isotope Effect Experiment Data 60
Supplementary Figure 5. Rate Measurement of Hydroazidation 61
Supplementary Figure 6. Rate Measurement of Deuteroazidation 62
2.7 Spectroscopic studies of different iron species and time-resolved UV-visible studies 62
Supplementary Figure 7. UV-Vis Studies of Iron Complexes 62
2.8 CV studies of photocatalytic hydroazidation 64
Supplementary Figure 8. Cyclic Voltammetry Studies of Reaction Components 64
III. Supplemental Figures 66
IV. Supplemental References 321

I. Supplemental Methods

1.1 General Information

All reagents were purchased from commercially available sources and used without further purification. All reactions were monitored by either ^{1}H NMR or thin layer chromatography (TLC) carried out on 0.25 mm pre-coated silica plates (F-254) purchased from Silicycle, Quebec, Canada, using shortwave UV light as visualizing agent and KMnO4 or phosphomolybdic acid (PMA) as developing agents. Flash column chromatography was performed using SiliaFlash-P60 silica gel (40 – 63 μm) purchased from Silicycle, Quebec, Canada. ^{1}H, ^{13}C and ^{19}F NMR spectra were recorded on a Bruker DRX-600 spectrometers operating at 600 MHz for proton nuclei, 151 MHz for carbon nuclei and 565 MHz for fluorine nuclei were calibrated using residual undeuterated solvent as an internal reference (CDCl3: 7.26 ppm ^{1}H NMR and 77.00 ppm ^{13}C NMR). PR160L 390 nm LEDs (25% intensity) from Kessil Lights were used as light source. For reporting NMR peak multiplicities, the following abbreviations were used: s = singlet, d = doublet, t = triplet, q = quartet, quin = quintet, hept = heptet, m = multiplet. High-resolution mass spectra (HRMS) or mass spectra (MS) were recorded on an Agilent UHPLC TOF mass spectrometer using electrospray ionization time-of-flight (ESI-TOF), chemical ionization time-of-flight (CI-TOF), atmospheric pressure chemical ionization (APCI) or gas chromatography–mass spectrometry (GC-MS).

1.2 General Procedures for Substrate Synthesis

General Procedure 1 for the Synthesis of Unactivated Alkenes

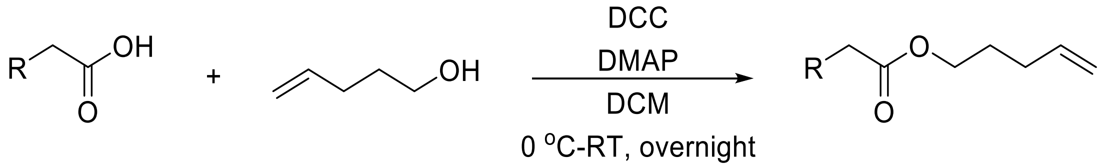

To an RB flask were added 5-pentenol (4.9 mmol, 1.96 equiv), carboxylic acid (2.5 mmol, 1.0 equiv), 4-dimethylamino pyridine (0.24 mmol, 0.097 equiv), and a stir bar. The RB flask was then evacuated and backfilled with nitrogen gas three times. Dry dichloromethane (0.225 M) was added via syringe to the RB flask, dissolving the solid components. The RB flask was then placed in an ice bath positioned on top of a stirring plate. Dicyclohexyl carbodiimide (4.85 mmol, 1.94 mmol) was added to the mixture via syringe dropwise over a period of 5 minutes. The ice bath was then removed, allowing the reaction to return to room temperature. The reaction was left to stir overnight. Following reaction, the mixture was concentrated through rotary

evaporation. Subsequent flash column chromatography (hexanes/EtOAc) allowed for isolation of the ester.[1-2]

General Procedure 2 for the Synthesis of Unactivated Alkenes

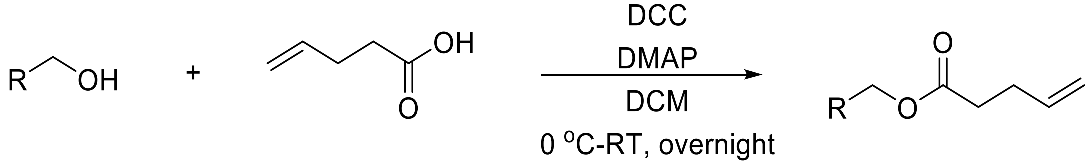

To an RB flask were added 5-pentenoic acid (2.5 mmol, 1.0 equiv), alcohol (4.9 mmol, 1.96 equiv), 4-dimethylamino pyridine (0.24 mmol, 0.097 equiv), and a stir bar. The RB flask was then evacuated and backfilled with nitrogen gas three times. Dry dichloromethane (0.225 M) was added via syringe to the RB flask, dissolving the solid components. The RB flask was then placed in an ice bath positioned on top of a stirring plate. Dicyclohexyl carbodiimide (4.85 mmol, 1.94 mmol) was added to the mixture via syringe dropwise over a period of 5 minutes. The ice bath was then removed, allowing the reaction to return to room temperature. The reaction was left to stir overnight. Following reaction, the mixture was concentrated through rotary evaporation. Subsequent flash column chromatography (hexanes/EtOAc) allowed for isolation of the ester.[1-2]

Procedure for the Synthesis of N-(but-3-en-1-yl)benzenesulfonamide

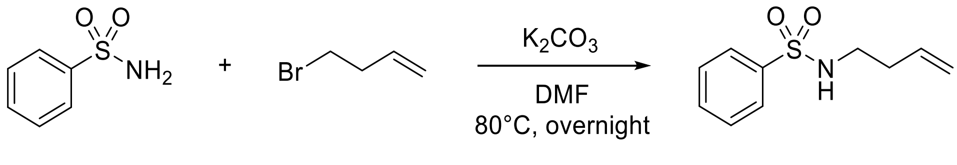

To an RB flask were added benzenesulfonamide (944 mg, 6.0 mmol), 4-bromobut-1-ene (0.8 mL, 6.0 mmol), dimethylformamide (30 mL), and a stir bar. Potassium carbonate (830 mg, 6.0 mmol) was added to the reaction mixture. After stirring overnight at 80 °C, the mixture was

cooled to room temperature and quenched with water. The reaction mixture was then washed with brine and extracted with diethyl ether. The organic layer was concentrated through rotary evaporation. Subsequent flash column chromatography (hexanes/EtOAc) (3:1) allowed for isolation of *N*-(but-3-en-1-yl)benzenesulfonamide.[3]

Procedure for the Synthesis of N-(but-3-en-1-yl)-N-methylbenzenesulfonamide

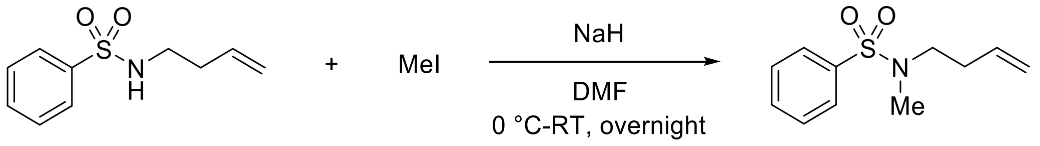

To an RB flask were added sodium hydride (60% in mineral oil, 240 mg, 6 mmol), dimethylformamide (25 mL), a solution of *N*-(but-3-en-1-yl)benzenesulfonamide (1.20 g, 5 mmol) in DMF (5 mL), and a stir bar in an ice bath at 0 °C. The reaction mixture was brought to room temperature and stirred for 30 minutes. The reaction mixture was cooled to 0 °C in an ice bath again, and a solution of methyl iodide (1.06 g, 7.5 mmol) in DMF (5 mL) was added dropwise over a period of 5 minutes by syringe. The reaction mixture was brought to room temperature and left to run overnight. The reaction mixture was quenched with a saturated aqueous solution of sodium bicarbonate. The mixture was then washed with brine and extracted with diethyl ether. The organic phase was dried over sodium sulfate and concentrated through rotary evaporation. Subsequent flash column chromatography (hexanes/EtOAc) (10:1) produced *N*-(but-3-en-1-yl)-*N*-methylbenzenesulfonamide.[4]

Procedure for the Synthesis of 2-(but-3-en-1-yl)isoindoline-1,3-dione

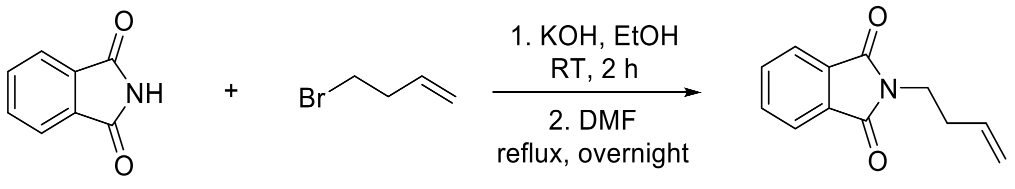

To an RB flask were added phthalimide (1.71 g, 11.6 mmol), potassium hydroxide (0.650 g, 11.6 mmol), ethyl alcohol (20 mL), and a stir bar. The reaction mixture was stirred at room temperature for 2 h and evaporated to remove EtOH. The resulting residue was then dissolved in dimethylformamide (15 mL) and 4-bromobut-1-ene (1.10 mL, 12.8 mmol) was added. The reaction mixture was stirred at reflux overnight. The reaction mixture was cooled, diluted with ethyl acetate, and quenched with saturated sodium bicarbonate. The mixture was then washed with brine. The extracted organic layer was dried over sodium sulfate and concentrated through rotary evaporation. Subsequent flash column chromatography (hexanes/EtOAc) (10:1) produced 2-(but-3-en-1-yl)isoindoline-1,3-dione.[5]

1.3 General Procedures for Photocatalytic Hydro- and Haloazidation of Alkenes

General Procedure A for hydroazidation of alkenes

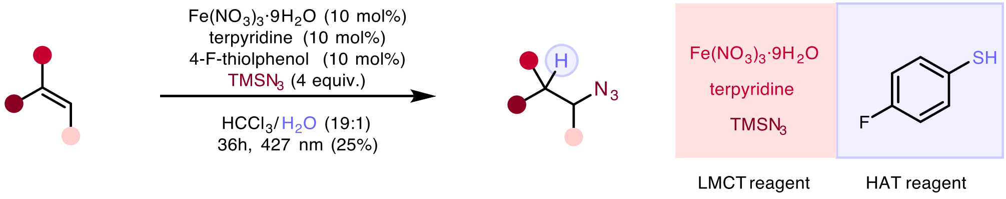

$Fe(NO_3)_3·9H_2O$ (10 mol%, 0.1 equiv.) and terpyridine (10 mol%, 0.1 equiv.) were added in an oven-dried 8-mL test vial containing a Teflon®-coated magnetic stir bar. The vial was evacuated and backfilled with N2 (repeated for 4 times), followed by addition of alkenes (0.1 mmol, 1.0 equiv.), $TMSN_3$ (0.40 mmol, 4.0 equiv.), 4-F-thiolphenol (10 mol%, 0.1 equiv.) in $HCCl_3$/H2O (19:1, 0.1 M in regard to alkenes) via syringe under N2. The reaction mixture was placed under 427nm Kessil® light after sealing the punctured holes of the vial cap with vacuum grease and electric tape/parafilm for better air-tight protection and allowed to react at room temperature for 36 h. Following this, the reaction mixture was filtered through a pad of celite which was subsequently rinsed with DCM. The filtrate was concentrated, and the residue was then purified by flash column chromatography or preparatory thin-layer chromatography to give the corresponding hydroazidated products.

General Procedure B for deuteroazidation of alkenes

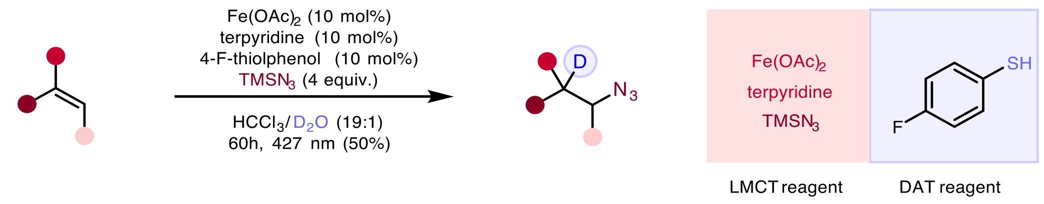

$Fe(OAc)_2$ (10 mol%, 0.1 equiv.) and terpyridine (10 mol%, 0.1 equiv.) were added in an oven-dried 8-mL test vial containing a Teflon®-coated magnetic stir bar. The vial was evacuated and backfilled with N2 (repeated for 4 times), followed by addition of alkenes (0.1 mmol, 1.0 equiv.), $TMSN_3$ (0.40 mmol, 4.0 equiv.), 4-F-thiolphenol (10 mol%, 0.1 equiv.) in $HCCl_3$/D2O (19:1, 0.1 M in regard to alkenes) via syringe under N2. The reaction mixture was placed under 427nm Kessil® light after sealing the punctured holes of the vial cap with vacuum grease and electric tape/parafilm for better air-tight protection and allowed to react at room temperature for 60 h. Following this, the reaction mixture was filtered through a pad of celite which was subsequently rinsed with DCM. The filtrate was concentrated, and the residue was then purified by flash column chromatography or preparatory thin-layer chromatography to give the corresponding deuteroazidated products.

General Procedure C for chloroazidation of alkenes

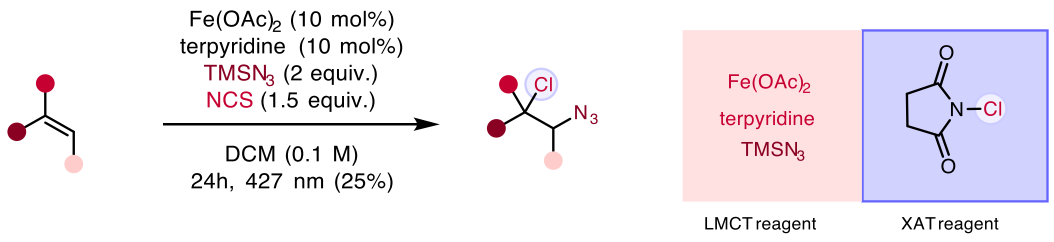

$Fe(OAc)_2$ (10 mol%, 0.1 equiv.), terpyridine (10 mol%, 0.1 equiv.) and NCS (0.15 mmol, 1.5 equiv.) were added in an oven-dried 8-mL test vial containing a Teflon®-coated magnetic stir bar. The vial was evacuated and backfilled with N2 (repeated for 4 times), followed by addition of alkenes (0.1 mmol, 1.0 equiv.), $TMSN_3$ (0.20 mmol, 2.0 equiv.) in DCM (0.1 M in regard to alkenes) via syringe under N2. The reaction mixture was placed under 427nm Kessil® light after sealing the punctured holes of the vial cap with vacuum grease and electric tape/parafilm

for better air-tight protection and allowed to react at room temperature for 24 h. Following this, the reaction mixture was filtered through a pad of celite which was subsequently rinsed with DCM. The filtrate was concentrated, and the residue was then purified by flash column chromatography or preparatory thin-layer chromatography to give the corresponding chloroazidated products.

General Procedure D for bromoazidation of alkenes

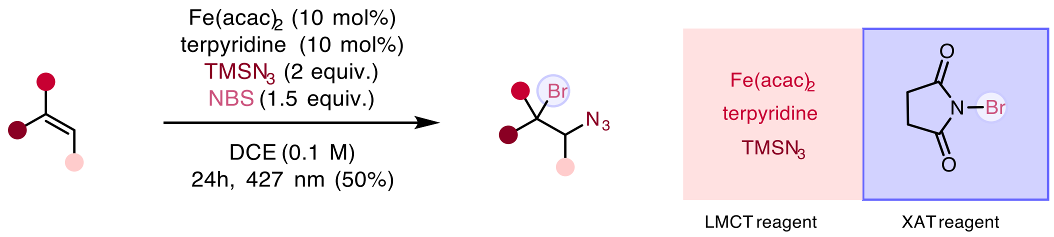

$Fe(acac)_2$ (10 mol%, 0.1 equiv.), terpyridine (10 mol%, 0.1 equiv.) and NBS (0.15 mmol, 1.5 equiv.) were added in an oven-dried 8-mL test vial containing a Teflon®-coated magnetic stir bar. The vial was evacuated and backfilled with N2 (repeated for 4 times), followed by addition of alkenes (0.1 mmol, 1.0 equiv.), $TMSN_3$ (0.20 mmol, 2.0 equiv.) in DCE (0.1 M in regard to alkenes) via syringe under N2. The reaction mixture was placed under 427nm Kessil® light after sealing the punctured holes of the vial cap with vacuum grease and electric tape/parafilm for better air-tight protection and allowed to react at room temperature for 24 h. Following this, the reaction mixture was filtered through a pad of celite which was subsequently rinsed with DCM. The filtrate was concentrated, and the residue was then purified by flash column chromatography or preparatory thin-layer chromatography to give the corresponding bromoazidated products.

II. Supplemental Discussion

2.1 Optimization of Hydroazidation

Supplementary Table 1. Initial testing of hydroazidation.

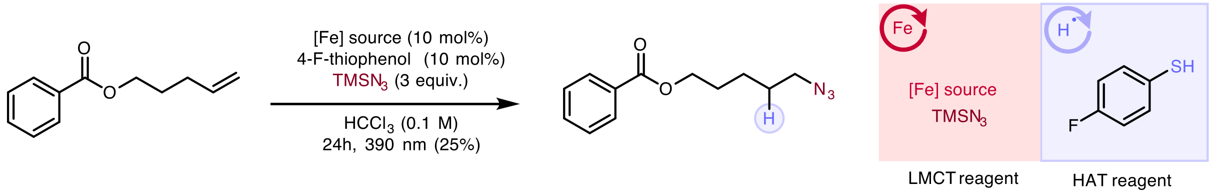

entry	[Fe] source	yield
1	$Fe(NO_3)_3 \cdot 9H_2O$	28
2	$Fe(OAc)_2$	2
3	$FeCl_2$	8
4	$Fe(acac)_3$	4
5	$Fe_2(SO_4)_3 \cdot 5H_2O$	8
6	$FeCl_3 \cdot 6H_2O$	20
7	$Fe(OTf)_3$	22
8[a]	$Fe(NO_3)_3 \cdot 9H_2O$	14 (no D incorporation)

[a] $CDCl_3$ (0.1M).

Supplementary Table 2. Bidentate ligand screening for hydroazidation.

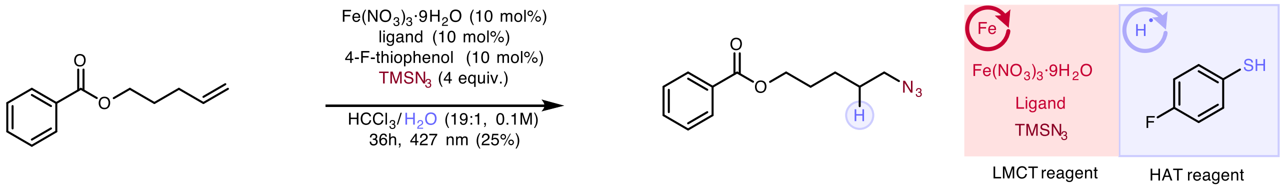

entry	Ligand	yield
1	L1	4
2	L2	12
3	L3	48
4	L4	12
5	L5	ND
6	L6	22
7	L7	16

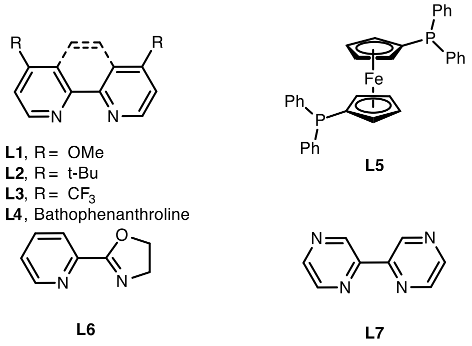

Supplementary Table 3. Tridentate ligand screening for hydroazidation.

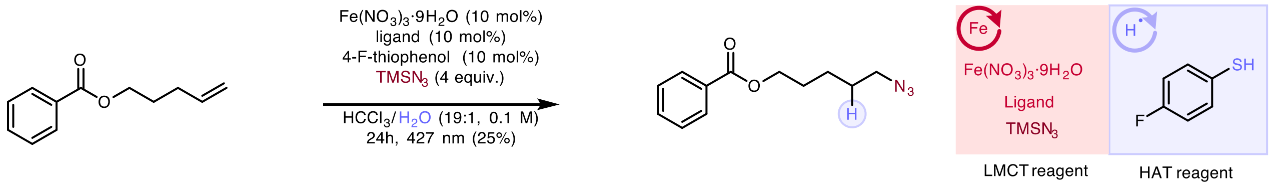

entry	Ligand	yield
1[a]	L1	78 (76)
2	L2	50
3	L3	48
4	L4	4
5	L5	62
6	L6	48
7	L7	52
8[a]	L8	66
9[a]	L9	18

Isolated yield in parentheses. [a] 36h.

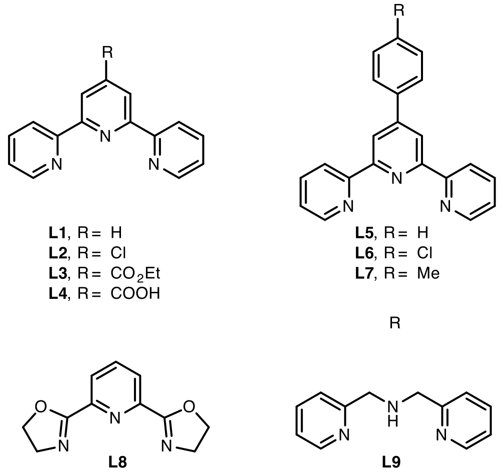

Supplementary Table 4. Solvent screening for hydroazidation.

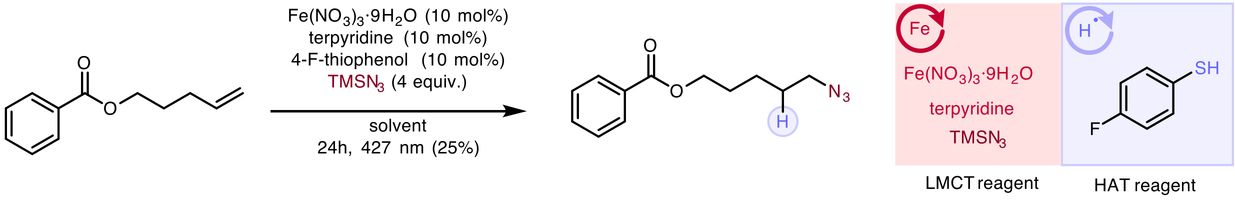

entry	Solvent	yield
1	DCM/H_2O = 19:1 (0.1 M)	44
2	DCE/H_2O = 19:1 (0.1 M)	42
3	THF/H_2O = 19:1 (0.1 M)	2
4	$PhCF_3/H_2O$ = 19:1 (0.1 M)	16
5	EA/H_2O = 19:1 (0.1 M)	12
6[a]	CH_3CN/H_2O = 19:1 (0.1 M)	trace
7	Acetone/H_2O = 19:1 (0.1 M)	8
8	$HCCl_3/H_2O$ = 9:1 (0.1 M)	46
9	$HCCl_3/H_2O$ = 19:1 (0.2 M)	66

[a] With 3.0 equiv. $TMSN_3$ and 2-(but-3-en-1-yl)isoindoline-1,3-dione as substrate. [b] With 3.0 equiv. $TMSN_3$, 48h.

Supplementary Table 5. HAT co-catalyst screening for hydroazidation.

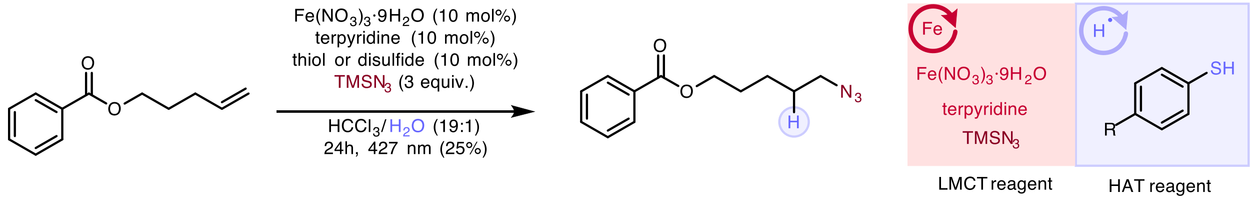

entry	thiol or disulfide HAT co-catalyst	yield
1[a]	triisopropylsilanethiol	trace
2[a]	4-OH-thiophenol	trace
3[a]	4-OMe-thiophenol	trace
4	4-CF_3-thiophenol	52
5	4-F-thiophenol	66[b];34[c]
6	pentafluorothiophenol	44
7	3,5-CF_3-thiophenol	42
8	4-F-phenyldisulfide	66
9	4-NO_2-phenyldisulfide	16
10	2,4,5-Cl-phenyldisulfide	64

[a] With 2-(but-3-en-1-yl)isoindoline-1,3-dione as substrate. [b] 48h. [c] With 10 mol% Na_2CO_3.

Supplementary Table 6. Control experiments of hydroazidation.

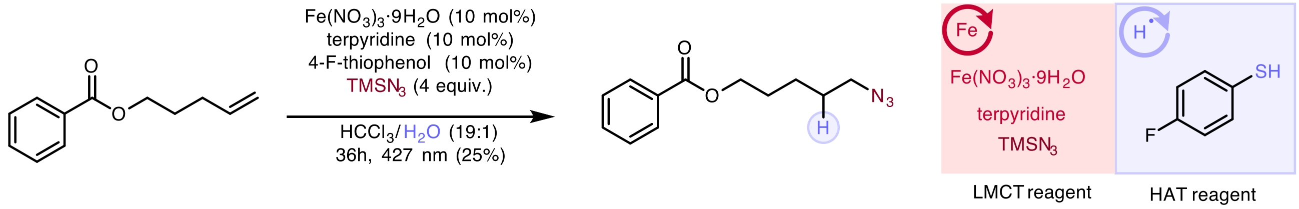

entry	deviation from standard conditions	yield
1	no iron/ligand	ND
2	no thiol	36
3	no light	ND
4	390 nm (25%)	22
5	no ligand	trace
6	30 mol% [Fe], no ligand/thiol	20

2.2 Optimization of Chloroazidation

Supplementary Table 7. Initial testing of chloroazidation.

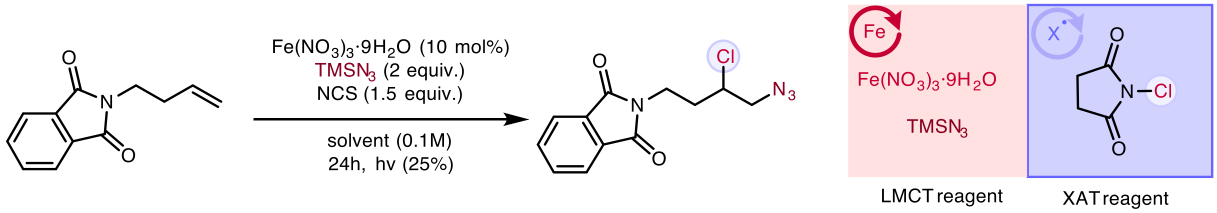

entry	initial testing	yield; di-N_3 yield
1	CH_3CN (0.1M), 390nm	32;28
2	CH_3CN (0.1M), 427nm	52;24
3	DCM (0.1M), 427nm	36;trace

Supplementary Table 8. Ligand effect screening for chloroazidation.

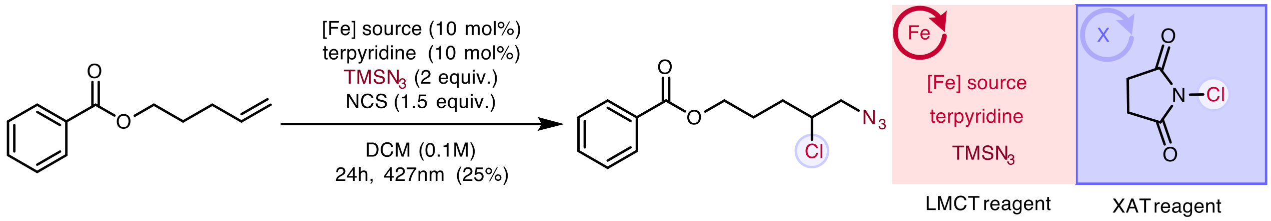

entry	[Fe] source + ligand acceleration	yield; di-N_3 yield
1	$Fe(NO_3)_3 \cdot 9H_2O$ + terpyridine	80;12
2[a]	$Fe(OAc)_2$ + terpyridine	72;trace
3[b]	$Fe(OAc)_2$ + terpyridine	12

[a] Entry 2 is selected for further optimization due to higher mass balance. [b] In the dark.

Supplementary Table 9. Catalyst type/loading and azide amount screening for chloroazidation.

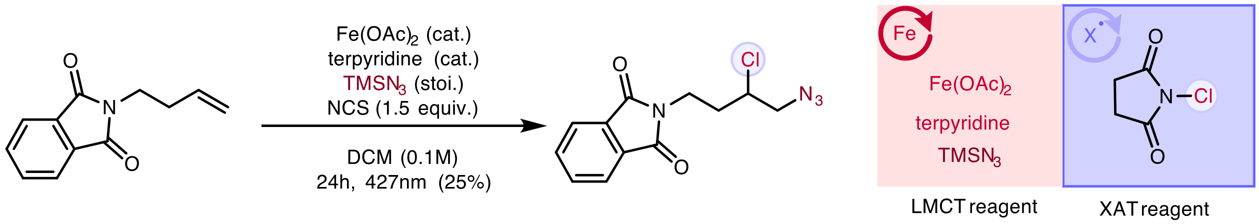

entry	$Fe(OAc)_2$/terpyridine	$TMSN_3$	yield
1	10 mol%	2.0 equiv.	74(72)
2	5 mol%	2.0 equiv.	60
3	10 mol%	1.5 equiv.	64
4[a]	10 mol%	2.0 equiv.	64
5[b]	10 mol%	2.0 equiv.	60

Isolated yield in parentheses. [a] $FeCl_2$. [b] $Fe(OTf)_2$.

Supplementary Table 10. Control experiments of chloroazidation.

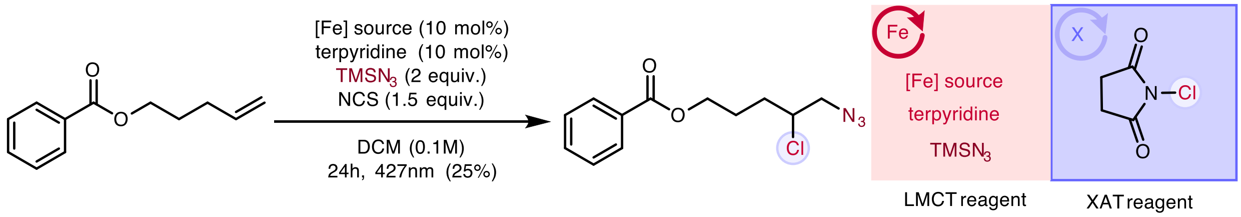

entry	iron salt	ligand	light	yield; di-N_3 yield
1	$Fe(NO_3)_3 \cdot 9H_2O$	terpyridine	427% (25%)	80; 12
2	$Fe(NO_3)_3 \cdot 9H_2O$	terpyridine	dark	48
3	$Fe(NO_3)_3 \cdot 9H_2O$	no ligand	dark	ND
4	$Fe(OAc)_2$	terpyridine	427% (25%)	72; trace
5	$Fe(OAc)_2$	terpyridine	dark	12

2.3 Optimization of Bromoazidation

Supplementary Table 11. Azide and NBS amount screening for bromoazidation.

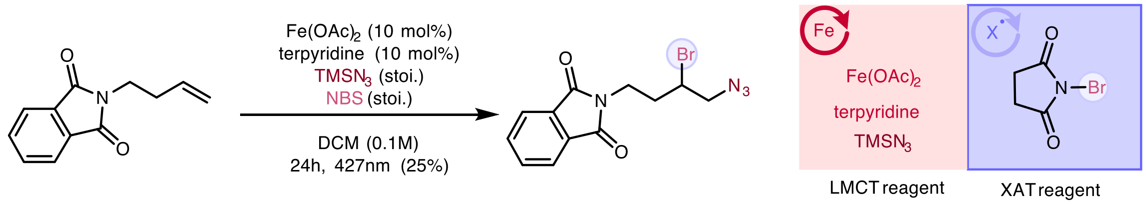

entry	$TMSN_3$	NBS	yield
1	2.0 equiv.	1.2 equiv.	44
2	2.0 equiv.	2.0 equiv.	44
3	1.5 equiv.	2.0 equiv.	48

Supplementary Table 12. Iron screening for bromoazidation.

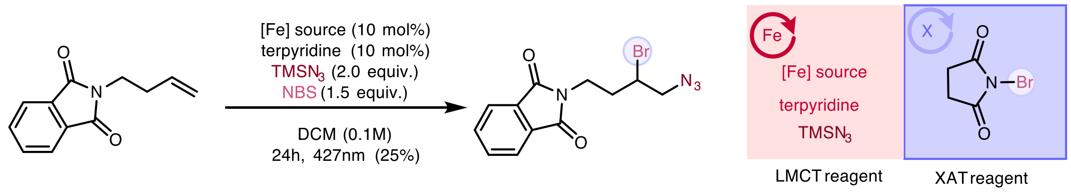

entry	[Fe] source	yield
1	$Fe(NO_3)_3{\cdot}9H_2O$	48
2	$Fe(OTf)_3$	48
3	$Fe(acac)_3$	36
4	$FeCl_3{\cdot}6H_2O$	36
5	$FeCl_2$	44
6	$Fe(OAc)_2$	48
7[a]	$Fe(acac)_2$	44

[a] Entry 7 is selected for further optimization due to higher mass balance.

Supplementary Table 13. Tridentate ligand screening for bromoazidation.

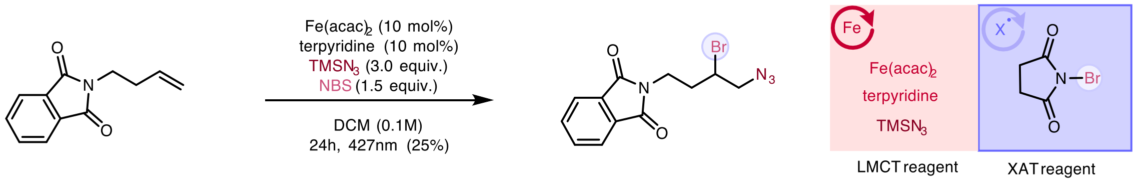

entry	Ligand	yield
1	L1	48
2	L2	24
3	L3	32
4	L4	30
5	L5	32
6	L6	34
7[a]	L1	46
8[a,b]	L1	56 (51)

Isolated yield in parentheses. [a] With 2.0 equiv. $TMSN_3$. [b] With pent-4-en-1-yl benzoate as substrate; DCE was selected due to high mass balance.

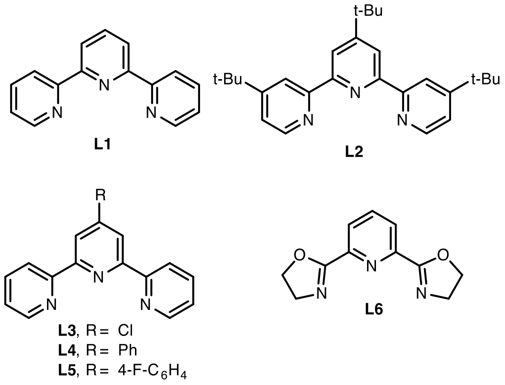

Supplementary Table 14. Control experiments of bromoazidation.

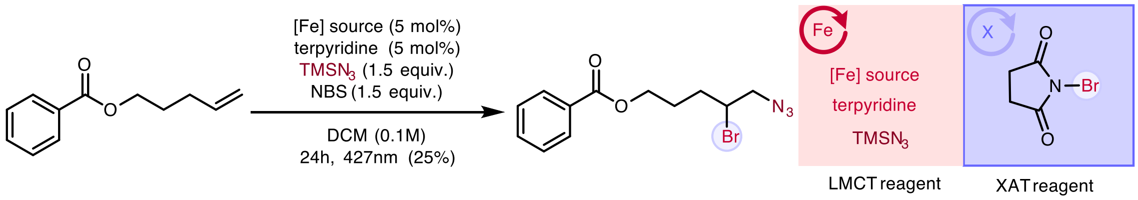

entry	iron salt	ligand	light	yield
1	$Fe(NO_3)_3{\cdot}9H_2O$	terpyridine	427% (25%)	48
2	$Fe(NO_3)_3{\cdot}9H_2O$	terpyridine	dark	68 (4:1 r.r.)
3	no iron	no ligand	427% (25%)	ND
4[a]	$Fe(OAc)_2$	terpyridine	427% (25%)	52
5[a]	$Fe(OAc)_2$	terpyridine	dark	36 (1.3:1 r.r.)

[a] With 10 mol% $Fe(OAc)_2$, ligand and 2.0 equiv. $TMSN_3$.

2.3 Characterization of Corresponding Products

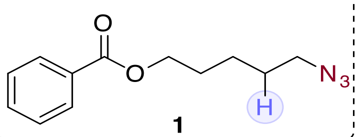

1

Prepared according to General Procedure A (substrate 0.1 mmol scale): $Fe(NO_3)_3{\cdot}9H_2O$ (10 mol%, 0.1 equiv.) and terpyridine (10 mol%, 0.1 equiv.) were added in an oven-dried 8-mL test vial containing a Teflon®-coated magnetic stir bar. The vial was evacuated and backfilled with N2 (repeated for 4 times), followed by addition of alkene (0.1 mmol, 1.0 equiv.), $TMSN_3$ (0.40 mmol, 4.0 equiv.), 4-F-thiolphenol (10 mol%, 0.1 equiv.) in $HCCl_3/H_2O$ (19:1, 0.1 M in regard to alkenes) via syringe under N2. The reaction mixture was placed under 427nm Kessil® light (25%) with proper sealing (see General Procedure A) and allowed to react at room temperature for 36 h. Following this, the reaction mixture was filtered through a pad of celite and rinsed with DCM. The concentrated filtrate was then purified through preparatory thin-layer chromatography (with eluent of Hex: EA = 10:1) to give the corresponding hydroazidation products as colorless oil.

Yield 76%, 17.7 mg, **r.r.** = 12:1 (determined by ^{1}H NMR).

^{1}H NMR (600 MHz, $CDCl_3$) δ 8.11 – 7.97 (m, 2H), 7.62 – 7.50 (m, 1H), 7.49 – 7.38 (m, 2H), 4.33 (t, J = 6.5 Hz, 2H), 3.31 (t, J = 6.8 Hz, 2H), 1.86-1.76 (m, 2H), 1.73-1.65 (m, 2H), 1.59 – 1.51 (m, 2H).

^{13}C NMR (151 MHz, $CDCl_3$) δ 166.62, 132.92, 130.34, 129.55, 128.37, 64.65, 51.28, 28.56, 28.32, 23.36.

HRMS ESI: $[M+H]^+$ calcd. for C12H16N3O2: 234.1237; Found 234.1234

The compound characterization was reported in literature and the NMR data matched with previous characterization.[10]

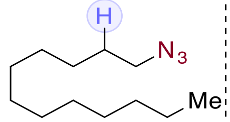

2

Prepared according to General Procedure A (substrate 0.1 mmol scale): $Fe(NO_3)_3{\cdot}9H_2O$ (10 mol%, 0.1 equiv.) and terpyridine (10 mol%, 0.1 equiv.) were added in an oven-dried 8-mL test vial containing a Teflon®-coated magnetic stir bar. The vial was evacuated and backfilled with N2 (repeated for 4 times), followed by addition of alkene (0.1 mmol, 1.0 equiv.), $TMSN_3$ (0.40 mmol, 4.0 equiv.), 4-F-thiolphenol (10 mol%, 0.1 equiv.) in $HCCl_3/H_2O$ (19:1, 0.1 M in regard to alkenes) via syringe under N2. The reaction mixture was placed under 427nm Kessil® light (50%) with proper sealing (see General Procedure A) and allowed to react at room temperature for 60 h. Following this, the reaction mixture was filtered through a pad of celite and rinsed with DCM. The concentrated filtrate was then purified through column chromatography (with eluent of Hexane) to give the corresponding hydroazidation products as colorless oil.

Yield 56%, 11.8 mg, **r.r.** = 10:1 (determined by ^{1}H NMR).

^{1}H NMR (600 MHz, $CDCl_3$) δ 3.30-3.20 (m, 2H), 1.61 – 1.56 (m, 2H), 1.39 – 1.24 (m, 18H), 0.91 – 0.85 (m, 3H).

^{13}C NMR (151 MHz, $CDCl_3$) ^{13}C NMR (151 MHz, $CDCl_3$) δ 51.51, 31.92, 29.63, 29.56, 29.50, 29.35, 29.17, 28.85, 26.73, 22.70, 14.12.

HRMS APCI: $[M-N2+H]^+$ calcd. for C12H26N: 184.2060; Found 184.2057

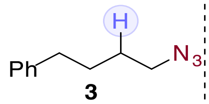

3

Prepared according to General Procedure A (substrate 0.1 mmol scale): $Fe(NO_3)_3{\cdot}9H_2O$ (10 mol%, 0.1 equiv.) and terpyridine (10 mol%, 0.1 equiv.) were added in an oven-dried 8-mL test vial containing a Teflon®-coated magnetic stir bar. The vial was evacuated and backfilled with N2 (repeated for 4 times), followed by addition of alkene (0.1 mmol, 1.0 equiv.), $TMSN_3$ (0.40 mmol, 4.0 equiv.), 4-F-thiolphenol (10 mol%, 0.1 equiv.) in $HCCl_3/H_2O$ (19:1, 0.1 M in regard to alkenes) via syringe under N2. The reaction mixture was placed under 427nm Kessil® light (25%) with proper sealing (see General Procedure A) and allowed to react at room temperature for 36 h. Following this, the reaction mixture was filtered through a pad of celite and rinsed with DCM. The concentrated filtrate was then purified through preparatory thin-layer chromatography (with eluent of Hex: EA = 20:1) to give the corresponding hydroazidation products as colorless oil.

Yield 62%, 10.9 mg, **r.r.** = 14:1 (determined by ^{1}H NMR).
Properties colorless liquid.
^{1}H NMR (600 MHz, CDCl$_3$) δ 7.33-7.27 (m, 2H), 7.23-7.16 (m, 3H), 3.29 (t, *J* = 6.8 Hz, 2H), 2.66 (t, *J* = 7.6 Hz, 2H), 1.76-1.69 (m, 2H), 1.68 – 1.61 (m, 2H).
^{13}C NMR (151 MHz, CDCl$_3$) δ 141.82, 128.39, 128.37, 125.91, 51.34, 35.36, 28.44.
HRMS APCI: [M-N2+H]$^+$ calcd. for C10H14N: 148.1121; Found 148.1118

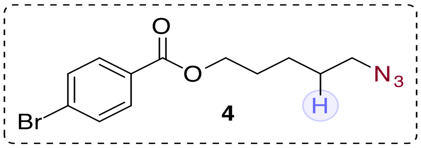

Prepared according to General Procedure A (substrate 0.1 mmol scale): $Fe(NO_3)_3 \cdot 9H_2O$ (10 mol%, 0.1 equiv.) and terpyridine (10 mol%, 0.1 equiv.) were added in an oven-dried 8-mL test vial containing a Teflon®-coated magnetic stir bar. The vial was evacuated and backfilled with N2 (repeated for 4 times), followed by addition of alkene (0.1 mmol, 1.0 equiv.), $TMSN_3$ (0.40 mmol, 4.0 equiv.), 4-F-thiolphenol (10 mol%, 0.1 equiv.) in $HCCl_3/H_2O$ (19:1, 0.1 M in regard to alkenes) via syringe under N2. The reaction mixture was placed under 427nm Kessil® light (25%) with proper sealing (see General Procedure A) and allowed to react at room temperature for 36 h. Following this, the reaction mixture was filtered through a pad of celite and rinsed with DCM. The concentrated filtrate was then purified through preparatory thin-layer chromatography (with eluent of Hex: EA = 10:1) to give the corresponding hydroazidation products as colorless oil.
Yield 75%, 23.4 mg, **r.r.** = 13:1 (determined by ^{1}H NMR).
^{1}H NMR (600 MHz, CDCl$_3$) δ 7.93 – 7.85 (m, 2H), 7.61 – 7.54 (m, 2H), 4.32 (t, *J* = 6.5 Hz, 2H), 3.30 (t, *J* = 6.8 Hz, 2H), 1.85 – 1.75 (m, 2H), 1.71-1.63 (m, 2H), 1.59 – 1.49 (m, 2H).
^{13}C NMR (151 MHz, CDCl$_3$) δ 165.88, 131.73, 131.10, 129.23, 128.04, 64.93, 51.26, 28.55, 28.28, 23.33.
HRMS ESI: [M+H]$^+$ calcd. for C12H15BrN3O2: 312.0342; Found 312.0338

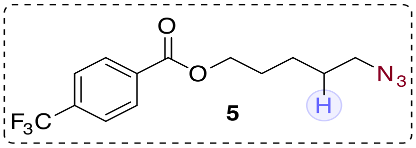

Prepared according to General Procedure A (substrate 0.1 mmol scale): $Fe(NO_3)_3 \cdot 9H_2O$ (10 mol%, 0.1 equiv.) and terpyridine (10 mol%, 0.1 equiv.) were added in an oven-dried 8-mL test vial containing a Teflon®-coated magnetic stir bar. The vial was evacuated and backfilled with N2 (repeated for 4 times), followed by addition of alkene (0.1 mmol, 1.0 equiv.), $TMSN_3$ (0.40 mmol, 4.0 equiv.), 4-F-thiolphenol (10 mol%, 0.1 equiv.) in $HCCl_3/H_2O$ (19:1, 0.1 M in regard to alkenes) via syringe under N2. The reaction mixture was placed under 427nm Kessil® light (25%) with proper sealing (see General Procedure A) and allowed to react at room temperature for 36 h. Following this, the reaction mixture was filtered through a pad of celite and rinsed with DCM. The concentrated filtrate was then purified through preparatory thin-layer chromatography (with eluent of Hex: EA = 10:1) to give the corresponding hydroazidation products as colorless oil.
Yield 55%, 16.0 mg, **r.r.** = 12:1 (determined by ^{1}H NMR).
^{1}H NMR (600 MHz, CDCl$_3$) δ 8.15 (d, *J* = 8.0 Hz, 2H), 7.71 (d, *J* = 8.2 Hz, 2H), 4.37 (t, *J* = 6.6 Hz, 2H), 3.31 (t, *J* = 6.8 Hz, 2H), 1.87 – 1.78 (m, 2H), 1.72 – 1.65 (m, 2H), 1.58 – 1.51 (m, 2H).
^{13}C NMR (151 MHz, CDCl$_3$) δ 165.40, 134.44 (q, *J* = 32.7 Hz), 133.54, 129.96, 125.43 (q, *J* = 3.7 Hz), 123.64 (q, *J* = 272.8 Hz), 65.23, 51.24, 28.55, 28.25, 23.32.
^{19}F NMR (564 MHz, CDCl$_3$) δ -63.11 (s).
HRMS APCI: [M-N2+H]$^+$ calcd. for C13H15F3NO2: 274.1049; Found 274.1043

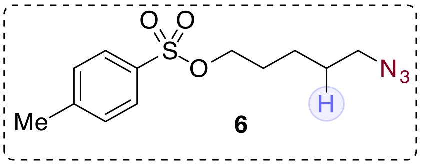

Prepared according to General Procedure A (substrate 0.1 mmol scale): $Fe(NO_3)_3 \cdot 9H_2O$ (10 mol%, 0.1 equiv.) and terpyridine (10 mol%, 0.1 equiv.) were added in an oven-dried 8-mL test vial containing a Teflon®-coated magnetic stir bar. The vial was evacuated and backfilled with N2 (repeated for 4

times), followed by addition of alkene (0.1 mmol, 1.0 equiv.), $TMSN_3$ (0.40 mmol, 4.0 equiv.), 4-F-thiolphenol (10 mol%, 0.1 equiv.) in $HCCl_3/H_2O$ (19:1, 0.1 M in regard to alkenes) via syringe under N2. The reaction mixture was placed under 427nm Kessil® light (25%) with proper sealing (see General Procedure A) and allowed to react at room temperature for 36 h. Following this, the reaction mixture was filtered through a pad of celite and rinsed with DCM. The concentrated filtrate was then purified through preparatory thin-layer chromatography (with eluent of Hex: EA = 10:1) to give the corresponding hydroazidation products as colorless oil.
Yield 66%, 18.7 mg, **r.r.** = 13:1 (determined by ^{1}H NMR).
Properties colorless liquid.
^{1}H NMR (600 MHz, $CDCl_3$) δ 7.78 (d, J = 8.0 Hz, 2H), 7.35 (d, J = 7.9 Hz, 2H), 4.03 (t, J = 6.3 Hz, 2H), 3.23 (t, J = 6.8 Hz, 2H), 2.45 (s, 3H), 1.72 – 1.63 (m, 2H), 1.54 (p, J = 7.0 Hz, 2H), 1.44 – 1.36 (m, 2H).
^{13}C NMR (151 MHz, $CDCl_3$) δ 144.82, 133.06, 129.87, 127.88, 70.12, 51.13, 28.40, 28.22, 22.70, 21.64.
HRMS ESI: $[M+H]^+$ calcd. for C12H18N3O3S: 284.1063; Found 284.1058

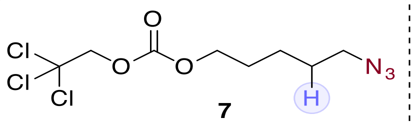

7

Prepared according to General Procedure A (substrate 0.1 mmol scale): $Fe(NO_3)_3{\cdot}9H_2O$ (10 mol%, 0.1 equiv.) and terpyridine (10 mol%, 0.1 equiv.) were added in an oven-dried 8-mL test vial containing a Teflon®-coated magnetic stir bar. The vial was evacuated and backfilled with N2 (repeated for 4 times), followed by addition of alkene (0.1 mmol, 1.0 equiv.), $TMSN_3$ (0.40 mmol, 4.0 equiv.), 4-F-thiolphenol (10 mol%, 0.1 equiv.) in $HCCl_3/H_2O$ (19:1, 0.1 M in regard to alkenes) via syringe under N2. The reaction mixture was placed under 427nm Kessil® light (25%) with proper sealing (see General Procedure A) and allowed to react at room temperature for 60 h. Following this, the reaction mixture was filtered through a pad of celite and rinsed with DCM. The concentrated filtrate was then purified through column chromatography (with eluent of Hex: EA = 10:1) to give the corresponding hydroazidation products as colorless oil.
Yield 65%, 19.8 mg, **r.r.** = 11:1 (determined by ^{1}H NMR).
Properties colorless liquid.
^{1}H NMR (600 MHz, $CDCl_3$) δ 4.77 (s, 2H), 4.24 (t, J = 6.6 Hz, 2H), 3.29 (t, J = 6.8 Hz, 2H), 1.79 – 1.73 (m, 2H), 1.68-1.61 (m, 2H), 1.52 – 1.46 (m, 2H).
^{13}C NMR (151 MHz, $CDCl_3$) δ 154.03, 94.46, 76.75, 68.88, 51.21, 28.46, 28.13, 22.94.
HRMS ESI: $[M+H]^+$ calcd. for C8H13Cl3N3O3: 304.0017; Found 304.0013

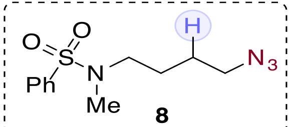

8

Prepared according to General Procedure A (substrate 0.1 mmol scale): $Fe(NO_3)_3{\cdot}9H_2O$ (10 mol%, 0.1 equiv.) and terpyridine (10 mol%, 0.1 equiv.) were added in an oven-dried 8-mL test vial containing a Teflon®-coated magnetic stir bar. The vial was evacuated and backfilled with N2 (repeated for 4 times), followed by addition of alkene (0.1 mmol, 1.0 equiv.), $TMSN_3$ (0.40 mmol, 4.0 equiv.), 4-F-thiolphenol (10 mol%, 0.1 equiv.) in $HCCl_3/H_2O$ (19:1, 0.1 M in regard to alkenes) via syringe under N2. The reaction mixture was placed under 427nm Kessil® light (25%) with proper sealing (see General Procedure A) and allowed to react at room temperature for 36 h. Following this, the reaction mixture was filtered through a pad of celite and rinsed with DCM. The concentrated filtrate was then purified through preparatory thin-layer chromatography (with eluent of Hex: EA = 10:1) to give the corresponding hydroazidation products as colorless oil.
Yield 56%, 15.0 mg, **r.r.** = 13:1 (determined by ^{1}H NMR).
^{1}H NMR (600 MHz, $CDCl_3$) δ 7.81 – 7.75 (m, 2H), 7.62 – 7.57 (m, 1H), 7.56 – 7.50 (m, 2H), 3.33 (t, J = 6.3 Hz, 2H), 3.03 (t, J = 6.6 Hz, 2H), 2.72 (s, 3H), 1.70 – 1.60 (m, 4H).
^{13}C NMR (151 MHz, $CDCl_3$) δ 137.40, 132.62, 129.10, 127.33, 50.85, 49.39, 34.58, 25.76, 24.57.
HRMS ESI: $[M+H]^+$ calcd. for C11H17N4O2S: 269.1067; Found 269.1061

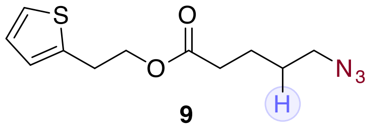

9

Prepared according to General Procedure A (substrate 0.1 mmol scale): $Fe(NO_3)_3·9H_2O$ (10 mol%, 0.1 equiv.) and terpyridine (10 mol%, 0.1 equiv.) were added in an oven-dried 8-mL test vial containing a Teflon®-coated magnetic stir bar. The vial was evacuated and backfilled with N2 (repeated for 4 times), followed by addition of alkene (0.1 mmol, 1.0 equiv.), $TMSN_3$ (0.40 mmol, 4.0 equiv.), 4-F-thiolphenol (10 mol%, 0.1 equiv.) in $HCCl_3/H_2O$ (19:1, 0.1 M in regard to alkenes) via syringe under N2. The reaction mixture was placed under 427nm Kessil® light (25%) with proper sealing (see General Procedure A) and allowed to react at room temperature for 36 h. Following this, the reaction mixture was filtered through a pad of celite and rinsed with DCM. The concentrated filtrate was then purified through preparatory thin-layer chromatography (with eluent of Hex: EA = 10:1) to give the corresponding hydroazidation products as colorless oil.

Yield 43%, 10.9 mg, **r.r.** = 16:1 (determined by ^{1}H NMR).

^{1}H NMR (600 MHz, $CDCl_3$) δ 7.16 (dd, J = 5.1, 1.2 Hz, 1H), 6.94 (dd, J = 5.1, 3.4 Hz, 1H), 6.86 (dd, J = 3.4, 1.1 Hz, 1H), 4.31 (t, J = 6.7 Hz, 2H), 3.28 (t, J = 6.8 Hz, 2H), 3.16 (t, J = 6.8 Hz, 2H), 2.36 (t, J = 7.3 Hz, 2H), 1.75 – 1.67 (m, 2H), 1.63 – 1.59 (m, 2H).

^{13}C NMR (151 MHz, $CDCl_3$) δ 172.99, 139.92, 126.88, 125.54, 124.05, 64.62, 51.05, 33.64, 29.32, 28.25, 22.06.

HRMS ESI: $[M+H]^+$ calcd. for C11H16N3O2S: 254.0958; Found 254.0953

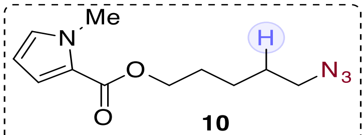

10

Prepared according to General Procedure A (substrate 0.1 mmol scale): $Fe(NO_3)_3·9H_2O$ (10 mol%, 0.1 equiv.) and terpyridine (10 mol%, 0.1 equiv.) were added in an oven-dried 8-mL test vial containing a Teflon®-coated magnetic stir bar. The vial was evacuated and backfilled with N2 (repeated for 4 times), followed by addition of alkene (0.1 mmol, 1.0 equiv.), $TMSN_3$ (0.40 mmol, 4.0 equiv.), 4-F-thiolphenol (10 mol%, 0.1 equiv.) in $HCCl_3/H_2O$ (19:1, 0.1 M in regard to alkenes) via syringe under N2. The reaction mixture was placed under 427nm Kessil® light (50%) with proper sealing (see General Procedure A) and allowed to react at room temperature for 60 h. Following this, the reaction mixture was filtered through a pad of celite and rinsed with DCM. The concentrated filtrate was then purified through preparatory thin-layer chromatography (with eluent of Hex: EA = 10:1) to give the corresponding hydroazidation products as colorless oil.

Yield 34%, 8.0 mg, **r.r.** = 12:1 (determined by ^{1}H NMR).

Properties colorless liquid.

^{1}H NMR (600 MHz, $CDCl_3$) δ 6.94 (dd, J = 4.0, 1.8 Hz, 1H), 6.78 (t, J = 2.2 Hz, 1H), 6.11 (dd, J = 4.0, 2.5 Hz, 1H), 4.23 (t, J = 6.5 Hz, 2H), 3.92 (s, 3H), 3.30 (t, J = 6.9 Hz, 2H), 1.79-1.73 (m, 2H), 1.70 – 1.64 (m, 2H), 1.55 – 1.48 (m, 2H).

^{13}C NMR (151 MHz, $CDCl_3$) δ 161.33, 129.49, 122.53, 117.73, 107.82, 63.43, 51.30, 36.82, 28.55, 28.42, 23.37.

HRMS ESI: $[M+H]^+$ calcd. for C11H17N4O2: 237.1346; Found 237.1342

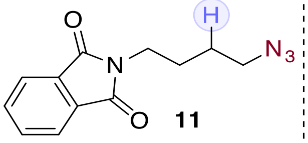

11

Prepared according to General Procedure A (substrate 0.1 mmol scale): $Fe(NO_3)_3·9H_2O$ (10 mol%, 0.1 equiv.), terpyridine (10 mol%, 0.1 equiv.) and alkene (0.1 mmol, 1.0 equiv.) were added in an oven-dried 8-mL test vial containing a Teflon®-coated magnetic stir bar. The vial was evacuated and backfilled with N2 (repeated for 4 times), followed by addition of $TMSN_3$ (0.40 mmol, 4.0 equiv.), 4-F-thiolphenol (10 mol%, 0.1 equiv.) in $HCCl_3/H_2O$ (19:1, 0.1 M in regard to alkenes) via syringe under N2. The reaction mixture was placed under 427nm Kessil® light (50%) with proper sealing (see General Procedure A) and allowed to react at room temperature for 60 h. Following this, the reaction mixture was filtered through a pad of celite and rinsed with DCM. The concentrated filtrate was then purified through preparatory thin-layer chromatography (with eluent of Hex: EA = 5:1) to give the corresponding hydroazidation products as colorless oil.

Yield 64%, 15.6 mg, **r.r.** = 13:1 (determined by ^{1}H NMR).
^{1}H NMR (600 MHz, CDCl$_3$) δ 7.84 (dd, *J* = 5.4, 3.1 Hz, 2H), 7.72 (dd, *J* = 5.4, 3.0 Hz, 2H), 3.72 (t, *J* = 7.1 Hz, 2H), 3.33 (t, *J* = 6.8 Hz, 2H), 1.80-1.74 (m, 2H), 1.67 – 1.62 (m, 2H).
^{13}C NMR (151 MHz, CDCl$_3$) δ 168.40, 134.01, 132.06, 123.28, 50.91, 37.29, 26.26, 25.85.
HRMS ESI: [M+H]$^+$ calcd. for C12H13N4O2: 245.1033; Found 245.1030

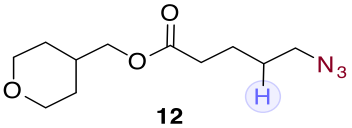

12

Prepared according to General Procedure A (substrate 0.1 mmol scale): $Fe(NO_3)_3\cdot 9H_2O$ (10 mol%, 0.1 equiv.) and terpyridine (10 mol%, 0.1 equiv.) were added in an oven-dried 8-mL test vial containing a Teflon®-coated magnetic stir bar. The vial was evacuated and backfilled with N2 (repeated for 4 times), followed by addition of alkene (0.1 mmol, 1.0 equiv.), $TMSN_3$ (0.40 mmol, 4.0 equiv.), 4-F-thiolphenol (10 mol%, 0.1 equiv.) in $HCCl_3/H_2O$ (19:1, 0.1 M in regard to alkenes) via syringe under N2. The reaction mixture was placed under 427nm Kessil® light (25%) with proper sealing (see General Procedure A) and allowed to react at room temperature for 36 h. Following this, the reaction mixture was filtered through a pad of celite and rinsed with DCM. The concentrated filtrate was then purified through column chromatography (with eluent of Hex: EA = 10:1) to give the corresponding hydroazidation products as colorless oil.
Yield 61%, 14.7 mg, **r.r.** = 9:1 (at least, determined by ^{1}H NMR).
Properties colorless liquid.
^{1}H NMR (600 MHz, CDCl$_3$) δ 4.02 – 3.91 (m, 4H), 3.43-3.34 (m, 2H), 3.29 (t, *J* = 6.7 Hz, 2H), 2.35 (t, *J* = 7.3 Hz, 2H), 1.94-1.85 (m, 1H), 1.77 – 1.69 (m, 2H), 1.66 – 1.60 (m, 4H), 1.41-1.34 (m, 2H).
^{13}C NMR (151 MHz, CDCl$_3$) δ 173.15, 68.68, 67.47, 51.05, 34.52, 33.62, 29.52, 28.31, 22.14.
HRMS ESI: [M+H]$^+$ calcd. for C11H20N3O3: 242.1499; Found 242.1495

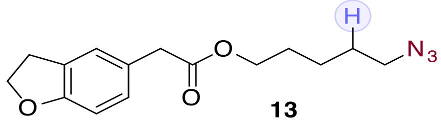

13

Prepared according to General Procedure A (substrate 0.1 mmol scale): $Fe(NO_3)_3\cdot 9H_2O$ (10 mol%, 0.1 equiv.) and terpyridine (10 mol%, 0.1 equiv.) were added in an oven-dried 8-mL test vial containing a Teflon®-coated magnetic stir bar. The vial was evacuated and backfilled with N2 (repeated for 4 times), followed by addition of alkene (0.1 mmol, 1.0 equiv.), $TMSN_3$ (0.40 mmol, 4.0 equiv.), 4-F-thiolphenol (10 mol%, 0.1 equiv.) in $HCCl_3/H_2O$ (19:1, 0.1 M in regard to alkenes) via syringe under N2. The reaction mixture was placed under 427nm Kessil® light (50%) with proper sealing (see General Procedure A) and allowed to react at room temperature for 60 h. Following this, the reaction mixture was filtered through a pad of celite and rinsed with DCM. The concentrated filtrate was then purified through preparatory thin-layer chromatography (with eluent of Hex: EA = 8:1) to give the corresponding hydroazidation products as colorless oil.
Yield 45%, 13.0 mg, **r.r.** = 13:1 (determined by ^{1}H NMR).
Properties colorless liquid.
^{1}H NMR (600 MHz, CDCl$_3$) δ 7.12 (d, *J* = 1.9 Hz, 1H), 6.99 (dd, *J* = 8.1, 1.9 Hz, 1H), 6.72 (d, *J* = 8.1 Hz, 1H), 4.55 (t, *J* = 8.7 Hz, 2H), 4.09 (t, *J* = 6.5 Hz, 2H), 3.53 (s, 2H), 3.25 (t, *J* = 6.9 Hz, 2H), 3.19 (t, *J* = 8.7 Hz, 2H), 1.68-1.62 (m, 2H), 1.61 – 1.56 (m, 2H), 1.44 – 1.36 (m, 2H).
^{13}C NMR (151 MHz, CDCl$_3$) δ 172.18, 159.26, 128.86, 127.39, 125.93, 125.81, 109.18, 71.31, 64.47, 51.27, 40.77, 29.71, 28.47, 28.14, 23.17.
HRMS ESI: [M+H]$^+$ calcd. for C15H20N3O3: 290.1499; Found 290.1492

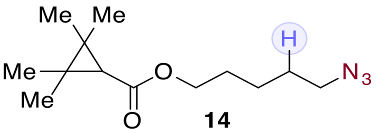

14

Prepared according to General Procedure A (substrate 0.1 mmol scale): $Fe(NO_3)_3\cdot 9H_2O$ (10 mol%, 0.1 equiv.) and terpyridine (10 mol%, 0.1 equiv.) were added in an oven-dried 8-mL test

vial containing a Teflon®-coated magnetic stir bar. The vial was evacuated and backfilled with N2 (repeated for 4 times), followed by addition of alkene (0.1 mmol, 1.0 equiv.), $TMSN_3$ (0.40 mmol, 4.0 equiv.), 4-F-thiolphenol (10 mol%, 0.1 equiv.) in $HCCl_3/H_2O$ (19:1, 0.1 M in regard to alkenes) via syringe under N2. The reaction mixture was placed under 427nm Kessil® light (25%) with proper sealing (see General Procedure A) and allowed to react at room temperature for 36 h. Following this, the reaction mixture was filtered through a pad of celite and rinsed with DCM. The concentrated filtrate was then purified through column chromatography (with eluent of Hex: EA = 20:1) to give the corresponding hydroazidation products as colorless oil.
Yield 70%, 17.7 mg, **r.r.** = 14:1 (determined by ^{1}H NMR).
^{1}H NMR (600 MHz, $CDCl_3$) δ 4.03 (t, J = 6.6 Hz, 2H), 3.28 (t, J = 6.9 Hz, 2H), 1.69 – 1.61 (m, 4H), 1.48 – 1.41 (m, 2H), 1.23 (s, 6H), 1.18-1.16 (m, 7H).
^{13}C NMR (151 MHz, $CDCl_3$) δ 172.26, 63.32, 51.31, 35.75, 30.02, 28.55, 28.36, 23.55, 23.33, 16.59.
HRMS ESI: $[M+H]^+$ calcd. for C13H24N3O2: 254.1863; Found 254.1858

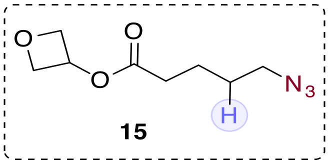

15

Prepared according to General Procedure A (substrate 0.1 mmol scale): $Fe(NO_3)_3·9H_2O$ (10 mol%, 0.1 equiv.) and terpyridine (10 mol%, 0.1 equiv.) were added in an oven-dried 8-mL test vial containing a Teflon®-coated magnetic stir bar. The vial was evacuated and backfilled with N2 (repeated for 4 times), followed by addition of alkene (0.1 mmol, 1.0 equiv.), $TMSN_3$ (0.40 mmol, 4.0 equiv.), 4-F-thiolphenol (10 mol%, 0.1 equiv.) in $HCCl_3/H_2O$ (19:1, 0.1 M in regard to alkenes) via syringe under N2. The reaction mixture was placed under 427nm Kessil® light (25%) with proper sealing (see General Procedure A) and allowed to react at room temperature for 36 h. Following this, the reaction mixture was filtered through a pad of celite and rinsed with DCM. The concentrated filtrate was then purified through column chromatography (with eluent of Hex: EA = 10:1) to give the corresponding hydroazidation products as colorless oil.
Yield 65%, 12.9 mg, **r.r.** = 14:1 (determined by ^{1}H NMR).
^{1}H NMR (600 MHz, $CDCl_3$) δ 5.43 (tt, J = 6.4, 5.3 Hz, 1H), 4.92-4.86 (m, 2H), 4.65-4.58 (m, 2H), 3.31 (t, J = 6.7 Hz, 2H), 2.40 (t, J = 7.3 Hz, 2H), 1.78 – 1.69 (m, 2H), 1.69 – 1.58 (m, 2H).
^{13}C NMR (151 MHz, $CDCl_3$) δ 172.41, 77.57, 67.90, 51.00, 33.31, 28.23, 21.96.
HRMS ESI: $[M+H]^+$ calcd. for C8H14N3O3: 200.1030; Found 200.1026

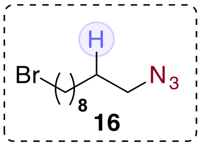

16

Prepared according to General Procedure A (substrate 0.1 mmol scale): $Fe(NO_3)_3·9H_2O$ (10 mol%, 0.1 equiv.) and terpyridine (10 mol%, 0.1 equiv.) were added in an oven-dried 8-mL test vial containing a Teflon®-coated magnetic stir bar. The vial was evacuated and backfilled with N2 (repeated for 4 times), followed by addition of alkene (0.1 mmol, 1.0 equiv.), $TMSN_3$ (0.40 mmol, 4.0 equiv.), 4-F-thiolphenol (10 mol%, 0.1 equiv.) in $HCCl_3/H_2O$ (19:1, 0.1 M in regard to alkenes) via syringe under N2. The reaction mixture was placed under 427nm Kessil® light (25%) with proper sealing (see General Procedure A) and allowed to react at room temperature for 36 h. Following this, the reaction mixture was filtered through a pad of celite and rinsed with DCM. The concentrated filtrate was then purified through column chromatography (with eluent of Hex: EA = 20:1) to give the corresponding hydroazidation products as colorless oil.
Yield 72%, 18.9 mg, **r.r.** = 12:1 (determined by ^{1}H NMR).
^{1}H NMR (600 MHz, $CDCl_3$) δ 3.40 (t, J = 6.9 Hz, 2H), 3.25 (t, J = 7.0 Hz, 2H), 1.89-1.81 (m, 2H), 1.59 (p, J = 7.0 Hz, 2H), 1.45 – 1.40 (m, 2H), 1.38 – 1.29 (m, 10H).
^{13}C NMR (151 MHz, $CDCl_3$) δ 51.47, 34.03, 32.80, 29.35, 29.31, 29.09, 28.83, 28.71, 28.14, 26.69.
HRMS APCI: $[M-N2+H]^+$ calcd. for C10H21BrN: 234.0852; Found 234.0849

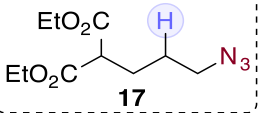

17

Prepared according to General Procedure A (substrate 0.1 mmol scale): $Fe(NO_3)_3{\cdot}9H_2O$ (10 mol%, 0.1 equiv.) and terpyridine (10 mol%, 0.1 equiv.) were added in an oven-dried 8-mL test vial containing a Teflon®-coated magnetic stir bar. The vial was evacuated and backfilled with N2 (repeated for 4 times), followed by addition of alkene (0.1 mmol, 1.0 equiv.), $TMSN_3$ (0.40 mmol, 4.0 equiv.), 4-F-thiolphenol (10 mol%, 0.1 equiv.) in $HCCl_3/H_2O$ (19:1, 0.1 M in regard to alkenes) via syringe under N2. The reaction mixture was placed under 427nm Kessil® light (25%) with proper sealing (see General Procedure A) and allowed to react at room temperature for 36 h. Following this, the reaction mixture was filtered through a pad of celite and rinsed with DCM. The concentrated filtrate was then purified through column chromatography (with eluent of Hex: EA = 10:1) to give the corresponding hydroazidation products as colorless oil.
Yield 41%, 10.0 mg, **r.r.** = 8:1 (at least, determined by ^{1}H NMR).
^{1}H NMR (600 MHz, $CDCl_3$) δ 4.20 (qt, *J* = 7.2, 3.5 Hz, 4H), 3.37-3.27 (m, 3H), 1.97 (m, 2H), 1.64 (p, *J* = 7.1 Hz, 2H), 1.27 (t, *J* = 7.1 Hz, 6H).
^{13}C NMR (151 MHz, $CDCl_3$) δ 169.10, 61.52, 51.46, 50.95, 26.62, 25.92, 14.07.
HRMS ESI: $[M+H]^+$ calcd. for C10H18N3O4: 244.1292; Found 244.1288

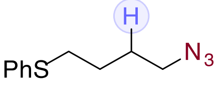

18

Prepared according to General Procedure A (substrate 0.1 mmol scale): $Fe(NO_3)_3{\cdot}9H_2O$ (10 mol%, 0.1 equiv.) and terpyridine (10 mol%, 0.1 equiv.) were added in an oven-dried 8-mL test vial containing a Teflon®-coated magnetic stir bar. The vial was evacuated and backfilled with N2 (repeated for 4 times), followed by addition of alkene (0.1 mmol, 1.0 equiv.), $TMSN_3$ (0.40 mmol, 4.0 equiv.), 4-F-thiolphenol (10 mol%, 0.1 equiv.) in $HCCl_3/H_2O$ (19:1, 0.1 M in regard to alkenes) via syringe under N2. The reaction mixture was placed under 427nm Kessil® light (25%) with proper sealing (see General Procedure A) and allowed to react at room temperature for 60 h. Following this, the reaction mixture was filtered through a pad of celite and rinsed with DCM. The concentrated filtrate was then purified through preparatory thin-layer chromatography (with eluent of Hex: EA = 10:1) to give the corresponding hydroazidation products as colorless oil.
Yield 52%, 10.8 mg, **r.r.** = 12:1 (determined by ^{1}H NMR).
^{1}H NMR (600 MHz, $CDCl_3$) δ 7.36 – 7.27 (m, 4H), 7.22-7.15 (m, 1H), 3.35 – 3.22 (m, 2H), 3.00 – 2.88 (m, 2H), 1.81-1.66 (m, 4H).
^{13}C NMR (151 MHz, $CDCl_3$) δ 136.24, 129.36, 128.96, 126.09, 50.98, 33.28, 27.89, 26.29.
HRMS APCI: $[M-N2+H]^+$ calcd. for C10H14NS: 180.0841; Found 180.0839

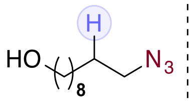

19

Prepared according to General Procedure A (substrate 0.1 mmol scale): $Fe(NO_3)_3{\cdot}9H_2O$ (10 mol%, 0.1 equiv.) and terpyridine (10 mol%, 0.1 equiv.) were added in an oven-dried 8-mL test vial containing a Teflon®-coated magnetic stir bar. The vial was evacuated and backfilled with N2 (repeated for 4 times), followed by addition of alkene (0.1 mmol, 1.0 equiv.), $TMSN_3$ (0.40 mmol, 4.0 equiv.), 4-F-thiolphenol (10 mol%, 0.1 equiv.) in $HCCl_3/H_2O$ (19:1, 0.1 M in regard to alkenes) via syringe under N2. The reaction mixture was placed under 427nm Kessil® light (25%) with proper sealing (see General Procedure A) and allowed to react at room temperature for 36 h. Following this, the reaction mixture was filtered through a pad of celite and rinsed with DCM. The concentrated filtrate was then purified through column chromatography (with eluent of Hex: EA = 10:1) to give the corresponding hydroazidation products as colorless oil.
Yield 61%, 12.2 mg, **r.r.** = 13:1 (determined by ^{1}H NMR).
^{1}H NMR (600 MHz, $CDCl_3$) δ 3.63 (t, *J* = 6.6 Hz, 2H), 3.25 (t, *J* = 7.0 Hz, 2H), 1.62 – 1.51 (m, 4H), 1.38 – 1.28 (m, 12H).
^{13}C NMR (151 MHz, $CDCl_3$) δ 136.24, 129.36, 128.96, 126.09, 50.98, 33.28, 27.89, 26.29.

HRMS ESI: $[M+H]^+$ calcd. for C10H22N3O: 200.1757; Found 200.1755

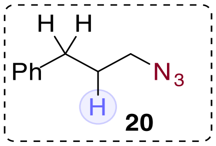

Prepared according to General Procedure A (substrate 0.1 mmol scale): $Fe(NO_3)_3 \cdot 9H_2O$ (10 mol%, 0.1 equiv.) and terpyridine (10 mol%, 0.1 equiv.) were added in an oven-dried 8-mL test vial containing a Teflon®-coated magnetic stir bar. The vial was evacuated and backfilled with N2 (repeated for 4 times), followed by addition of alkene (0.1 mmol, 1.0 equiv.), $TMSN_3$ (0.40 mmol, 4.0 equiv.), 4-F-thiolphenol (10 mol%, 0.1 equiv.) in $HCCl_3/H_2O$ (19:1, 0.1 M in regard to alkenes) via syringe under N2. The reaction mixture was placed under 427nm Kessil® light (25%) with proper sealing (see General Procedure A) and allowed to react at room temperature for 60 h. Following this, the reaction mixture was filtered through a pad of celite and rinsed with DCM. The concentrated filtrate was then purified through preparatory thin-layer chromatography (with eluent of Hex: EA = 20:1) to give the corresponding hydroazidation products as colorless oil.

Yield 37%, 6.0 mg, **r.r.** = 10:1 (determined by ^{1}H NMR).

^{1}H NMR (600 MHz, $CDCl_3$) δ 7.30 (t, J = 7.6 Hz, 2H), 7.24 – 7.16 (m, 3H), 3.29 (t, J = 6.8 Hz, 2H), 2.71 (t, J = 7.6 Hz, 2H), 1.97 – 1.87 (m, 2H).

^{13}C NMR (151 MHz, $CDCl_3$) δ 140.84, 128.51, 128.45, 126.14, 50.64, 32.76, 30.42.

HRMS APCI: $[M-N2+H]^+$ calcd. for C9H12N: 134.0964; Found 134.0964

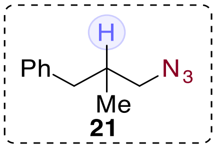

Prepared according to General Procedure A (substrate 0.1 mmol scale): $Fe(NO_3)_3 \cdot 9H_2O$ (10 mol%, 0.1 equiv.) and terpyridine (10 mol%, 0.1 equiv.) were added in an oven-dried 8-mL test vial containing a Teflon®-coated magnetic stir bar. The vial was evacuated and backfilled with N2 (repeated for 4 times), followed by addition of alkene (0.1 mmol, 1.0 equiv.), $TMSN_3$ (0.40 mmol, 4.0 equiv.), 4-F-thiolphenol (10 mol%, 0.1 equiv.) in $HCCl_3/H_2O$ (19:1, 0.1 M in regard to alkenes) via syringe under N2. The reaction mixture was placed under 427nm Kessil® light (25%) with proper sealing (see General Procedure A) and allowed to react at room temperature for 36 h. Following this, the reaction mixture was filtered through a pad of celite and rinsed with DCM. The concentrated filtrate was then purified through preparatory thin-layer chromatography (with eluent of Hex: EA = 20:1) to give the corresponding hydroazidation products as colorless oil.

Yield 56%, 9.8 mg, **r.r.** > 20:1 (determined by ^{1}H NMR).

^{1}H NMR (600 MHz, $CDCl_3$) δ 7.33 – 7.27 (m, 2H), 7.24 – 7.19 (m, 1H), 7.18 – 7.14 (m, 2H), 3.23 (dd, J = 12.0, 5.8 Hz, 1H), 3.15 (dd, J = 12.0, 6.5 Hz, 1H), 2.71 (dd, J = 13.6, 6.7 Hz, 1H), 2.48 (dd, J = 13.5, 7.7 Hz, 1H), 2.09-1.95 (m, 1H), 0.97 (d, J = 6.7 Hz, 3H).

^{13}C NMR (151 MHz, $CDCl_3$) δ 139.80, 129.14, 128.37, 126.13, 56.91, 40.40, 35.53, 17.57.

HRMS APCI: $[M-N2+H]^+$ calcd. for C10H14N: 148.1121; Found 148.1119

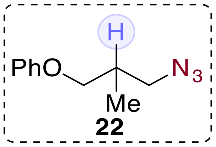

Prepared according to General Procedure A (substrate 0.1 mmol scale): $Fe(NO_3)_3 \cdot 9H_2O$ (10 mol%, 0.1 equiv.) and terpyridine (10 mol%, 0.1 equiv.) were added in an oven-dried 8-mL test vial containing a Teflon®-coated magnetic stir bar. The vial was evacuated and backfilled with N2 (repeated for 4 times), followed by addition of alkene (0.1 mmol, 1.0 equiv.), $TMSN_3$ (0.40 mmol, 4.0 equiv.), 4-F-thiolphenol (10 mol%, 0.1 equiv.) in $HCCl_3/H_2O$ (19:1, 0.1 M in regard to alkenes) via syringe under N2. The reaction mixture was placed under 427nm Kessil® light (25%) with proper sealing (see General Procedure A) and allowed to react at room temperature for 60 h. Following this, the reaction mixture was filtered through a pad of celite and rinsed with DCM. The concentrated filtrate was then purified through preparatory thin-layer chromatography (with eluent of Hex: EA = 20:1) to give the corresponding hydroazidation products as colorless oil.

Yield 57%, 10.9 mg, **r.r.** > 20:1 (determined by ^{1}H NMR).
^{1}H NMR (600 MHz, CDCl$_3$) ^{1}H NMR (600 MHz, Chloroform-*d*) δ 7.31 – 7.26 (m, 2H), 6.98-6.93 (m, 1H), 6.92 – 6.89 (m, 2H), 3.91 – 3.83 (m, 2H), 3.48 (dd, *J* = 12.1, 6.0 Hz, 1H), 3.39 (dd, *J* = 12.1, 6.2 Hz, 1H), 2.27 – 2.18 (m, 1H), 1.10 (d, *J* = 6.9 Hz, 3H).
^{13}C NMR (151 MHz, CDCl$_3$) δ 158.78, 129.48, 120.86, 114.50, 69.59, 54.37, 33.85, 14.79.
HRMS APCI: [M-N2+H]$^+$ calcd. for C10H14NO: 164.1070; Found 164.1069

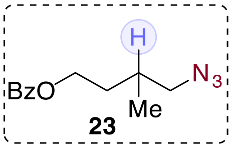

Prepared according to General Procedure A (substrate 0.1 mmol scale): $Fe(NO_3)_3·9H_2O$ (10 mol%, 0.1 equiv.) and terpyridine (10 mol%, 0.1 equiv.) were added in an oven-dried 8-mL test vial containing a Teflon®-coated magnetic stir bar. The vial was evacuated and backfilled with N2 (repeated for 4 times), followed by addition of alkene (0.1 mmol, 1.0 equiv.), $TMSN_3$ (0.40 mmol, 4.0 equiv.), 4-F-thiolphenol (10 mol%, 0.1 equiv.) in $HCCl_3/H_2O$ (19:1, 0.1 M in regard to alkenes) via syringe under N2. The reaction mixture was placed under 427nm Kessil® light (25%) with proper sealing (see General Procedure A) and allowed to react at room temperature for 60 h. Following this, the reaction mixture was filtered through a pad of celite and rinsed with DCM. The concentrated filtrate was then purified through preparatory thin-layer chromatography (with eluent of Hex: EA = 10:1) to give the corresponding hydroazidation products as colorless oil.
Yield 68%, 15.9 mg, **r.r.** > 20:1 (determined by ^{1}H NMR).
^{1}H NMR (600 MHz, CDCl$_3$) δ 8.06 – 8.00 (m, 2H), 7.59 – 7.53 (m, 1H), 7.48 – 7.41 (m, 2H), 4.45 – 4.31 (m, 2H), 3.27 (m, *J* = 12.1, 6.1 Hz, 2H), 2.02 – 1.88 (m, 2H), 1.68 – 1.61 (m, 1H), 1.06 (d, *J* = 6.6 Hz, 3H).
^{13}C NMR (151 MHz, CDCl$_3$) δ 166.69, 133.11, 130.34, 129.68, 128.52, 62.87, 57.64, 33.00, 30.97, 17.67.
HRMS ESI: [M+H]$^+$ calcd. for C12H16N3O2: 234.1237; Found 234.1233

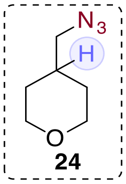

Prepared according to General Procedure A (substrate 0.1 mmol scale): $Fe(NO_3)_3·9H_2O$ (10 mol%, 0.1 equiv.) and terpyridine (10 mol%, 0.1 equiv.) were added in an oven-dried 8-mL test vial containing a Teflon®-coated magnetic stir bar. The vial was evacuated and backfilled with N2 (repeated for 4 times), followed by addition of alkene (0.1 mmol, 1.0 equiv.), $TMSN_3$ (0.40 mmol, 4.0 equiv.), 4-F-thiolphenol (10 mol%, 0.1 equiv.) in $HCCl_3/H_2O$ (19:1, 0.1 M in regard to alkenes) via syringe under N2. The reaction mixture was placed under 427nm Kessil® light (50%) with proper sealing (see General Procedure A) and allowed to react at room temperature for 60 h. Following this, the reaction mixture was filtered through a pad of celite and rinsed with DCM. The concentrated filtrate was then purified through column chromatography (with eluent of Hex: EA = 20:1) to give the corresponding hydroazidation products as colorless oil.
Yield 78% (^{1}H NMR yield due to volatile nature), 11.0 mg, **r.r.** > 20:1 (determined by ^{1}H NMR).
^{1}H NMR (600 MHz, CDCl$_3$) δ 4.04 – 3.95 (m, 2H), 3.42-3.35 (m, 2H), 3.19 (d, *J* = 6.8 Hz, 2H), 1.85-1.76 (m, 1H), 1.69-1.62 (m, 2H), 1.39-1.30 (m, 2H).
^{13}C NMR (151 MHz, CDCl$_3$) δ 67.50, 57.25, 35.46, 30.45.

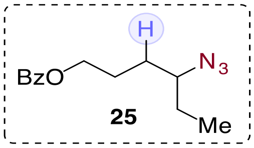

Prepared according to General Procedure A (substrate 0.1 mmol scale): $Fe(NO_3)_3·9H_2O$ (10 mol%, 0.1 equiv.) and terpyridine (10 mol%, 0.1 equiv.) were added in an oven-dried 8-mL test vial containing a Teflon®-coated magnetic stir bar. The vial was evacuated and backfilled with N2 (repeated for 4 times), followed by addition of alkene (0.1 mmol, 1.0 equiv.), $TMSN_3$ (0.40 mmol, 4.0 equiv.), 4-F-thiolphenol (10 mol%, 0.1 equiv.) in $HCCl_3/H_2O$ (19:1, 0.1 M in regard to alkenes) via syringe under N2. The reaction mixture was placed under 427nm Kessil® light (25%) with proper sealing (see General Procedure A) and allowed to react at room

temperature for 36 h. Following this, the reaction mixture was filtered through a pad of celite and rinsed with DCM. The concentrated filtrate was then purified through preparatory thin-layer chromatography (with eluent of Hex: EA = 10:1) to give the corresponding hydroazidation products as colorless oil.
Yield 54%, 13.4 mg, **r.r.** = 1.2:1 (determined by ^{1}H NMR).
^{1}H NMR (600 MHz, CDCl$_3$) δ ^{1}H NMR (600 MHz, Chloroform-*d*) δ 8.10 – 7.99 (m, 2H), 7.60 – 7.53 (m, 1H), 7.50 – 7.39 (m, 2H), 4.50 – 4.31 (m, 2H), 3.61 – 3.46 (m, 0.45H), 3.31-3.24 (m, 0.55H), 2.05 – 1.80 (m, 2H), 1.73 – 1.40 (m, 4H), 1.01 (t, *J* = 7.4 Hz, 1.70H), 0.96 (t, *J* = 7.3 Hz, 1.30H).
^{13}C NMR (151 MHz, CDCl$_3$) δ 166.58, 166.45, 133.05, 132.96, 130.25, 130.10, 129.57, 129.56, 128.42, 128.39, 64.51, 64.06, 61.82, 59.74, 36.66, 33.46, 30.53, 27.42, 25.49, 19.26, 13.84, 10.50.
HRMS ESI: $[M+H]^+$ calcd. for C13H18N3O3: 248.1394; Found 248.1389

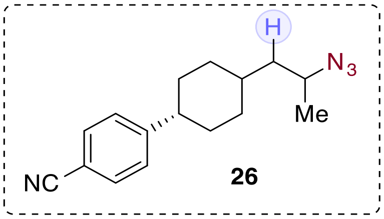

Prepared according to General Procedure A (substrate 0.1 mmol scale): $Fe(NO_3)_3{\cdot}9H_2O$ (10 mol%, 0.1 equiv.), terpyridine (10 mol%, 0.1 equiv.) and alkene (0.1 mmol, 1.0 equiv.) were added in an oven-dried 8-mL test vial containing a Teflon®-coated magnetic stir bar. The vial was evacuated and backfilled with N2 (repeated for 4 times), followed by addition of $TMSN_3$ (0.40 mmol, 4.0 equiv.), 4-F-thiolphenol (10 mol%, 0.1 equiv.) in $HCCl_3/H_2O$ (19:1, 0.1 M in regard to alkenes) via syringe under N2. The reaction mixture was placed under 427nm Kessil® light (50%) with proper sealing (see General Procedure A) and allowed to react at room temperature for 60 h. Following this, the reaction mixture was filtered through a pad of celite and rinsed with DCM. The concentrated filtrate was then purified through preparatory thin-layer chromatography (with eluent of Hex: EA = 10:1) to give the corresponding hydroazidation products as colorless oil.
Yield 55%, 14.8 mg, **r.r.** = 1.3:1 (determined by ^{1}H NMR).
^{1}H NMR (600 MHz, CDCl$_3$) δ 7.57 (d, *J* = 8.1, 2H), 7.29 (d, *J* = 7.9 Hz, 2H), 3.58 – 3.51 (m, 0.57H), 3.11-3.06 (m, 0.43H), 2.58-2.49 (m, 1H), 2.00 – 1.80 (m, 4H), 1.58 – 1.41 (m, 4H), 1.38 – 1.20 (m, 3.47H), 1.17 – 1.00 (m, 2.53H).
^{13}C NMR (151 MHz, CDCl$_3$) δ 152.89, 152.61, 132.27, 132.25, 127.67, 127.65, 119.16, 119.12, 109.84, 109.75, 69.92, 55.38, 44.58, 44.38, 43.62, 41.22, 34.06, 33.66, 33.52, 33.48, 33.45, 33.33, 32.68, 29.87, 28.24, 24.70, 19.96, 10.92.
HRMS ESI: $[M+H]^+$ calcd. for C16H21N4: 269.1761; Found 269.1755

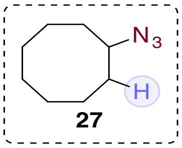

Prepared according to General Procedure A (substrate 0.1 mmol scale): $Fe(NO_3)_3{\cdot}9H_2O$ (10 mol%, 0.1 equiv.) and terpyridine (10 mol%, 0.1 equiv.) were added in an oven-dried 8-mL test vial containing a Teflon®-coated magnetic stir bar. The vial was evacuated and backfilled with N2 (repeated for 4 times), followed by addition of alkene (0.1 mmol, 1.0 equiv.), $TMSN_3$ (0.40 mmol, 4.0 equiv.), 4-F-thiolphenol (10 mol%, 0.1 equiv.) in $HCCl_3/H_2O$ (19:1, 0.1 M in regard to alkenes) via syringe under N2. The reaction mixture was placed under 427nm Kessil® light (25%) with proper sealing (see General Procedure A) and allowed to react at room temperature for 36 h. Following this, the reaction mixture was filtered through a pad of celite and rinsed with DCM. The concentrated filtrate was then purified through column chromatography (with eluent of Hexane) to give the corresponding hydroazidation products as colorless oil.
Yield 64%, 9.8 mg.
^{1}H NMR (600 MHz, CDCl$_3$) δ ^{1}H NMR (600 MHz, Chloroform-*d*) δ 3.56 (tt, *J* = 8.4, 3.9 Hz, 1H), 1.91-1.83 (m, 2H), 1.76 – 1.69 (m, 4H), 1.60-1.48 (m, 8H).
^{13}C NMR (151 MHz, CDCl$_3$) δ 62.25, 30.81, 27.22, 25.14, 23.17.
HRMS APCI: $[M-N2+H]^+$ calcd. for C8H12N: 126.1277; Found 126.1278

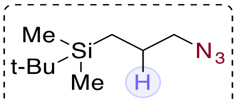

28 Prepared according to General Procedure A (substrate 0.1 mmol scale): $Fe(NO_3)_3 \cdot 9H_2O$ (10 mol%, 0.1 equiv.) and terpyridine (10 mol%, 0.1 equiv.) were added in an oven-dried 8-mL test vial containing a Teflon®-coated magnetic stir bar. The vial was evacuated and backfilled with N2 (repeated for 4 times), followed by addition of alkene (0.1 mmol, 1.0 equiv.), $TMSN_3$ (0.40 mmol, 4.0 equiv.), 4-F-thiolphenol (10 mol%, 0.1 equiv.) in $HCCl_3/H_2O$ (19:1, 0.1 M in regard to alkenes) via syringe under N2. The reaction mixture was placed under 427nm Kessil® light (25%) with proper sealing (see General Procedure A) and allowed to react at room temperature for 36 h. Following this, the reaction mixture was filtered through a pad of celite and rinsed with DCM. The concentrated filtrate was then purified through column chromatography (with eluent of Hexane) to give the corresponding hydroazidation products as colorless oil.

Yield 50%, 10.0 mg, **r.r.** > 20:1 (determined by ^{1}H NMR).

^{1}H NMR (600 MHz, $CDCl_3$) δ 3.24 (t, J = 7.1 Hz, 2H), 1.62-1.55 (m, 2H), 0.87 (s, 9H), 0.58 – 0.50 (m, 2H), -0.05 (s, 6H).

^{13}C NMR (151 MHz, $CDCl_3$) δ 54.71, 26.53, 24.08, 16.51, 9.65, -6.37.

HRMS APCI: $[M\text{-}N2\text{+}H]^+$ calcd. for C9H22NO: 172.1516; Found 172.1513

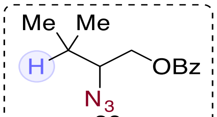

29 Prepared according to General Procedure A (substrate 0.1 mmol scale): $Fe(NO_3)_3 \cdot 9H_2O$ (10 mol%, 0.1 equiv.) and terpyridine (10 mol%, 0.1 equiv.) were added in an oven-dried 8-mL test vial containing a Teflon®-coated magnetic stir bar. The vial was evacuated and backfilled with N2 (repeated for 4 times), followed by addition of alkene (0.1 mmol, 1.0 equiv.), $TMSN_3$ (0.40 mmol, 4.0 equiv.), 4-F-thiolphenol (10 mol%, 0.1 equiv.) in $HCCl_3/H_2O$ (19:1, 0.1 M in regard to alkenes) via syringe under N2. The reaction mixture was placed under 427nm Kessil® light (25%) with proper sealing (see General Procedure A) and allowed to react at room temperature for 36 h. Following this, the reaction mixture was filtered through a pad of celite and rinsed with DCM. The concentrated filtrate was then purified through preparatory thin-layer chromatography (with eluent of Hex: EA = 10:1) to give the corresponding hydroazidation products as colorless oil.

Yield 51%, 11.9 mg, **r.r.** > 20:1 (determined by ^{1}H NMR).

^{1}H NMR (600 MHz, $CDCl_3$) δ 8.11 – 8.03 (m, 2H), 7.61 – 7.53 (m, 1H), 7.50 – 7.41 (m, 2H), 4.56 (dd, J = 11.5, 3.2 Hz, 1H), 4.32 (dd, J = 11.5, 8.5 Hz, 1H), 3.58-3.51 (m, 1H), 1.98 – 1.88 (m, 1H), 1.09-0.99 (m, 6H).

^{13}C NMR (151 MHz, $CDCl_3$) δ 166.34, 133.27, 129.79, 128.50, 67.07, 66.17, 29.95, 19.53, 18.29.

HRMS ESI: $[M+H]^+$ calcd. for C12H16N3O2: 234.1237; Found 234.1235

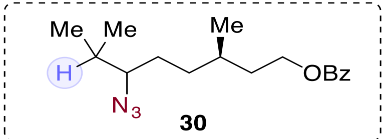

30 Prepared according to General Procedure A (substrate 0.1 mmol scale): $Fe(NO_3)_3 \cdot 9H_2O$ (10 mol%, 0.1 equiv.) and terpyridine (10 mol%, 0.1 equiv.) were added in an oven-dried 8-mL test vial containing a Teflon®-coated magnetic stir bar. The vial was evacuated and backfilled with N2 (repeated for 4 times), followed by addition of alkene (0.1 mmol, 1.0 equiv.), $TMSN_3$ (0.40 mmol, 4.0 equiv.), 4-F-thiolphenol (10 mol%, 0.1 equiv.) in $HCCl_3/H_2O$ (19:1, 0.1 M in regard to alkenes) via syringe under N2. The reaction mixture was placed under 427nm Kessil® light (25%) with proper sealing (see General Procedure A) and allowed to react at room temperature for 60 h. Following this, the reaction mixture was filtered through a pad of celite and rinsed with DCM. The concentrated filtrate was then purified through preparatory thin-layer chromatography (with eluent of Hex: EA = 10:1) to give the corresponding hydroazidation products as colorless oil.

Yield 74%, 22.5 mg, **r.r.** > 20:1, d.r. = 1:1 (determined by ^{1}H NMR).

^{1}H NMR (600 MHz, $CDCl_3$) δ 8.07-8.01 (m, 2H), 7.57 – 7.53 (m, 1H), 7.47-7.40 (m, 2H), 4.41 – 4.32 (m, 2H), 3.19-2.90 (m, 1H), 1.89 – 1.76 (m, 2H), 1.70 – 1.38 (m, 6H), 1.01 – 0.90 (m, 9H).

^{13}C NMR (151 MHz, $CDCl_3$) δ 166.65, 132.86, 130.43, 129.54, 128.35, 69.62, 69.53, 63.32, 63.31, 35.68, 35.39, 33.78, 33.72, 32.60, 32.45, 30.07, 29.99, 29.00, 28.98, 19.58, 19.56, 19.49, 19.38, 18.01, 17.82.

HRMS ESI: $[M+H]^+$ calcd. for C17H26N3O2: 304.2020; Found 304.2013

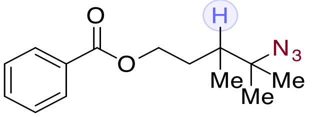

31

Prepared according to General Procedure A (substrate 0.1 mmol scale): $Fe(NO_3)_3{\cdot}9H_2O$ (10 mol%, 0.1 equiv.) and terpyridine (10 mol%, 0.1 equiv.) were added in an oven-dried 8-mL test vial containing a Teflon®-coated magnetic stir bar. The vial was evacuated and backfilled with N2 (repeated for 4 times), followed by addition of alkene (0.1 mmol, 1.0 equiv.), $TMSN_3$ (0.40 mmol, 4.0 equiv.), 4-F-thiolphenol (10 mol%, 0.1 equiv.) in $HCCl_3/H_2O$ (19:1, 0.1 M in regard to alkenes) via syringe under N2. The reaction mixture was placed under 427nm Kessil® light (25%) with proper sealing (see General Procedure A) and allowed to react at room temperature for 60 h. Following this, the reaction mixture was filtered through a pad of celite and rinsed with DCM. The concentrated filtrate was then purified through preparatory thin-layer chromatography (with eluent of Hex: EA = 10:1) to give the corresponding hydroazidation products as colorless oil.
Yield 39%, 10.2 mg.
^{1}H NMR (600 MHz, $CDCl_3$) δ 8.09 – 7.99 (m, 2H), 7.59 – 7.52 (m, 1H), 7.48 – 7.40 (m, 2H), 4.46 – 4.39 (m, 1H), 4.36 – 4.30 (m, 1H), 2.17 – 2.08 (m, 1H), 1.75 – 1.64 (m, 1H), 1.49 – 1.41 (m, 1H), 1.30 (s, 3H), 1.25 (s, 3H), 1.01 (d, *J* = 7.0 Hz, 3H).
^{13}C NMR (151 MHz, $CDCl_3$) δ 166.64, 132.94, 130.29, 129.57, 128.39, 64.60, 63.61, 39.28, 30.74, 23.90, 22.61, 14.52.
HRMS ESI: $[M+Na]^+$ calcd. for C14H19N3O2Na: 284.1369; Found 284.1370

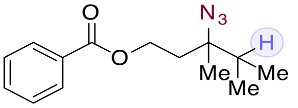

31'

Prepared according to General Procedure A (substrate 0.1 mmol scale): $Fe(NO_3)_3{\cdot}9H_2O$ (10 mol%, 0.1 equiv.) and terpyridine (10 mol%, 0.1 equiv.) were added in an oven-dried 8-mL test vial containing a Teflon®-coated magnetic stir bar. The vial was evacuated and backfilled with N2 (repeated for 4 times), followed by addition of alkene (0.1 mmol, 1.0 equiv.), $TMSN_3$ (0.40 mmol, 4.0 equiv.), 4-F-thiolphenol (10 mol%, 0.1 equiv.) in $HCCl_3/H_2O$ (19:1, 0.1 M in regard to alkenes) via syringe under N2. The reaction mixture was placed under 427nm Kessil® light (25%) with proper sealing (see General Procedure A) and allowed to react at room temperature for 60 h. Following this, the reaction mixture was filtered through a pad of celite and rinsed with DCM. The concentrated filtrate was then purified through preparatory thin-layer chromatography (with eluent of Hex: EA = 10:1) to give the corresponding hydroazidation products as colorless oil.
Yield 19%, 4.9 mg.
^{1}H NMR (600 MHz, $CDCl_3$) δ 8.08 – 8.00 (m, 2H), 7.59 – 7.52 (m, 1H), 7.48 – 7.41 (m, 2H), 4.46 (td, *J* = 7.0, 1.4 Hz, 2H), 2.00 (t, *J* = 7.1 Hz, 2H), 1.92 – 1.81 (m, 1H), 1.31 (d, *J* = 1.5 Hz, 3H), 1.03 – 0.94 (m, 6H).
^{13}C NMR (151 MHz, $CDCl_3$) δ 166.57, 133.00, 130.18, 129.58, 128.40, 65.87, 61.25, 36.34, 35.66, 19.73, 17.48, 17.41.
HRMS ESI: $[M+Na]^+$ calcd. for C14H19N3O2Na: 284.1369; Found 284.1376

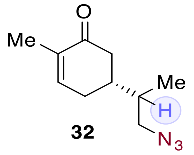

32

Prepared according to General Procedure A (substrate 0.1 mmol scale): $Fe(NO_3)_3{\cdot}9H_2O$ (10 mol%, 0.1 equiv.) and terpyridine (10 mol%, 0.1 equiv.) were added in an oven-dried 8-mL test vial containing a Teflon®-coated magnetic stir bar. The vial was evacuated and backfilled with N2 (repeated for 4 times), followed by addition of alkene (0.1 mmol, 1.0 equiv.), $TMSN_3$ (0.40 mmol, 4.0 equiv.), 4-F-thiolphenol (10 mol%, 0.1 equiv.) in $HCCl_3/H_2O$ (19:1, 0.1 M in regard to alkenes) via syringe under N2. The reaction mixture was placed

under 427nm Kessil® light (25%) with proper sealing (see General Procedure A) and allowed to react at room temperature for 36 h. Following this, the reaction mixture was filtered through a pad of celite and rinsed with DCM. The concentrated filtrate was then purified through preparatory thin-layer chromatography (with eluent of Hex: EA = 5:1) to give the corresponding hydroazidation products as colorless oil.
Yield 31%, 6.0 mg, **r.r.** > 20:1, d.r. = 1:1 (determined by ^{1}H NMR).
^{1}H NMR (600 MHz, CDCl$_3$) δ 6.74 (dt, *J* = 6.2, 1.8 Hz, 1H), 3.36-3.29 (m, 1H), 3.27-3.21 (m, 1H), 2.54 – 2.45 (m, 1H), 2.38 – 2.28 (m, 1H), 2.23 – 2.08 (m, 3H), 1.77 (s, 3H), 1.72 (p, *J* = 6.3 Hz, 1H), 0.99 (dd, *J* = 6.9, 2.6 Hz, 3H).
^{13}C NMR (151 MHz, CDCl$_3$) δ 199.60, 199.53, 144.58, 144.54, 135.61, 55.20, 55.11, 42.34, 40.77, 37.75, 37.69, 37.27, 37.19, 30.34, 28.60, 15.67, 15.65, 14.56, 14.55.
HRMS ESI: [M+H]$^{+}$ calcd. for C10H16N3O: 194.1288; Found 194.1286

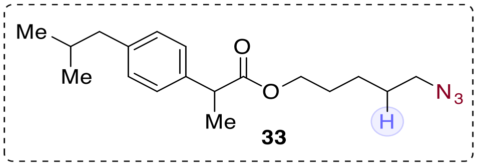

Prepared according to General Procedure A (substrate 0.1 mmol scale): Fe(NO$_3$)$_3$·9H$_2$O (10 mol%, 0.1 equiv.) and terpyridine (10 mol%, 0.1 equiv.) were added in an oven-dried 8-mL test vial containing a Teflon®-coated magnetic stir bar. The vial was evacuated and backfilled with N2 (repeated for 4 times), followed by addition of alkene (0.1 mmol, 1.0 equiv.), TMSN$_3$ (0.40 mmol, 4.0 equiv.), 4-F-thiolphenol (10 mol%, 0.1 equiv.) in HCCl$_3$/H$_2$O (19:1, 0.1 M in regard to alkenes) via syringe under N2. The reaction mixture was placed under 427nm Kessil® light (25%) with proper sealing (see General Procedure A) and allowed to react at room temperature for 60 h. Following this, the reaction mixture was filtered through a pad of celite and rinsed with DCM. The concentrated filtrate was then purified through preparatory thin-layer chromatography (with eluent of Hex: EA = 10:1) to give the corresponding hydroazidation products as colorless oil.
Yield 69%, 21.9 mg, **r.r.** = 13:1 (determined by ^{1}H NMR).
^{1}H NMR (600 MHz, CDCl$_3$) δ 7.20 (d, *J* = 7.2 Hz, 2H), 7.09 (d, *J* = 7.0 Hz, 2H), 4.14-4.00 (m, 2H), 3.74 – 3.64 (m, 1H), 3.20 (td, *J* = 6.9, 1.5 Hz, 2H), 2.45 (dd, *J* = 7.2, 1.5 Hz, 2H), 1.90 – 1.77 (m, 1H), 1.64 – 1.57 (m, 2H), 1.57 – 1.51 (m, 2H), 1.49 (dd, *J* = 7.4, 1.5 Hz, 3H), 1.36 – 1.29 (m, 2H), 0.90 (dd, *J* = 6.6, 1.5 Hz, 6H).
^{13}C NMR (151 MHz, CDCl$_3$) δ 174.76, 140.51, 137.82, 129.29, 127.14, 64.25, 51.21, 45.17, 45.02, 30.19, 28.37, 28.06, 23.05, 22.37, 18.39.
HRMS ESI: [M+H]$^{+}$ calcd. for C18H28N3O2: 318.2176; Found 318.2170

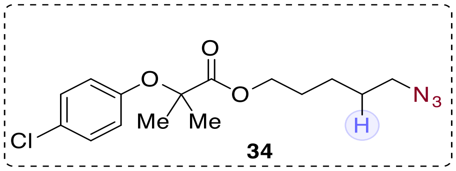

Prepared according to General Procedure A (substrate 0.1 mmol scale): Fe(NO$_3$)$_3$·9H$_2$O (10 mol%, 0.1 equiv.) and terpyridine (10 mol%, 0.1 equiv.) were added in an oven-dried 8-mL test vial containing a Teflon®-coated magnetic stir bar. The vial was evacuated and backfilled with N2 (repeated for 4 times), followed by addition of alkene (0.1 mmol, 1.0 equiv.), TMSN$_3$ (0.40 mmol, 4.0 equiv.), 4-F-thiolphenol (10 mol%, 0.1 equiv.) in HCCl$_3$/H$_2$O (19:1, 0.1 M in regard to alkenes) via syringe under N2. The reaction mixture was placed under 427nm Kessil® light (25%) with proper sealing (see General Procedure A) and allowed to react at room temperature for 60 h. Following this, the reaction mixture was filtered through a pad of celite and rinsed with DCM. The concentrated filtrate was then purified through preparatory thin-layer chromatography (with eluent of Hex: EA = 10:1) to give the corresponding hydroazidation products as colorless oil.
Yield 76%, 24.8 mg, **r.r.** = 13:1 (determined by ^{1}H NMR).
^{1}H NMR (600 MHz, CDCl$_3$) δ 7.22 – 7.15 (m, 2H), 6.81 – 6.72 (m, 2H), 4.15 (t, *J* = 6.5 Hz, 2H), 3.21 (t, *J* = 6.8 Hz, 2H), 1.66 – 1.61 (m, 2H), 1.58 (s, 6H), 1.54 (p, *J* = 7.0 Hz, 2H), 1.33 – 1.27 (m, 2H).
^{13}C NMR (151 MHz, CDCl$_3$) δ 174.01, 154.14, 129.12, 127.08, 120.16, 79.46, 65.19, 51.16, 28.40, 28.01, 25.34, 23.08.
HRMS ESI: [M+H]$^{+}$ calcd. for C15H21ClN3O3: 326.1266; Found 326.1262

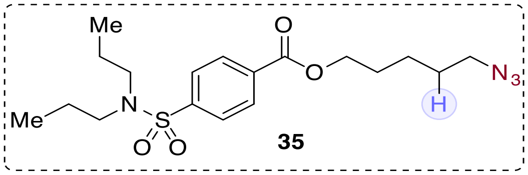
Prepared according to General Procedure A (substrate 0.1 mmol scale): $Fe(NO_3)_3 \cdot 9H_2O$ (10 mol%, 0.1 equiv.) and terpyridine (10 mol%, 0.1 equiv.) were added in an oven-dried 8-mL test vial containing a Teflon®-coated magnetic stir bar. The vial was evacuated and backfilled with N2 (repeated for 4 times), followed by addition of alkene (0.1 mmol, 1.0 equiv.), $TMSN_3$ (0.40 mmol, 4.0 equiv.), 4-F-thiolphenol (10 mol%, 0.1 equiv.) in $HCCl_3/H_2O$ (19:1, 0.1 M in regard to alkenes) via syringe under N2. The reaction mixture was placed under 427nm Kessil® light (25%) with proper sealing (see General Procedure A) and allowed to react at room temperature for 60 h. Following this, the reaction mixture was filtered through a pad of celite and rinsed with DCM. The concentrated filtrate was then purified through preparatory thin-layer chromatography (with eluent of Hex: EA = 8:1) to give the corresponding hydroazidation products as colorless oil.
Yield 65%, 25.8 mg, **r.r.** = 12:1 (determined by ^{1}H NMR).
^{1}H NMR (600 MHz, CDCl$_3$) δ 8.14 (d, *J* = 8.2 Hz, 2H), 7.87 (d, *J* = 8.2 Hz, 2H), 4.36 (t, *J* = 6.5 Hz, 2H), 3.31 (t, *J* = 6.8 Hz, 2H), 3.13-3.05 (m, 4H), 1.82 (p, *J* = 6.8 Hz, 2H), 1.68 (p, *J* = 7.0 Hz, 2H), 1.59 – 1.49 (m, 6H), 0.86 (t, *J* = 7.4 Hz, 6H).
^{13}C NMR (151 MHz, CDCl$_3$) δ 165.26, 144.26, 133.60, 130.18, 127.02, 65.31, 51.23, 49.93, 28.54, 28.24, 23.31, 21.94, 11.16.
HRMS ESI: $[M+H]^+$ calcd. for C18H29N4O4S: 397.1904; Found 397.1896

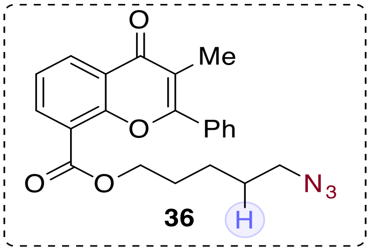
Prepared according to General Procedure A (substrate 0.1 mmol scale): $Fe(NO_3)_3 \cdot 9H_2O$ (10 mol%, 0.1 equiv.), terpyridine (10 mol%, 0.1 equiv.) and alkene (0.1 mmol, 1.0 equiv.) were added in an oven-dried 8-mL test vial containing a Teflon®-coated magnetic stir bar. The vial was evacuated and backfilled with N2 (repeated for 4 times), followed by addition of $TMSN_3$ (0.40 mmol, 4.0 equiv.), 4-F-thiolphenol (10 mol%, 0.1 equiv.) in $HCCl_3/H_2O$ (19:1, 0.1 M in regard to alkenes) via syringe under N2. The reaction mixture was placed under 427nm Kessil® light (50%) with proper sealing (see General Procedure A) and allowed to react at room temperature for 60 h. Following this, the reaction mixture was filtered through a pad of celite and rinsed with DCM. The concentrated filtrate was then purified through preparatory thin-layer chromatography (with eluent of Hex: EA = 5:1) to give the corresponding hydroazidation products as colorless oil.
Yield 74%, 29.0 mg, **r.r.** = 13:1 (determined by ^{1}H NMR).
^{1}H NMR (600 MHz, CDCl$_3$) δ 8.46 (dt, *J* = 7.9, 1.2 Hz, 1H), 8.26 (dt, *J* = 7.5, 1.2 Hz, 1H), 7.80 – 7.74 (m, 2H), 7.57-7.50 (m, 3H), 7.45 (t, *J* = 7.6 Hz, 1H), 4.35 (t, *J* = 6.6 Hz, 2H), 3.19 (t, *J* = 6.9 Hz, 2H), 2.23 (s, 3H), 1.77 – 1.71 (m, 2H), 1.58-1.51 (m, 2H), 1.44 – 1.36 (m, 2H).
^{13}C NMR (151 MHz, CDCl$_3$) δ 178.28, 164.58, 161.06, 154.45, 136.16, 133.11, 130.82, 130.50, 129.34, 128.44, 124.04, 123.32, 120.74, 117.73, 65.28, 51.16, 28.46, 28.22, 23.26, 11.77.
HRMS ESI: $[M+H]^+$ calcd. for C22H22N3O4: 392.1605; Found 392.1594

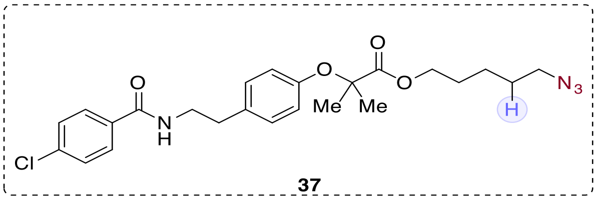
Prepared according to General Procedure A (substrate 0.1 mmol scale): $Fe(NO_3)_3 \cdot 9H_2O$ (10 mol%, 0.1 equiv.), terpyridine (10 mol%, 0.1 equiv.) and alkene (0.1 mmol, 1.0 equiv.) were added in an oven-dried 8-mL test vial containing a Teflon®-coated magnetic stir bar. The vial was

evacuated and backfilled with N2 (repeated for 4 times), followed by addition of $TMSN_3$ (0.40 mmol, 4.0 equiv.), 4-F-thiolphenol (10 mol%, 0.1 equiv.) in $HCCl_3/H_2O$ (19:1, 0.1 M in regard to alkenes) via syringe under N2. The reaction mixture was placed under 427nm Kessil® light (25%) with proper sealing (see General Procedure A) and allowed to react at room temperature for 60 h. Following this, the reaction mixture was filtered through a pad of celite and rinsed with DCM. The concentrated filtrate was then purified through preparatory thin-layer chromatography (with eluent of Hex: EA = 3:1) to give the corresponding hydroazidation products as colorless oil.
Yield 70%, 33.1 mg, **r.r.** = 13:1 (determined by ^{1}H NMR).
^{1}H NMR (600 MHz, $CDCl_3$) δ 7.61 (d, *J* = 8.5 Hz, 1H), 7.35 (d, *J* = 8.5 Hz, 1H), 7.07 (d, *J* = 8.2 Hz, 1H), 6.78 (d, *J* = 8.5 Hz, 1H), 6.24 (t, *J* = 5.7 Hz, 1H), 4.15 (t, *J* = 6.5 Hz, 2H), 3.63 (q, *J* = 6.7 Hz, 2H), 3.19 (t, *J* = 6.8 Hz, 2H), 2.84 (t, *J* = 7.0 Hz, 2H), 1.63 (p, *J* = 6.7 Hz, 2H), 1.58 (s, 6H), 1.54 (p, *J* = 7.0 Hz, 2H), 1.38 – 1.30 (m, 2H).
^{13}C NMR (151 MHz, $CDCl_3$) δ 174.41, 166.52, 154.26, 137.70, 133.11, 132.42, 129.56, 128.88, 128.38, 119.35, 79.21, 65.17, 51.25, 41.37, 34.81, 28.46, 28.09, 25.48, 23.17.
HRMS ESI: $[M+H]^+$ calcd. for C24H30ClN4O4: 473.1950; Found 473.1941

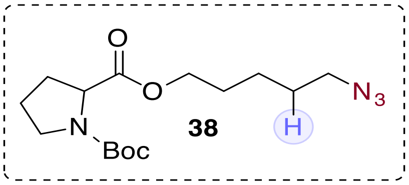

38

Prepared according to General Procedure A (substrate 0.1 mmol scale): $Fe(NO_3)_3{\cdot}9H_2O$ (10 mol%, 0.1 equiv.) and terpyridine (10 mol%, 0.1 equiv.) were added in an oven-dried 8-mL test vial containing a Teflon®-coated magnetic stir bar. The vial was evacuated and backfilled with N2 (repeated for 4 times), followed by addition of alkene (0.1 mmol, 1.0 equiv.), $TMSN_3$ (0.40 mmol, 4.0 equiv.), 4-F-thiolphenol (10 mol%, 0.1 equiv.) in $HCCl_3/H_2O$ (19:1, 0.1 M in regard to alkenes) via syringe under N2. The reaction mixture was placed under 427nm Kessil® light (50%) with proper sealing (see General Procedure A) and allowed to react at room temperature for 48 h. Following this, the reaction mixture was filtered through a pad of celite and rinsed with DCM. The concentrated filtrate was then purified through column chromatography (with eluent of Hex: EA = 5:1) to give the corresponding hydroazidation products as colorless oil.
Yield 55%, 18.0 mg, **r.r.** = 9:1 (determined by ^{13}C NMR).
^{1}H NMR (600 MHz, $CDCl_3$) δ 4.35 – 3.99 (m, 3H), 3.63 – 3.19 (m, 4H), 2.25-2.12 (m, 1H), 2.00 – 1.46 (m, 9H), 1.46-1.37 (m, 9H).
^{13}C NMR (151 MHz, $CDCl_3$) δ 173.28, 173.06, 154.40, 153.82, 79.84, 79.72, 64.58, 64.55, 59.17, 58.87, 51.25, 51.22, 46.56, 46.33, 30.95, 29.99, 28.49, 28.46, 28.44, 28.33, 28.23, 28.18, 24.33, 23.63, 23.20, 23.09.
HRMS ESI: $[M+H]^+$ calcd. for C15H27N4O4: 327.2027; Found 327.2020

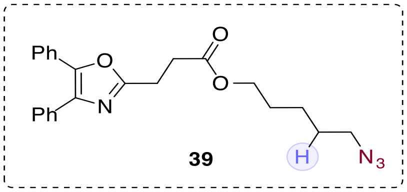

39

Prepared according to General Procedure A (substrate 0.1 mmol scale): $Fe(NO_3)_3{\cdot}9H_2O$ (10 mol%, 0.1 equiv.) and terpyridine (10 mol%, 0.1 equiv.) were added in an oven-dried 8-mL test vial containing a Teflon®-coated magnetic stir bar. The vial was evacuated and backfilled with N2 (repeated for 4 times), followed by addition of alkene (0.1 mmol, 1.0 equiv.), $TMSN_3$ (0.40 mmol, 4.0 equiv.), 4-F-thiolphenol (10 mol%, 0.1 equiv.) in $HCCl_3/H_2O$ (19:1, 0.1 M in regard to alkenes) via syringe under N2. The reaction mixture was placed under 427nm Kessil® light (50%) with proper sealing (see General Procedure A) and allowed to react at room temperature for 72 h. Following this, the reaction mixture was filtered through a pad of celite and rinsed with DCM. The concentrated filtrate was then purified through preparatory thin-layer chromatography (with eluent of Hex: EA = 5:1) to give the corresponding hydroazidation products as colorless oil.
Yield 43%, 17.4 mg, **r.r.** = 11:1 (determined by ^{1}H NMR).
^{1}H NMR (600 MHz, $CDCl_3$) δ 7.65 – 7.61 (m, 2H), 7.59-7.54 (m, 2H), 7.39-7.29 (m, 6H), 4.14 (t, *J* = 6.5 Hz, 2H), 3.30-3.05 (m, 4H), 2.92 (t, *J* = 7.5 Hz, 2H), 1.69 – 1.62 (m, 2H), 1.62-1.55 (m, 2H), 1.45 – 1.38 (m, 2H).
^{13}C NMR (151 MHz, $CDCl_3$) δ 172.04, 161.79, 145.43, 135.11, 132.43, 128.97, 128.67, 128.57, 128.50, 128.11, 127.90, 126.47, 64.51, 51.20, 31.16, 28.48, 28.19, 23.57, 23.18.
HRMS ESI: $[M+H]^+$ calcd. for C23H25N4O3: 405.1921; Found 405.1912

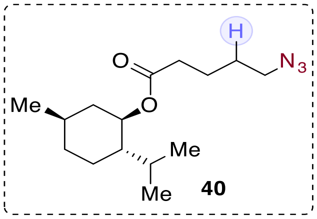

40

Prepared according to General Procedure A (substrate 0.1 mmol scale): $Fe(NO_3)_3{\cdot}9H_2O$ (10 mol%, 0.1 equiv.) and terpyridine (10 mol%, 0.1 equiv.) were added in an oven-dried 8-mL test vial containing a Teflon®-coated magnetic stir bar. The vial was evacuated and backfilled with N2 (repeated for 4 times), followed by addition of alkene (0.1 mmol, 1.0 equiv.), $TMSN_3$ (0.40 mmol, 4.0 equiv.), 4-F-thiolphenol (10 mol%, 0.1 equiv.) in $HCCl_3/H_2O$ (19:1, 0.1 M in regard to alkenes) via syringe under N2. The reaction mixture was placed under 427nm Kessil® light (25%) with proper sealing (see General Procedure A) and allowed to react at room temperature for 60 h. Following this, the reaction mixture was filtered column thin-layer chromatography (with eluent of Hex: EA = 20:1) to give the corresponding hydroazidation products as colorless oil.

Yield 63%, 17.7 mg, **r.r.** = 11:1 (determined by ^{1}H NMR).

^{1}H NMR (600 MHz, $CDCl_3$) δ 4.68 (td, *J* = 10.9, 4.4 Hz, 1H), 3.29 (t, *J* = 6.7 Hz, 2H), 2.32 (t, *J* = 7.3 Hz, 2H), 2.02 – 1.93 (m, 1H), 1.88-1.78 (m, 1H), 1.76 – 1.59 (m, 6H), 1.53-1.43 (m, 1H), 1.40-1.32 (m, 1H), 1.08 – 1.01 (m, 1H), 0.98 – 0.82 (m, 8H), 0.75 (d, *J* = 7.0 Hz, 3H).

^{13}C NMR (151 MHz, $CDCl_3$) δ 172.75, 74.22, 51.09, 47.01, 40.95, 34.25, 34.06, 31.39, 28.31, 26.31, 23.42, 22.27, 22.03, 20.76, 16.30.

HRMS ESI: $[M+H]^+$ calcd. for C15H28N3O2: 282.2176; Found 282.2171

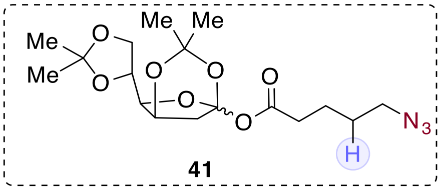

41

Prepared according to General Procedure A (substrate 0.1 mmol scale): $Fe(NO_3)_3{\cdot}9H_2O$ (10 mol%, 0.1 equiv.) and terpyridine (10 mol%, 0.1 equiv.) were added in an oven-dried 8-mL test vial containing a Teflon®-coated magnetic stir bar. The vial was evacuated and backfilled with N2 (repeated for 4 times), followed by addition of alkene (0.1 mmol, 1.0 equiv.), $TMSN_3$ (0.40 mmol, 4.0 equiv.), 4-F-thiolphenol (10 mol%, 0.1 equiv.) in $HCCl_3/H_2O$ (19:1, 0.1 M in regard to alkenes) via syringe under N2. The reaction mixture was placed under 427nm Kessil® light (25%) with proper sealing (see General Procedure A) and allowed to react at room temperature for 60 h. Following this, the reaction mixture was filtered through a pad of celite and rinsed with DCM. The concentrated filtrate was then purified through column chromatography (with eluent of Hex: EA = 10:1) to give the corresponding hydroazidation products as colorless oil.

Yield 37%, 14.3 mg, **r.r.** = 10:1 (determined by ^{1}H NMR).

^{1}H NMR (600 MHz, $CDCl_3$) δ 6.14 (s, 1H), 4.85 (dd, *J* = 5.8, 3.6 Hz, 1H), 4.68 (d, *J* = 5.8 Hz, 1H), 4.46 – 4.33 (m, 1H), 4.09 (dd, *J* = 8.8, 6.4 Hz, 1H), 4.05-3.97 (m, 2H), 3.30 (t, *J* = 6.7 Hz, 2H), 2.40-2.28 (m, 2H), 1.71 (p, *J* = 7.3 Hz, 2H), 1.66-1.60 (m, 2H), 1.48 (s, 3H), 1.45 (s, 3H), 1.37 (s, 3H), 1.34 (s, 3H).

^{13}C NMR (151 MHz, $CDCl_3$) δ 171.54, 113.31, 109.38, 100.77, 85.07, 82.29, 79.32, 72.87, 66.84, 51.01, 33.61, 28.17, 26.99, 25.95, 25.13, 24.67, 21.86.

HRMS ESI: $[M+NH4]^+$ calcd. for C17H31N4O7: 403.2187; Found 403.2179

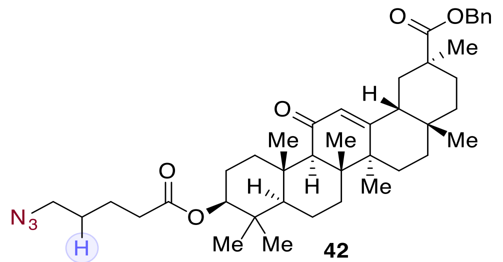

42

Prepared according to General Procedure A (substrate 0.1 mmol scale): $Fe(NO_3)_3 \cdot 9H_2O$ (10 mol%, 0.1 equiv.), terpyridine (10 mol%, 0.1 equiv.) and alkene (0.1 mmol, 1.0 equiv.) were added in an oven-dried 8-mL test vial containing a Teflon®-coated magnetic stir bar. The vial was evacuated and backfilled with N2 (repeated for 4 times), followed by addition of $TMSN_3$ (0.40 mmol, 4.0 equiv.), 4-F-thiolphenol (10 mol%, 0.1 equiv.) in $HCCl_3/H_2O$ (19:1, 0.1 M in regard to alkenes) via syringe under N2. The reaction mixture was placed under 427nm Kessil® light (25%) with proper sealing (see General Procedure A) and allowed to react at room temperature for 96 h. Following this, the reaction mixture was filtered through a pad of celite and rinsed with DCM. The concentrated filtrate was then purified through preparatory thin-layer chromatography (with eluent of Hex: EA = 5:1) to give the corresponding hydroazidation products as white solid, **metling point** 169 °C.
Yield 68%, 46.6 mg, **r.r.** = 10:1 (determined by ^{1}H NMR).
^{1}H NMR (600 MHz, CDCl$_3$) δ 7.39 – 7.31 (m, 5H), 5.54 (s, 1H), 5.20 (d, *J* = 12.2 Hz, 1H), 5.08 (d, *J* = 12.2 Hz, 1H), 4.52 (dd, *J* = 11.8, 4.7 Hz, 1H), 3.29 (t, *J* = 6.7 Hz, 2H), 2.79 (dt, *J* = 13.6, 3.6 Hz, 1H), 2.37 – 2.30 (m, 3H), 2.04 – 1.92 (m, 4H), 1.82 – 1.56 (m, 11H), 1.47 – 1.27 (m, 9H), 1.16-1.14 (m, 6H), 1.10 (s, 3H), 1.07 – 0.96 (m, 2H), 0.90-0.85 (m, 6H), 0.73 (s, 3H).
^{13}C NMR (151 MHz, CDCl$_3$) δ 200.01, 176.22, 172.93, 169.11, 136.14, 128.63, 128.47, 128.31, 128.26, 80.65, 66.23, 61.68, 55.01, 51.09, 48.23, 45.37, 44.00, 43.18, 41.06, 38.78, 38.09, 37.65, 36.93, 34.14, 32.70, 31.79, 31.17, 28.42, 28.37, 28.29, 28.11, 26.46, 26.40, 23.61, 23.31, 22.28, 18.68, 17.38, 16.78, 16.41.
HRMS ESI: $[M+H]^+$ calcd. for C42H60N3O5: 686.4527; Found 686.4514

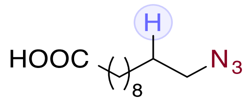

43

Prepared according to General Procedure A (substrate 0.1 mmol scale): $Fe(NO_3)_3 \cdot 9H_2O$ (10 mol%, 0.1 equiv.) and terpyridine (10 mol%, 0.1 equiv.) were added in an oven-dried 8-mL test vial containing a Teflon®-coated magnetic stir bar. The vial was evacuated and backfilled with N2 (repeated for 4 times), followed by addition of alkene (0.1 mmol, 1.0 equiv.), $TMSN_3$ (0.40 mmol, 4.0 equiv.), 4-F-thiolphenol (10 mol%, 0.1 equiv.) in $HCCl_3/H_2O$ (19:1, 0.1 M in regard to alkenes) via syringe under N2. The reaction mixture was placed under 427nm Kessil® light (25%) with proper sealing (see General Procedure A) and allowed to react at room temperature for 60 h. Following this, the reaction mixture was filtered through a pad of celite and rinsed with DCM. The concentrated filtrate was then purified through column chromatography (with eluent of Hex: EA = 10:1) to give the corresponding hydroazidation products as colorless oil.
Yield 62%, 14.1 mg, **r.r.** = 11:1 (determined by ^{1}H NMR).
^{1}H NMR (600 MHz, CDCl$_3$) δ 3.25 (t, *J* = 7.0 Hz, 2H), 2.35 (t, *J* = 7.6 Hz, 2H), 1.66-1.56 (m, 4H), 1.40 – 1.27 (m, 12H).
^{13}C NMR (151 MHz, CDCl$_3$) δ 180.17, 51.49, 34.06, 29.38, 29.29, 29.18, 29.11, 29.02, 28.83, 26.70, 24.65.
HRMS ESI: $[M-H]^-$ calcd. for C11H20N3O2: 226.1550; Found 226.1557

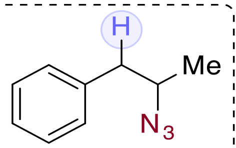
44

Prepared according to General Procedure A (substrate 0.1 mmol scale): $Fe(NO_3)_3 \cdot 9H_2O$ (10 mol%, 0.1 equiv.) and terpyridine (10 mol%, 0.1 equiv.) were added in an oven-dried 8-mL test vial containing a Teflon®-coated magnetic stir bar. The vial was evacuated and backfilled with N2 (repeated for 4 times), followed by addition of alkene (0.1 mmol, 1.0 equiv.), $TMSN_3$ (0.40 mmol, 4.0 equiv.), 4-F-thiolphenol (10 mol%, 0.1 equiv.) in $HCCl_3/H_2O$ (19:1, 0.1 M in regard to alkenes) via syringe under N2. The reaction mixture was placed under 427nm Kessil® light (25%) with proper sealing (see General Procedure A) and allowed to react at room temperature for 36 h. Following this, the reaction mixture was filtered through a pad of celite and rinsed with DCM. The concentrated filtrate was then purified through preparatory thin-layer chromatography (with eluent of Hexane) to give the corresponding hydroazidation products as colorless oil.

Yield 42%, 6.8 mg, **r.r.** > 20:1 (determined by ^{1}H NMR).

^{1}H NMR (600 MHz, $CDCl_3$) δ 7.34-7.29 (m, 2H), 7.27 – 7.24 (m, 1H), 7.22 – 7.18 (m, 2H), 3.69 (q, *J* = 6.7 Hz, 1H), 2.84 (dd, *J* = 13.7, 7.3 Hz, 1H), 2.73 (dd, *J* = 13.7, 6.5 Hz, 1H), 1.26 (d, *J* = 6.5 Hz, 3H).

^{13}C NMR (151 MHz, $CDCl_3$) δ 137.76, 129.29, 128.50, 126.72, 59.04, 42.57, 19.12.

HRMS APCI: $[M-N2+H]^+$ calcd. for C9H12N: 134.0964; Found 134.0964

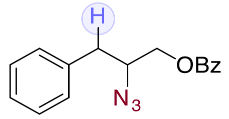
45

Prepared according to General Procedure A (substrate 0.1 mmol scale): $Fe(NO_3)_3 \cdot 9H_2O$ (10 mol%, 0.1 equiv.) and terpyridine (10 mol%, 0.1 equiv.) were added in an oven-dried 8-mL test vial containing a Teflon®-coated magnetic stir bar. The vial was evacuated and backfilled with N2 (repeated for 4 times), followed by addition of alkene (0.1 mmol, 1.0 equiv.), $TMSN_3$ (0.40 mmol, 4.0 equiv.), 4-F-thiolphenol (10 mol%, 0.1 equiv.) in $HCCl_3/H_2O$ (19:1, 0.1 M in regard to alkenes) via syringe under N2. The reaction mixture was placed under 427nm Kessil® light (25%) with proper sealing (see General Procedure A) and allowed to react at room temperature for 36 h. Following this, the reaction mixture was filtered through a pad of celite and rinsed with DCM. The concentrated filtrate was then purified through preparatory thin-layer chromatography (with eluent of Hex: EA = 10:1) to give the corresponding hydroazidation products as colorless oil.

Yield 60%, 16.9 mg, **r.r.** > 20:1 (determined by ^{1}H NMR).

^{1}H NMR (600 MHz, $CDCl_3$) δ 8.10 – 8.04 (m, 2H), 7.62 – 7.56 (m, 1H), 7.49-7.44 (m, 2H), 7.37-7.32 (m, 2H), 7.30 – 7.23 (m, 3H), 4.49 (dd, *J* = 11.5, 3.6 Hz, 1H), 4.29 (dd, *J* = 11.6, 7.3 Hz, 1H), 3.97 (qd, *J* = 7.3, 3.5 Hz, 1H), 2.98 – 2.88 (m, 2H).

^{13}C NMR (151 MHz, $CDCl_3$) δ 166.18, 136.51, 133.35, 129.80, 129.52, 129.26, 128.79, 128.54, 127.14, 66.42, 62.01, 37.37.

HRMS ESI: $[M+H]^+$ calcd. for C16H16N3O2: 282.1237; Found 282.1232

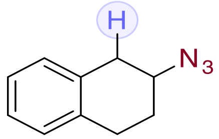
46

Prepared according to General Procedure A (substrate 0.1 mmol scale): $Fe(NO_3)_3 \cdot 9H_2O$ (10 mol%, 0.1 equiv.) and terpyridine (10 mol%, 0.1 equiv.) were added in an oven-dried 8-mL test vial containing a Teflon®-coated magnetic stir bar. The vial was evacuated and backfilled with N2 (repeated for 4 times), followed by addition of alkene (0.1 mmol, 1.0 equiv.), $TMSN_3$ (0.40 mmol, 4.0 equiv.), 4-F-thiolphenol (10 mol%, 0.1 equiv.) in $HCCl_3/H_2O$ (19:1, 0.1 M in regard to alkenes) via syringe under N2. The reaction mixture was placed under 427nm Kessil® light (25%) with proper sealing (see General Procedure A) and allowed to react at room temperature for 36 h. Following this, the reaction mixture was filtered through a pad of celite and rinsed with DCM.

The concentrated filtrate was then purified through preparatory thin-layer chromatography (with eluent of Hexane) to give the corresponding hydroazidation products as colorless oil.
Yield 57%, 9.9 mg, **r.r.** > 20:1 (determined by ^{1}H NMR).
^{1}H NMR (600 MHz, CDCl$_3$) δ 7.22 – 6.91 (m, 5H), 3.93-3.84 (m, 1H), 3.13-3.05 (m, 1H), 3.03-2.92 (m, 1H), 2.89-2.80 (m, 2H), 2.17 – 2.09 (m, 1H), 1.94-1.84 (m, 1H).
^{13}C NMR (151 MHz, CDCl$_3$) δ 135.18, 133.37, 129.29, 128.75, 126.34, 126.08, 57.10, 34.67, 28.09, 27.08.
HRMS ESI: $[M-N2+H]^+$ calcd. for C10H12N: 146.0964; Found 146.0962

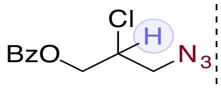

47

Prepared according to General Procedure A (substrate 0.1 mmol scale): $Fe(NO_3)_3·9H_2O$ (10 mol%, 0.1 equiv.) and terpyridine (10 mol%, 0.1 equiv.) were added in an oven-dried 8-mL test vial containing a Teflon®-coated magnetic stir bar. The vial was evacuated and backfilled with N2 (repeated for 4 times), followed by addition of alkene (0.1 mmol, 1.0 equiv.), $TMSN_3$ (0.40 mmol, 4.0 equiv.), 4-F-thiolphenol (10 mol%, 0.1 equiv.) in $HCCl_3/H_2O$ (19:1, 0.1 M in regard to alkenes) via syringe under N2. The reaction mixture was placed under 427nm Kessil® light (50%) with proper sealing (see General Procedure A) and allowed to react at room temperature for 60 h. Following this, the reaction mixture was filtered through a pad of celite and rinsed with DCM. The concentrated filtrate was then purified through preparatory thin-layer chromatography (with eluent of Hex: EA = 10:1) to give the corresponding hydroazidation products as colorless oil.
Yield 37%, 8.9 mg, **r.r.** > 20:1 (determined by ^{1}H NMR).
^{1}H NMR (600 MHz, CDCl$_3$) ^{1}H NMR (600 MHz, Chloroform-*d*) δ 8.08 – 8.02 (m, 2H), 7.63 – 7.56 (m, 1H), 7.51-7.43 (m, 2H), 4.61 – 4.52 (m, 2H), 4.31 (p, J = 5.7 Hz, 1H), 3.75 – 3.64 (m, 2H).
^{13}C NMR (151 MHz, CDCl$_3$) δ 165.85, 133.52, 129.77, 129.27, 128.57, 64.91, 56.45, 53.96.
HRMS ESI: $[M+H]^+$ calcd. for C10H11ClN3O2: 240.0534; Found 240.0529

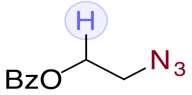

48

Prepared according to General Procedure A (substrate 0.1 mmol scale): $Fe(NO_3)_3·9H_2O$ (10 mol%, 0.1 equiv.) and terpyridine (10 mol%, 0.1 equiv.) were added in an oven-dried 8-mL test vial containing a Teflon®-coated magnetic stir bar. The vial was evacuated and backfilled with N2 (repeated for 4 times), followed by addition of alkene (0.1 mmol, 1.0 equiv.), $TMSN_3$ (0.40 mmol, 4.0 equiv.), 4-F-thiolphenol (10 mol%, 0.1 equiv.) in $HCCl_3/H_2O$ (19:1, 0.1 M in regard to alkenes) via syringe under N2. The reaction mixture was placed under 427nm Kessil® light (50%) with proper sealing (see General Procedure A) and allowed to react at room temperature for 60 h. Following this, the reaction mixture was filtered through a pad of celite and rinsed with DCM. The concentrated filtrate was then purified through preparatory thin-layer chromatography (with eluent of Hex: EA = 10:1) to give the corresponding hydroazidation products as colorless oil.
Yield 51%, 9.8 mg, **r.r.** > 20:1 (determined by ^{1}H NMR).
^{1}H NMR (600 MHz, CDCl$_3$) δ 8.11 – 8.03 (m, 2H), 7.61 – 7.56 (m, 1H), 7.49 – 7.42 (m, 2H), 4.55 – 4.47 (m, 2H), 3.65 – 3.54 (m, 2H).
^{13}C NMR (151 MHz, CDCl$_3$) ^{13}C NMR (151 MHz, CDCl$_3$) δ 166.28, 133.31, 129.78, 129.53, 128.50, 63.65, 49.99.
HRMS ESI: $[M+H]^+$ calcd. for C9H10N3O2: 192.0768; Found 192.0764

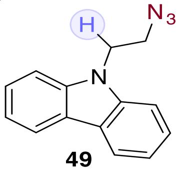

49

Prepared according to General Procedure A (substrate 0.1 mmol scale): $Fe(OAc)_2$ (10 mol%, 0.1 equiv.) and terpyridine (10 mol%, 0.1 equiv.) were added in an oven-dried 8-mL test vial containing a Teflon®-coated magnetic stir bar. The vial was evacuated and backfilled with N2 (repeated for 4 times), followed by addition

of alkene (0.1 mmol, 1.0 equiv.), $TMSN_3$ (0.40 mmol, 4.0 equiv.), 4-F-thiolphenol (10 mol%, 0.1 equiv.) in $HCCl_3/H_2O$ (19:1, 0.1 M in regard to alkenes) via syringe under N2. The reaction mixture was placed under 427nm Kessil® light (25%) with proper sealing (see General Procedure A) and allowed to react at room temperature for 36 h. Following this, the reaction mixture was filtered through a pad of celite and rinsed with DCM. The concentrated filtrate was then purified through preparatory thin-layer chromatography (with eluent of Hex: EA = 10:1) to give the corresponding hydroazidation products as colorless oil.
Yield 46%, 10.9 mg, **isolated as single regioisomer**, **r.r.** = 1.3:1 (anti-Markovnikov:Markovnikov, determined by ^{1}H NMR of crude reaction).
^{1}H NMR (600 MHz, $CDCl_3$) δ 8.15-8.07 (m, 2H), 7.53-7.47 (m, 2H), 7.46-7.42 (m, 2H), 7.29 – 7.26 (m, 2H), 4.50 (t, *J* = 6.2 Hz, 2H), 3.74 (t, *J* = 6.2 Hz, 2H).
^{13}C NMR (151 MHz, $CDCl_3$) δ 140.19, 125.95, 123.19, 120.55, 119.49, 108.40, 49.91, 42.38.
HRMS ESI: $[M+H]^+$ calcd. for C14H13N4: 237.1135; Found 237.1131

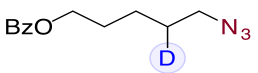

50 [89% D] Prepared according to General Procedure B (substrate 0.1 mmol scale): $Fe(OAc)_2$ (10 mol%, 0.1 equiv.) and terpyridine (10 mol%, 0.1 equiv.) were added in an oven-dried 8-mL test vial containing a Teflon®-coated magnetic stir bar. The vial was evacuated and backfilled with N2 (repeated for 4 times), followed by addition of alkene (0.1 mmol, 1.0 equiv.), $TMSN_3$ (0.40 mmol, 4.0 equiv.), 4-F-thiolphenol (10 mol%, 0.1 equiv.) in $HCCl_3/D_2O$ (19:1, 0.1 M in regard to alkenes) via syringe under N2. The reaction mixture was placed under 427nm Kessil® light (50%) with proper sealing (see General Procedure B) and allowed to react at room temperature for 60 h. Following this, the reaction mixture was filtered through a pad of celite and rinsed with DCM. The concentrated filtrate was then purified through preparatory thin-layer chromatography (with eluent of Hex: EA = 10:1) to give the corresponding hydroazidation products as colorless oil.
Yield 48%, 11.2 mg, **deuteration rate** 89% D, **r.r.** = 14:1 (determined by ^{1}H NMR).
^{1}H NMR (600 MHz, Benzene-*d*$_6$) δ 8.21 – 8.13 (m, 2H), 7.13 – 7.09 (m, 1H), 7.09 – 7.04 (m, 2H), 4.05 (t, *J* = 6.6 Hz, 2H), 2.57 (d, *J* = 6.8 Hz, 2H), 1.32 – 1.24 (m, 2H), 1.09 – 0.96 (m, 3.11H).
^{13}C NMR (151 MHz, C_6D_6) δ 165.91, 132.55, 130.79, 129.50, 128.25, 64.25, 50.60, 28.02, 27.77 (t), 22.82.
HRMS ESI: $[M+H]^+$ calcd. for C12H15DN3O2: 235.1300; Found 235.1297

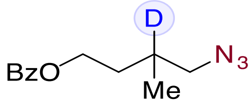

51 [82% D] Prepared according to General Procedure B (substrate 0.1 mmol scale): $Fe(OAc)_2$ (10 mol%, 0.1 equiv.) and terpyridine (10 mol%, 0.1 equiv.) were added in an oven-dried 8-mL test vial containing a Teflon®-coated magnetic stir bar. The vial was evacuated and backfilled with N2 (repeated for 4 times), followed by addition of alkene (0.1 mmol, 1.0 equiv.), $TMSN_3$ (0.40 mmol, 4.0 equiv.), 4-F-thiolphenol (10 mol%, 0.1 equiv.) in $HCCl_3/D_2O$ (19:1, 0.1 M in regard to alkenes) via syringe under N2. The reaction mixture was placed under 427nm Kessil® light (50%) with proper sealing (see General Procedure B) and allowed to react at room temperature for 60 h. Following this, the reaction mixture was filtered through a pad of celite and rinsed with DCM. The concentrated filtrate was then purified through preparatory thin-layer chromatography (with eluent of Hex: EA = 10:1) to give the corresponding hydroazidation products as colorless oil.
Yield 55%, 12.8 mg, **deuteration rate** 82% D, **r.r.** > 20:1 (determined by ^{1}H NMR).
^{1}H NMR (600 MHz, Benzene-*d*$_6$) δ 8.18 – 8.11 (m, 2H), 7.14 – 7.09 (m, 1H), 7.00-703 (m, 2H), 4.12 – 4.02 (m, 2H), 2.60 – 2.49 (m, 2H), 1.49-1.41 (m, 1.18H), 1.16 – 1.09 (m, 1H), 0.61 (s, 3H).
^{13}C NMR (151 MHz, C_6D_6) δ 165.84, 132.59, 130.67, 129.50, 62.37, 56.73, 30.11 (t), 29.85, 16.82.
HRMS ESI: $[M+H]^+$ calcd. for C12H15DN3O2: 235.1300; Found 235.1297

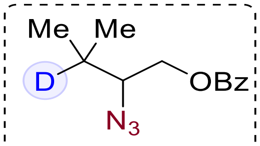

52 [80% D] Prepared according to General Procedure B (substrate 0.1 mmol scale): $Fe(OAc)_2$ (10 mol%, 0.1 equiv.) and terpyridine (10 mol%, 0.1 equiv.) were added in an oven-dried 8-mL test vial containing a Teflon®-coated magnetic stir bar. The vial was evacuated and backfilled with N2 (repeated for 4 times), followed by addition of alkene (0.1 mmol, 1.0 equiv.), $TMSN_3$ (0.40 mmol, 4.0 equiv.), 4-F-thiolphenol (10 mol%, 0.1 equiv.) in $HCCl_3/D_2O$ (19:1, 0.1 M in regard to alkenes) via syringe under N2. The reaction mixture was placed under 427nm Kessil® light (50%) with proper sealing (see General Procedure B) and allowed to react at room temperature for 60 h. Following this, the reaction mixture was filtered through a pad of celite and rinsed with DCM. The concentrated filtrate was then purified through preparatory thin-layer chromatography (with eluent of Hex: EA = 10:1) to give the corresponding hydroazidation products as colorless oil.

Yield 42%, 9.8 mg, **deuteration rate** 80% D, **r.r.** > 20:1 (determined by ^{1}H NMR).

^{1}H NMR (600 MHz, Benzene-*d*$_6$) δ 8.25 – 8.19 (m, 2H), 7.13 – 7.09 (m, 1H), 7.08-7.04 (m, 2H), 4.30 – 4.21 (m, 1H), 4.02 (dd, J = 11.5, 8.5 Hz, 1H), 2.96 (dd, J = 8.6, 3.3 Hz, 1H), 1.40-1.36 (m, 0.20H), 0.67 (s, 3H), 0.62 (s, 3H).

^{13}C NMR (151 MHz, C_6D_6) δ 165.65, 132.88, 130.06, 129.74, 66.67, 65.89, 29.07 (t), 18.84, 17.54.

HRMS ESI: $[M+H]^+$ calcd. for C12H15DN3O2: 235.1300; Found 235.1298

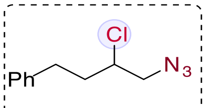

53 Prepared according to General Procedure C (substrate 0.1 mmol scale): $Fe(OAc)_2$ (10 mol%, 0.1 equiv.), terpyridine (10 mol%, 0.1 equiv.), NCS (0.15 mmol, 1.5 equiv.) were added in an oven-dried 8-mL test vial containing a Teflon®-coated magnetic stir bar. The vial was evacuated and backfilled with N2 (repeated for 4 times), followed by addition of alkene (0.1 mmol, 1.0 equiv.) and $TMSN_3$ (0.2 mmol, 2.0 equiv.) in DCM (0.1 M in regard to alkenes) via syringe under N2. The reaction mixture was placed under 427nm Kessil® light (25%) with proper sealing (see General Procedure C) and allowed to react at room temperature for 24 h. Following this, the reaction mixture was filtered through a pad of celite and rinsed with DCM. The concentrated filtrate was then purified through preparatory thin-layer chromatography (with eluent of Hexane) to give the corresponding chloroazidation products as colorless oil.

Yield 52%, 10.9 mg.

^{1}H NMR (600 MHz, $CDCl_3$) δ 7.33-7.28 (m, 2H), 7.25 – 7.18 (m, 3H), 3.97 – 3.90 (m, 1H), 3.56 – 3.47 (m, 2H), 2.94-2.87 (m, 1H), 2.79-2.72 (m, 1H), 2.14-1.99 (m, 2H).

^{13}C NMR (151 MHz, $CDCl_3$) δ 140.32, 128.63, 128.52, 126.35, 60.10, 57.17, 36.98, 32.21.

HRMS APCI: $[M-N2+H]^+$ calcd. for C10H13ClNO: 182.0731; Found 182.0730

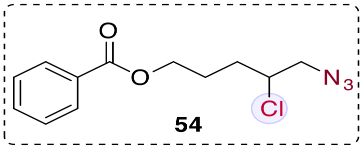

54 Prepared according to General Procedure C (substrate 0.1 mmol scale): $Fe(OAc)_2$ (10 mol%, 0.1 equiv.), terpyridine (10 mol%, 0.1 equiv.), NCS (0.15 mmol, 1.5 equiv.) were added in an oven-dried 8-mL test vial containing a Teflon®-coated magnetic stir bar. The vial was evacuated and backfilled with N2 (repeated for 4 times), followed by addition of alkene (0.1 mmol, 1.0 equiv.) and $TMSN_3$ (0.2 mmol, 2.0 equiv.) in DCM (0.1 M in regard to alkenes) via syringe under N2. The reaction mixture was placed under 427nm Kessil® light (25%) with proper sealing (see General Procedure C) and allowed to react at room temperature for 24 h. Following this, the reaction mixture was filtered through a pad of celite and rinsed with DCM. The concentrated filtrate was then purified through preparatory thin-layer chromatography (with eluent of Hex: EA = 10:1) to give the corresponding chloroazidation products as colorless oil.

Yield 70%, 18.7 mg.

^{1}H NMR (600 MHz, $CDCl_3$) δ 8.06 – 8.01 (m, 2H), 7.59-7.54 (m, 1H), 7.48-7.42 (m, 2H), 4.41 – 4.32 (m, 2H), 4.08-4.02 (m, 1H), 3.60 – 3.48 (m, 2H), 2.11-2.05 (m, 1H), 2.03 – 1.96 (m, 1H), 1.96 – 1.83 (m, 2H).

^{13}C NMR (151 MHz, $CDCl_3$) δ 166.54, 133.06, 130.12, 129.58, 128.43, 64.02, 60.37, 57.06, 32.10, 25.62.
HRMS ESI: $[M+H]^+$ calcd. for C12H15ClN3O2: 268.0847; Found 268.0843

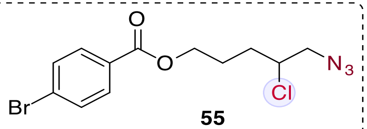

55

Prepared according to General Procedure C (substrate 0.1 mmol scale): Fe(OAc)$_2$ (10 mol%, 0.1 equiv.), terpyridine (10 mol%, 0.1 equiv.), NCS (0.15 mmol, 1.5 equiv.) were added in an oven-dried 8-mL test vial containing a Teflon®-coated magnetic stir bar. The vial was evacuated and backfilled with N2 (repeated for 4 times), followed by addition of alkene (0.1 mmol, 1.0 equiv.) and TMSN$_3$ (0.2 mmol, 2.0 equiv.) in DCM (0.1 M in regard to alkenes) via syringe under N2. The reaction mixture was placed under 427nm Kessil® light (25%) with proper sealing (see General Procedure C) and allowed to react at room temperature for 24 h. Following this, the reaction mixture was filtered through a pad of celite and rinsed with DCM. The concentrated filtrate was then purified through preparatory thin-layer chromatography (with eluent of Hex: EA = 10:1) to give the corresponding chloroazidation products as colorless oil.
Yield 65%, 22.4 mg.
^{1}H NMR (600 MHz, $CDCl_3$) δ 7.89 (dd, J = 8.2, 1.2 Hz, 2H), 7.59 (dd, J = 8.3, 1.2 Hz, 2H), 4.41 – 4.30 (m, 2H), 4.07-4.00 (m, 1H), 3.59-3.49 (m, 2H), 2.10 – 2.02 (m, 1H), 2.01-1.96 (m, 1H), 1.95 – 1.81 (m, 2H).
^{13}C NMR (151 MHz, $CDCl_3$) δ 165.81, 131.80, 131.11, 129.00, 128.20, 64.30, 60.26, 57.03, 32.05, 25.56.
HRMS ESI: $[M+H]^+$ calcd. for C12H14BrClN3O2: 345.9952; Found 345.9945

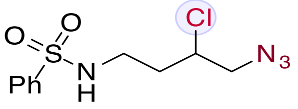

56

Prepared according to General Procedure C (substrate 0.1 mmol scale): Fe(OAc)$_2$ (10 mol%, 0.1 equiv.), terpyridine (10 mol%, 0.1 equiv.), NCS (0.15 mmol, 1.5 equiv.) were added in an oven-dried 8-mL test vial containing a Teflon®-coated magnetic stir bar. The vial was evacuated and backfilled with N2 (repeated for 4 times), followed by addition of alkene (0.1 mmol, 1.0 equiv.) and TMSN$_3$ (0.2 mmol, 2.0 equiv.) in DCM (0.1 M in regard to alkenes) via syringe under N2. The reaction mixture was placed under 427nm Kessil® light (25%) with proper sealing (see General Procedure C) and allowed to react at room temperature for 24 h. Following this, the reaction mixture was filtered through a pad of celite and rinsed with DCM. The concentrated filtrate was then purified through preparatory thin-layer chromatography (with eluent of Hex: EA = 5:1) to give the corresponding chloroazidation products as colorless oil.
Yield 58%, 16.7 mg.
^{1}H NMR (600 MHz, $CDCl_3$) δ 7.92 – 7.85 (m, 2H), 7.64 – 7.59 (m, 1H), 7.54 (t, J = 7.7 Hz, 2H), 4.75-5.65 (t, J = 6.5 Hz, 1H), 4.12 – 4.04 (m, 1H), 3.51 (d, J = 5.5 Hz, 2H), 3.18 (m, 2H), 2.10-2.02 (m, 1H), 1.88-1.79 (m, 1H).
^{13}C NMR (151 MHz, $CDCl_3$) δ 139.60, 132.95, 129.30, 127.06, 57.77, 56.87, 40.17, 35.27.
HRMS ESI: $[M+H]^+$ calcd. for C10H14ClN4O2S: 289.0521; Found 289.0515

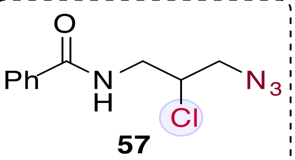

57

Prepared according to General Procedure C (substrate 0.1 mmol scale): Fe(OAc)$_2$ (10 mol%, 0.1 equiv.), terpyridine (10 mol%, 0.1 equiv.), NCS (0.15 mmol, 1.5 equiv.) were added in an oven-dried 8-mL test vial containing a Teflon®-coated magnetic stir bar. The vial was evacuated and backfilled with N2 (repeated for 4 times), followed by addition of alkene (0.1 mmol, 1.0 equiv.) and TMSN$_3$ (0.2 mmol, 2.0 equiv.) in DCM (0.1 M in regard to alkenes) via syringe under N2. The reaction mixture was placed under 427nm Kessil® light (25%) with proper sealing (see General Procedure C) and allowed to react at room temperature for 48 h. Following this, the reaction mixture was filtered through a pad of celite and rinsed with DCM. The concentrated filtrate was then

purified through preparatory thin-layer chromatography (with eluent of Hex: EA = 5:1) to give the corresponding chloroazidation products as colorless oil.
Yield 56%, 13.4 mg.
^{1}H NMR (600 MHz, Chloroform-*d*) δ 7.81 – 7.75 (m, 2H), 7.57 – 7.49 (m, 1H), 7.48 – 7.42 (m, 2H), 6.59 (s, 1H), 4.31-4.26 (m, 1H), 3.96-3.89 (m, 1H), 3.72 – 3.63 (m, 2H), 3.64-3.56 (m, 1H).
^{13}C NMR (151 MHz, CDCl$_3$) δ 167.76, 133.73, 132.00, 128.76, 127.00, 59.65, 54.89, 43.71.
HRMS ESI: $[M+H]^+$ calcd. for C10H10ClN4O2: 239.0694; Found 239.0689

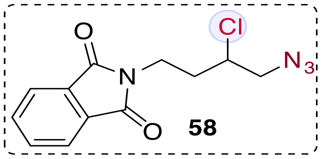

58

Prepared according to General Procedure C (substrate 0.1 mmol scale): $Fe(OAc)_2$ (10 mol%, 0.1 equiv.), terpyridine (10 mol%, 0.1 equiv.), alkene (0.1 mmol, 1.0 equiv.), NCS (0.15 mmol, 1.5 equiv.) were added in an oven-dried 8-mL test vial containing a Teflon®-coated magnetic stir bar. The vial was evacuated and backfilled with N2 (repeated for 4 times), followed by addition of $TMSN_3$ (0.2 mmol, 2.0 equiv.) in DCM (0.1 M in regard to alkenes) via syringe under N2. The reaction mixture was placed under 427nm Kessil® light (25%) with proper sealing (see General Procedure C) and allowed to react at room temperature for 24 h. Following this, the reaction mixture was filtered through a pad of celite and rinsed with DCM. The concentrated filtrate was then purified through preparatory thin-layer chromatography (with eluent of Hex: EA = 5:1) to give the corresponding chloroazidation products as colorless oil.
Yield 72%, 20.1 mg.
^{1}H NMR (600 MHz, CDCl$_3$) δ 7.85 (dd, *J* = 5.4, 3.1 Hz, 2H), 7.73 (dd, *J* = 5.5, 3.0 Hz, 2H), 4.05-3.98 (m, 1H), 3.93-3.82 (m, 2H), 3.62 – 3.53 (m, 2H), 2.29-2.17 (m, 1H), 2.14-2.05 (m, 1H).
^{13}C NMR (151 MHz, CDCl$_3$) δ 168.21, 134.15, 131.98, 123.41, 57.99, 56.83, 35.16, 34.11.
HRMS ESI: $[M+H]^+$ calcd. for C12H12ClN4O2: 279.0643; Found 279.0640

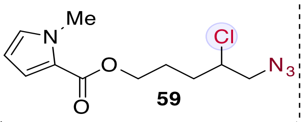

59

Prepared according to General Procedure C (substrate 0.1 mmol scale): $Fe(OAc)_2$ (10 mol%, 0.1 equiv.), terpyridine (10 mol%, 0.1 equiv.), NCS (0.15 mmol, 1.5 equiv.) were added in an oven-dried 8-mL test vial containing a Teflon®-coated magnetic stir bar. The vial was evacuated and backfilled with N2 (repeated for 4 times), followed by addition of alkene (0.1 mmol, 1.0 equiv.) and $TMSN_3$ (0.2 mmol, 2.0 equiv.) in DCM (0.1 M in regard to alkenes) via syringe under N2. The reaction mixture was placed under 427nm Kessil® light (25%) with proper sealing (see General Procedure C) and allowed to react at room temperature for 48 h. Following this, the reaction mixture was filtered through a pad of celite and rinsed with DCM. The concentrated filtrate was then purified through preparatory thin-layer chromatography (with eluent of Hex: EA = 10:1) to give the corresponding chloroazidation products as colorless oil.
Yield 37%, 10.0 mg.
^{1}H NMR (600 MHz, CDCl$_3$) δ 6.94 (dd, *J* = 4.0, 1.8 Hz, 1H), 6.79 (t, *J* = 2.1 Hz, 1H), 6.12 (dd, *J* = 4.0, 2.5 Hz, 1H), 4.31-4.20 (m, 2H), 4.08 – 4.02 (m, 1H), 3.92 (s, 3H), 3.58 – 3.48 (m, 2H), 2.05 – 1.93 (m, 2H), 1.91 – 1.79 (m, 2H).
^{13}C NMR (151 MHz, CDCl$_3$) δ 161.18, 129.68, 122.29, 117.86, 107.91, 62.78, 60.44, 57.09, 36.83, 32.16, 25.70.
HRMS ESI: $[M+H]^+$ calcd. for C11H16ClN4O2: 271.0956; Found 271.0951

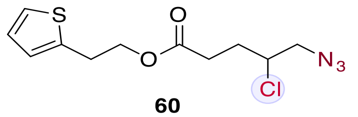

60

Prepared according to General Procedure C(substrate 0.1 mmol scale): $Fe(OAc)_2$ (10 mol%, 0.1 equiv.), terpyridine (10 mol%, 0.1 equiv.), NCS (0.15 mmol, 1.5 equiv.) were added in an oven-dried 8-mL test vial containing a Teflon®-coated magnetic stir bar. The vial was evacuated and backfilled with

N2 (repeated for 4 times), followed by addition of alkene (0.1 mmol, 1.0 equiv.) and $TMSN_3$ (0.2 mmol, 2.0 equiv.) in DCM (0.1 M in regard to alkenes) via syringe under N2. The reaction mixture was placed under 427nm Kessil® light (25%) with proper sealing (see General Procedure C) and allowed to react at room temperature for 24 h. Following this, the reaction mixture was filtered through a pad of celite and rinsed with DCM. The concentrated filtrate was then purified through preparatory thin-layer chromatography (with eluent of Hex: EA = 10:1) to give the corresponding chloroazidation products as colorless oil.
Yield 45%, 13.0 mg.
^{1}H NMR (600 MHz, $CDCl_3$) δ 7.17 (dd, J = 5.1, 1.2 Hz, 1H), 6.95 (dd, J = 5.1, 3.4 Hz, 1H), 6.86 (dt, J = 3.5, 1.0 Hz, 1H), 4.33 (t, J = 6.6 Hz, 2H), 4.07-3.98 (m, 1H), 3.55 – 3.48 (m, 2H), 3.20-3.14 (m, 2H), 2.63 – 2.51 (m, 2H), 2.21-2.13 (m, 1H), 1.99-1.91 (m, 1H).
^{13}C NMR (151 MHz, $CDCl_3$) δ 172.27, 139.77, 126.93, 125.61, 124.12, 64.87, 59.87, 57.02, 30.68, 30.41, 29.28.
HRMS ESI: $[M+H]^+$ calcd. for C11H15ClN3O2S: 288.0568; Found 288.0563

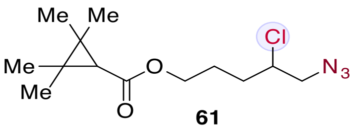

61

Prepared according to General Procedure C (substrate 0.1 mmol scale): $Fe(OAc)_2$ (10 mol%, 0.1 equiv.), terpyridine (10 mol%, 0.1 equiv.), NCS (0.15 mmol, 1.5 equiv.) were added in an oven-dried 8-mL test vial containing a Teflon®-coated magnetic stir bar. The vial was evacuated and backfilled with N2 (repeated for 4 times), followed by addition of alkene (0.1 mmol, 1.0 equiv.) and $TMSN_3$ (0.2 mmol, 2.0 equiv.) in DCM (0.1 M in regard to alkenes) via syringe under N2. The reaction mixture was placed under 427nm Kessil® light (25%) with proper sealing (see General Procedure C) and allowed to react at room temperature for 48 h. Following this, the reaction mixture was filtered through a pad of celite and rinsed with DCM. The concentrated filtrate was then purified through column chromatography (with eluent of Hex: EA = 20:1) to give the corresponding chloroazidation products as colorless oil.
Yield 51%, 14.7 mg.
^{1}H NMR (600 MHz, $CDCl_3$) δ 4.1-3.95 (m, 3H), 3.57 – 3.47 (m, 2H), 1.94-1.86 (m, 2H), 1.80 – 1.73 (m, 2H), 1.23 (s, 6H), 1.18 (s, 6H).
^{13}C NMR (151 MHz, $CDCl_3$) δ 172.15, 62.63, 60.47, 57.08, 35.68, 32.13, 30.21, 25.64, 23.54, 16.58.
HRMS ESI: $[M+H]^+$ calcd. for C13H23ClN3O2: 288.1473; Found 288.1469

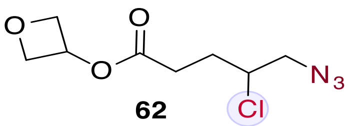

62

Prepared according to General Procedure C (substrate 0.1 mmol scale): $Fe(OAc)_2$ (10 mol%, 0.1 equiv.), terpyridine (10 mol%, 0.1 equiv.), NCS (0.15 mmol, 1.5 equiv.) were added in an oven-dried 8-mL test vial containing a Teflon®-coated magnetic stir bar. The vial was evacuated and backfilled with N2 (repeated for 4 times), followed by addition of alkene (0.1 mmol, 1.0 equiv.) and $TMSN_3$ (0.2 mmol, 2.0 equiv.) in DCM (0.1 M in regard to alkenes) via syringe under N2. The reaction mixture was placed under 427nm Kessil® light (25%) with proper sealing (see General Procedure C) and allowed to react at room temperature for 24 h. Following this, the reaction mixture was filtered through a pad of celite and rinsed with DCM. The concentrated filtrate was then purified through column chromatography (with eluent of Hex: EA = 10:1) to give the corresponding chloroazidation products as colorless oil.
Yield 61%, 14.2 mg.
^{1}H NMR (600 MHz, $CDCl_3$) δ 5.45 (p, J = 5.8 Hz, 1H), 4.93-4.85 (m, 2H), 4.66-4.60 (m, 2H), 4.10-4.02 (m, 1H), 3.58-3.49 (m, 2H), 2.68 – 2.53 (m, 2H), 2.24-2.16 (m, 1H), 2.01 – 1.92 (m, 1H).
^{13}C NMR (151 MHz, $CDCl_3$) δ 171.75, 77.46, 77.45, 68.14, 59.64, 56.97, 30.39, 30.20.
HRMS ESI: $[M+H]^+$ calcd. for C8H13ClN3O3: 234.0640; Found 234.0636

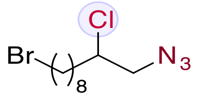

63

Prepared according to General Procedure C (substrate 0.1 mmol scale): $Fe(OAc)_2$ (10 mol%, 0.1 equiv.), terpyridine (10 mol%, 0.1 equiv.), NCS (0.15 mmol, 1.5 equiv.) were added in an oven-dried 8-mL test vial containing a Teflon®-coated magnetic stir bar. The vial was evacuated and backfilled with N2 (repeated for 4 times), followed by addition of alkene (0.1 mmol, 1.0 equiv.) and $TMSN_3$ (0.2 mmol, 2.0 equiv.) in DCM (0.1 M in regard to alkenes) via syringe under N2. The reaction mixture was placed under 427nm Kessil® light (25%) with proper sealing (see General Procedure C) and allowed to react at room temperature for 48 h. Following this, the reaction mixture was filtered through a pad of celite and rinsed with DCM. The concentrated filtrate was then purified through preparatory thin-layer chromatography (with eluent of Hexane) to give the corresponding chloroazidation products as colorless oil.
Yield 57%, 16.9 mg.
^{1}H NMR (600 MHz, $CDCl_3$) δ 4.00-3.94 (m, 1H), 3.53-3.47 (m, 2H), 3.41 (t, J = 6.8 Hz, 2H), 1.88 – 1.83 (m, 2H), 1.81 – 1.77 (m, 1H), 1.75 – 1.67 (m, 1H), 1.45 – 1.29 (m, 10H).
^{13}C NMR (151 MHz, $CDCl_3$) δ 61.06, 57.13, 35.37, 34.00, 32.76, 29.19, 28.88, 28.62, 28.09, 26.07.
HRMS APCI: $[M-N2+H]^+$ calcd. for C10H20BrClN: 268.0462; Found 268.0457

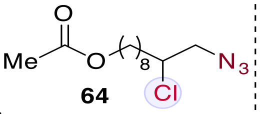

64

Prepared according to General Procedure C (substrate 0.1 mmol scale): $Fe(OAc)_2$ (10 mol%, 0.1 equiv.), terpyridine (10 mol%, 0.1 equiv.), NCS (0.15 mmol, 1.5 equiv.) were added in an oven-dried 8-mL test vial containing a Teflon®-coated magnetic stir bar. The vial was evacuated and backfilled with N2 (repeated for 4 times), followed by addition of alkene (0.1 mmol, 1.0 equiv.) and $TMSN_3$ (0.2 mmol, 2.0 equiv.) in DCM (0.1 M in regard to alkenes) via syringe under N2. The reaction mixture was placed under 427nm Kessil® light (25%) with proper sealing (see General Procedure C) and allowed to react at room temperature for 24 h. Following this, the reaction mixture was filtered through a pad of celite and rinsed with DCM. The concentrated filtrate was then purified through column chromatography (with eluent of Hex: EA = 20:1) to give the corresponding chloroazidation products as colorless oil.
Yield 58%, 16.0 mg.
^{1}H NMR (600 MHz, $CDCl_3$) δ 3.98 (t, J = 6.8 Hz, 2H), 3.99-3.94 (m, 1H), 3.53-3.46 (m, 2H), 1.98 (s, 3H), 1.75 – 1.70 (m, 1H), 1.68 – 1.62 (m, 1H), 1.64-1.60 (m, 2H), 1.50 – 1.43 (m, 1H), 1.36 – 1.23 (m, 10H).
^{13}C NMR (151 MHz, $CDCl_3$) δ 171.26, 64.58, 61.06, 57.12, 35.37, 29.25, 29.10, 28.90, 28.57, 26.08, 25.85, 21.03.
HRMS ESI: $[M+H]^+$ calcd. for C12H15ClN3O2: 276.1473; Found 276.1469

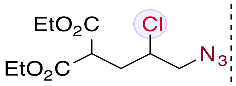

65

Prepared according to General Procedure C (substrate 0.1 mmol scale): $Fe(OAc)_2$ (10 mol%, 0.1 equiv.), terpyridine (10 mol%, 0.1 equiv.), NCS (0.15 mmol, 1.5 equiv.) were added in an oven-dried 8-mL test vial containing a Teflon®-coated magnetic stir bar. The vial was evacuated and backfilled with N2 (repeated for 4 times), followed by addition of alkene (0.1 mmol, 1.0 equiv.) and $TMSN_3$ (0.2 mmol, 2.0 equiv.) in DCM (0.1 M in regard to alkenes) via syringe under N2. The reaction mixture was placed under 427nm Kessil® light (25%) with proper sealing (see General Procedure C) and allowed to react at room temperature for 48 h. Following this, the reaction mixture was filtered through a pad of celite and rinsed with DCM. The concentrated filtrate was then purified through column chromatography (with eluent of Hex: EA = 10:1) to give the corresponding chloroazidation products as colorless oil.
Yield 57%, 15.8 mg.
^{1}H NMR (600 MHz, $CDCl_3$) δ 4.29 – 4.16 (m, 4H), 4.11-4.03 (m, 1H), 3.74-3.61 (m, 1H), 3.58-3.52 (m, 2H), 2.49-2.40 (m, 1H), 2.22-2.14 (m, 1H), 1.31-1.24 (m, 6H).
^{13}C NMR (151 MHz, $CDCl_3$) δ 168.68, 168.51, 61.89, 61.85, 58.36, 57.05, 48.96, 34.34, 14.04, 14.01.

HRMS ESI: $[M+H]^+$ calcd. for C12H17ClN3O4: 278.0902; Found 278.0898

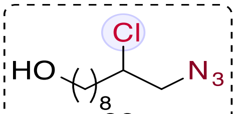

66

Prepared according to General Procedure C (substrate 0.1 mmol scale $Fe(OAc)_2$ (10 mol%, 0.1 equiv.), terpyridine (10 mol%, 0.1 equiv.), NCS (0.15 mmol, 1.5 equiv.) were added in an oven-dried 8-mL test vial containing a Teflon®-coated magnetic stir bar. The vial was evacuated and backfilled with N2 (repeated for 4 times), followed by addition of alkene (0.1 mmol, 1.0 equiv.) and $TMSN_3$ (0.2 mmol, 2.0 equiv.) in DCM (0.1 M in regard to alkenes) via syringe under N2. The reaction mixture was placed under 427nm Kessil® light (25%) with proper sealing (see General Procedure C) and allowed to react at room temperature for 24 h. Following this, the reaction mixture was filtered through a pad of celite and rinsed with DCM. The concentrated filtrate was then purified through column chromatography (with eluent of Hex: EA = 10:1) to give the corresponding chloroazidation products as colorless oil.
Yield 76%, 17.8 mg.
^{1}H NMR (600 MHz, $CDCl_3$) δ 4.01-3.94 (m, 1H), 3.64 (t, J = 6.6 Hz, 2H), 3.53-3.47 (m, 2H), 1.83-1.76 (m, 1H), 1.75 – 1.67 (m, 1H), 1.59 – 1.52 (m, 3H), 1.42 – 1.30 (m, 10H).
^{13}C NMR (151 MHz, $CDCl_3$) δ 63.04, 61.08, 57.13, 35.39, 32.74, 29.34, 29.27, 28.93, 26.10, 25.68.
HRMS ESI: $[M+H]^+$ calcd. for C10H21ClN3O: 234.1368; Found 234.1369

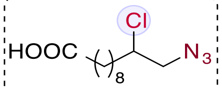

67

Prepared according to General Procedure C (substrate 0.1 mmol scale): $Fe(OAc)_2$ (10 mol%, 0.1 equiv.), terpyridine (10 mol%, 0.1 equiv.), NCS (0.15 mmol, 1.5 equiv.) were added in an oven-dried 8-mL test vial containing a Teflon®-coated magnetic stir bar. The vial was evacuated and backfilled with N2 (repeated for 4 times), followed by addition of alkene (0.1 mmol, 1.0 equiv.) and $TMSN_3$ (0.2 mmol, 2.0 equiv.) in DCM (0.1 M in regard to alkenes) via syringe under N2. The reaction mixture was placed under 427nm Kessil® light (25%) with proper sealing (see General Procedure C) and allowed to react at room temperature for 24 h. Following this, the reaction mixture was filtered through a pad of celite and rinsed with DCM. The concentrated filtrate was then purified through column chromatography (with eluent of Hex: EA = 10:1) to give the corresponding chloroazidation products as colorless oil.
Yield 63%, 16.5 mg.
^{1}H NMR (600 MHz, $CDCl_3$) δ 4.00 – 3.95 (m, 1H), 3.54 – 3.46 (m, 2H), 2.35 (t, J = 7.5 Hz, 2H), 1.82-1.76 (m, 1H), 1.73 – 1.68 (m, 1H), 1.64-1.61 (m, 1H), 1.56 – 1.48 (m, 1H), 1.43 – 1.29 (m, 10H).
^{13}C NMR (151 MHz, $CDCl_3$) δ 179.97, 61.07, 57.13, 35.38, 34.00, 29.15, 29.08, 28.96, 28.91, 26.08, 24.61.
HRMS ESI: $[M-H]^-$ calcd. for C11H19ClN3O2: 260.1160; Found 260.1170

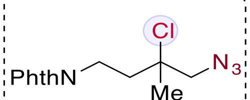

68

Prepared according to General Procedure C (substrate 0.1 mmol scale): $Fe(OAc)_2$ (10 mol%, 0.1 equiv.), terpyridine (10 mol%, 0.1 equiv.), alkene (0.1 mmol, 1.0 equiv.), NCS (0.15 mmol, 1.5 equiv.) were added in an oven-dried 8-mL test vial containing a Teflon®-coated magnetic stir bar. The vial was evacuated and backfilled with N2 (repeated for 4 times), followed by addition of $TMSN_3$ (0.2 mmol, 2.0 equiv.) in DCM (0.1 M in regard to alkenes) via syringe under N2. The reaction mixture was placed under 427nm Kessil® light (25%) with proper sealing (see General Procedure C) and allowed to react at room temperature for 24 h. Following this, the reaction mixture was filtered through a pad of celite and rinsed with DCM. The concentrated filtrate was then purified through preparatory thin-layer chromatography (with eluent of Hex: EA = 5:1) to give the corresponding chloroazidation products as colorless oil.
Yield 64%, 18.7 mg.

^{1}H NMR (600 MHz, CDCl$_3$) δ 7.85 (dd, *J* = 5.4, 3.0 Hz, 2H), 7.72 (dd, *J* = 5.5, 3.0 Hz, 2H), 3.97-3.90 (m, 1H), 3.89-3.84 (m, 1H), 3.58 – 3.48 (m, 2H), 2.25-2.18 (m, 1H), 2.15-2.08 (m, 1H), 1.65 (s, 3H).
^{13}C NMR (151 MHz, CDCl$_3$) δ 168.10, 134.06, 132.07, 123.33, 69.41, 61.81, 38.92, 33.90, 27.59.
HRMS ESI: [M+H]$^+$ calcd. for C13H14ClN4O2: 292.0800; Found 293.0794

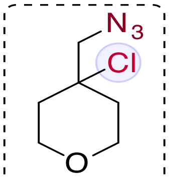

69

Prepared according to General Procedure C (substrate 0.1 mmol scale): Fe(OAc)$_2$ (10 mol%, 0.1 equiv.), terpyridine (10 mol%, 0.1 equiv.), NCS (0.15 mmol, 1.5 equiv.) were added in an oven-dried 8-mL test vial containing a Teflon®-coated magnetic stir bar. The vial was evacuated and backfilled with N2 (repeated for 4 times), followed by addition of alkene (0.1 mmol, 1.0 equiv.) and TMSN$_3$ (0.2 mmol, 2.0 equiv.) in DCM (0.1 M in regard to alkenes) via syringe under N2. The reaction mixture was placed under 427nm Kessil® light (25%) with proper sealing (see General Procedure C) and allowed to react at room temperature for 24 h. Following this, the reaction mixture was filtered through a pad of celite and rinsed with DCM. The concentrated filtrate was then purified through column chromatography (with eluent of Hex: EA = 20:1) to give the corresponding chloroazidation products as colorless oil.
Yield 45%, 7.9 mg.
^{1}H NMR (600 MHz, CDCl$_3$) δ 3.91 – 3.76 (m, 4H), 3.51 (s, 2H), 1.94 – 1.80 (m, 4H).
^{13}C NMR (151 MHz, CDCl$_3$) δ 70.54, 63.31, 62.89, 36.71.
HRMS ESI: [M+H]$^+$ calcd. for C6H11ClN3O1: 176.0585; Found 176.0584

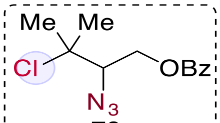

70

Prepared according to General Procedure C (substrate 0.1 mmol scale): Fe(OAc)$_2$ (10 mol%, 0.1 equiv.), terpyridine (10 mol%, 0.1 equiv.), NCS (0.15 mmol, 1.5 equiv.) were added in an oven-dried 8-mL test vial containing a Teflon®-coated magnetic stir bar. The vial was evacuated and backfilled with N2 (repeated for 4 times), followed by addition of alkene (0.1 mmol, 1.0 equiv.) and TMSN$_3$ (0.2 mmol, 2.0 equiv.) in DCM (0.1 M in regard to alkenes) via syringe under N2. The reaction mixture was placed under 427nm Kessil® light (25%) with proper sealing (see General Procedure C) and allowed to react at room temperature for 48 h. Following this, the reaction mixture was filtered through a pad of celite and rinsed with DCM. The concentrated filtrate was then purified through preparatory thin-layer chromatography (with eluent of Hex: EA = 10:1) to give the corresponding chloroazidation products as colorless oil.
Yield 59%, 15.8 mg.
^{1}H NMR (600 MHz, CDCl$_3$) δ 8.11 – 8.05 (m, 2H), 7.61 – 7.57 (m, 1H), 7.50 – 7.44 (m, 2H), 4.92 (dd, *J* = 11.5, 2.8 Hz, 1H), 4.39 (dd, *J* = 11.5, 9.3 Hz, 1H), 3.89 (dd, *J* = 9.3, 2.8 Hz, 1H), 1.71 (s, 3H), 1.64 (s, 3H).
^{13}C NMR (151 MHz, CDCl$_3$) δ 166.23, 133.44, 129.81, 129.38, 128.57, 70.04, 68.57, 65.02, 30.37, 28.80.
HRMS ESI: [M+H]$^+$ calcd. for C12H15ClN3O2: 268.0847; Found 268.0843
;

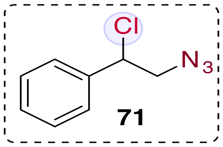

71

Prepared according to General Procedure C (substrate 0.1 mmol scale): Fe(OAc)$_2$ (10 mol%, 0.1 equiv.), terpyridine (10 mol%, 0.1 equiv.), NCS (0.15 mmol, 1.5 equiv.) were added in an oven-dried 8-mL test vial containing a Teflon®-coated magnetic stir bar. The vial was evacuated and backfilled with N2 (repeated for 4 times), followed by addition of alkene (0.1 mmol, 1.0 equiv.) and TMSN$_3$ (0.2 mmol, 2.0 equiv.) in DCM (0.1 M in regard to alkenes) via syringe under N2. The reaction mixture was placed under 427nm Kessil® light (25%) with proper sealing (see General Procedure C) and allowed to react at room temperature for 24 h. Following this, the

reaction mixture was filtered through a pad of celite and rinsed with DCM. The concentrated filtrate was then purified through preparatory thin-layer chromatography (with eluent of Hexane) to give the corresponding chloroazidation products as colorless oil.
Yield 68%, 12.3 mg.
^{1}H NMR (600 MHz, CDCl$_3$) δ 7.45 – 7.34 (m, 5H), 4.97 (t, J = 6.9 Hz, 1H), 3.77 (dd, J = 12.9, 7.5 Hz, 1H), 3.70 (dd, J = 12.9, 6.2 Hz, 1H).
^{13}C NMR (151 MHz, CDCl$_3$) δ 138.13, 129.18, 128.95, 127.26, 61.23, 57.98.
HRMS APCI: [M-N2+H]$^+$ calcd. for C8H9ClN: 154.0418; Found 154.0416

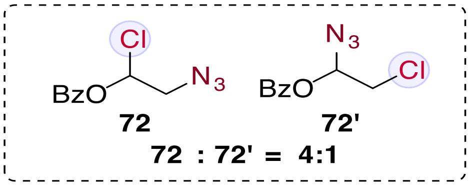

Prepared according to General Procedure C (substrate 0.1 mmol scale): Fe(OAc)$_2$ (10 mol%, 0.1 equiv.), terpyridine (10 mol%, 0.1 equiv.), NCS (0.15 mmol, 1.5 equiv.) were added in an oven-dried 8-mL test vial containing a Teflon®-coated magnetic stir bar. The vial was evacuated and backfilled with N2 (repeated for 4 times), followed by addition of alkene (0.1 mmol, 1.0 equiv.) and TMSN$_3$ (0.2 mmol, 2.0 equiv.) in DCM (0.1 M in regard to alkenes) via syringe under N2. The reaction mixture was placed under 427nm Kessil® light (25%) with proper sealing (see General Procedure C) and allowed to react at room temperature for 24 h. Following this, the reaction mixture was filtered through a pad of celite and rinsed with DCM. The concentrated filtrate was then purified through preparatory thin-layer chromatography (with eluent of Hex: EA = 10:1) to give the corresponding chloroazidation products as colorless oil.
Yield 72%, 16.2 mg, r.r. = 4:1 (determined by ^{1}H NMR).
^{1}H NMR (600 MHz, CDCl$_3$) δ 8.12 – 8.08 (m, 2H), 7.66 – 7.61 (m, 1H), 7.51-7.47 (m, 2H), 6.73 (dd, J = 7.1, 3.8 Hz, 0.77H), 6.26 (t, J = 5.3 Hz, 0.20H), 3.87 (dd, J = 13.4, 7.1 Hz, 0.84H), 3.71 (dd, J = 13.4, 3.8 Hz, 0.84H), 3.54 (t, J = 5.6 Hz, 0.42H).
^{13}C NMR (151 MHz, CDCl$_3$) δ 165.71, 163.91, 134.26, 134.09, 130.20, 130.09, 128.74, 128.70, 128.40, 128.16, 83.94, 81.33, 55.64, 52.84.
HRMS APCI: [M-N2+H]$^+$ calcd. for C9H9ClN3O2: 198.0316; Found 196.0314

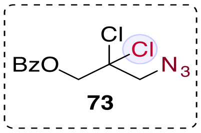

Prepared according to General Procedure C (substrate 0.1 mmol scale): Fe(OAc)$_2$ (10 mol%, 0.1 equiv.), terpyridine (10 mol%, 0.1 equiv.), NCS (0.15 mmol, 1.5 equiv.) were added in an oven-dried 8-mL test vial containing a Teflon®-coated magnetic stir bar. The vial was evacuated and backfilled with N2 (repeated for 4 times), followed by addition of alkene (0.1 mmol, 1.0 equiv.) and TMSN$_3$ (0.2 mmol, 2.0 equiv.) in DCM (0.1 M in regard to alkenes) via syringe under N2. The reaction mixture was placed under 427nm Kessil® light (25%) with proper sealing (see General Procedure C) and allowed to react at room temperature for 24 h. Following this, the reaction mixture was filtered through a pad of celite and rinsed with DCM. The concentrated filtrate was then purified through preparatory thin-layer chromatography (with eluent of Hex: EA = 10:1) to give the corresponding chloroazidation products as colorless oil.
Yield 43%, 11.8 mg.
^{1}H NMR (600 MHz, CDCl$_3$) δ 8.10 – 8.02 (m, 2H), 7.65 – 7.59 (m, 1H), 7.52 – 7.44 (m, 2H), 4.79 (s, 2H), 3.92 (s, 2H).
^{13}C NMR (151 MHz, CDCl$_3$) δ 165.03, 133.74, 129.87, 128.88, 128.64, 86.46, 68.24, 60.19.
HRMS APCI: [M-N2+H]$^+$ calcd. for C10H10Cl2NO2: 246.0083; Found 246.0081

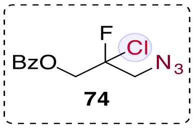

Prepared according to General Procedure C (substrate 0.1 mmol scale): Fe(OAc)$_2$ (10 mol%, 0.1 equiv.), terpyridine (10 mol%, 0.1 equiv.), NCS (0.15 mmol, 1.5 equiv.) were added in an oven-dried 8-mL test vial

containing a Teflon®-coated magnetic stir bar. The vial was evacuated and backfilled with N2 (repeated for 4 times), followed by addition of alkene (0.1 mmol, 1.0 equiv.) and $TMSN_3$ (0.2 mmol, 2.0 equiv.) in DCM (0.1 M in regard to alkenes) via syringe under N2. The reaction mixture was placed under 427nm Kessil® light (25%) with proper sealing (see General Procedure C) and allowed to react at room temperature for 48 h. Following this, the reaction mixture was filtered through a pad of celite and rinsed with DCM. The concentrated filtrate was then purified through preparatory thin-layer chromatography (with eluent of Hex: EA = 10:1) to give the corresponding chloroazidation products as colorless oil.

Yield 38%, 9.8 mg.

^{1}H NMR (600 MHz, $CDCl_3$) δ 8.07-8.03 (m, 2H), 7.64 – 7.58 (m, 1H), 7.50-7.45 (m, 2H), 4.81 – 4.69 (m, 2H), 3.92-3.85 (m, 1H), 3.83-3.75 (m, 1H).

^{13}C NMR (151 MHz, $CDCl_3$) δ 165.15, 133.76, 129.88, 128.81, 128.64, 110.04 (d, J = 248.1 Hz), 65.47 (d, J = 28.2 Hz), 56.33 (d, J = 26.7 Hz).

^{19}F NMR (564 MHz, $CDCl_3$) δ -111.25 – -126.15 (m).

HRMS APCI: $[M-N2+H]^+$ calcd. for C10H10ClFNO2: 230.0379; Found 230.0375

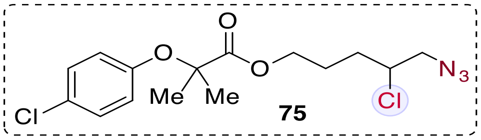

75

Prepared according to General Procedure C (substrate 0.1 mmol scale): $Fe(OAc)_2$ (10 mol%, 0.1 equiv.), terpyridine (10 mol%, 0.1 equiv.), NCS (0.15 mmol, 1.5 equiv.) were added in an oven-dried 8-mL test vial containing a Teflon®-coated magnetic stir bar. The vial was evacuated and backfilled with N2 (repeated for 4 times), followed by addition of alkene (0.1 mmol, 1.0 equiv.) and $TMSN_3$ (0.2 mmol, 2.0 equiv.) in DCM (0.1 M in regard to alkenes) via syringe under N2. The reaction mixture was placed under 427nm Kessil® light (25%) with proper sealing (see General Procedure C) and allowed to react at room temperature for 24 h. Following this, the reaction mixture was filtered through a pad of celite and rinsed with DCM. The concentrated filtrate was then purified through preparatory thin-layer chromatography (with eluent of Hex: EA = 10:1) to give the corresponding chloroazidation products as colorless oil.

Yield 77%, 27.7 mg.

^{1}H NMR (600 MHz, Chloroform-*d*) δ 7.20 (d, J = 8.9 Hz, 2H), 6.76 (d, J = 8.9 Hz, 2H), 4.21 – 4.15 (m, 2H), 3.93 – 3.81 (m, 1H), 3.47-3.33 (m, 2H), 1.92 – 1.86 (m, 1H), 1.74 – 1.65 (m, 2H), 1.60-1.58 (m, 7H).

^{13}C NMR (151 MHz, $CDCl_3$) δ 173.96, 154.14, 129.20, 127.08, 119.98, 79.42, 64.60, 60.21, 56.97, 31.75, 25.40, 25.34, 25.31.

HRMS ESI: $[M+NH4]^+$ calcd. for C15H23Cl2N4O3: 377.1142; Found 377.1138

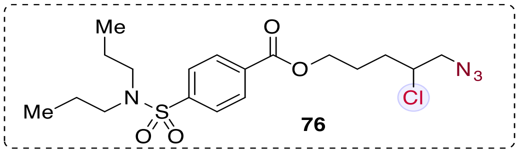

76

Prepared according to General Procedure C (substrate 0.1 mmol scale): $Fe(OAc)_2$ (10 mol%, 0.1 equiv.), terpyridine (10 mol%, 0.1 equiv.), NCS (0.15 mmol, 1.5 equiv.) were added in an oven-dried 8-mL test vial containing a Teflon®-coated magnetic stir bar. The vial was evacuated and backfilled with N2 (repeated for 4 times), followed by addition of alkene (0.1 mmol, 1.0 equiv.) and $TMSN_3$ (0.2 mmol, 2.0 equiv.) in DCM (0.1 M in regard to alkenes) via syringe under N2. The reaction mixture was placed under 427nm Kessil® light (25%) with proper sealing (see General Procedure C) and allowed to react at room temperature for 24 h. Following this, the reaction mixture was filtered through a pad of celite and rinsed with DCM. The concentrated filtrate was then purified through preparatory thin-layer chromatography (with eluent of Hex: EA = 7:1) to give the corresponding chloroazidation products as colorless oil.

Yield 60%, 25.8 mg.

^{1}H NMR (600 MHz, $CDCl_3$) δ 8.14 (d, J = 8.4 Hz, 2H), 7.87 (d, J = 8.5 Hz, 2H), 4.44 – 4.34 (m, 2H), 4.07-4.02 (m, 1H), 3.59-3.50 (m, 2H), 3.11 – 3.08 (m, 4H), 2.12 – 2.04 (m, 1H), 2.03 – 1.96 (m, 1H), 1.96 – 1.82 (m, 2H), 1.57-1.51 (m, 4H), 0.88-0.84 (m, 6H).

^{13}C NMR (151 MHz, $CDCl_3$) δ 165.19, 144.39, 133.36, 130.21, 127.06, 64.69, 60.21, 57.00, 49.93, 32.01, 25.54, 21.94, 11.16.

HRMS ESI: $[M+H]^+$ calcd. for C18H28ClN4O4S: 431.1514; Found 431.1507

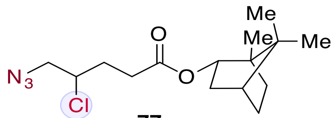
77

Prepared according to General Procedure C (substrate 0.1 mmol scale): $Fe(OAc)_2$ (10 mol%, 0.1 equiv.), terpyridine (10 mol%, 0.1 equiv.), NCS (0.15 mmol, 1.5 equiv.) were added in an oven-dried 8-mL test vial containing a Teflon®-coated magnetic stir bar. The vial was evacuated and backfilled with N2 (repeated for 4 times), followed by addition of alkene (0.1 mmol, 1.0 equiv.) and $TMSN_3$ (0.2 mmol, 2.0 equiv.) in DCM (0.1 M in regard to alkenes) via syringe under N2. The reaction mixture was placed under 427nm Kessil® light (25%) with proper sealing (see General Procedure C) and allowed to react at room temperature for 24 h. Following this, the reaction mixture was filtered through a pad of celite and rinsed with DCM. The concentrated filtrate was then purified through column chromatography (with eluent of Hex: EA = 20:1) to give the corresponding chloroazidation products as colorless oil.

Yield 54%, 16.9 mg.

^{1}H NMR (600 MHz, $CDCl_3$) δ 4.92-4.87 (m, 1H), 4.11-4.03 (m, 1H), 3.57-3.50 (m, 2H), 2.63-2.49 (m, 2H), 2.39-2.31 (m, 1H), 2.23-2.15 (m, 1H), 2.01 – 1.94 (m, 1H), 1.91 (m, 1H), 1.80-1.71 (m, 1H), 1.70-1.66 (m, 1H), 1.35-1.27 (m, 1H), 1.25-1.20 (m, 1H), 0.90 (s, 3H), 0.87 (s, 3H), 0.83 (s, 3H).

^{13}C NMR (151 MHz, $CDCl_3$) δ 172.73, 80.36, 80.34, 60.02, 60.00, 57.07, 48.79, 47.83, 44.87, 36.81, 36.77, 31.03, 30.59, 30.56, 28.04, 27.11, 19.70, 18.83, 13.55, 13.54.

HRMS ESI: $[M+H]^+$ calcd. for C15H25ClN3O2: 314.1630; Found 314.1624

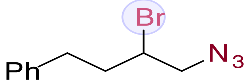
78

Prepared according to General Procedure D (substrate 0.1 mmol scale): $Fe(acac)_2$ (10 mol%, 0.1 equiv.), terpyridine (10 mol%, 0.1 equiv.), NBS (0.15 mmol, 1.5 equiv.) were added in an oven-dried 8-mL test vial containing a Teflon®-coated magnetic stir bar. The vial was evacuated and backfilled with N2 (repeated for 4 times), followed by addition of alkene (0.1 mmol, 1.0 equiv.) and $TMSN_3$ (0.2 mmol, 2.0 equiv.) in DCE (0.1 M in regard to alkenes) via syringe under N2. The reaction mixture was placed under 427nm Kessil® light (50%) with proper sealing (see General Procedure D) and allowed to react at room temperature for 24 h. Following this, the reaction mixture was filtered through a pad of celite and rinsed with DCM. The concentrated filtrate was then purified through preparatory thin-layer chromatography (with eluent of Hexane) to give the corresponding bromoazidation products as colorless oil.

Yield 50% (^{1}H NMR yield by using CH_2Br_2 asinternal standard).

^{1}H NMR (600 MHz, $CDCl_3$) δ 7.33 – 7.29 (m, 2H), 7.25-7.19 (m, 3H), 4.02-3.94 (m, 1H), 3.67 (dd, J = 13.0, 6.2 Hz, 1H), 3.61 (dd, J = 13.0, 5.9 Hz, 1H), 2.94-2.88 (m, 1H), 2.79-2.72 (m, 1H), 2.21 – 2.08 (m, 2H).

^{13}C NMR (151 MHz, $CDCl_3$) δ 140.19, 128.61, 128.51, 126.36, 57.53, 52.31, 37.45, 33.24.

HRMS APCI: $[M-N2+H]^+$ calcd. for C10H13BrN: 226.0226; Found 226.0222

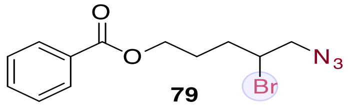
79

Prepared according to General Procedure D (substrate 0.1 mmol scale): $Fe(acac)_2$ (10 mol%, 0.1 equiv.), terpyridine (10 mol%, 0.1 equiv.), NBS (0.15 mmol, 1.5 equiv.) were added in an oven-dried 8-mL test vial containing a Teflon®-coated magnetic stir bar. The vial was evacuated and backfilled with N2 (repeated for 4 times), followed by addition of alkene (0.1 mmol, 1.0 equiv.) and $TMSN_3$ (0.2 mmol, 2.0 equiv.) in DCE (0.1 M in regard to alkenes) via syringe under N2. The reaction mixture was placed under 427nm Kessil® light (50%) with proper sealing (see General Procedure D) and allowed to react at room temperature for 24 h. Following this, the reaction mixture was filtered through a pad of celite and rinsed with DCM. The concentrated filtrate was then purified through preparatory thin-layer chromatography (with eluent of Hex: EA = 10:1) to give the corresponding bromoazidation products as colorless oil.

Yield 51%, 15.9 mg.

^{1}H NMR (600 MHz, CDCl$_3$) δ 8.08 – 8.00 (m, 2H), 7.60 – 7.53 (m, 1H), 7.45 (t, *J* = 7.7 Hz, 2H), 4.41-4.33 (m, 2H), 4.14-4.07 (m, 1H), 3.70 (dd, *J* = 13.0, 6.1 Hz, 1H), 3.63 (dd, *J* = 13.0, 6.0 Hz, 1H), 2.13 – 2.03 (m, 2H), 1.99 – 1.88 (m, 2H).
^{13}C NMR (151 MHz, CDCl$_3$) δ 166.54, 133.06, 130.11, 129.58, 128.44, 63.91, 57.44, 52.29, 32.61, 26.71.
HRMS ESI: [M+H]$^+$ calcd. for C12H15BrN3O2: 312.0342; Found 312.0340

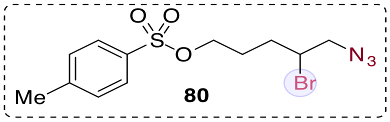

Prepared according to General Procedure D (substrate 0.1 mmol scale): Fe(acac)$_2$ (10 mol%, 0.1 equiv.), terpyridine (10 mol%, 0.1 equiv.), NBS (0.15 mmol, 1.5 equiv.) were added in an oven-dried 8-mL test vial containing a Teflon®-coated magnetic stir bar. The vial was evacuated and backfilled with N2 (repeated for 4 times), followed by addition of alkene (0.1 mmol, 1.0 equiv.) and TMSN$_3$ (0.2 mmol, 2.0 equiv.) in DCE (0.1 M in regard to alkenes) via syringe under N2. The reaction mixture was placed under 427nm Kessil® light (50%) with proper sealing (see General Procedure D) and allowed to react at room temperature for 24 h. Following this, the reaction mixture was filtered through a pad of celite and rinsed with DCM. The concentrated filtrate was then purified through preparatory thin-layer chromatography (with eluent of Hex: EA = 10:1) to give the corresponding bromoazidation products as colorless oil.
Yield 63%, 22.8 mg.
^{1}H NMR (600 MHz, CDCl$_3$) δ 7.79 (d, *J* = 8.3 Hz, 2H), 7.36 (d, *J* = 8.0 Hz, 2H), 4.11 – 4.03 (m, 2H), 3.98-3.92 (m, 1H), 3.63 (dd, *J* = 13.0, 6.1 Hz, 1H), 3.56 (dd, *J* = 13.0, 5.9 Hz, 1H), 2.46 (s, 3H), 2.00 – 1.90 (m, 2H), 1.84 – 1.73 (m, 2H).
^{13}C NMR (151 MHz, CDCl$_3$) δ 145.00, 132.89, 129.95, 127.91, 69.30, 57.40, 51.88, 31.84, 26.88, 21.67.
HRMS ESI: [M+H]$^+$ calcd. for C12H17BrN3O2S: 362.0169; Found 362.0162

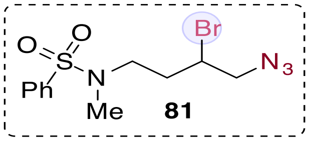

Prepared according to General Procedure D (substrate 0.1 mmol scale): Fe(acac)$_2$ (10 mol%, 0.1 equiv.), terpyridine (10 mol%, 0.1 equiv.), NBS (0.15 mmol, 1.5 equiv.) were added in an oven-dried 8-mL test vial containing a Teflon®-coated magnetic stir bar. The vial was evacuated and backfilled with N2 (repeated for 4 times), followed by addition of alkene (0.1 mmol, 1.0 equiv.) and TMSN$_3$ (0.2 mmol, 2.0 equiv.) in DCE (0.1 M in regard to alkenes) via syringe under N2. The reaction mixture was placed under 427nm Kessil® light (50%) with proper sealing (see General Procedure D) and allowed to react at room temperature for 24 h. Following this, the reaction mixture was filtered through a pad of celite and rinsed with DCM. The concentrated filtrate was then purified through preparatory thin-layer chromatography (with eluent of Hex: EA = 10:1) to give the corresponding bromoazidation products as colorless oil.
Yield 49%, 17.0 mg.
^{1}H NMR (600 MHz, CDCl$_3$) δ 7.82-7.77 (m, 2H), 7.63 – 7.59 (m, 1H), 7.58 – 7.52 (m, 2H), 4.21 – 4.15 (m, 1H), 3.75 (dd, *J* = 13.2, 5.7 Hz, 1H), 3.68 (dd, *J* = 13.2, 5.5 Hz, 1H), 3.22 – 3.12 (m, 2H), 2.77 (s, 3H), 2.25-2.17 (m, 1H), 2.05 – 1.97 (m, 1H).
^{13}C NMR (151 MHz, CDCl$_3$) δ 136.97, 132.87, 129.22, 127.43, 57.16, 49.36, 48.38, 35.77, 34.43.
HRMS ESI: [M+H]$^+$ calcd. for C14H16BrN4O2S: 347.0172; Found 347.0167

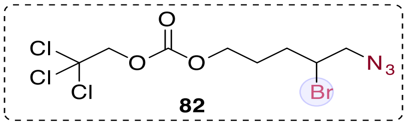

Prepared according to General Procedure D (substrate 0.1 mmol scale): Fe(acac)$_2$ (10 mol%, 0.1 equiv.), terpyridine (10 mol%, 0.1 equiv.), NBS (0.15 mmol, 1.5 equiv.) were added in an oven-dried 8-mL test vial containing a Teflon®-coated magnetic stir bar. The vial was evacuated and backfilled with N2 (repeated for 4 times), followed by addition of alkene (0.1 mmol, 1.0 equiv.) and TMSN$_3$ (0.2 mmol, 2.0 equiv.) in DCE (0.1 M in regard to alkenes) via syringe under N2. The reaction mixture was placed under 427nm Kessil® light (50%) with proper sealing (see General Procedure D) and allowed to react at room temperature for 24 h.

Following this, the reaction mixture was filtered through a pad of celite and rinsed with DCM. The concentrated filtrate was then purified through column chromatography (with eluent of Hex: EA = 10:1) to give the corresponding bromoazidation products as colorless oil.
Yield 41%, 15.7 mg.
^{1}H NMR (600 MHz, CDCl$_3$) δ 4.78 (s, 2H), 4.31-4.25 (m, 2H), 4.07-4.02 (m, 1H), 3.69 (dd, *J* = 13.0, 6.1 Hz, 1H), 3.62 (dd, *J* = 13.0, 6.1 Hz, 1H), 2.08 – 1.98 (m, 2H), 1.93 – 1.82 (m, 2H).
^{13}C NMR (151 MHz, CDCl$_3$) δ 153.96, 94.39, 76.81, 68.11, 57.42, 52.00, 32.12, 26.60.
HRMS APCI: [M-N2+H]$^+$ calcd. for C8H12BrCl3NO3: 353.9061; Found 353.9054

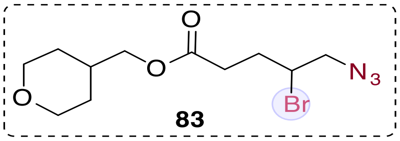

Prepared according to General Procedure D (substrate 0.1 mmol scale): Fe(acac)$_2$ (10 mol%, 0.1 equiv.), terpyridine (10 mol%, 0.1 equiv.), NBS (0.15 mmol, 1.5 equiv.) were added in an oven-dried 8-mL test vial containing a Teflon®-coated magnetic stir bar. The vial was evacuated and backfilled with N2 (repeated for 4 times), followed by addition of alkene (0.1 mmol, 1.0 equiv.) and TMSN$_3$ (0.2 mmol, 2.0 equiv.) in DCE (0.1 M in regard to alkenes) via syringe under N2. The reaction mixture was placed under 427nm Kessil® light (50%) with proper sealing (see General Procedure D) and allowed to react at room temperature for 24 h. Following this, the reaction mixture was filtered through a pad of celite and rinsed with DCM. The concentrated filtrate was then purified through column chromatography (with eluent of Hex: EA = 10:1) to give the corresponding bromoazidation products as colorless oil.
Yield 40%, 12.8 mg.
^{1}H NMR (600 MHz, CDCl$_3$) δ 4.14-4.09 (m, 1H), 4.01-3.92 (m, 4H), 3.69 (dd, *J* = 13.1, 6.1 Hz, 1H), 3.62 (dd, *J* = 13.2, 6.2 Hz, 1H), 3.42-3.36 (m, 2H), 2.64-2.57 (m, 1H), 2.56-2.50 (m, 1H), 2.30-2.23 (m, 1H), 2.07-2.00 (m, 1H), 1.94-1.87 (m, 1H), 1.64 – 1.61 (m, 2H), 1.42-1.33 (m, 2H).
^{13}C NMR (151 MHz, CDCl$_3$) δ 172.35, 68.96, 67.45, 57.42, 51.83, 34.50, 31.77, 30.95, 29.50.
HRMS ESI: [M+H]$^+$ calcd. for C11H19BrN3O3: 320.0604; Found 320.0601

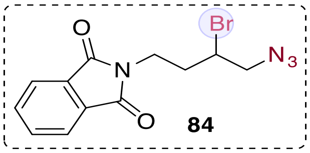

Prepared according to General Procedure D (substrate 0.1 mmol scale): Fe(acac)$_2$ (10 mol%, 0.1 equiv.), terpyridine (10 mol%, 0.1 equiv.), alkene (0.1 mmol, 1.0 equiv.), NBS (0.15 mmol, 1.5 equiv.) were added in an oven-dried 8-mL test vial containing a Teflon®-coated magnetic stir bar. The vial was evacuated and backfilled with N2 (repeated for 4 times), followed by addition of TMSN$_3$ (0.2 mmol, 2.0 equiv.) in DCE (0.1 M in regard to alkenes) via syringe under N2. The reaction mixture was placed under 427nm Kessil® light (50%) with proper sealing (see General Procedure D) and allowed to react at room temperature for 24 h. Following this, the reaction mixture was filtered through a pad of celite and rinsed with DCM. The concentrated filtrate was then purified through preparatory thin-layer chromatography (with eluent of Hex: EA = 5:1) to give the corresponding bromoazidation products as colorless oil.
Yield 62%, 20.0 mg.
^{1}H NMR (600 MHz, CDCl$_3$) δ 7.85 (dd, *J* = 5.4, 3.1 Hz, 2H), 7.73 (dd, *J* = 5.5, 3.0 Hz, 2H), 4.08 – 4.01 (m, 1H), 3.94-3.88 (m, 1H), 3.87-3.82 (m, 1H), 3.73 (dd, *J* = 13.1, 6.0 Hz, 1H), 3.66 (dd, *J* = 13.1, 5.7 Hz, 1H), 2.37 – 2.29 (m, 1H), 2.23-2.13 (m, 1H).
^{13}C NMR (151 MHz, CDCl$_3$) δ 168.21, 134.16, 131.96, 123.42, 57.21, 49.13, 36.09, 34.65.
HRMS ESI: [M+H]$^+$ calcd. for C12H12BrN4O2: 323.0138; Found 323.0135

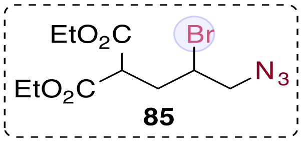

Prepared according to General Procedure D (substrate 0.1 mmol scale): Fe(acac)$_2$ (10 mol%, 0.1 equiv.), terpyridine (10 mol%, 0.1 equiv.), NBS (0.15 mmol, 1.5 equiv.) were added in an oven-dried 8-

mL test vial containing a Teflon®-coated magnetic stir bar. The vial was evacuated and backfilled with N2 (repeated for 4 times), followed by addition of alkene (0.1 mmol, 1.0 equiv.) and $TMSN_3$ (0.2 mmol, 2.0 equiv.) in DCE (0.1 M in regard to alkenes) via syringe under N2. The reaction mixture was placed under 427nm Kessil® light (50%) with proper sealing (see General Procedure D) and allowed to react at room temperature for 24 h. Following this, the reaction mixture was filtered through a pad of celite and rinsed with DCM. The concentrated filtrate was then purified through column chromatography (with eluent of Hex: EA = 10:1) to give the corresponding bromoazidation products as colorless oil.
Yield 46%, 14.8 mg.
^{1}H NMR (600 MHz, $CDCl_3$) δ 4.28 – 4.17 (m, 4H), 4.13-4.06 (m, 1H), 3.76 – 3.62 (m, 3H), 2.56-2.47 (m, 1H), 2.31-2.21 (m, 1H), 1.30-1.24 (m, 6H).
^{13}C NMR (151 MHz, $CDCl_3$) δ 168.59, 168.43, 61.91, 61.86, 57.47, 50.09, 50.04, 34.89, 14.05, 14.02.
HRMS ESI: $[M+H]^+$ calcd. for C10H17BrN3O4: 322.0397; Found 322.0393

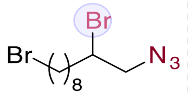

86

Prepared according to General Procedure D (substrate 0.1 mmol scale): $Fe(acac)_2$ (10 mol%, 0.1 equiv.), terpyridine (10 mol%, 0.1 equiv.), NBS (0.15 mmol, 1.5 equiv.) were added in an oven-dried 8-mL test vial containing a Teflon®-coated magnetic stir bar. The vial was evacuated and backfilled with N2 (repeated for 4 times), followed by addition of alkene (0.1 mmol, 1.0 equiv.) and $TMSN_3$ (0.2 mmol, 2.0 equiv.) in DCE (0.1 M in regard to alkenes) via syringe under N2. The reaction mixture was placed under 427nm Kessil® light (50%) with proper sealing (see General Procedure D) and allowed to react at room temperature for 24 h. Following this, the reaction mixture was filtered through a pad of celite and rinsed with DCM. The concentrated filtrate was then purified through column chromatography (with eluent of Hexane) to give the corresponding bromoazidation products as colorless oil.
Yield 41%, 14.0 mg.
^{1}H NMR (600 MHz, $CDCl_3$) δ 4.07 – 4.01 (m, 1H), 3.65 (dd, J = 13.0, 6.3 Hz, 1H), 3.60 (dd, J = 13.0, 5.8 Hz, 1H), 3.41 (t, J = 6.8 Hz, 2H), 1.91 – 1.77 (m, 4H), 1.55-1.51 (m, 1H), 1.46-1.40 (m, 3H), 1.36-1.27 (m, 6H).
^{13}C NMR (151 MHz, $CDCl_3$) δ 57.50, 53.24, 35.88, 33.99, 32.76, 29.17, 28.78, 28.62, 28.09, 27.15.
HRMS APCI: $[M-N2+H]^+$ calcd. for C10H20Br2N: 311.9957; Found 311.9954

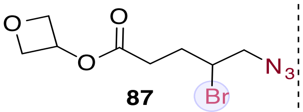

87

Prepared according to General Procedure D (substrate 0.1 mmol scale): $Fe(acac)_2$ (10 mol%, 0.1 equiv.), terpyridine (10 mol%, 0.1 equiv.), NBS (0.15 mmol, 1.5 equiv.) were added in an oven-dried 8-mL test vial containing a Teflon®-coated magnetic stir bar. The vial was evacuated and backfilled with N2 (repeated for 4 times), followed by addition of alkene (0.1 mmol, 1.0 equiv.) and $TMSN_3$ (0.2 mmol, 2.0 equiv.) in DCE (0.1 M in regard to alkenes) via syringe under N2. The reaction mixture was placed under 427nm Kessil® light (50%) with proper sealing (see General Procedure D) and allowed to react at room temperature for 24 h. Following this, the reaction mixture was filtered through a pad of celite and rinsed with DCM. The concentrated filtrate was then purified through preparatory thin-layer chromatography (with eluent of Hex: EA = 10:1) to give the corresponding bromoazidation products as colorless oil.
Yield 39%, 10.8 mg.
^{1}H NMR (600 MHz, $CDCl_3$) δ 5.45 (p, J = 5.8 Hz, 1H), 4.93-4.83 (m, 2H), 4.69 – 4.59 (m, 2H), 4.14-4.06 (m, 1H), 3.70 (dd, J = 13.0, 6.0 Hz, 1H), 3.62 (dd, J = 13.1, 6.1 Hz, 1H), 2.72-2.62 (m, 1H), 2.61-2.54 (m, 1H), 2.30-2.24 (m, 1H), 2.10 – 2.01 (m, 1H).
^{13}C NMR (151 MHz, $CDCl_3$) δ 171.65, 77.46, 68.15, 57.37, 51.51, 31.56, 30.71.
HRMS ESI: $[M+H]^+$ calcd. for C8H13BrN3O3: 278.0135; Found 278.0131

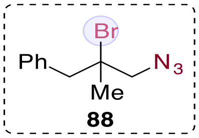

Prepared according to General Procedure D (substrate 0.1 mmol scale): $Fe(acac)_2$ (10 mol%, 0.1 equiv.), terpyridine (10 mol%, 0.1 equiv.), NBS (0.15 mmol, 1.5 equiv.) were added in an oven-dried 8-mL test vial containing a Teflon®-coated magnetic stir bar. The vial was evacuated and backfilled with N2 (repeated for 4 times), followed by addition of alkene (0.1 mmol, 1.0 equiv.) and $TMSN_3$ (0.2 mmol, 2.0 equiv.) in DCE (0.1 M in regard to alkenes) via syringe under N2. The reaction mixture was placed under 427nm Kessil® light (50%) with proper sealing (see General Procedure D) and allowed to react at room temperature for 24 h. Following this, the reaction mixture was filtered through a pad of celite and rinsed with DCM. The concentrated filtrate was then purified through preparatory thin-layer chromatography (with eluent of Hexane) to give the corresponding bromoazidation products as colorless oil.

Yield 43%, 10.9 mg.

^{1}H NMR (600 MHz, $CDCl_3$) δ 7.35 – 7.26 (m, 5H), 3.57 – 3.49 (m, 2H), 3.27 (d, *J* = 14.0 Hz, 1H), 3.20 (d, *J* = 14.0 Hz, 1H), 1.75 (s, 3H).

^{13}C NMR (151 MHz, $CDCl_3$) δ 135.90, 130.79, 128.22, 127.25, 67.25, 61.57, 48.02, 29.12.

HRMS APCI: $[M-N2+H]^+$ calcd. for C10H13BrN: 226.0226; Found 226.0221

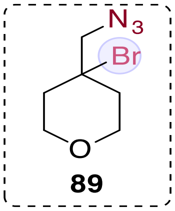

Prepared according to General Procedure D (substrate 0.1 mmol scale): $Fe(acac)_2$ (10 mol%, 0.1 equiv.), terpyridine (10 mol%, 0.1 equiv.), NBS (0.15 mmol, 1.5 equiv.) were added in an oven-dried 8-mL test vial containing a Teflon®-coated magnetic stir bar. The vial was evacuated and backfilled with N2 (repeated for 4 times), followed by addition of alkene (0.1 mmol, 1.0 equiv.) and $TMSN_3$ (0.2 mmol, 2.0 equiv.) in DCE (0.1 M in regard to alkenes) via syringe under N2. The reaction mixture was placed under 427nm Kessil® light (50%) with proper sealing (see General Procedure D) and allowed to react at room temperature for 24 h. Following this, the reaction mixture was filtered through a pad of celite and rinsed with DCM. The concentrated filtrate was then purified through preparatory thin-layer chromatography (with eluent of Hex: EA = 20:1) to give the corresponding bromoazidation products as colorless oil.

Yield 47%, 10.3 mg.

^{1}H NMR (600 MHz, $CDCl_3$) δ 3.94-3.87 (m, 2H), 3.86-3.81 (t, *J* = 11.3 Hz, 2H), 3.67 (s, 2H), 1.96-1.90 (m, 2H), 1.88-1.83 (m, 2H).

^{13}C NMR (151 MHz, $CDCl_3$) δ 68.17, 64.04, 63.81, 37.56.

HRMS APCI: $[M-N2+H]^+$ calcd. for C6H11BrNO: 192.0019; Found 192.0016

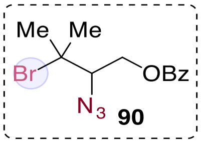

Prepared according to General Procedure D (substrate 0.1 mmol scale): $Fe(acac)_2$ (10 mol%, 0.1 equiv.), terpyridine (10 mol%, 0.1 equiv.), NBS (0.15 mmol, 1.5 equiv.) were added in an oven-dried 8-mL test vial containing a Teflon®-coated magnetic stir bar. The vial was evacuated and backfilled with N2 (repeated for 4 times), followed by addition of alkene (0.1 mmol, 1.0 equiv.) and $TMSN_3$ (0.2 mmol, 2.0 equiv.) in DCE (0.1 M in regard to alkenes) via syringe under N2. The reaction mixture was placed under 427nm Kessil® light (50%) with proper sealing (see General Procedure D) and allowed to react at room temperature for 24 h. Following this, the reaction mixture was filtered through a pad of celite and rinsed with DCM. The concentrated filtrate was then purified through preparatory thin-layer chromatography (with eluent of Hex: EA = 10:1) to give the corresponding bromoazidation products as colorless oil.

Yield 50%, 15.6 mg.

¹H NMR (600 MHz, $CDCl_3$) δ 8.11 – 8.04 (m, 2H), 7.62 – 7.56 (m, 1H), 7.47 (t, *J* = 7.8 Hz, 2H), 4.97 (dd, *J* = 11.5, 2.8 Hz, 1H), 4.41 (dd, *J* = 11.4, 9.3 Hz, 1H), 3.88 (dd, *J* = 9.3, 2.8 Hz, 1H), 1.89 (s, 3H), 1.82 (s, 3H).
¹³C NMR (151 MHz, $CDCl_3$) δ 166.21, 133.45, 129.82, 129.35, 128.57, 70.66, 65.90, 63.05, 31.92, 30.62.
HRMS ESI: $[M+H]^+$ calcd. for C12H15BrN3O2: 312.0342; Found 312.0338

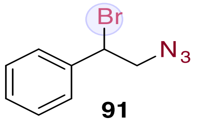

91

Prepared according to General Procedure D (substrate 0.1 mmol scale): Fe(acac)$_2$ (10 mol%, 0.1 equiv.), terpyridine (10 mol%, 0.1 equiv.), NBS (0.15 mmol, 1.5 equiv.) were added in an oven-dried 8-mL test vial containing a Teflon®-coated magnetic stir bar. The vial was evacuated and backfilled with N2 (repeated for 4 times), followed by addition of alkene (0.1 mmol, 1.0 equiv.) and $TMSN_3$ (0.2 mmol, 2.0 equiv.) in DCE (0.1 M in regard to alkenes) via syringe under N2. The reaction mixture was placed under 427nm Kessil® light (50%) with proper sealing (see General Procedure D) and allowed to react at room temperature for 24 h. Following this, the reaction mixture was filtered through a pad of celite and rinsed with DCM. The concentrated filtrate was then purified through preparatory thin-layer chromatography (with eluent of Hexane) to give the corresponding bromoazidation products as colorless oil.
Yield 83%, 18.7 mg.
¹H NMR (600 MHz, $CDCl_3$) δ 7.47 – 7.31 (m, 5H), 5.01 (t, *J* = 7.3 Hz, 1H), 3.94-3.80 (m, 2H).
¹³C NMR (151 MHz, $CDCl_3$) δ 138.53, 129.25, 129.03, 127.69, 57.62, 51.12.
HRMS APCI: $[M-N2+H]^+$ calcd. for C8H9BrN: 197.9913; Found 197.9910

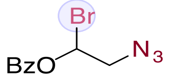

92

Prepared according to General Procedure D (substrate 0.1 mmol scale): Fe(acac)$_2$ (10 mol%, 0.1 equiv.), terpyridine (10 mol%, 0.1 equiv.), NBS (0.15 mmol, 1.5 equiv.) were added in an oven-dried 8-mL test vial containing a Teflon®-coated magnetic stir bar. The vial was evacuated and backfilled with N2 (repeated for 4 times), followed by addition of alkene (0.1 mmol, 1.0 equiv.) and $TMSN_3$ (0.2 mmol, 2.0 equiv.) in DCE (0.1 M in regard to alkenes) via syringe under N2. The reaction mixture was placed under 427nm Kessil® light (50%) with proper sealing (see General Procedure D) and allowed to react at room temperature for 24 h. Following this, the reaction mixture was filtered through a pad of celite and rinsed with DCM. The concentrated filtrate was then purified through preparatory thin-layer chromatography (with eluent of Hex: EA = 10:1) to give the corresponding bromoazidation products as colorless oil.
Yield 69%, 18.6 mg.
¹H NMR (600 MHz, $CDCl_3$) δ 8.09 (d, *J* = 7.7 Hz, 2H), 7.69 – 7.60 (m, 1H), 7.49 (t, *J* = 7.2 Hz, 2H), 6.92 – 6.86 (m, 1H), 4.01 (dd, *J* = 13.6, 7.7 Hz, 1H), 3.77 (dt, *J* = 13.5, 2.5 Hz, 1H).
¹³C NMR (151 MHz, $CDCl_3$) δ ¹³C NMR (151 MHz, $CDCl_3$) δ 163.78, 134.31, 130.22, 128.76, 128.13, 72.27, 56.39.
HRMS APCI: $[M-N2+H]^+$ calcd. for C9H9BrNO2: 241.9811; Found 241.9808

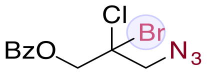

93

Prepared according to General Procedure D (substrate 0.1 mmol scale): Fe(acac)$_2$ (10 mol%, 0.1 equiv.), terpyridine (10 mol%, 0.1 equiv.), NBS (0.15 mmol, 1.5 equiv.) were added in an oven-dried 8-mL test vial containing a Teflon®-coated magnetic stir bar. The vial was evacuated and backfilled with N2 (repeated for 4 times), followed by addition of alkene (0.1 mmol, 1.0 equiv.) and $TMSN_3$ (0.2 mmol, 2.0 equiv.) in DCE (0.1 M in regard to alkenes) via syringe under N2. The reaction mixture was placed under 427nm Kessil® light (50%) with proper sealing (see General Procedure D) and allowed to react at room temperature for 24 h. Following this, the reaction mixture was filtered through a pad of celite and rinsed with DCM. The concentrated filtrate was then

purified through preparatory thin-layer chromatography (with eluent of Hex: EA = 10:1) to give the corresponding bromoazidation products as colorless oil.
Yield 62%, 19.8 mg.
^{1}H NMR (600 MHz, CDCl$_3$) δ 8.11 – 8.03 (m, 2H), 7.65 – 7.58 (m, 1H), 7.54 – 7.44 (m, 2H), 4.92-4.76 (m, 2H), 4.05 – 3.95 (m, 2H).
^{13}C NMR (151 MHz, CDCl$_3$) δ 164.95, 133.73, 129.88, 128.91, 128.65, 75.92, 68.99, 61.08.
HRMS ESI: $[M+H]^+$ calcd. for C10H10BrClN3O2: 317.9639; Found 317.9637

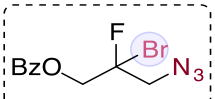

94

Prepared according to General Procedure D (substrate 0.1 mmol scale): Fe(acac)$_2$ (10 mol%, 0.1 equiv.), terpyridine (10 mol%, 0.1 equiv.), NBS (0.15 mmol, 1.5 equiv.) were added in an oven-dried 8-mL test vial containing a Teflon®-coated magnetic stir bar. The vial was evacuated and backfilled with N2 (repeated for 4 times), followed by addition of alkene (0.1 mmol, 1.0 equiv.) and TMSN$_3$ (0.2 mmol, 2.0 equiv.) in DCE (0.1 M in regard to alkenes) via syringe under N2. The reaction mixture was placed under 427nm Kessil® light (50%) with proper sealing (see General Procedure D) and allowed to react at room temperature for 24 h. Following this, the reaction mixture was filtered through a pad of celite and rinsed with DCM. The concentrated filtrate was then purified through preparatory thin-layer chromatography (with eluent of Hex: EA = 10:1) to give the corresponding bromoazidation products as colorless oil.
Yield 68%, 20.5 mg.
^{1}H NMR (600 MHz, CDCl$_3$) δ 8.11 – 8.03 (m, 2H), 7.65 – 7.58 (m, 1H), 7.54 – 7.44 (m, 2H), 4.92-4.76 (m, 2H), 4.05 – 3.95 (m, 2H).
^{13}C NMR (151 MHz, CDCl$_3$) δ 165.09, 133.76, 129.89, 128.82, 128.64, 104.74 (d, J = 258.1 Hz), 66.47 (d, J = 26.6 Hz), 57.50 (d, J = 25.2 Hz).
^{19}F NMR (564 MHz, CDCl$_3$) δ -118.51 (tt, J = 17.3, 12.5 Hz).
HRMS ESI: $[M+Na]^+$ calcd. for C10H9BrFN3NaO2: 323.9754; Found 323.9747

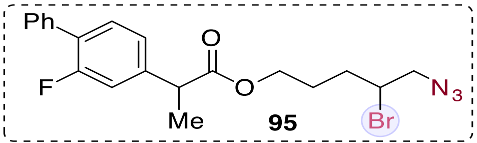

95

Prepared according to General Procedure D (substrate 0.1 mmol scale): Fe(acac)$_2$ (10 mol%, 0.1 equiv.), terpyridine (10 mol%, 0.1 equiv.), NBS (0.15 mmol, 1.5 equiv.) were added in an oven-dried 8-mL test vial containing a Teflon®-coated magnetic stir bar. The vial was evacuated and backfilled with N2 (repeated for 4 times), followed by addition of alkene (0.1 mmol, 1.0 equiv.) and TMSN$_3$ (0.2 mmol, 2.0 equiv.) in DCE (0.1 M in regard to alkenes) via syringe under N2. The reaction mixture was placed under 427nm Kessil® light (50%) with proper sealing (see General Procedure D) and allowed to react at room temperature for 24 h. Following this, the reaction mixture was filtered through a pad of celite and rinsed with DCM. The concentrated filtrate was then purified through preparatory thin-layer chromatography (with eluent of Hex: EA = 10:1) to give the corresponding bromoazidation products as colorless oil.
Yield 34%, 14.7 mg.
^{1}H NMR (600 MHz, CDCl$_3$) δ 7.57-7.51 (m, 2H), 7.46-7.42 (m, 2H), 7.42 – 7.35 (m, 2H), 7.18 – 7.10 (m, 2H), 4.20 – 4.09 (m, 2H), 4.01-3.93 (m, 1H), 3.76 (q, J = 7.2 Hz, 1H), 3.63-3.57 (m, 1H), 3.52 (dd, J = 13.0, 6.1 Hz, 1H), 1.95 – 1.81 (m, 2H), 1.79-1.70 (m, 2H), 1.54 (d, J = 7.2 Hz, 3H).
^{13}C NMR (151 MHz, CDCl$_3$) δ 173.89 (d, J = 2.0 Hz), 159.70 (d, J = 248.4 Hz), 141.74 (d, J = 7.6 Hz), 135.41, 130.86 (d, J = 3.9 Hz), 128.94 (d, J = 2.9 Hz), 128.50, 127.91 (d, J = 13.6 Hz), 127.73, 123.55 (d, J = 3.3 Hz), 115.25 (d, J = 23.6 Hz), 63.86 (d, J = 12.2 Hz), 57.36, 52.13 (d, J = 2.6 Hz), 45.04, 32.36 (d, J = 11.0 Hz), 26.47 (d, J = 1.7 Hz), 18.20 (d, J = 2.7 Hz).
^{19}F NMR (564 MHz, CDCl$_3$) δ -113.13 – -125.43 (m).
HRMS ESI: $[M+NH4]^+$ calcd. for C20H25BrFN4O2: 451.1139; Found 451.1134

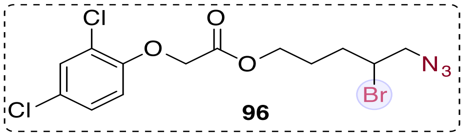

Prepared according to General Procedure D (substrate 0.1 mmol scale): Fe(acac)$_2$ (10 mol%, 0.1 equiv.), terpyridine (10 mol%, 0.1 equiv.), NBS (0.15 mmol, 1.5 equiv.) were added in an oven-dried 8-mL test vial containing a Teflon®-coated magnetic stir bar. The vial was evacuated and backfilled with N2 (repeated for 4 times), followed by addition of alkene (0.1 mmol, 1.0 equiv.) and TMSN$_3$ (0.2 mmol, 2.0 equiv.) in DCE (0.1 M in regard to alkenes) via syringe under N2. The reaction mixture was placed under 427nm Kessil® light (50%) with proper sealing (see General Procedure D) and allowed to react at room temperature for 24 h. Following this, the reaction mixture was filtered through a pad of celite and rinsed with DCM. The concentrated filtrate was then purified through preparatory thin-layer chromatography (with eluent of Hex: EA = 10:1) to give the corresponding bromoazidation products as colorless oil.
Yield 41%, 16.8 mg.
^{1}H NMR (600 MHz, CDCl$_3$) δ 7.40 (d, J = 2.5 Hz, 1H), 7.17 (dd, J = 8.8, 2.5 Hz, 1H), 6.78 (d, J = 8.8 Hz, 1H), 4.70 (s, 2H), 4.28 – 4.20 (m, 2H), 4.05 – 3.95 (m, 1H), 3.65 (dd, J = 13.0, 6.0 Hz, 1H), 3.57 (dd, J = 13.0, 6.2 Hz, 1H), 1.98 – 1.87 (m, 2H), 1.82 – 1.74 (m, 2H).
^{13}C NMR (151 MHz, CDCl$_3$) δ 168.09, 152.35, 130.44, 127.61, 127.19, 124.27, 114.62, 66.35, 64.52, 57.37, 51.98, 32.30, 26.48.
HRMS ESI: $[M+Na]^+$ calcd. for C13H14BrCl2N3NaO3: 431.9488; Found 431.9485

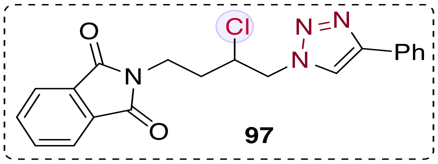

Prepared according to Procedure of late-stage application (substrate 0.1 mmol scale) The reaction mixture was purified through preparatory thin-layer chromatography (with eluent of Hex: EA = 2:1) to give the corresponding triazole products as white solid, **melting point** 178 ºC.
Yield 42%, 16.0 mg.
^{1}H NMR (600 MHz, CDCl$_3$) δ 7.95 (s, 1H), 7.86-7.80 (m, 4H), 7.75-7.69 (m, 2H), 7.44-.39 (m, 2H), 7.36-7.30 (m, 1H), 4.79-4.71 (m, 2H), 4.40-4.32 (m, 1H), 3.97 (dt, J = 13.7, 6.7 Hz, 1H), 3.91 (dt, J = 14.0, 6.9 Hz, 1H), 2.31-2.22 (m, 1H), 2.09-2.01 (m, 1H).
^{13}C NMR (151 MHz, CDCl$_3$) δ 168.22, 134.21, 131.93, 130.34, 128.87, 128.29, 125.81, 123.45, 121.08, 57.45, 55.76, 34.95, 34.10.
HRMS ESI: $[M+H]^+$ calcd. for C20H18ClN4O2: 381.1113; Found 381.1108

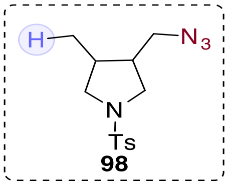

Prepared according to General Procedure A (substrate 0.1 mmol scale): Fe(NO$_3$)$_3$·9H$_2$O (10 mol%, 0.1 equiv.) and terpyridine (10 mol%, 0.1 equiv.) were added in an oven-dried 8-mL test vial containing a Teflon®-coated magnetic stir bar. The vial was evacuated and backfilled with N2 (repeated for 4 times), followed by addition of alkene (0.1 mmol, 1.0 equiv.) TMSN$_3$ (0.40 mmol, 4.0 equiv.), 4-F-thiolphenol (10 mol%, 0.1 equiv.) in HCCl$_3$/H$_2$O (19:1, 0.1 M in regard to alkenes) via syringe under N2. The reaction mixture was placed under 427nm Kessil® light (25%) with proper sealing (see General Procedure A) and allowed to react at room temperature for 36 h. Following this, the reaction mixture was filtered through a pad of celite and rinsed with DCM. The concentrated filtrate was then purified through preparatory thin-layer chromatography (with eluent of Hex: EA = 5:1) to give the corresponding hydroazidation products as colorless oil.
Yield 45%, 13.2mg, **d.r.** = 2:1.
^{1}H NMR (600 MHz, CDCl$_3$) δ 7.74-7.68 (m, 2H), 7.36-7.31 (m, 2H), 3.52 – 3.38 (m, 2H), 3.33-3.23 (m, 1H), 3.16 – 3.09 (m, 1H), 3.07-3.02 (m, 0.46H), 2.99 – 2.93 (m, 1H), 2.82-2.75 (m, 0.46H), 2.45-2.40 (m, 3H), 2.35 – 2.19 (m, 1.40H), 1.91 – 1.80 (m, 0.70H), 0.96 (d, J = 6.5 Hz, 1H), 0.83 (d, J = 7.1 Hz, 2H).

^{13}C NMR (151 MHz, $CDCl_3$) δ 143.62, 143.59, 133.72, 133.41, 129.73, 129.72, 127.59, 127.46, 54.58, 54.22, 52.50, 51.03, 49.94, 49.91, 45.34, 41.31, 36.18, 34.52, 21.55, 16.75, 12.95.
HRMS ESI: $[M+H]^+$ calcd. for C13H15N4O2S: 295.1223; Found 295.1218

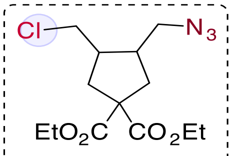

99

Prepared according to General Procedure C (substrate 0.1 mmol scale): $Fe(OAc)_2$ (10 mol%, 0.1 equiv.), terpyridine (10 mol%, 0.1 equiv.), NCS (0.15 mmol, 1.5 equiv.) were added in an oven-dried 8-mL test vial containing a Teflon®-coated magnetic stir bar. The vial was evacuated and backfilled with N2 (repeated for 4 times), followed by addition of alkene (0.1 mmol, 1.0 equiv.) and $TMSN_3$ (0.2 mmol, 2.0 equiv.) in DCM (0.1 M in regard to alkenes) via syringe under N2. The reaction mixture was placed under 427nm Kessil® light (25%) with proper sealing (see General Procedure C) and allowed to react at room temperature for 24 h. Following this, the reaction mixture was filtered through a pad of celite and rinsed with DCM. The concentrated filtrate was then purified through column chromatography (with eluent of Hex: EA = 10:1) to give the corresponding chloroazidation products as colorless oil.
Yield 45%, 22.8mg, **d.r.** = 1:1.
^{1}H NMR (600 MHz, $CDCl_3$) δ 4.19 (q, J = 7.1 Hz, 4H), 3.58-3.52 (m, 1H), 3.52-3.46 (m, 1H), 3.44-3.38 (m, 1H), 3.36-3.29 (m, 1H), 2.59-2.36 (m, 4H), 2.23-2.12 (m, 2H), 1.24 (t, J = 7.1 Hz, 6H).
^{13}C NMR (151 MHz, $CDCl_3$) δ 172.04, 171.98, 61.80, 61.78, 58.64, 51.22, 44.41, 43.54, 40.56, 37.43, 37.07, 14.01.
HRMS ESI: $[M+H]^+$ calcd. for C13H21ClN3O4: 318.1215; Found 318.1212

2.4 Additional substrates for photocatalytic hydroazidation

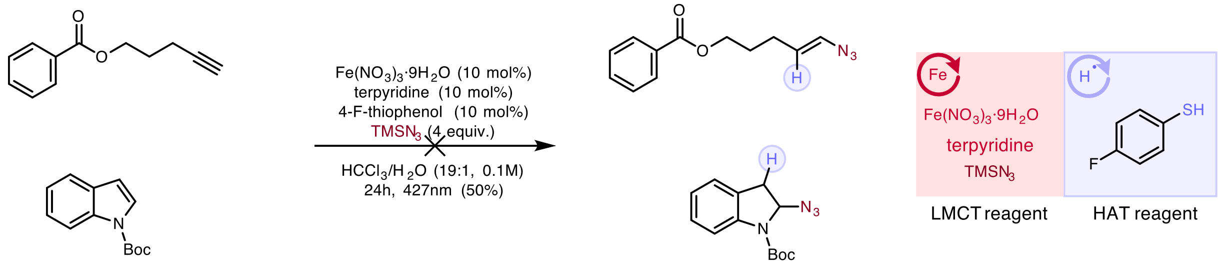

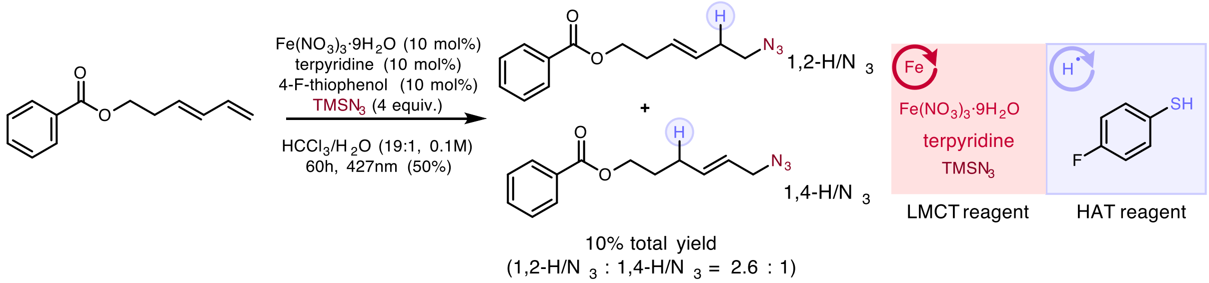

Following standard conditions, we have also carried out our photocatalytic hydroazidation on other class of substrates. First, alkyne and protected indole are not sutiable for the cooperative systems, giving almost full recovery of starting materials.

Interestingly, conjugated alkenes could afford corresponding product with 1,2- and 1,4-hydroazidation in a ratio of 2.6 : 1, and in relatively lower yields. Nevertheless, as this class of substrate was not compatible with previous systems, it demonstrates the synthetic advantage of this cooperative system and we expect by adjusting the ligand framework and other variables of reaction conditions, a higher yield and ratio could be obtained.

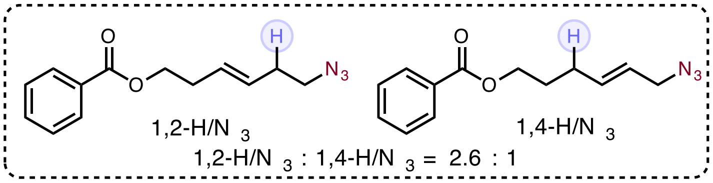

^{1}H NMR (600 MHz, $CDCl_3$) δ 8.06 – 8.01 (m, 2H), 7.59 – 7.54 (m, 1H), 7.48 – 7.40 (m, 2H), 5.84 – 5.72 (m, 0.37H), 5.67 – 5.53 (m, 1.45H), 5.34 – 5.27 (m, 0.18H), 4.40 – 4.30 (m, 2H), 4.02 – 3.67 (m, 0.60H), 3.33 – 3.18 (m, 1.26H), 2.58 – 2.25 (m, 3H), 1.93 – 1.68 (m, 1H).
^{13}C NMR (151 MHz, $CDCl_3$) δ 166.55, 135.34, 132.92, 130.35, 129.56, 128.83, 128.79, 128.38, 128.36, 127.90, 123.95, 64.13, 52.70, 50.94, 32.23, 32.12, 28.76, 28.17.
HRMS ESI: $[M+Na]^+$ calcd. for C13H15N3O2Na: 268.1056; Found 268.1058

Supplementary Figure 1. 1H NMR of Diene Hydroazidation

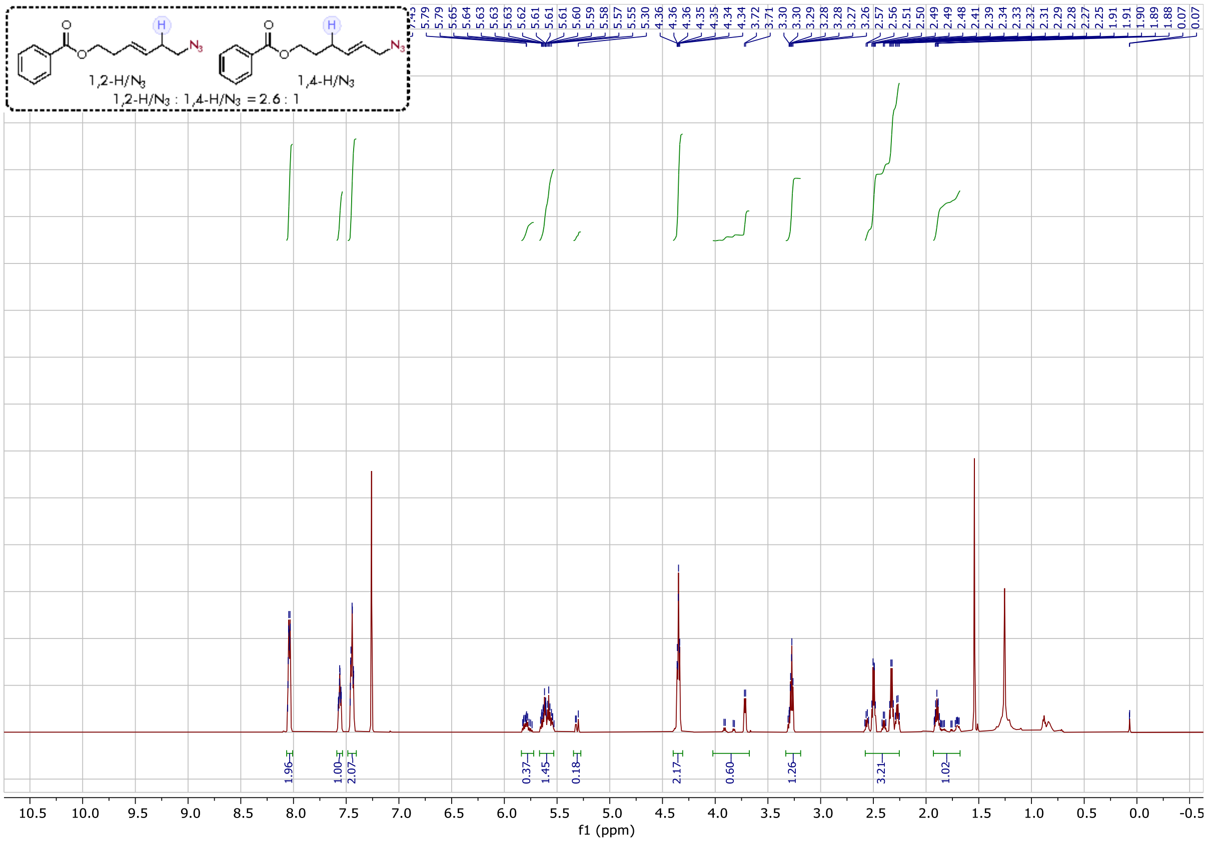

Supplementary Figure 2. 13C NMR of Diene Hydroazidation

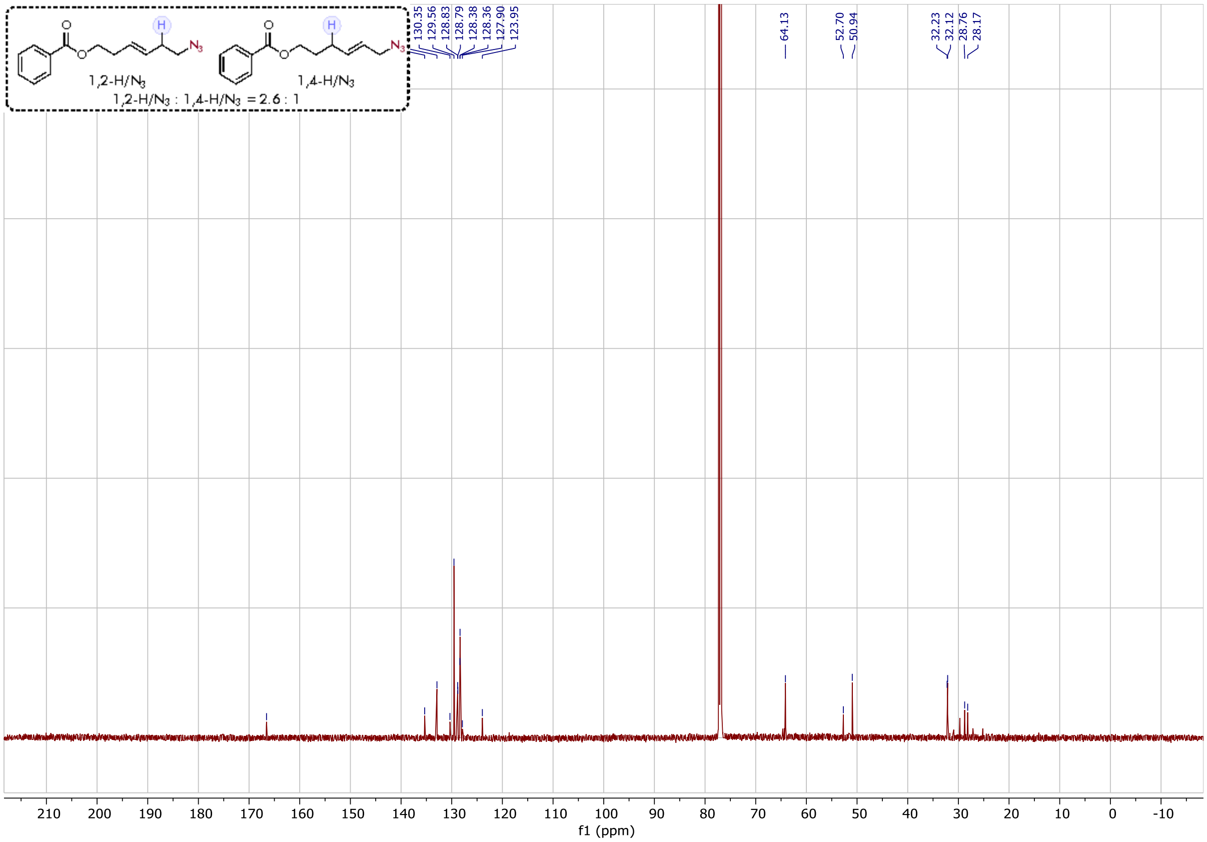

2.5 Scale up reaction and late-stage application

Supplementary Figure 3. Scale Up of Hydroazidation Reaction

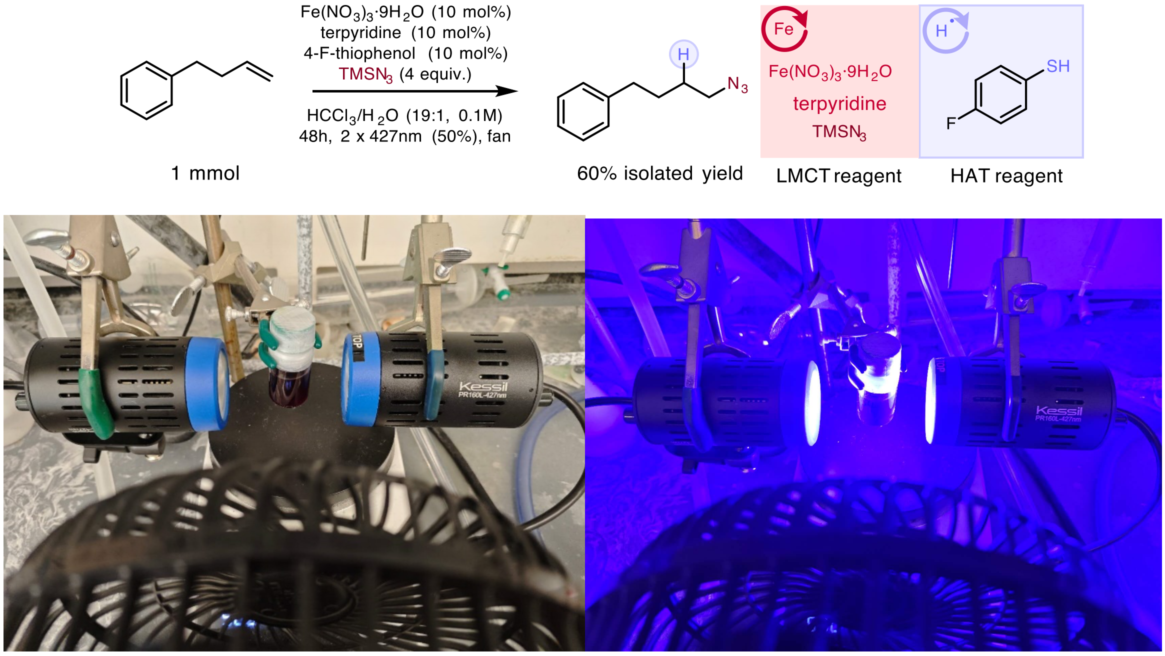

$Fe(NO_3)_3 \cdot 9H_2O$ (10 mol%, 0.1 equiv.) and terpyridine (10 mol%, 0.1 equiv.) were added in an oven-dried 20-mL test vial containing a Teflon®-coated magnetic stir bar. The vial was evacuated and backfilled with N2 (repeated for 4 times), followed by addition of alkenes (1.0 mmol, 1.0 equiv.), $TMSN_3$ (4.0 mmol, 4.0 equiv.), 4-F-thiolphenol (10 mol%, 0.1 equiv.) in $HCCl_3$/H2O (19:1, 0.1 M in regard to alkenes) via syringe under N2. The reaction mixture was placed under 427nm Kessil® light (2 lamps) after sealing the punctured holes of the vial cap with vacuum grease and electric tape/parafilm for better air-tight protection and allowed to react at room temperature for 48 h with fan for cooling. Following this, the reaction mixture was filtered through a pad of celite which was subsequently rinsed with DCM. The filtrate was concentrated, and the residue was then purified by flash column chromatography to give the corresponding hydroazidated products.

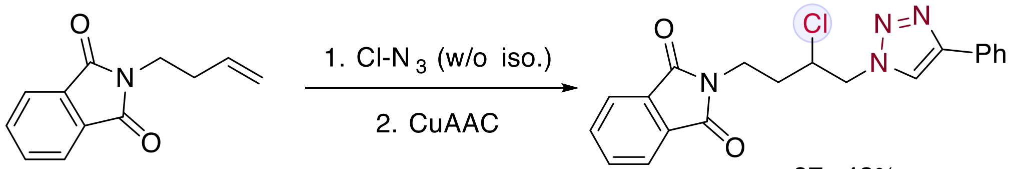

97, 42%

Step 1:

$Fe(OAc)_2$ (10 mol%, 0.1 equiv.), terpyridine (10 mol%, 0.1 equiv.), 2-(but-3-en-1-yl)isoindoline-1,3-dione (0.1 mmol, 1 equiv.) and NCS (0.15 mmol, 1.5 equiv.) were added in an oven-dried 8-mL test vial containing a Teflon®-coated magnetic stir bar. The vial was evacuated and backfilled with N2 (repeated for 4 times), followed by addition of $TMSN_3$ (0.20 mmol, 2.0 equiv.) in DCM (0.1 M in regard to alkenes) via syringe under N2. The reaction mixture was placed under 427nm Kessil® light after sealing the punctured holes of the vial cap with vacuum grease and electric tape/parafilm for better air-tight protection and allowed to react at room temperature for 24 h. Following this, the reaction mixture was filtered through a pad of celite which was subsequently rinsed with DCM. The filtrate was concentrated and subjected to the following Huisgen cyclization without further purification.

Step 2:

An oven-dried 8-mL test vial containing a Teflon®-coated magnetic stir bar was charged with $CuSO_4{\cdot}5H_2O$ (10 mol%, 0.1 equiv.) and sodium ascorbate (0.2 mmol, 2 equiv.). The vial was evacuated and backfilled with N2 (repeated for 4 times), followed by addition of DMF/H_2O mixed solution (10:1). The suspension was stirred at room temperature for 30 minutes followed by addition of triethylamine (0.2 mmol, 2 equiv.), phenylacetylene (0.2 mmol, 2equiv.) and the crude chloroazidation product from Step 1 in the mixed solution of DMF/H_2O (10:1; half of total volume is used for premixing and half for solution of chloroazidation product which in total makes 0.11 M of concentration in regard to chloroazidation product.) via syringe under N2. The Cu-catalyzed alkyne-azide cycloaddition (CuAAC) was stirred for 48 h at room temperature. Following this, the reaction mixture was filtered through a pad of celite which was subsequently rinsed with DCM. The filtrate was concentrated, and the residue was then purified by preparatory thin-layer chromatography to give the corresponding chlorine-substituted triazole product.

2.6 Kinetic Isotope Experiments (KIE)

Supplementary Figure 4. Kinetic Isotope Effect Reaction Experiments

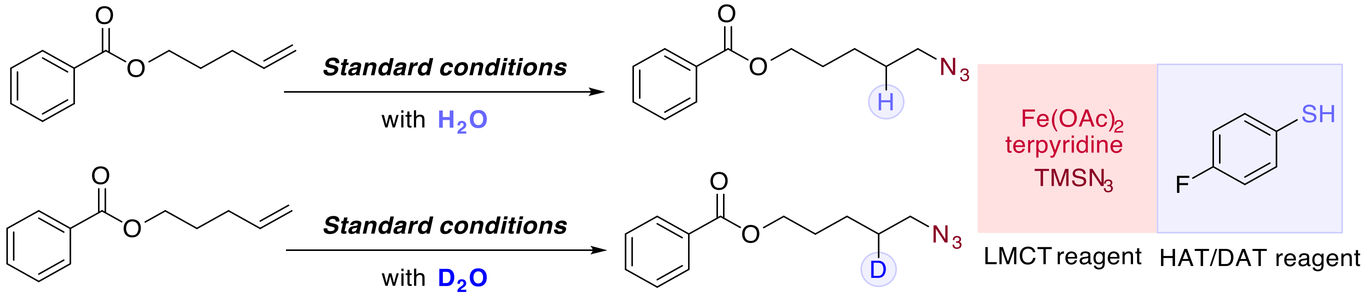

Fe(OAc)$_2$, terpyridine and alkene were added in an oven-dried 8-mL test vial containing a Teflon®-coated magnetic stir bar. The vial was evacuated and backfilled with N_2 (repeated for 4 times), followed by addition of TMSN$_3$ and 4-F-thiolphenol in HCCl$_3$/H_2O (19:1, 0.1 M in regard to alkenes) via syringe under N_2. The procedure of deuteroazidation is identical to hydroazidation with exception of using HCCl$_3$/D2O (19:1, 0.1 M in regard to alkenes) as solvent. Note: All reactions were run on 0.2 mmol of the substrate and CH_2Br_2 was added as internal standard. The reaction mixture was placed under 427nm Kessil® light. At different time points, the aliquot of reaction mixture is directly transferred to NMR tube and reaction progress was monitored by ^{1}H NMR.

Note: Fe(OAc)$_2$ is selected for KIE study to avoid competitive hydrogen atom transfer from hydrate in Fe(NO$_3$)$_3$·9H_2O.

Supplementary Table 15. Kinetic Isotope Effect Experiment Data

Reaction with **H_2O**		Reaction with **D_2O**	
t (s)	H-product/M	t (s)	D-product/M
7200	0.002	7200	0.001
10800	0.004	10800	0.002
14400	0.005	14400	0.002
18000	0.008	18000	0.003
21600	0.01	21600	0.004

25200	0.013	25200	0.005
28800	0.014	28800	0.007
32400	0.017	32400	0.008
36000	0.017	36000	0.009

entry	mmol N3	mmol alkene	mmol iron	mmol thiol	initial rate (M/s)
H_2O KIE	0.4	0.1	0.01	0.01	5.65×10^{-7}
D_2O KIE	0.4	0.1	0.01	0.01	2.87×10^{-7}

Supplementary Figure 5. Rate Measurement of Hydroazidation

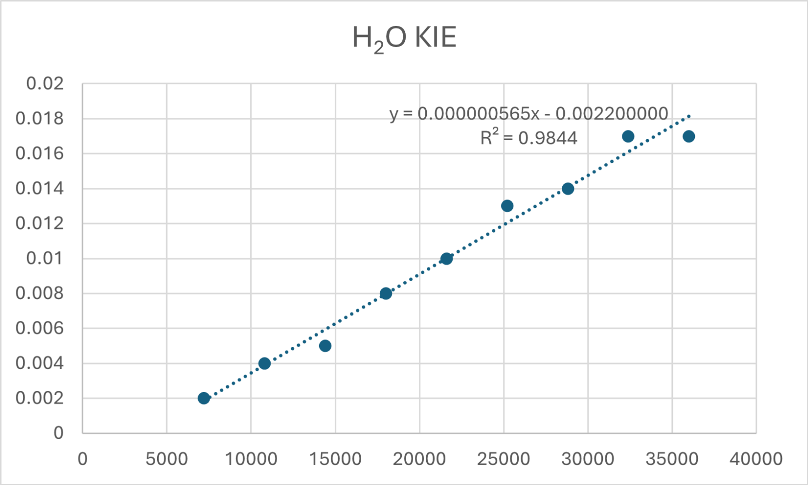

Reaction with H_2O (standard conditions): $y = (5.65 \times 10^{-7})(k_H)x - (2.2 \times 10^{-3})$, $R^2 = 0.9844$

Supplementary Figure 6. Rate Measurement of Deuteroazidation

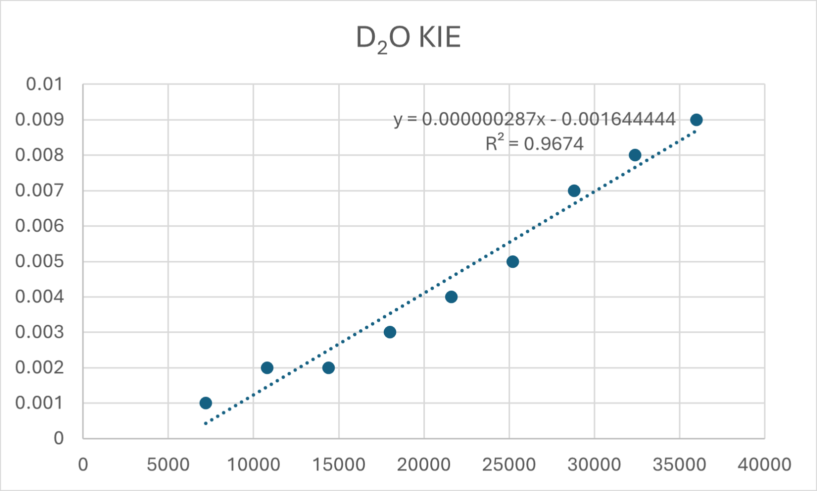

Reaction with D_2O (standard conditions): $y = (2.87 \times 10^{-7})(k_D)x - (1.6 \times 10^{-3})$, $R^2 = 0.9674$

KIE: $k_H/k_D = 2.0$

2.7 Spectroscopic studies of different iron species and time-resolved UV-visible studies

Supplementary Figure 7. UV-Vis Studies of Iron Complexes

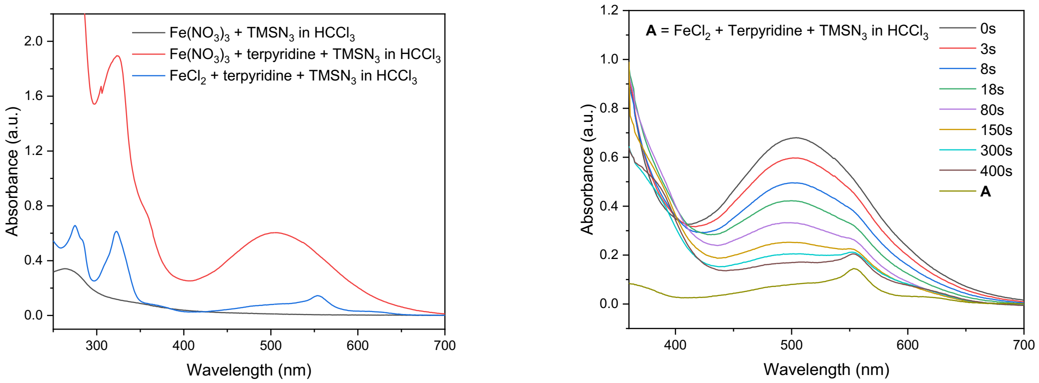

Left:

(a) Black line: $Fe(NO_3)_3 \cdot 9H_2O$ (0.01 mmol) and $TMSN_3$ (0.40 mmol) were added to $HCCl_3$ (1 ml), offering a 10 mM solution, followed by dilution to 1 mM in $HCCl_3$ and transferred to UV-visible cell for measurement.

(b) Blue line: $FeCl_2$ (0.01 mmol), terpyridine (0.01 mmol) and $TMSN_3$ (0.40 mmol) were added to $HCCl_3$ (1 ml), offering a 10 mM solution, followed by dilution to 1 mM in $HCCl_3$ and transferred to UV-visible cell for measurement.

(c) Red line: $Fe(NO_3)_3 \cdot 9H_2O$ (0.01 mmol), terpyridine (0.01 mmol) and $TMSN_3$ (0.40 mmol) were added to $HCCl_3$ (1 ml), offering a 10 mM solution, followed by dilution to 1 mM in $HCCl_3$ and transferred to UV-visible cell for measurement.

Right:

$Fe(NO_3)_3 \cdot 9H_2O$ (0.01 mmol) was added in an oven-dried 8-mL test vial. The vial was evacuated and backfilled with N2, followed by addition of cyclohexane (0.1 mmol) and $TMSN_3$ (0.40 mmol) in $HCCl_3$ (1 ml) via syringe under N2. 20 µl of mixture was transferred to a capped UV-visible cell (containing 2 ml $HCCl_3$) via syringe and degassed for 2 minutes. The UV-visible cell was irradiated by 390nm Kessil® light using irradiation time intervals of 3, 8, 18, 80, 150, 300, 400 s. After each irradiation, the cell was vigorously shaken for 15 sec, ensuring good mixing of the solution, followed by performing UV-visible characterization at respective intervals.

2.8 CV studies of photocatalytic hydroazidation

Supplementary Figure 8. Cyclic Voltammetry Studies of Reaction Components

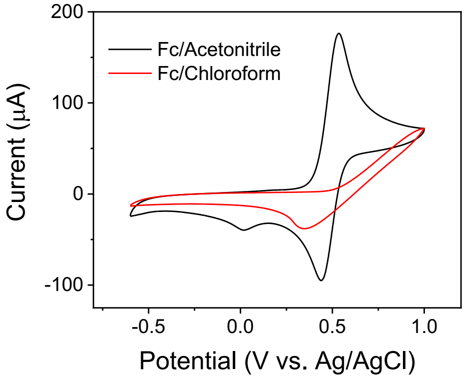

(a) CV Analysis of ferrocene (3 mM in MeCN or $CHCl_3$).

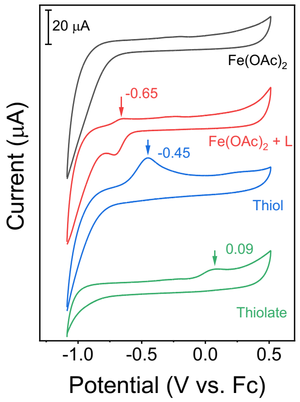

(b) CV Analysis of $Fe(OAc)_2$, $Fe(OAc)_2$ with terpy ligand, 4-fluorothiophenol, Sodium 4-fluorothiophenolate (3 mM in MeCN).

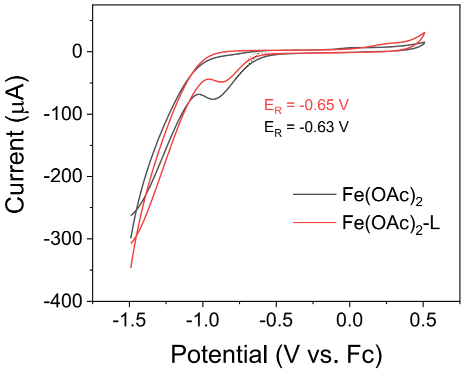

(c) CV Analysis of $Fe(OAc)_2$, $Fe(OAc)_2$ with terpy ligand (3 mM in EtOH).

Cyclic voltammetry (CV) was performed in a three-electrode system using a CHI 680D electrochemical workstation (CHI Instruments, USA). The working electrode was a glassy carbon disk electrode (diameter: 5.0 mm, PTFE shroud). A platinum wire served as the auxiliary electrode, and an Ag/AgCl electrode (saturated KCl solution) was used as the reference electrode. Electrolyte solutions consisted of either 0.1 M tetrabutylammonium tetrafluoroborate (NBu_4BF_4) in organic solvents (acetonitrile, ethanol, and chloroform) or 0.1 M KCl in water. The CV measurements were conducted at a scan rate of 50 mV s^{-1}. Prior to sample analysis, the solvent window was scanned to confirm the absence of electroactive impurities. Afterward, ferrocene (Fc, 3 mM) was introduced as an internal standard to calibrate the redox potential. Before measurements, the glassy

carbon electrode was polished with 0.05 μm alumina slurry and thoroughly rinsed with deionized water and acetone.

III. Supplemental Figures

The NMR spectra consisted of ^{1}H NMR, ^{13}C NMR and regioselectivities of isolated products.

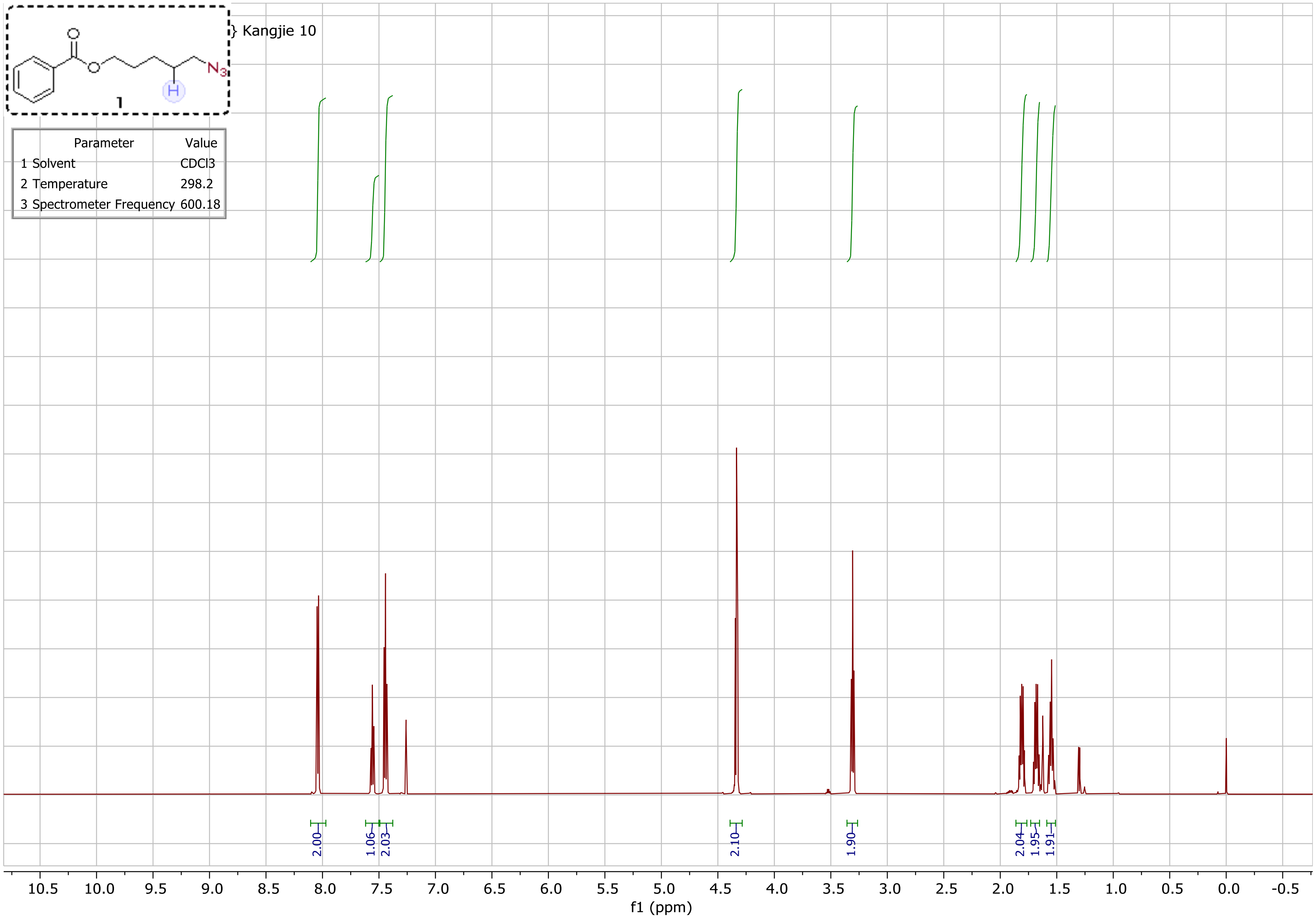

	Parameter	Value
1	Solvent	CDCl3
2	Temperature	298.2
3	Spectrometer Frequency	600.18

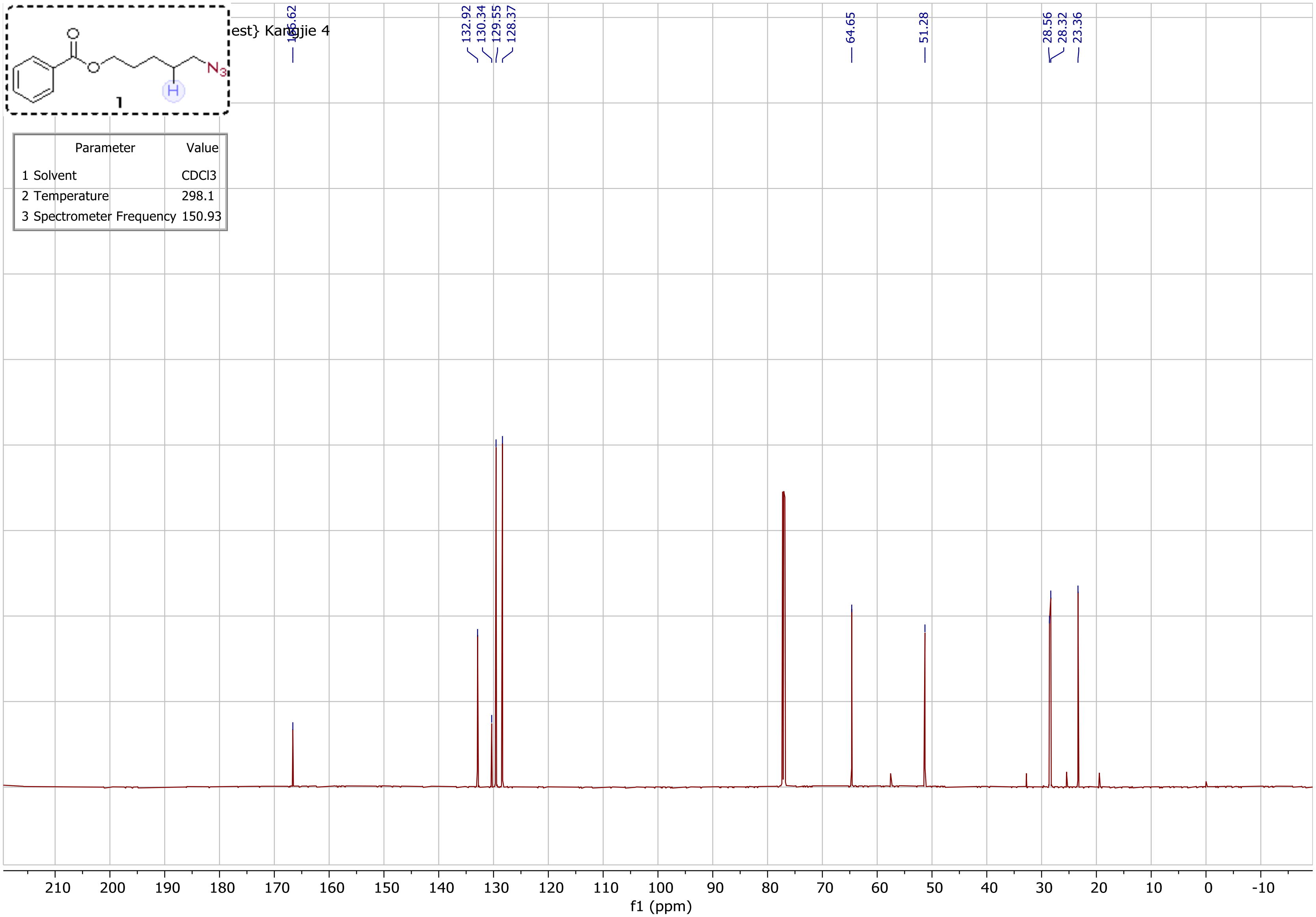

Parameter	Value
1 Solvent	CDCl3
2 Temperature	298.1
3 Spectrometer Frequency	150.93

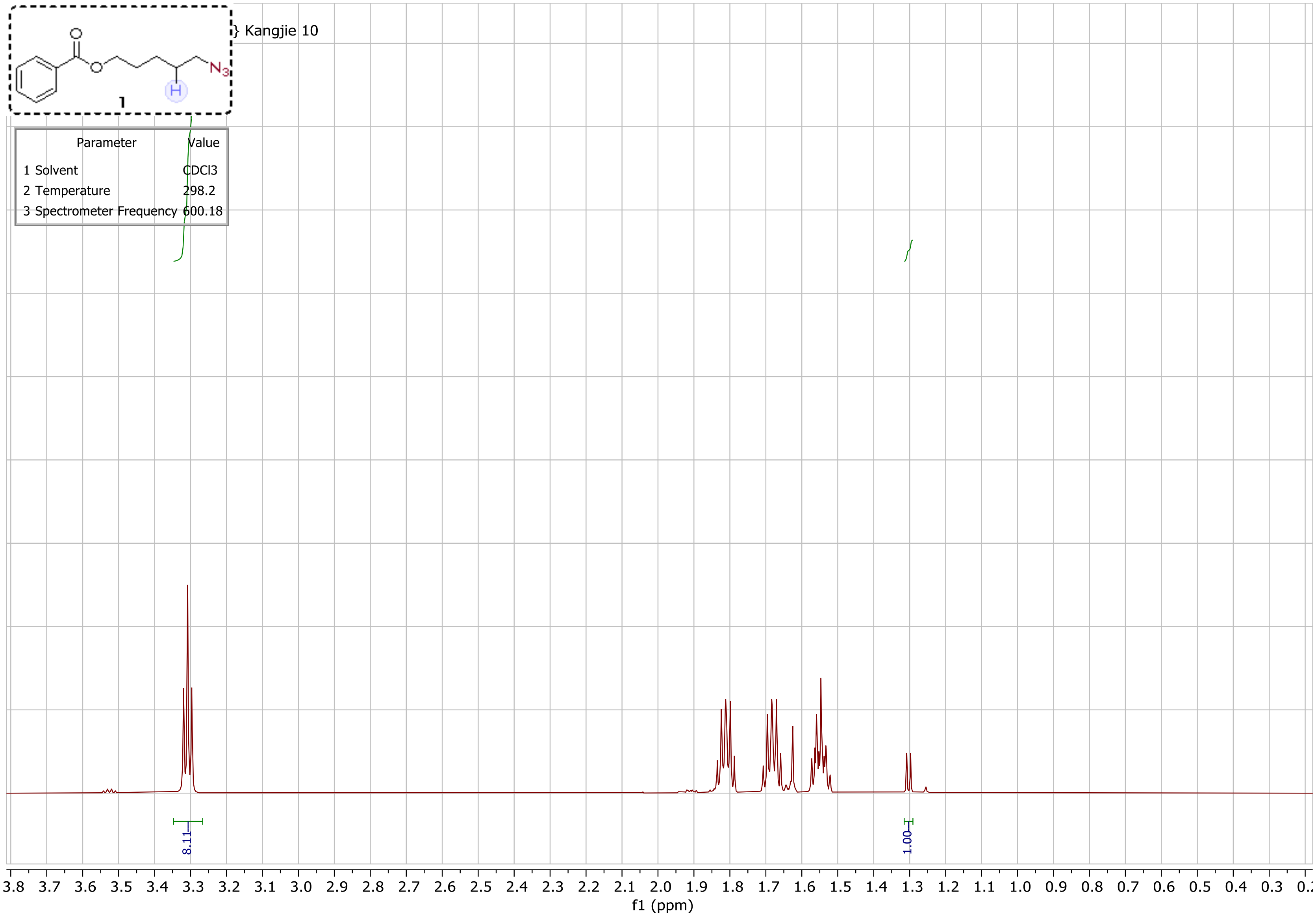

} Kangjie 10
N3
H
1
Parameter
Value
1 Solvent
CDCl3
2 Temperature
298.2
3 Spectrometer Frequency
600.18
8.11
1.00
3.8 3.7 3.6 3.5 3.4 3.3 3.2 3.1 3.0 2.9 2.8 2.7 2.6 2.5 2.4 2.3 2.2 2.1 2.0 1.9 1.8 1.7 1.6 1.5 1.4 1.3 1.2 1.1 1.0 0.9 0.8 0.7 0.6 0.5 0.4 0.3
f1 (ppm)

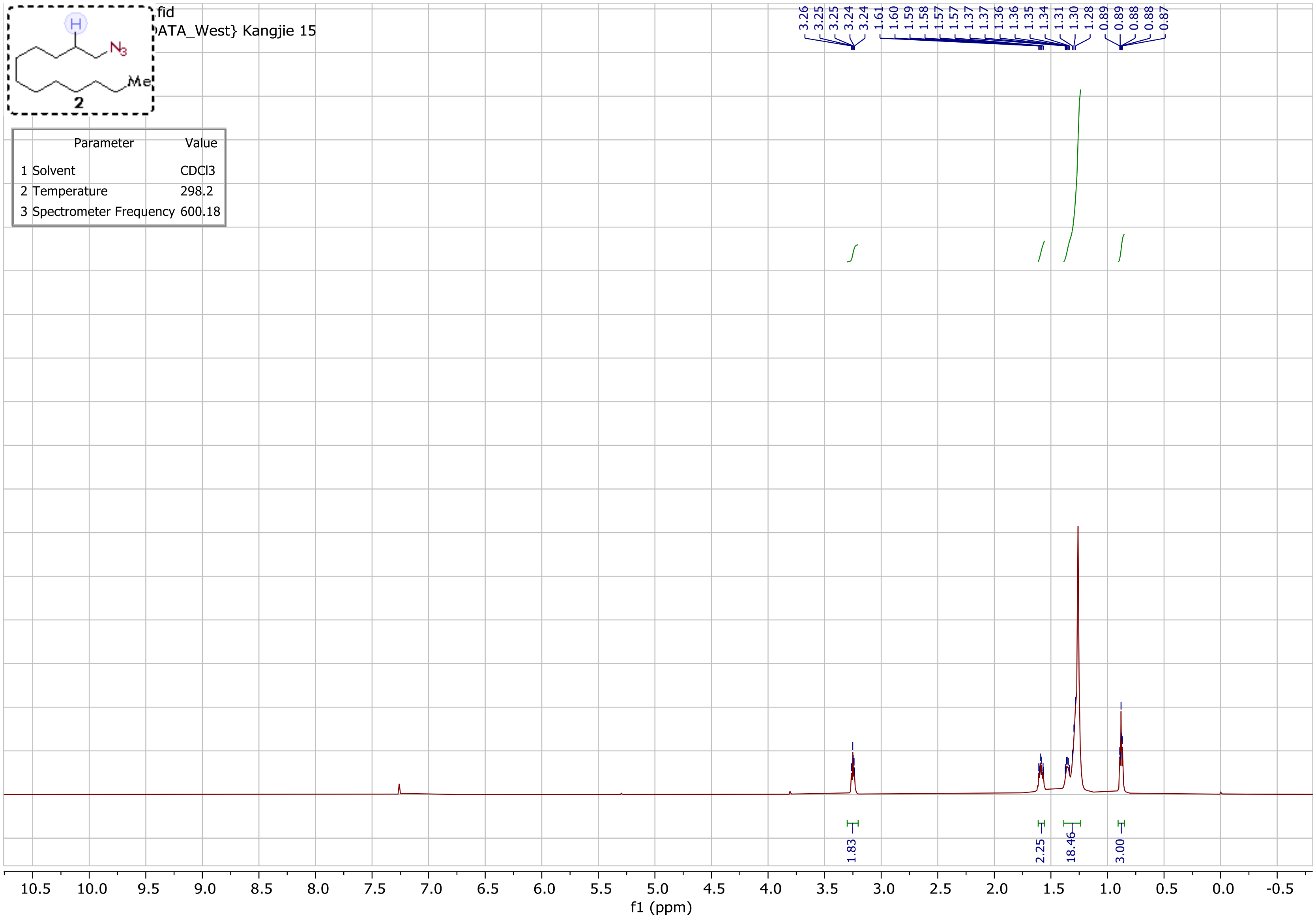

	Parameter	Value
1	Solvent	CDCl3
2	Temperature	298.2
3	Spectrometer Frequency	600.18

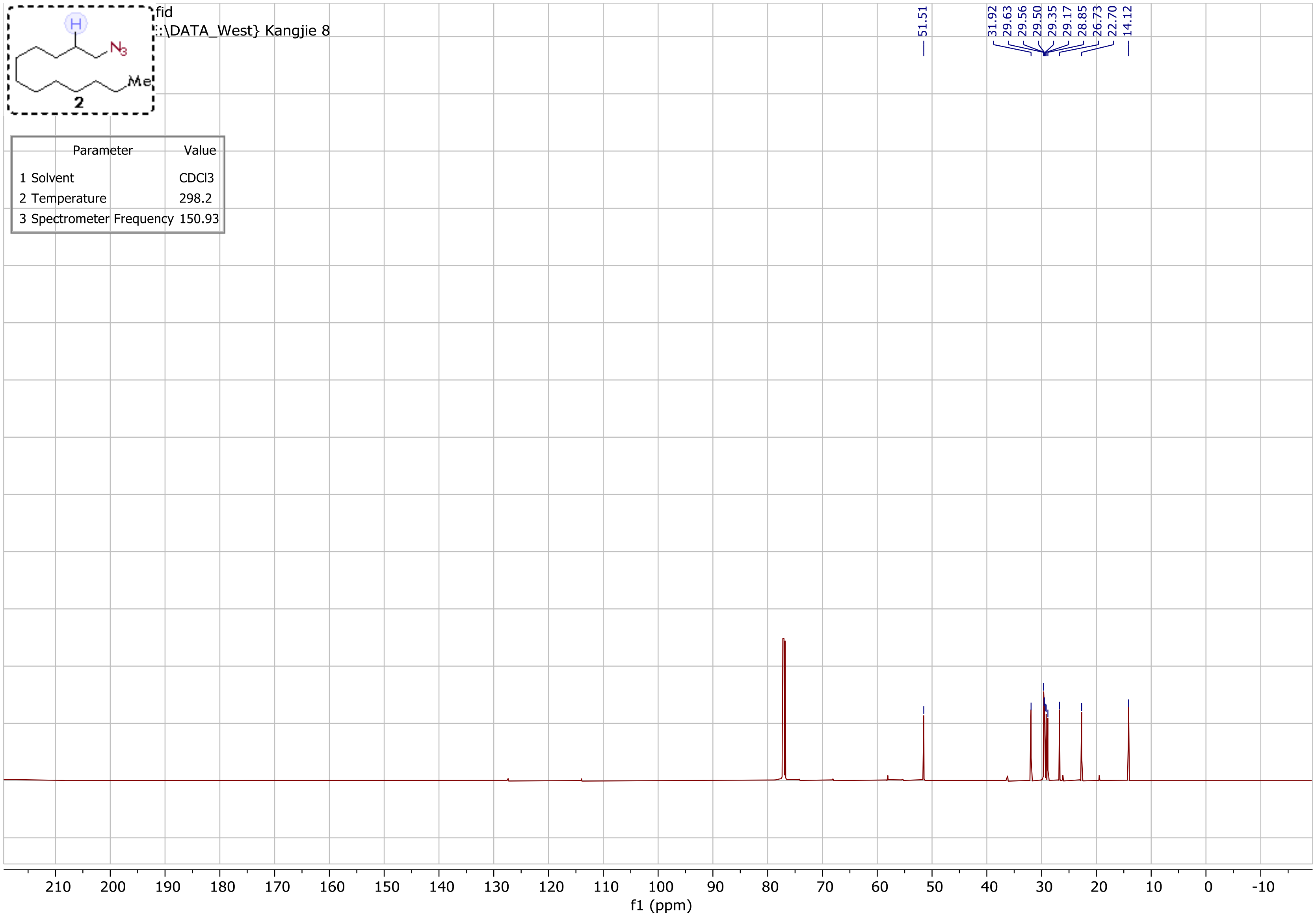
fid
::\DATA_West} Kangjie 8
2
Parameter Value
1 Solvent CDCl3
2 Temperature 298.2
3 Spectrometer Frequency 150.93
51.51
31.92
29.63
29.56
29.50
29.35
29.17
28.85
26.73
22.70
14.12
210 200 190 180 170 160 150 140 130 120 110 100 90 80 70 60 50 40 30 20 10 0 -10
f1 (ppm)

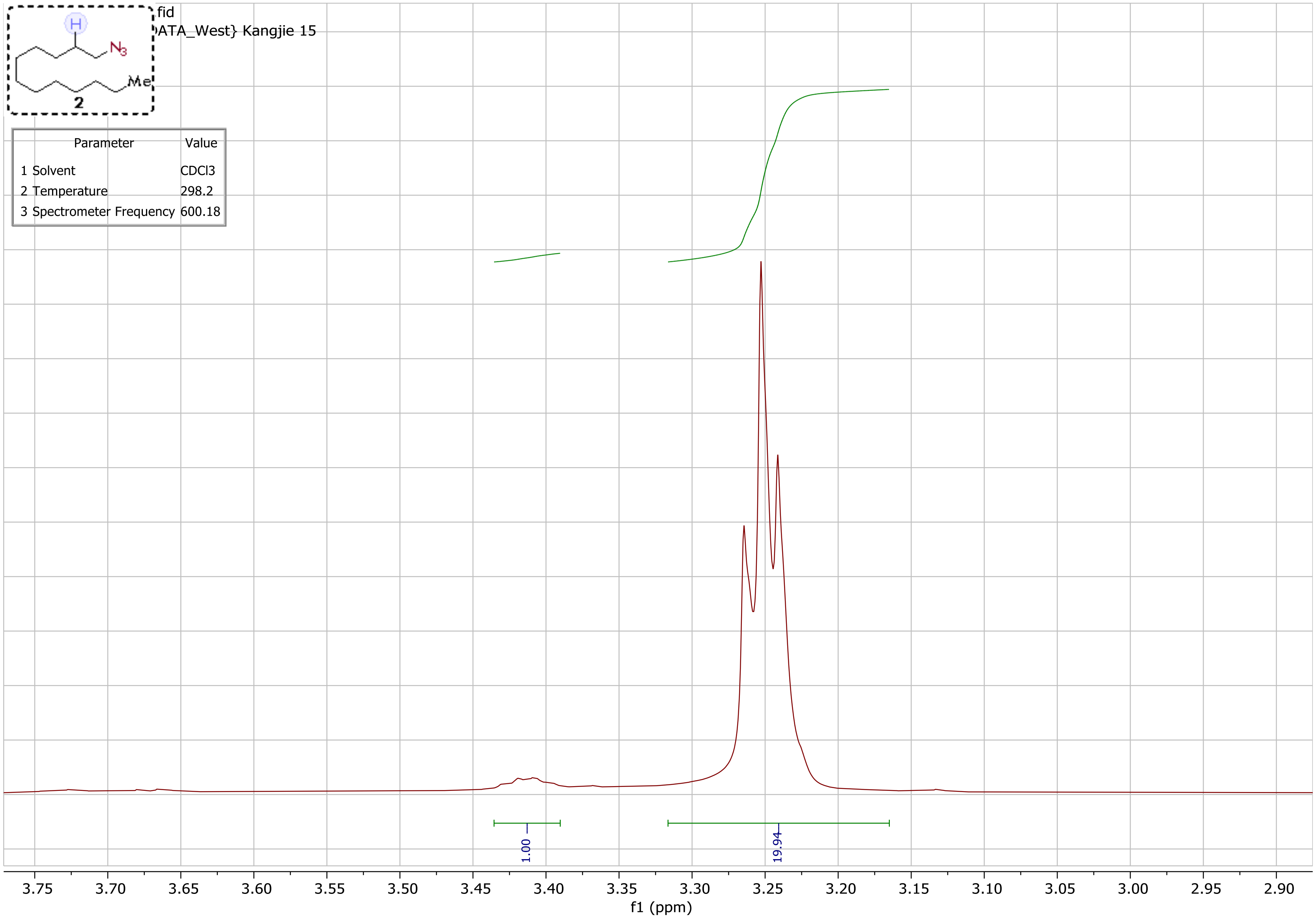
fid
)ATA_West} Kangjie 15
H
N3
Me
2
Parameter Value
1 Solvent CDCl3
2 Temperature 298.2
3 Spectrometer Frequency 600.18
1.00
19.94
3.75 3.70 3.65 3.60 3.55 3.50 3.45 3.40 3.35 3.30 3.25 3.20 3.15 3.10 3.05 3.00 2.95 2.90
f1 (ppm)

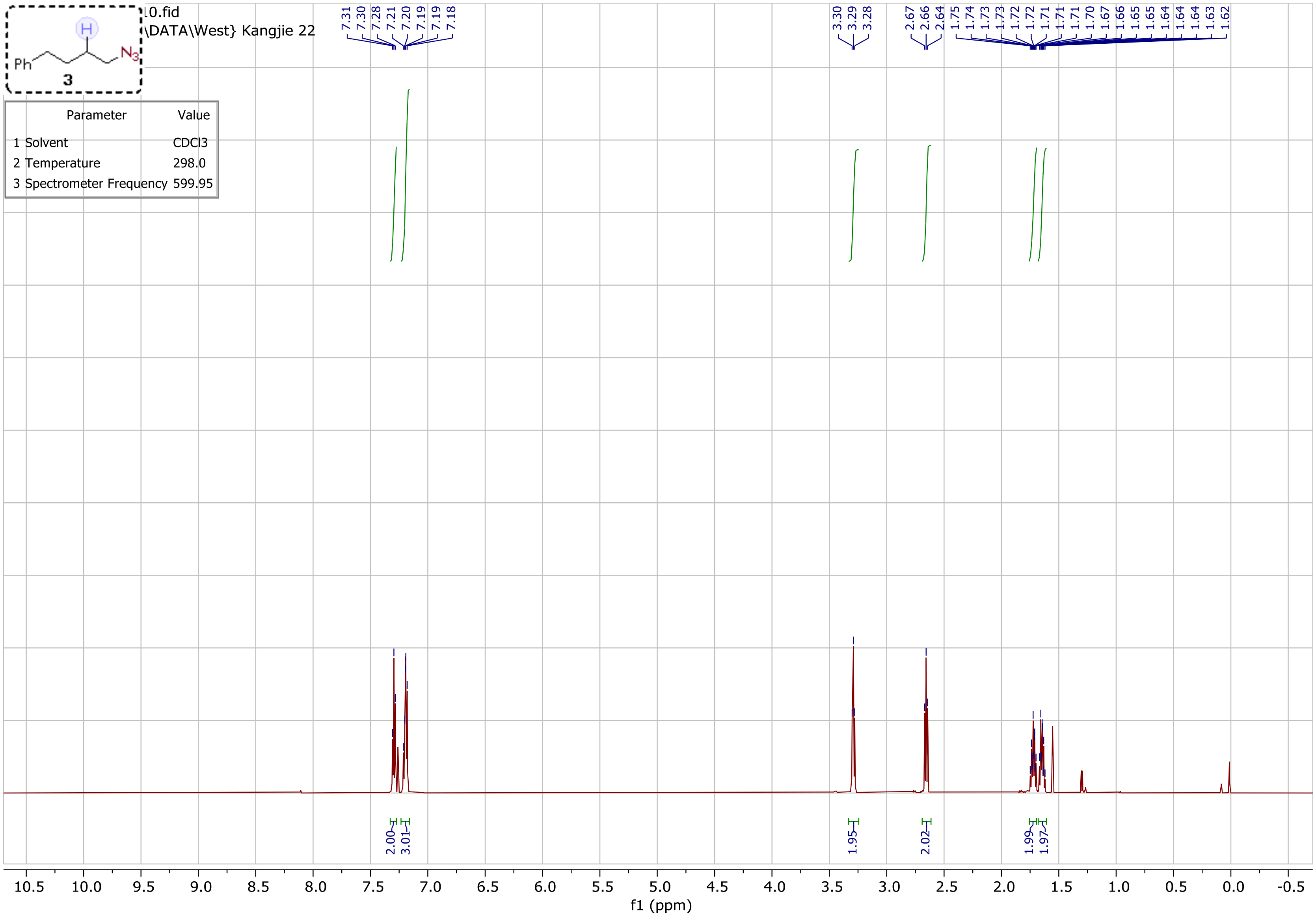

	Parameter	Value
1	Solvent	CDCl3
2	Temperature	298.0
3	Spectrometer Frequency	599.95

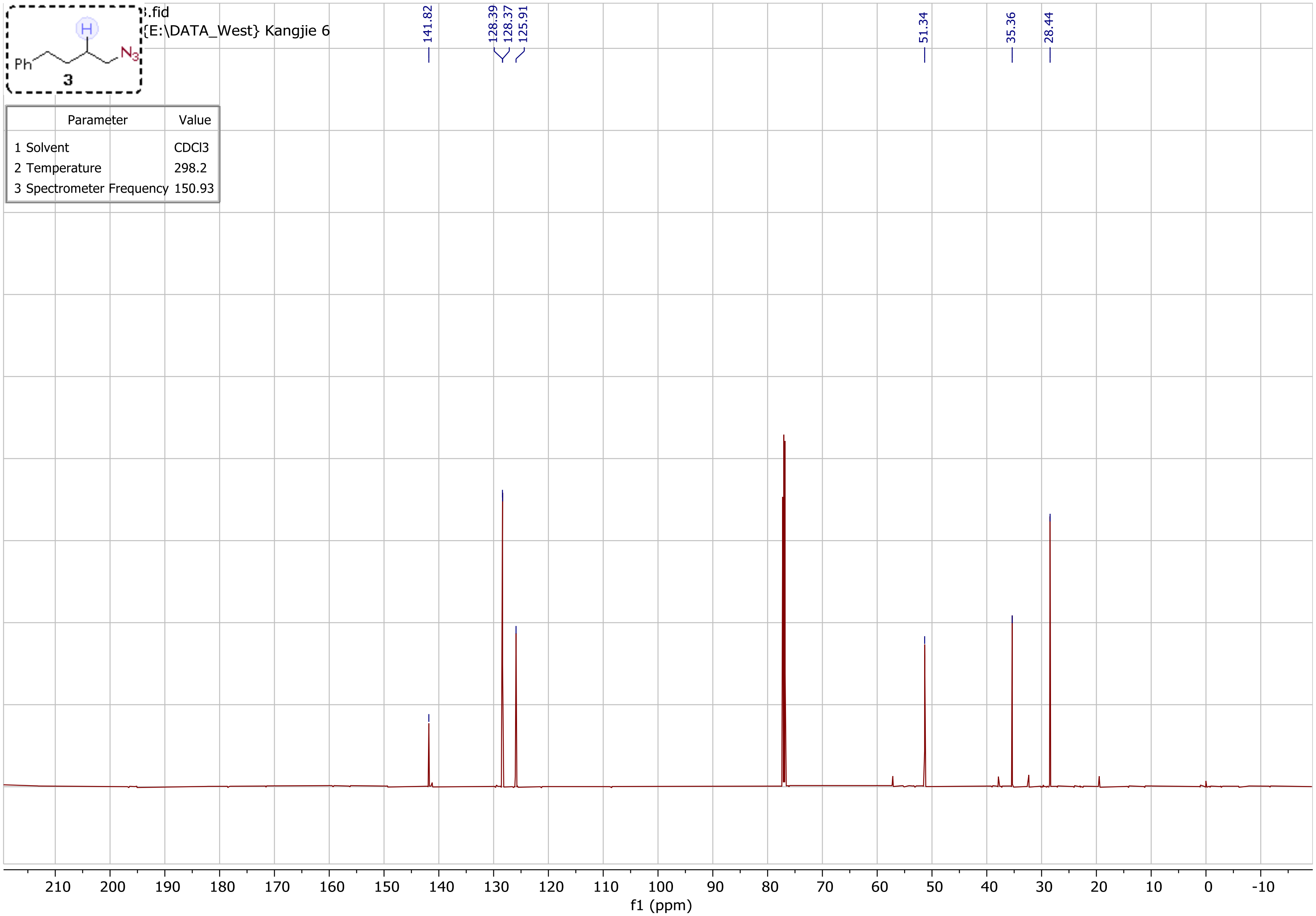

	Parameter	Value
1	Solvent	CDCl3
2	Temperature	298.2
3	Spectrometer Frequency	150.93

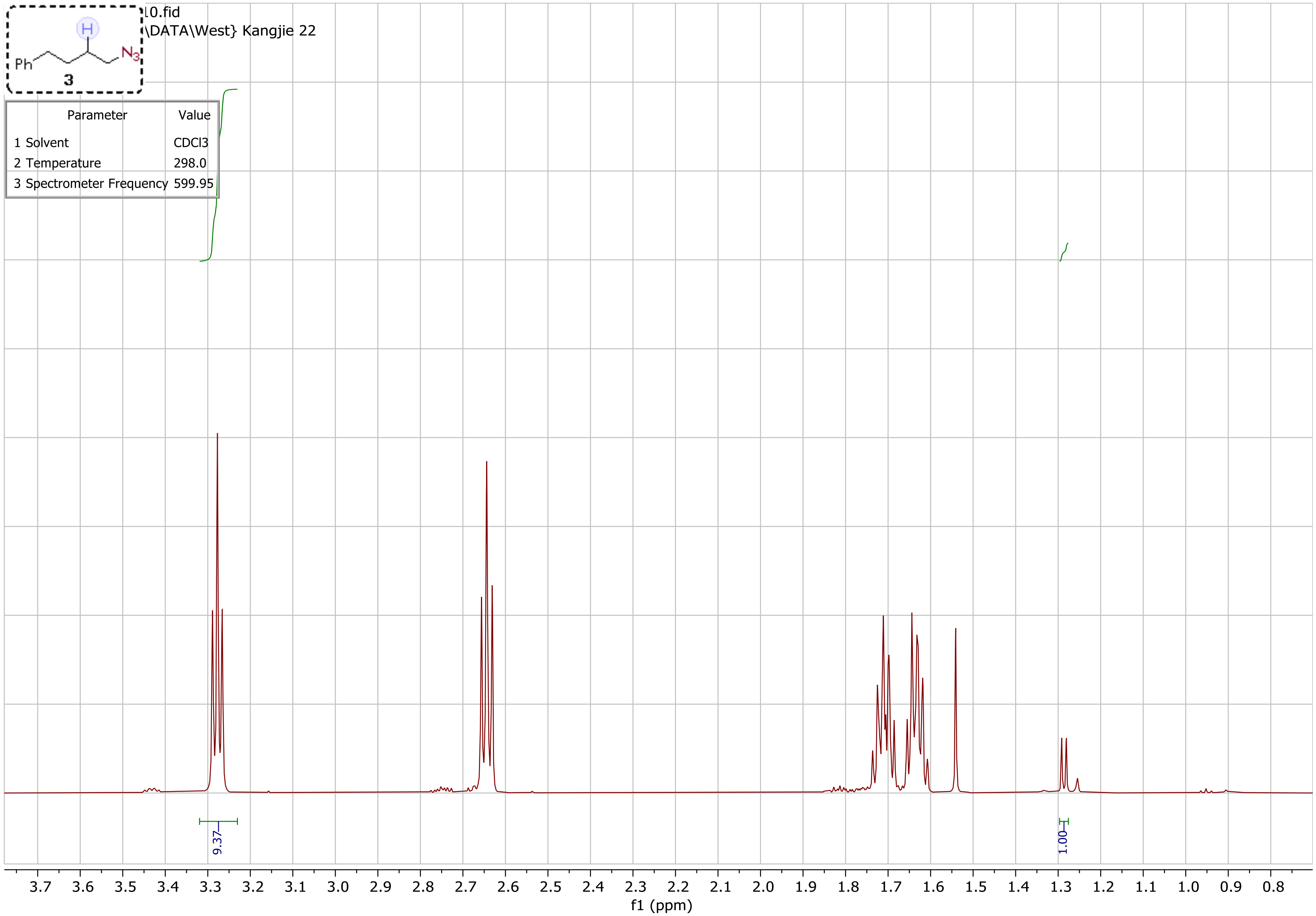

10.fid
\DATA\West} Kangjie 22
Ph
N3
3
Parameter Value
1 Solvent CDCl3
2 Temperature 298.0
3 Spectrometer Frequency 599.95
9.37
1.00
3.7 3.6 3.5 3.4 3.3 3.2 3.1 3.0 2.9 2.8 2.7 2.6 2.5 2.4 2.3 2.2 2.1 2.0 1.9 1.8 1.7 1.6 1.5 1.4 1.3 1.2 1.1 1.0 0.9 0.8
f1 (ppm)

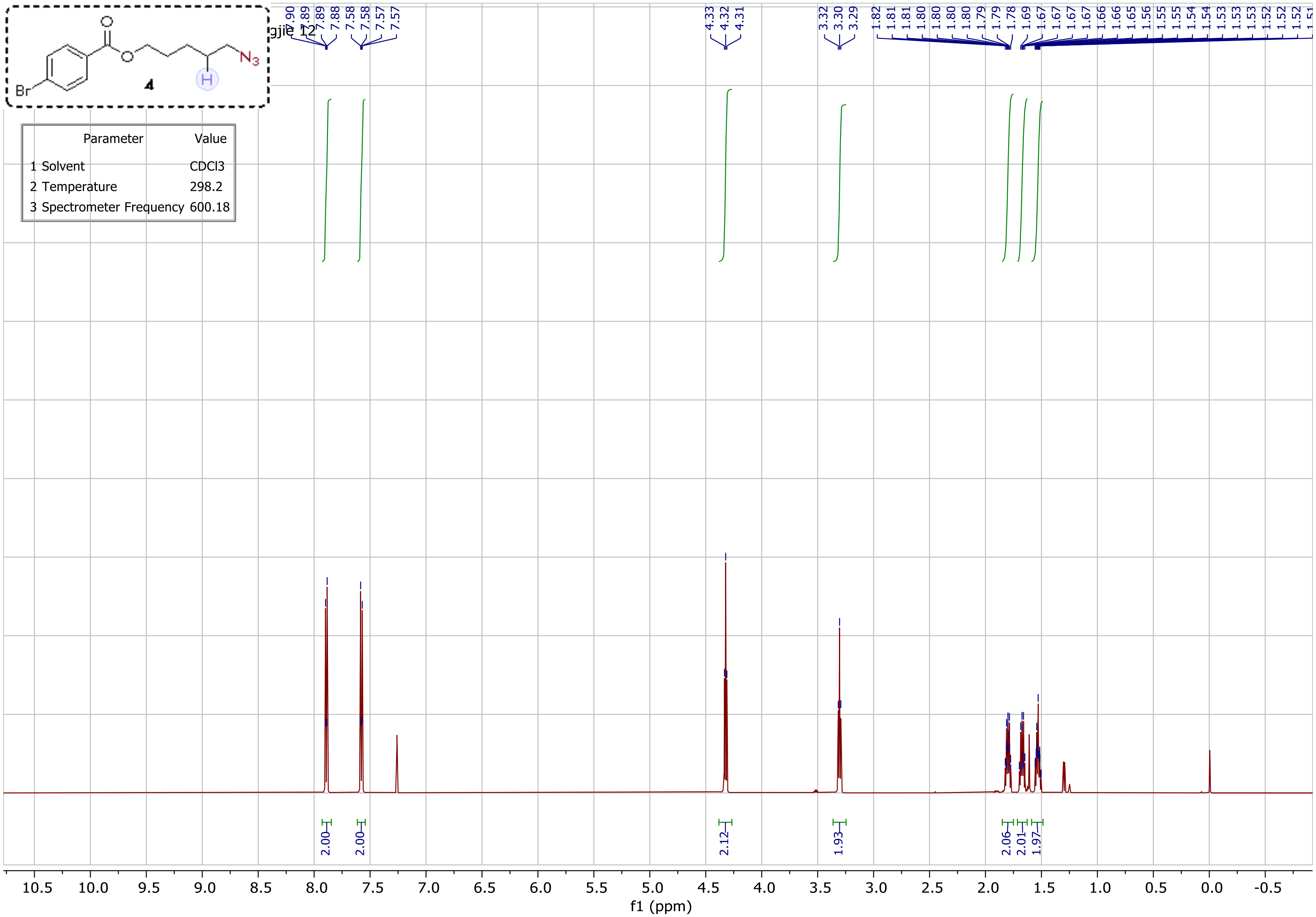
Br
N3
H
4
gjie 12
Parameter Value
1 Solvent CDCl3
2 Temperature 298.2
3 Spectrometer Frequency 600.18
7.90 7.89 7.89 7.88 7.58 7.58 7.57 7.57
4.33 4.32 4.31
3.32 3.30 3.29
1.82 1.81 1.81 1.80 1.80 1.80 1.80 1.79 1.78 1.69 1.67 1.67 1.67 1.67 1.66 1.66 1.65 1.56 1.55 1.55 1.54 1.53 1.53 1.53 1.52 1.52 1.52
2.00 2.00 2.12 1.93 2.06 2.01 1.97
10.5 10.0 9.5 9.0 8.5 8.0 7.5 7.0 6.5 6.0 5.5 5.0 4.5 4.0 3.5 3.0 2.5 2.0 1.5 1.0 0.5 0.0 -0.5
f1 (ppm)

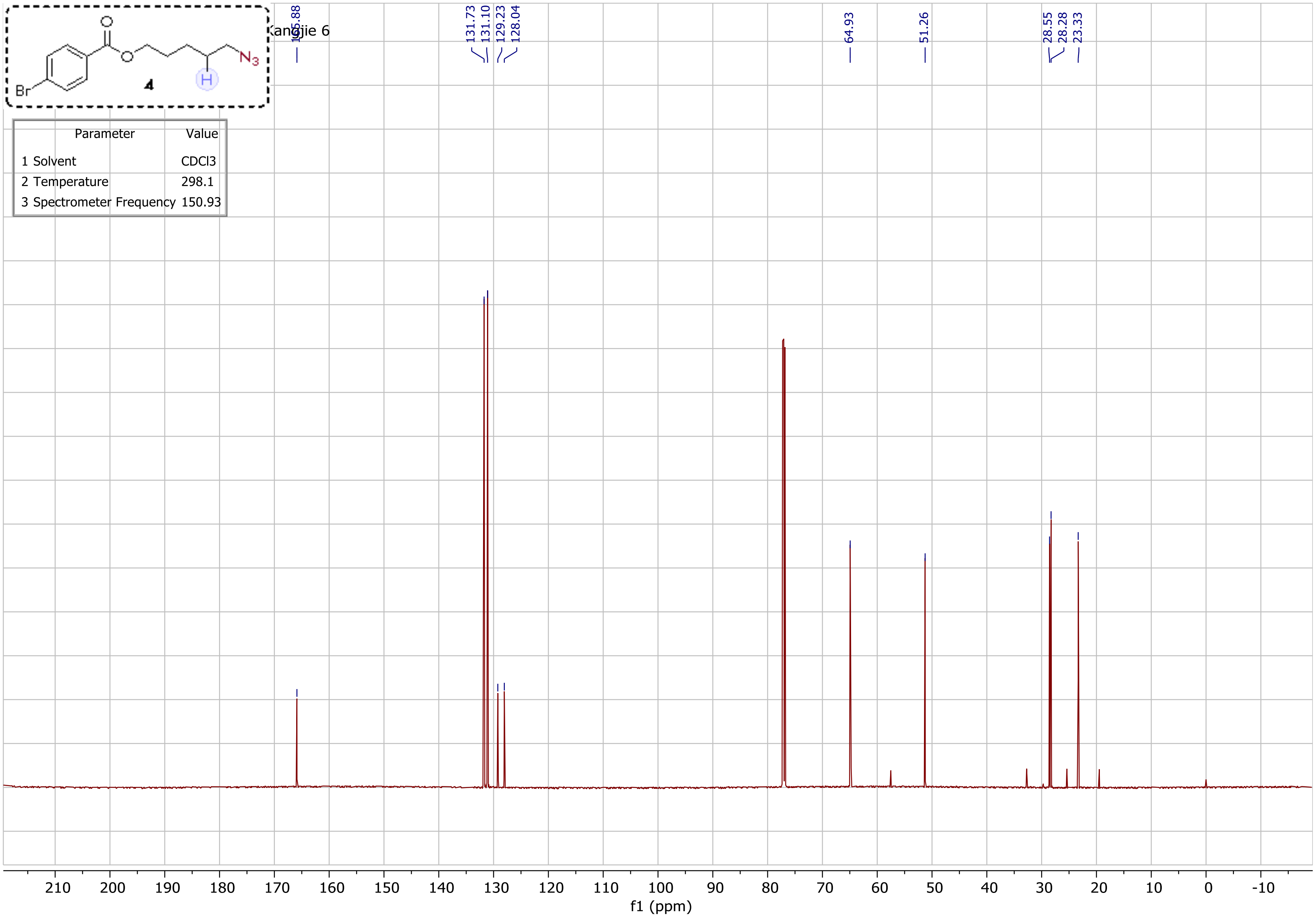

Br
N3
H
4
angjie 6
165.88
131.73
131.10
129.23
128.04
64.93
51.26
28.55
28.28
23.33
Parameter Value
1 Solvent CDCl3
2 Temperature 298.1
3 Spectrometer Frequency 150.93
210 200 190 180 170 160 150 140 130 120 110 100 90 80 70 60 50 40 30 20 10 0 -10
f1 (ppm)

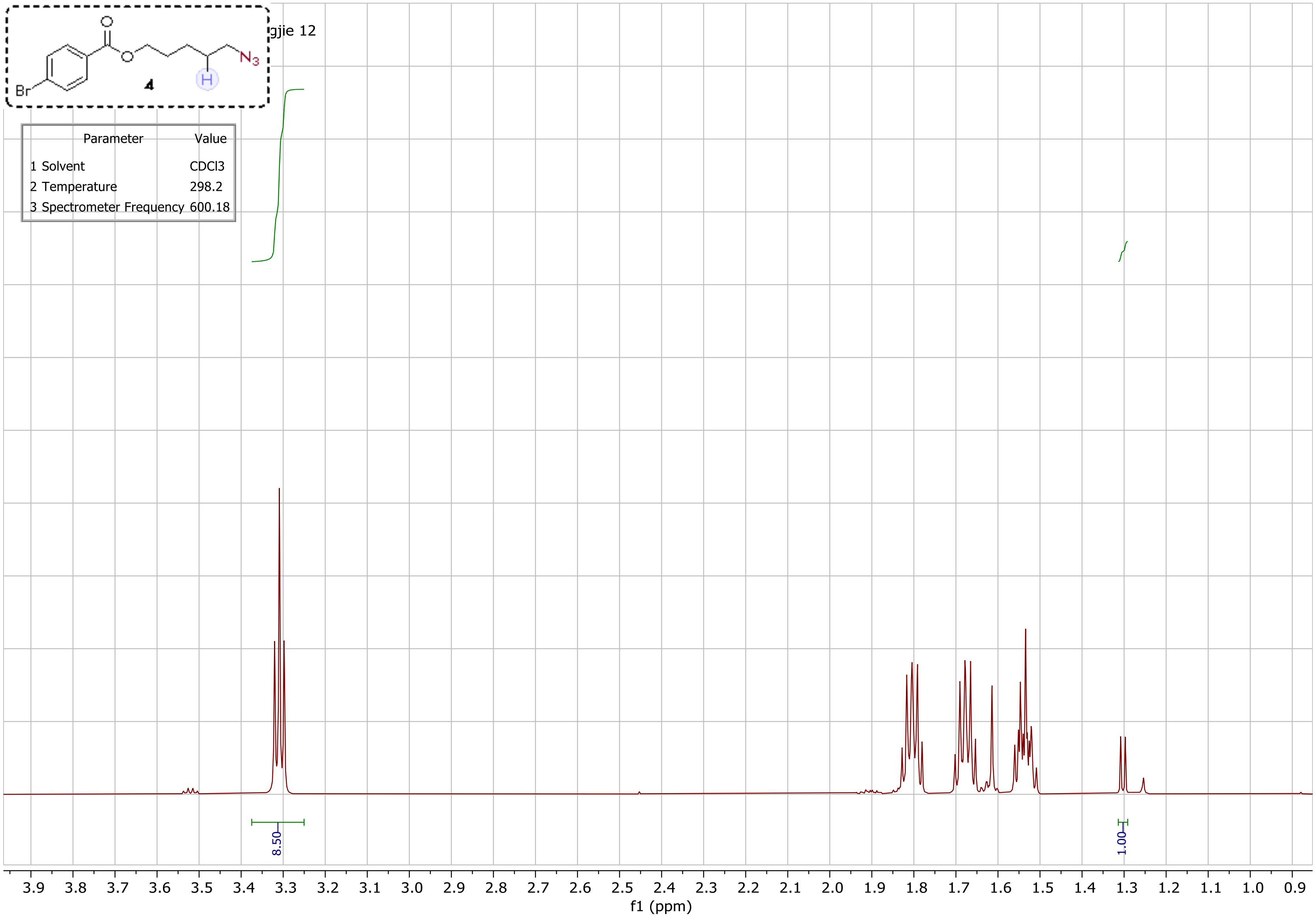

Parameter	Value
1 Solvent	CDCl3
2 Temperature	298.2
3 Spectrometer Frequency	600.18

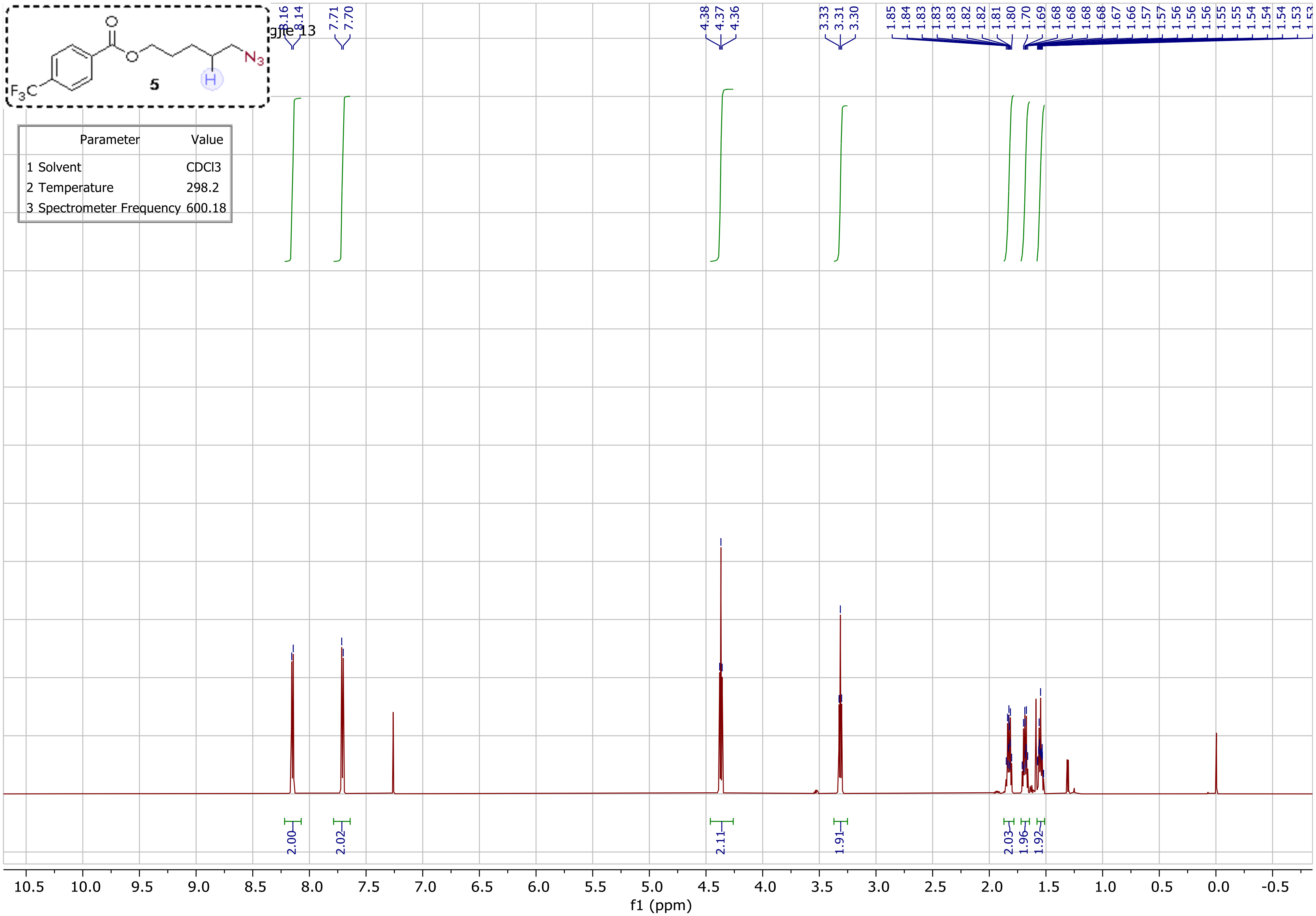
5
F3C
N3
H
Parameter Value
1 Solvent CDCl3
2 Temperature 298.2
3 Spectrometer Frequency 600.18
8.16
8.14
7.71
7.70
4.38
4.37
4.36
3.33
3.31
3.30
1.85
1.84
1.83
1.83
1.83
1.82
1.82
1.81
1.80
1.70
1.69
1.68
1.68
1.68
1.67
1.66
1.57
1.56
1.56
1.56
1.55
1.55
1.54
1.54
1.53
2.00
2.02
2.11
1.91
2.03
1.96
1.92
10.5 10.0 9.5 9.0 8.5 8.0 7.5 7.0 6.5 6.0 5.5 5.0 4.5 4.0 3.5 3.0 2.5 2.0 1.5 1.0 0.5 0.0 -0.5
f1 (ppm)

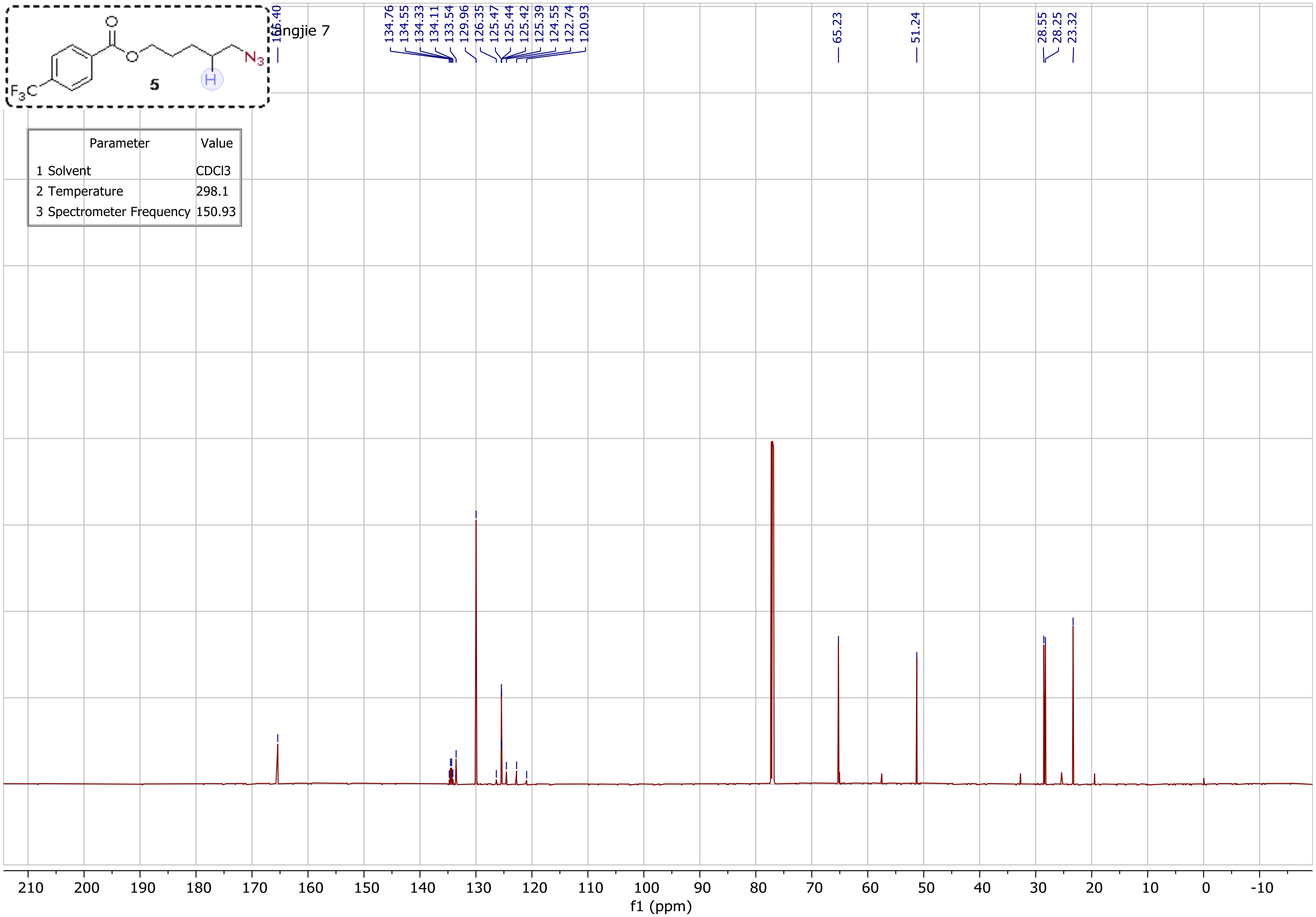

	Parameter	Value
1	Solvent	CDCl3
2	Temperature	298.1
3	Spectrometer Frequency	150.93

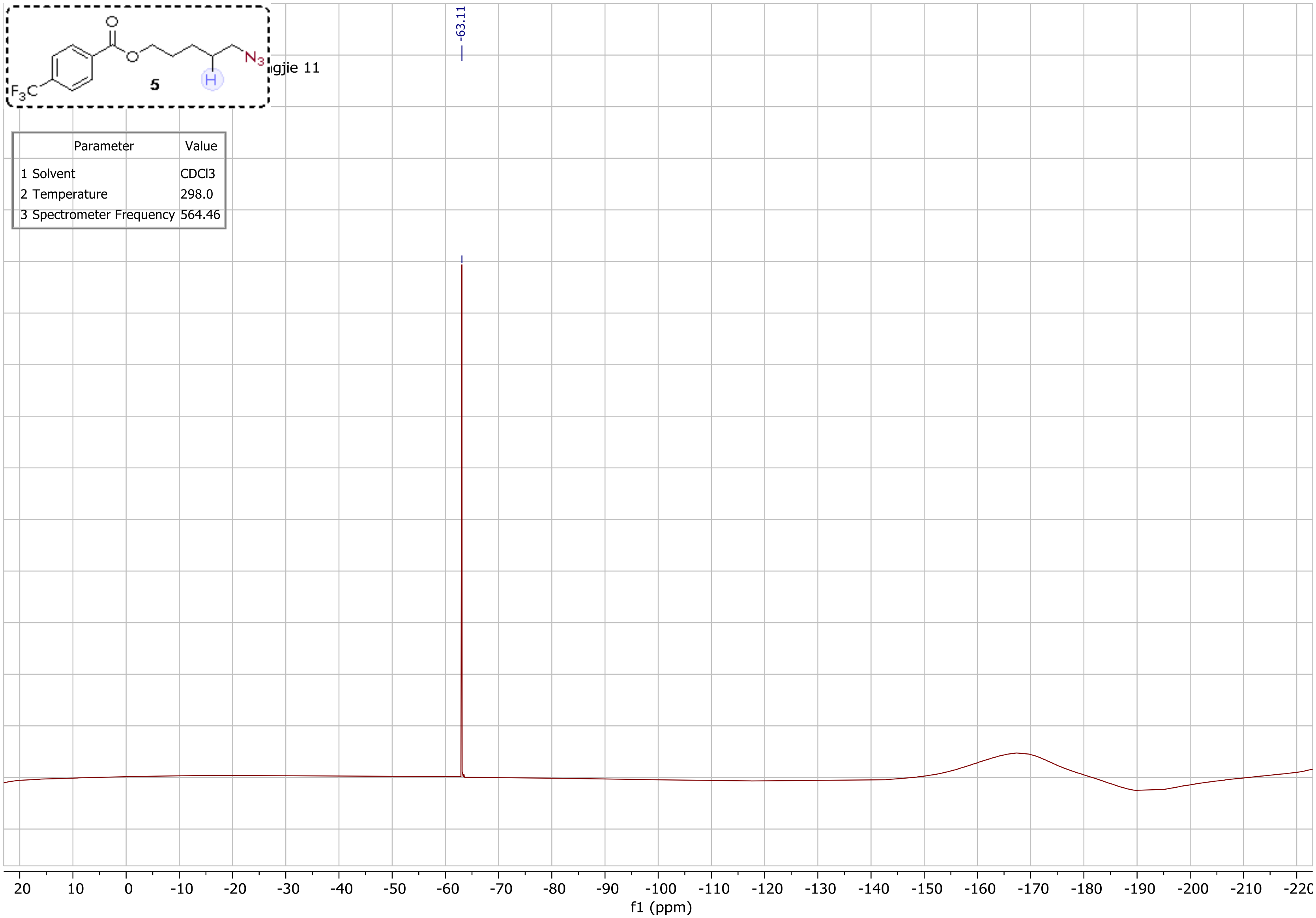

	Parameter	Value
1	Solvent	CDCl3
2	Temperature	298.0
3	Spectrometer Frequency	564.46

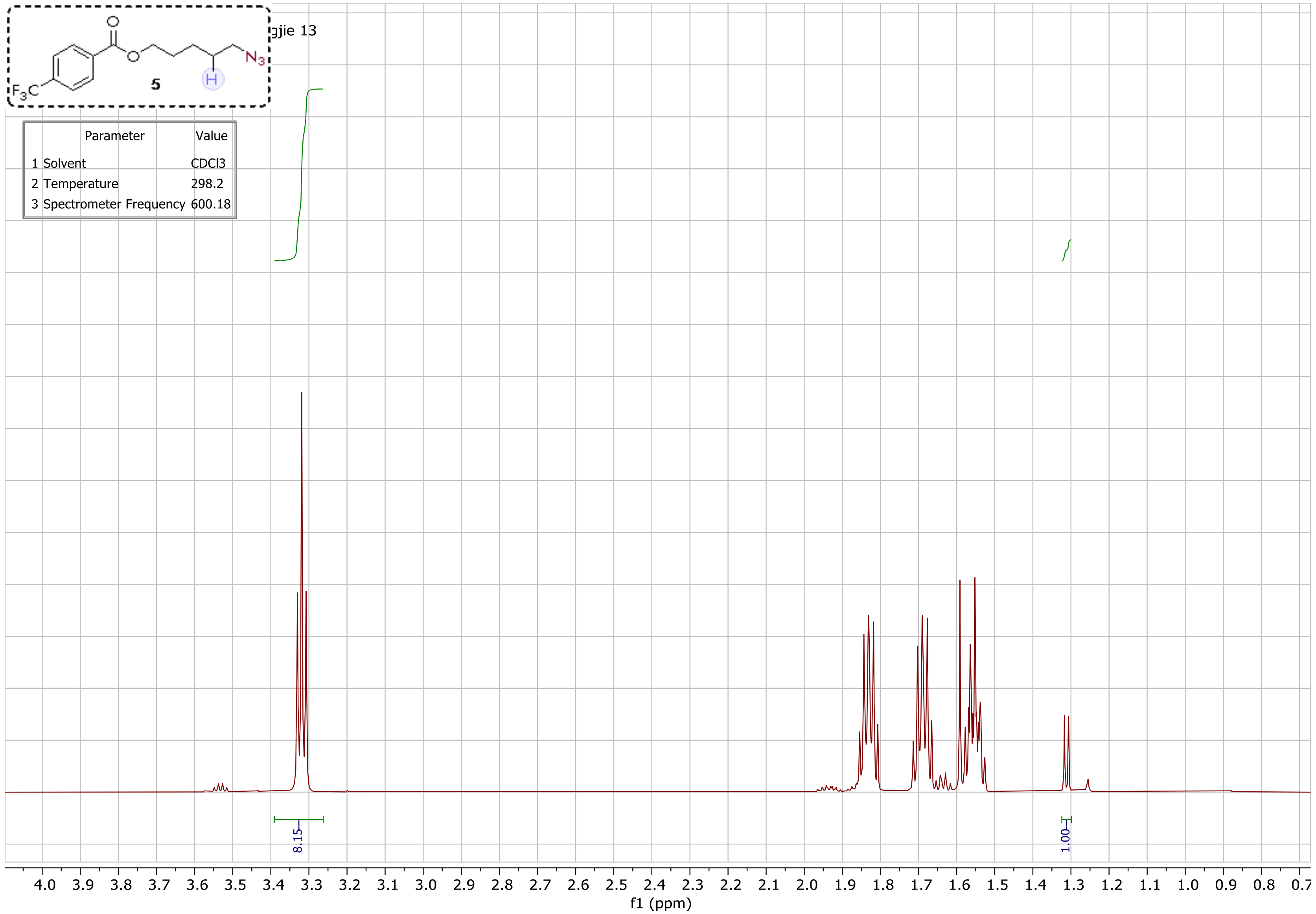

F3C
N3
H
5
gjie 13
Parameter
Value
1 Solvent
CDCl3
2 Temperature
298.2
3 Spectrometer Frequency
600.18
8.15
1.00
4.0 3.9 3.8 3.7 3.6 3.5 3.4 3.3 3.2 3.1 3.0 2.9 2.8 2.7 2.6 2.5 2.4 2.3 2.2 2.1 2.0 1.9 1.8 1.7 1.6 1.5 1.4 1.3 1.2 1.1 1.0 0.9 0.8 0.7
f1 (ppm)

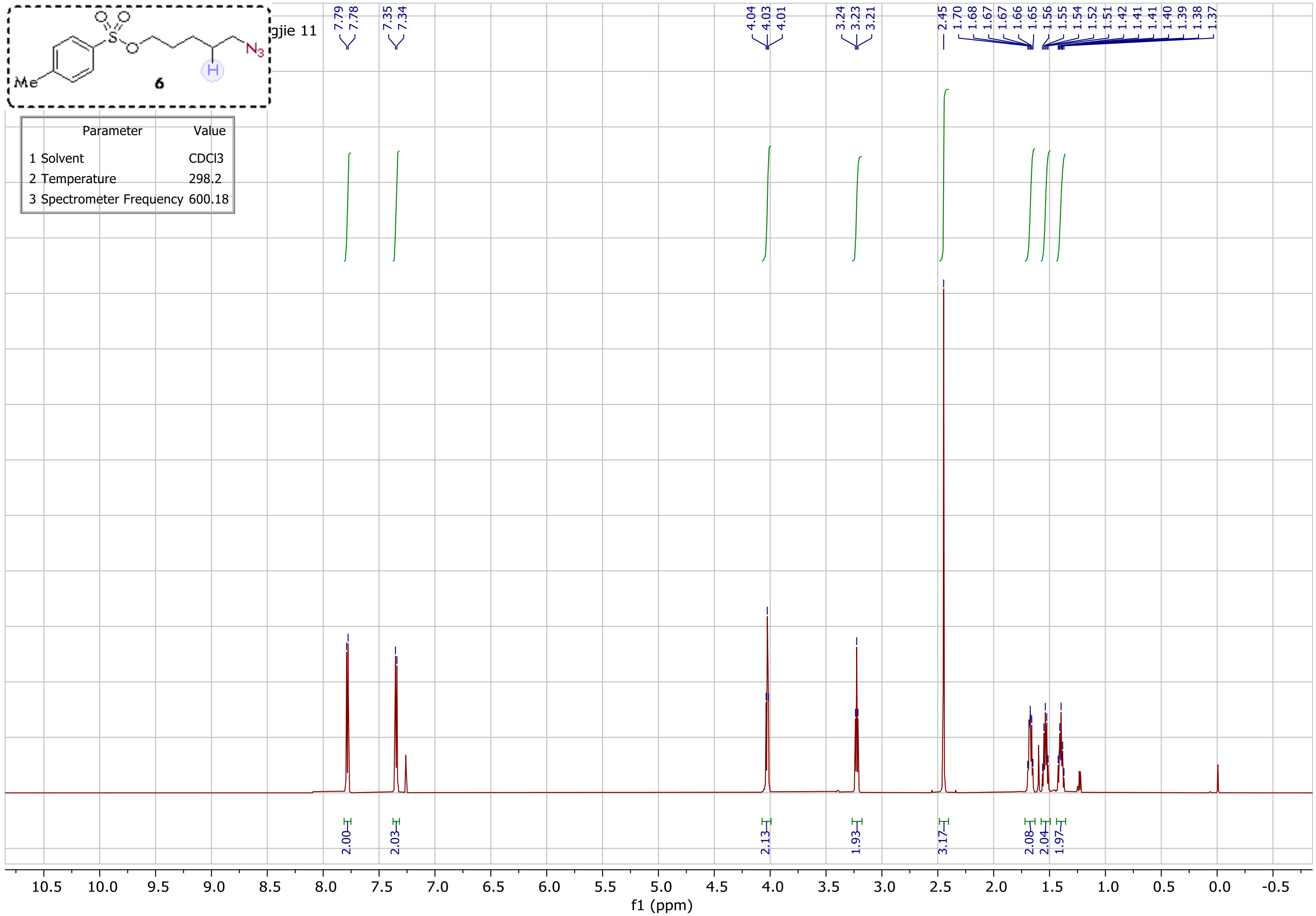
Me
6
N3
H
gjie 11
Parameter Value
1 Solvent CDCl3
2 Temperature 298.2
3 Spectrometer Frequency 600.18
7.79 7.78 7.35 7.34 4.04 4.03 4.01 3.24 3.23 3.21 2.45 1.70 1.68 1.67 1.67 1.66 1.65 1.56 1.55 1.54 1.52 1.51 1.42 1.41 1.41 1.40 1.39 1.38 1.37
2.00 2.03 2.13 1.93 3.17 2.08 2.04 1.97
10.5 10.0 9.5 9.0 8.5 8.0 7.5 7.0 6.5 6.0 5.5 5.0 4.5 4.0 3.5 3.0 2.5 2.0 1.5 1.0 0.5 0.0 -0.5
f1 (ppm)

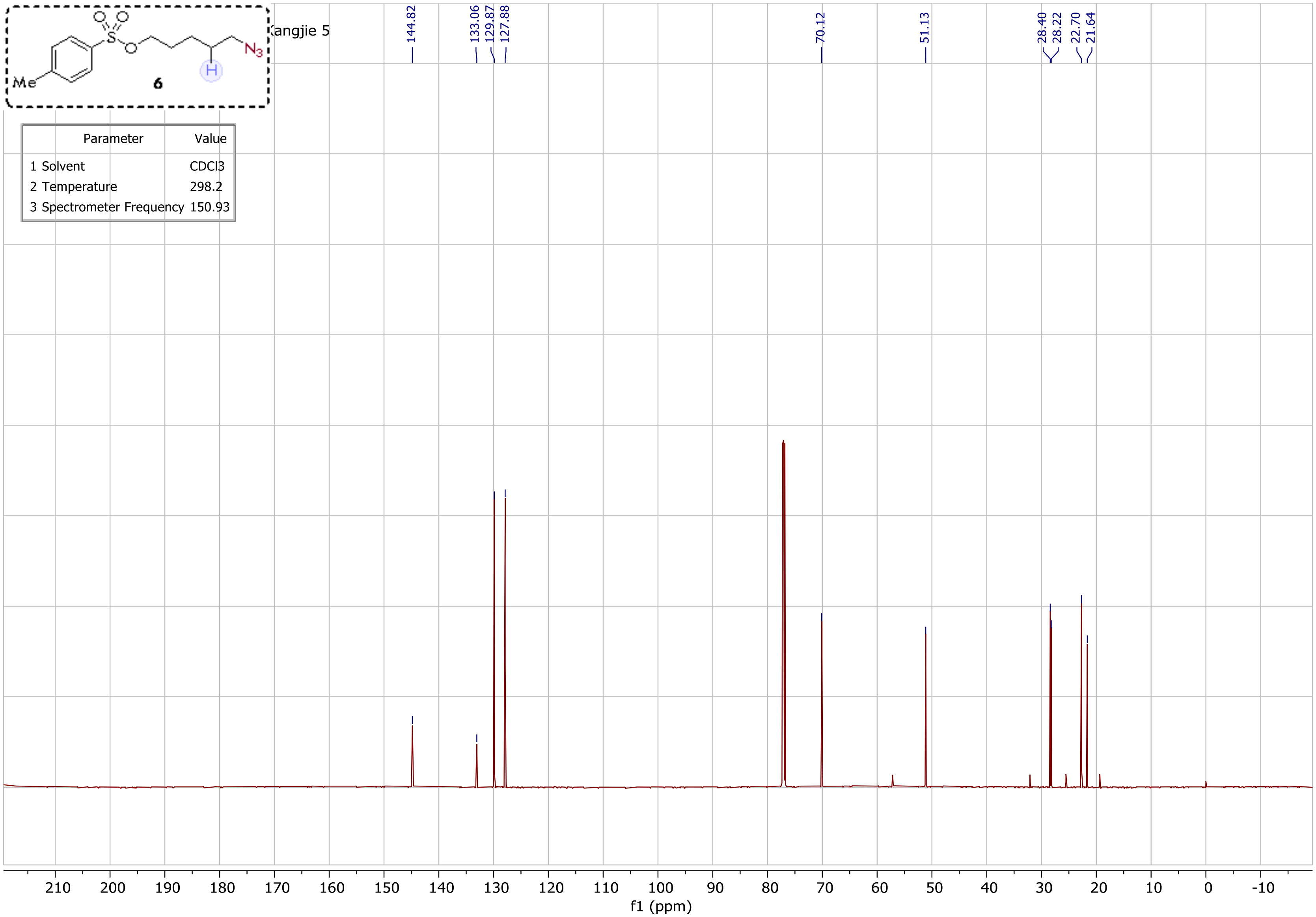
Me
6
N3
H
angjie 5
144.82
133.06
129.87
127.88
70.12
51.13
28.40
28.22
22.70
21.64
Parameter | Value
1 Solvent | CDCl3
2 Temperature | 298.2
3 Spectrometer Frequency | 150.93
210 200 190 180 170 160 150 140 130 120 110 100 90 80 70 60 50 40 30 20 10 0 -10
f1 (ppm)

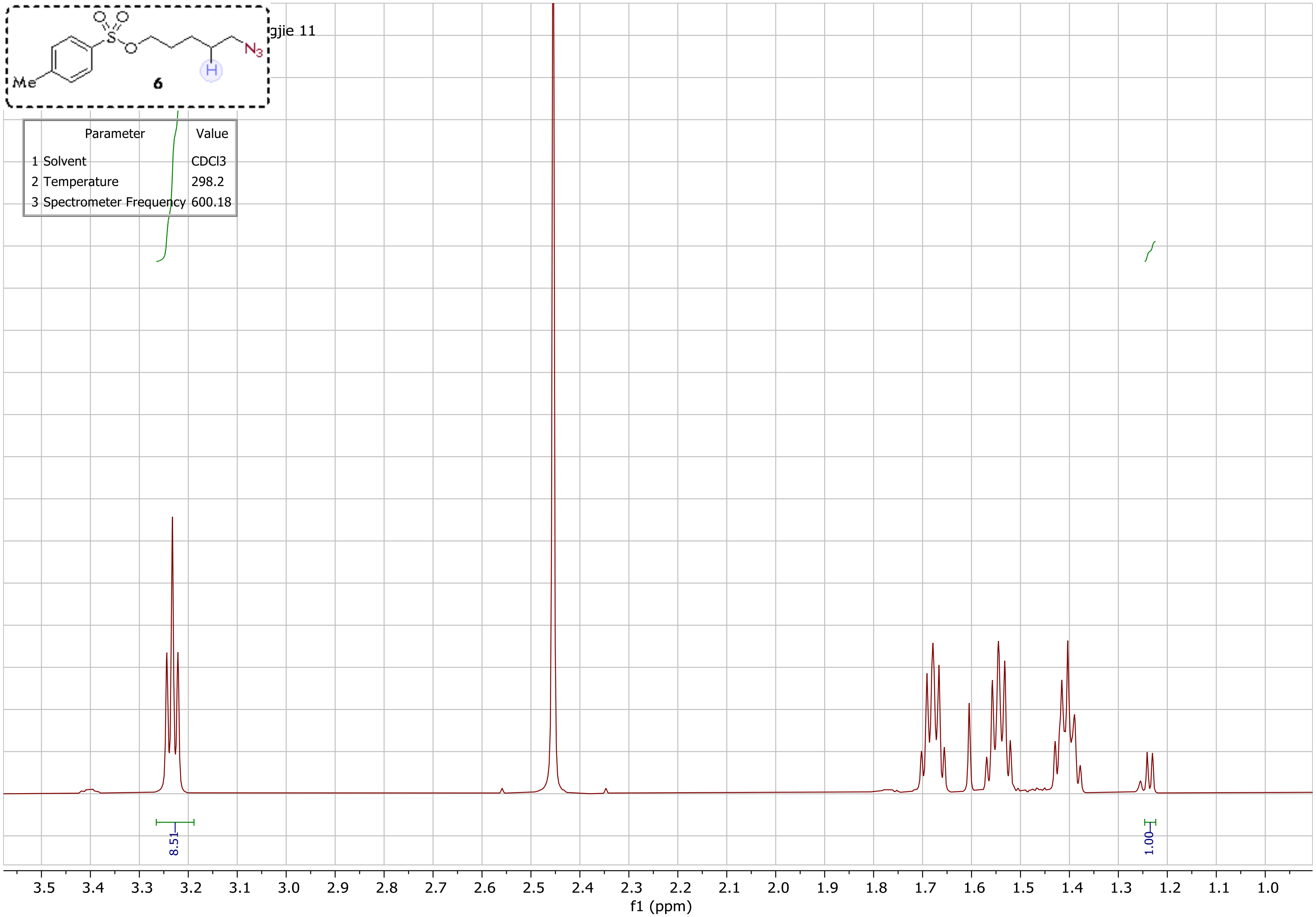

Me
6
N3
H
gjie 11
Parameter Value
1 Solvent CDCl3
2 Temperature 298.2
3 Spectrometer Frequency 600.18
8.51
1.00
3.5 3.4 3.3 3.2 3.1 3.0 2.9 2.8 2.7 2.6 2.5 2.4 2.3 2.2 2.1 2.0 1.9 1.8 1.7 1.6 1.5 1.4 1.3 1.2 1.1 1.0
f1 (ppm)

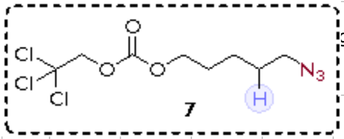

Parameter	Value
1 Solvent	CDCl3
2 Temperature	298.2
3 Spectrometer Frequency	600.18

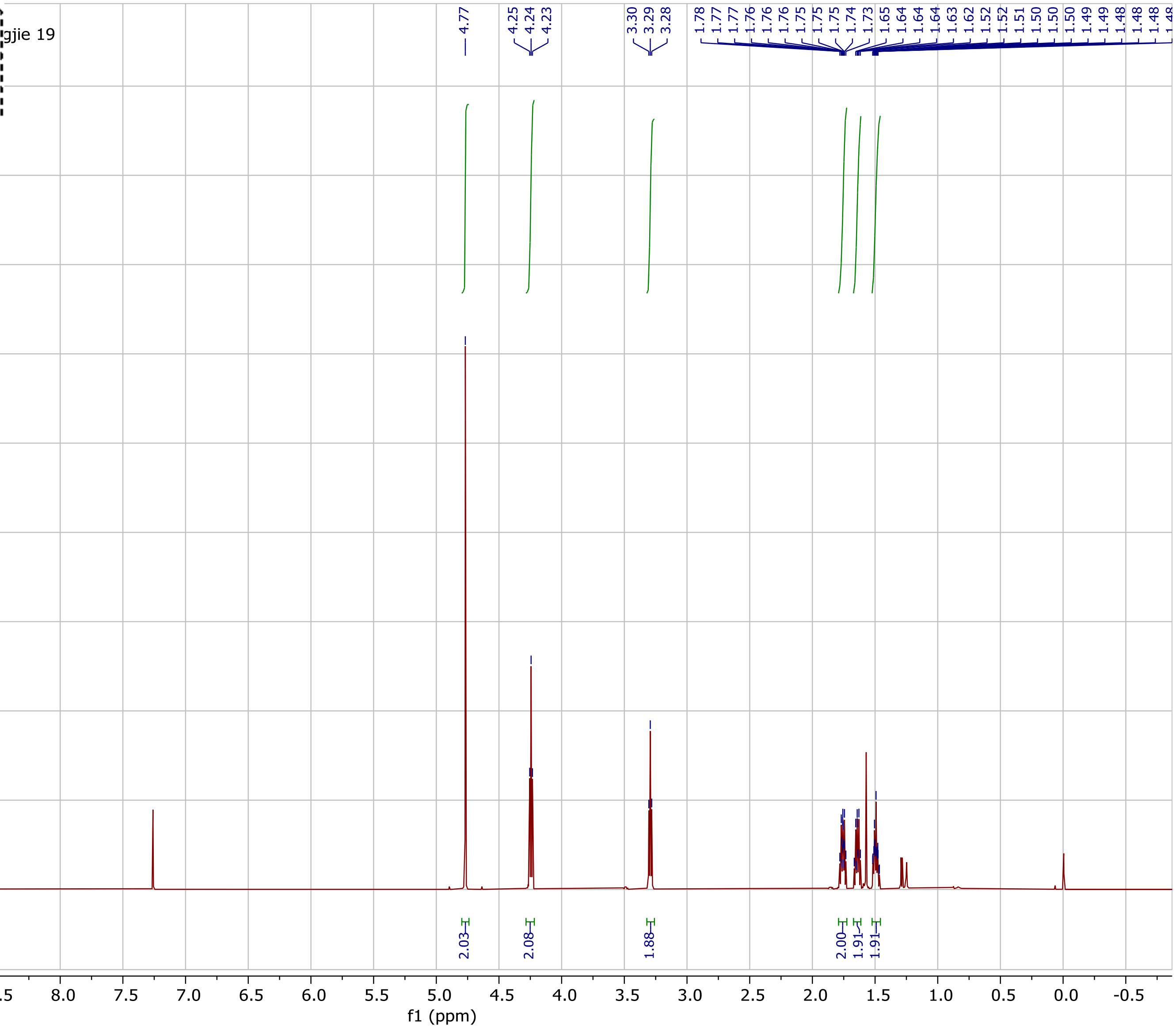

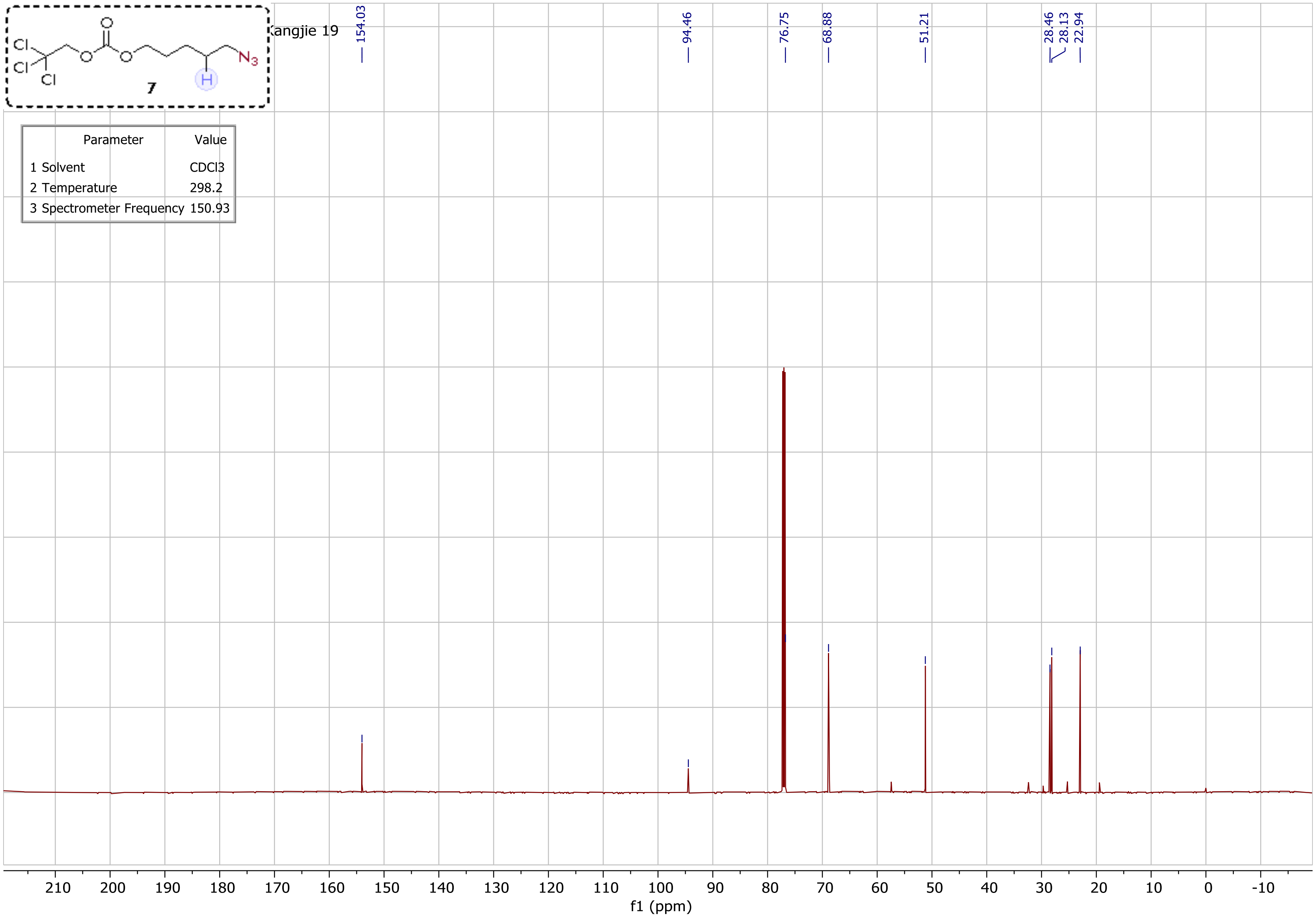
Cl Cl Cl O O O N3 H
7
angjie 19
154.03
94.46
76.75
68.88
51.21
28.46
28.13
22.94
Parameter Value
1 Solvent CDCl3
2 Temperature 298.2
3 Spectrometer Frequency 150.93
210 200 190 180 170 160 150 140 130 120 110 100 90 80 70 60 50 40 30 20 10 0 -10
f1 (ppm)

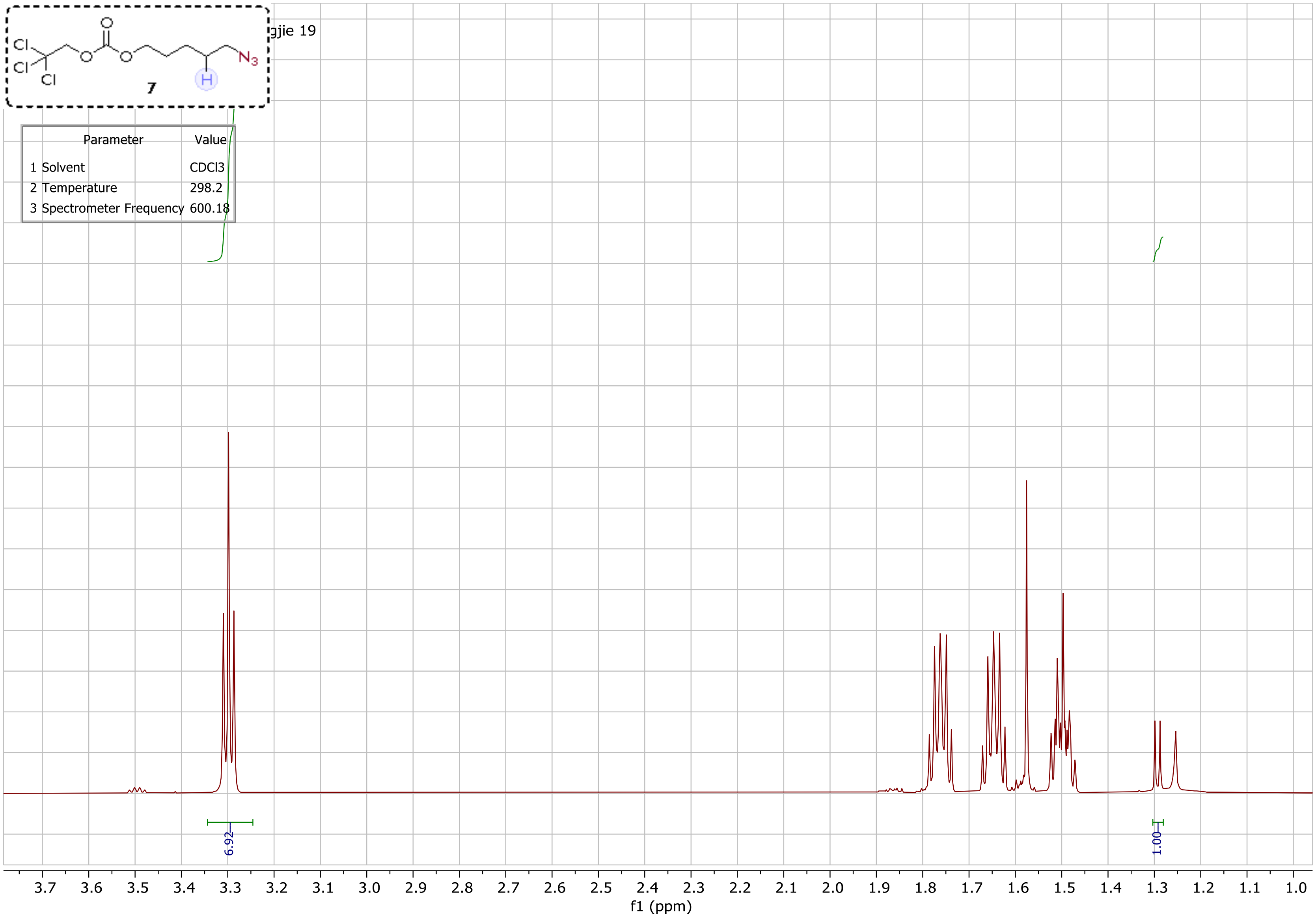
7
gjie 19
Parameter Value
1 Solvent CDCl3
2 Temperature 298.2
3 Spectrometer Frequency 600.18
6.92
1.00
3.7 3.6 3.5 3.4 3.3 3.2 3.1 3.0 2.9 2.8 2.7 2.6 2.5 2.4 2.3 2.2 2.1 2.0 1.9 1.8 1.7 1.6 1.5 1.4 1.3 1.2 1.1 1.0
f1 (ppm)

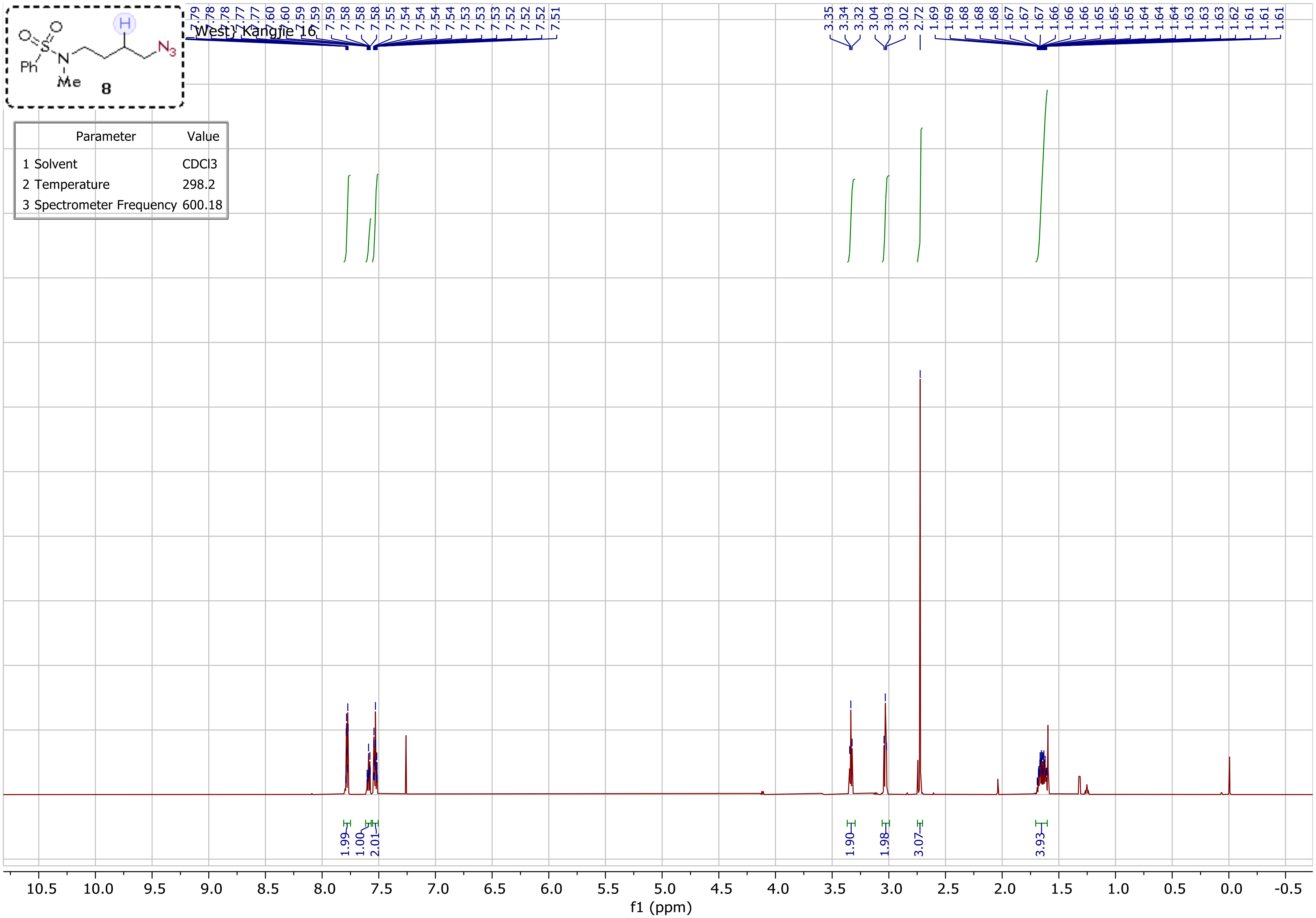
West} Kangjie 16
8
Parameter Value
1 Solvent CDCl3
2 Temperature 298.2
3 Spectrometer Frequency 600.18
f1 (ppm)

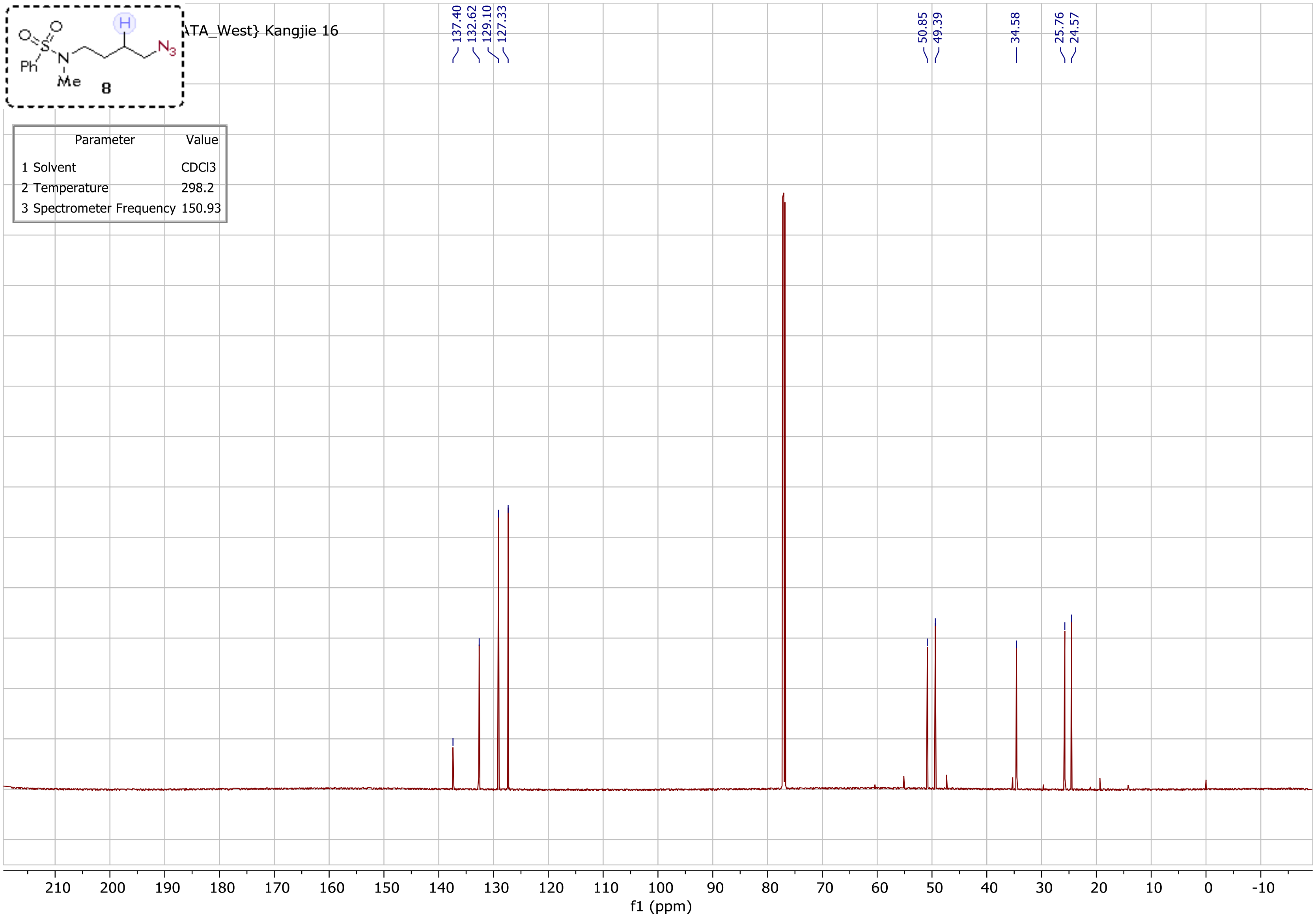
Ph
Me
N
N_3
H
8
ATA_West} Kangjie 16
137.40
132.62
129.10
127.33
50.85
49.39
34.58
25.76
24.57
Parameter Value
1 Solvent CDCl3
2 Temperature 298.2
3 Spectrometer Frequency 150.93
210 200 190 180 170 160 150 140 130 120 110 100 90 80 70 60 50 40 30 20 10 0 -10
f1 (ppm)

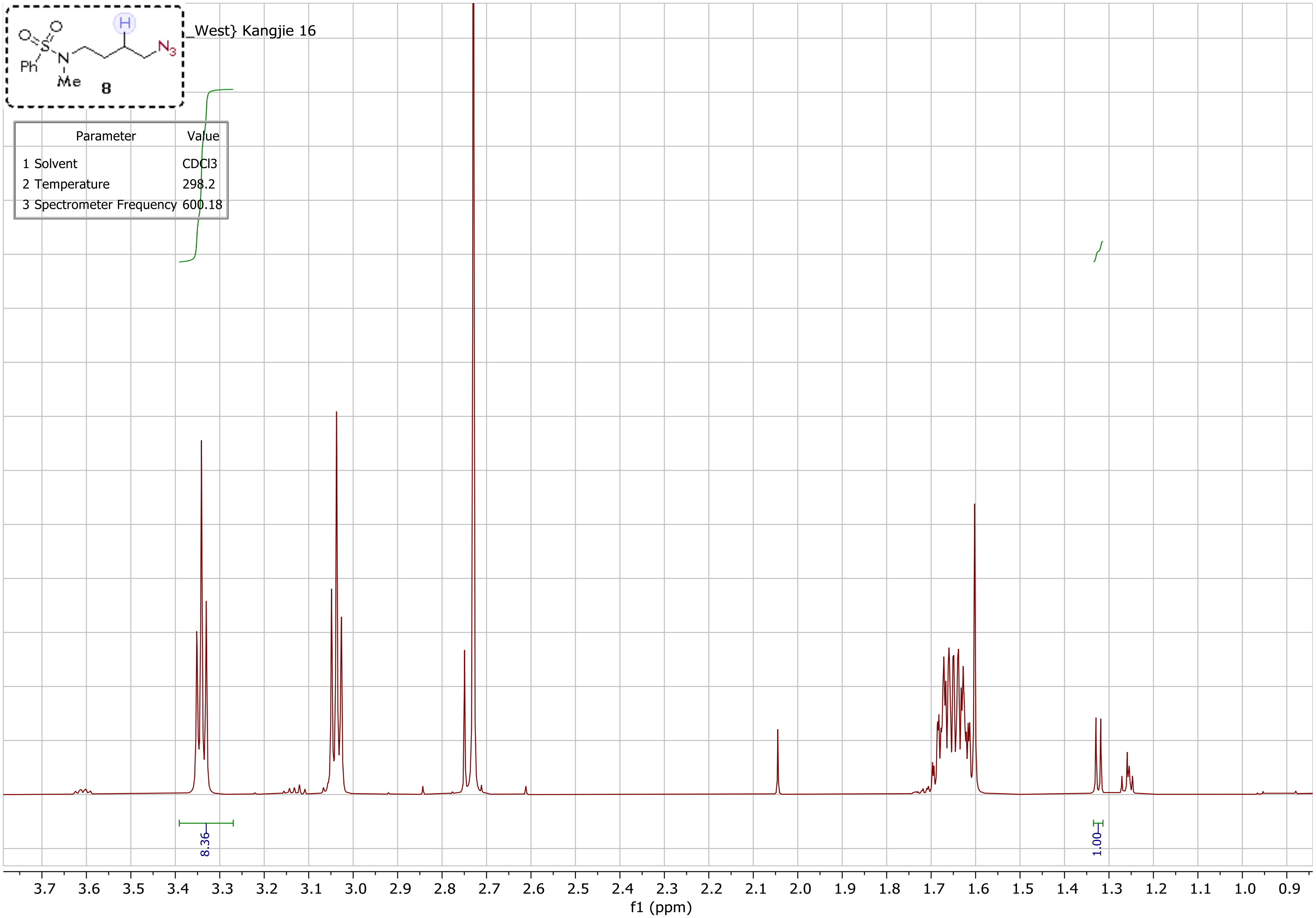

_West} Kangjie 16
8
Parameter | Value
1 Solvent | CDCl3
2 Temperature | 298.2
3 Spectrometer Frequency | 600.18
8.36
1.00
3.7 3.6 3.5 3.4 3.3 3.2 3.1 3.0 2.9 2.8 2.7 2.6 2.5 2.4 2.3 2.2 2.1 2.0 1.9 1.8 1.7 1.6 1.5 1.4 1.3 1.2 1.1 1.0 0.9
f1 (ppm)

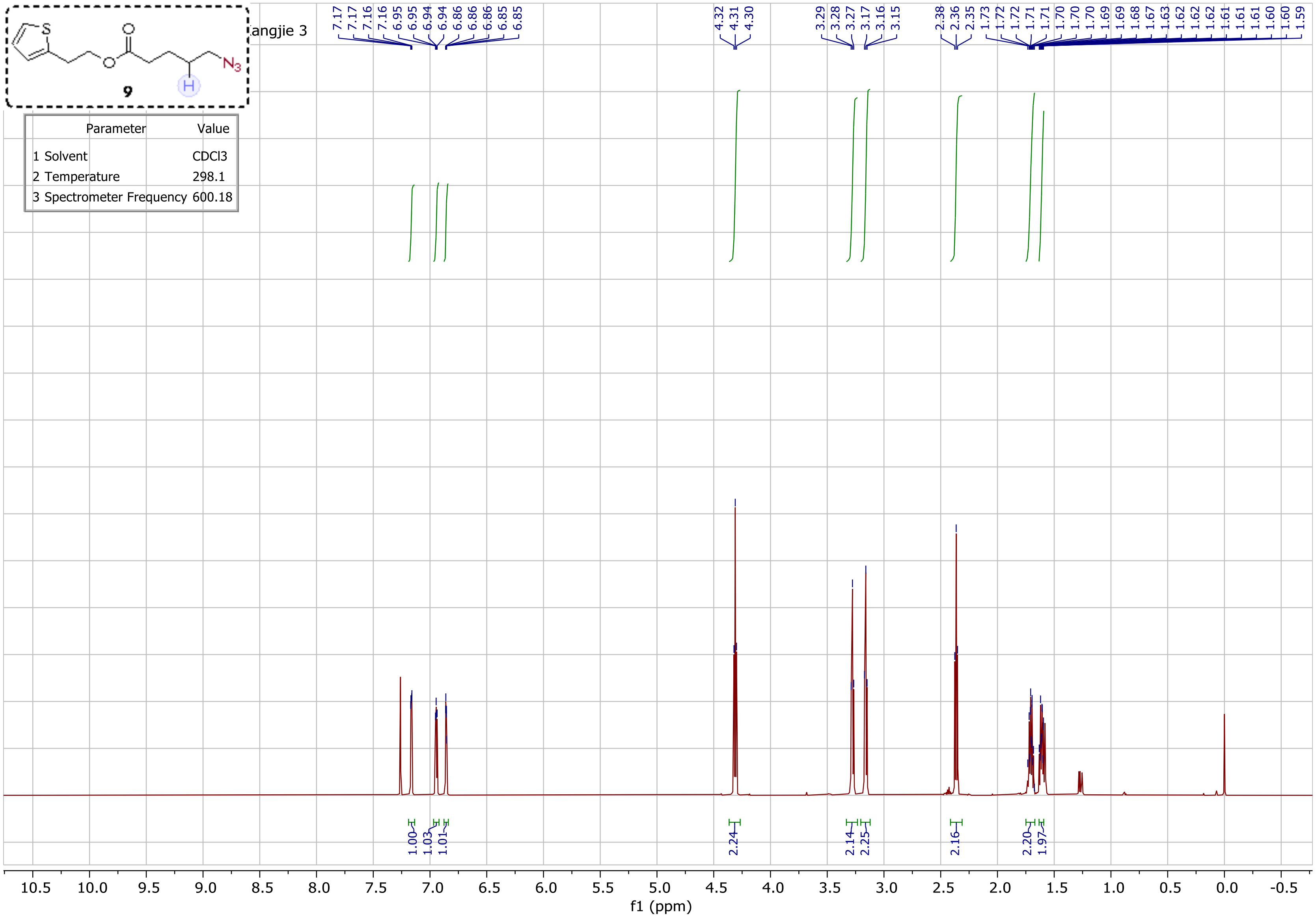

Parameter	Value
1 Solvent	CDCl3
2 Temperature	298.1
3 Spectrometer Frequency	600.18

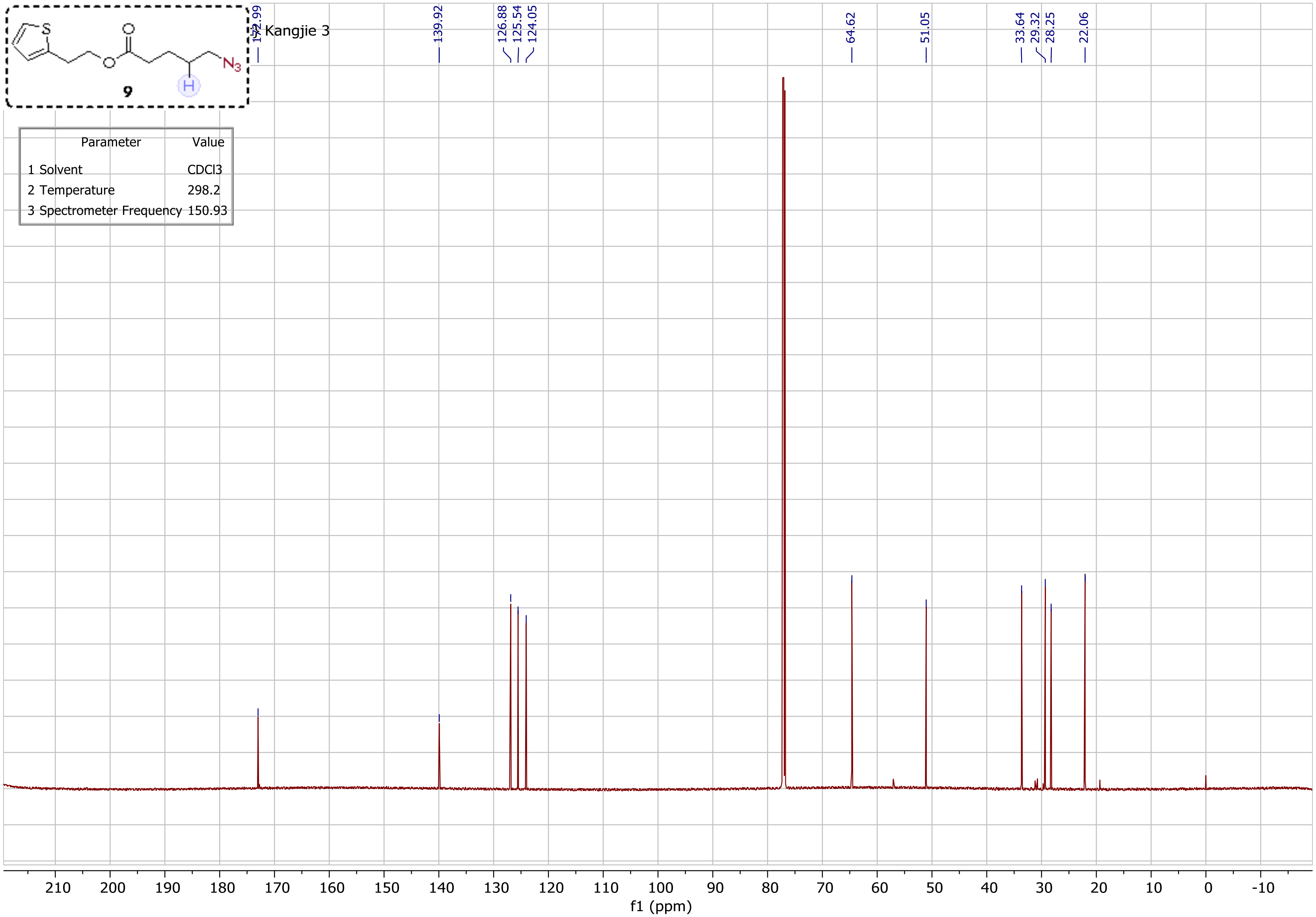
172.99
Kangjie 3
139.92
126.88
125.54
124.05
64.62
51.05
33.64
29.32
28.25
22.06
S
O
O
H
N3
9
Parameter Value
1 Solvent CDCl3
2 Temperature 298.2
3 Spectrometer Frequency 150.93
210 200 190 180 170 160 150 140 130 120 110 100 90 80 70 60 50 40 30 20 10 0 -10
f1 (ppm)

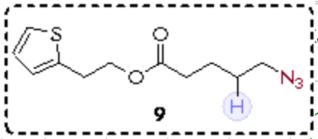

Parameter	Value
1 Solvent	CDCl3
2 Temperature	298.1
3 Spectrometer Frequency	600.18

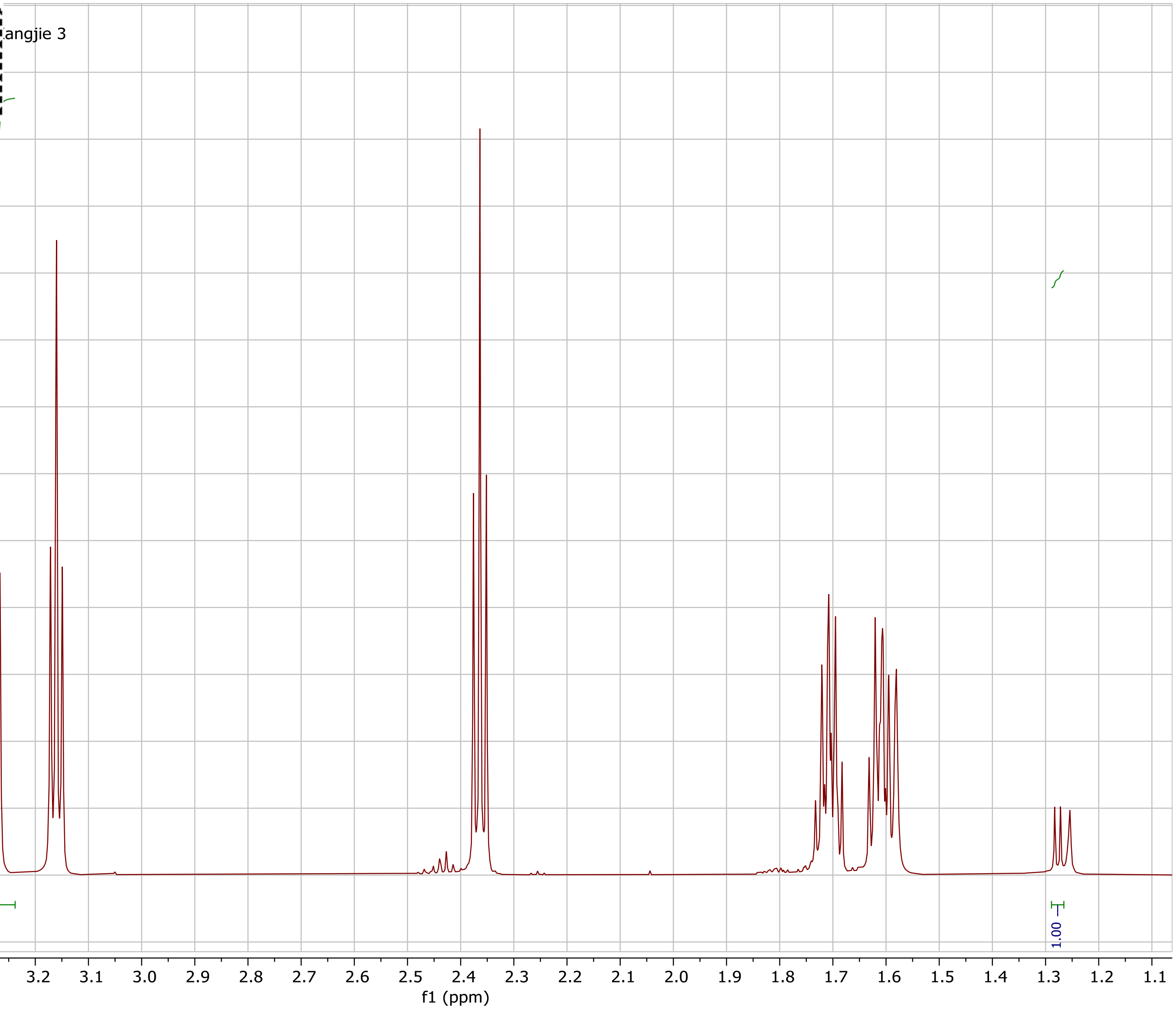

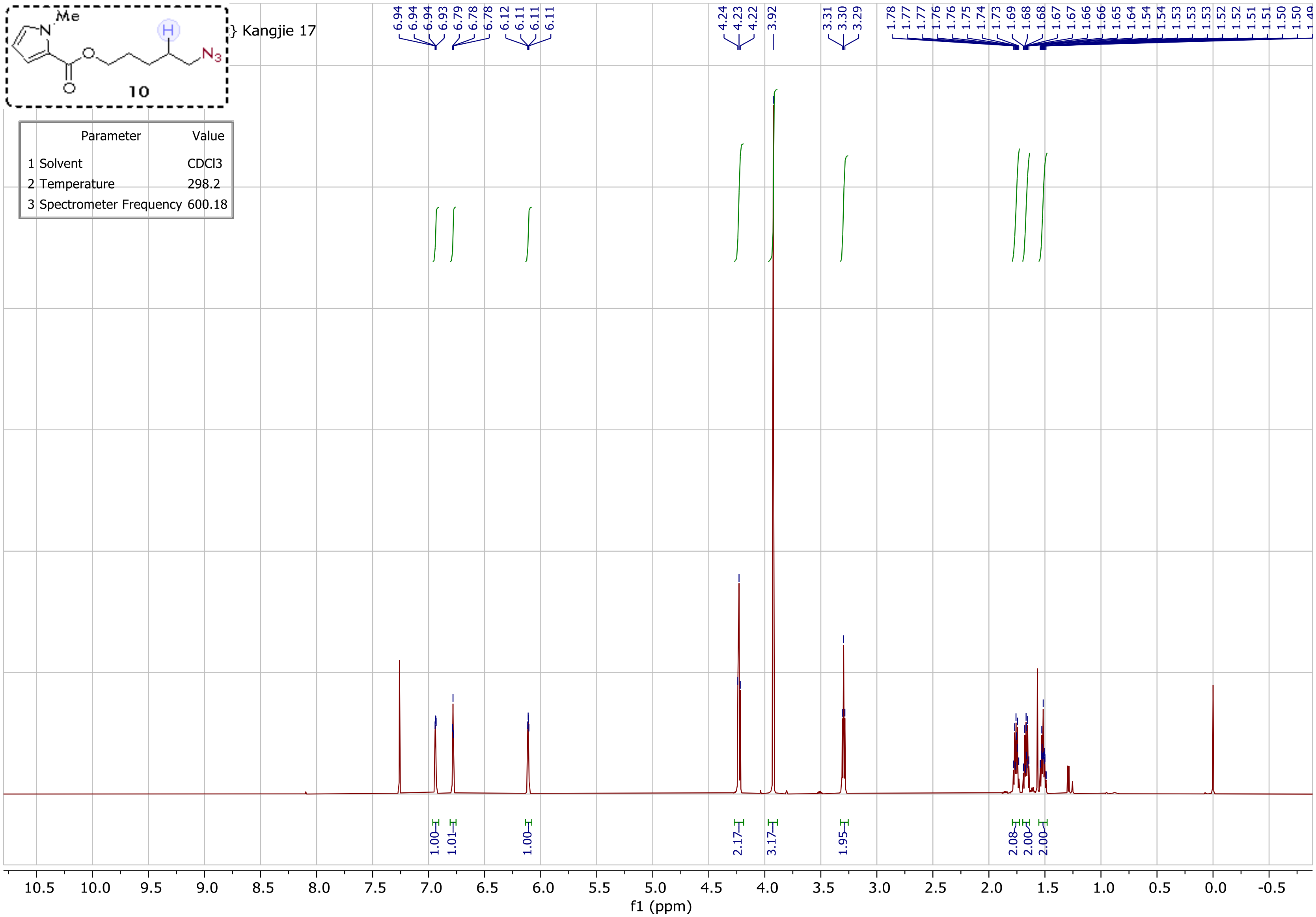
Me
N
O
O
N3
10
} Kangjie 17
Parameter Value
1 Solvent CDCl3
2 Temperature 298.2
3 Spectrometer Frequency 600.18
6.94 6.94 6.94 6.93 6.79 6.78 6.78 6.12 6.11 6.11 6.11
4.24 4.23 4.22 3.92 3.31 3.30 3.29
1.78 1.77 1.77 1.76 1.76 1.75 1.74 1.73 1.69 1.68 1.68 1.67 1.67 1.66 1.65 1.64 1.54 1.54 1.53 1.53 1.53 1.52 1.52 1.51 1.51 1.50 1.50
1.00 1.01 1.00 2.17 3.17 1.95 2.08 2.00 2.00
10.5 10.0 9.5 9.0 8.5 8.0 7.5 7.0 6.5 6.0 5.5 5.0 4.5 4.0 3.5 3.0 2.5 2.0 1.5 1.0 0.5 0.0 -0.5
f1 (ppm)

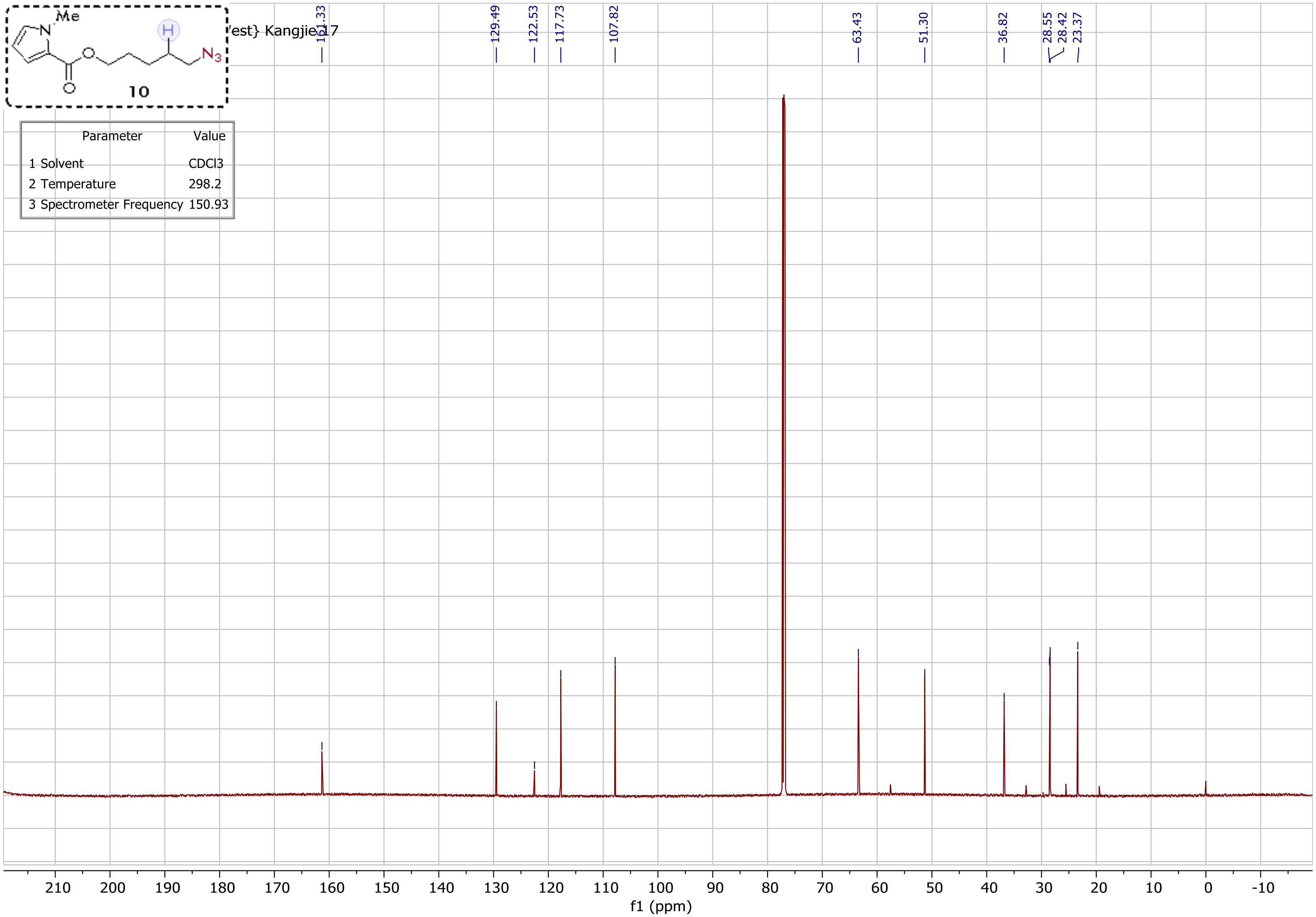

Me
N
H
N3
10
est} Kangjie17
161.33
129.49
122.53
117.73
107.82
63.43
51.30
36.82
28.55
28.42
23.37
Parameter Value
1 Solvent CDCl3
2 Temperature 298.2
3 Spectrometer Frequency 150.93
210 200 190 180 170 160 150 140 130 120 110 100 90 80 70 60 50 40 30 20 10 0 -10
f1 (ppm)

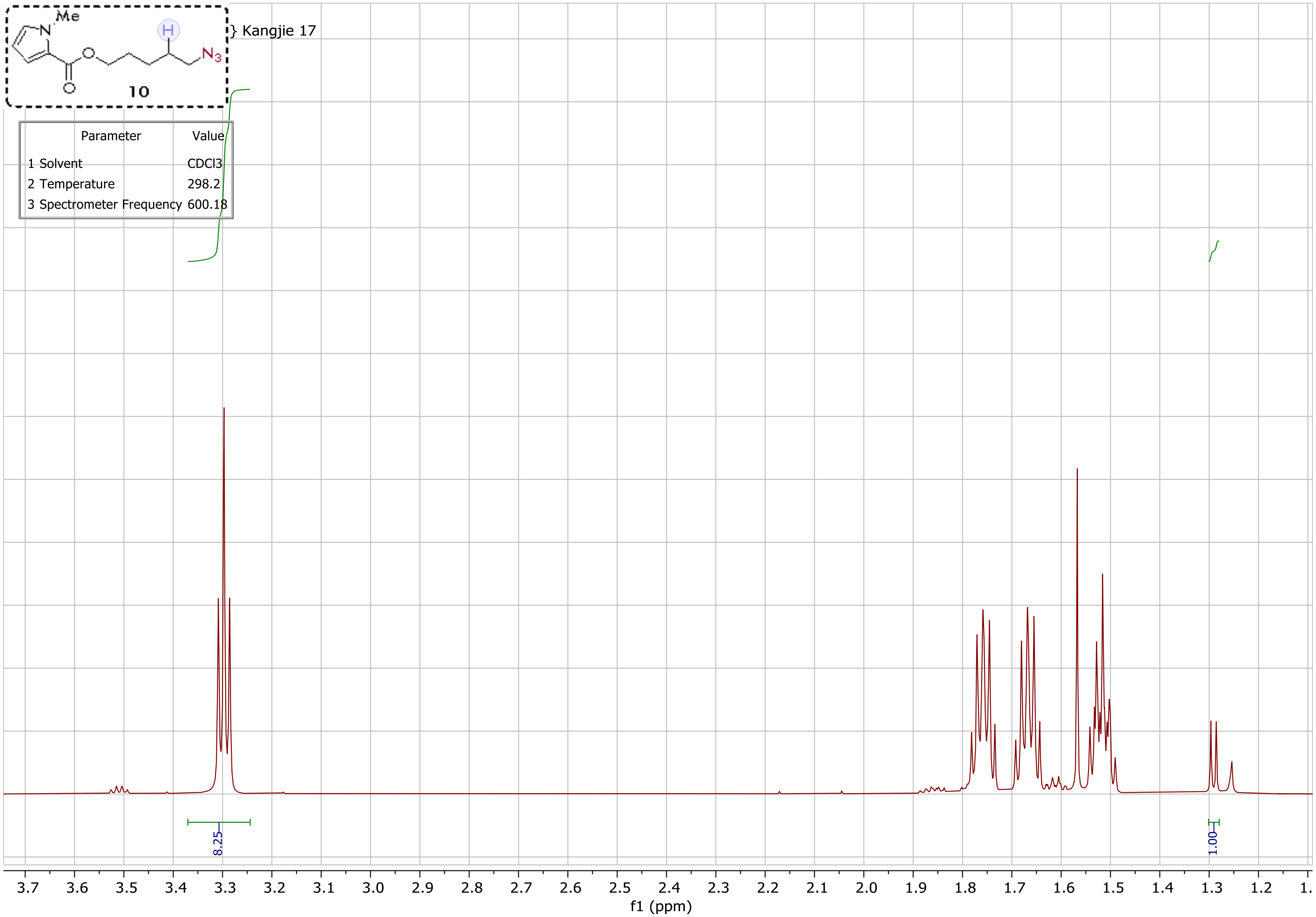
Me
N
O
O
H
N3
10
} Kangjie 17
Parameter Value
1 Solvent CDCl3
2 Temperature 298.2
3 Spectrometer Frequency 600.18
8.25
1.00
3.7 3.6 3.5 3.4 3.3 3.2 3.1 3.0 2.9 2.8 2.7 2.6 2.5 2.4 2.3 2.2 2.1 2.0 1.9 1.8 1.7 1.6 1.5 1.4 1.3 1.2
f1 (ppm)

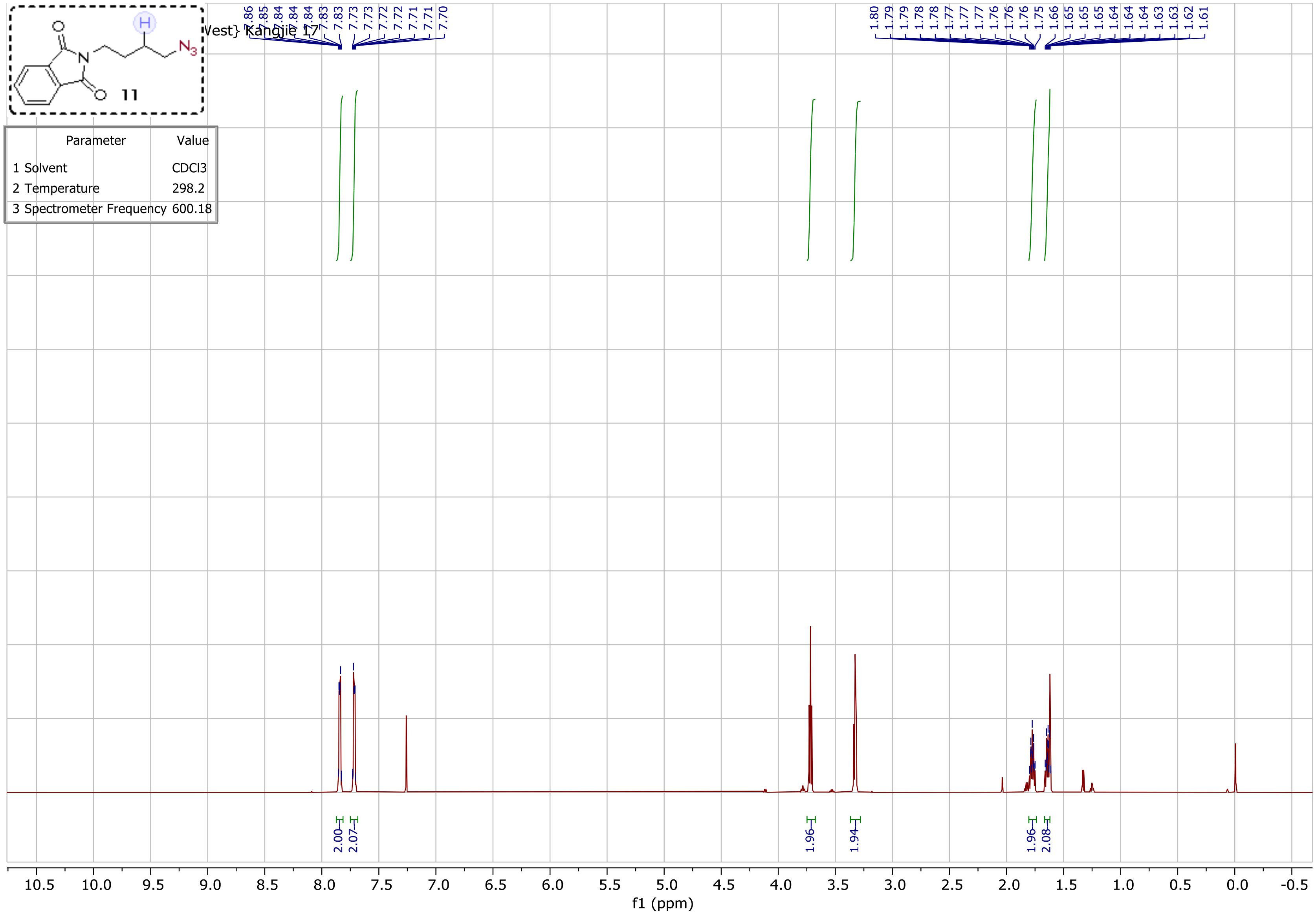

	Parameter	Value
1	Solvent	CDCl3
2	Temperature	298.2
3	Spectrometer Frequency	600.18

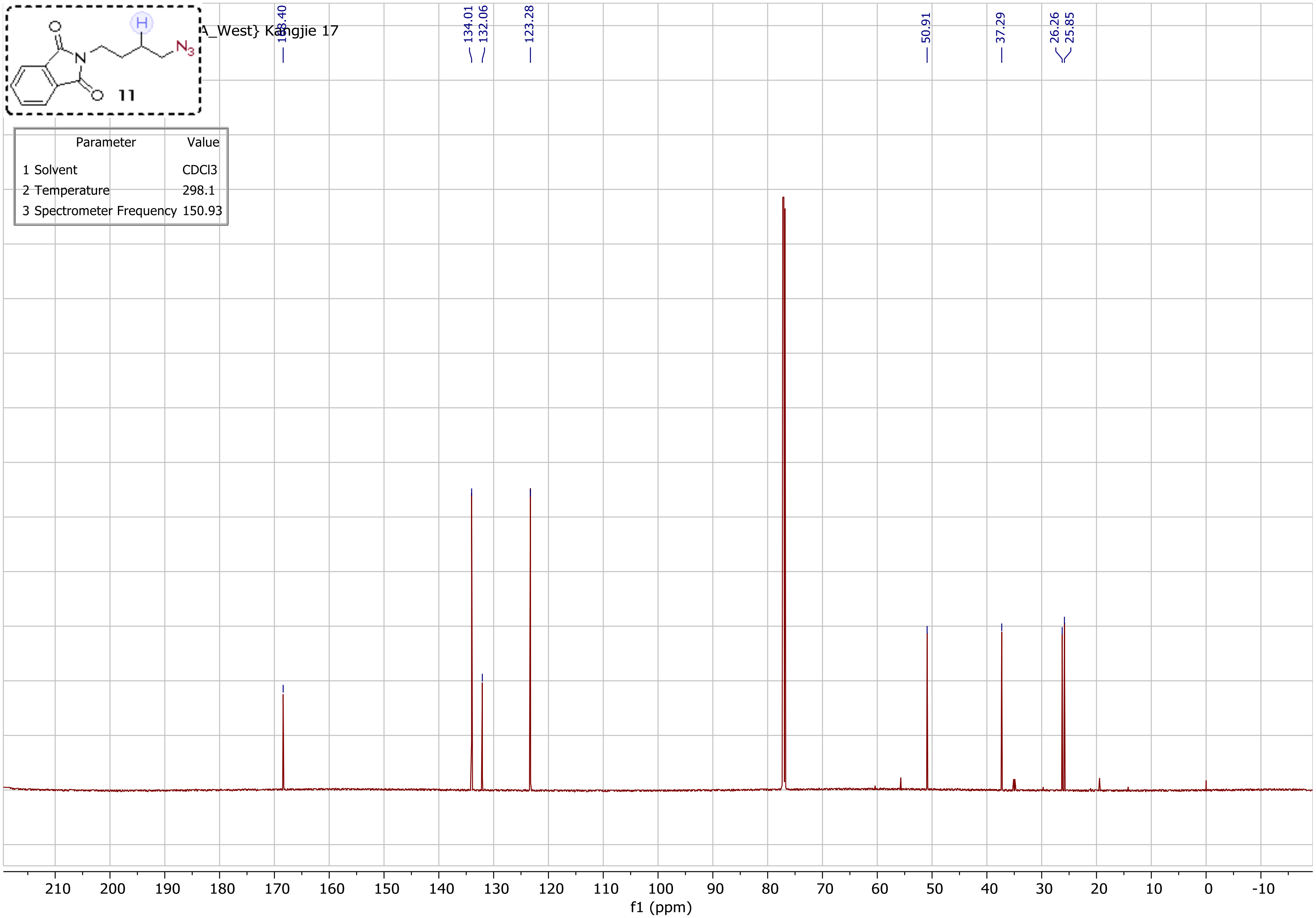
11
A_West} Kangjie 17
168.40
134.01
132.06
123.28
50.91
37.29
26.26
25.85
Parameter | Value
1 Solvent | CDCl3
2 Temperature | 298.1
3 Spectrometer Frequency | 150.93
210 200 190 180 170 160 150 140 130 120 110 100 90 80 70 60 50 40 30 20 10 0 -10
f1 (ppm)

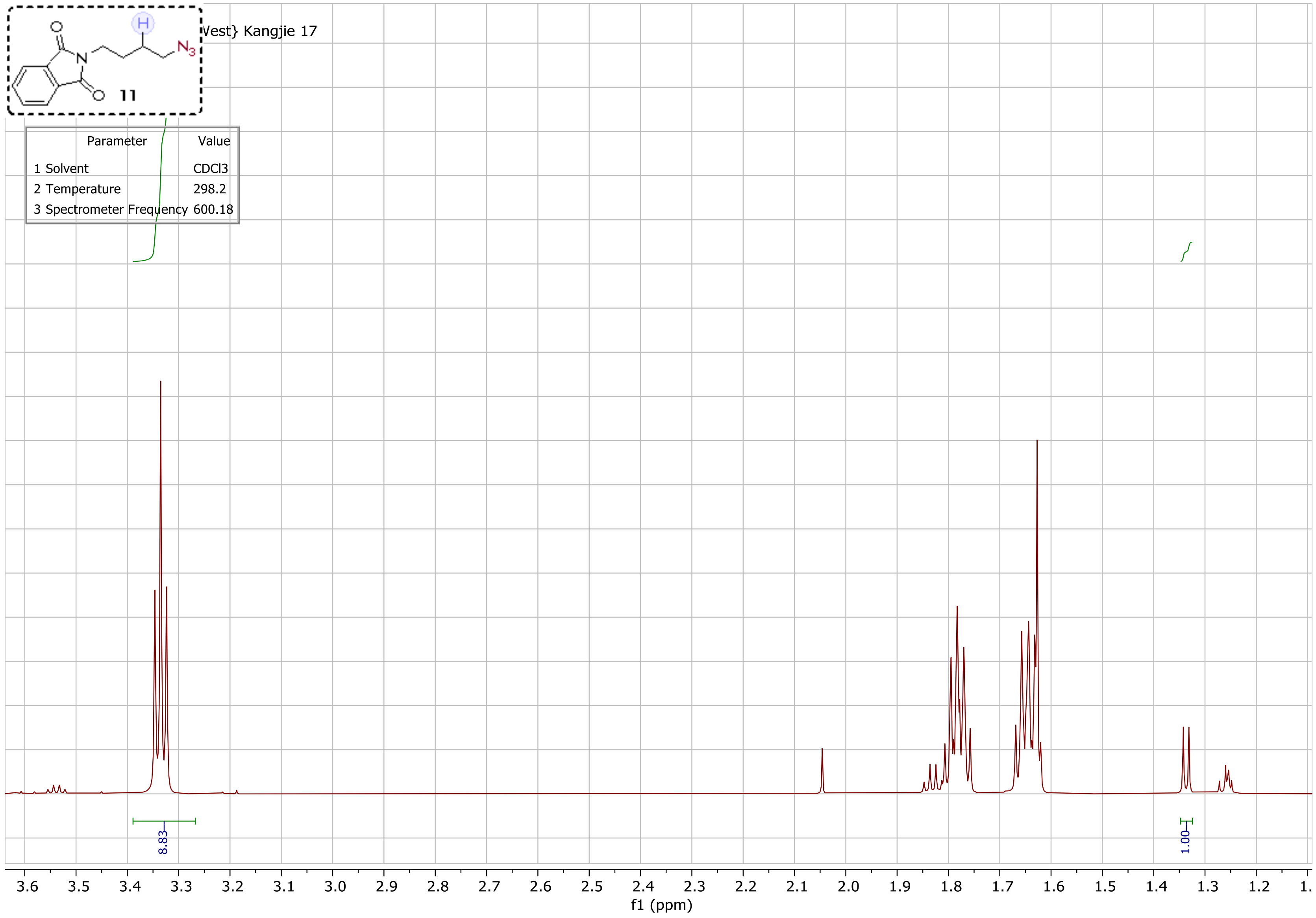

N
N3
11
Vest} Kangjie 17
Parameter
Value
1 Solvent
CDCl3
2 Temperature
298.2
3 Spectrometer Frequency
600.18
8.83
1.00
3.6
3.5
3.4
3.3
3.2
3.1
3.0
2.9
2.8
2.7
2.6
2.5
2.4
2.3
2.2
2.1
2.0
1.9
1.8
1.7
1.6
1.5
1.4
1.3
1.2
1.
f1 (ppm)

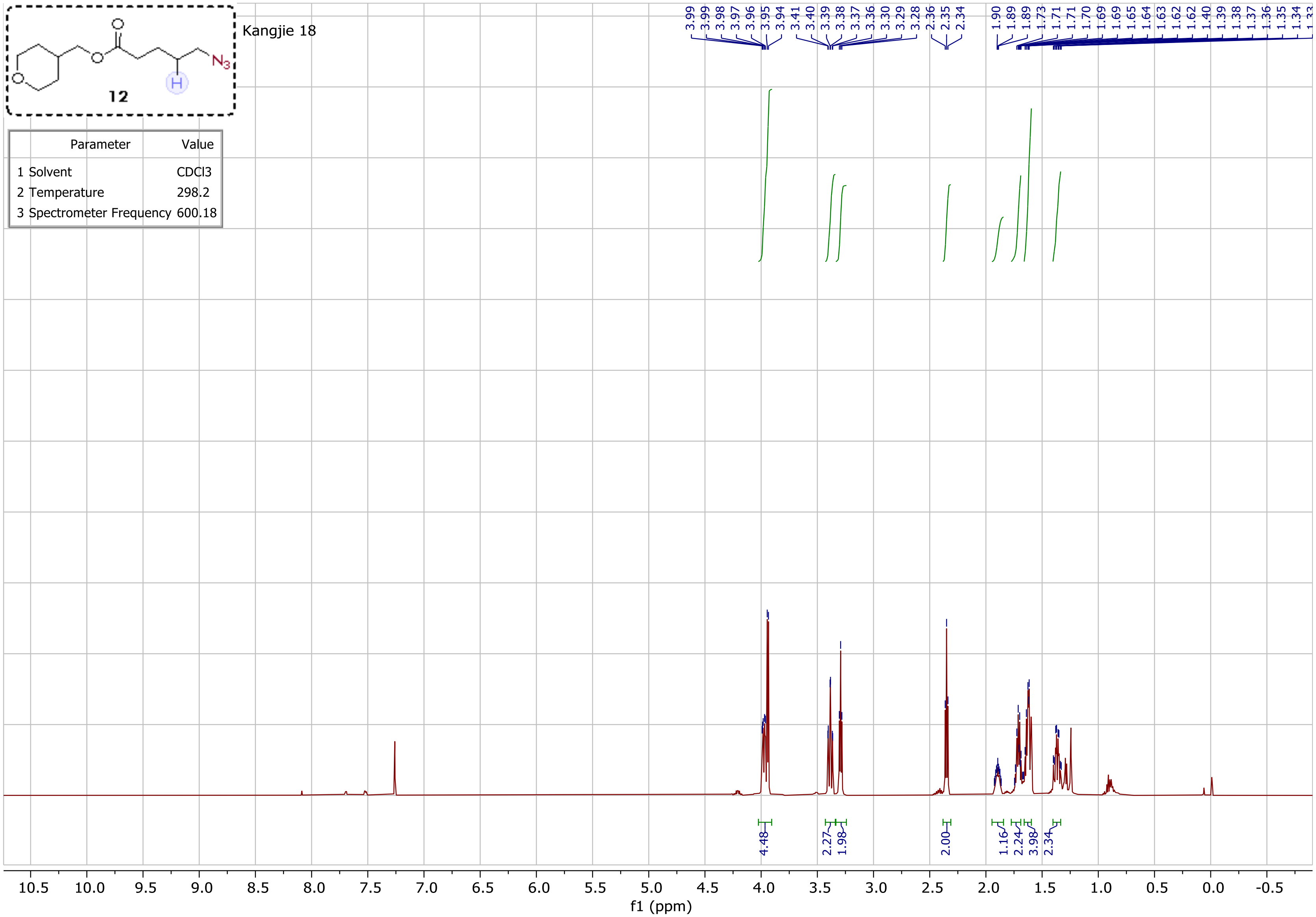
12
Kangjie 18
Parameter | Value
1 Solvent CDCl3
2 Temperature 298.2
3 Spectrometer Frequency 600.18
3.99 3.99 3.98 3.97 3.96 3.95 3.94 3.41 3.40 3.39 3.38 3.37 3.36 3.30 3.29 3.28 2.36 2.35 2.34 1.90 1.89 1.89 1.73 1.71 1.71 1.70 1.69 1.69 1.65 1.64 1.63 1.62 1.62 1.40 1.39 1.38 1.37 1.36 1.35 1.34
4.48 2.27 1.98 2.00 1.16 2.24 3.98 2.34
10.5 10.0 9.5 9.0 8.5 8.0 7.5 7.0 6.5 6.0 5.5 5.0 4.5 4.0 3.5 3.0 2.5 2.0 1.5 1.0 0.5 0.0 -0.5
f1 (ppm)

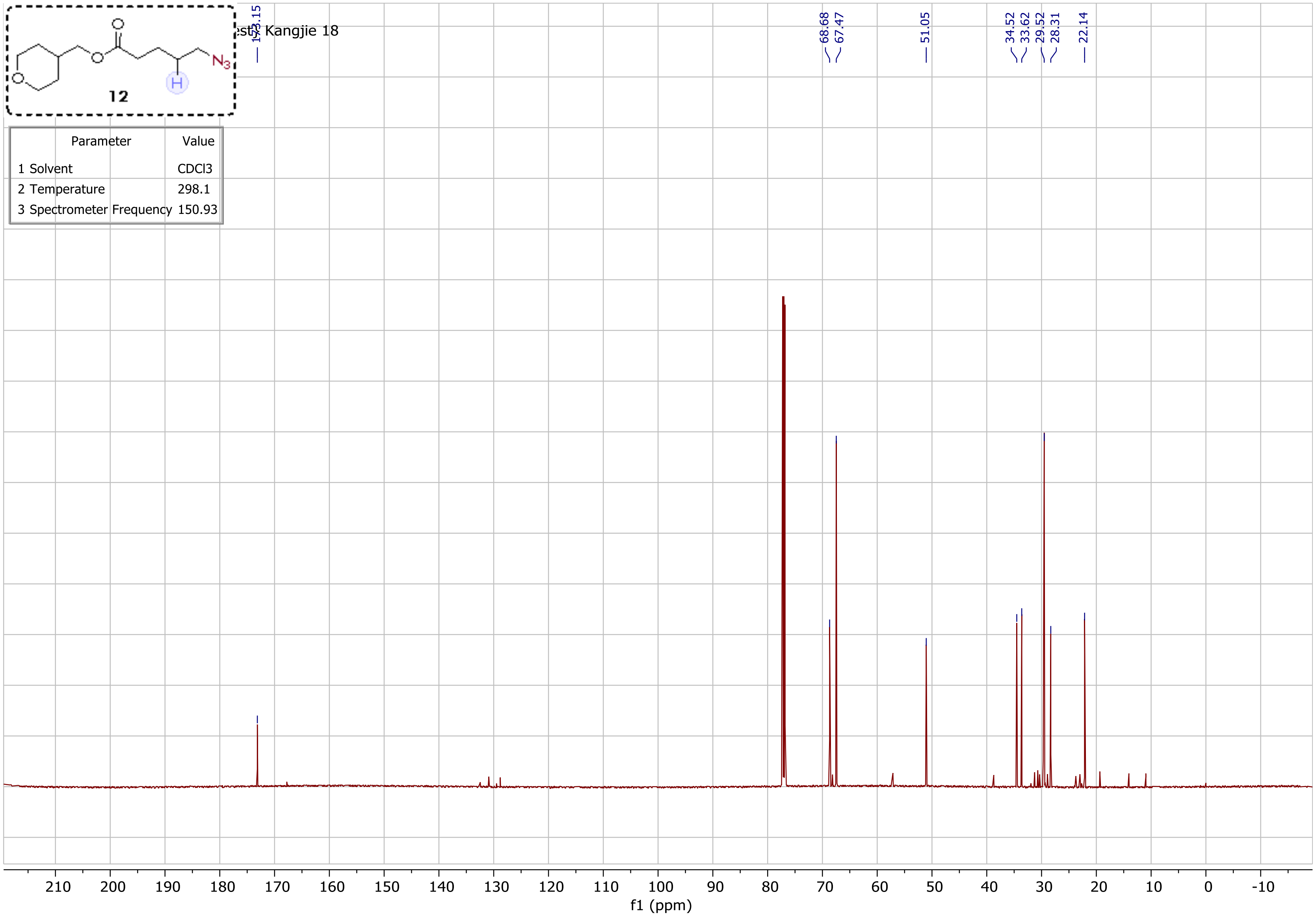

12
N3
H
est) Kangjie 18
173.15
68.68
67.47
51.05
34.52
33.62
29.52
28.31
22.14
Parameter Value
1 Solvent CDCl3
2 Temperature 298.1
3 Spectrometer Frequency 150.93
210 200 190 180 170 160 150 140 130 120 110 100 90 80 70 60 50 40 30 20 10 0 -10
f1 (ppm)

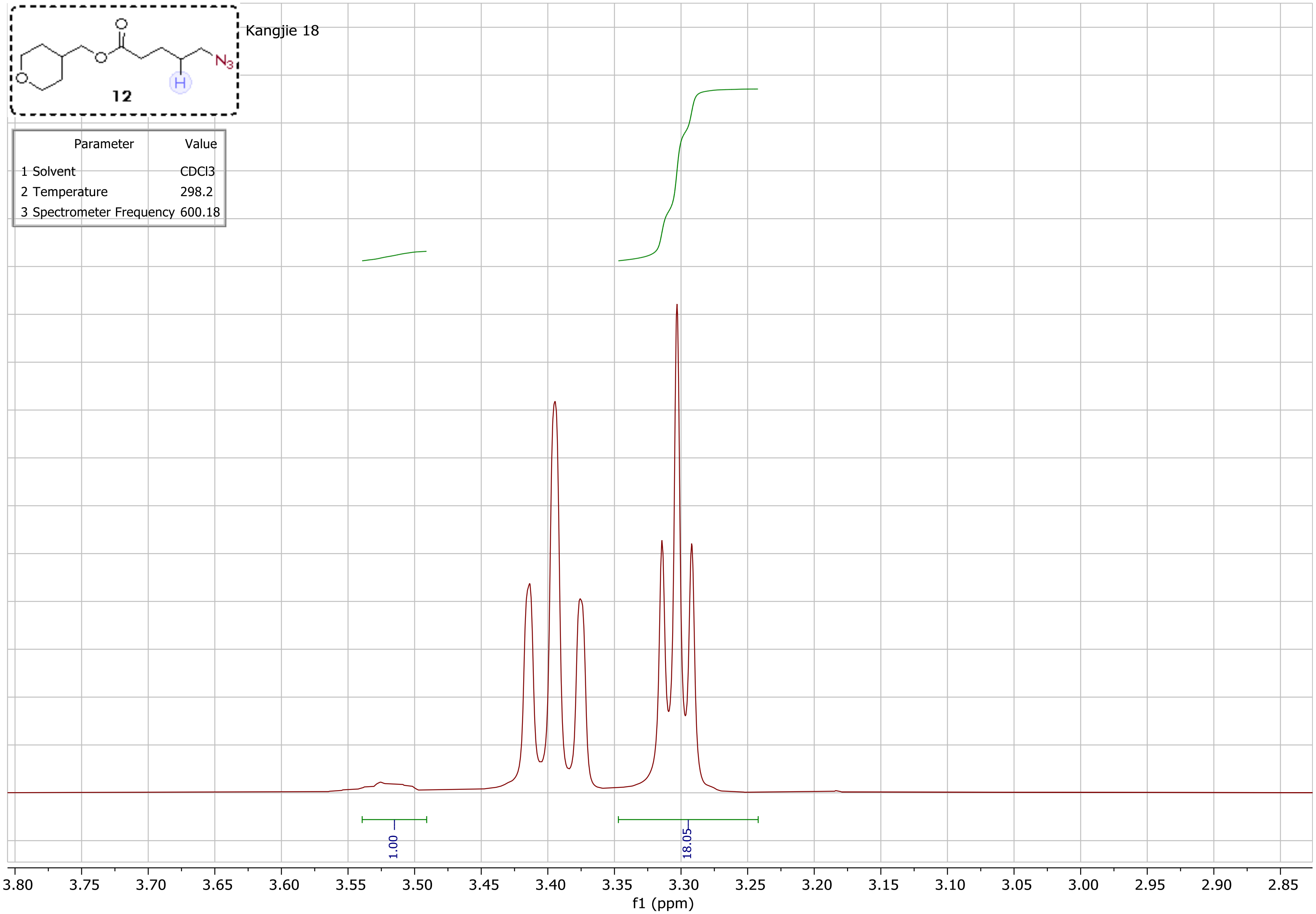

12
N3
Kangjie 18
Parameter Value
1 Solvent CDCl3
2 Temperature 298.2
3 Spectrometer Frequency 600.18
1.00
18.05
3.80 3.75 3.70 3.65 3.60 3.55 3.50 3.45 3.40 3.35 3.30 3.25 3.20 3.15 3.10 3.05 3.00 2.95 2.90 2.85
f1 (ppm)

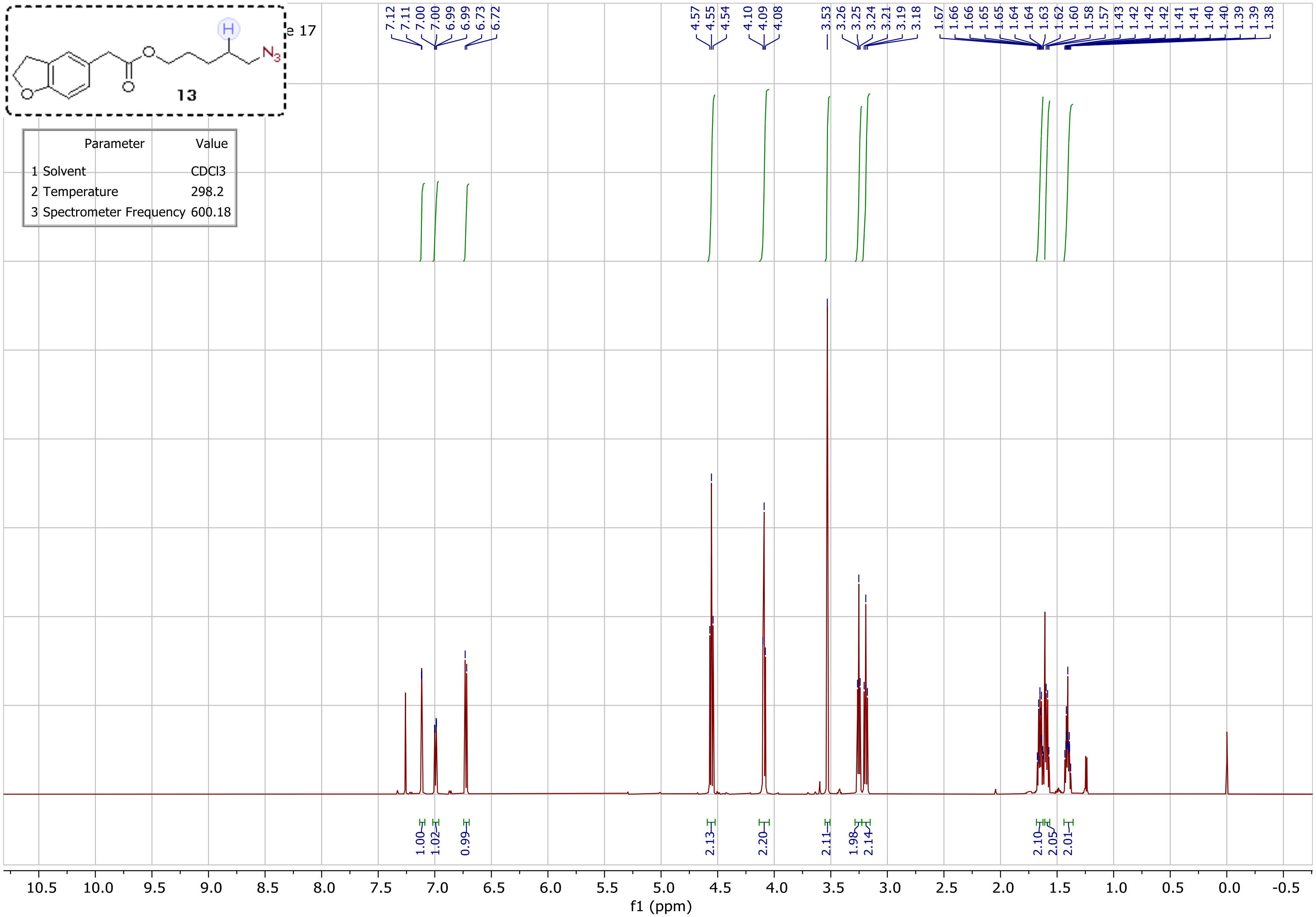

	Parameter	Value
1	Solvent	CDCl3
2	Temperature	298.2
3	Spectrometer Frequency	600.18

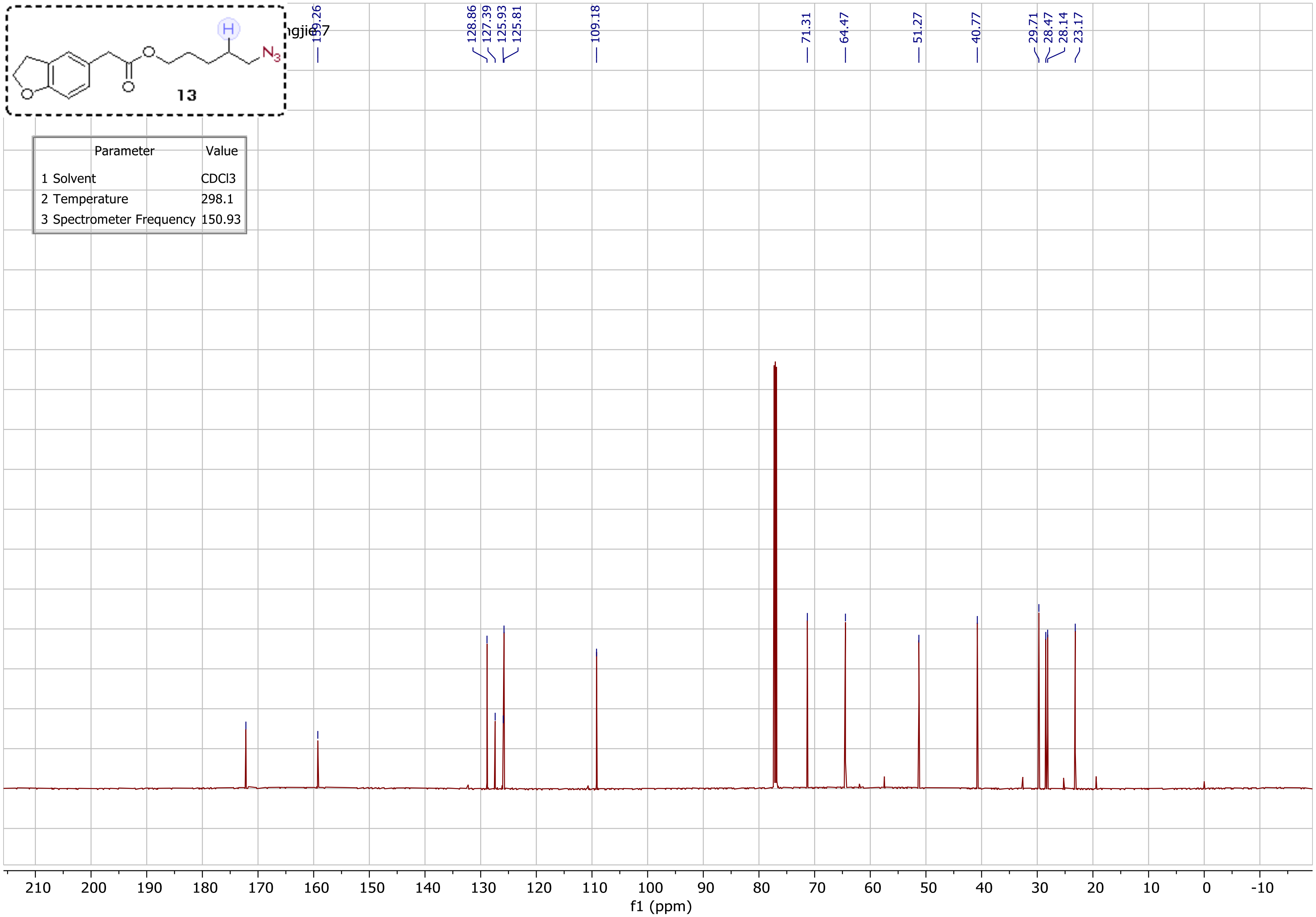
13
N3
Parameter Value
1 Solvent CDCl3
2 Temperature 298.1
3 Spectrometer Frequency 150.93
128.86
127.39
125.93
125.81
109.18
71.31
64.47
51.27
40.77
29.71
28.47
28.14
23.17
210 200 190 180 170 160 150 140 130 120 110 100 90 80 70 60 50 40 30 20 10 0 -10
f1 (ppm)

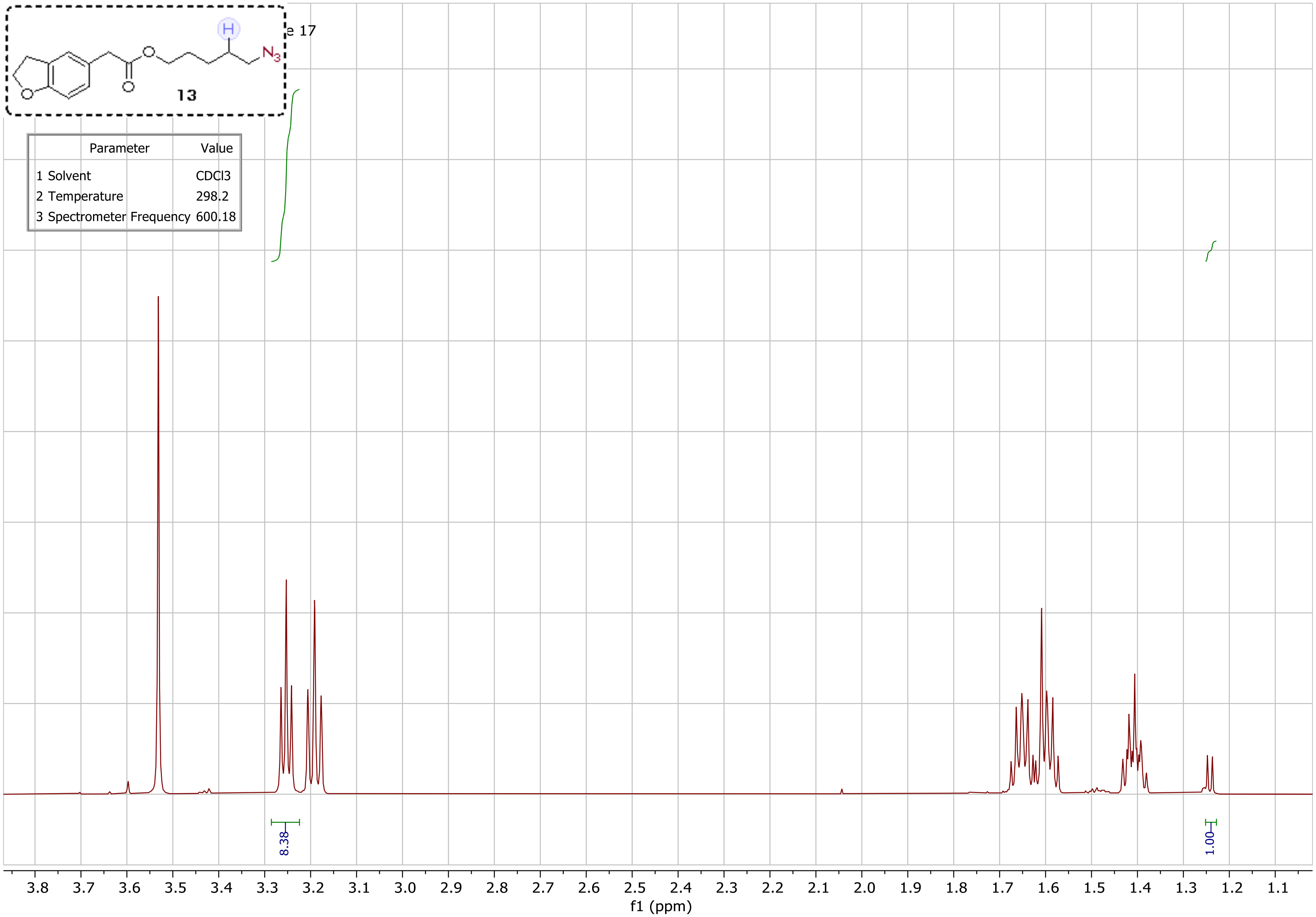

Parameter	Value
1 Solvent	CDCl3
2 Temperature	298.2
3 Spectrometer Frequency	600.18

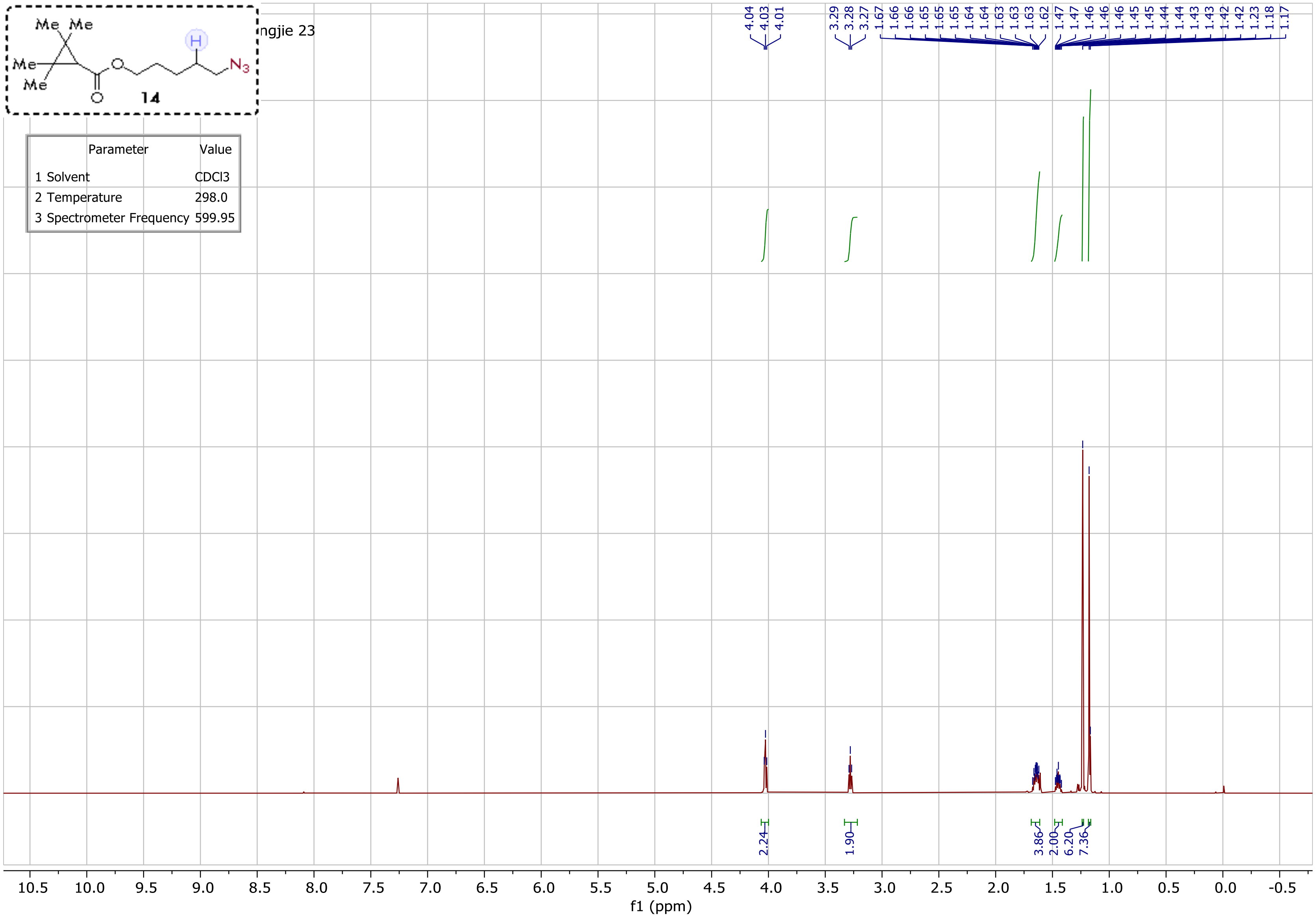

	Parameter	Value
1	Solvent	CDCl3
2	Temperature	298.0
3	Spectrometer Frequency	599.95

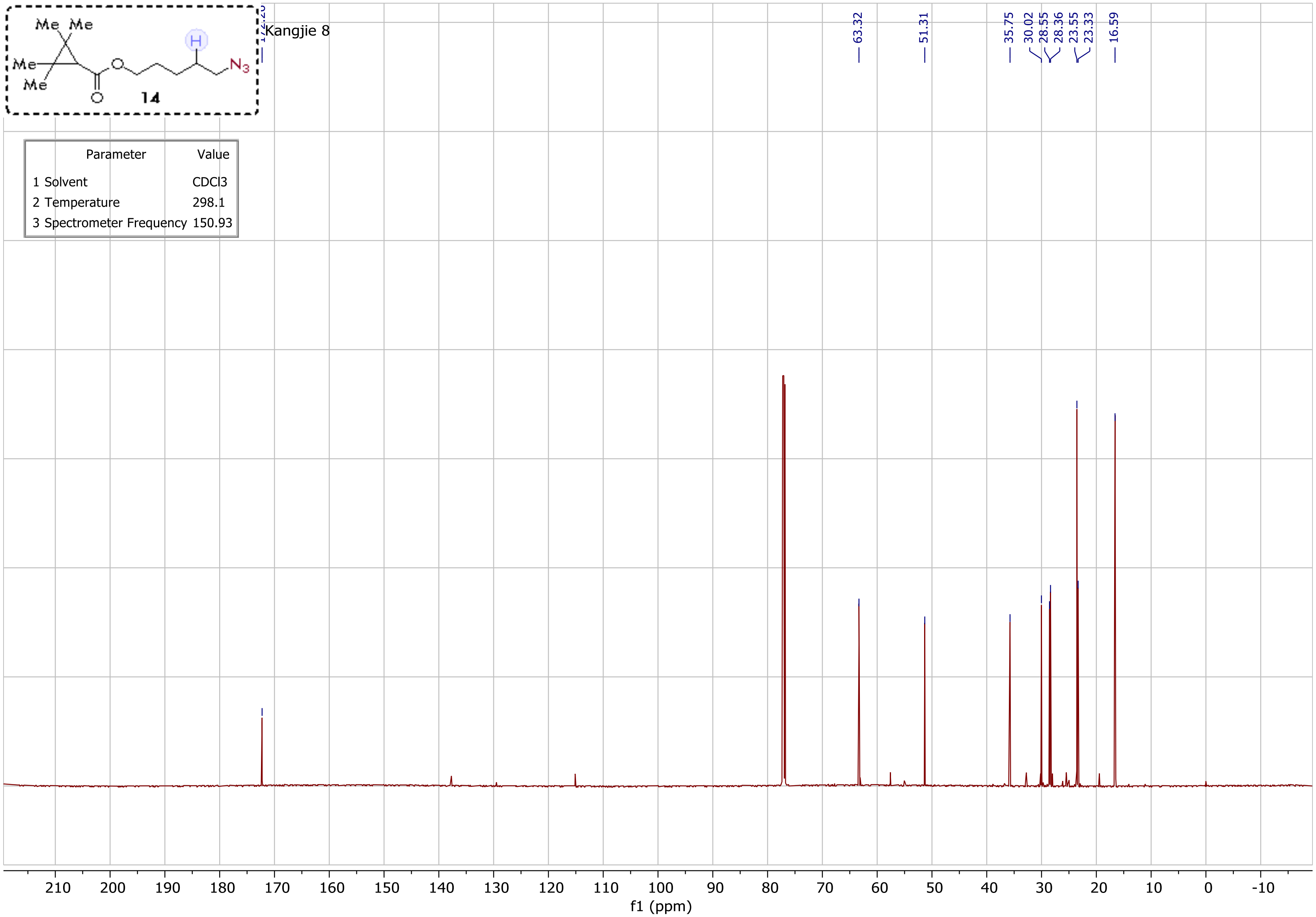
Me Me
Me
Me
O
O
H
N_3
14
Kangjie 8
63.32
51.31
35.75
30.02
28.55
28.36
23.55
23.33
16.59
Parameter Value
1 Solvent CDCl3
2 Temperature 298.1
3 Spectrometer Frequency 150.93
210 200 190 180 170 160 150 140 130 120 110 100 90 80 70 60 50 40 30 20 10 0 -10
f1 (ppm)

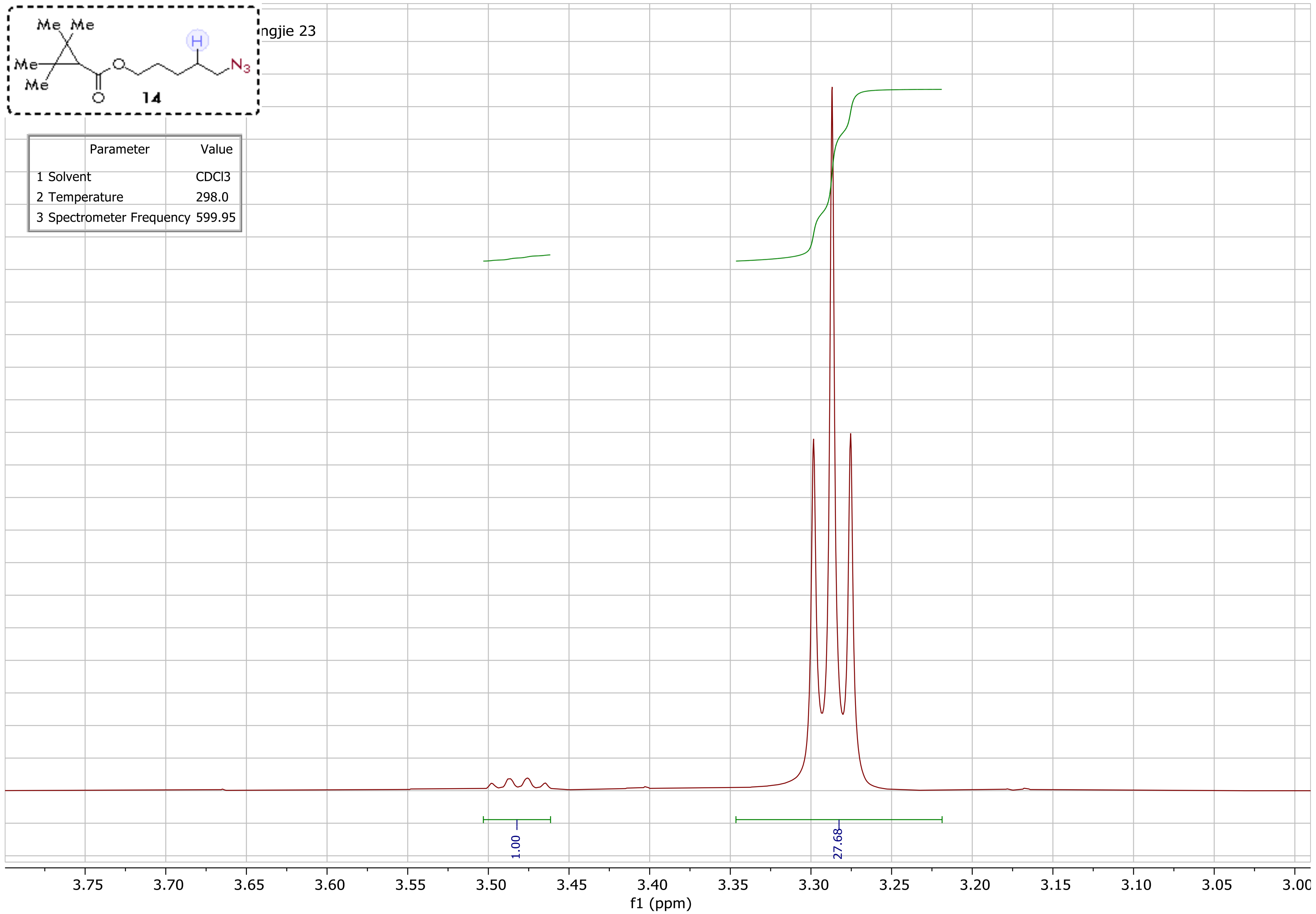

Me Me
Me
Me
H
N3
14
ngjie 23
Parameter Value
1 Solvent CDCl3
2 Temperature 298.0
3 Spectrometer Frequency 599.95
1.00
27.68
3.75 3.70 3.65 3.60 3.55 3.50 3.45 3.40 3.35 3.30 3.25 3.20 3.15 3.10 3.05 3.00
f1 (ppm)

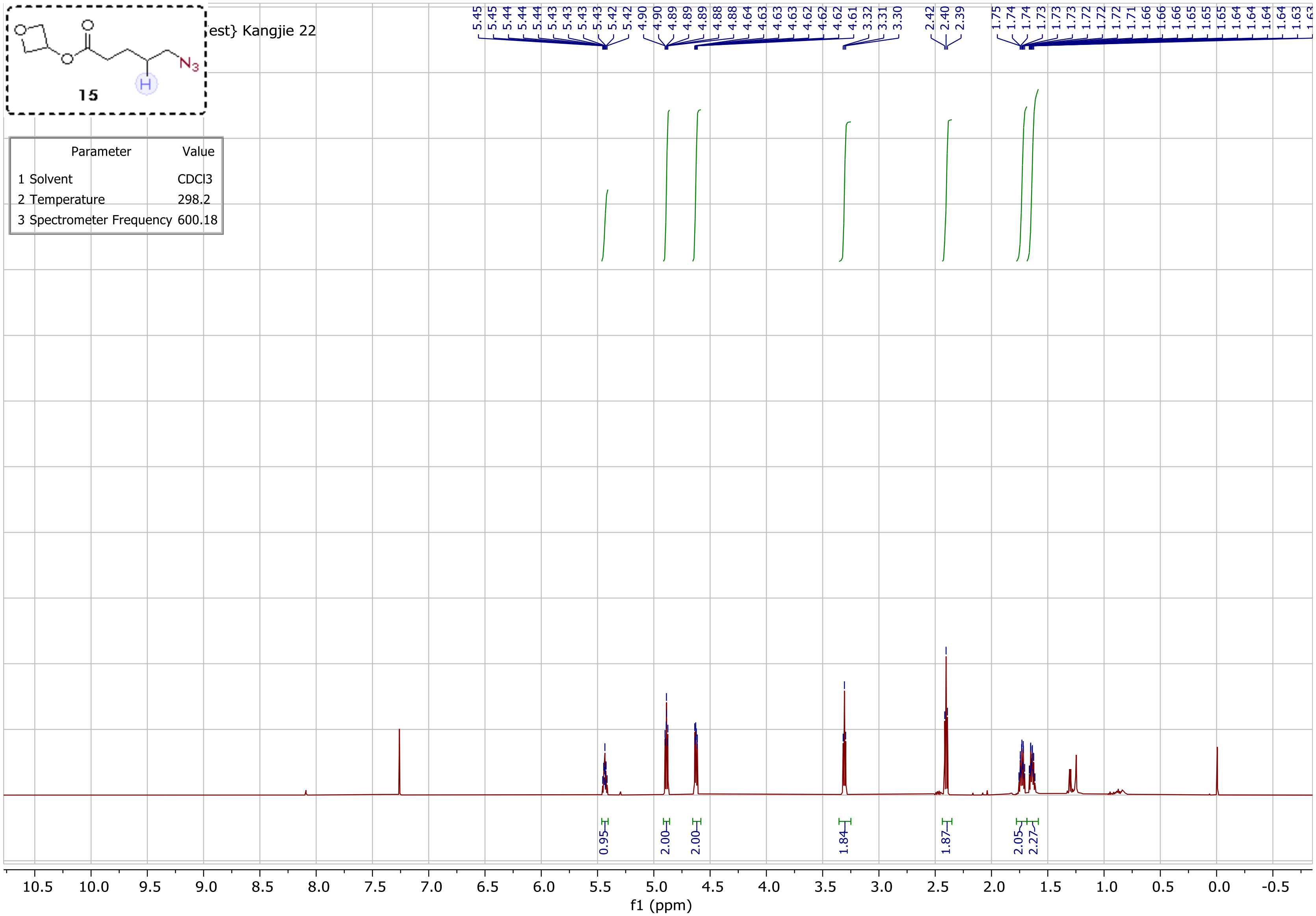
15
N3
H
est} Kangjie 22
Parameter Value
1 Solvent CDCl3
2 Temperature 298.2
3 Spectrometer Frequency 600.18
5.45 5.45 5.44 5.44 5.43 5.43 5.43 5.43 5.42 5.42 4.90 4.89 4.89 4.89 4.88 4.88 4.64 4.63 4.63 4.62 4.62 4.62 4.61 3.32 3.31 3.30 2.42 2.40 2.39 1.75 1.74 1.74 1.73 1.73 1.73 1.72 1.72 1.71 1.66 1.66 1.65 1.65 1.65 1.64 1.64 1.64 1.63
0.95 2.00 2.00 1.84 1.87 2.05 2.27
10.5 10.0 9.5 9.0 8.5 8.0 7.5 7.0 6.5 6.0 5.5 5.0 4.5 4.0 3.5 3.0 2.5 2.0 1.5 1.0 0.5 0.0 -0.5
f1 (ppm)

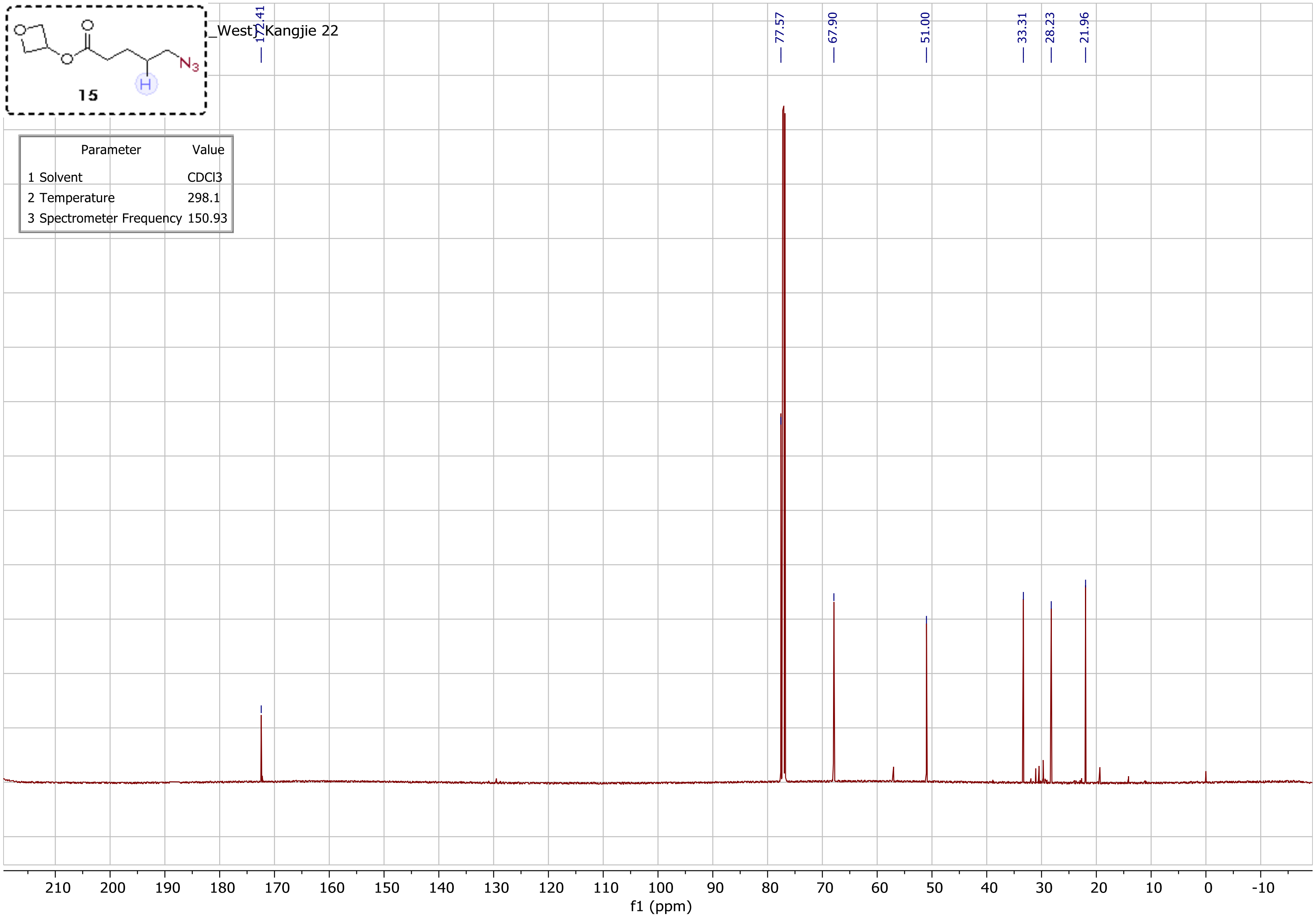
15
_West] Kangjie 22
172.41
77.57
67.90
51.00
33.31
28.23
21.96
Parameter Value
1 Solvent CDCl3
2 Temperature 298.1
3 Spectrometer Frequency 150.93
210 200 190 180 170 160 150 140 130 120 110 100 90 80 70 60 50 40 30 20 10 0 -10
f1 (ppm)

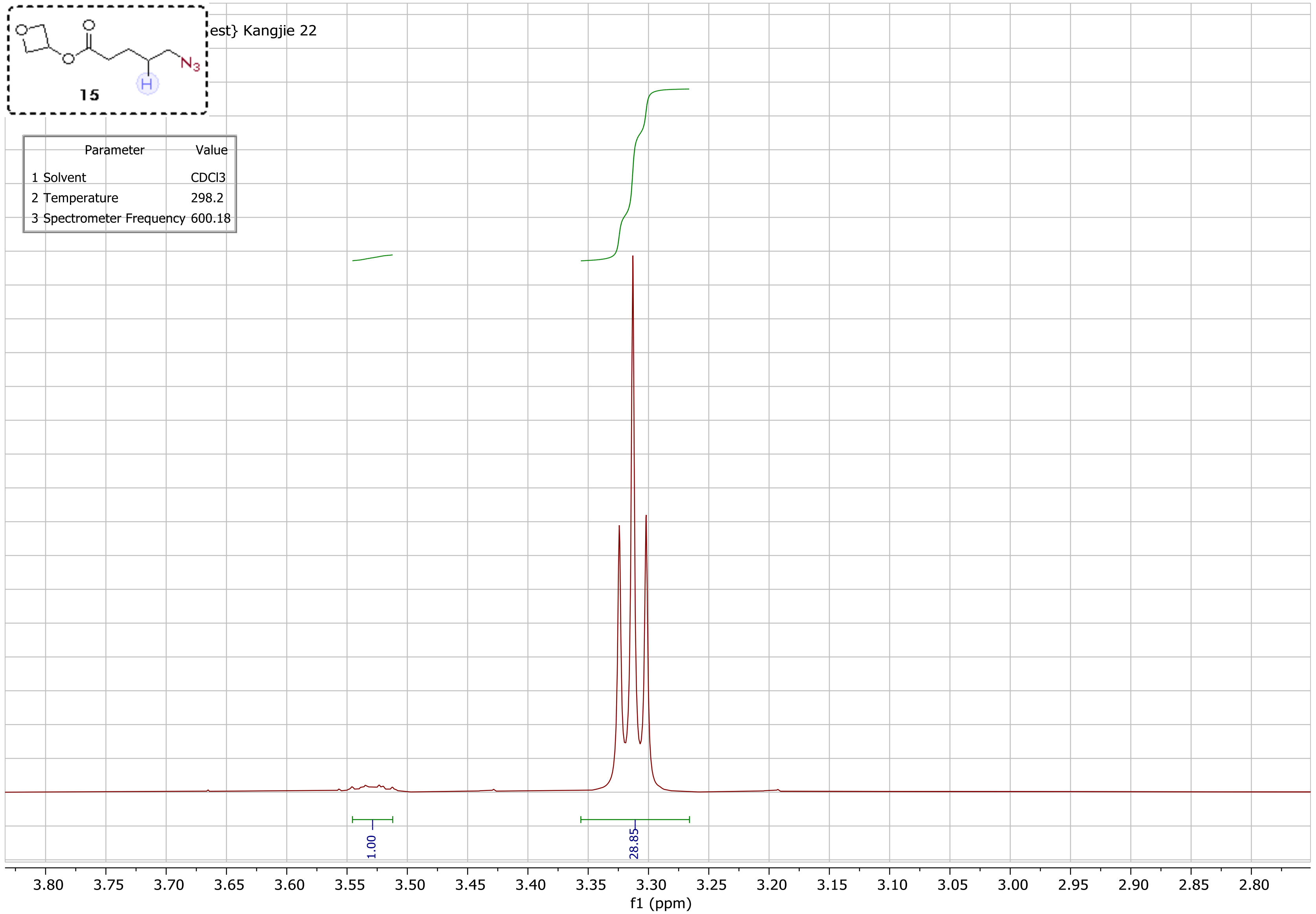
15
est} Kangjie 22
Parameter Value
1 Solvent CDCl3
2 Temperature 298.2
3 Spectrometer Frequency 600.18
1.00
28.85
3.80 3.75 3.70 3.65 3.60 3.55 3.50 3.45 3.40 3.35 3.30 3.25 3.20 3.15 3.10 3.05 3.00 2.95 2.90 2.85 2.80
f1 (ppm)

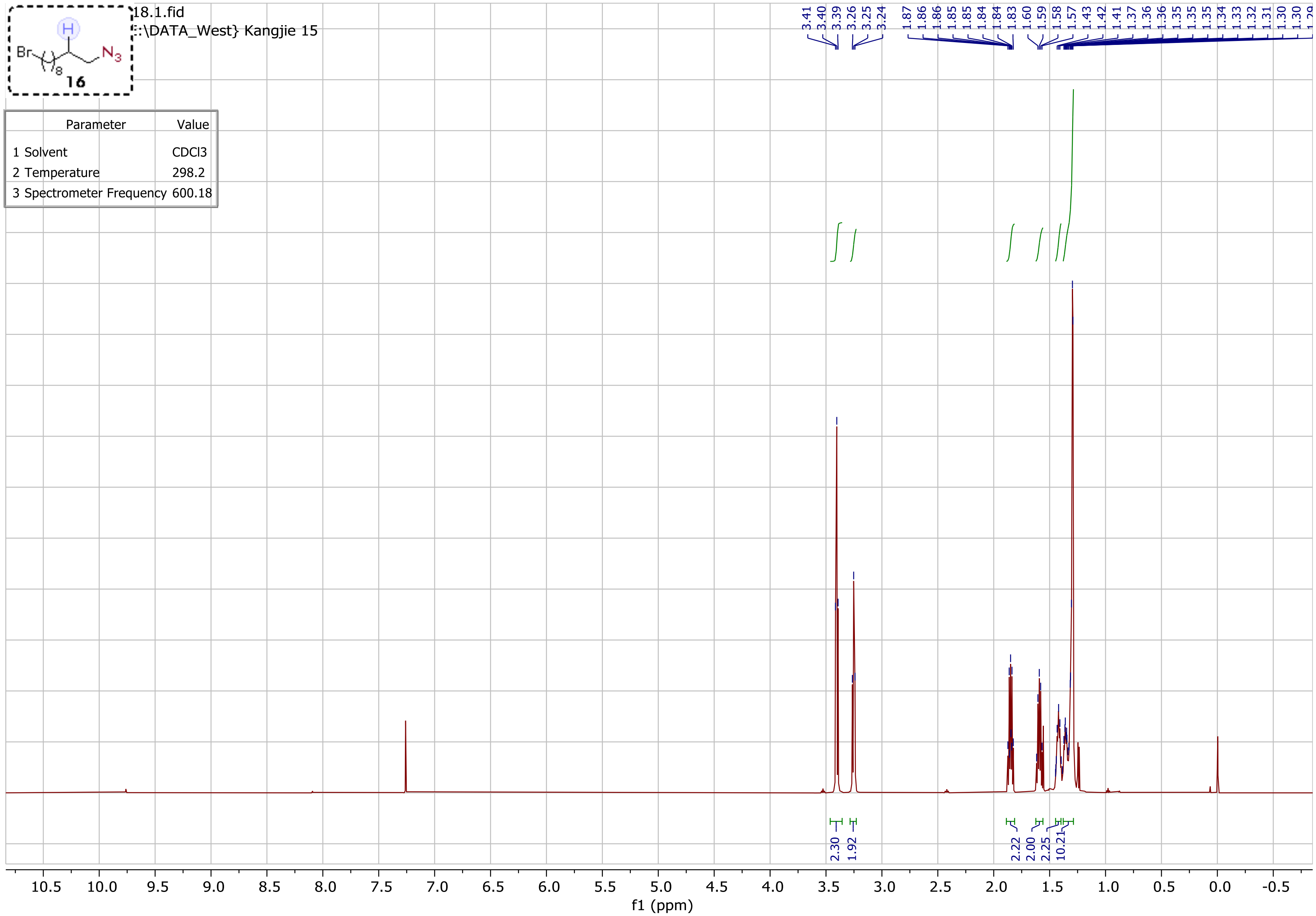

Parameter	Value
1 Solvent	CDCl3
2 Temperature	298.2
3 Spectrometer Frequency	600.18

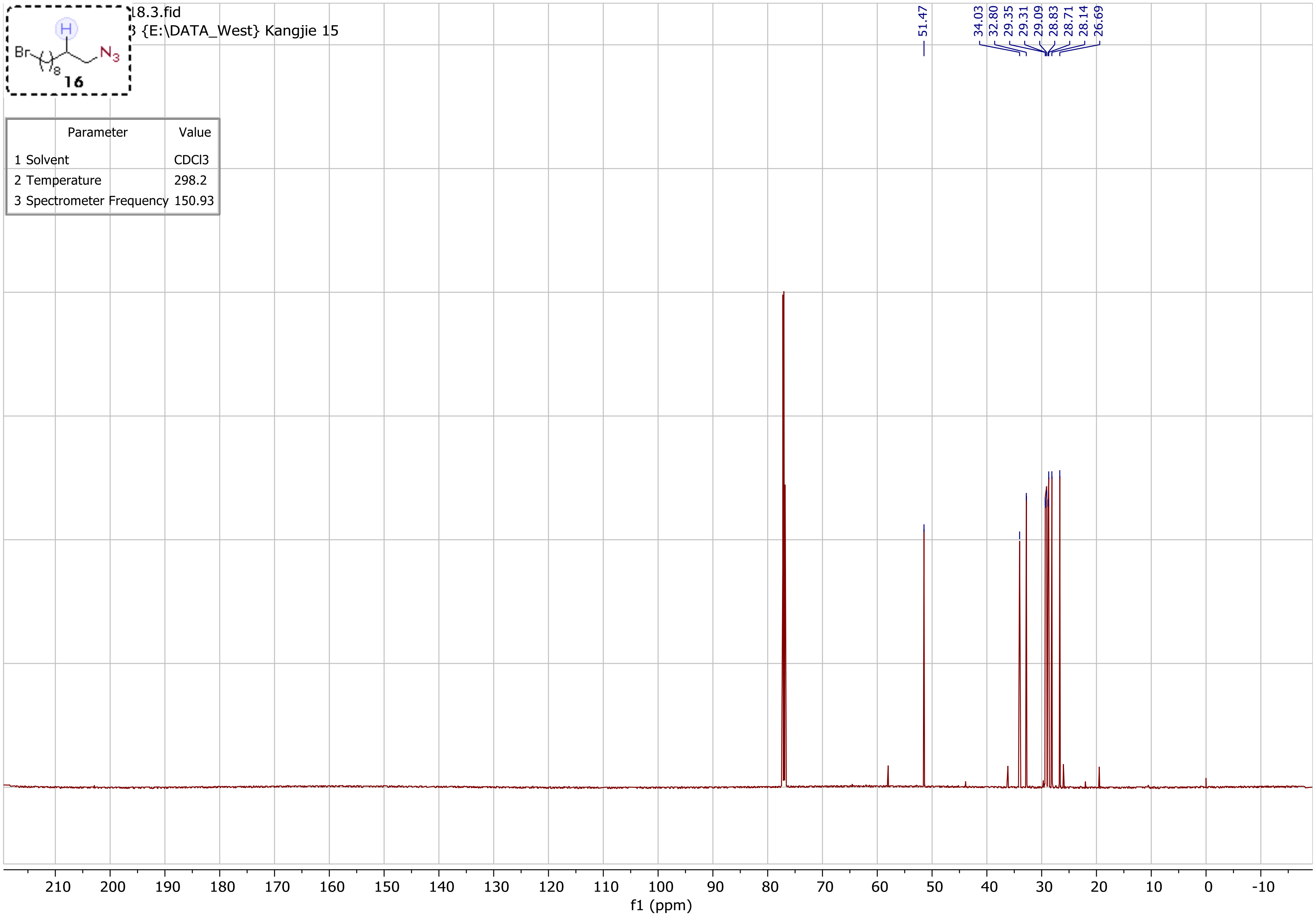

18.3.fid
3 {E:\DATA_West} Kangjie 15
Br
H
N3
8
16
Parameter | Value
1 Solvent | CDCl3
2 Temperature | 298.2
3 Spectrometer Frequency | 150.93
51.47
34.03
32.80
29.35
29.31
29.09
28.83
28.71
28.14
26.69
210 200 190 180 170 160 150 140 130 120 110 100 90 80 70 60 50 40 30 20 10 0 -10
f1 (ppm)

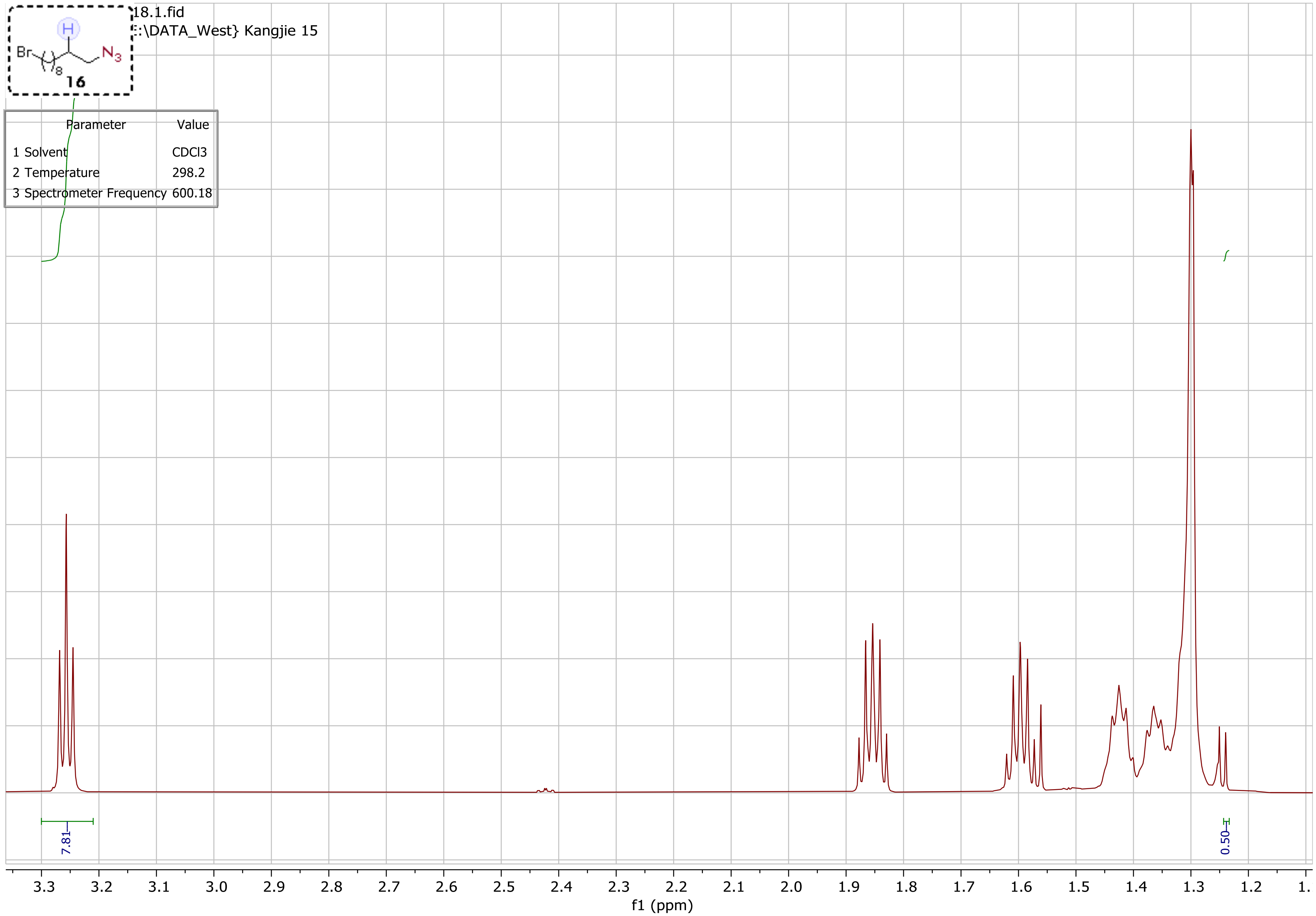

18.1.fid
:\DATA_West} Kangjie 15
Br
N3
8
16
Parameter Value
1 Solvent CDCl3
2 Temperature 298.2
3 Spectrometer Frequency 600.18
7.81
0.50
f1 (ppm)

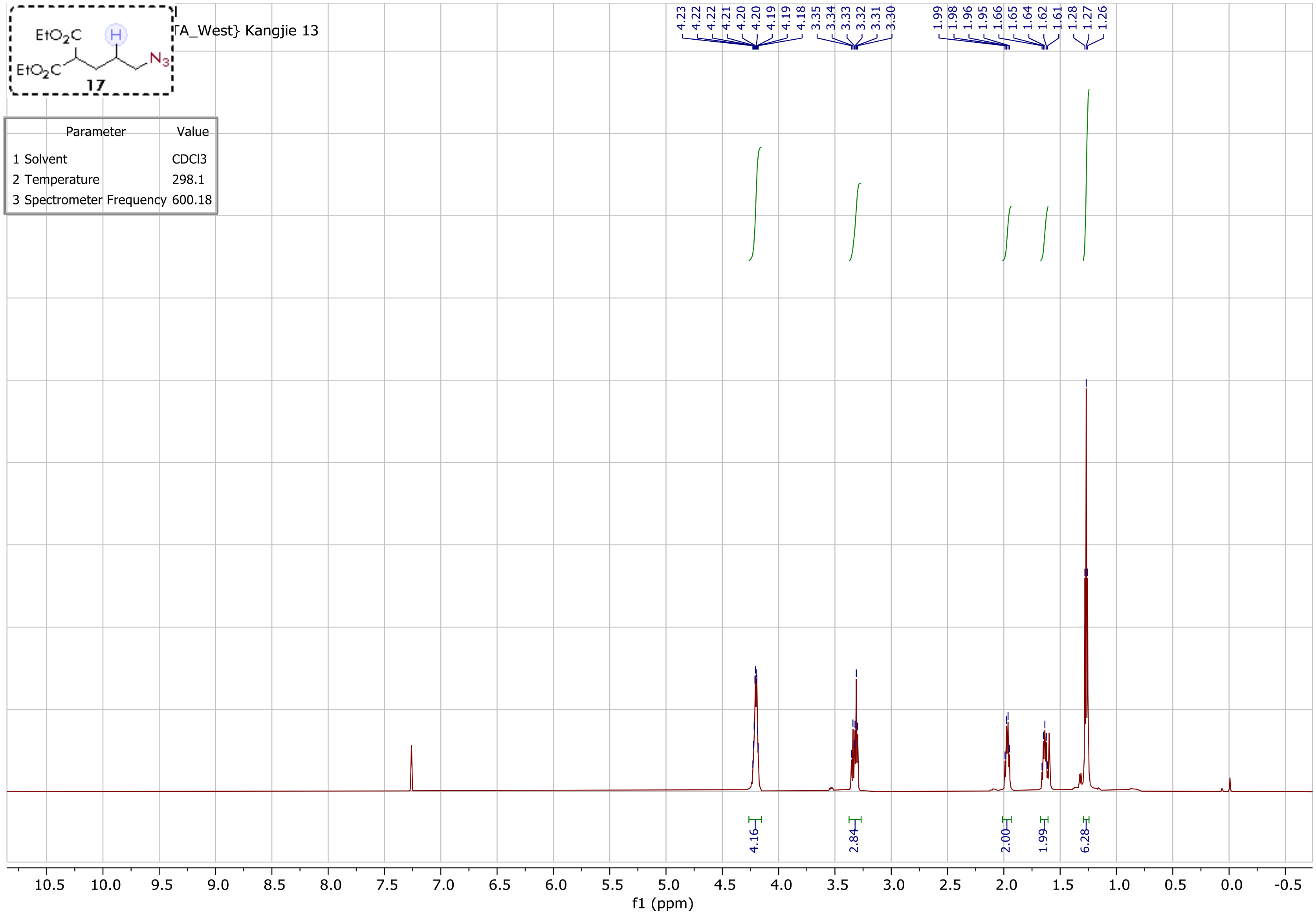
EtO2C
EtO2C
N3
17
[A_West} Kangjie 13
Parameter Value
1 Solvent CDCl3
2 Temperature 298.1
3 Spectrometer Frequency 600.18
4.23 4.22 4.22 4.21 4.20 4.20 4.19 4.19 4.18 3.35 3.34 3.33 3.32 3.31 3.30 1.99 1.98 1.96 1.95 1.66 1.65 1.64 1.62 1.61 1.28 1.27 1.26
4.16
2.84
2.00
1.99
6.28
10.5 10.0 9.5 9.0 8.5 8.0 7.5 7.0 6.5 6.0 5.5 5.0 4.5 4.0 3.5 3.0 2.5 2.0 1.5 1.0 0.5 0.0 -0.5
f1 (ppm)

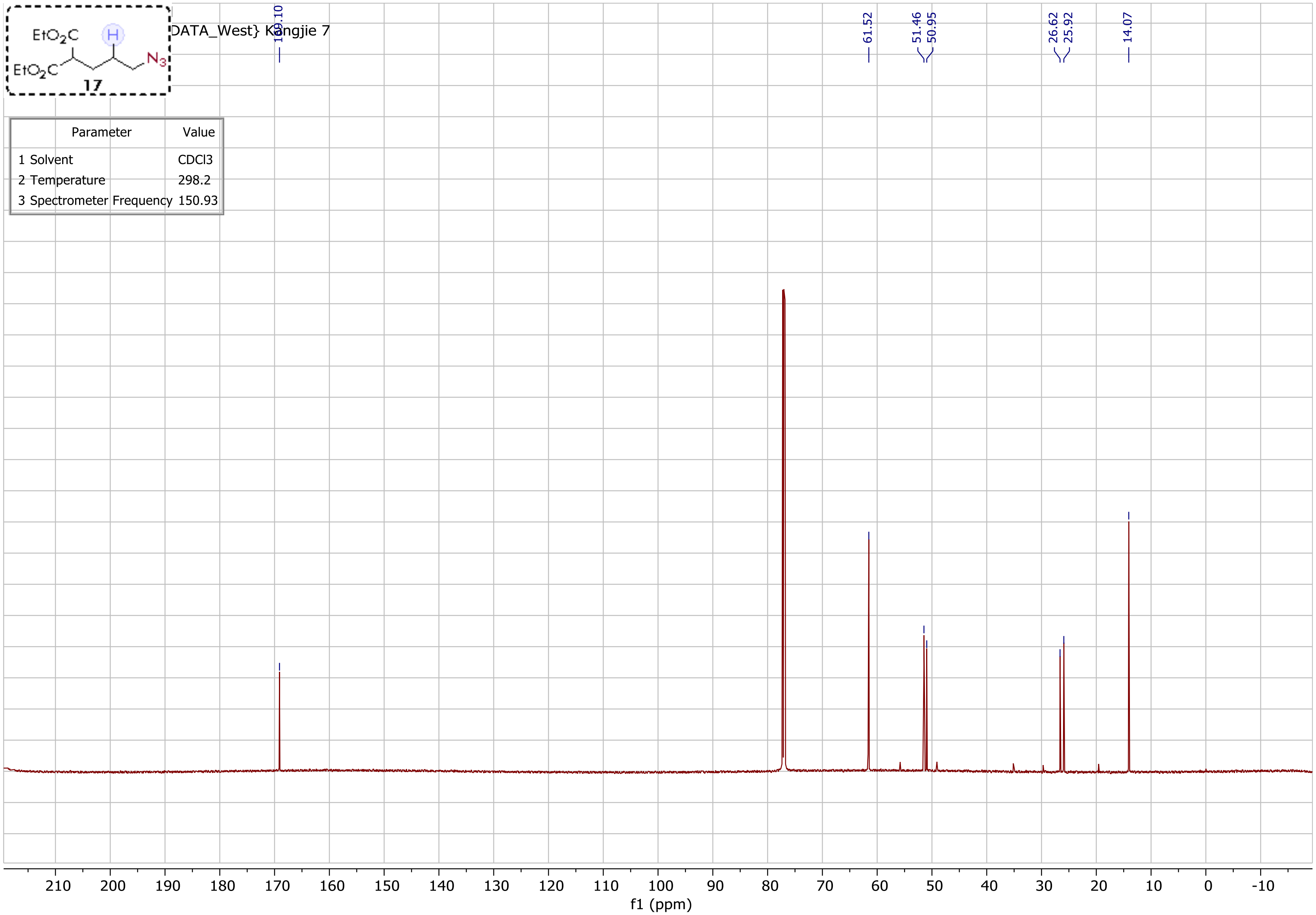
EtO2C
EtO2C
H
N3
17
DATA_West} Kangjie 7
169.10
61.52
51.46
50.95
26.62
25.92
14.07
Parameter Value
1 Solvent CDCl3
2 Temperature 298.2
3 Spectrometer Frequency 150.93
210 200 190 180 170 160 150 140 130 120 110 100 90 80 70 60 50 40 30 20 10 0 -10
f1 (ppm)

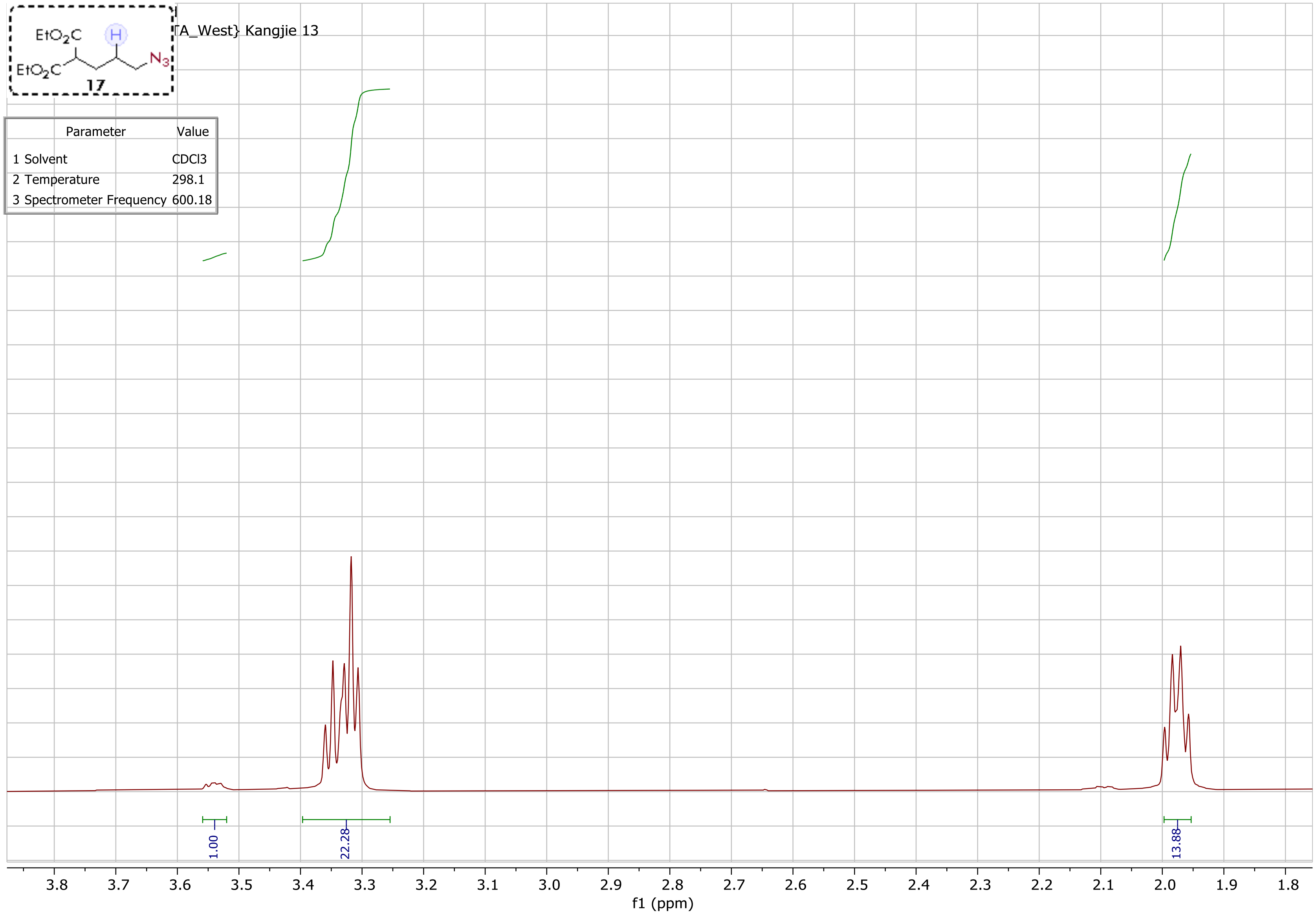

Parameter	Value
1 Solvent	CDCl3
2 Temperature	298.1
3 Spectrometer Frequency	600.18

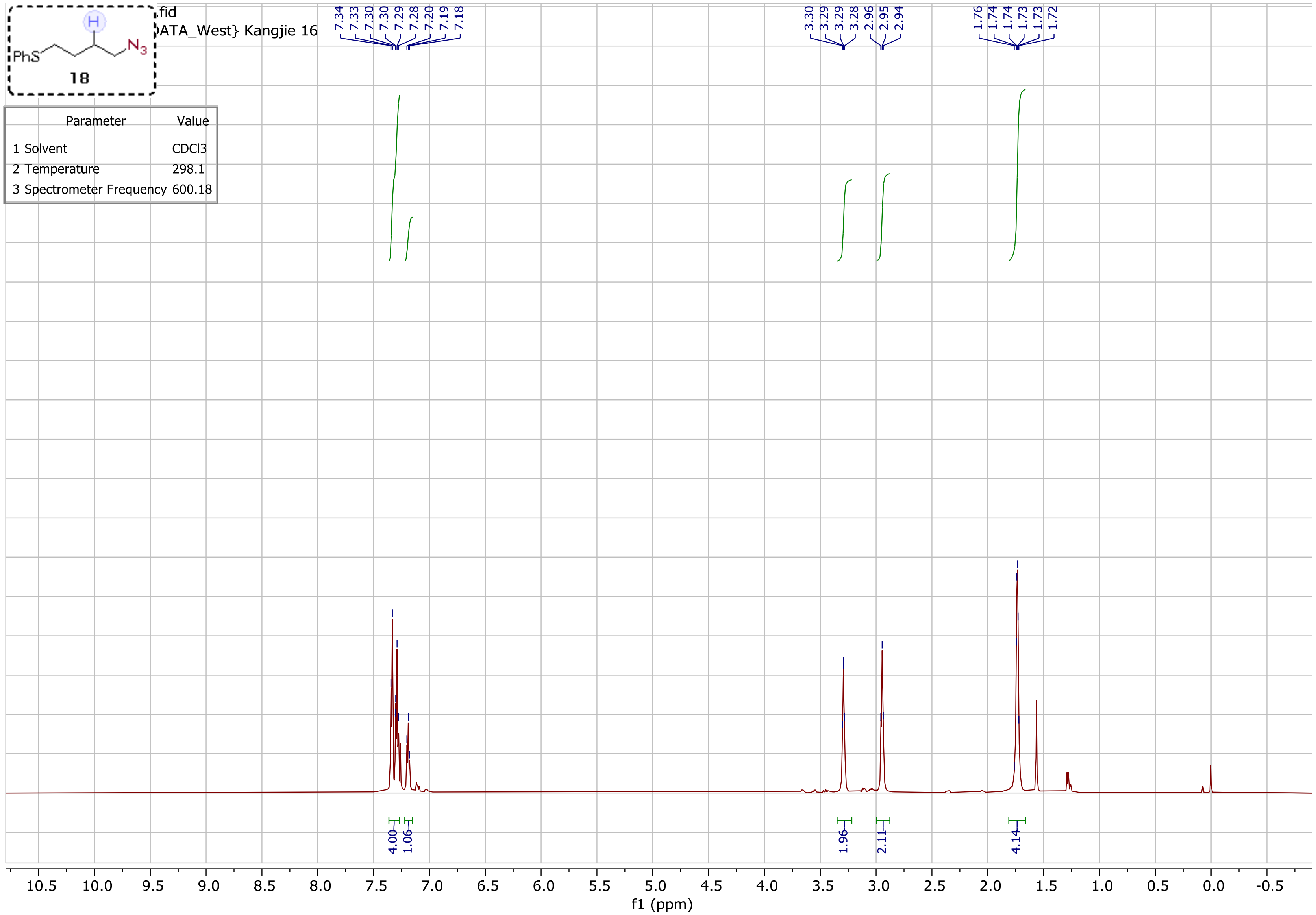
PhS
N3
18
fid
DATA_West} Kangjie 16
Parameter Value
1 Solvent CDCl3
2 Temperature 298.1
3 Spectrometer Frequency 600.18
7.34 7.33 7.30 7.30 7.29 7.28 7.20 7.19 7.18
3.30 3.29 3.29 3.28 2.96 2.95 2.94
1.76 1.74 1.74 1.73 1.73 1.72
4.00 1.06 1.96 2.11 4.14
10.5 10.0 9.5 9.0 8.5 8.0 7.5 7.0 6.5 6.0 5.5 5.0 4.5 4.0 3.5 3.0 2.5 2.0 1.5 1.0 0.5 0.0 -0.5
f1 (ppm)

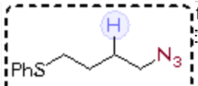

18

Parameter	Value
1 Solvent	CDCl3
2 Temperature	298.1
3 Spectrometer Frequency	150.93

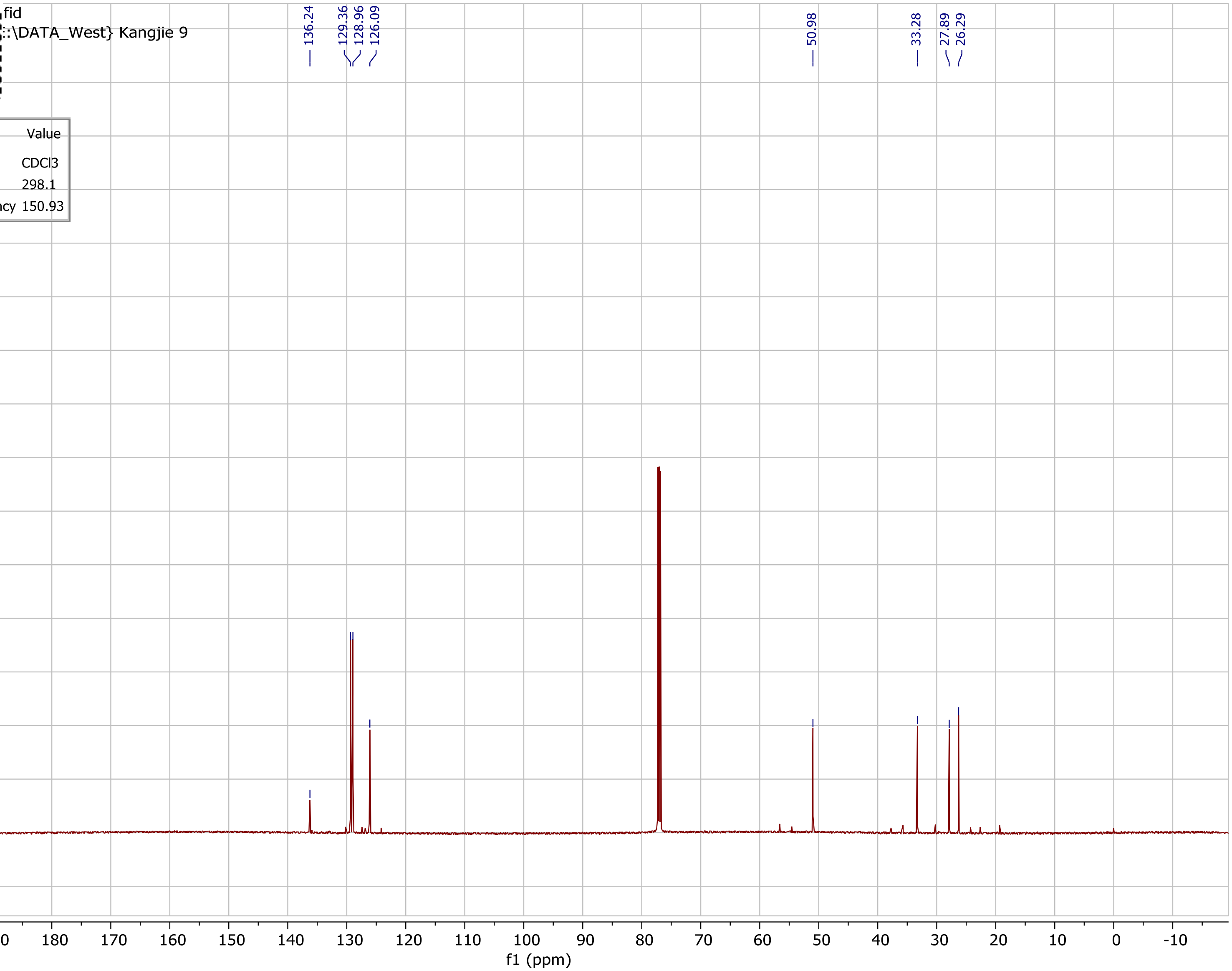

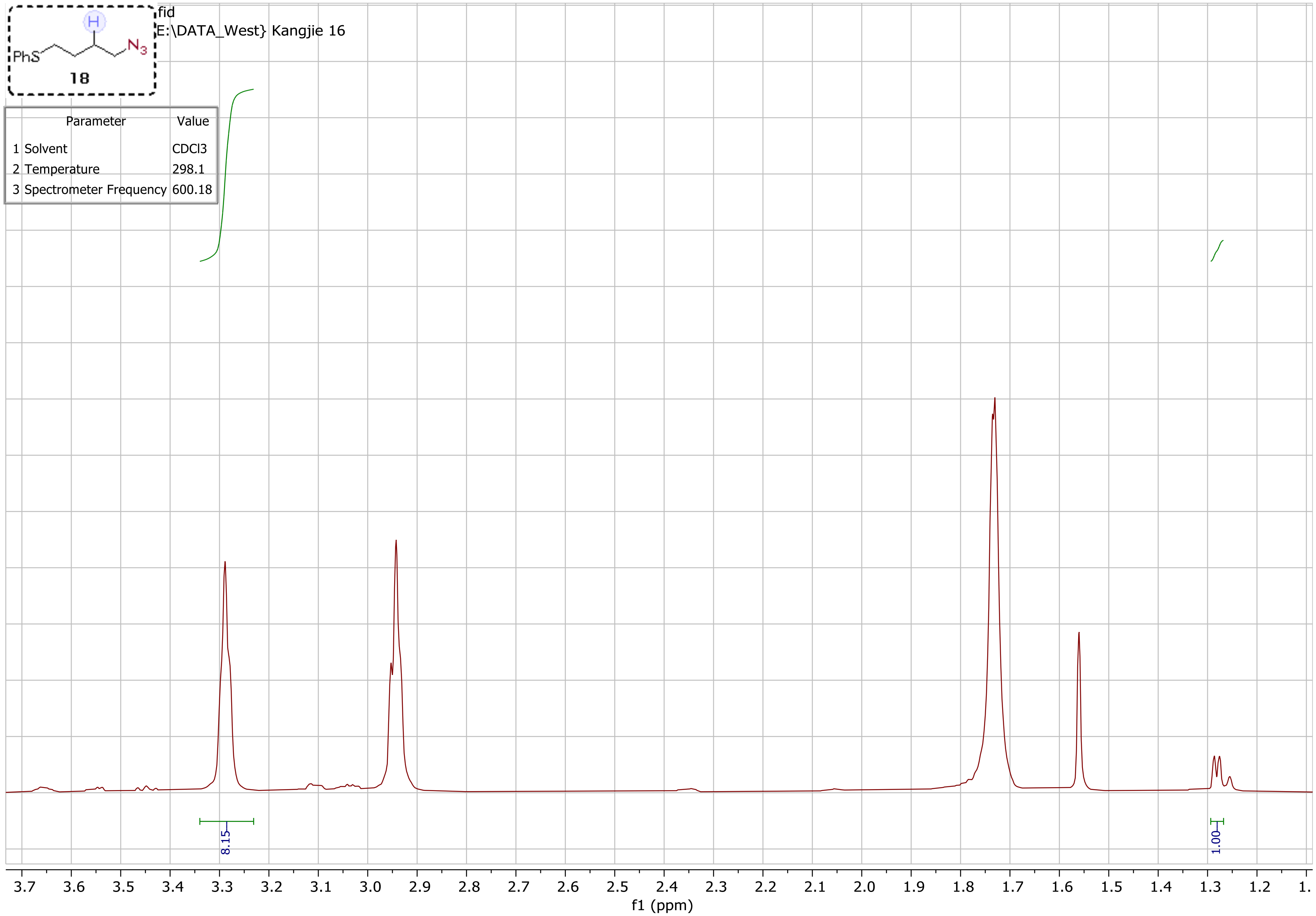

PhS
N3
18
fid
E:\DATA_West} Kangjie 16
Parameter Value
1 Solvent CDCl3
2 Temperature 298.1
3 Spectrometer Frequency 600.18
8.15
1.00
3.7 3.6 3.5 3.4 3.3 3.2 3.1 3.0 2.9 2.8 2.7 2.6 2.5 2.4 2.3 2.2 2.1 2.0 1.9 1.8 1.7 1.6 1.5 1.4 1.3 1.2 1.
f1 (ppm)

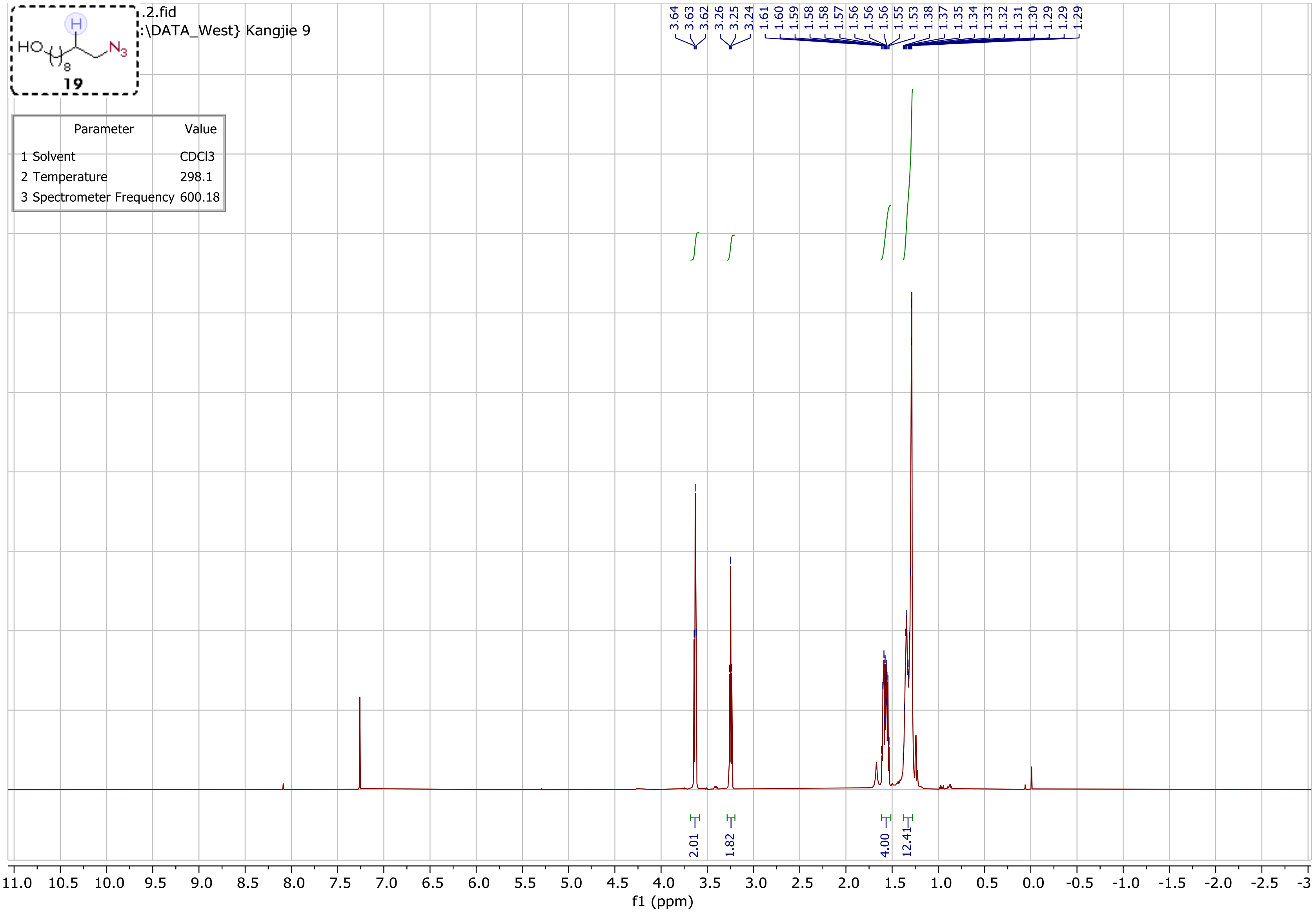
HO
N3
8
19
.2.fid
:\DATA_West} Kangjie 9
Parameter Value
1 Solvent CDCl3
2 Temperature 298.1
3 Spectrometer Frequency 600.18
3.64 3.63 3.62 3.26 3.25 3.24 1.61 1.60 1.59 1.58 1.58 1.57 1.56 1.56 1.56 1.55 1.53 1.38 1.37 1.35 1.34 1.33 1.32 1.31 1.30 1.29 1.29 1.29
2.01
1.82
4.00
12.41
11.0 10.5 10.0 9.5 9.0 8.5 8.0 7.5 7.0 6.5 6.0 5.5 5.0 4.5 4.0 3.5 3.0 2.5 2.0 1.5 1.0 0.5 0.0 -0.5 -1.0 -1.5 -2.0 -2.5 -3
f1 (ppm)

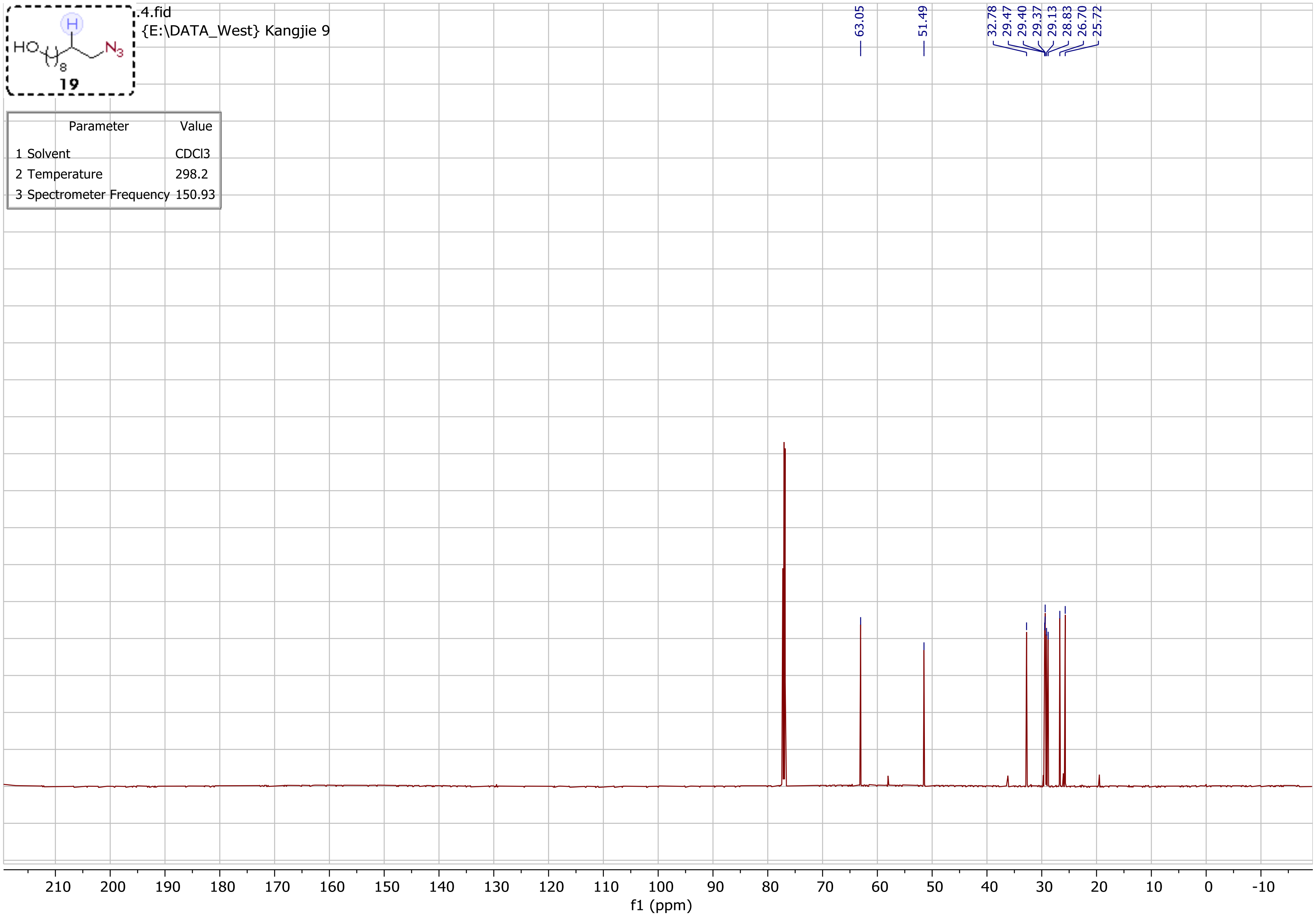
HO
N3
8
19
.4.fid
{E:\DATA_West} Kangjie 9
Parameter Value
1 Solvent CDCl3
2 Temperature 298.2
3 Spectrometer Frequency 150.93
63.05
51.49
32.78
29.47
29.40
29.37
29.13
28.83
26.70
25.72
210 200 190 180 170 160 150 140 130 120 110 100 90 80 70 60 50 40 30 20 10 0 -10
f1 (ppm)

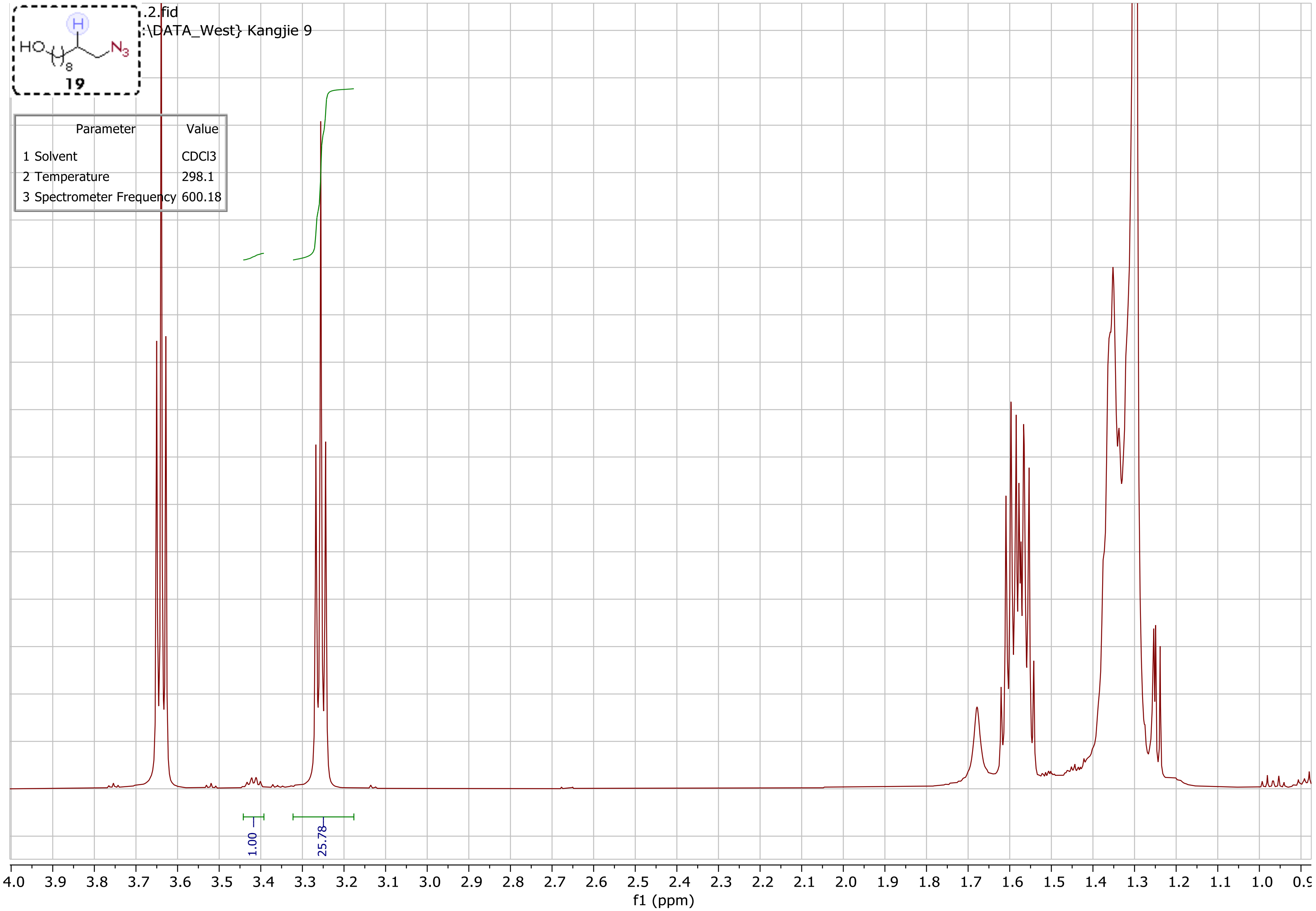
HO
H
N_3
8
19
.2.fid
:\DATA_West} Kangjie 9
Parameter Value
1 Solvent CDCl3
2 Temperature 298.1
3 Spectrometer Frequency 600.18
1.00
25.78
4.0 3.9 3.8 3.7 3.6 3.5 3.4 3.3 3.2 3.1 3.0 2.9 2.8 2.7 2.6 2.5 2.4 2.3 2.2 2.1 2.0 1.9 1.8 1.7 1.6 1.5 1.4 1.3 1.2 1.1 1.0 0.9
f1 (ppm)

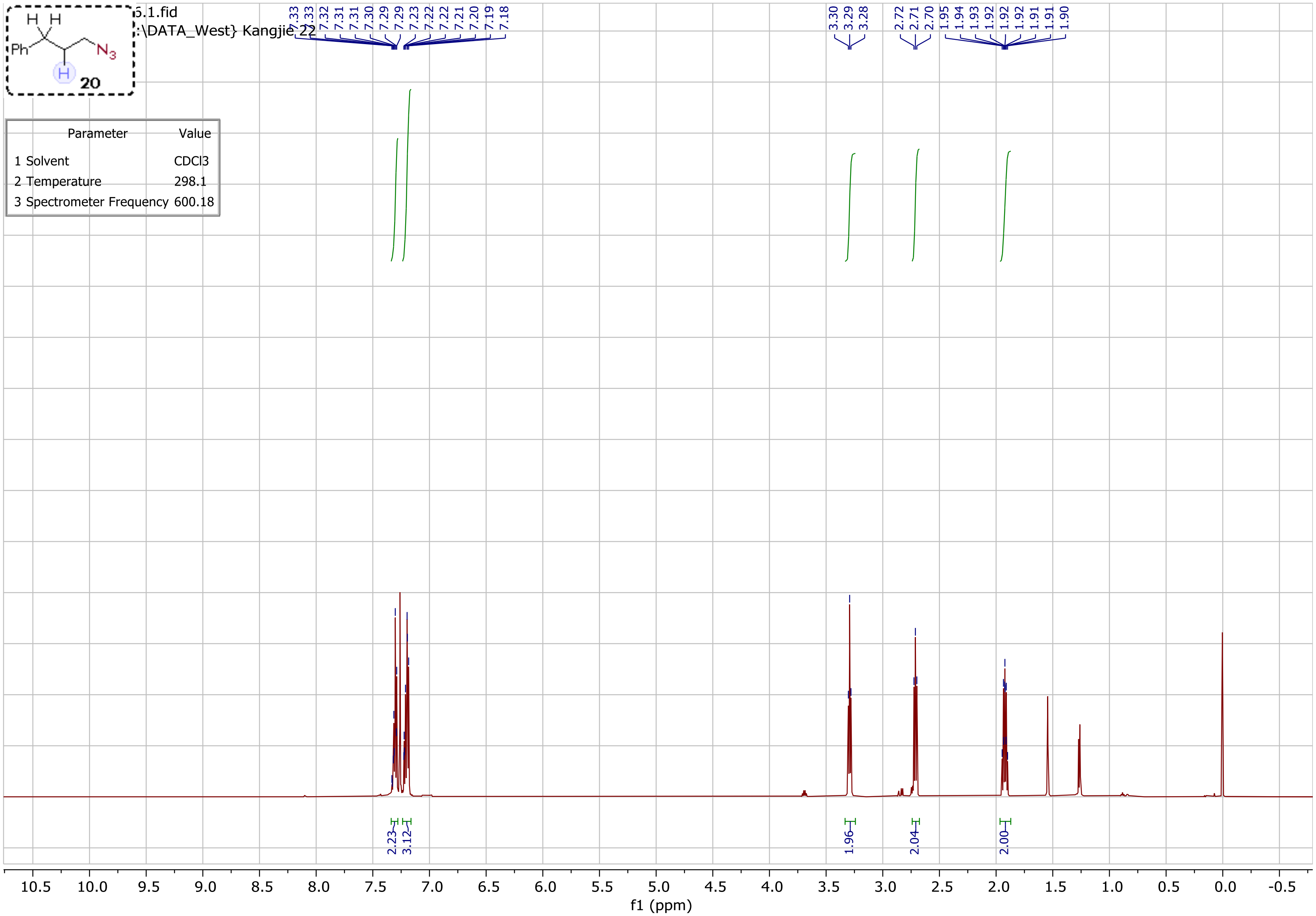

20
Ph
N3
.1.fid
:\DATA_West} Kangjie 22
Parameter Value
1 Solvent CDCl3
2 Temperature 298.1
3 Spectrometer Frequency 600.18
7.33 7.33 7.32 7.31 7.31 7.30 7.29 7.29 7.23 7.22 7.22 7.21 7.20 7.19 7.18
3.30 3.29 3.28
2.72 2.71 2.70
1.95 1.94 1.93 1.92 1.92 1.92 1.91 1.91 1.90
2.23
3.12
1.96
2.04
2.00
10.5 10.0 9.5 9.0 8.5 8.0 7.5 7.0 6.5 6.0 5.5 5.0 4.5 4.0 3.5 3.0 2.5 2.0 1.5 1.0 0.5 0.0 -0.5
f1 (ppm)

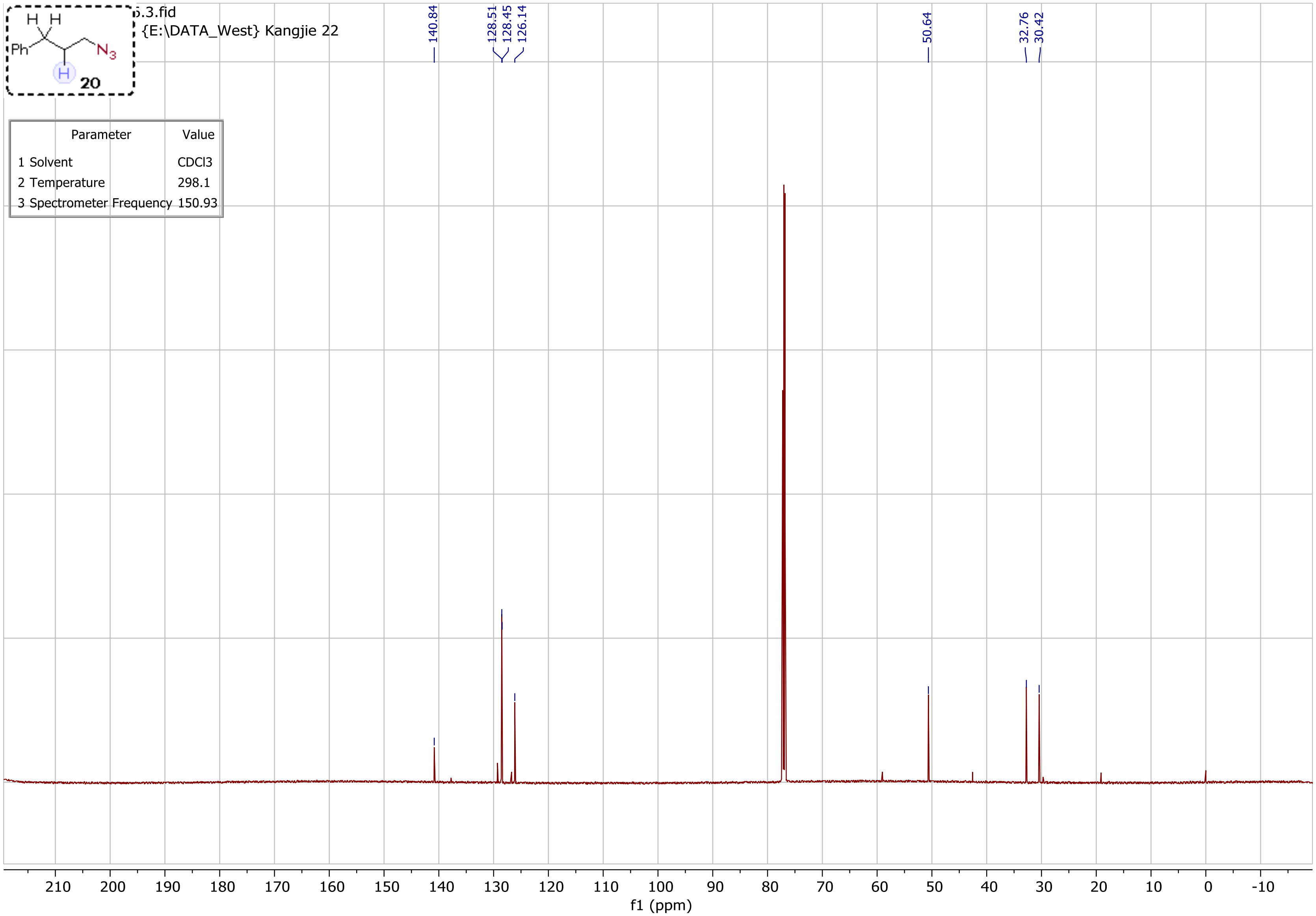

H H
Ph
N3
H
20
.3.fid
{E:\DATA_West} Kangjie 22
140.84
128.51
128.45
126.14
50.64
32.76
30.42
Parameter Value
1 Solvent CDCl3
2 Temperature 298.1
3 Spectrometer Frequency 150.93
210 200 190 180 170 160 150 140 130 120 110 100 90 80 70 60 50 40 30 20 10 0 -10
f1 (ppm)

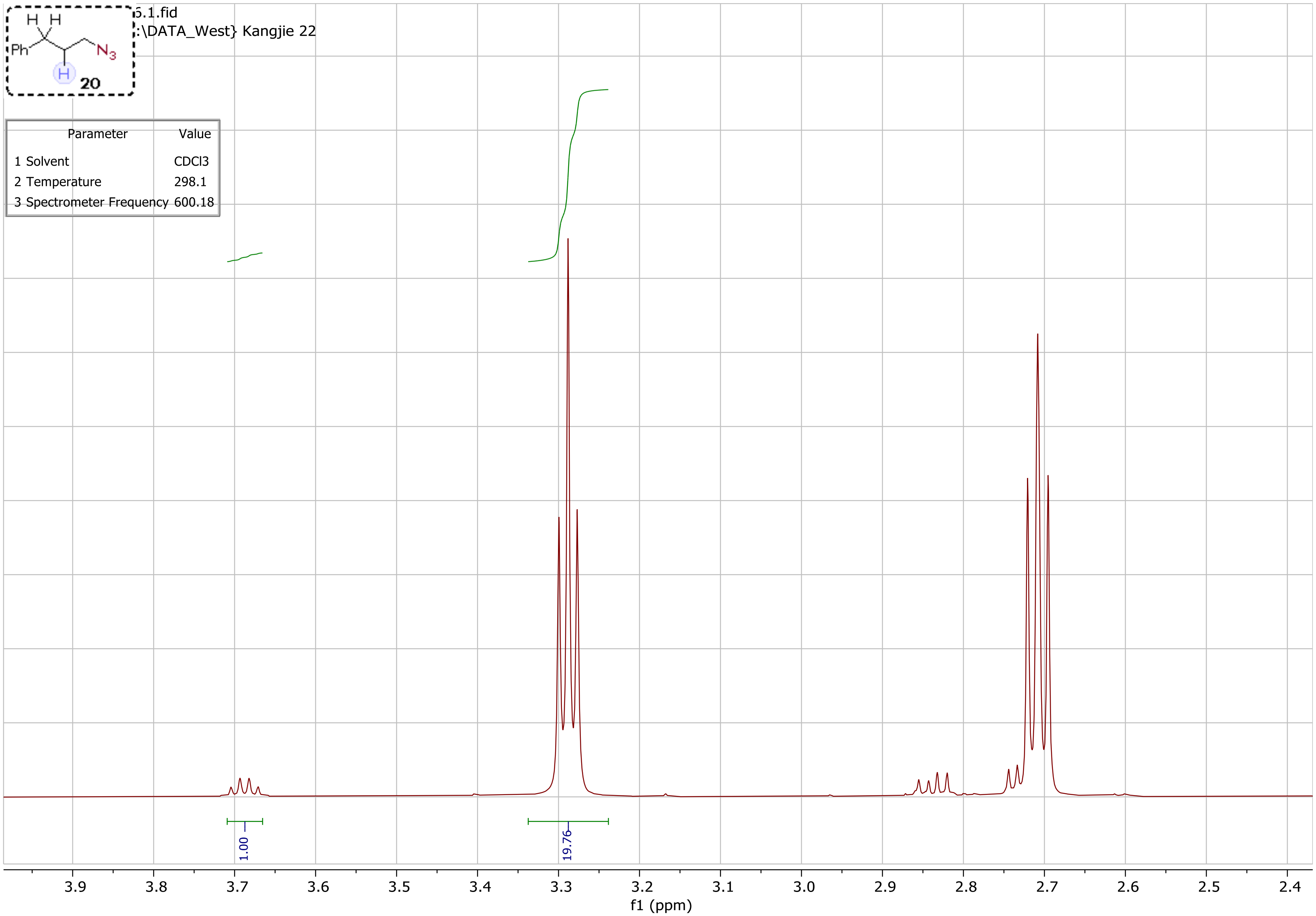
5.1.fid
:\DATA_West} Kangjie 22
Ph
N3
20
Parameter Value
1 Solvent CDCl3
2 Temperature 298.1
3 Spectrometer Frequency 600.18
1.00
19.76
3.9 3.8 3.7 3.6 3.5 3.4 3.3 3.2 3.1 3.0 2.9 2.8 2.7 2.6 2.5 2.4
f1 (ppm)

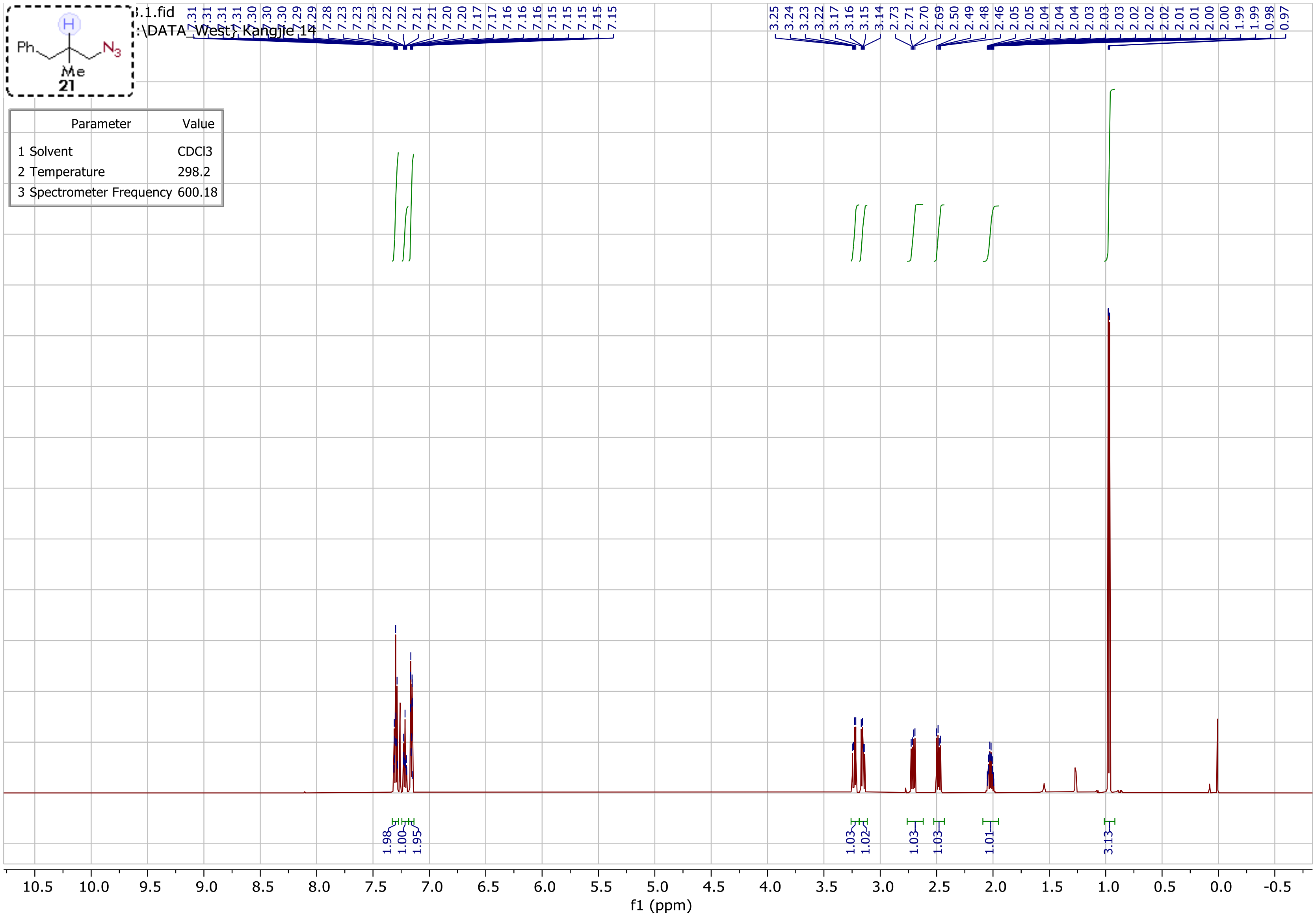

	Parameter	Value
1	Solvent	CDCl3
2	Temperature	298.2
3	Spectrometer Frequency	600.18

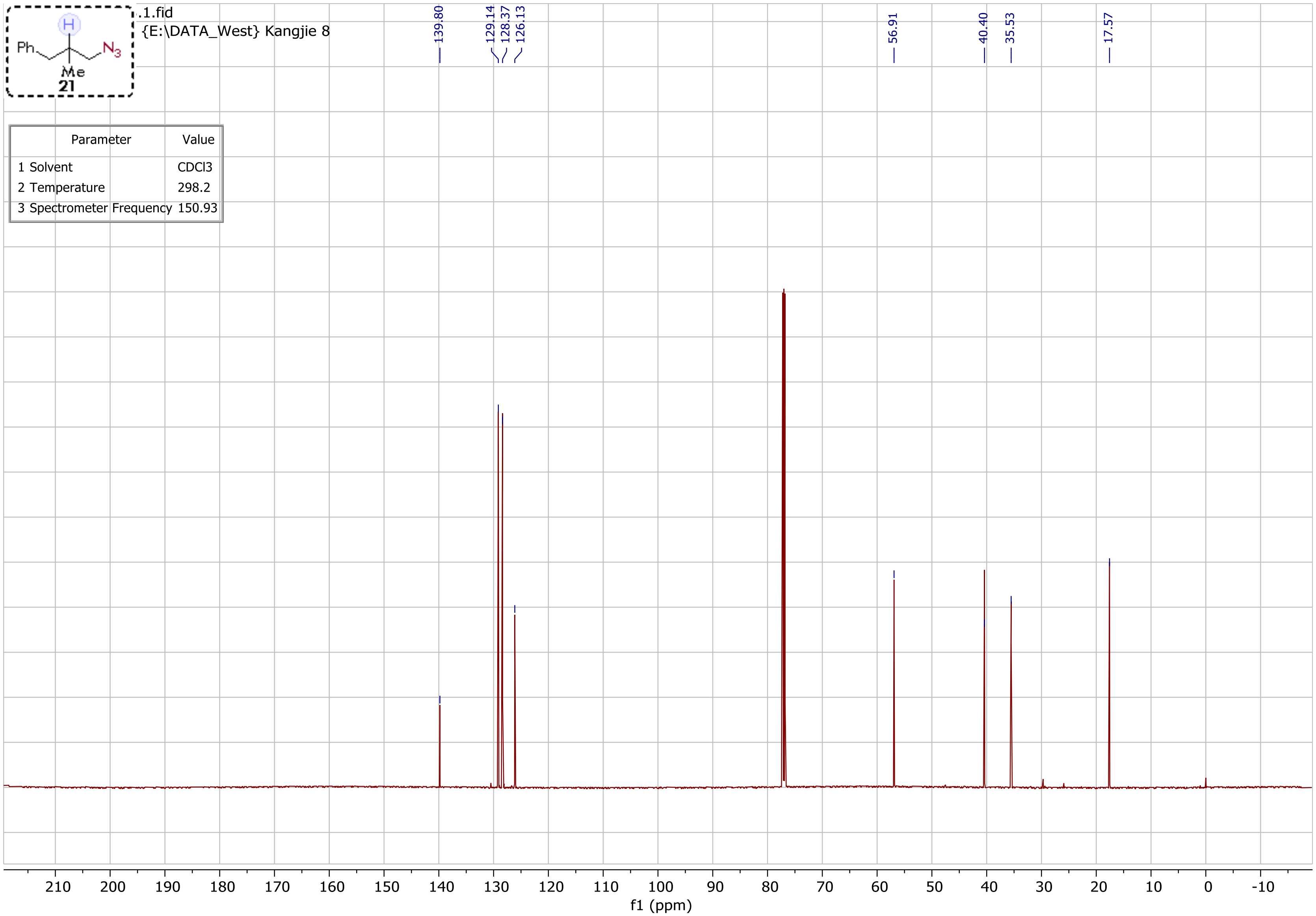

Ph
H
N3
Me
21
.1.fid
{E:\DATA_West} Kangjie 8
139.80
129.14
128.37
126.13
56.91
40.40
35.53
17.57
Parameter | Value
1 Solvent | CDCl3
2 Temperature | 298.2
3 Spectrometer Frequency | 150.93
210 200 190 180 170 160 150 140 130 120 110 100 90 80 70 60 50 40 30 20 10 0 -10
f1 (ppm)

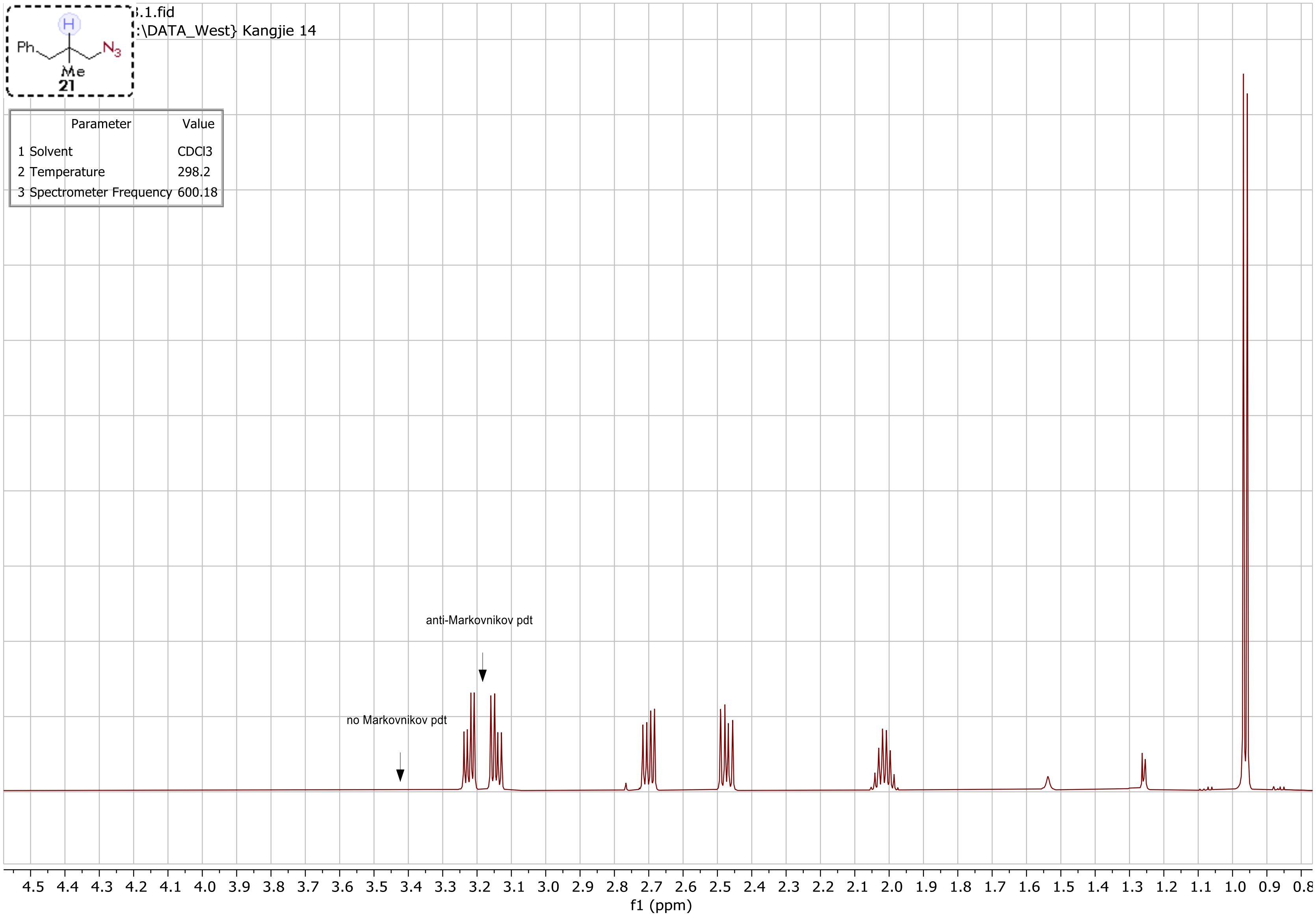

.1.fid
:\DATA_West} Kangjie 14
Ph
N3
Me
21
Parameter
Value
1 Solvent
CDCl3
2 Temperature
298.2
3 Spectrometer Frequency
600.18
anti-Markovnikov pdt
no Markovnikov pdt
4.5 4.4 4.3 4.2 4.1 4.0 3.9 3.8 3.7 3.6 3.5 3.4 3.3 3.2 3.1 3.0 2.9 2.8 2.7 2.6 2.5 2.4 2.3 2.2 2.1 2.0 1.9 1.8 1.7 1.6 1.5 1.4 1.3 1.2 1.1 1.0 0.9 0.8
f1 (ppm)

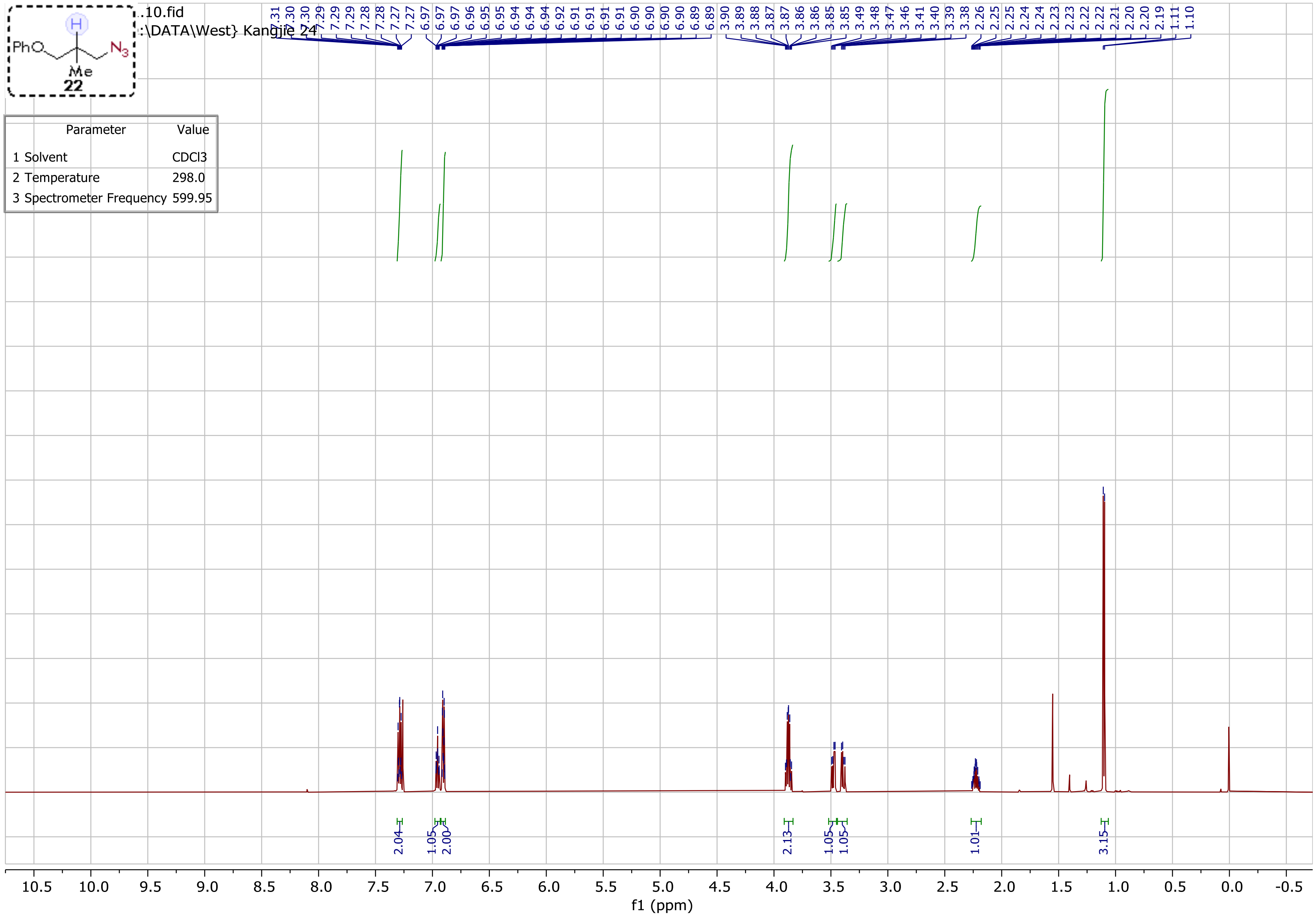

	Parameter	Value
1	Solvent	CDCl3
2	Temperature	298.0
3	Spectrometer Frequency	599.95

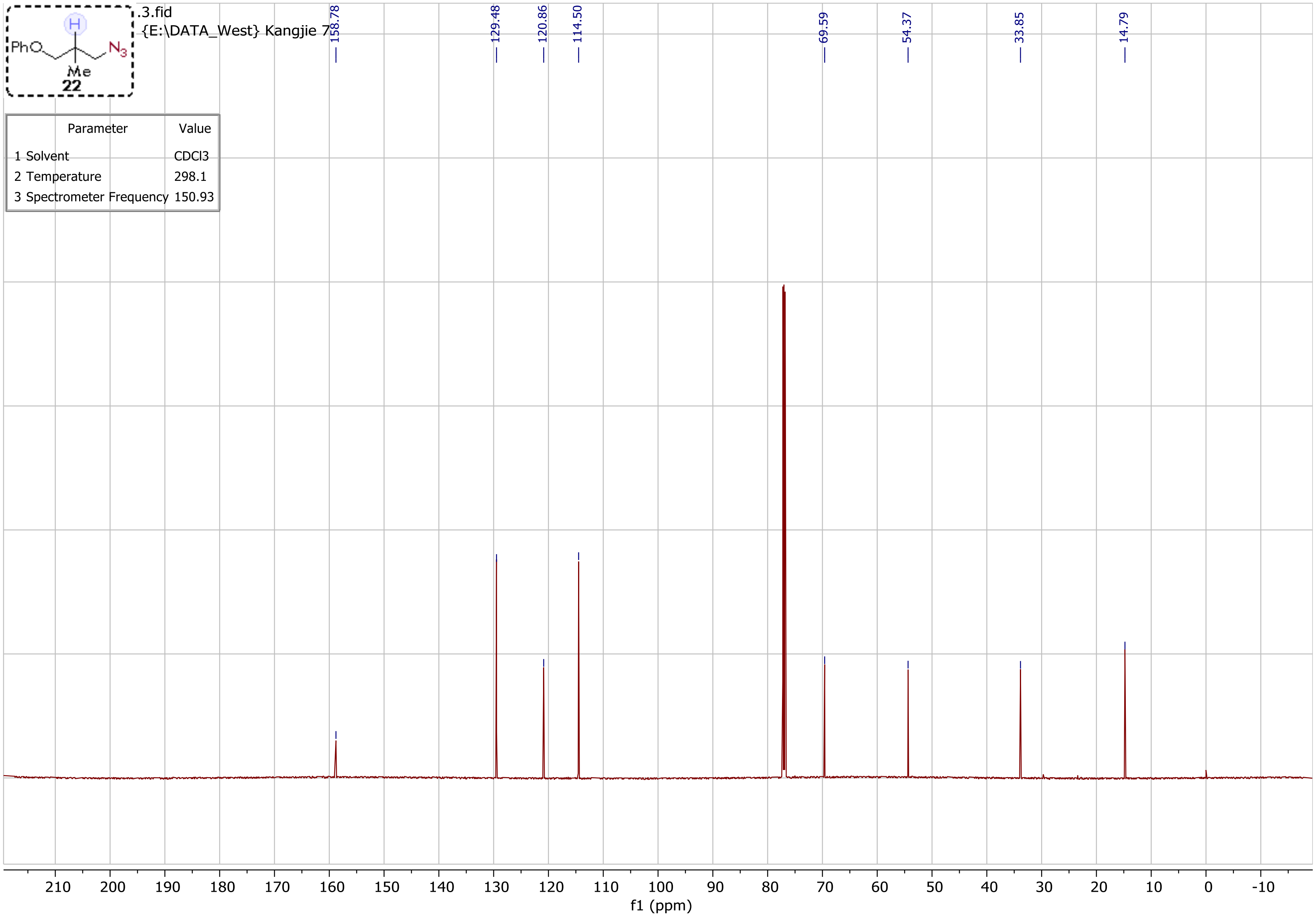

.3.fid
{E:\DATA_West} Kangjie 7
PhO
N3
Me
22
158.78
129.48
120.86
114.50
69.59
54.37
33.85
14.79
Parameter Value
1 Solvent CDCl3
2 Temperature 298.1
3 Spectrometer Frequency 150.93
210 200 190 180 170 160 150 140 130 120 110 100 90 80 70 60 50 40 30 20 10 0 -10
f1 (ppm)

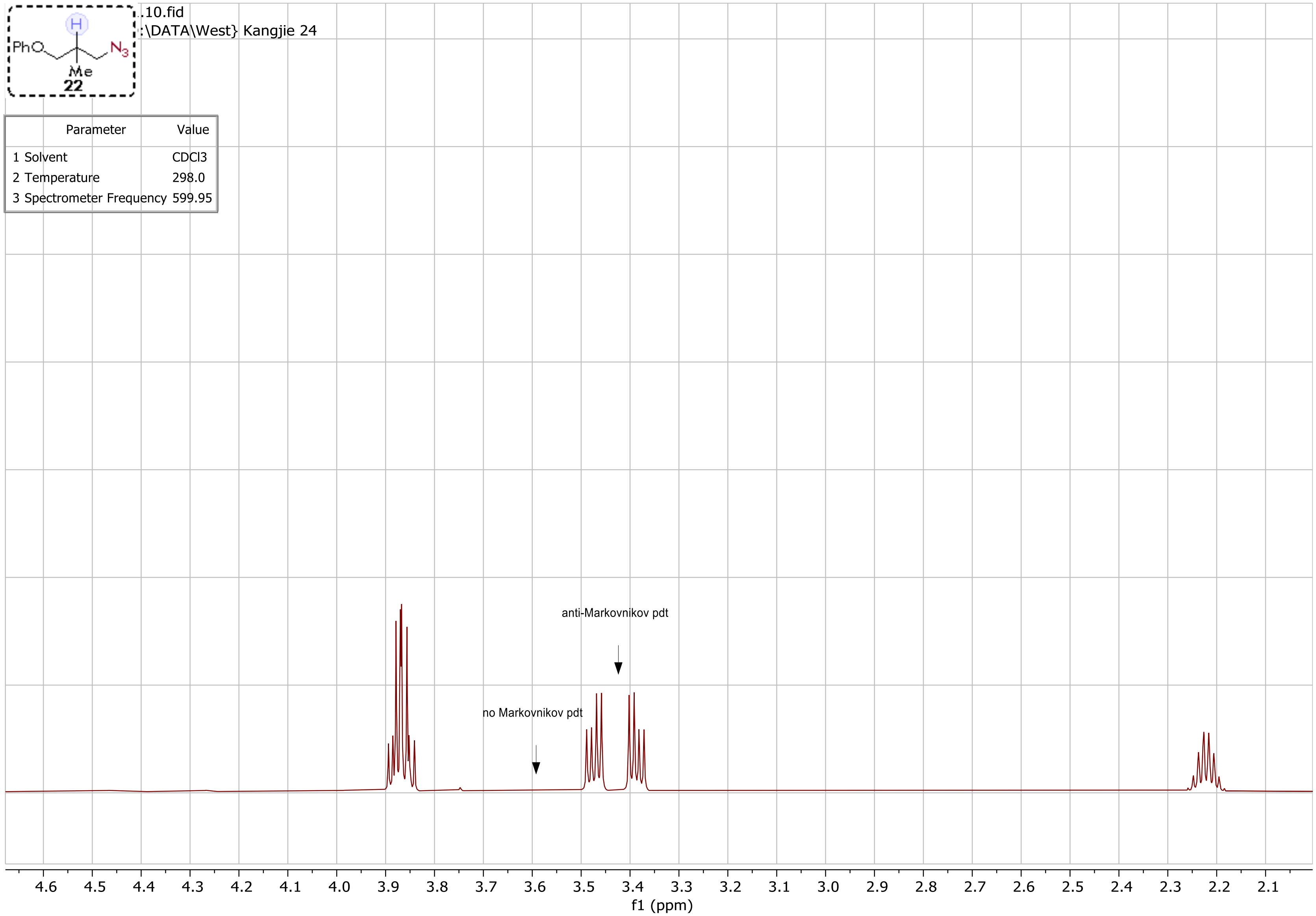
PhO
N3
Me
22
.10.fid
:\DATA\West} Kangjie 24
Parameter Value
1 Solvent CDCl3
2 Temperature 298.0
3 Spectrometer Frequency 599.95
anti-Markovnikov pdt
no Markovnikov pdt
4.6 4.5 4.4 4.3 4.2 4.1 4.0 3.9 3.8 3.7 3.6 3.5 3.4 3.3 3.2 3.1 3.0 2.9 2.8 2.7 2.6 2.5 2.4 2.3 2.2 2.1
f1 (ppm)

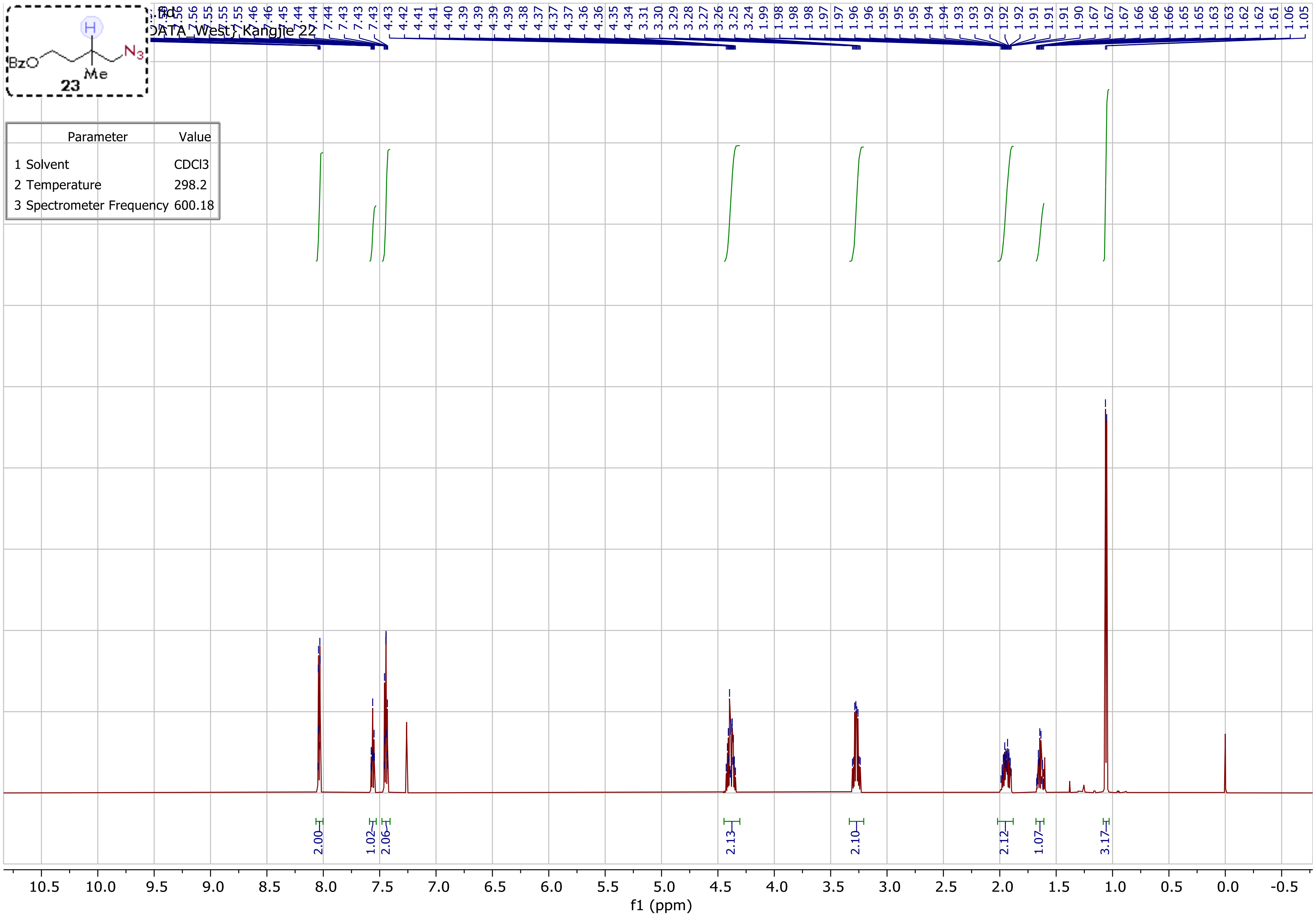
BzO
N3
Me
H
23
Parameter | Value
1 Solvent | CDCl3
2 Temperature | 298.2
3 Spectrometer Frequency | 600.18
f1 (ppm)

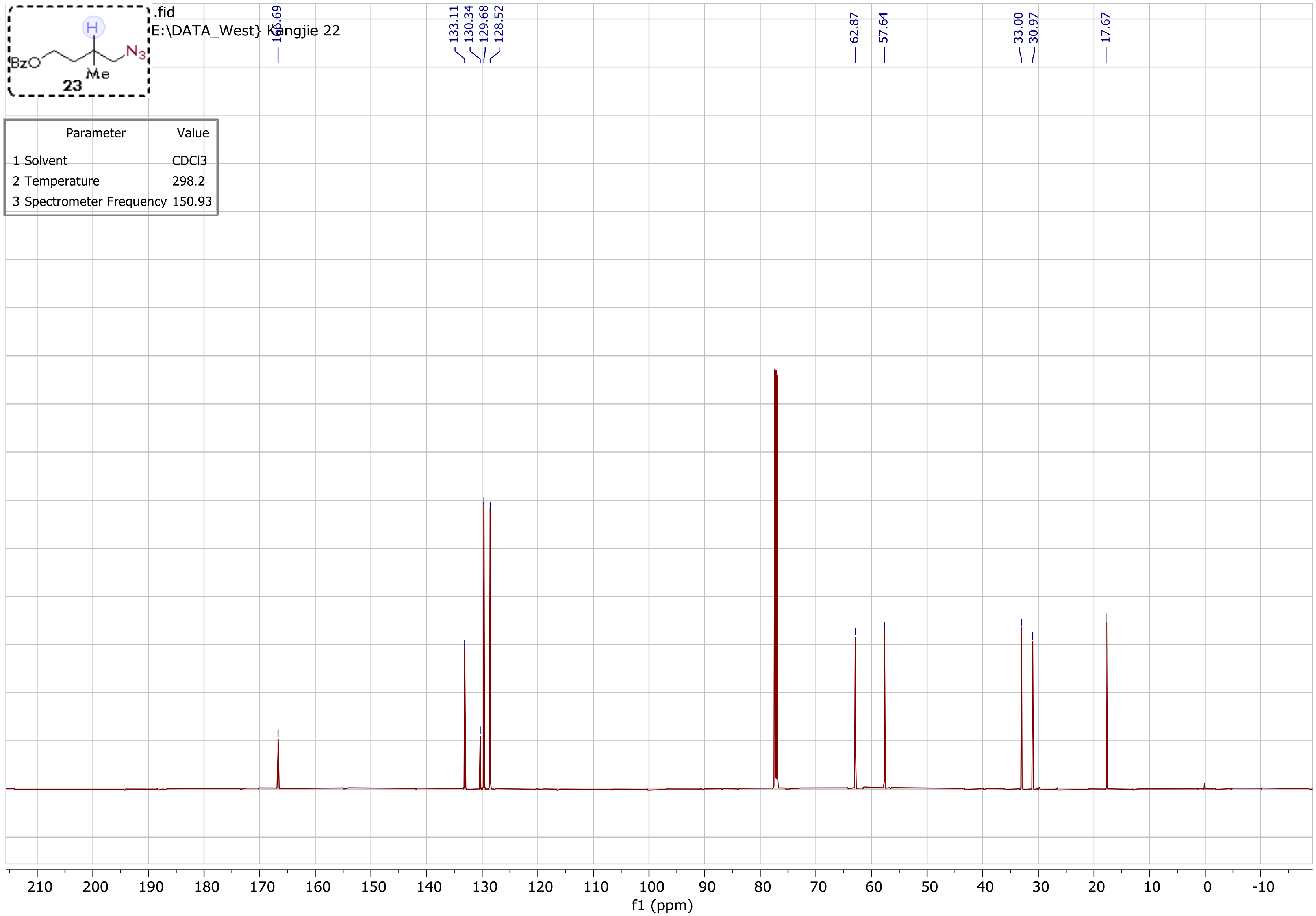

.fid
E:\DATA_West} Kangjie 22
133.11
130.34
129.68
128.52
62.87
57.64
33.00
30.97
17.67
BzO
N3
Me
23
Parameter | Value
1 Solvent | CDCl3
2 Temperature | 298.2
3 Spectrometer Frequency | 150.93
210 200 190 180 170 160 150 140 130 120 110 100 90 80 70 60 50 40 30 20 10 0 -10
f1 (ppm)

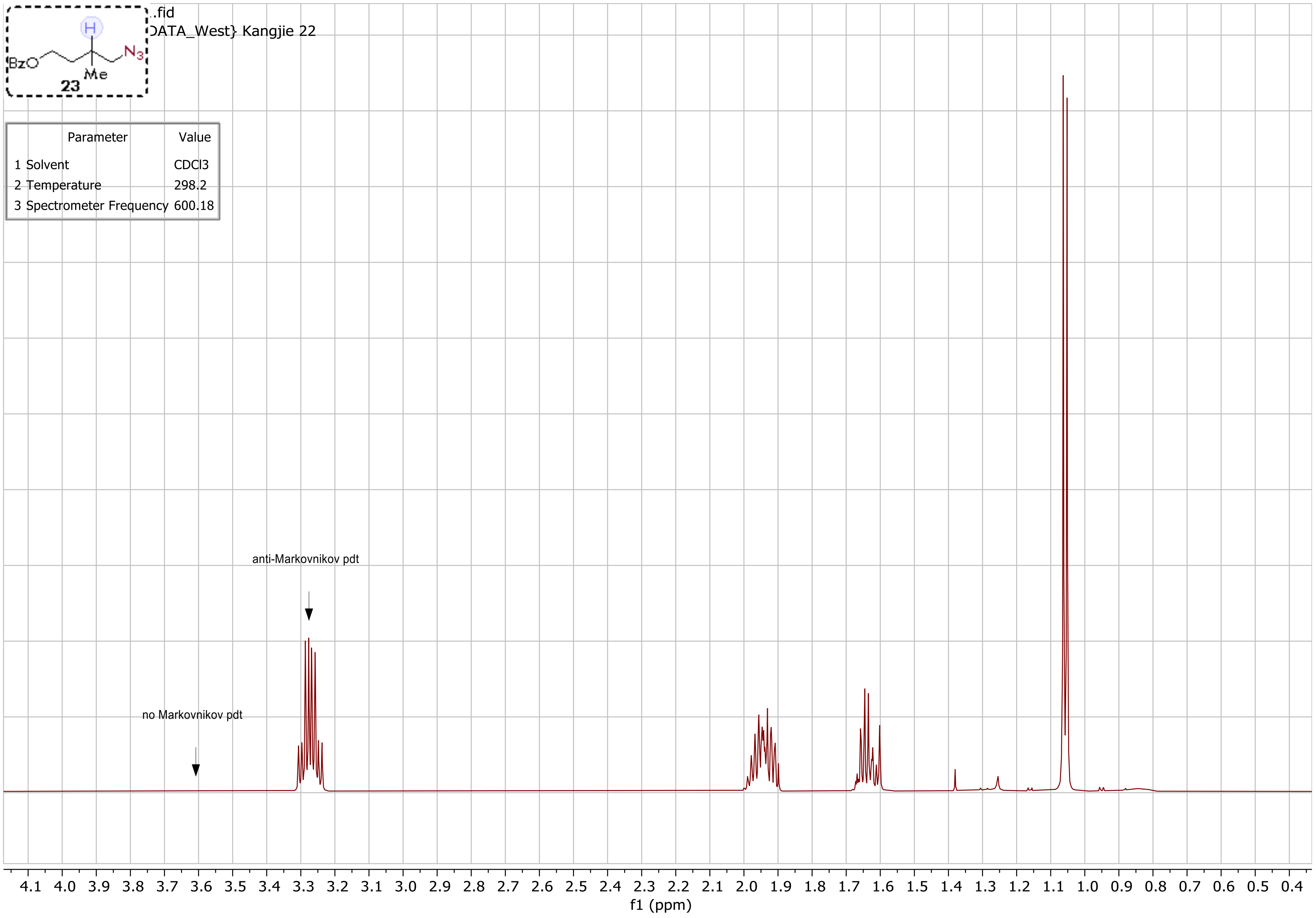
.fid
DATA_West} Kangjie 22
BzO
N3
Me
23
Parameter Value
1 Solvent CDCl3
2 Temperature 298.2
3 Spectrometer Frequency 600.18
anti-Markovnikov pdt
no Markovnikov pdt
4.1 4.0 3.9 3.8 3.7 3.6 3.5 3.4 3.3 3.2 3.1 3.0 2.9 2.8 2.7 2.6 2.5 2.4 2.3 2.2 2.1 2.0 1.9 1.8 1.7 1.6 1.5 1.4 1.3 1.2 1.1 1.0 0.9 0.8 0.7 0.6 0.5 0.4
f1 (ppm)

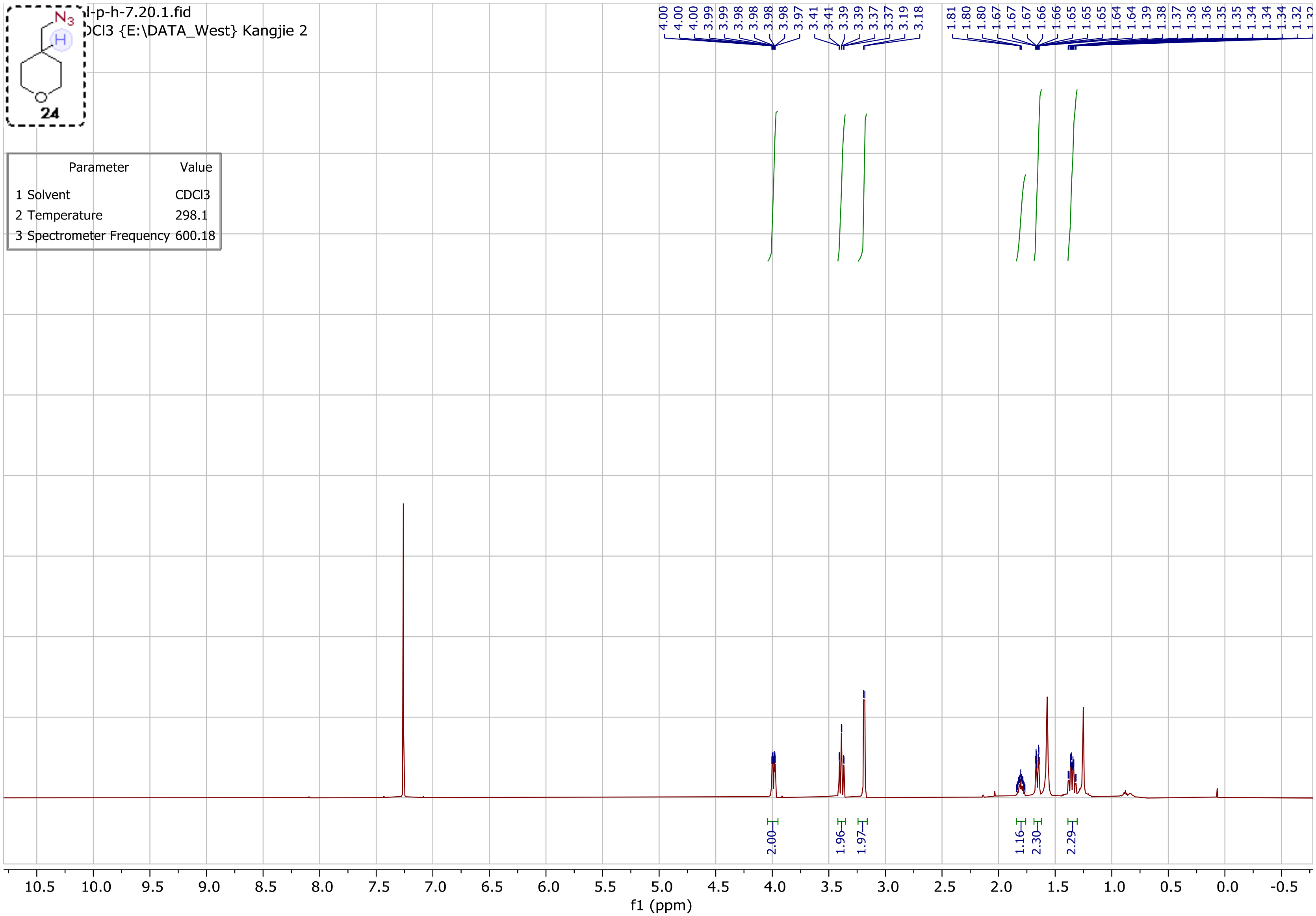
N3
H
O
24
l-p-h-7.20.1.fid
DCl3 {E:\DATA_West} Kangjie 2
Parameter Value
1 Solvent CDCl3
2 Temperature 298.1
3 Spectrometer Frequency 600.18
4.00 4.00 4.00 3.99 3.99 3.98 3.98 3.98 3.98 3.97 3.41 3.39 3.39 3.39 3.37 3.19 3.18
1.81 1.80 1.80 1.67 1.67 1.67 1.66 1.66 1.65 1.65 1.65 1.64 1.64 1.39 1.38 1.37 1.36 1.36 1.35 1.35 1.34 1.34 1.34 1.32
2.00
1.96
1.97
1.16
2.30
2.29
10.5 10.0 9.5 9.0 8.5 8.0 7.5 7.0 6.5 6.0 5.5 5.0 4.5 4.0 3.5 3.0 2.5 2.0 1.5 1.0 0.5 0.0 -0.5
f1 (ppm)

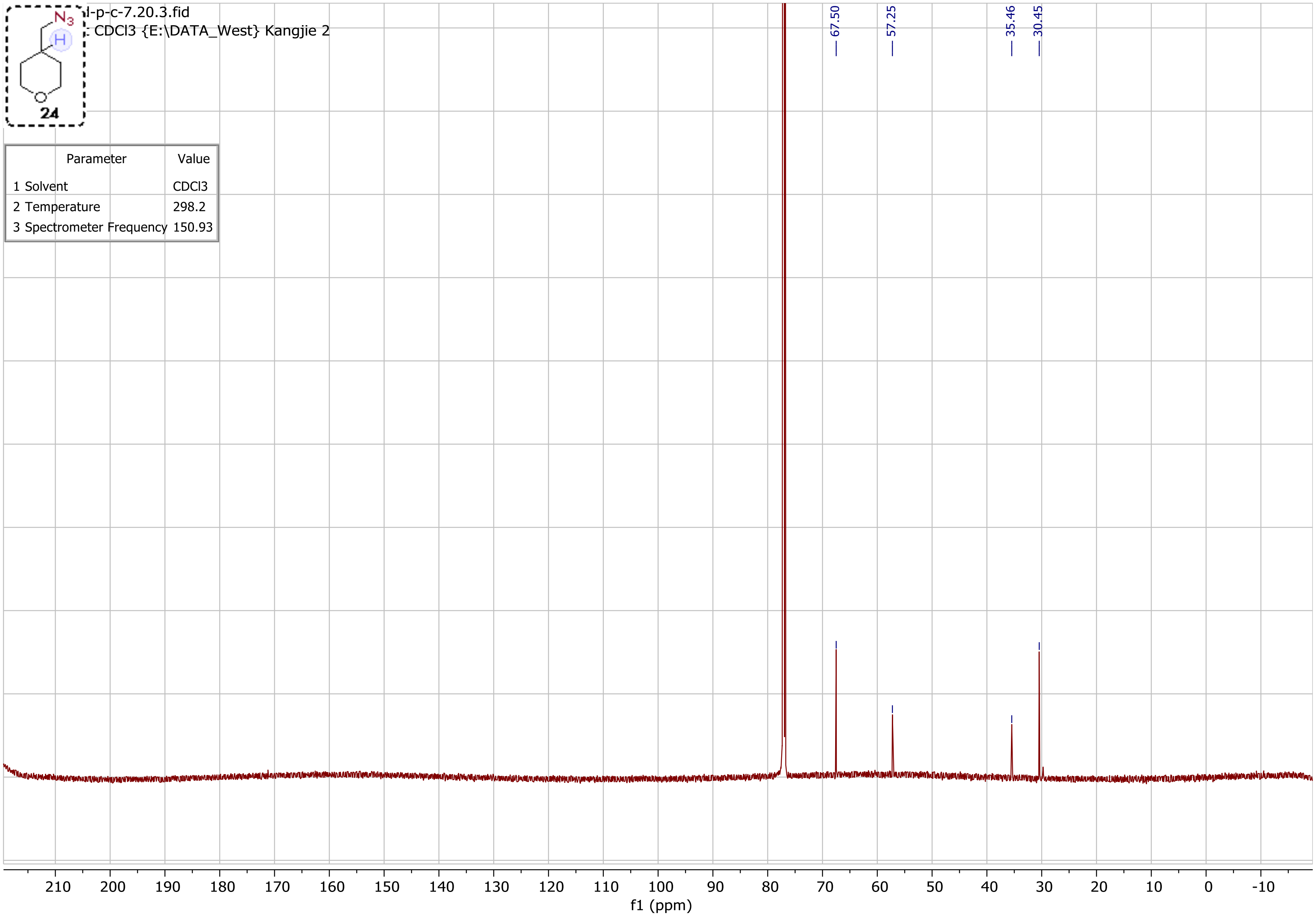
l-p-c-7.20.3.fid
: CDCl3 {E:\DATA_West} Kangjie 2
N3
H
O
24
Parameter | Value
1 Solvent | CDCl3
2 Temperature | 298.2
3 Spectrometer Frequency | 150.93
67.50
57.25
35.46
30.45
210 200 190 180 170 160 150 140 130 120 110 100 90 80 70 60 50 40 30 20 10 0 -10
f1 (ppm)

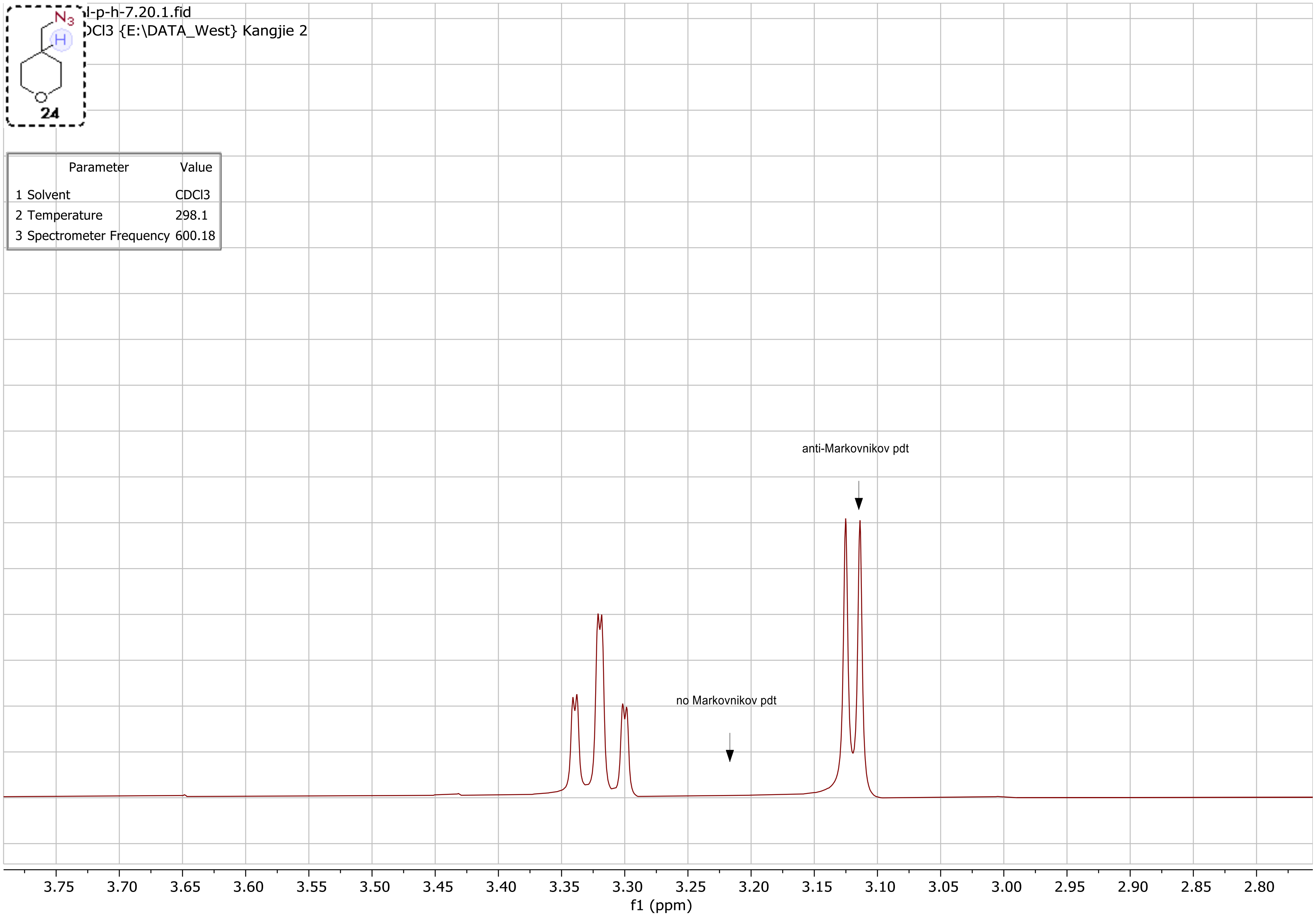

Parameter	Value
1 Solvent	CDCl3
2 Temperature	298.1
3 Spectrometer Frequency	600.18

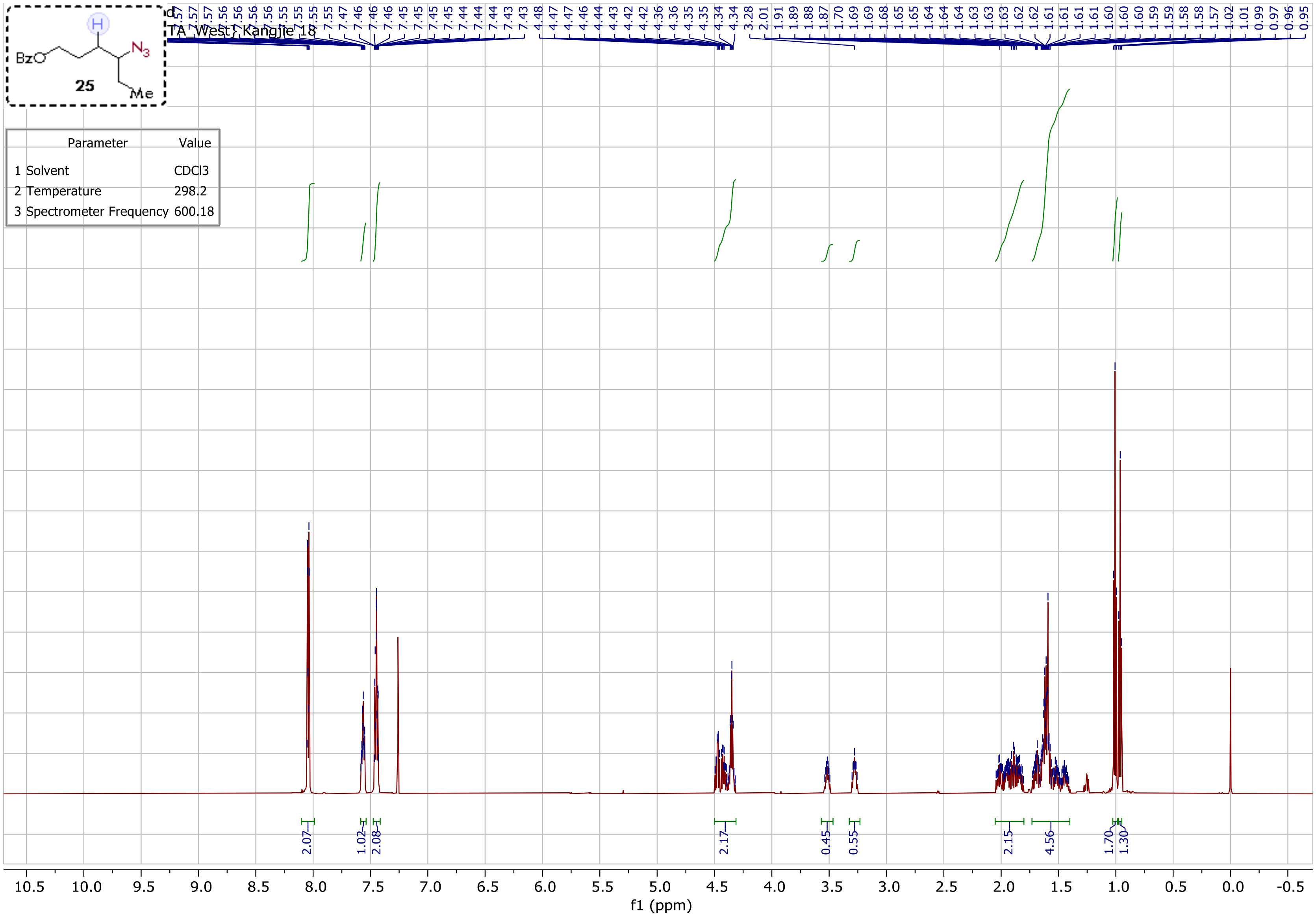

BzO
N3
25
Me
TA_West} Kangjie 18
Parameter Value
1 Solvent CDCl3
2 Temperature 298.2
3 Spectrometer Frequency 600.18
2.07
1.02
2.08
2.17
0.45
0.55
2.15
4.56
1.70
1.30
f1 (ppm)

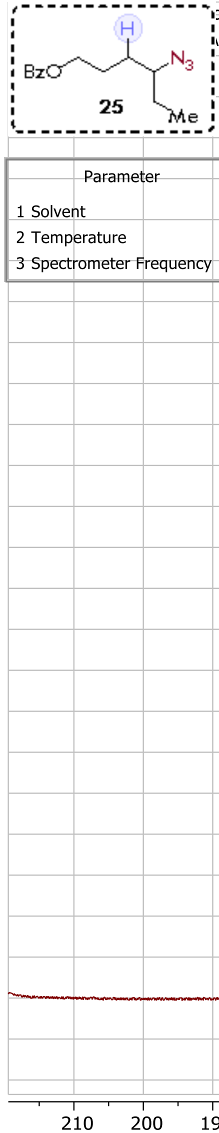

25

Parameter	Value
1 Solvent	CDCl3
2 Temperature	298.1
3 Spectrometer Frequency	150.93

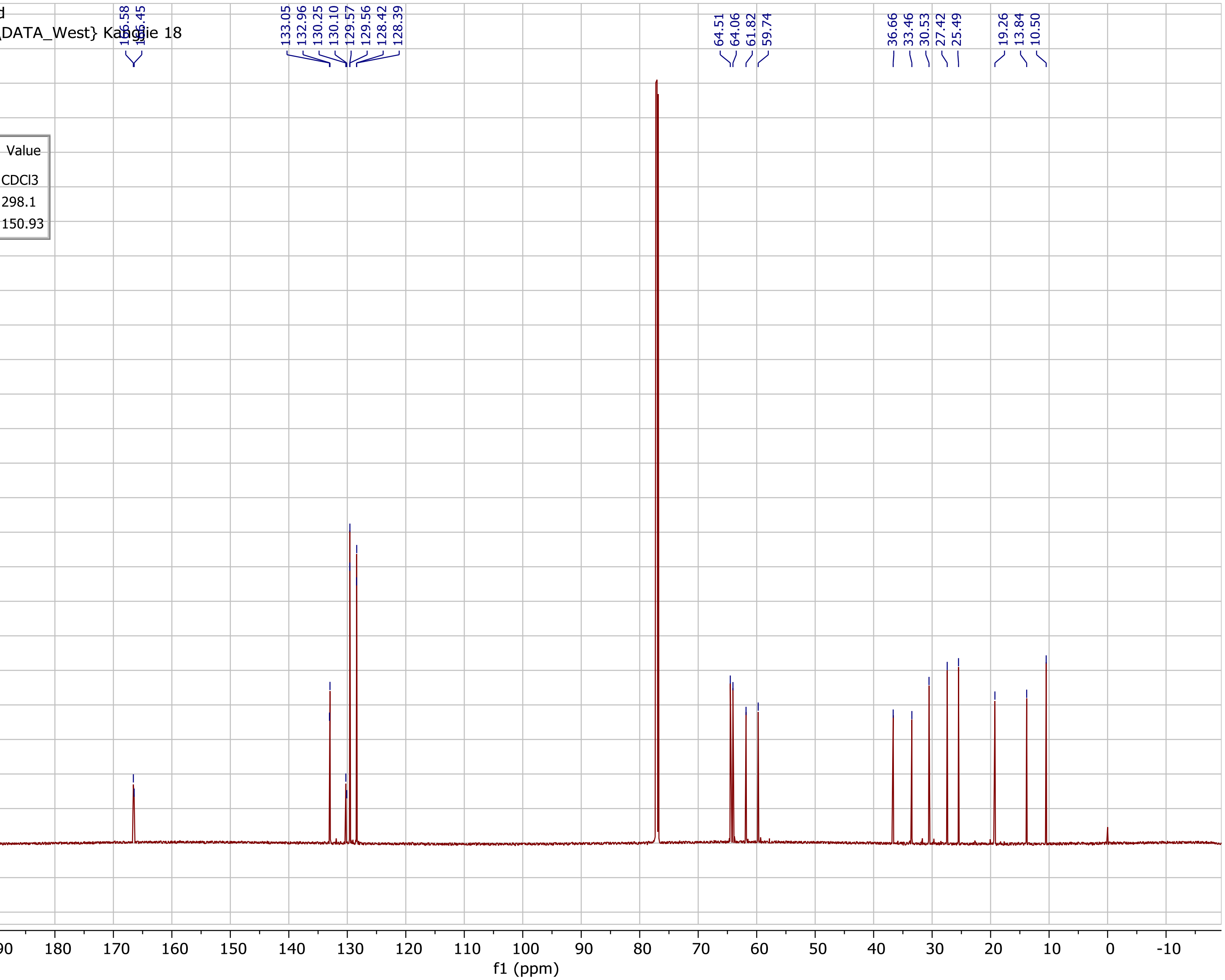

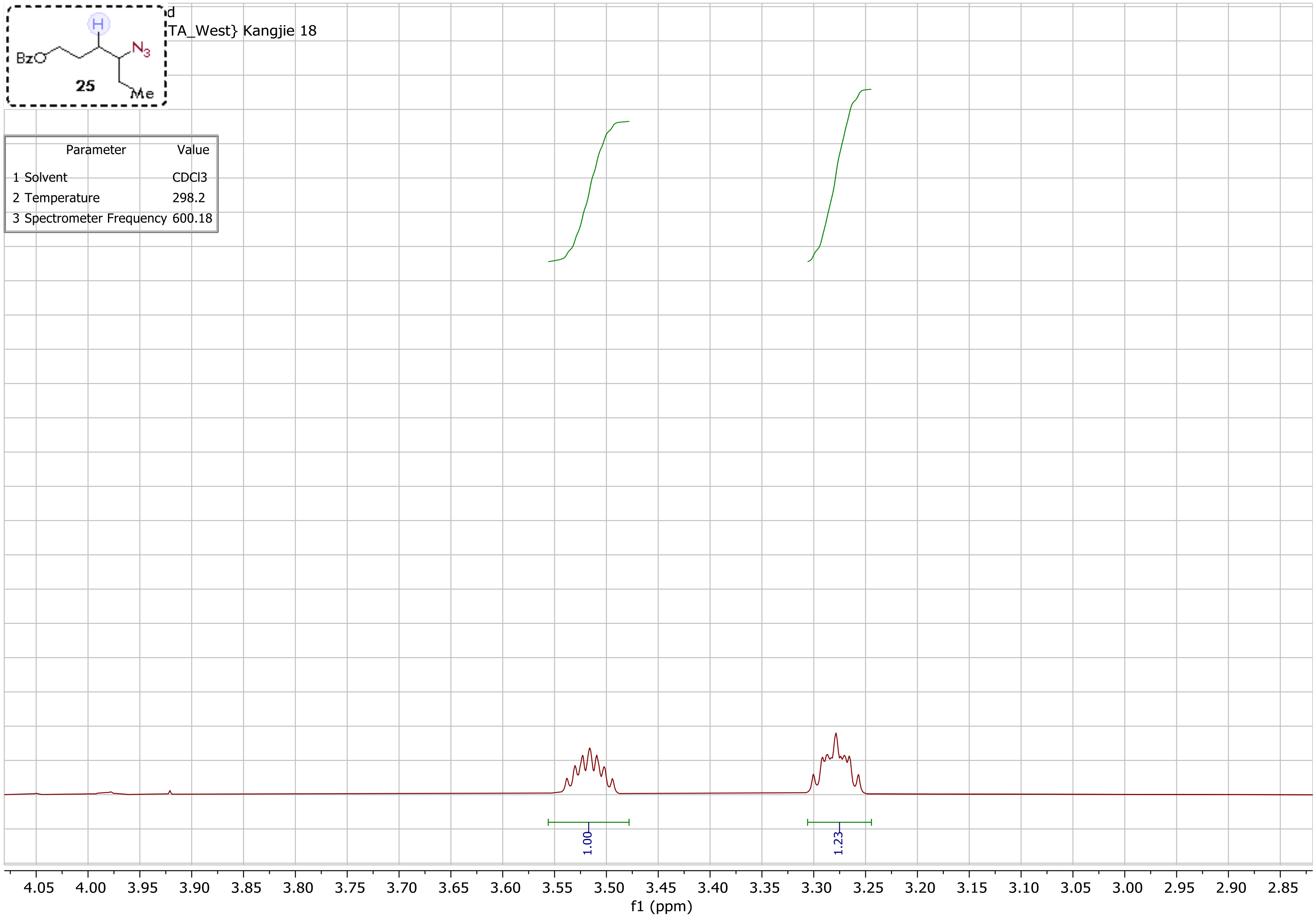
d
TA_West} Kangjie 18
BzO
N3
Me
25
Parameter Value
1 Solvent CDCl3
2 Temperature 298.2
3 Spectrometer Frequency 600.18
1.00
1.23
4.05 4.00 3.95 3.90 3.85 3.80 3.75 3.70 3.65 3.60 3.55 3.50 3.45 3.40 3.35 3.30 3.25 3.20 3.15 3.10 3.05 3.00 2.95 2.90 2.85
f1 (ppm)

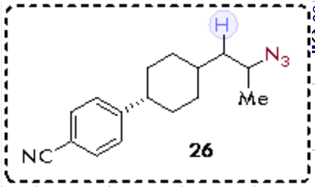

Parameter	Value
1 Solvent	CDCl3
2 Temperature	298.1
3 Spectrometer Frequency	600.18

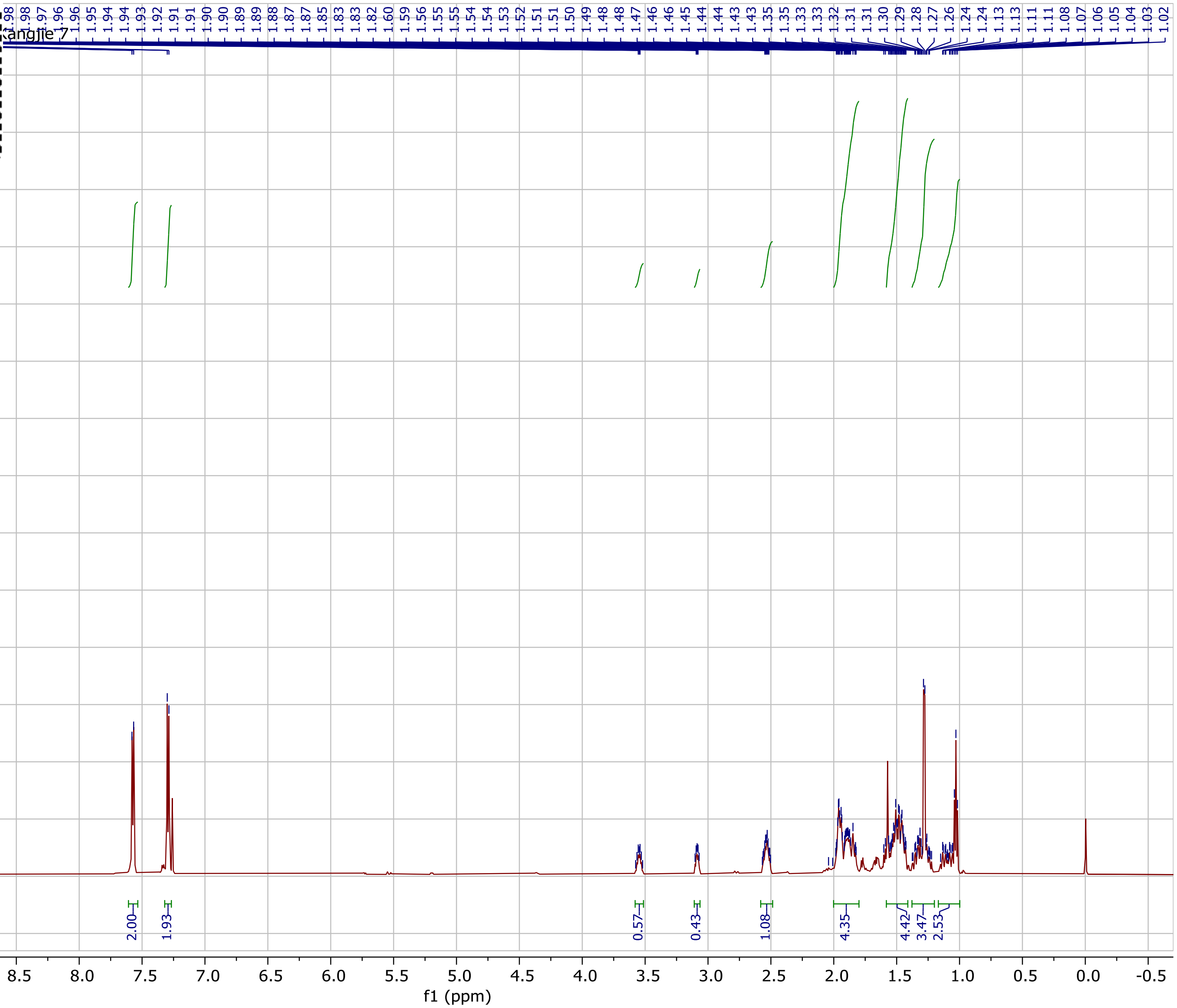

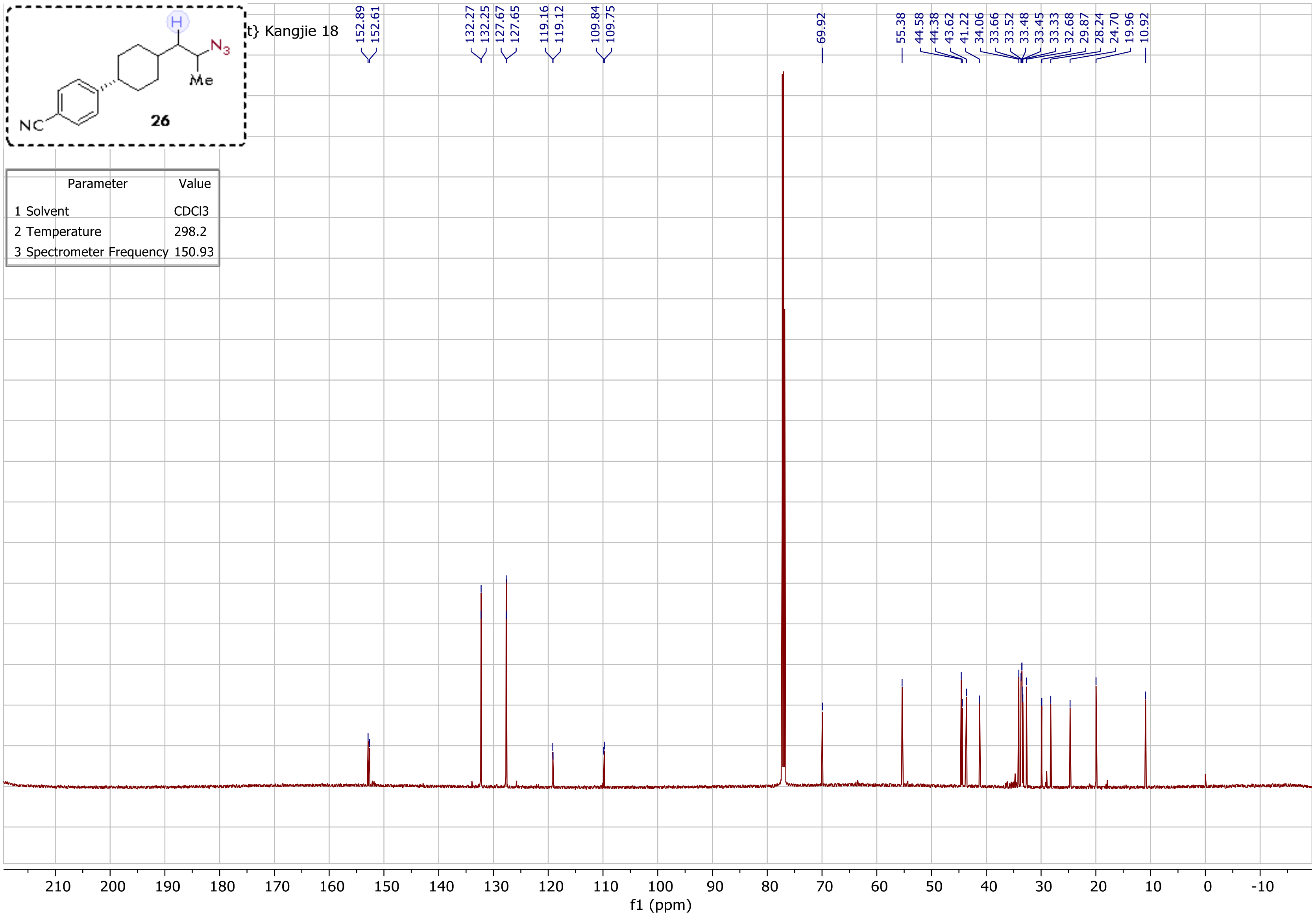
t} Kangjie 18
H
N3
Me
NC
26
Parameter Value
1 Solvent CDCl3
2 Temperature 298.2
3 Spectrometer Frequency 150.93
152.89
152.61
132.27
132.25
127.67
127.65
119.16
119.12
109.84
109.75
69.92
55.38
44.58
44.38
43.62
41.22
34.06
33.66
33.52
33.48
33.45
33.33
32.68
29.87
28.24
24.70
19.96
10.92
210 200 190 180 170 160 150 140 130 120 110 100 90 80 70 60 50 40 30 20 10 0 -10
f1 (ppm)

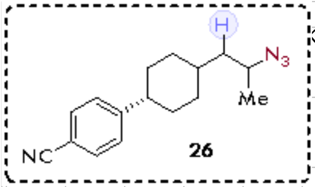

Parameter	Value
1 Solvent	CDCl3
2 Temperature	298.1
3 Spectrometer Frequency	600.18

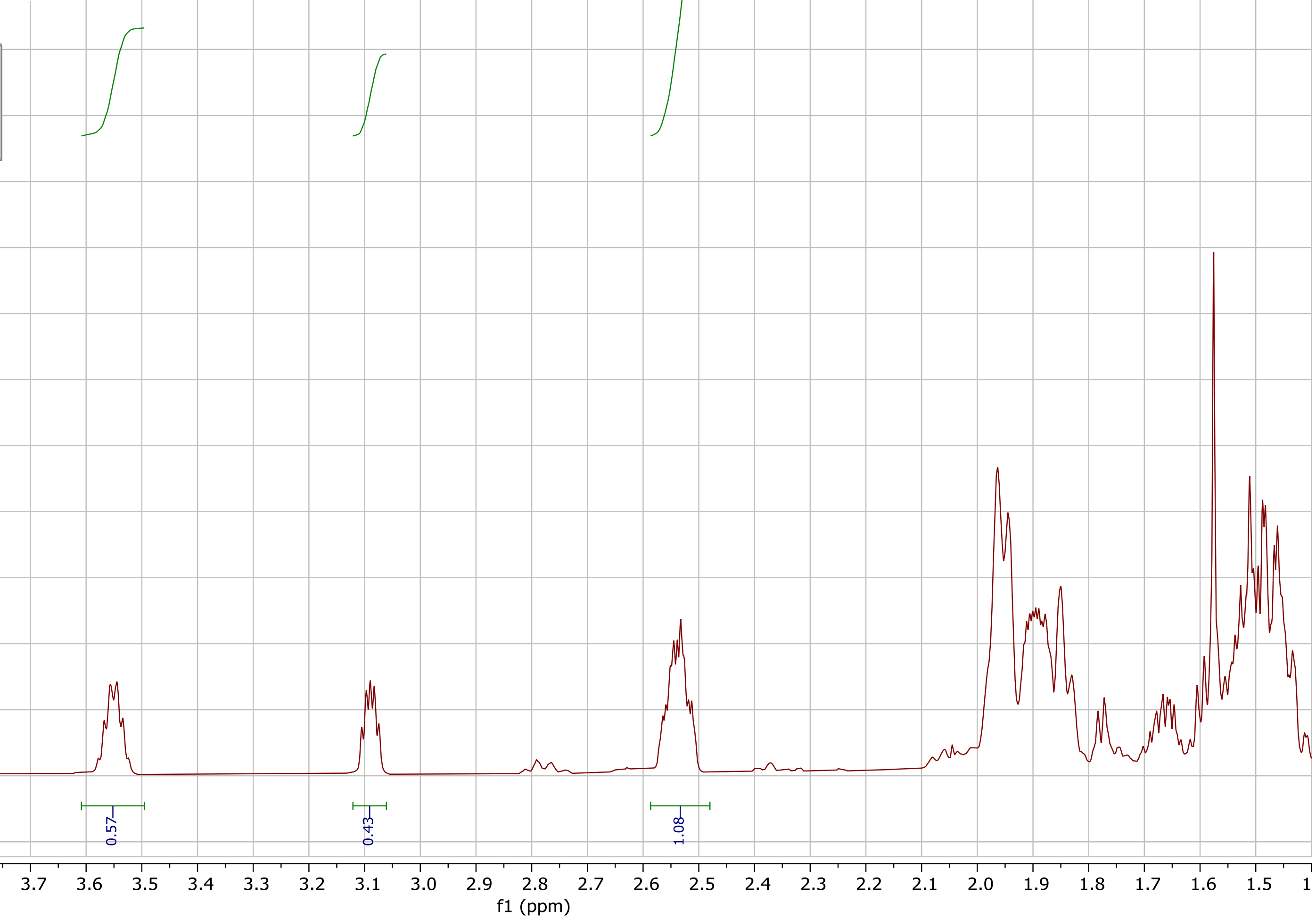

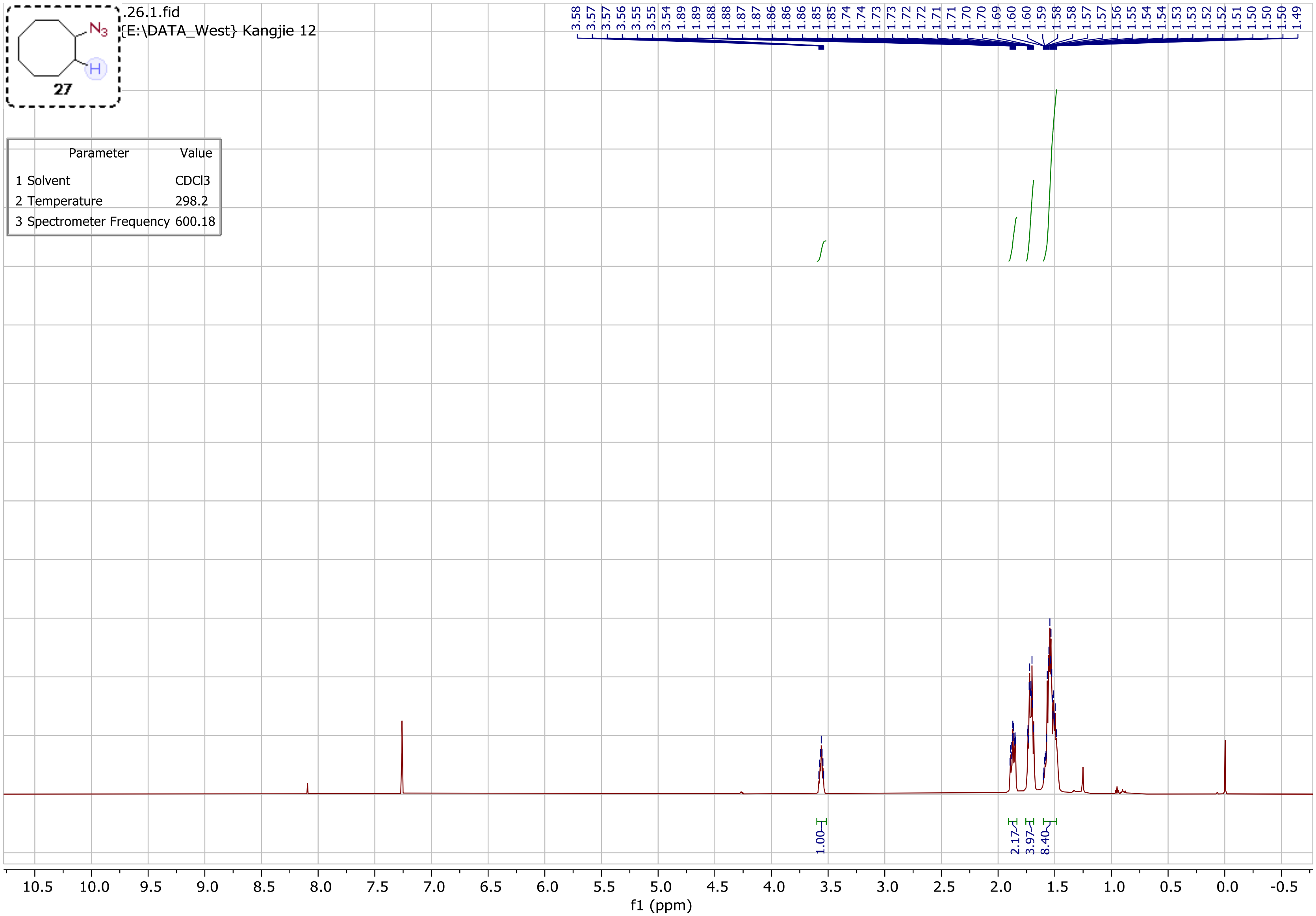
.26.1.fid
{E:\DATA_West} Kangjie 12
N3
H
27
Parameter Value
1 Solvent CDCl3
2 Temperature 298.2
3 Spectrometer Frequency 600.18
3.58 3.57 3.57 3.56 3.55 3.55 3.54 1.89 1.89 1.88 1.88 1.87 1.87 1.86 1.86 1.86 1.85 1.85 1.74 1.74 1.73 1.73 1.72 1.72 1.71 1.71 1.70 1.69 1.60 1.60 1.59 1.58 1.58 1.58 1.57 1.57 1.56 1.55 1.54 1.54 1.53 1.53 1.52 1.52 1.51 1.50 1.50 1.50 1.49
1.00
2.17
3.97
8.40
10.5 10.0 9.5 9.0 8.5 8.0 7.5 7.0 6.5 6.0 5.5 5.0 4.5 4.0 3.5 3.0 2.5 2.0 1.5 1.0 0.5 0.0 -0.5
f1 (ppm)

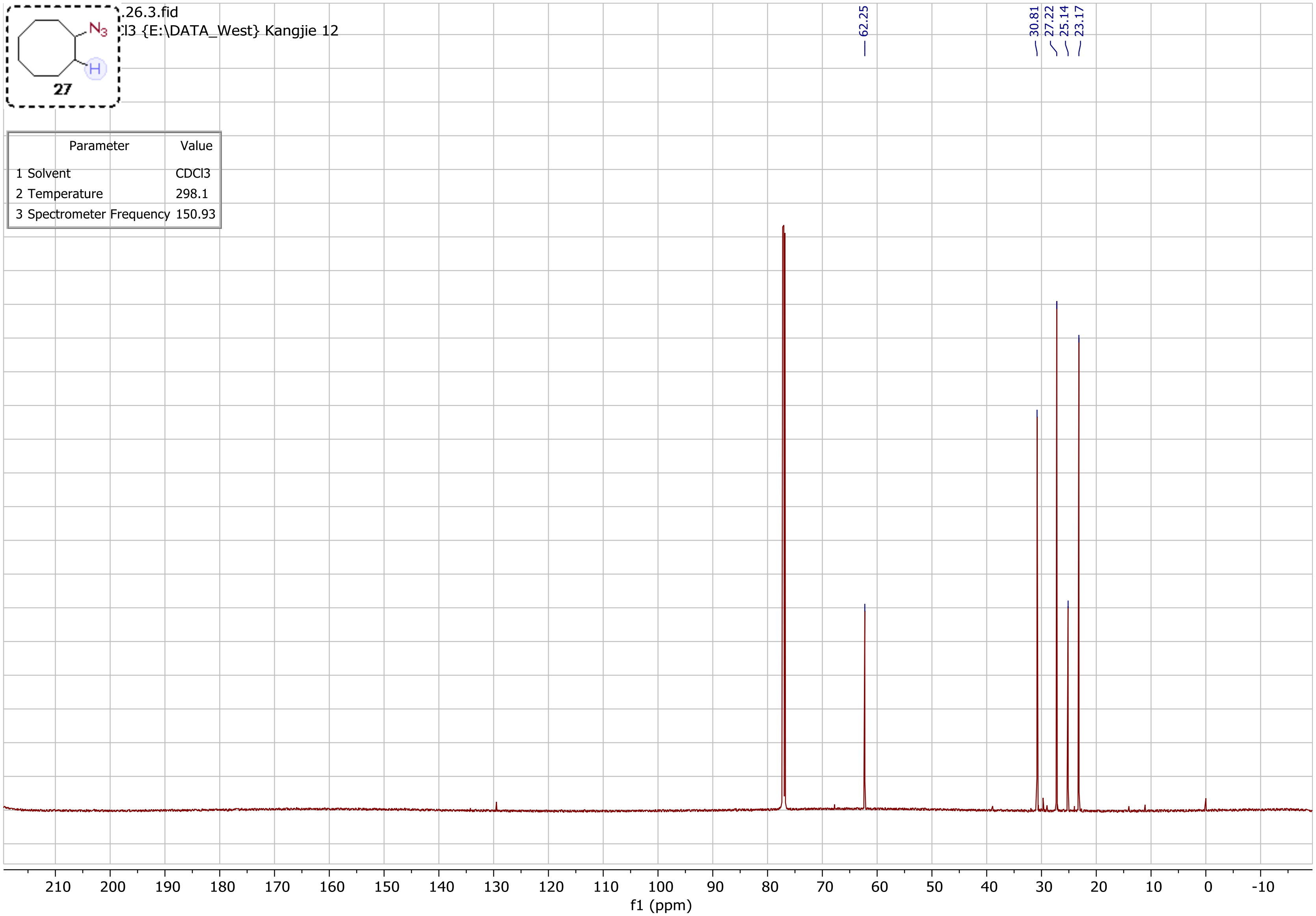

.26.3.fid
l3 {E:\DATA_West} Kangjie 12
N3
H
27
Parameter Value
1 Solvent CDCl3
2 Temperature 298.1
3 Spectrometer Frequency 150.93
62.25
30.81
27.22
25.14
23.17
210 200 190 180 170 160 150 140 130 120 110 100 90 80 70 60 50 40 30 20 10 0 -10
f1 (ppm)

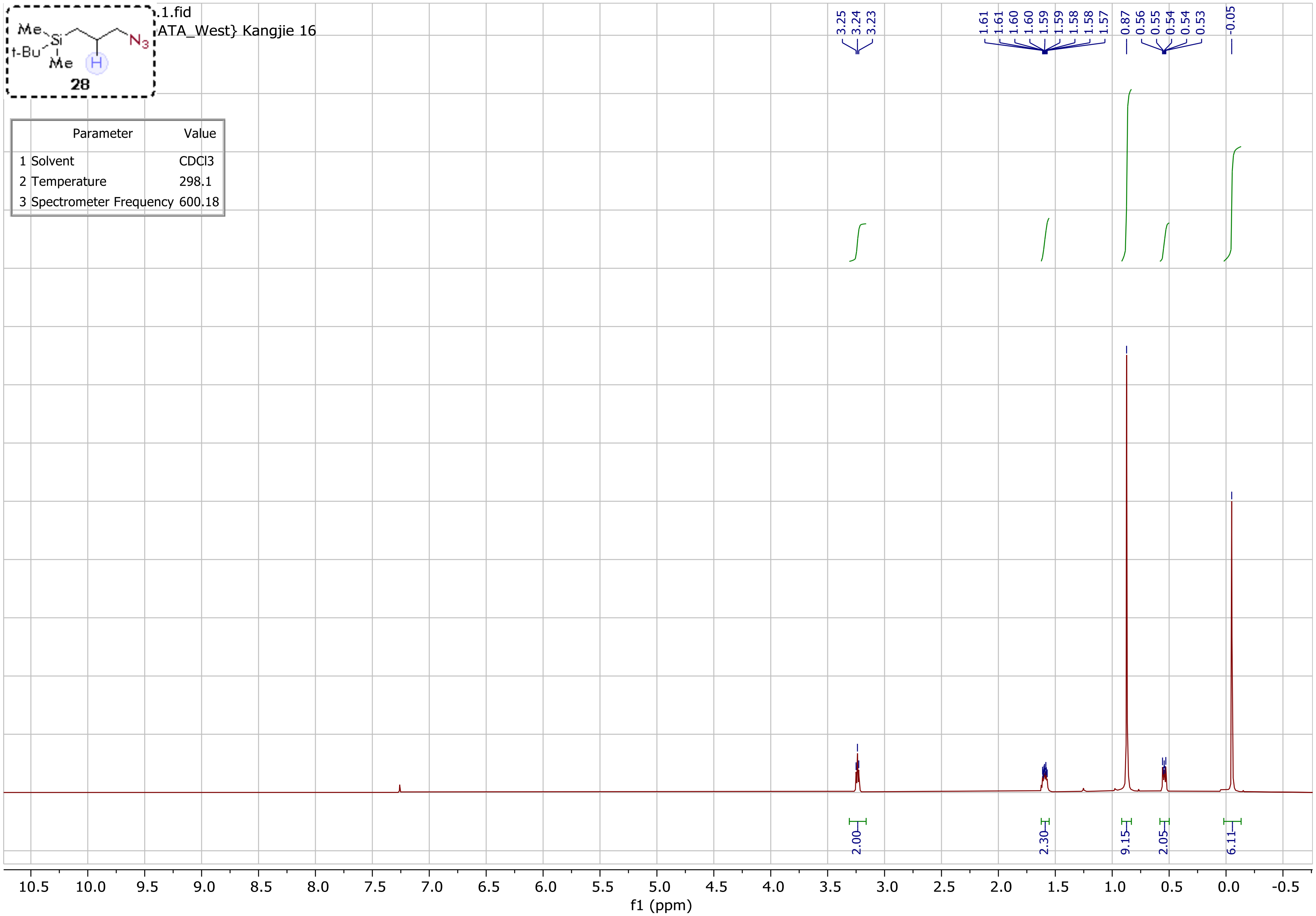

	Parameter	Value
1	Solvent	CDCl3
2	Temperature	298.1
3	Spectrometer Frequency	600.18

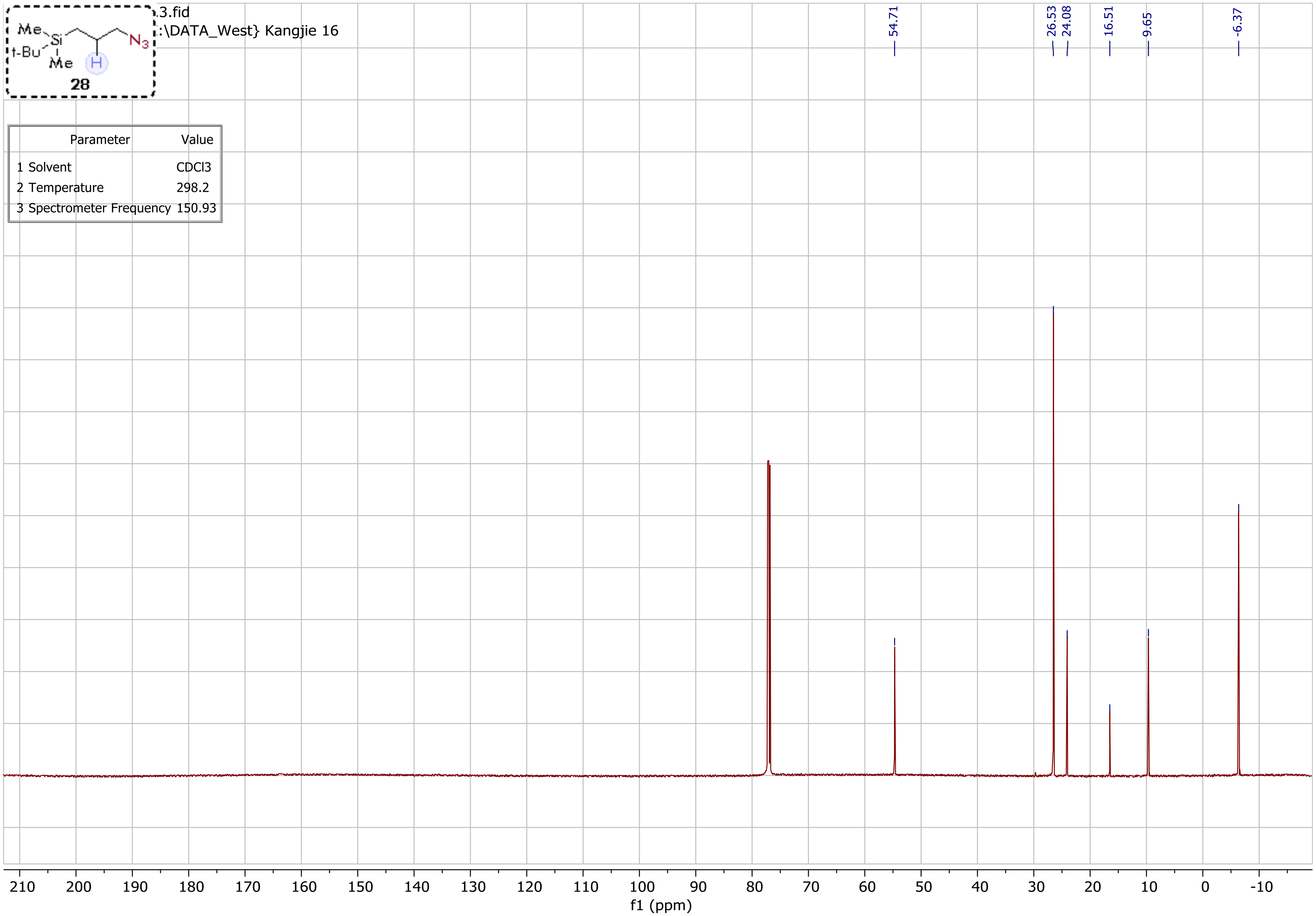
Me
Si
t-Bu
Me
H
N_3
28
.3.fid
:\DATA_West} Kangjie 16
Parameter Value
1 Solvent CDCl3
2 Temperature 298.2
3 Spectrometer Frequency 150.93
54.71
26.53
24.08
16.51
9.65
-6.37
210 200 190 180 170 160 150 140 130 120 110 100 90 80 70 60 50 40 30 20 10 0 -10
f1 (ppm)

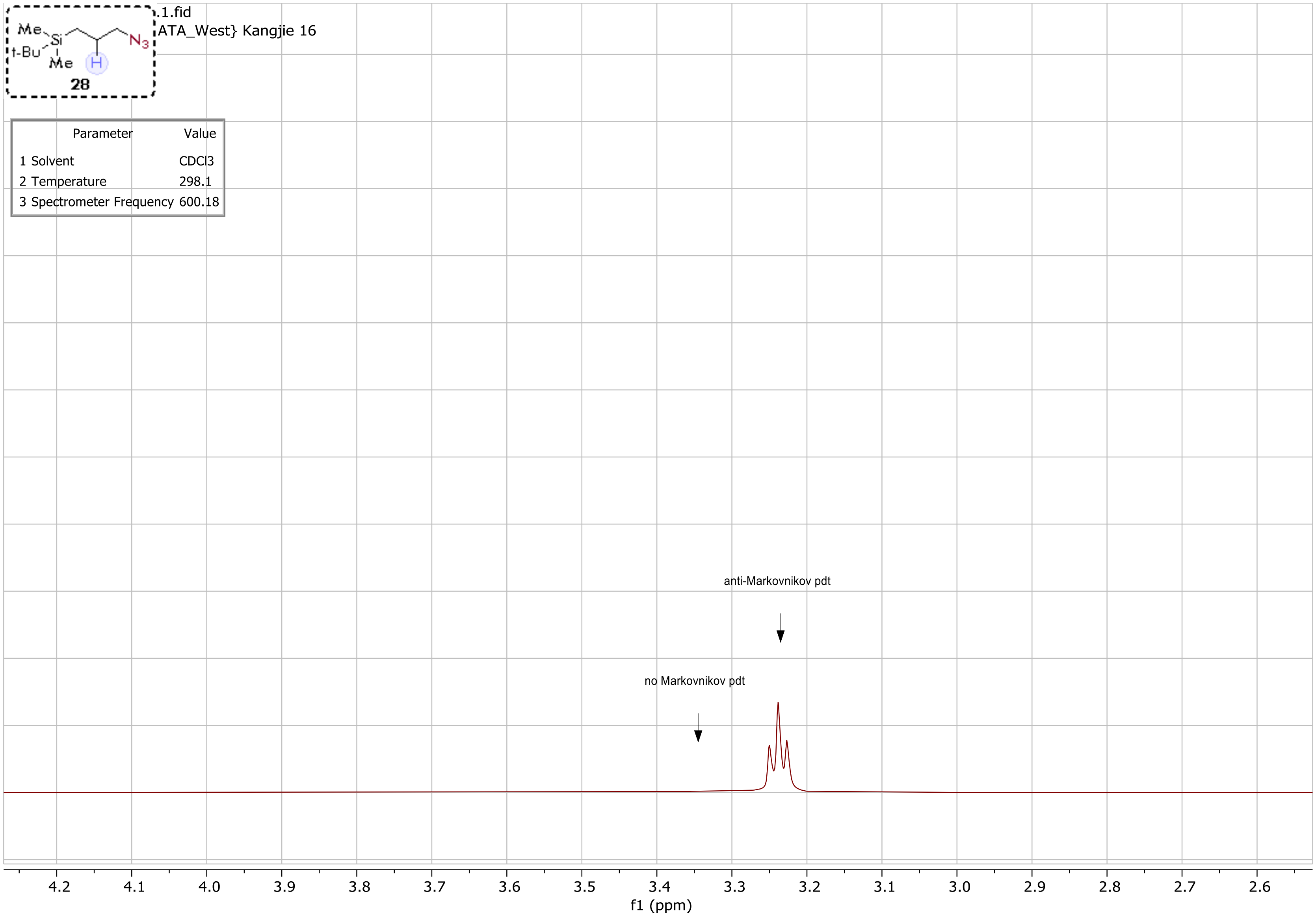
.1.fid
ATA_West} Kangjie 16
Me
Si
t-Bu
Me
H
N_3
28
Parameter Value
1 Solvent CDCl3
2 Temperature 298.1
3 Spectrometer Frequency 600.18
anti-Markovnikov pdt
no Markovnikov pdt
4.2 4.1 4.0 3.9 3.8 3.7 3.6 3.5 3.4 3.3 3.2 3.1 3.0 2.9 2.8 2.7 2.6
f1 (ppm)

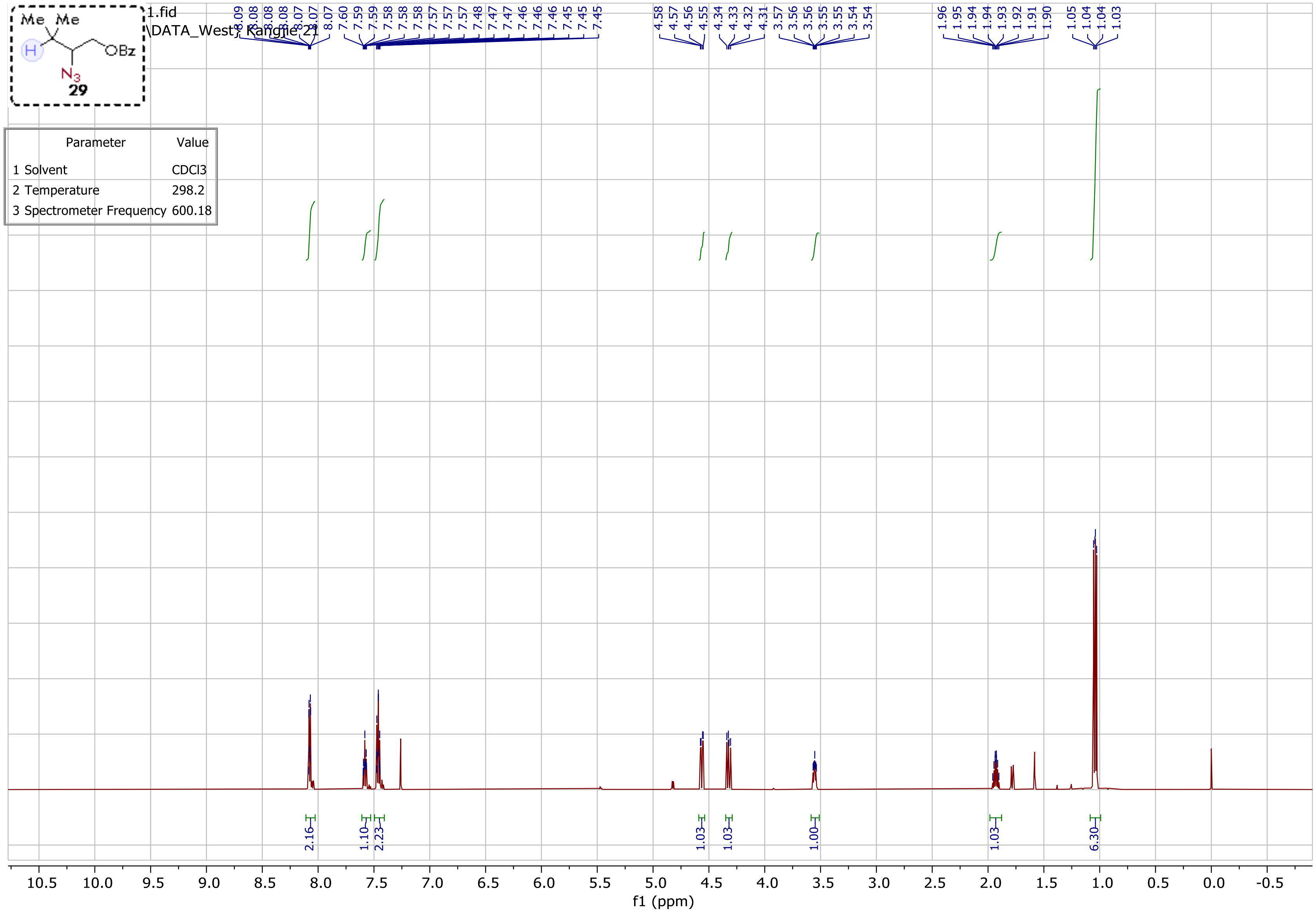

Me Me
H
OBz
N3
29
1.fid
\DATA_West\Kangjie 21
Parameter | Value
1 Solvent | CDCl3
2 Temperature | 298.2
3 Spectrometer Frequency | 600.18
8.09 8.08 8.08 8.08 8.07 8.07 8.07 7.60 7.59 7.59 7.58 7.58 7.58 7.57 7.57 7.57 7.48 7.47 7.47 7.46 7.46 7.46 7.45 7.45 7.45
4.58 4.57 4.56 4.55 4.34 4.33 4.32 4.31 3.57 3.56 3.56 3.55 3.55 3.54 3.54
1.96 1.95 1.94 1.94 1.93 1.92 1.91 1.90 1.05 1.04 1.04 1.03
2.16 1.10 2.23 1.03 1.03 1.00 1.03 6.30
10.5 10.0 9.5 9.0 8.5 8.0 7.5 7.0 6.5 6.0 5.5 5.0 4.5 4.0 3.5 3.0 2.5 2.0 1.5 1.0 0.5 0.0 -0.5
f1 (ppm)

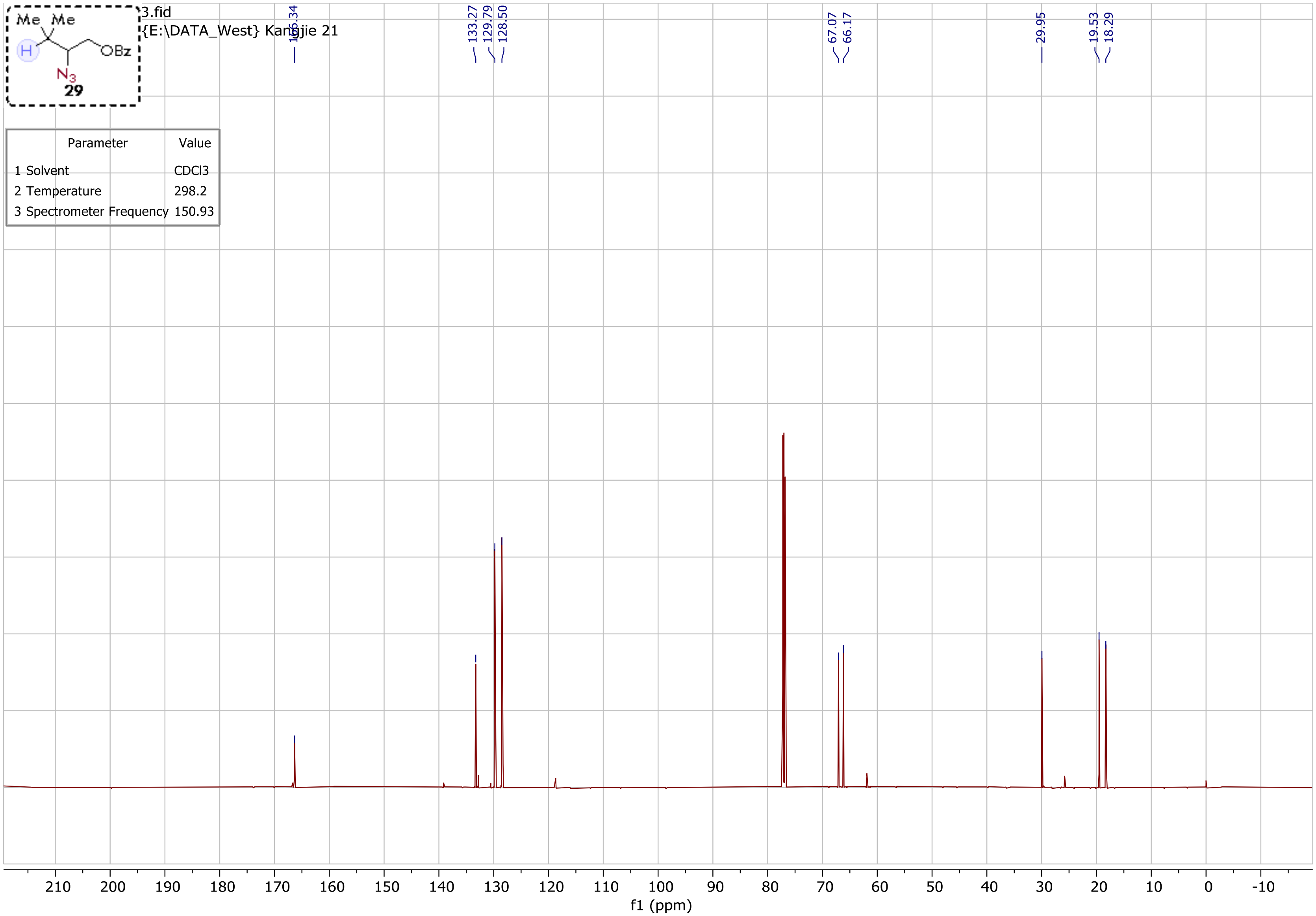

	Parameter	Value
1	Solvent	CDCl3
2	Temperature	298.2
3	Spectrometer Frequency	150.93

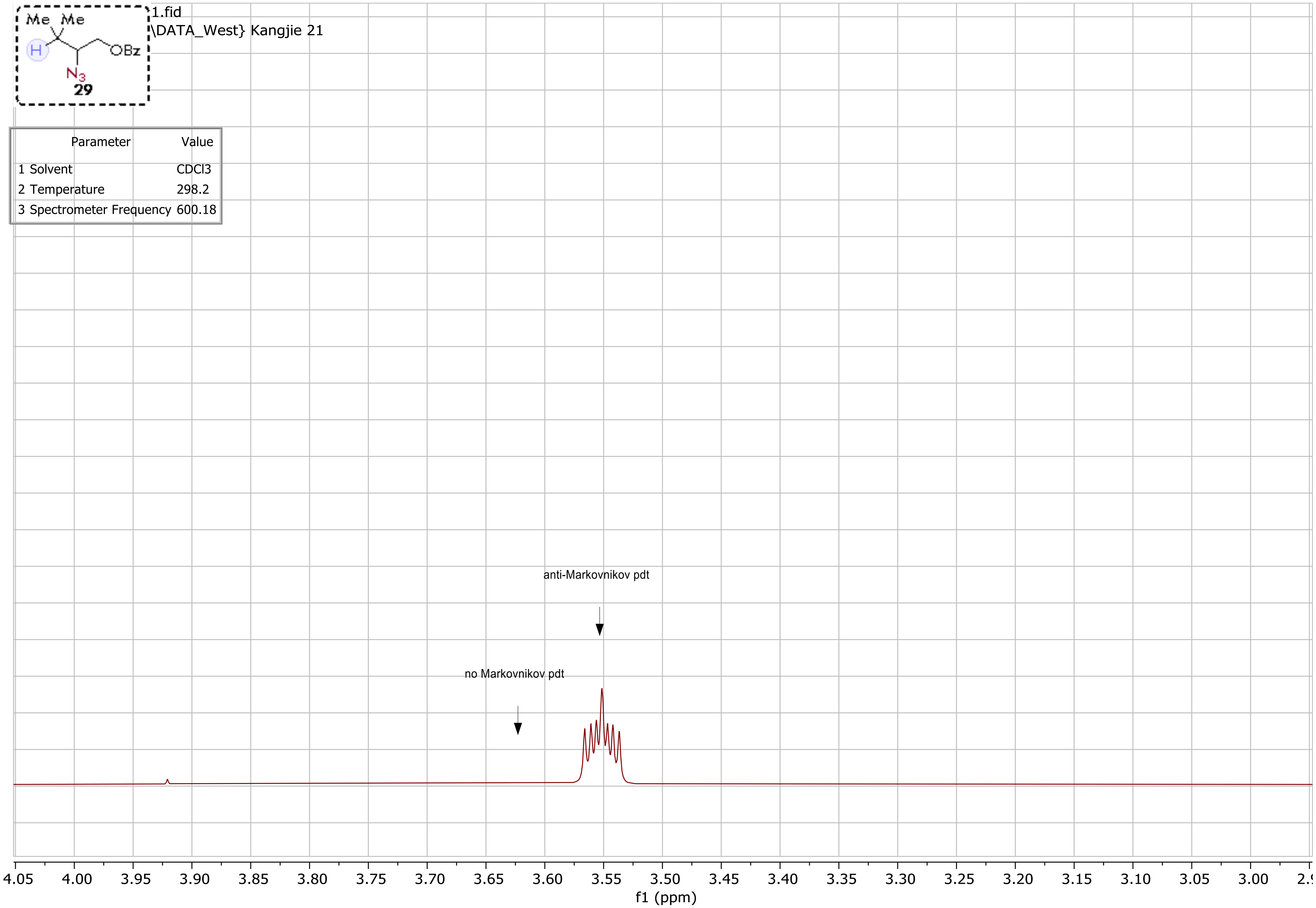

Parameter	Value
1 Solvent	CDCl3
2 Temperature	298.2
3 Spectrometer Frequency	600.18

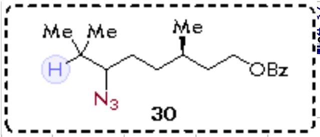

Parameter	Value
1 Solvent	CDCl3
2 Temperature	298.2
3 Spectrometer Frequency	600.18

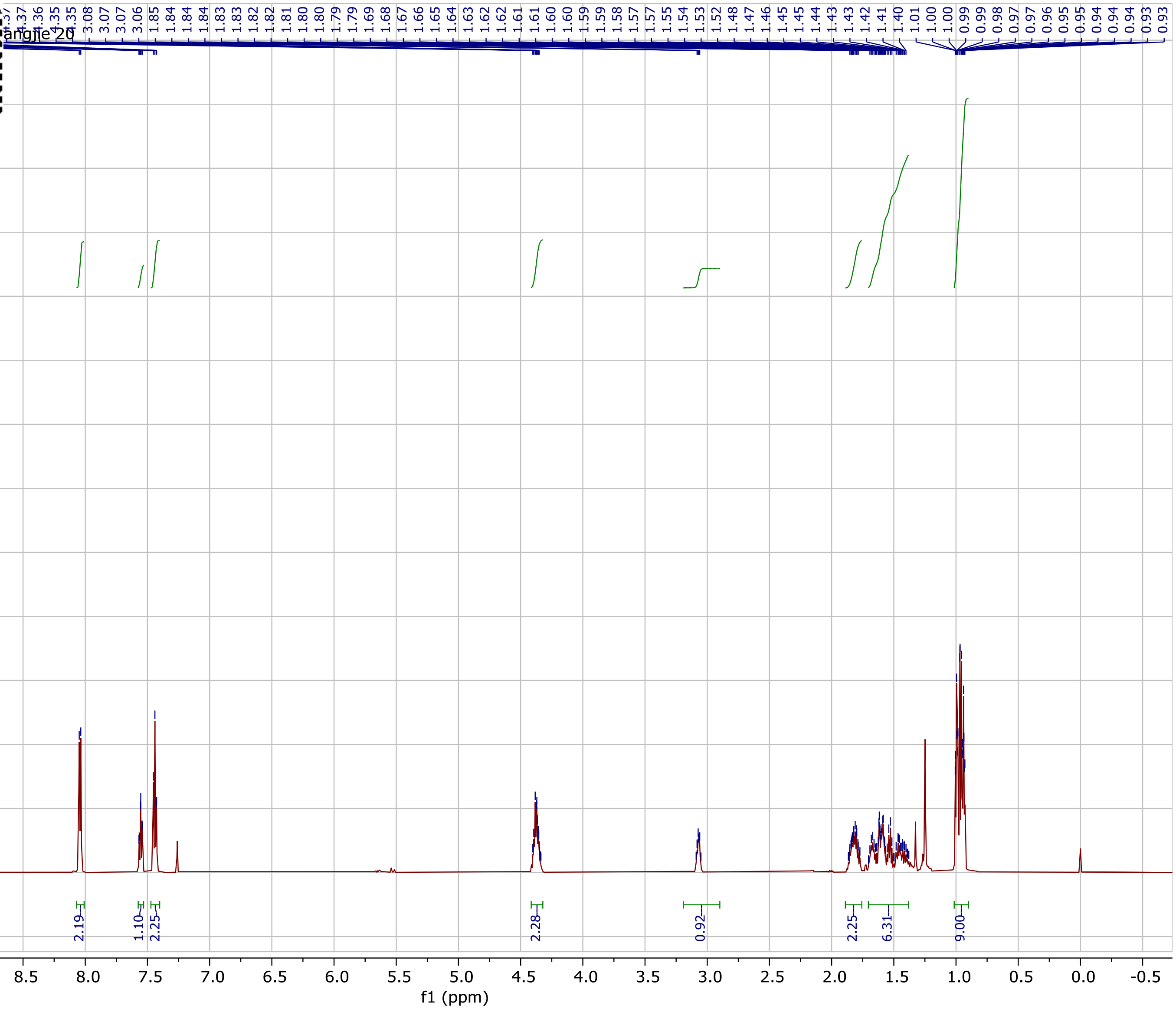

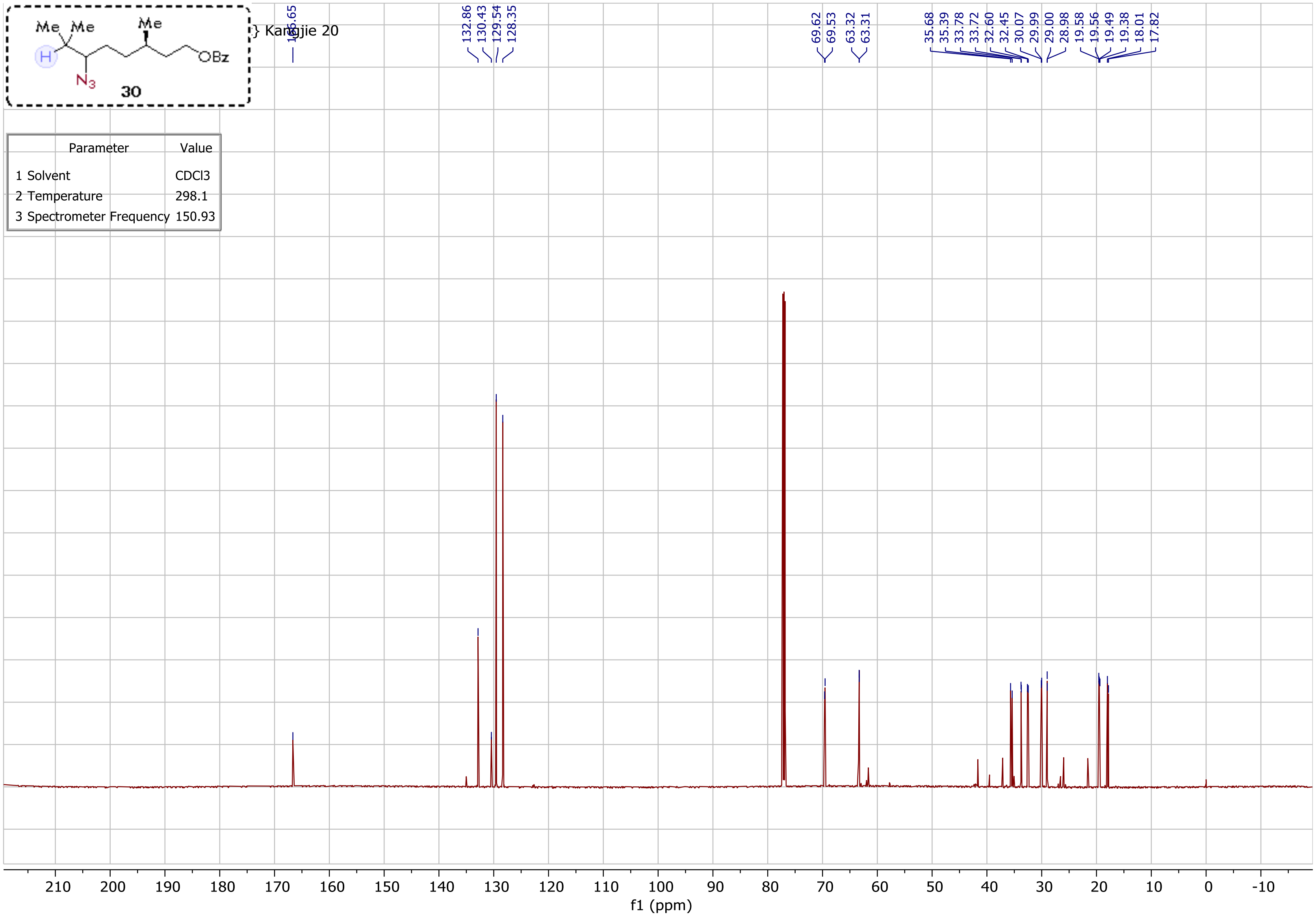

Parameter	Value
1 Solvent	CDCl3
2 Temperature	298.1
3 Spectrometer Frequency	150.93

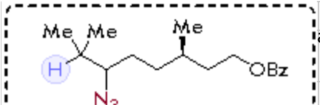

30

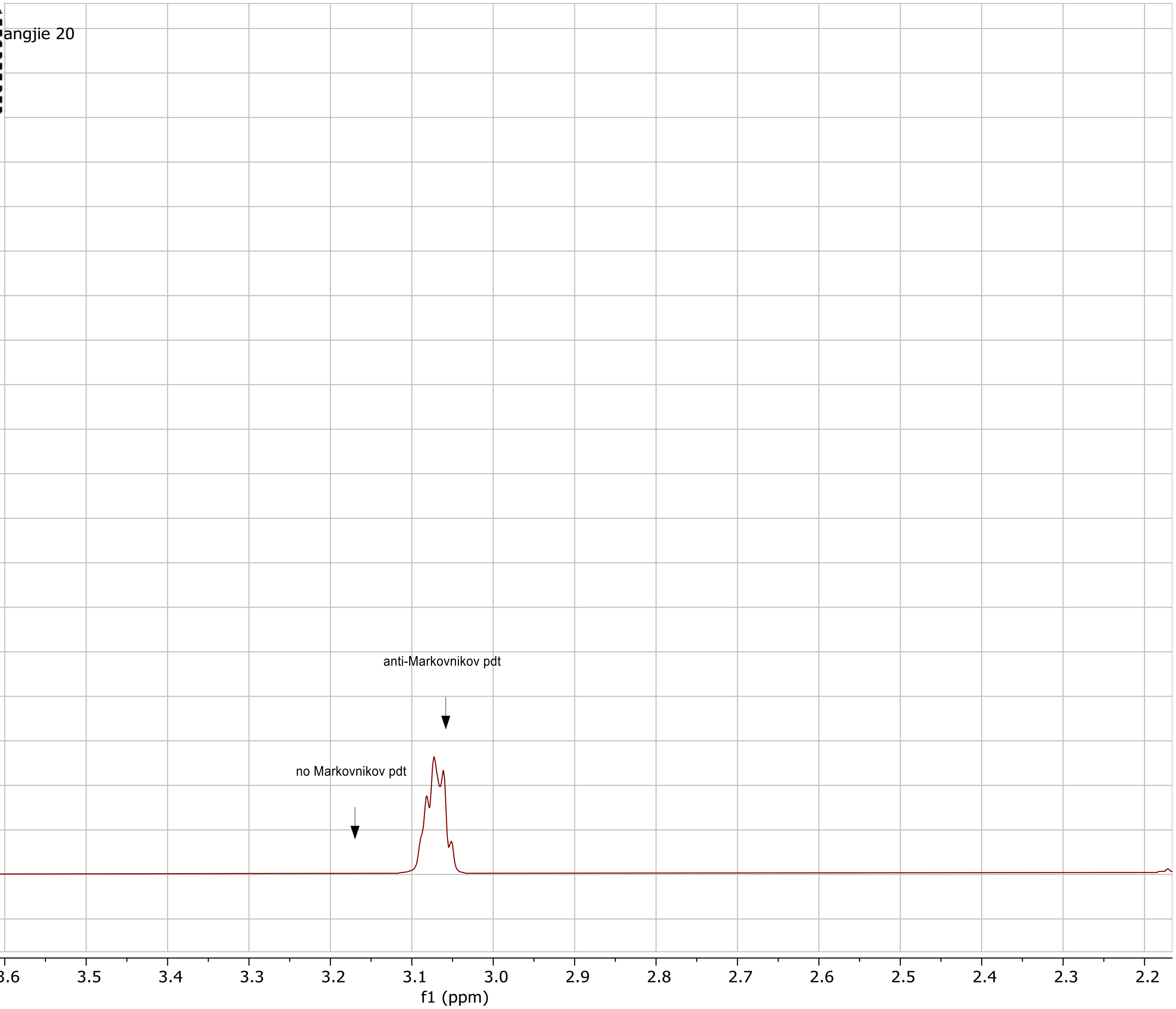

	Parameter	Value
1	Solvent	CDCl3
2	Temperature	298.2
3	Spectrometer Frequency	600.18

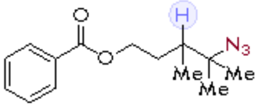

31

Parameter	Value
1 Solvent	CDCl3
2 Temperature	298.0
3 Spectrometer Frequency	599.95

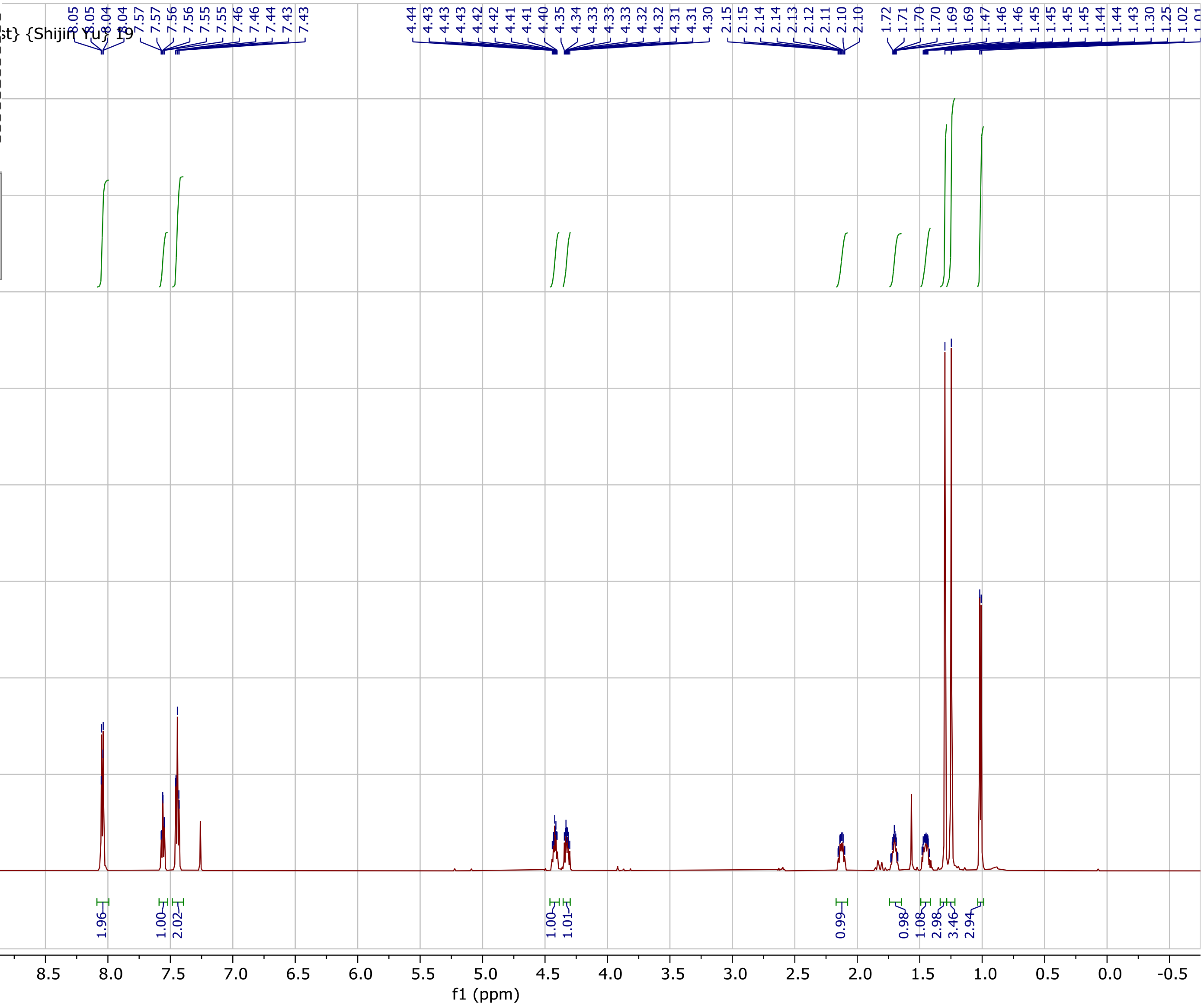

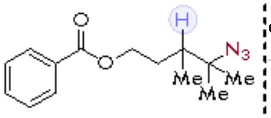

31

Parameter	Value
1 Solvent	CDCl3
2 Temperature	298.0
3 Spectrometer Frequency	150.87

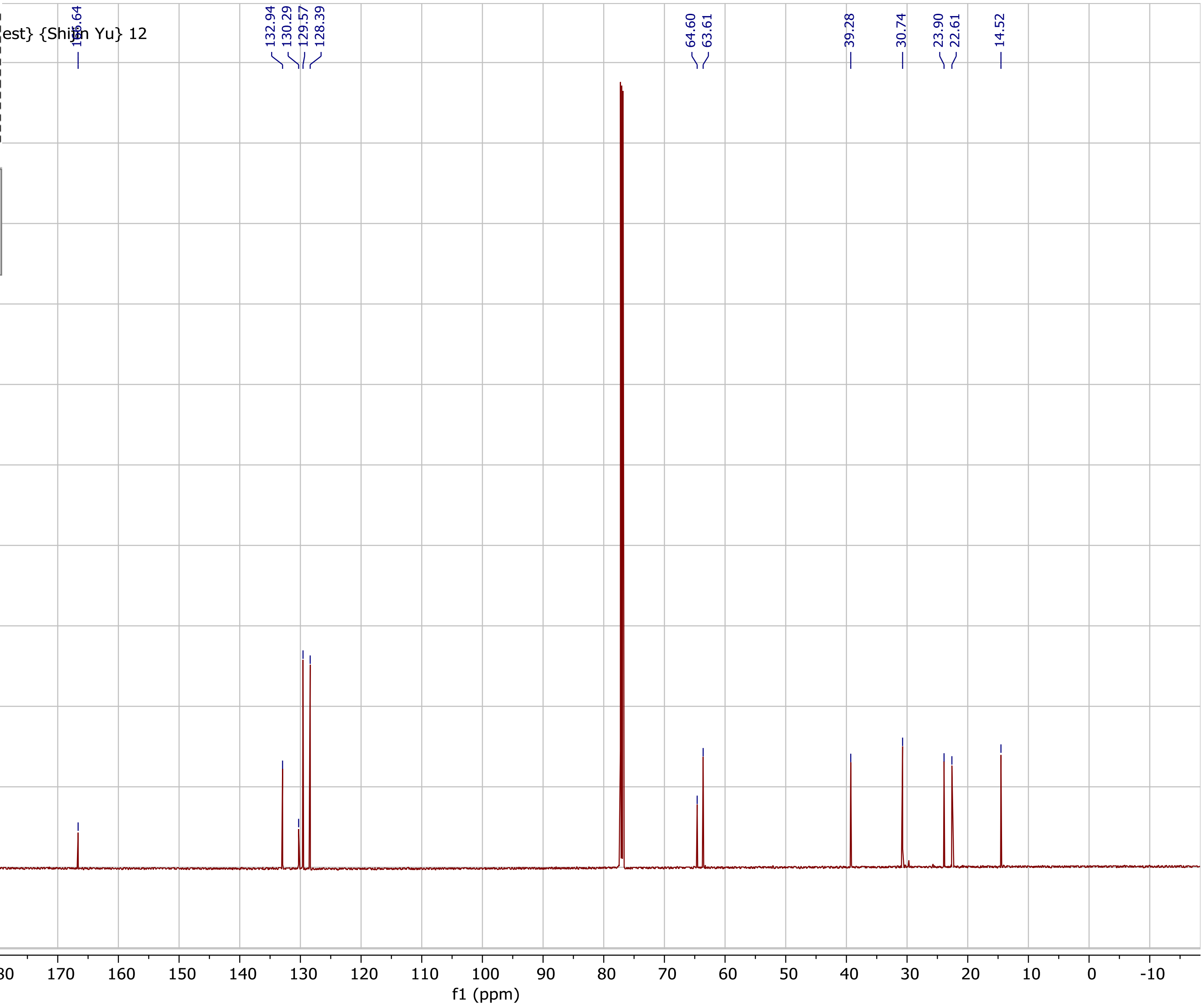

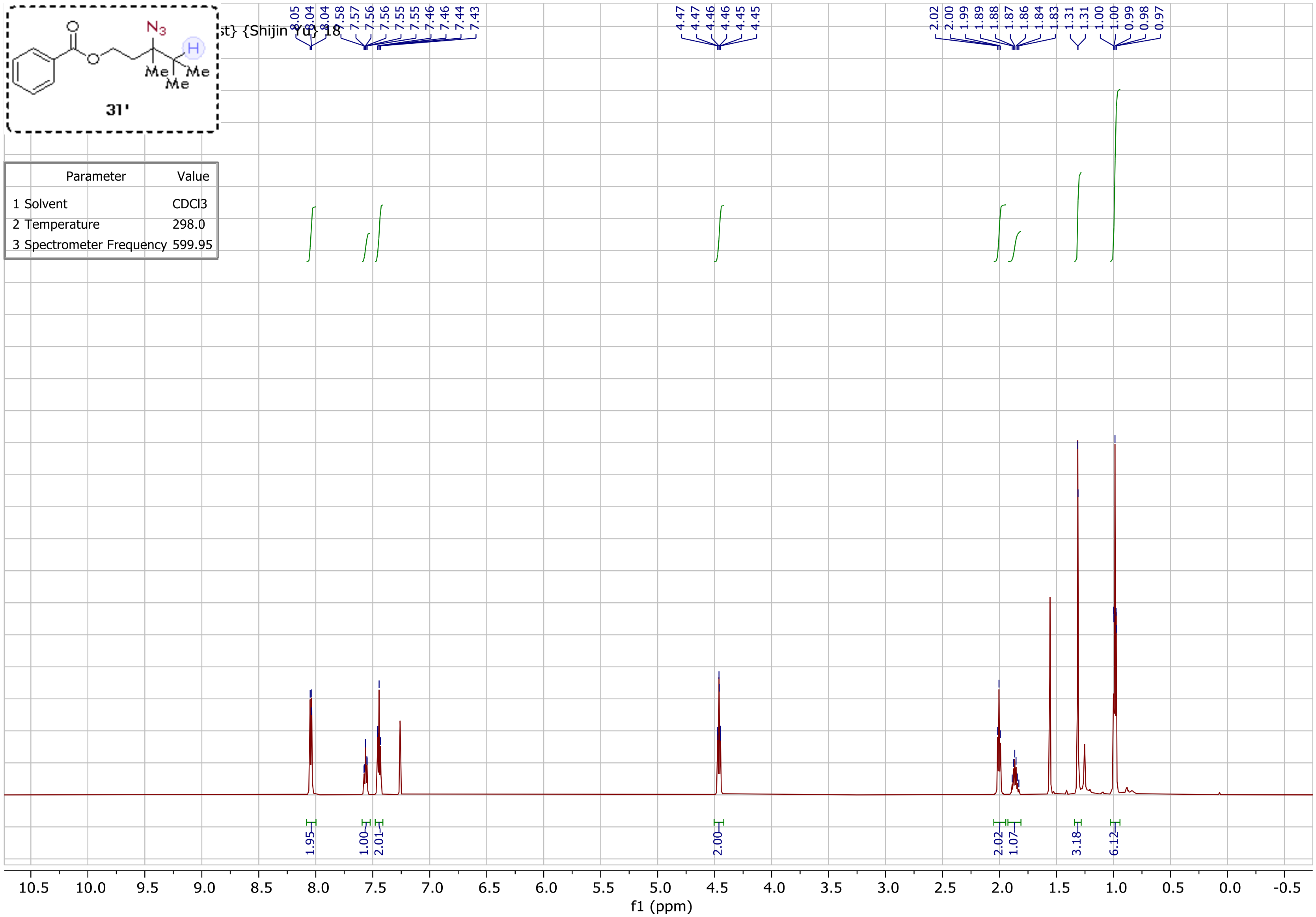

N3
H
Me
Me
Me
31'
Parameter Value
1 Solvent CDCl3
2 Temperature 298.0
3 Spectrometer Frequency 599.95
8.05 8.04 8.04 7.58 7.57 7.56 7.56 7.55 7.55 7.46 7.46 7.44 7.43
4.47 4.47 4.46 4.46 4.45 4.45
2.02 2.00 1.99 1.89 1.88 1.87 1.86 1.84 1.83 1.31 1.31 1.00 1.00 0.99 0.98 0.97
1.95 1.00 2.01 2.00 2.02 1.07 3.18 6.12
10.5 10.0 9.5 9.0 8.5 8.0 7.5 7.0 6.5 6.0 5.5 5.0 4.5 4.0 3.5 3.0 2.5 2.0 1.5 1.0 0.5 0.0 -0.5
f1 (ppm)

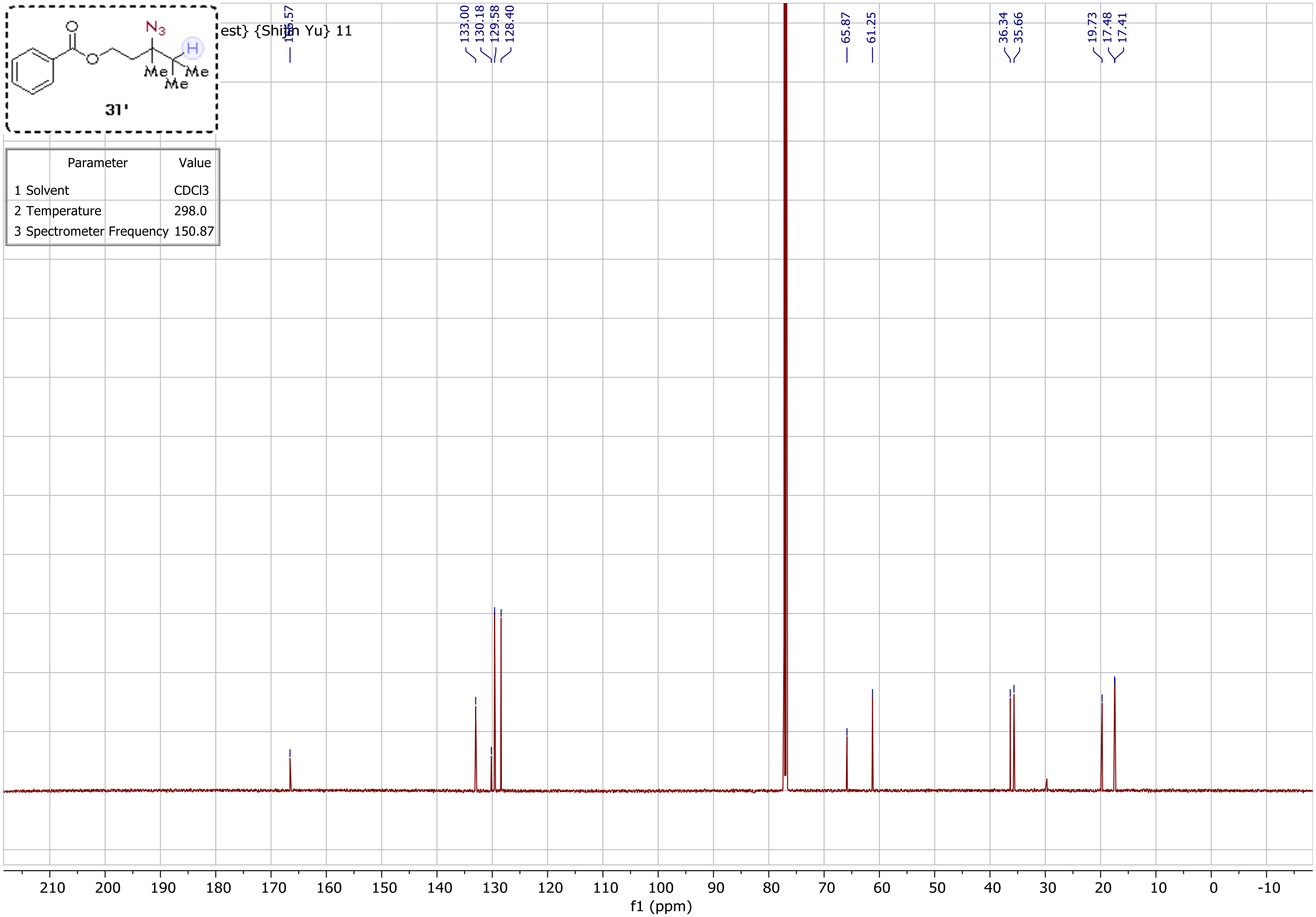

31'
Parameter | Value
1 Solvent CDCl3
2 Temperature 298.0
3 Spectrometer Frequency 150.87
133.00
130.18
129.58
128.40
65.87
61.25
36.34
35.66
19.73
17.48
17.41
f1 (ppm)

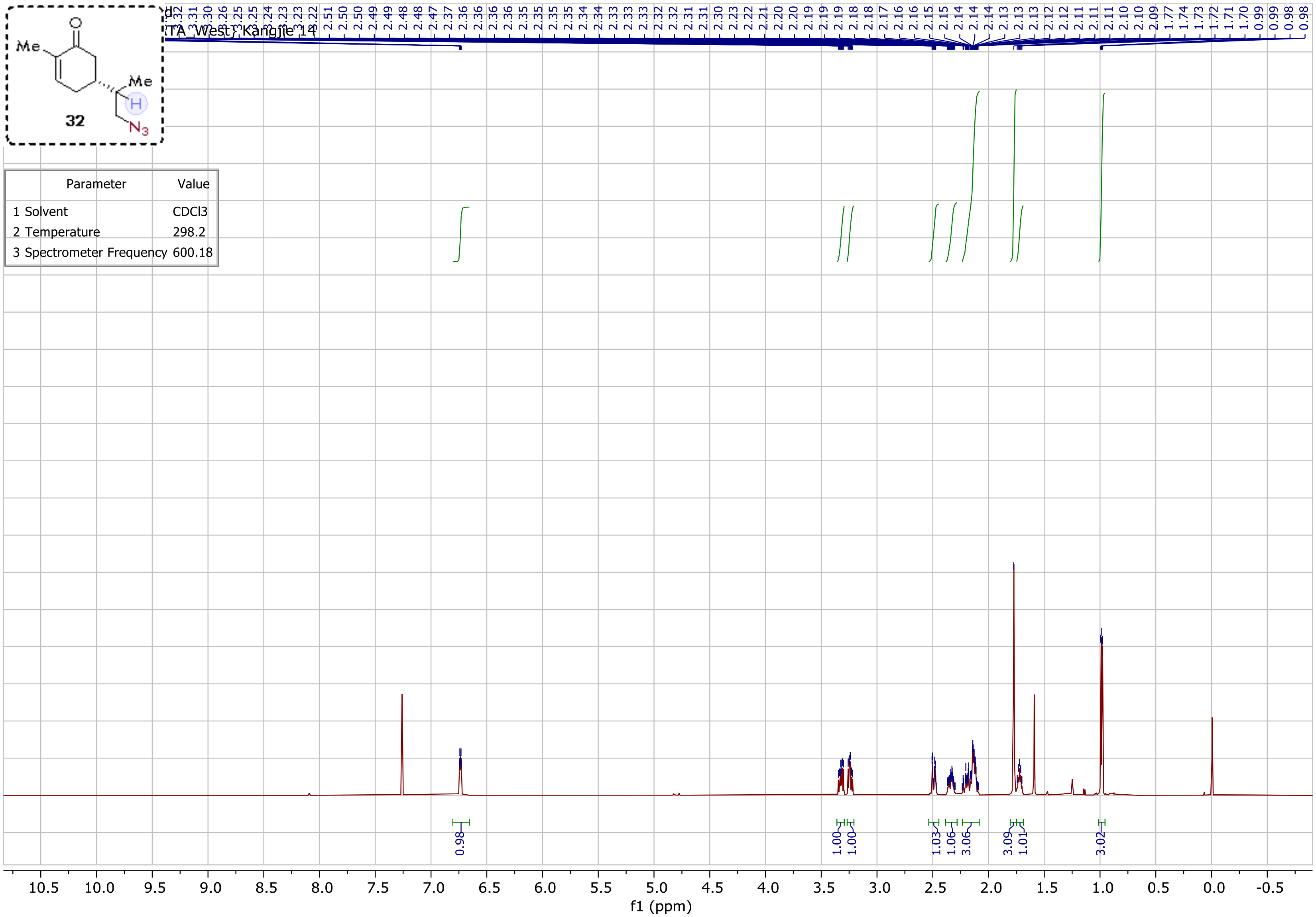

Parameter	Value
1 Solvent	CDCl3
2 Temperature	298.2
3 Spectrometer Frequency	600.18

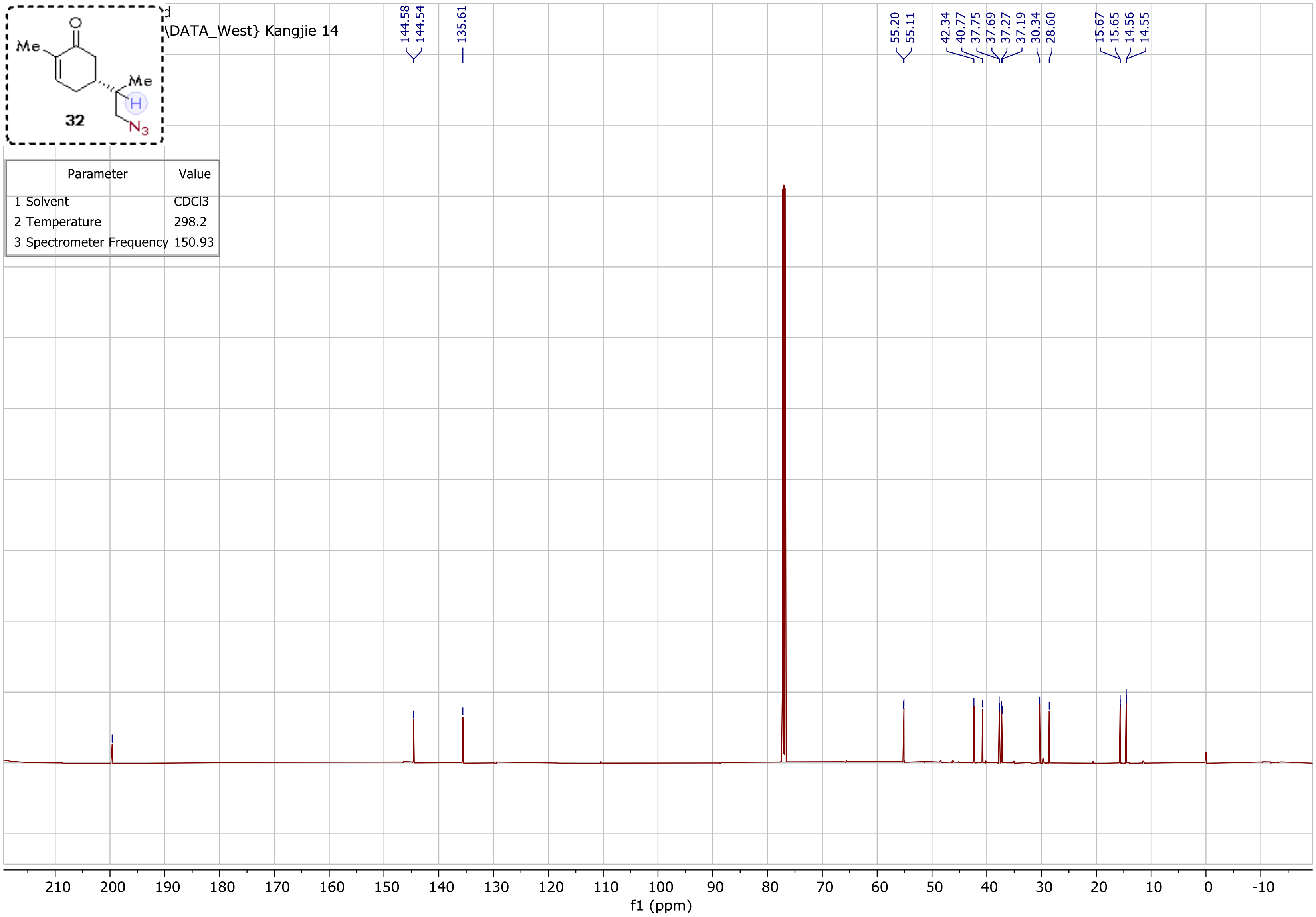

	Parameter	Value
1	Solvent	CDCl3
2	Temperature	298.2
3	Spectrometer Frequency	150.93

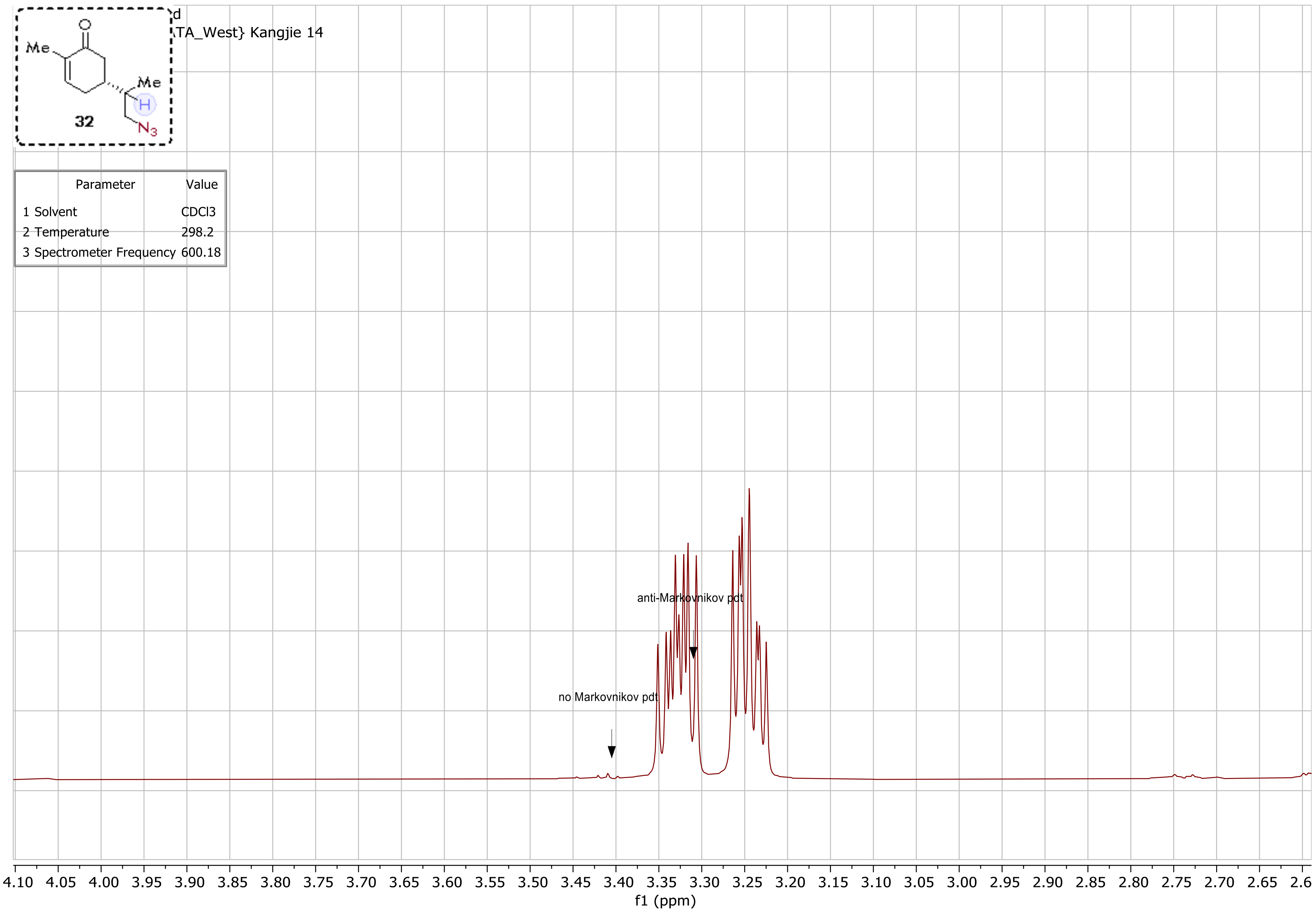
d
TA_West} Kangjie 14
Me
Me
H
N3
32
Parameter Value
1 Solvent CDCl3
2 Temperature 298.2
3 Spectrometer Frequency 600.18
anti-Markovnikov pdt
no Markovnikov pdt
4.10 4.05 4.00 3.95 3.90 3.85 3.80 3.75 3.70 3.65 3.60 3.55 3.50 3.45 3.40 3.35 3.30 3.25 3.20 3.15 3.10 3.05 3.00 2.95 2.90 2.85 2.80 2.75 2.70 2.65 2.6
f1 (ppm)

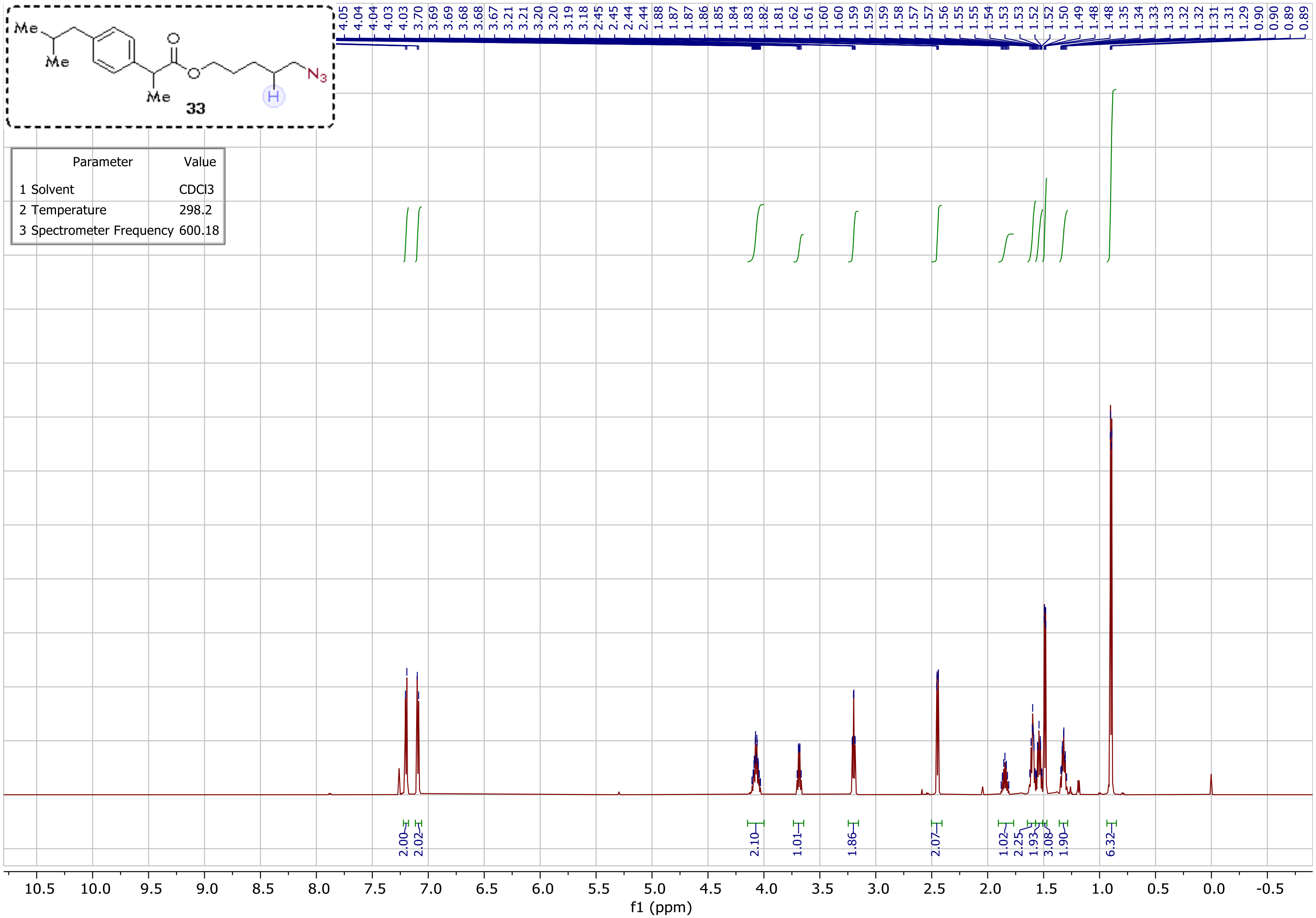

Parameter	Value
1 Solvent	CDCl3
2 Temperature	298.2
3 Spectrometer Frequency	600.18

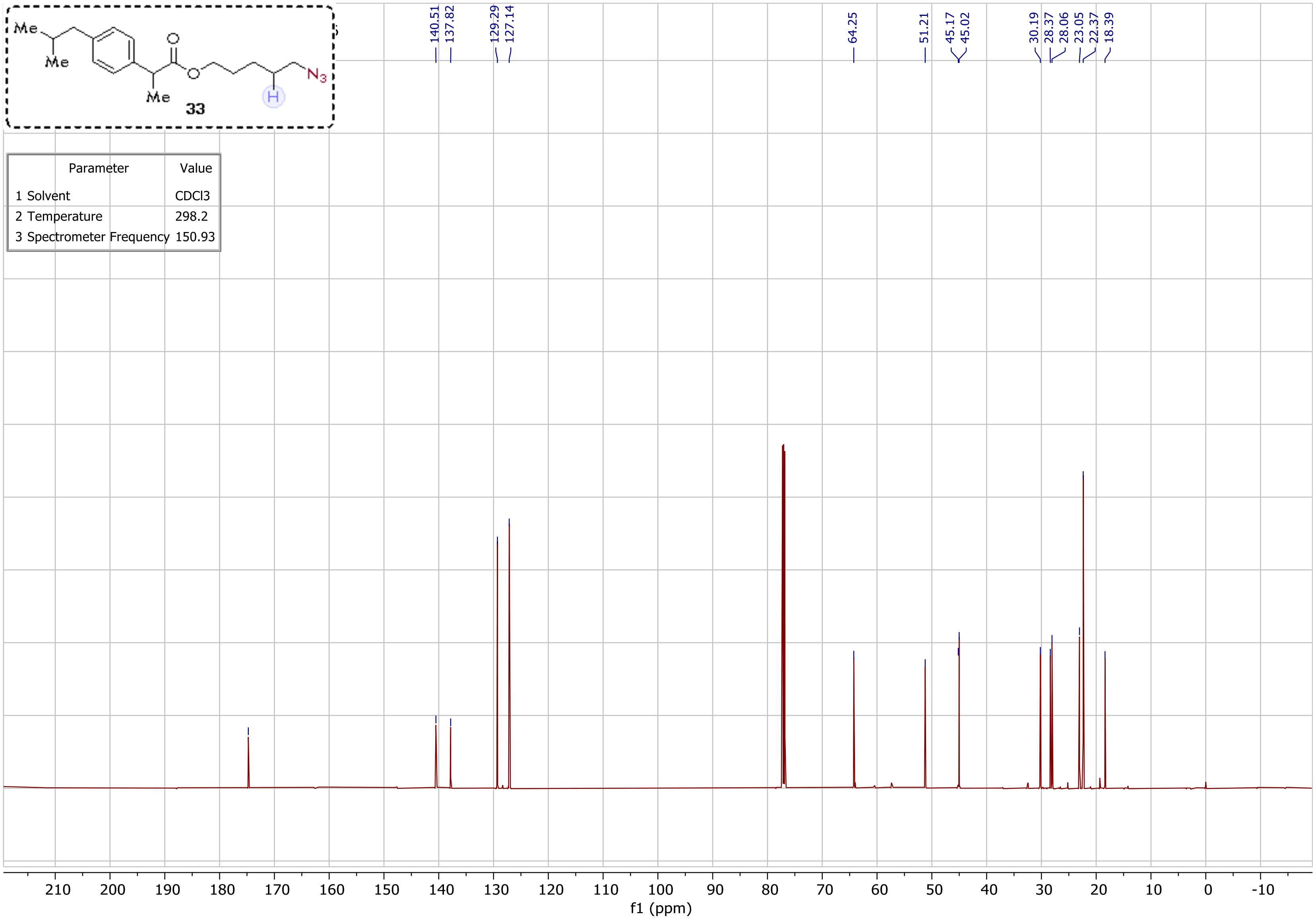

Parameter	Value
1 Solvent	CDCl3
2 Temperature	298.2
3 Spectrometer Frequency	150.93

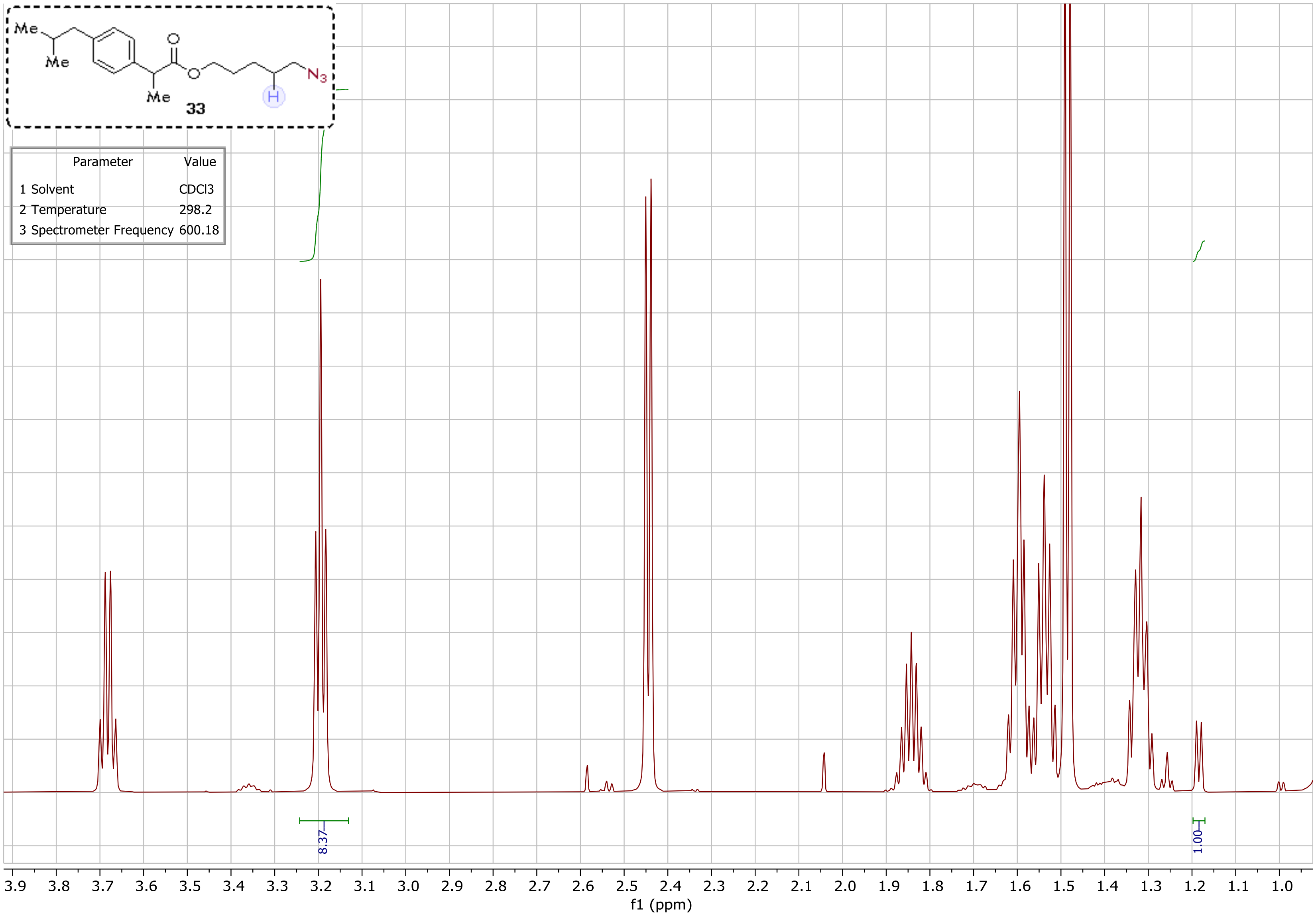
Me
Me
Me
N3
H
33
Parameter Value
1 Solvent CDCl3
2 Temperature 298.2
3 Spectrometer Frequency 600.18
8.37
1.00
3.9 3.8 3.7 3.6 3.5 3.4 3.3 3.2 3.1 3.0 2.9 2.8 2.7 2.6 2.5 2.4 2.3 2.2 2.1 2.0 1.9 1.8 1.7 1.6 1.5 1.4 1.3 1.2 1.1 1.0
f1 (ppm)

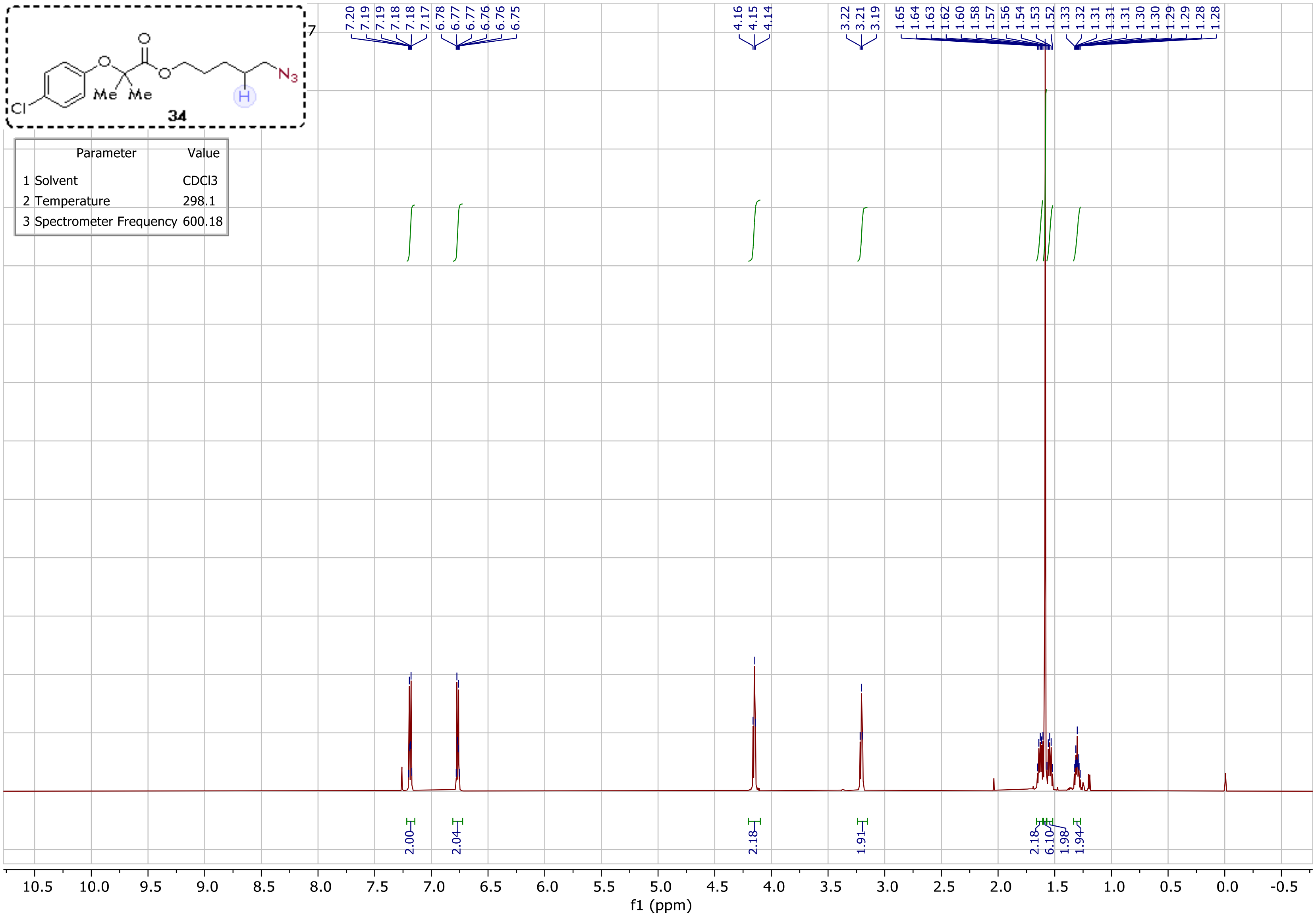

Parameter	Value
1 Solvent	CDCl3
2 Temperature	298.1
3 Spectrometer Frequency	600.18

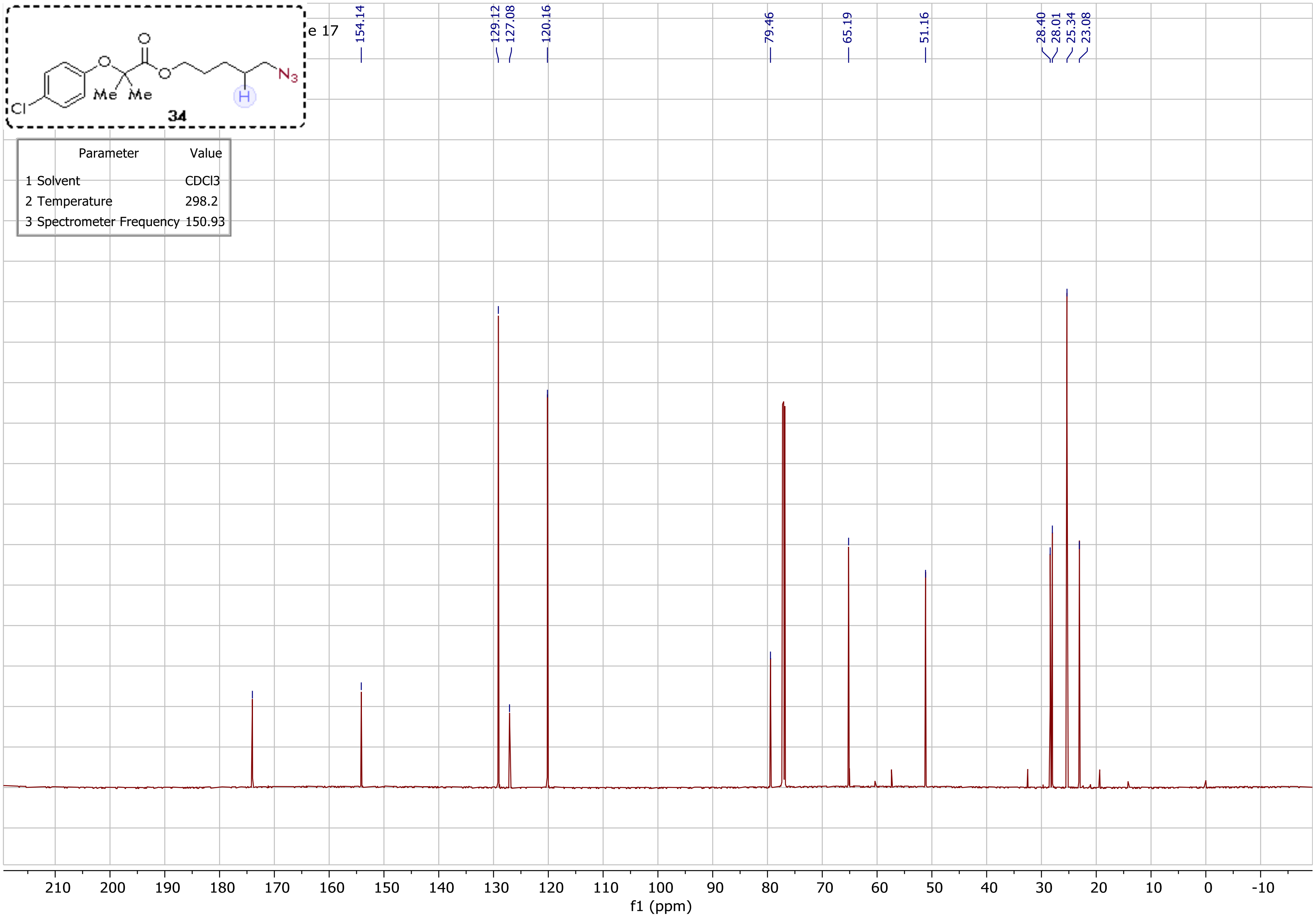

Parameter	Value
1 Solvent	CDCl3
2 Temperature	298.2
3 Spectrometer Frequency	150.93

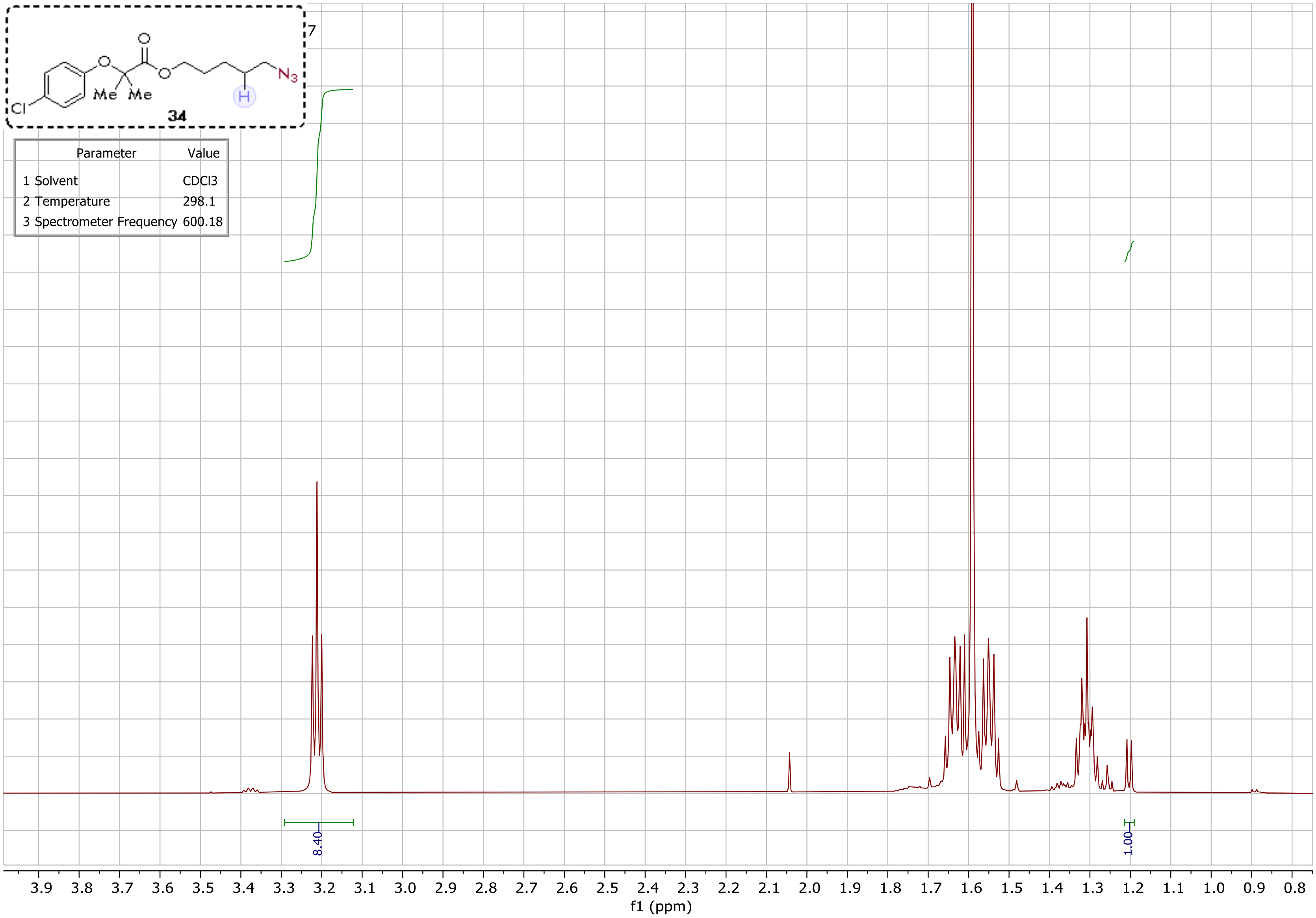

Parameter	Value
1 Solvent	CDCl3
2 Temperature	298.1
3 Spectrometer Frequency	600.18

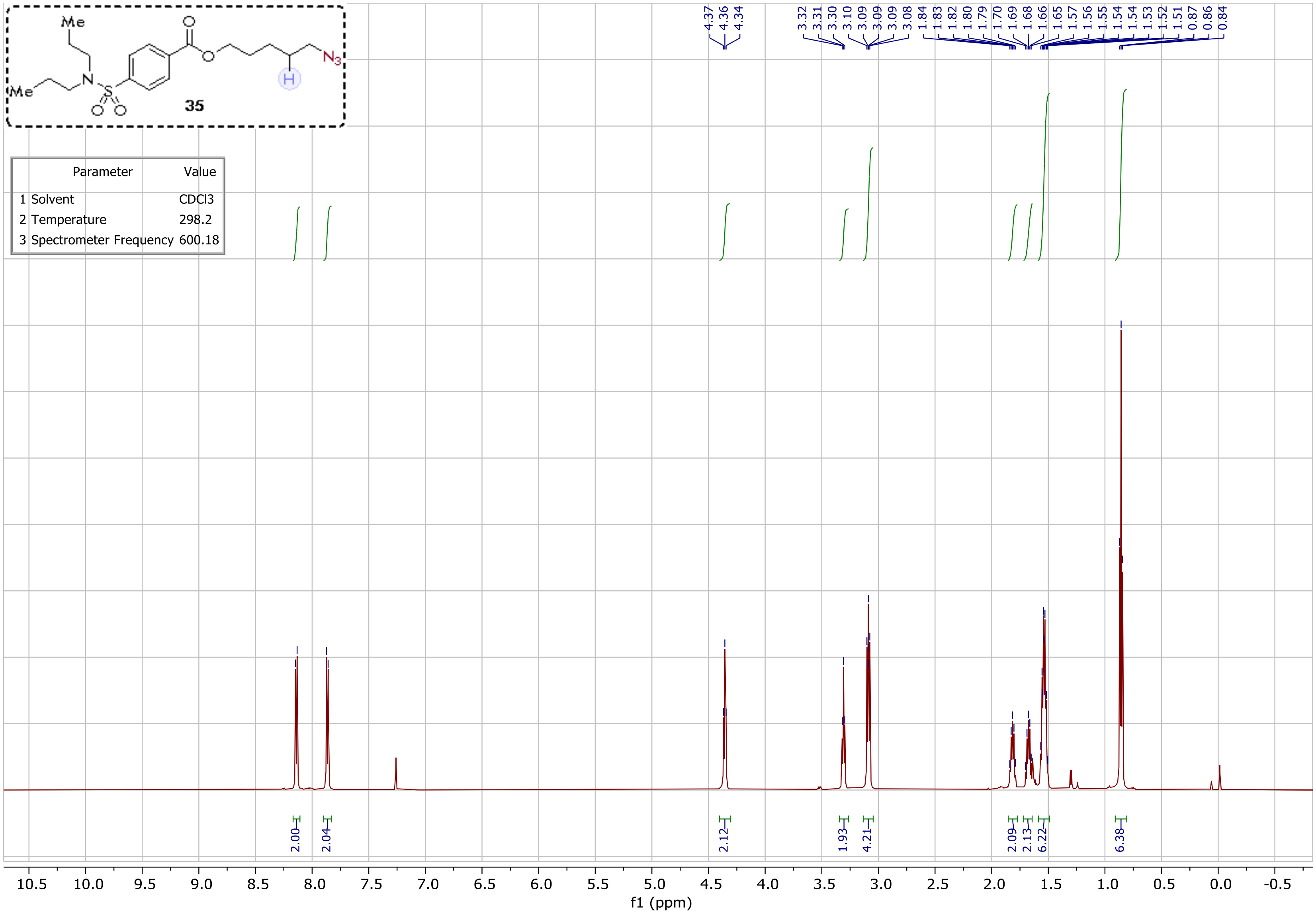
35
Parameter Value
1 Solvent CDCl3
2 Temperature 298.2
3 Spectrometer Frequency 600.18
4.37 4.36 4.34 3.32 3.31 3.30 3.10 3.09 3.09 3.09 3.08 1.84 1.83 1.82 1.80 1.79 1.70 1.69 1.68 1.66 1.65 1.57 1.56 1.55 1.54 1.54 1.53 1.52 1.51 0.87 0.86 0.84
2.00 2.04 2.12 1.93 4.21 2.09 2.13 6.22 6.38
f1 (ppm)

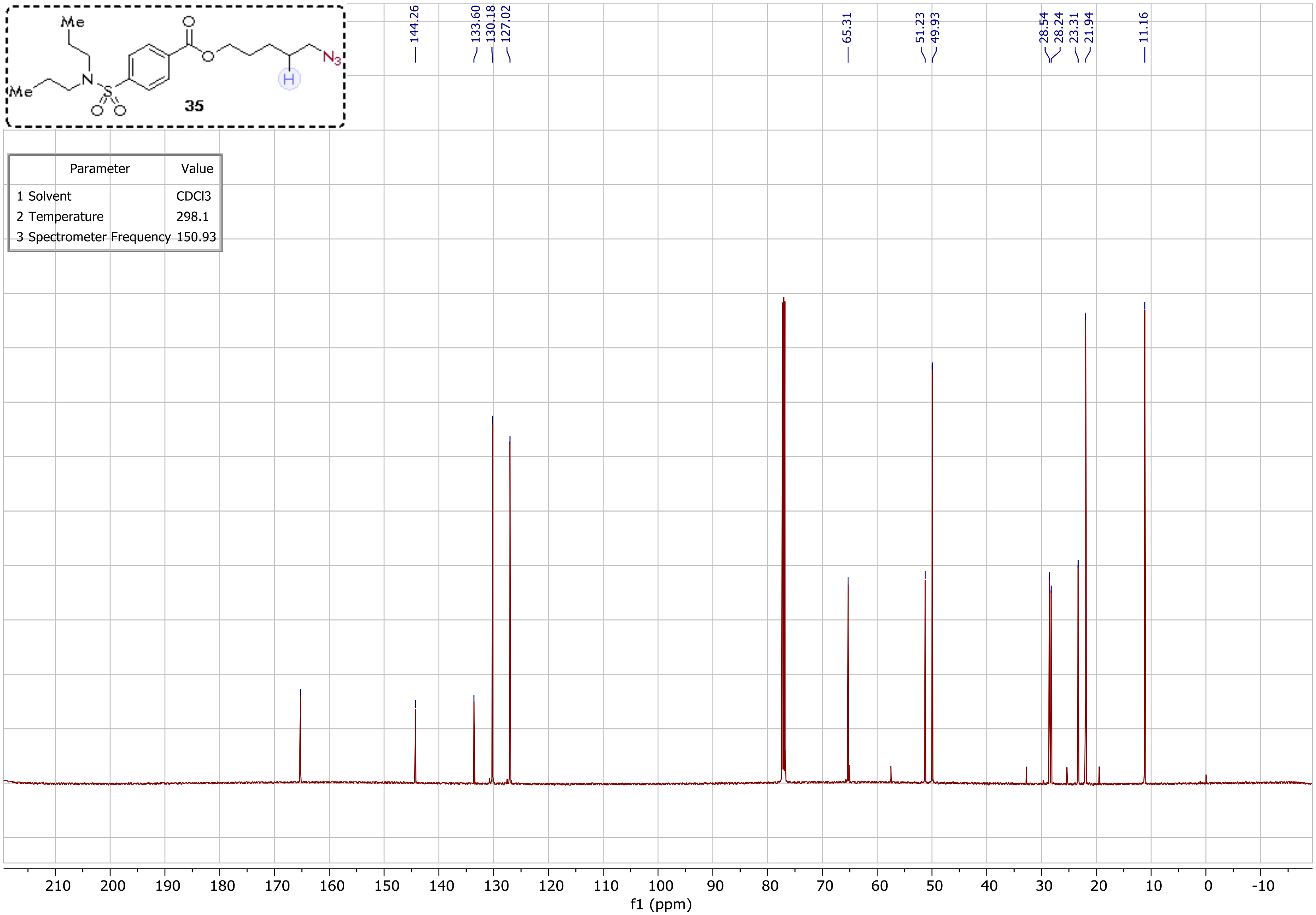
Me
Me
N
S
O
O
N3
H
35
144.26
133.60
130.18
127.02
65.31
51.23
49.93
28.54
28.24
23.31
21.94
11.16
Parameter Value
1 Solvent CDCl3
2 Temperature 298.1
3 Spectrometer Frequency 150.93
210 200 190 180 170 160 150 140 130 120 110 100 90 80 70 60 50 40 30 20 10 0 -10
f1 (ppm)

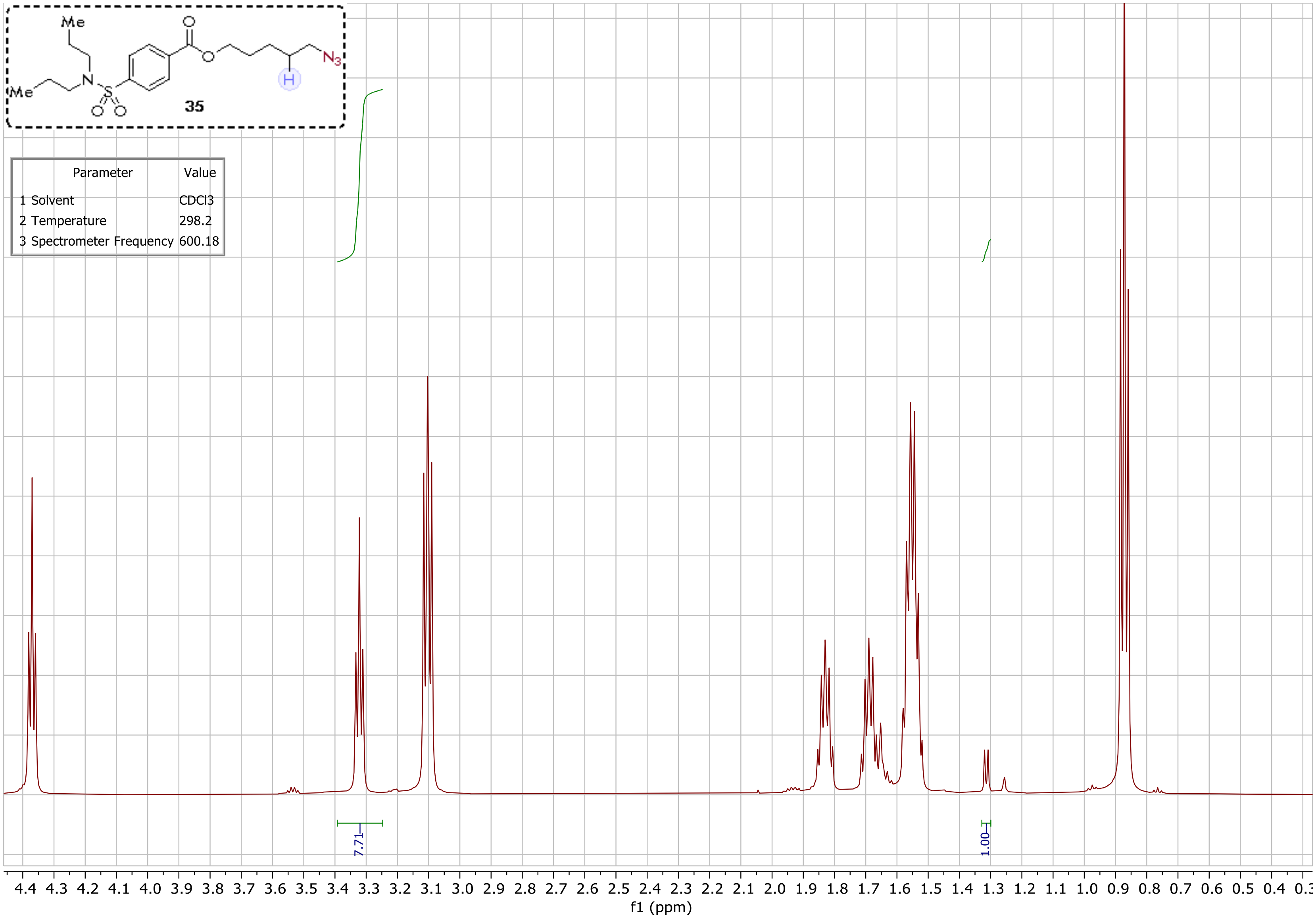
Me
Me
N
S
O
N3
H
35
Parameter | Value
1 Solvent | CDCl3
2 Temperature | 298.2
3 Spectrometer Frequency | 600.18
7.71
1.00
4.4 4.3 4.2 4.1 4.0 3.9 3.8 3.7 3.6 3.5 3.4 3.3 3.2 3.1 3.0 2.9 2.8 2.7 2.6 2.5 2.4 2.3 2.2 2.1 2.0 1.9 1.8 1.7 1.6 1.5 1.4 1.3 1.2 1.1 1.0 0.9 0.8 0.7 0.6 0.5 0.4
f1 (ppm)

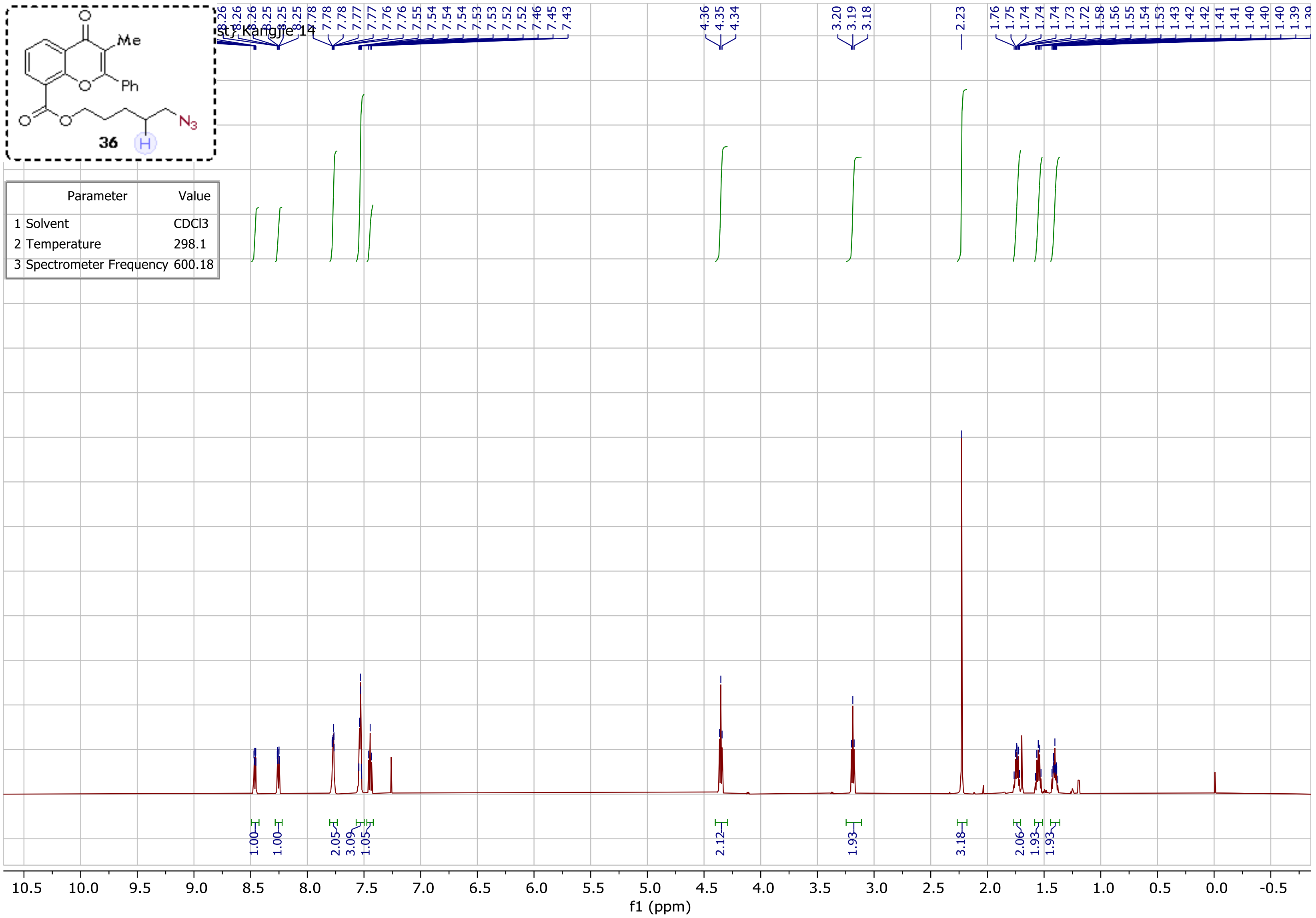

	Parameter	Value
1	Solvent	CDCl3
2	Temperature	298.1
3	Spectrometer Frequency	600.18

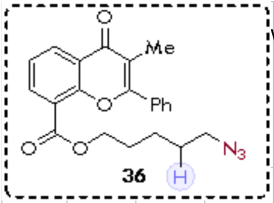

Parameter	Value
1 Solvent	CDCl3
2 Temperature	298.1
3 Spectrometer Frequency	150.93

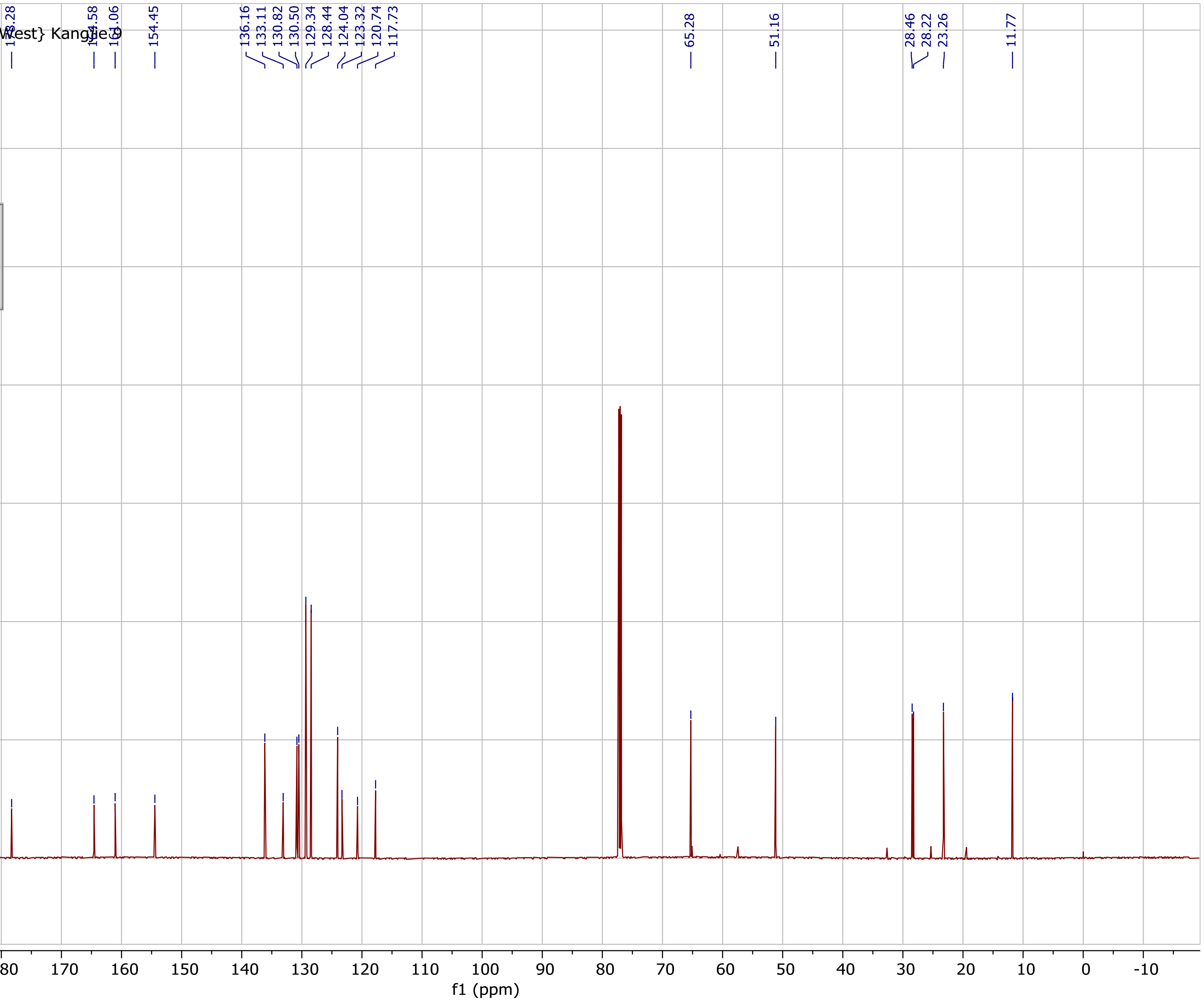

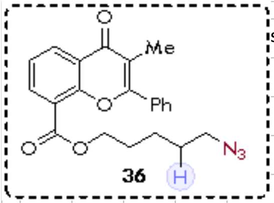

Parameter	Value
1 Solvent	CDCl3
2 Temperature	298.1
3 Spectrometer Frequency	600.18

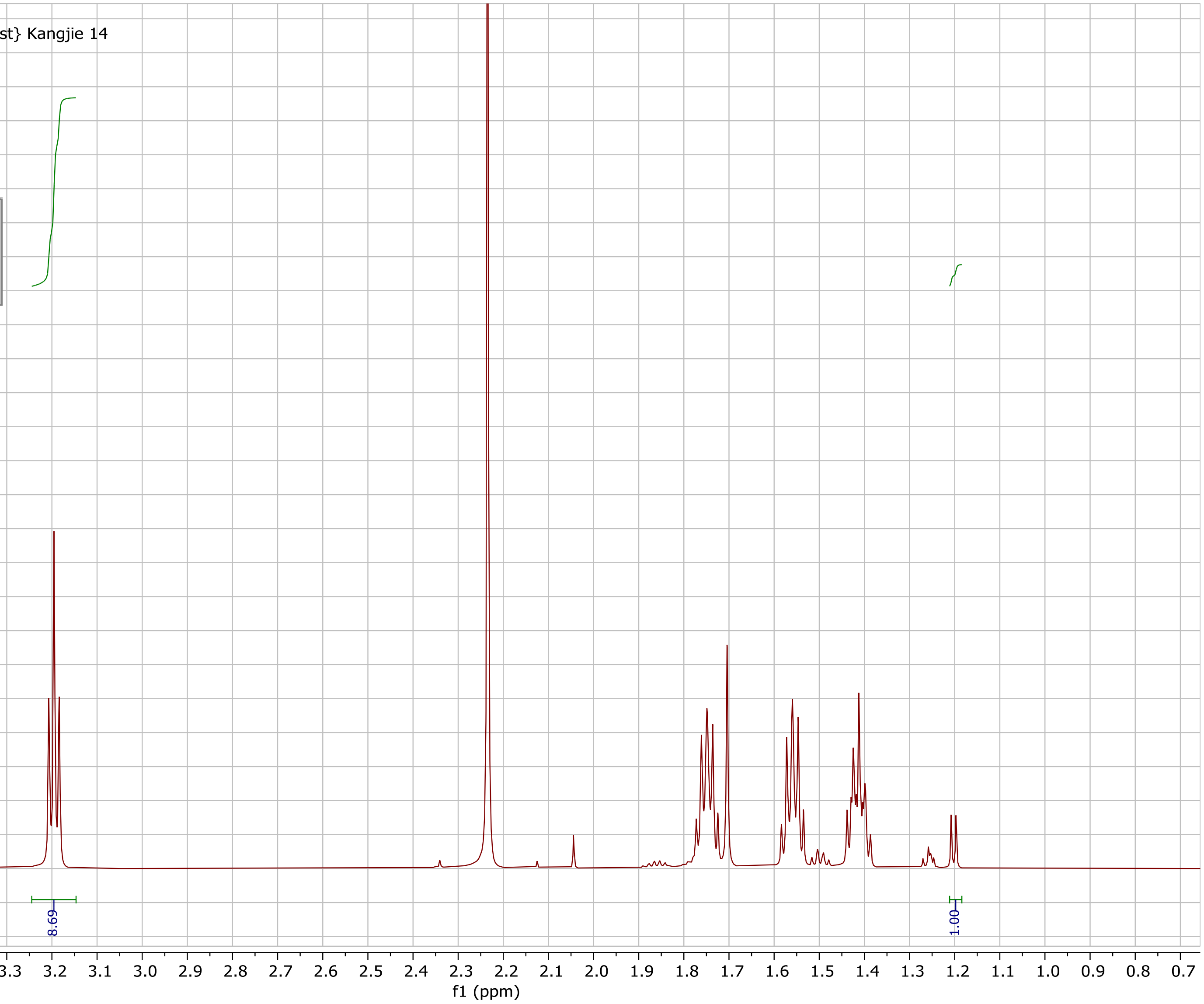

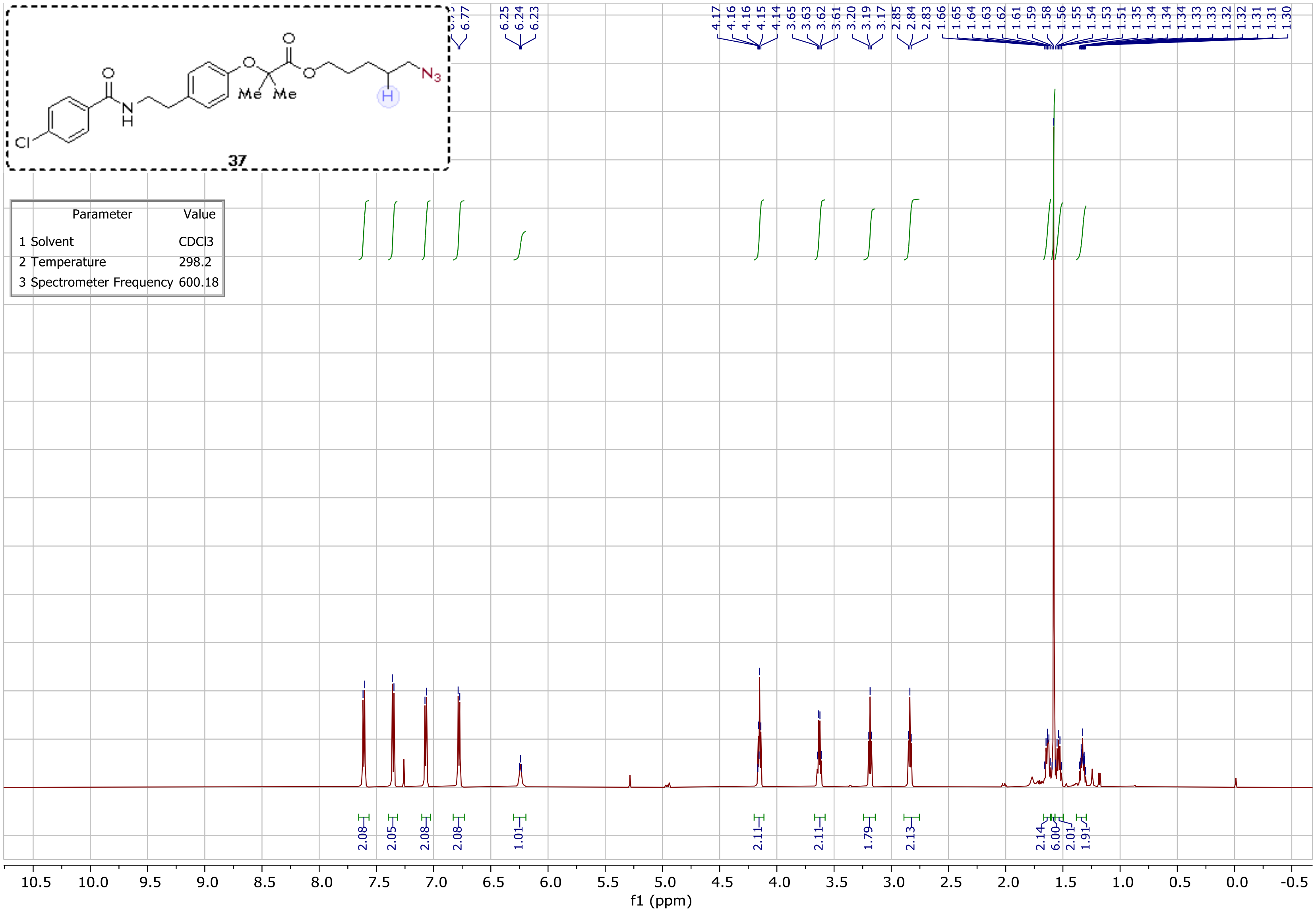

Parameter	Value
1 Solvent	CDCl3
2 Temperature	298.2
3 Spectrometer Frequency	600.18

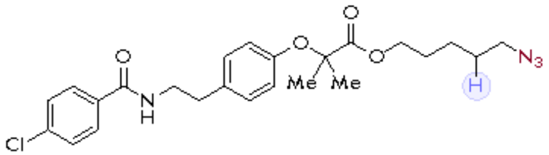

37

	Parameter	Value
1	Solvent	CDCl3
2	Temperature	298.2
3	Spectrometer Frequency	150.93

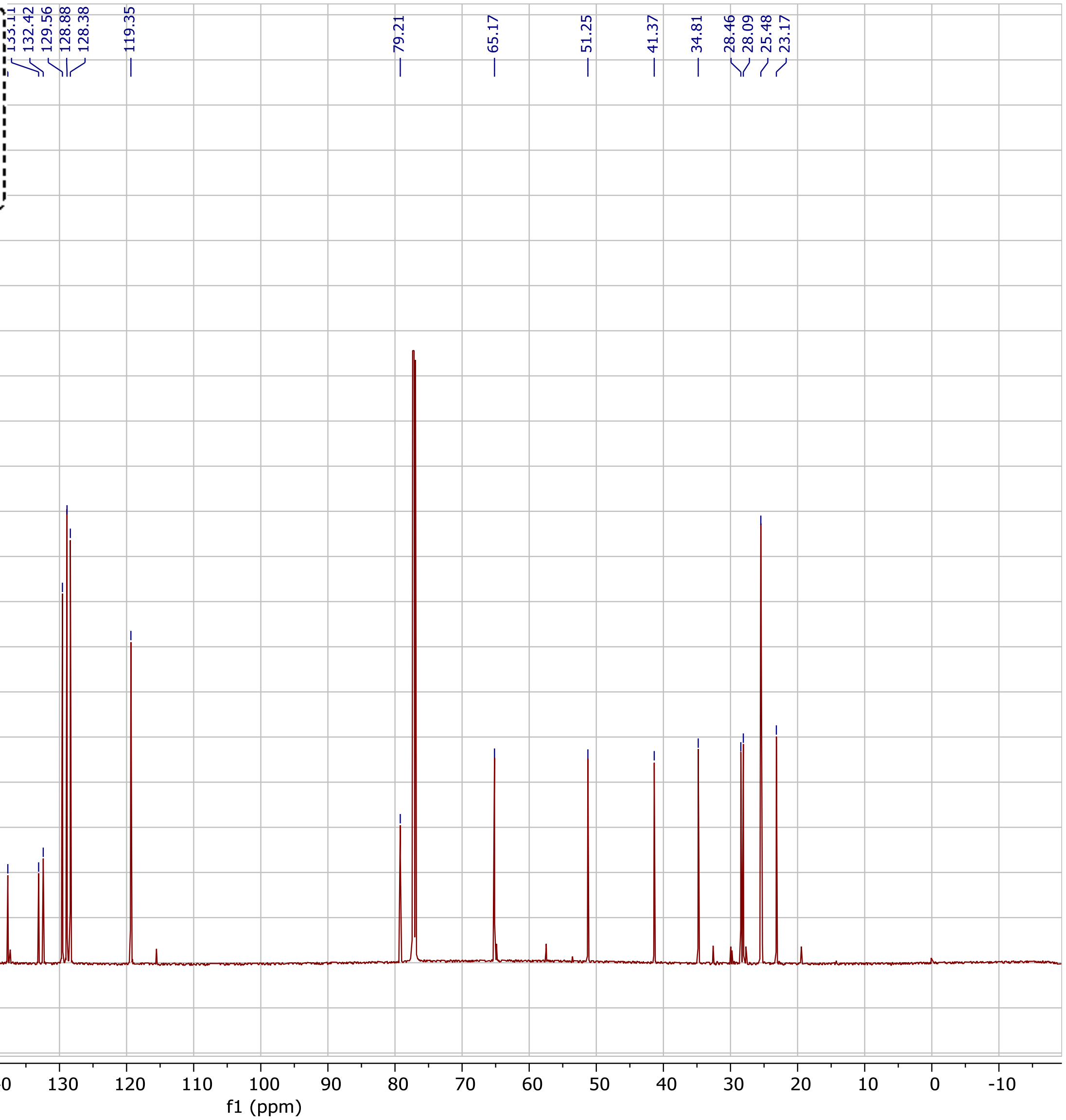

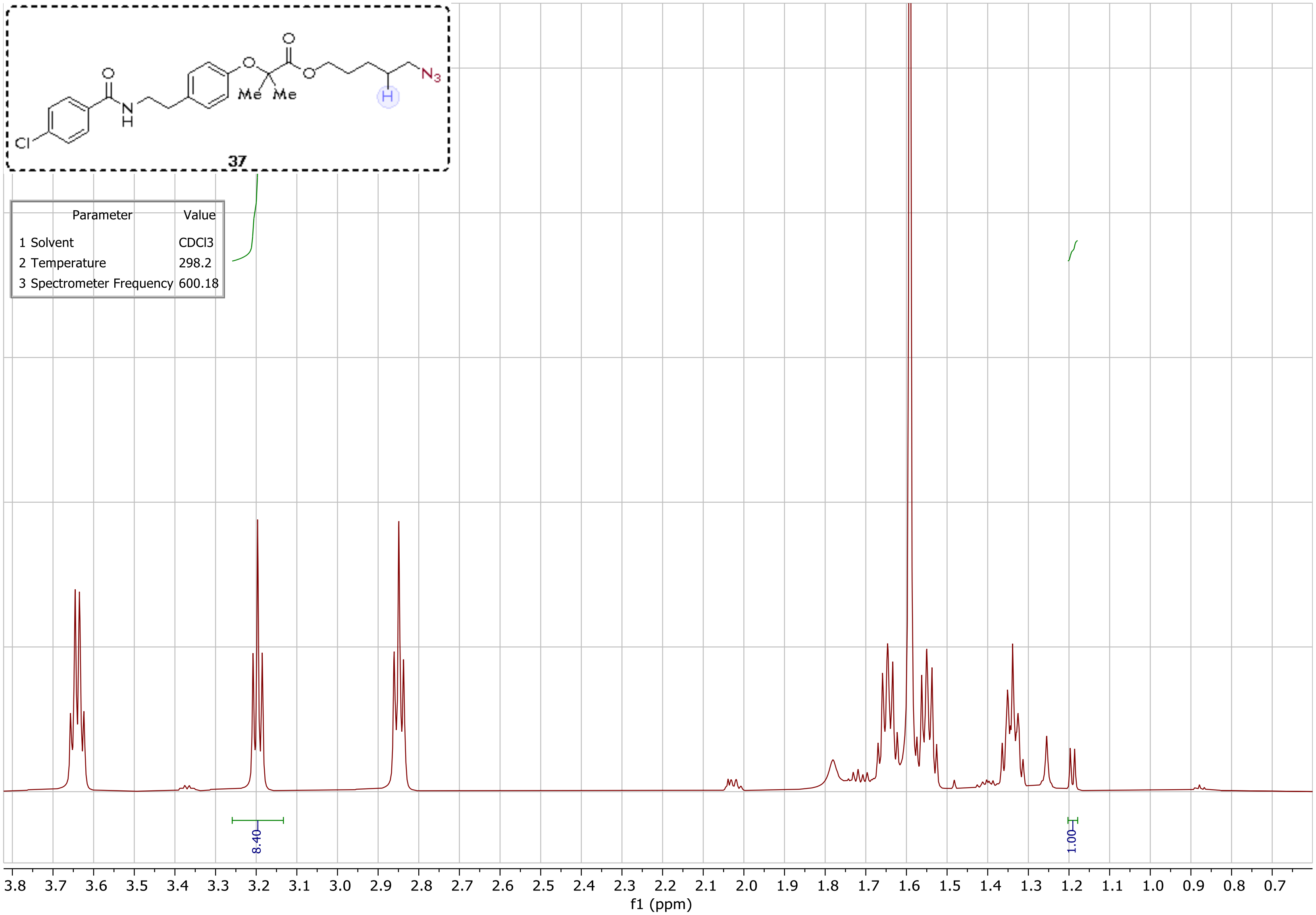
Cl
O
N
H
O
O
O
Me Me
H
N3
37
Parameter Value
1 Solvent CDCl3
2 Temperature 298.2
3 Spectrometer Frequency 600.18
8.40
1.00
3.8 3.7 3.6 3.5 3.4 3.3 3.2 3.1 3.0 2.9 2.8 2.7 2.6 2.5 2.4 2.3 2.2 2.1 2.0 1.9 1.8 1.7 1.6 1.5 1.4 1.3 1.2 1.1 1.0 0.9 0.8 0.7
f1 (ppm)

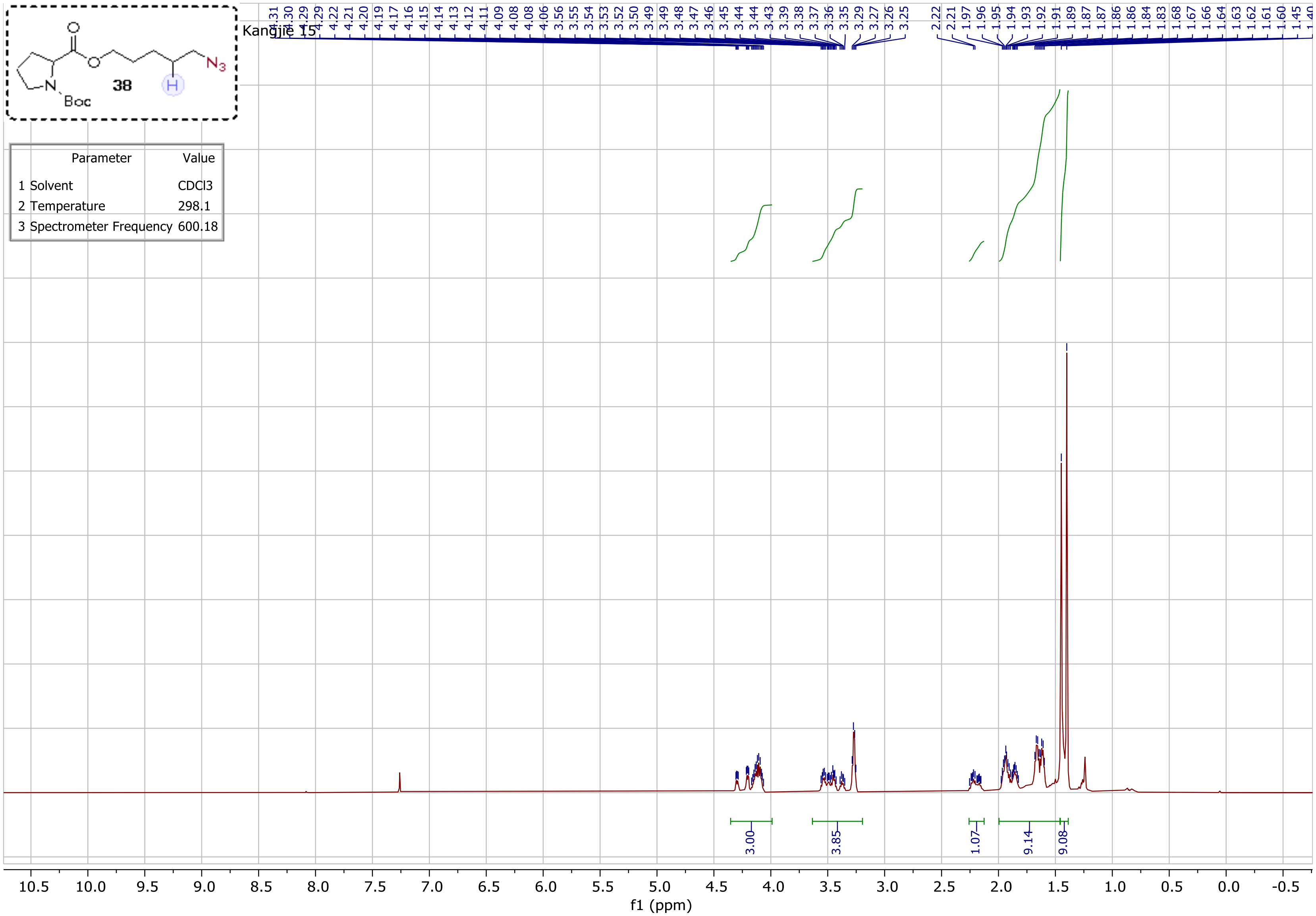
38
Boc
N3
Kangjie 15
Parameter Value
1 Solvent CDCl3
2 Temperature 298.1
3 Spectrometer Frequency 600.18
4.31 4.30 4.29 4.29 4.22 4.21 4.20 4.19 4.17 4.16 4.15 4.14 4.13 4.12 4.11 4.09 4.08 4.08 4.06 3.56 3.55 3.54 3.53 3.52 3.50 3.49 3.49 3.48 3.47 3.46 3.45 3.44 3.44 3.43 3.39 3.38 3.37 3.36 3.35 3.29 3.27 3.26 3.25
2.22 2.21 1.97 1.96 1.95 1.94 1.93 1.92 1.91 1.89 1.87 1.87 1.86 1.84 1.83 1.68 1.67 1.66 1.64 1.63 1.62 1.61 1.60 1.45 1.40
3.00
3.85
1.07
9.14
9.08
10.5 10.0 9.5 9.0 8.5 8.0 7.5 7.0 6.5 6.0 5.5 5.0 4.5 4.0 3.5 3.0 2.5 2.0 1.5 1.0 0.5 0.0 -0.5
f1 (ppm)

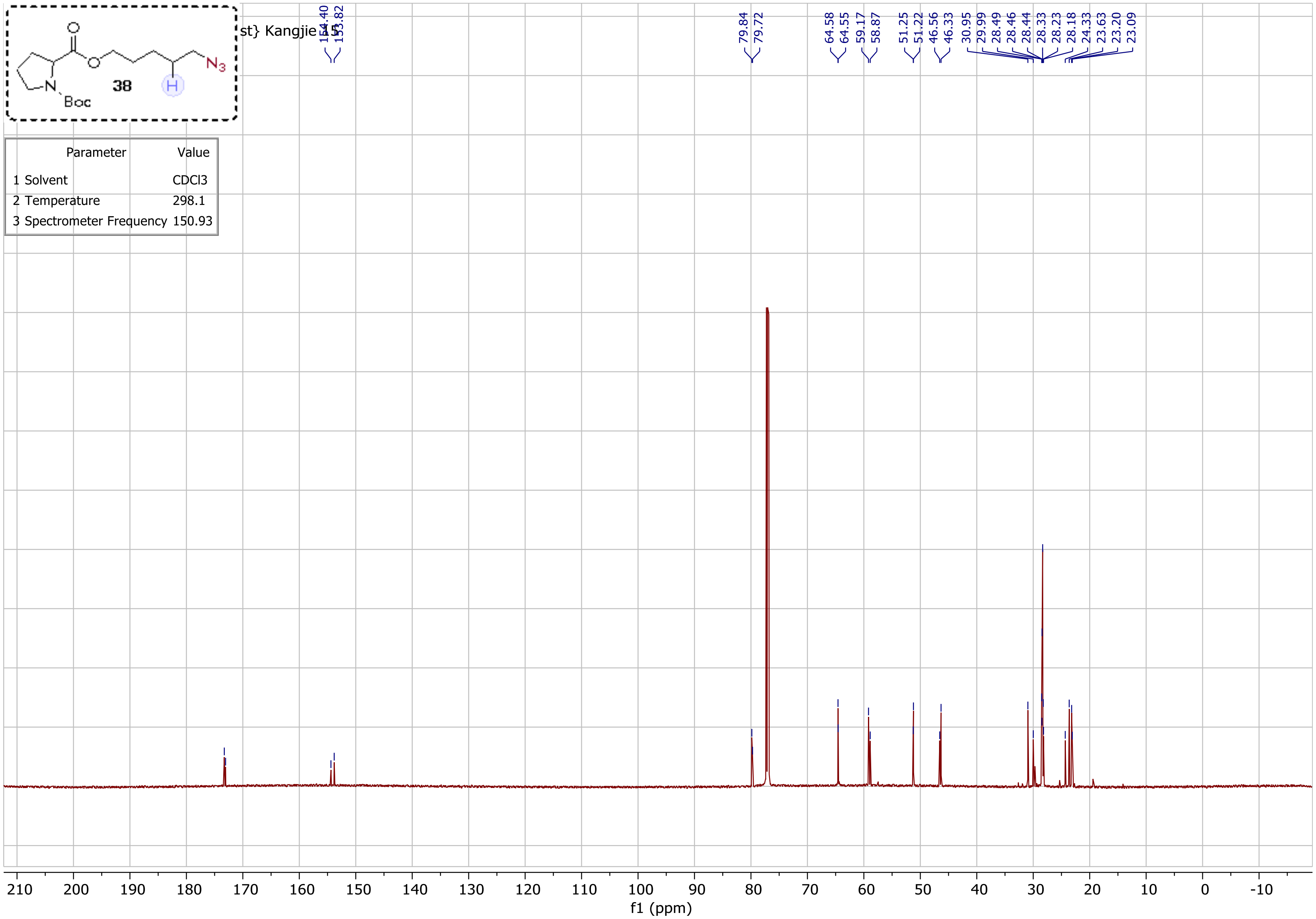

Parameter	Value
1 Solvent	CDCl3
2 Temperature	298.1
3 Spectrometer Frequency	150.93

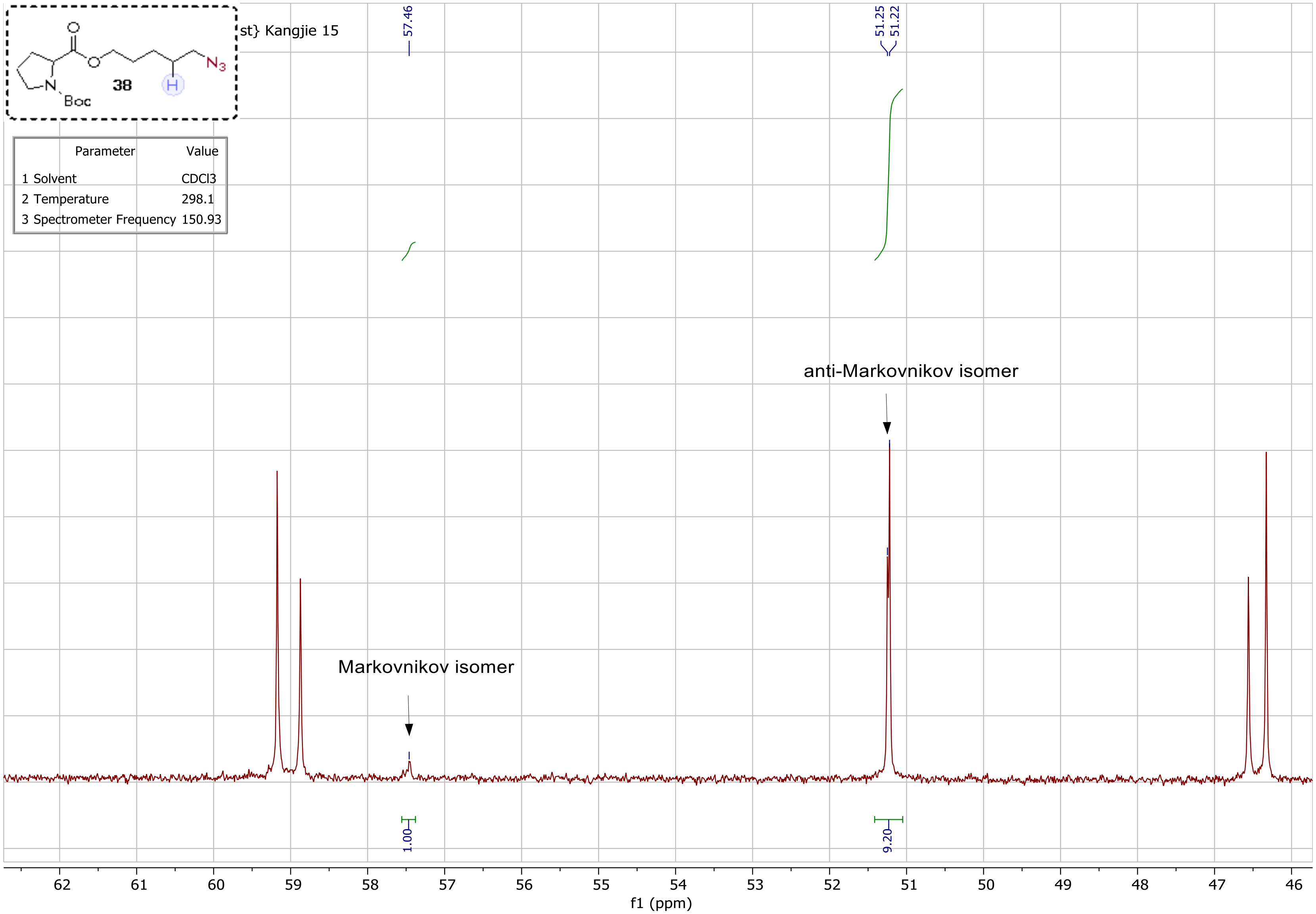

Parameter	Value
1 Solvent	CDCl3
2 Temperature	298.1
3 Spectrometer Frequency	150.93

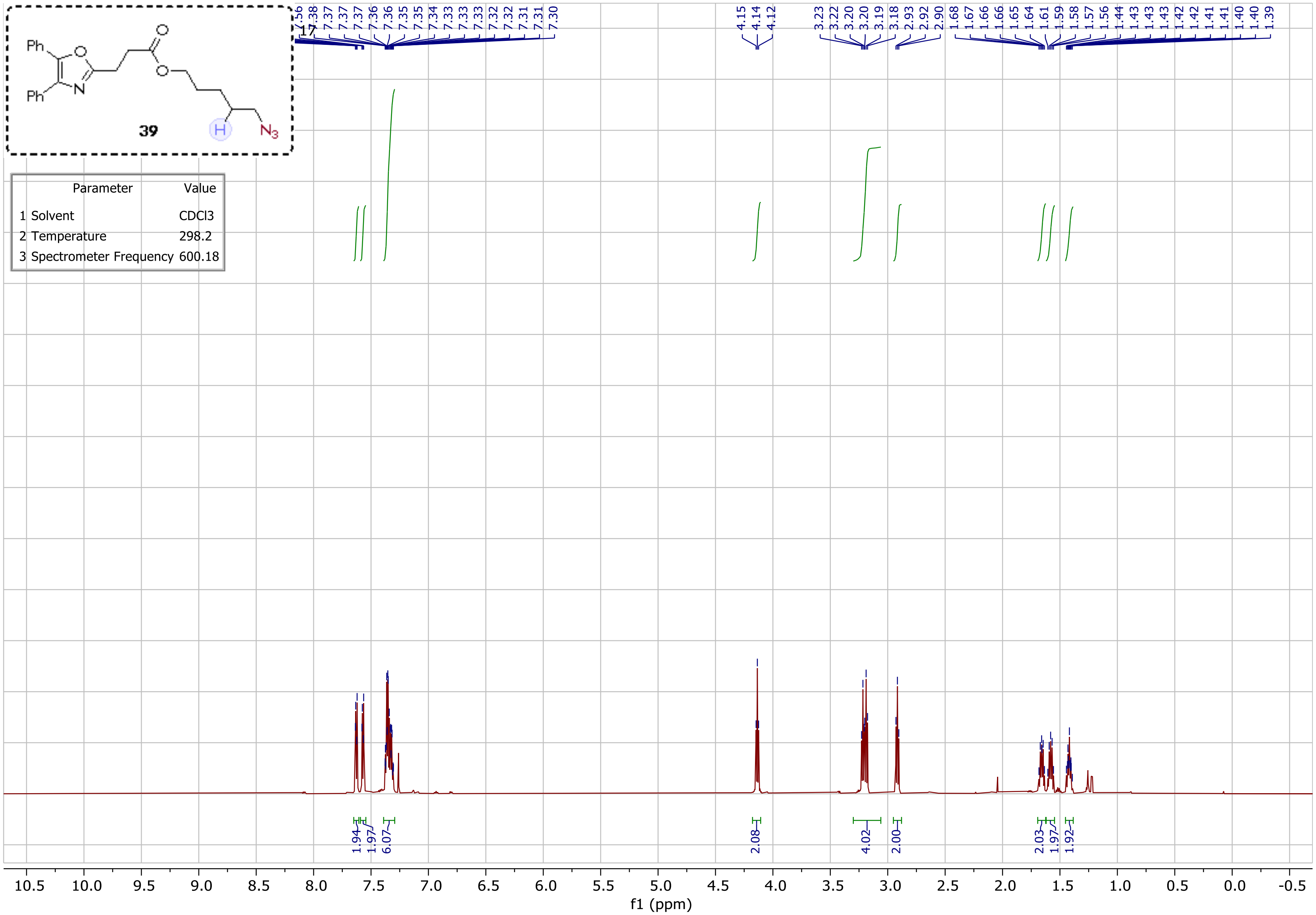
Ph
Ph
O
N
O
O
H
N3
39
Parameter Value
1 Solvent CDCl3
2 Temperature 298.2
3 Spectrometer Frequency 600.18
7.38 7.37 7.37 7.37 7.36 7.36 7.35 7.35 7.34 7.33 7.33 7.33 7.32 7.32 7.31 7.31 7.30
4.15 4.14 4.12
3.23 3.22 3.20 3.20 3.19 3.18 2.93 2.92 2.90
1.68 1.67 1.66 1.66 1.65 1.64 1.61 1.59 1.58 1.57 1.56 1.44 1.43 1.43 1.43 1.42 1.42 1.41 1.41 1.40 1.40 1.39
1.94 1.97 6.07 2.08 4.02 2.00 2.03 1.97 1.92
f1 (ppm)

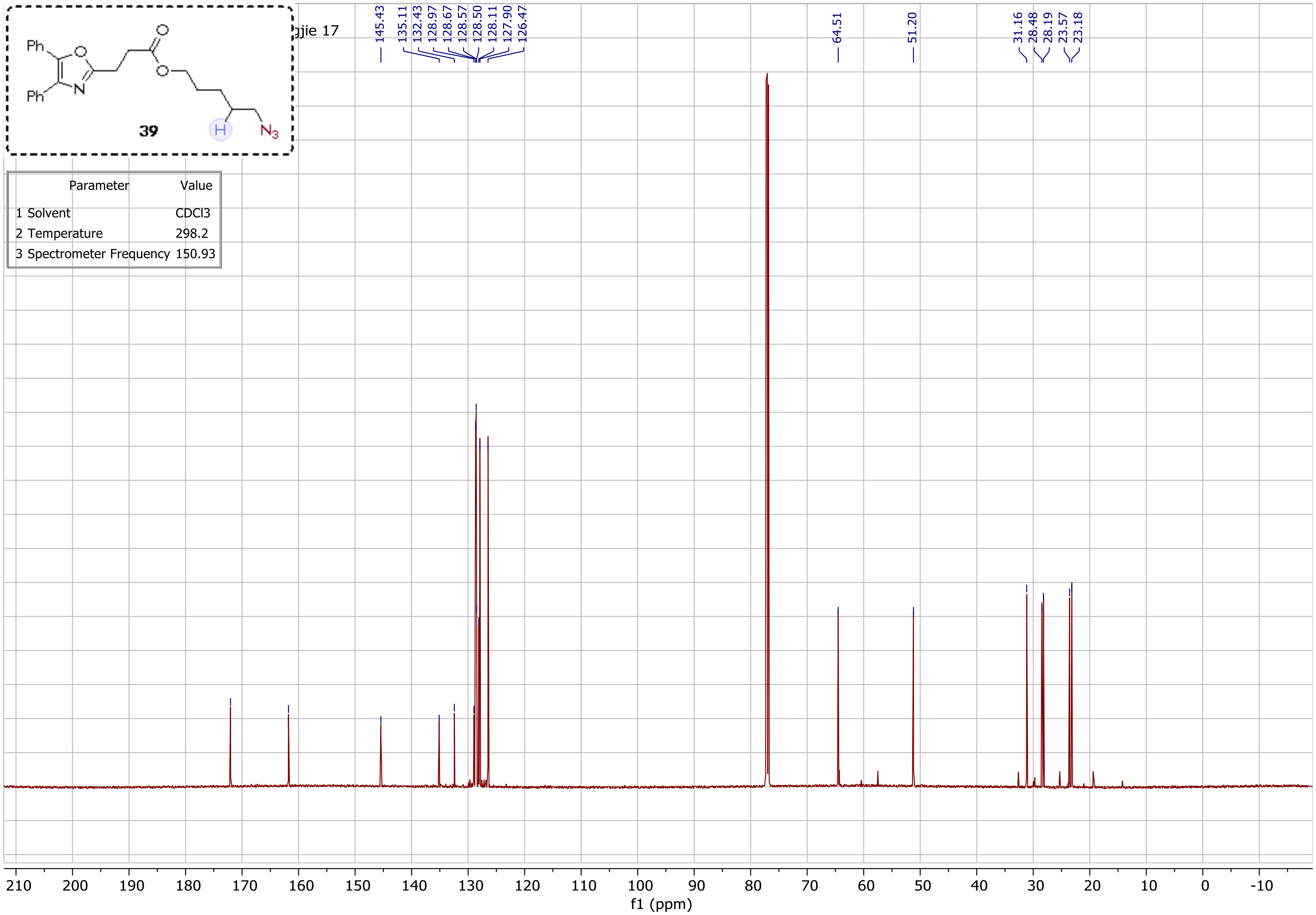

Parameter	Value
1 Solvent	CDCl3
2 Temperature	298.2
3 Spectrometer Frequency	150.93

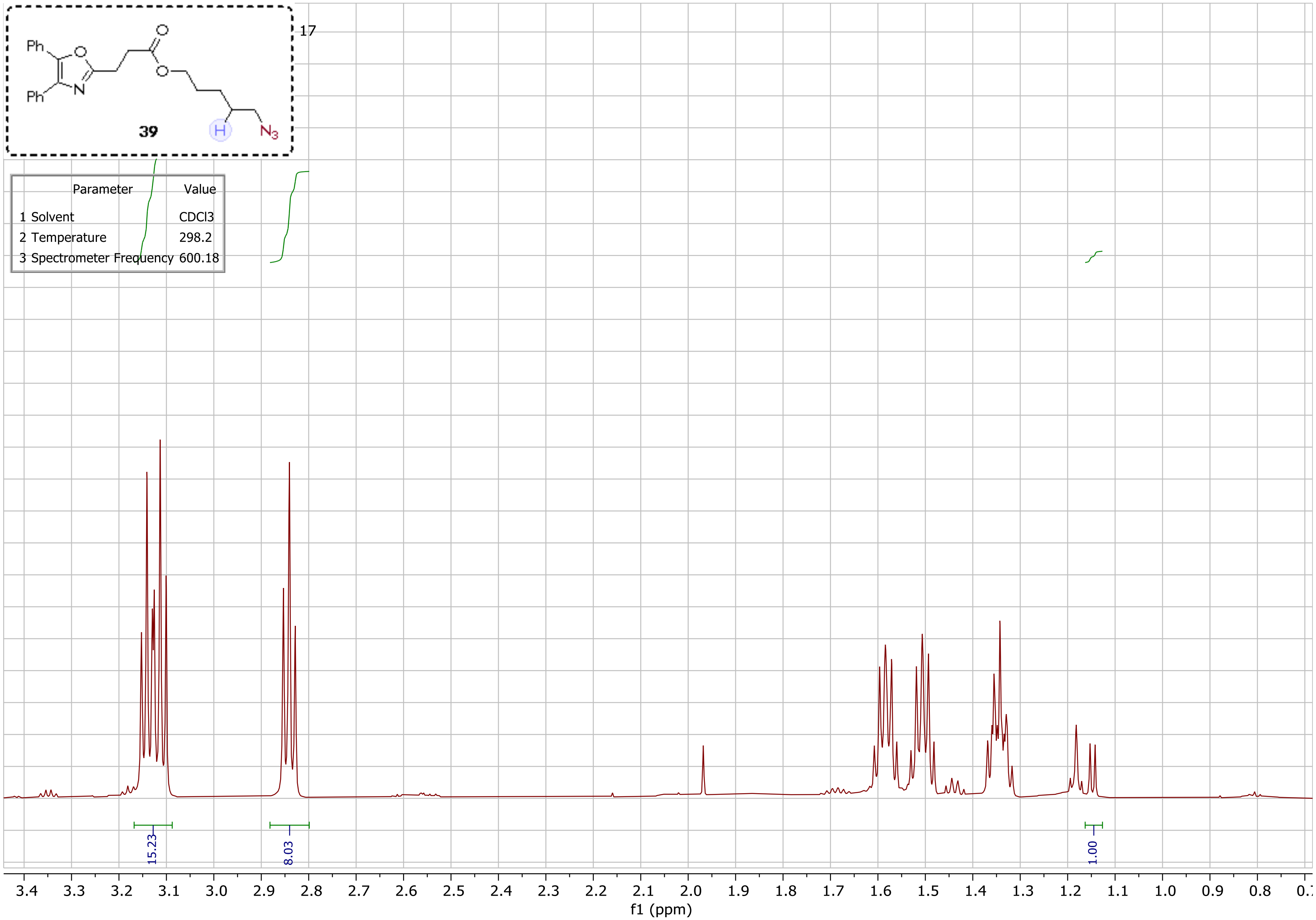
Ph
O
N
Ph
O
O
39
H
N3
17
Parameter Value
1 Solvent CDCl3
2 Temperature 298.2
3 Spectrometer Frequency 600.18
15.23
8.03
1.00
3.4 3.3 3.2 3.1 3.0 2.9 2.8 2.7 2.6 2.5 2.4 2.3 2.2 2.1 2.0 1.9 1.8 1.7 1.6 1.5 1.4 1.3 1.2 1.1 1.0 0.9 0.8
f1 (ppm)

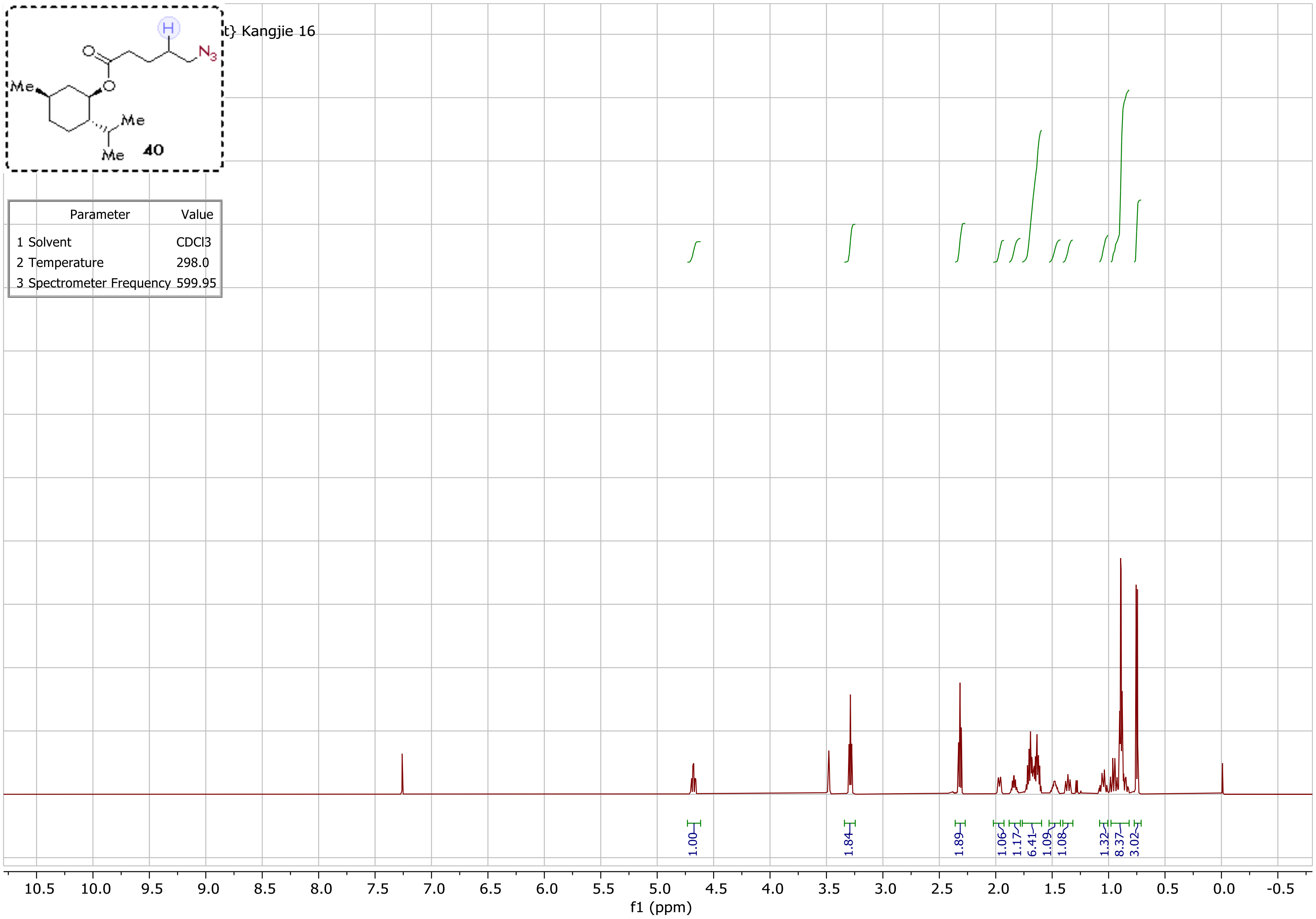

t} Kangjie 16
N3
Me
Me
Me
40
Parameter Value
1 Solvent CDCl3
2 Temperature 298.0
3 Spectrometer Frequency 599.95
1.00
1.84
1.89
1.06
1.17
6.41
1.09
1.08
1.32
8.37
3.02
10.5 10.0 9.5 9.0 8.5 8.0 7.5 7.0 6.5 6.0 5.5 5.0 4.5 4.0 3.5 3.0 2.5 2.0 1.5 1.0 0.5 0.0 -0.5
f1 (ppm)

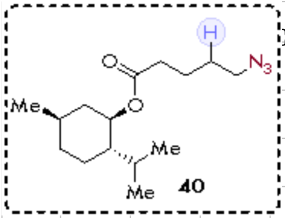

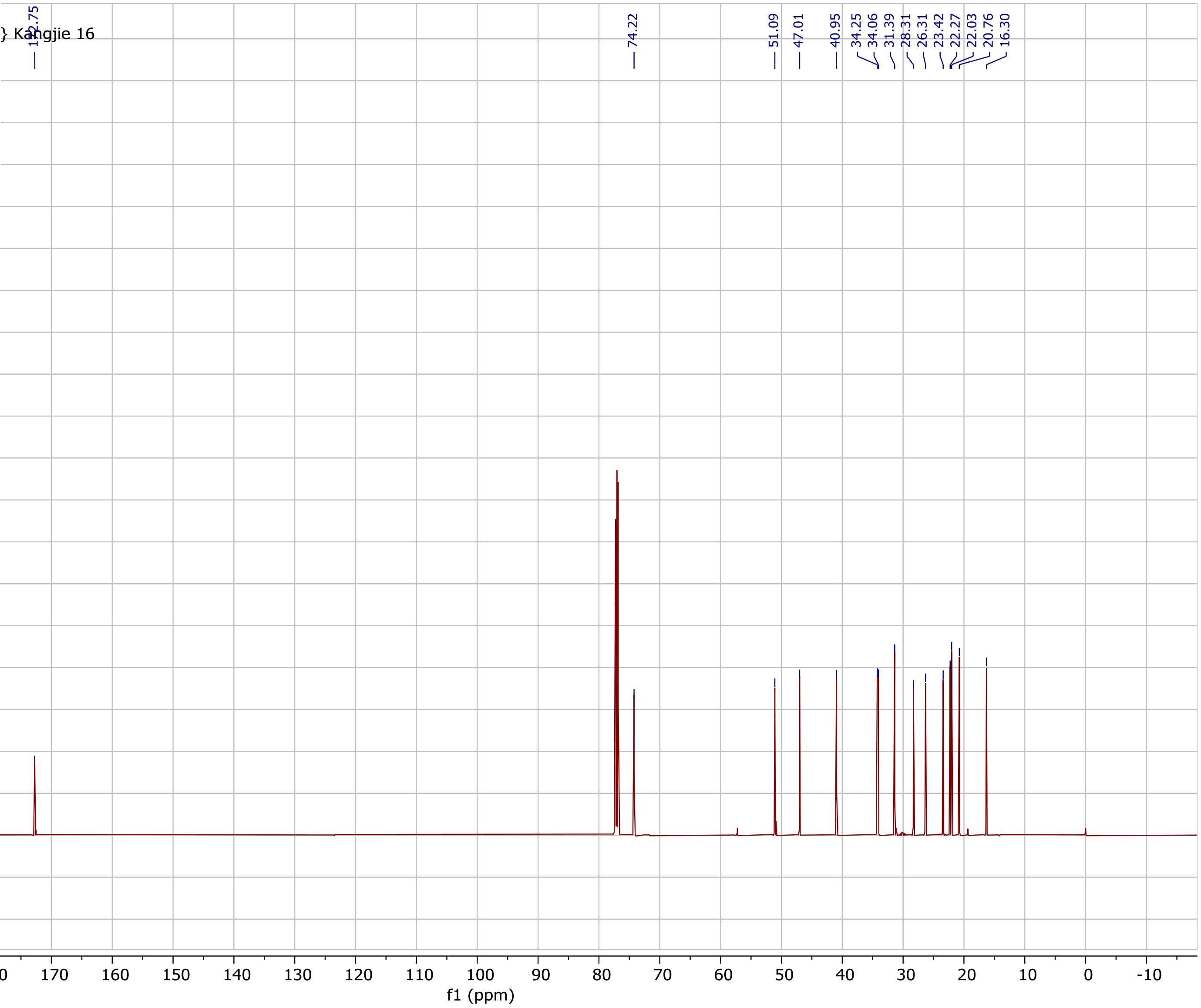

	Parameter	Value
1	Solvent	CDCl3
2	Temperature	298.0
3	Spectrometer Frequency	150.87

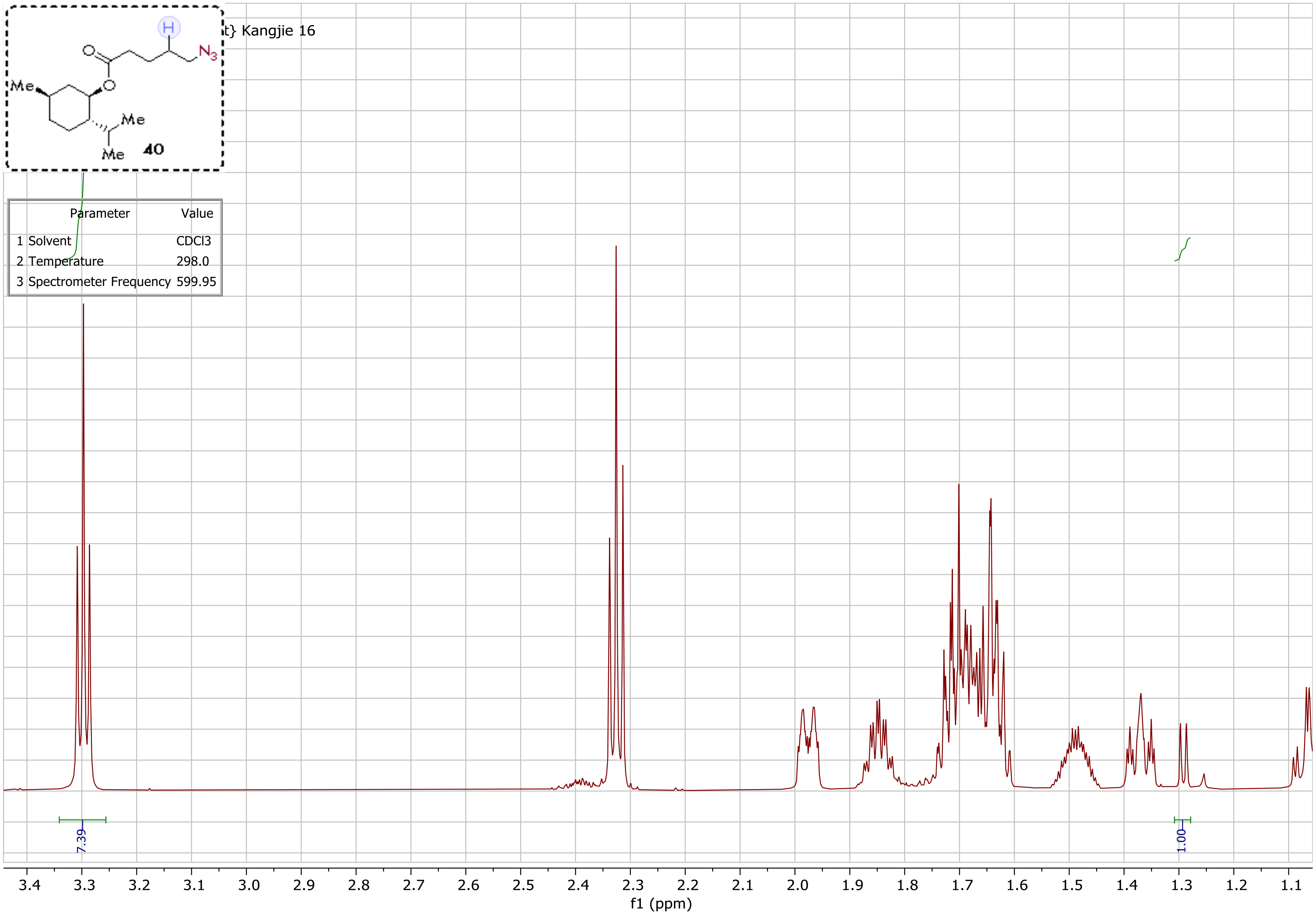

Me
Me
Me
N3
H
40
t} Kangjie 16
Parameter Value
1 Solvent CDCl3
2 Temperature 298.0
3 Spectrometer Frequency 599.95
7.39
1.00
3.4 3.3 3.2 3.1 3.0 2.9 2.8 2.7 2.6 2.5 2.4 2.3 2.2 2.1 2.0 1.9 1.8 1.7 1.6 1.5 1.4 1.3 1.2 1.1
f1 (ppm)

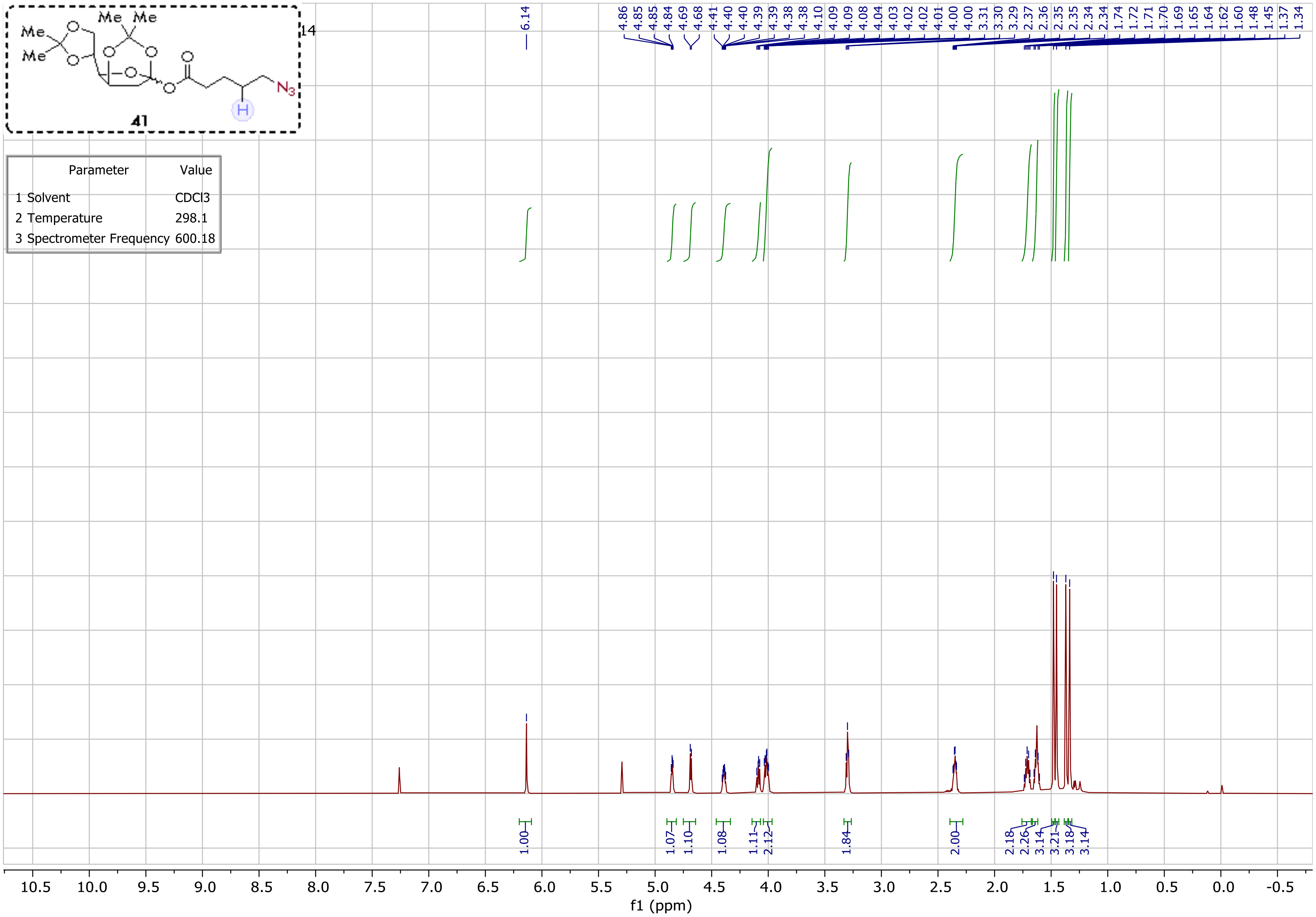

Parameter	Value
1 Solvent	CDCl3
2 Temperature	298.1
3 Spectrometer Frequency	600.18

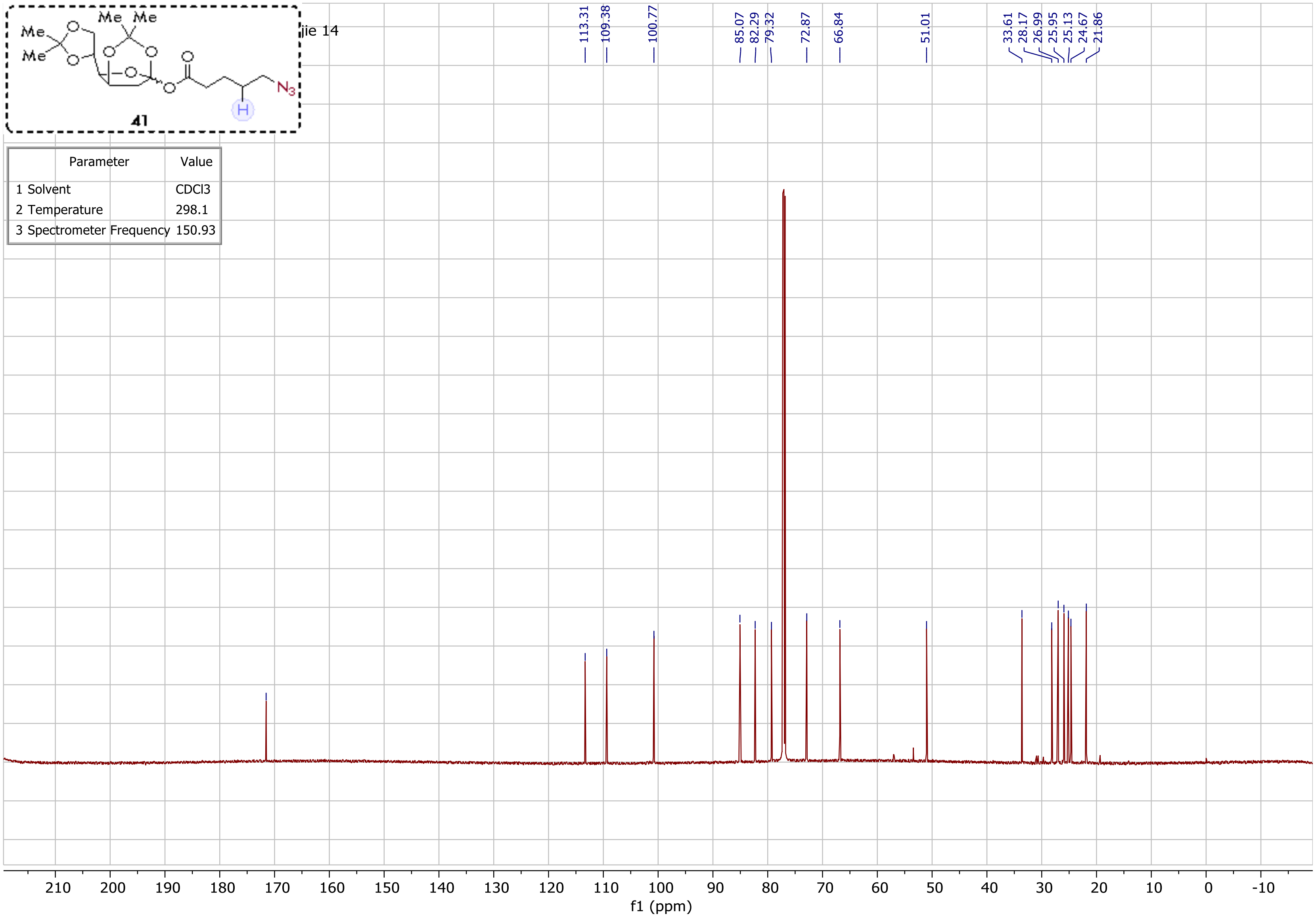

	Parameter	Value
1	Solvent	CDCl3
2	Temperature	298.1
3	Spectrometer Frequency	150.93

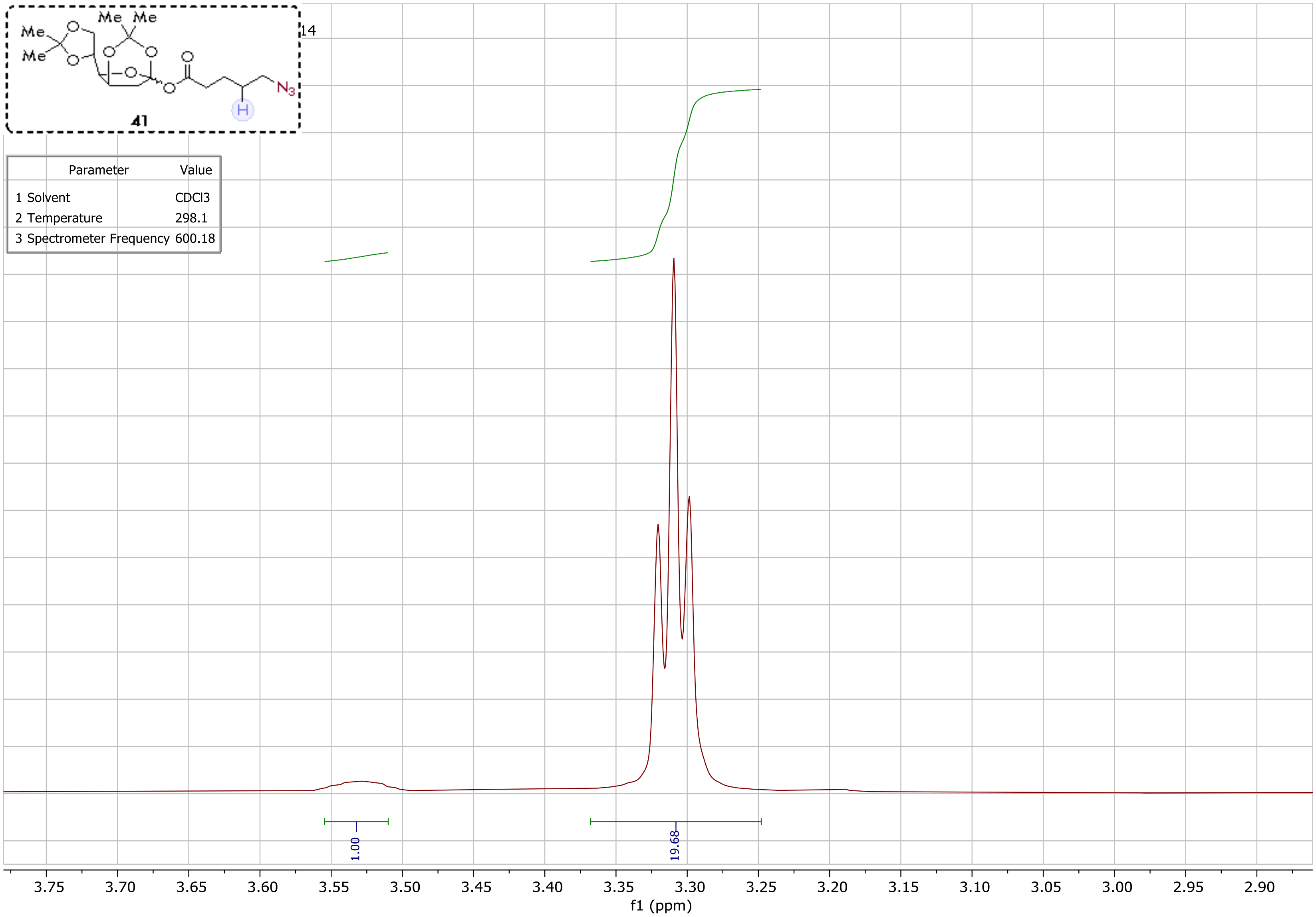

Parameter	Value
1 Solvent	CDCl3
2 Temperature	298.1
3 Spectrometer Frequency	600.18

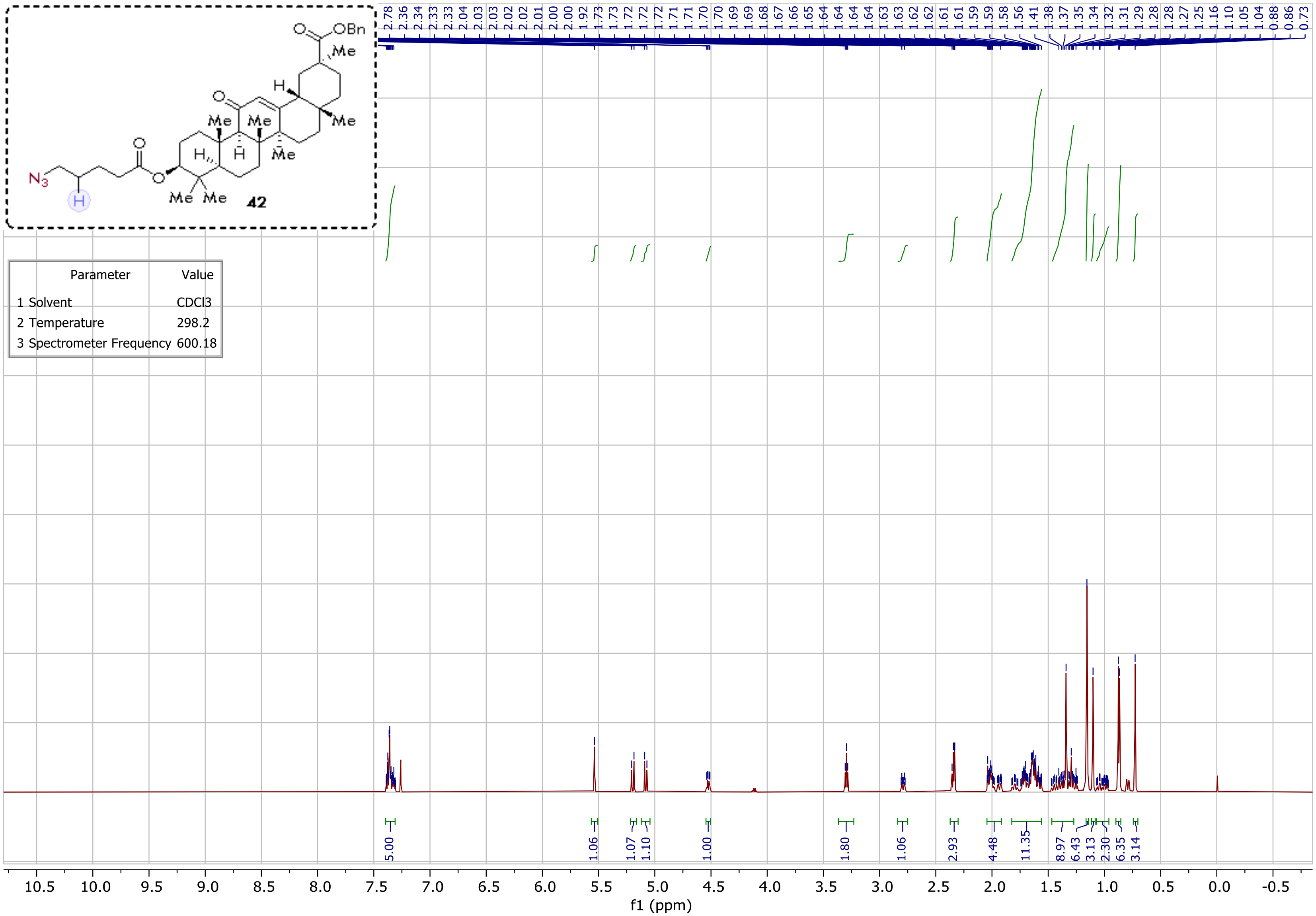

	Parameter	Value
1	Solvent	CDCl3
2	Temperature	298.2
3	Spectrometer Frequency	600.18

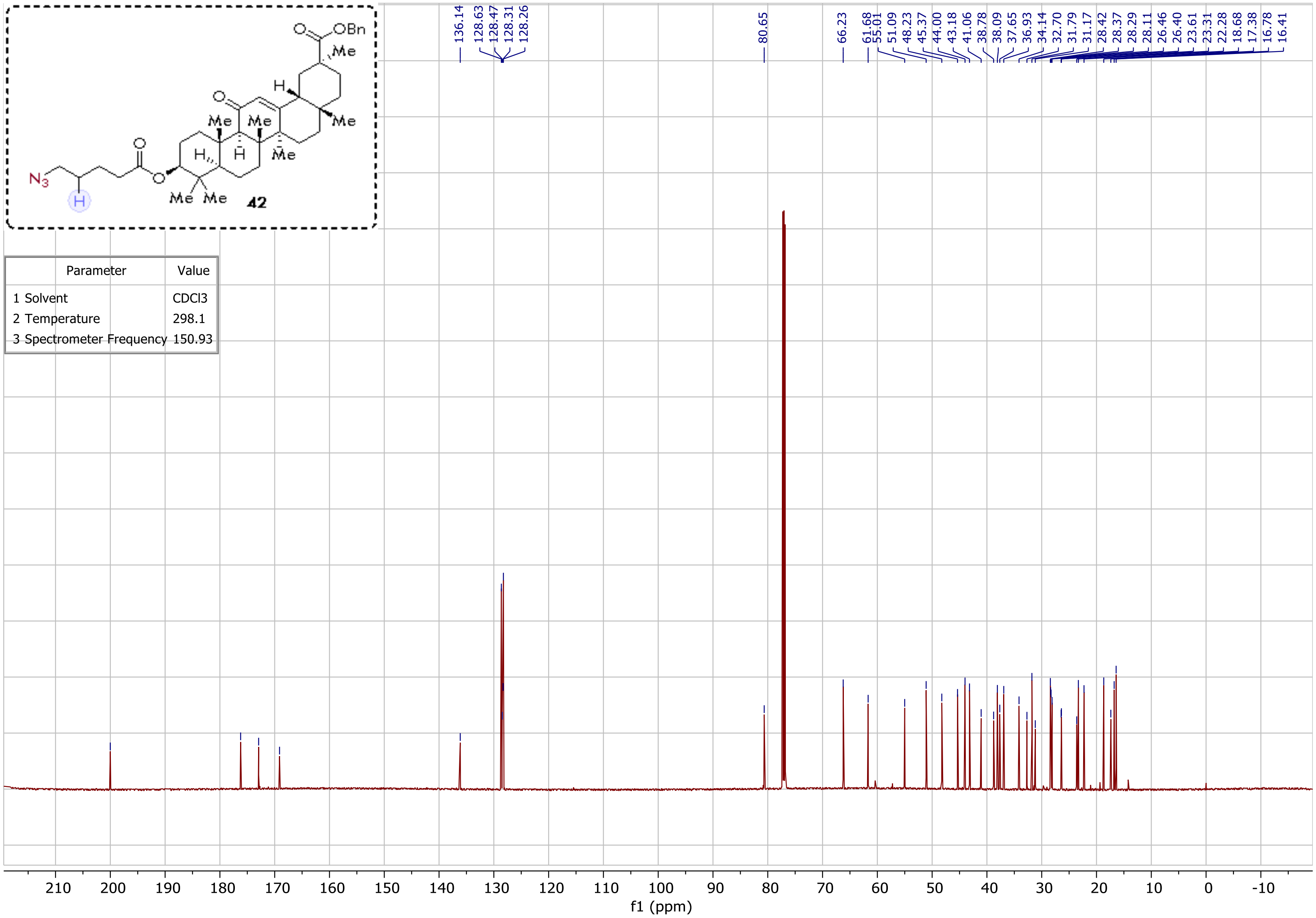

Parameter	Value
1 Solvent	CDCl3
2 Temperature	298.1
3 Spectrometer Frequency	150.93

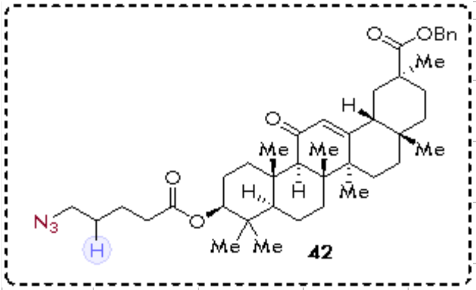

Parameter	Value
1 Solvent	CDCl3
2 Temperature	298.2
3 Spectrometer Frequency	600.18

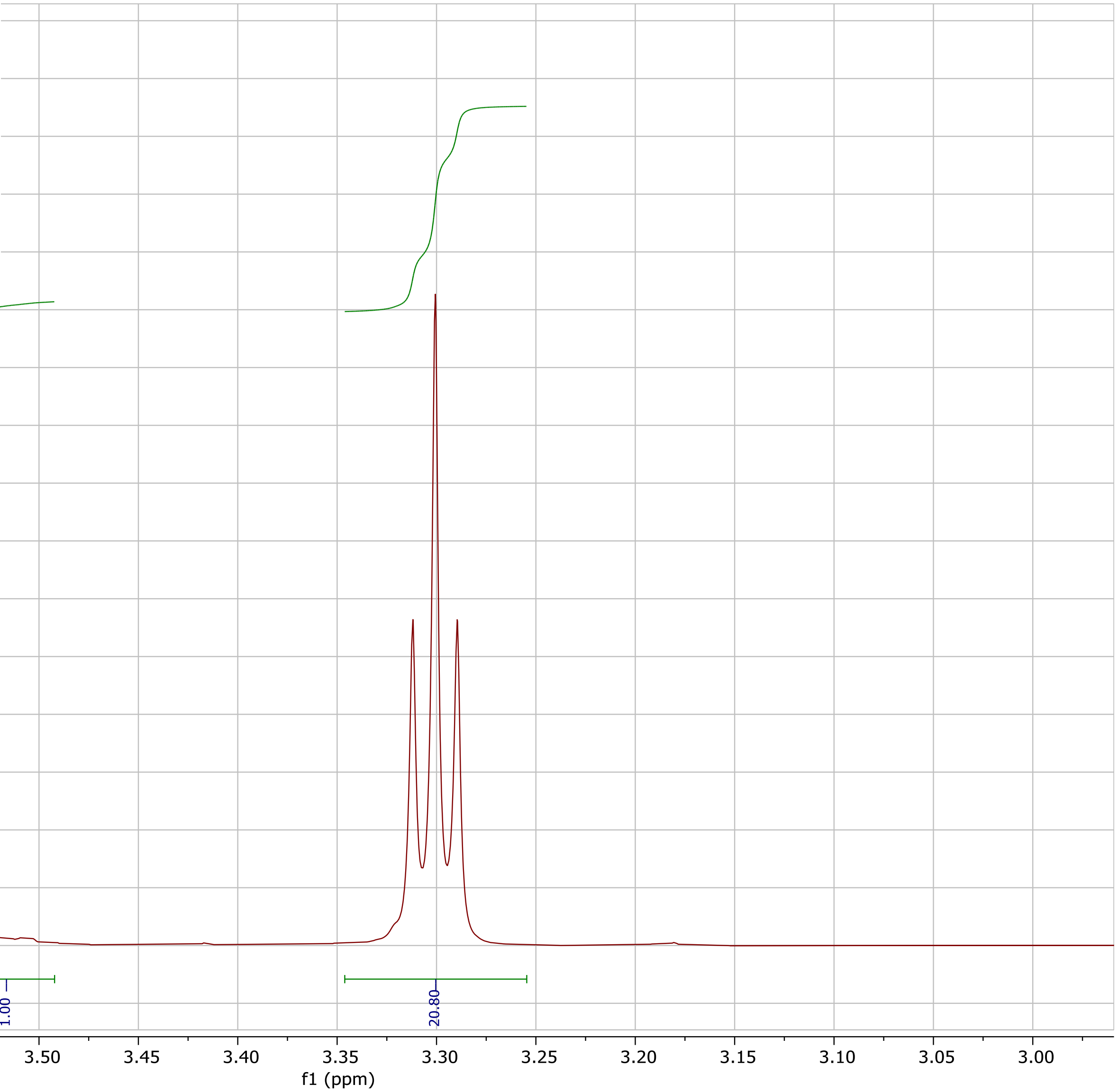

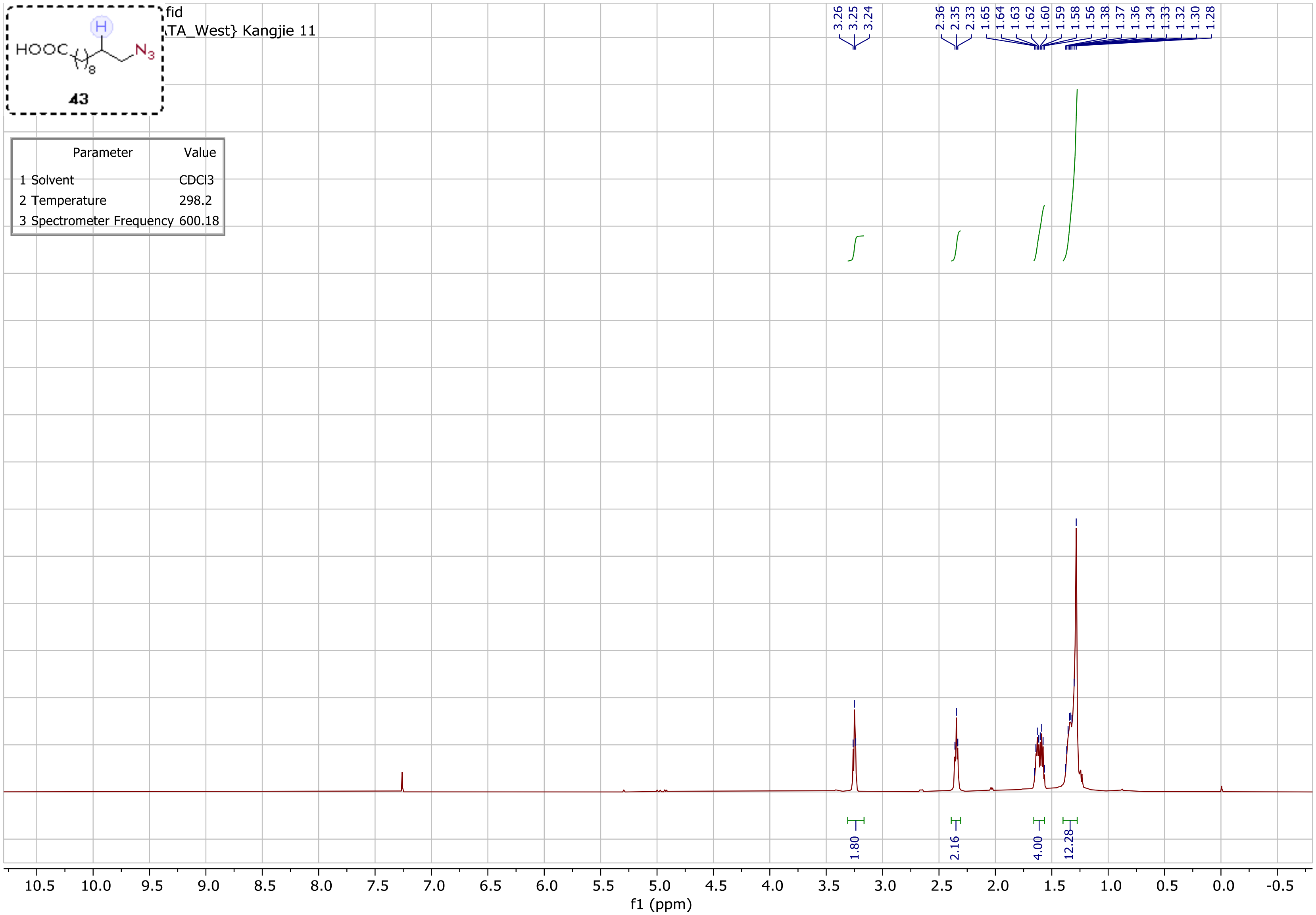

	Parameter	Value
1	Solvent	CDCl3
2	Temperature	298.2
3	Spectrometer Frequency	600.18

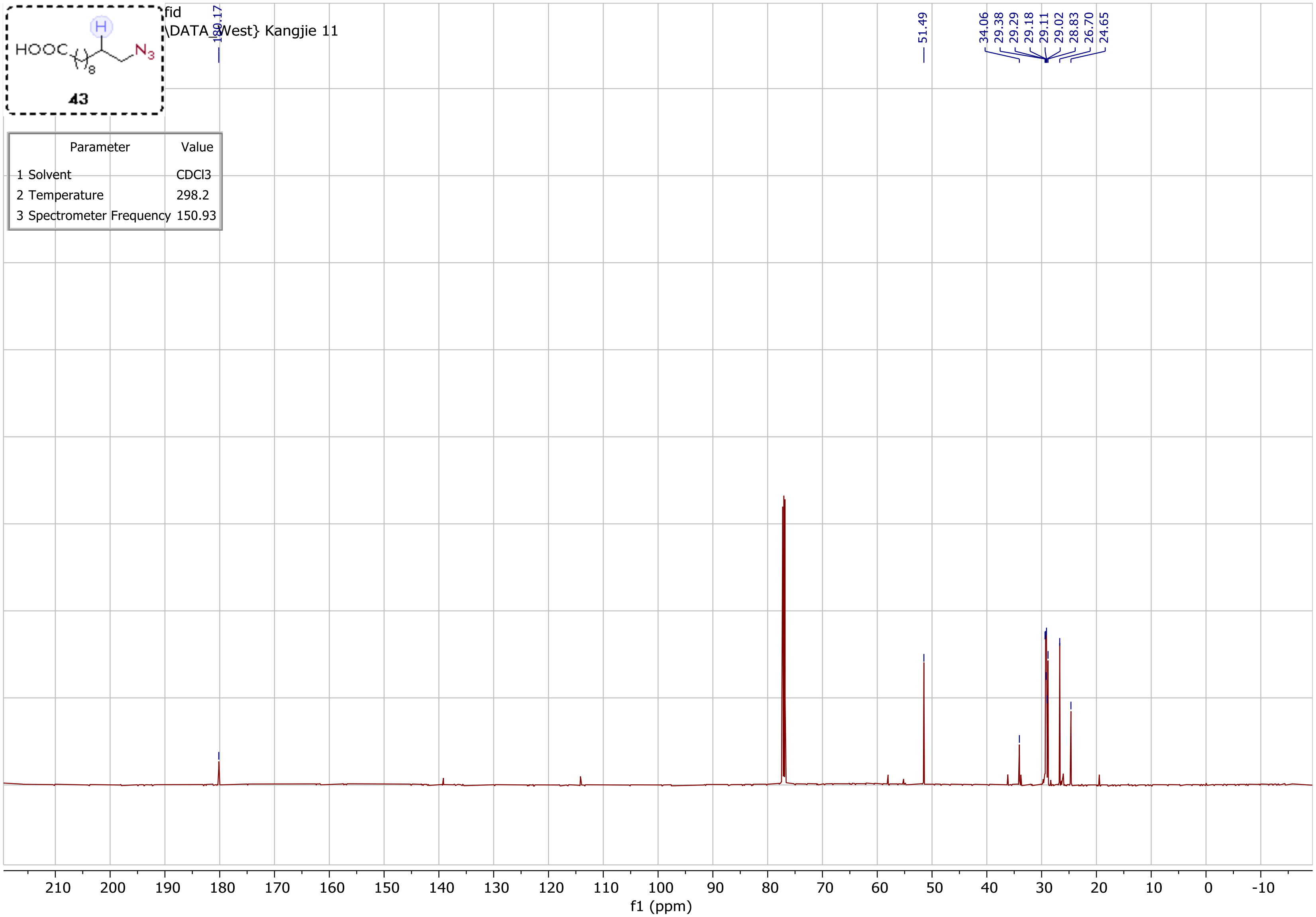
HOOC
N3
8
43
fid
\DATA_West} Kangjie 11
180.17
51.49
34.06
29.38
29.29
29.18
29.11
29.02
28.83
26.70
24.65
Parameter Value
1 Solvent CDCl3
2 Temperature 298.2
3 Spectrometer Frequency 150.93
210 200 190 180 170 160 150 140 130 120 110 100 90 80 70 60 50 40 30 20 10 0 -10
f1 (ppm)

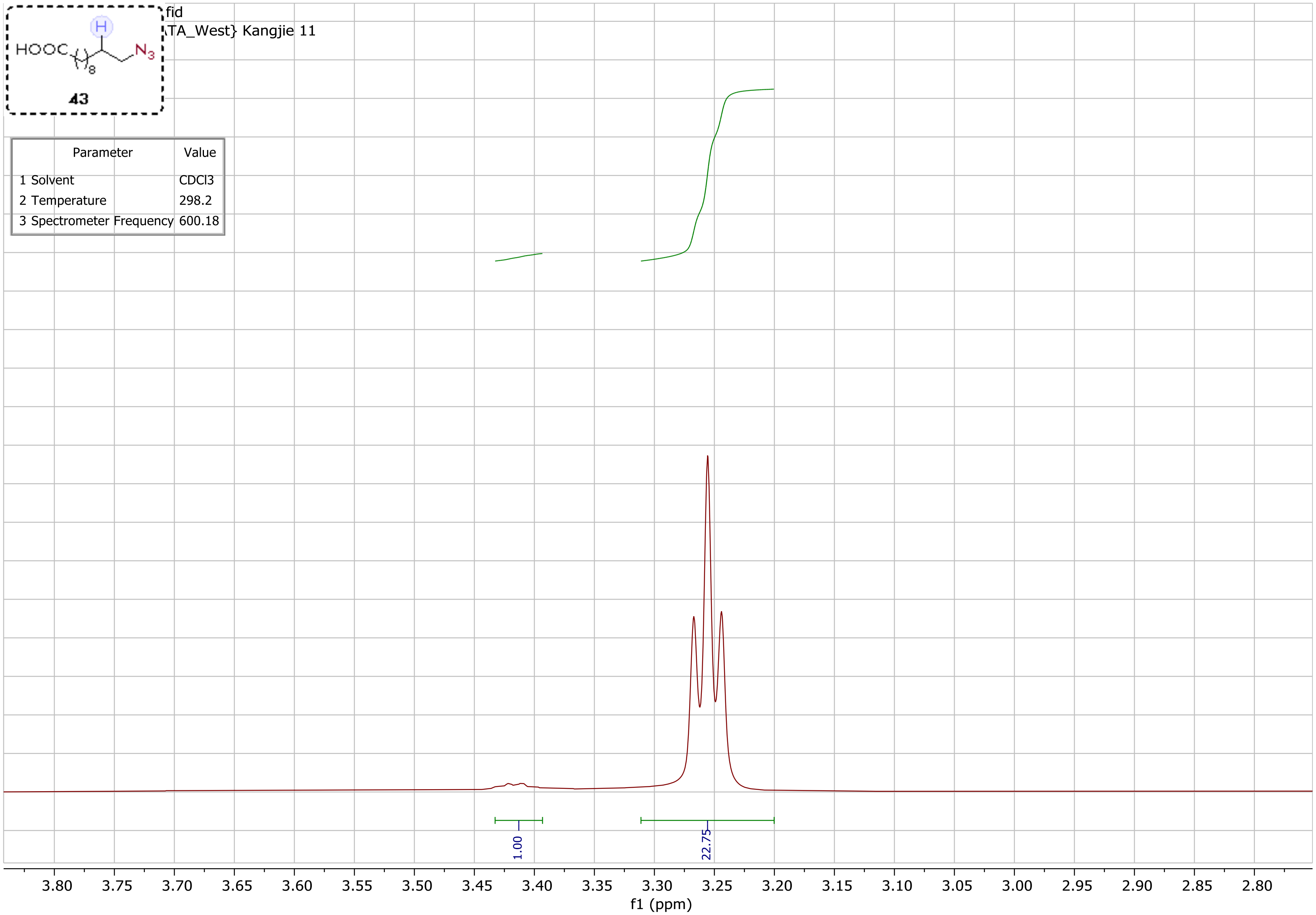

Parameter	Value
1 Solvent	CDCl3
2 Temperature	298.2
3 Spectrometer Frequency	600.18

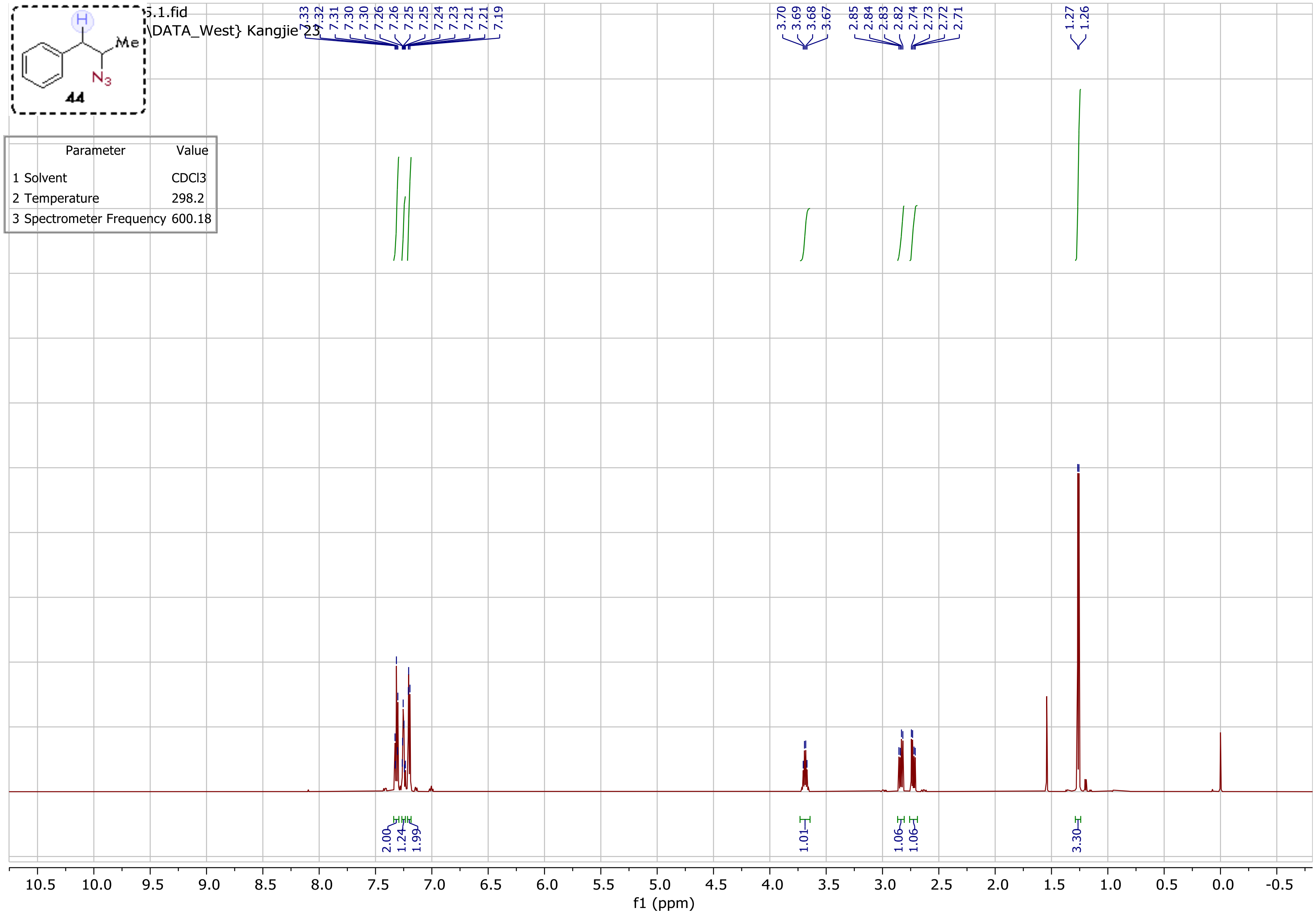

	Parameter	Value
1	Solvent	CDCl3
2	Temperature	298.2
3	Spectrometer Frequency	600.18

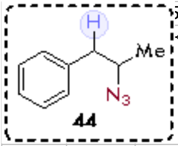

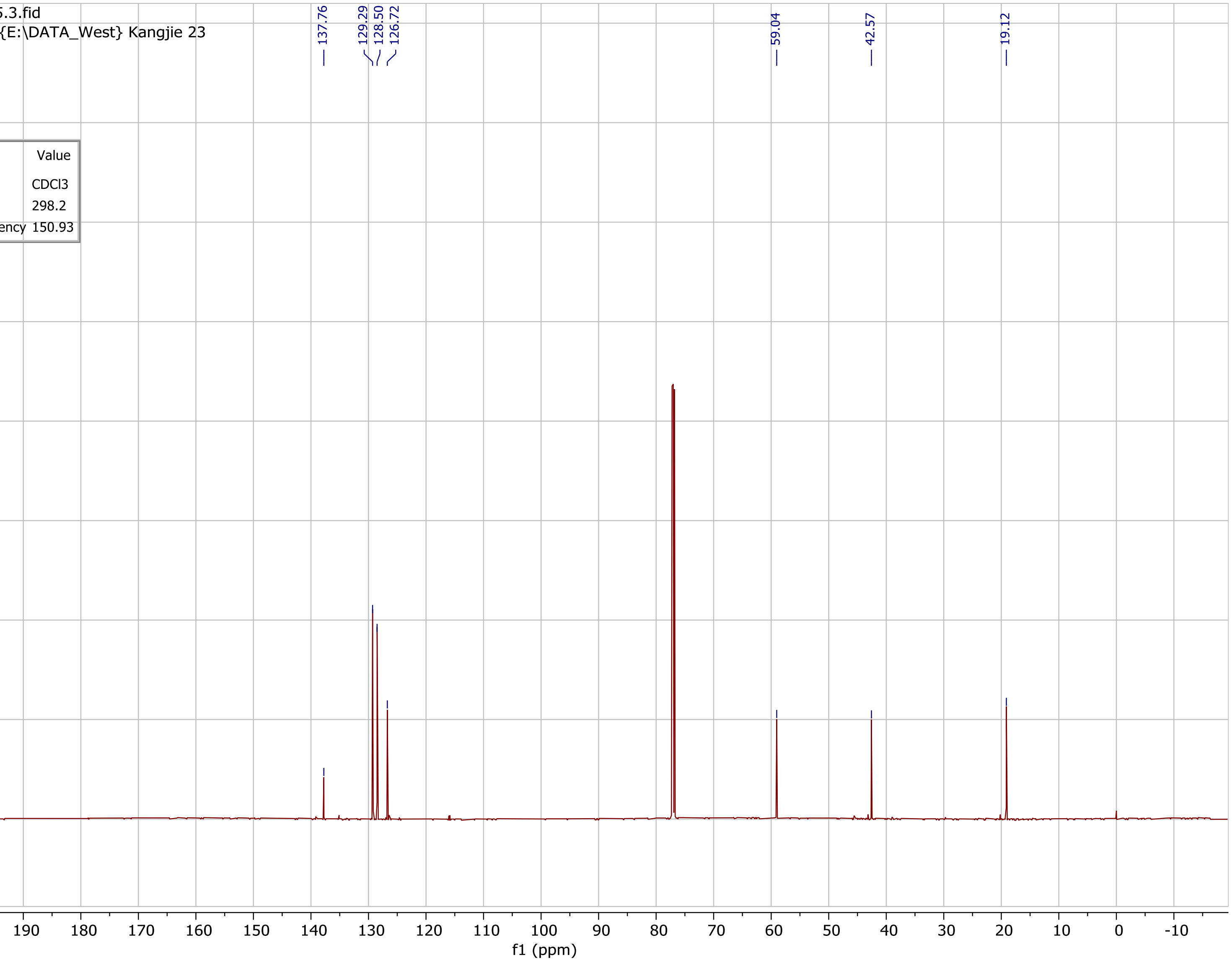

Parameter	Value
1 Solvent	CDCl3
2 Temperature	298.2
3 Spectrometer Frequency	150.93

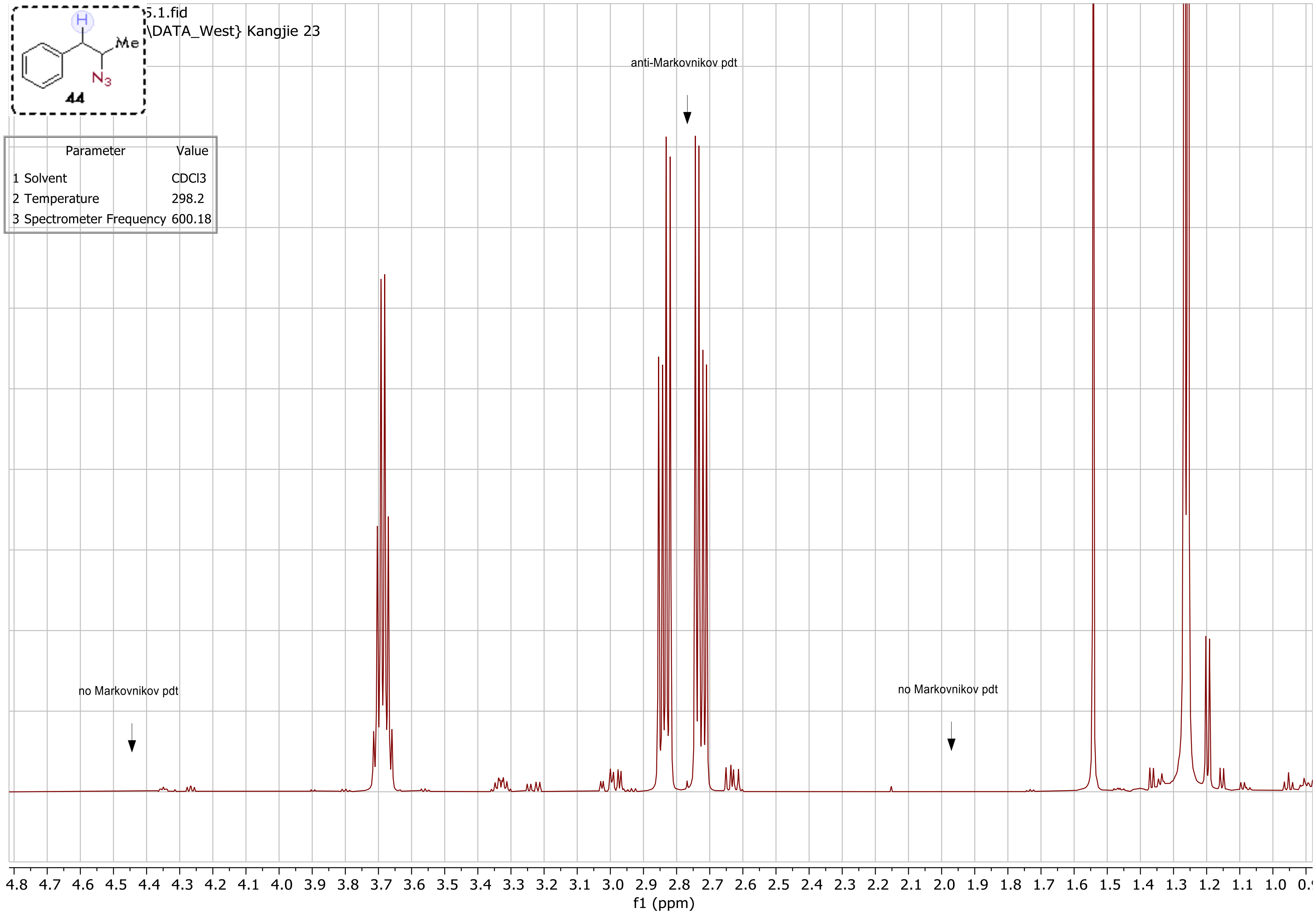

Parameter	Value
1 Solvent	CDCl3
2 Temperature	298.2
3 Spectrometer Frequency	600.18

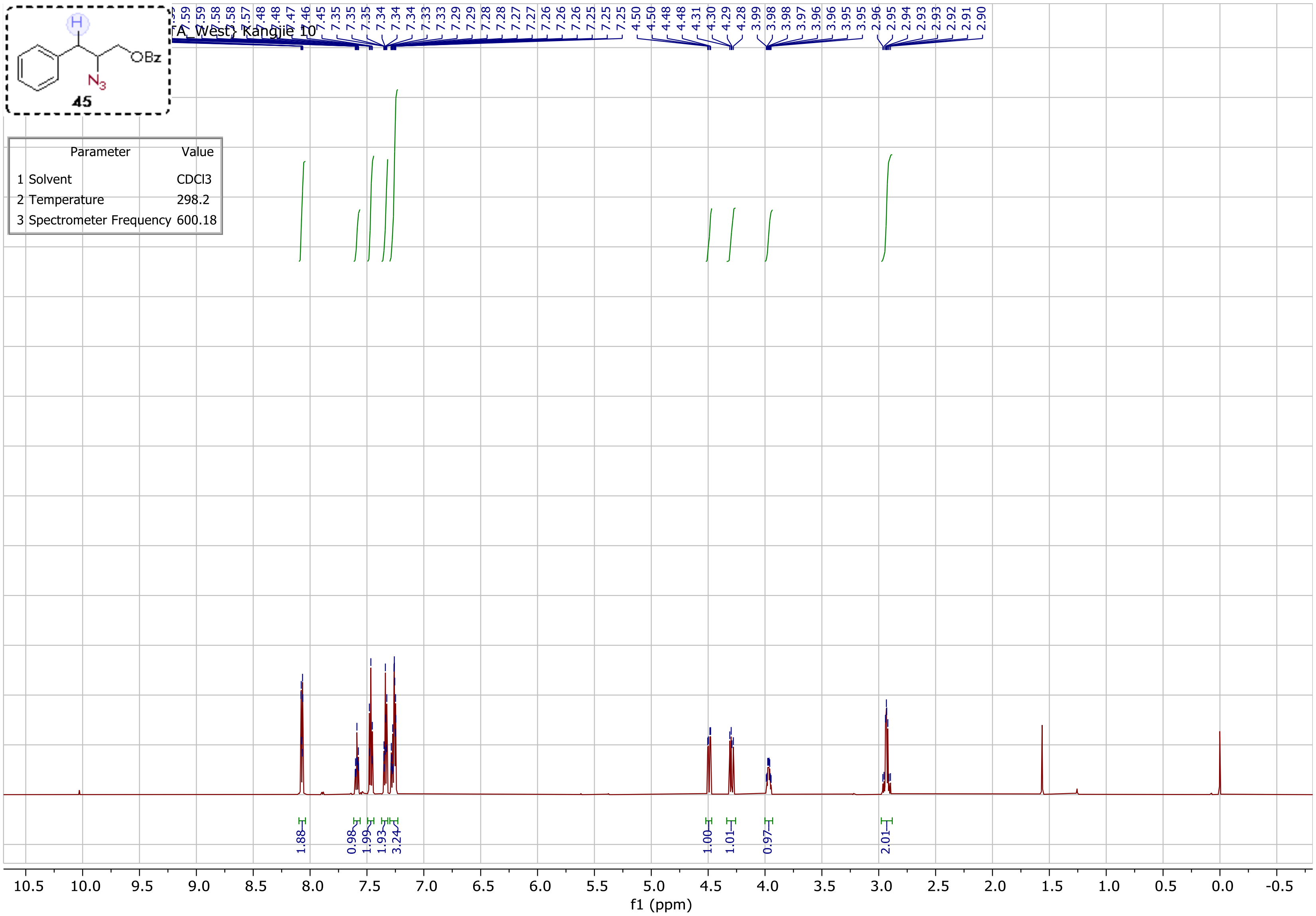
OBz
N3
45
TA_West; Kangjie 10
7.59 7.59 7.58 7.58 7.57 7.48 7.48 7.47 7.46 7.45 7.35 7.35 7.35 7.34 7.34 7.34 7.33 7.33 7.29 7.29 7.28 7.28 7.27 7.27 7.26 7.26 7.26 7.25 7.25 7.25 4.50 4.50 4.48 4.48 4.31 4.30 4.29 4.28 3.99 3.98 3.98 3.97 3.96 3.96 3.95 3.95 2.96 2.95 2.94 2.93 2.93 2.92 2.91 2.90
Parameter Value
1 Solvent CDCl3
2 Temperature 298.2
3 Spectrometer Frequency 600.18
1.88 0.98 1.99 1.93 3.24 1.00 1.01 0.97 2.01
10.5 10.0 9.5 9.0 8.5 8.0 7.5 7.0 6.5 6.0 5.5 5.0 4.5 4.0 3.5 3.0 2.5 2.0 1.5 1.0 0.5 0.0 -0.5
f1 (ppm)

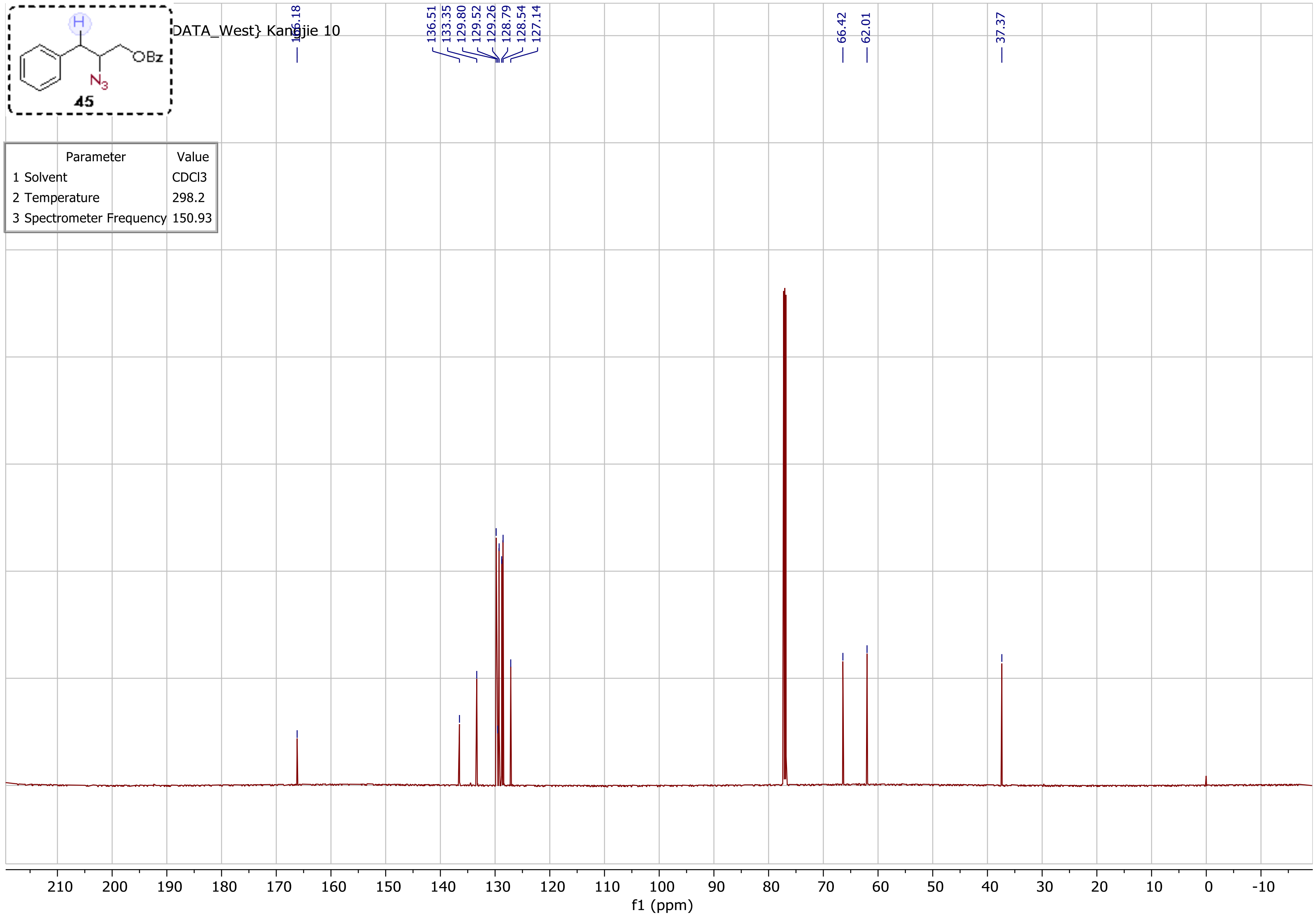

	Parameter	Value
1	Solvent	CDCl3
2	Temperature	298.2
3	Spectrometer Frequency	150.93

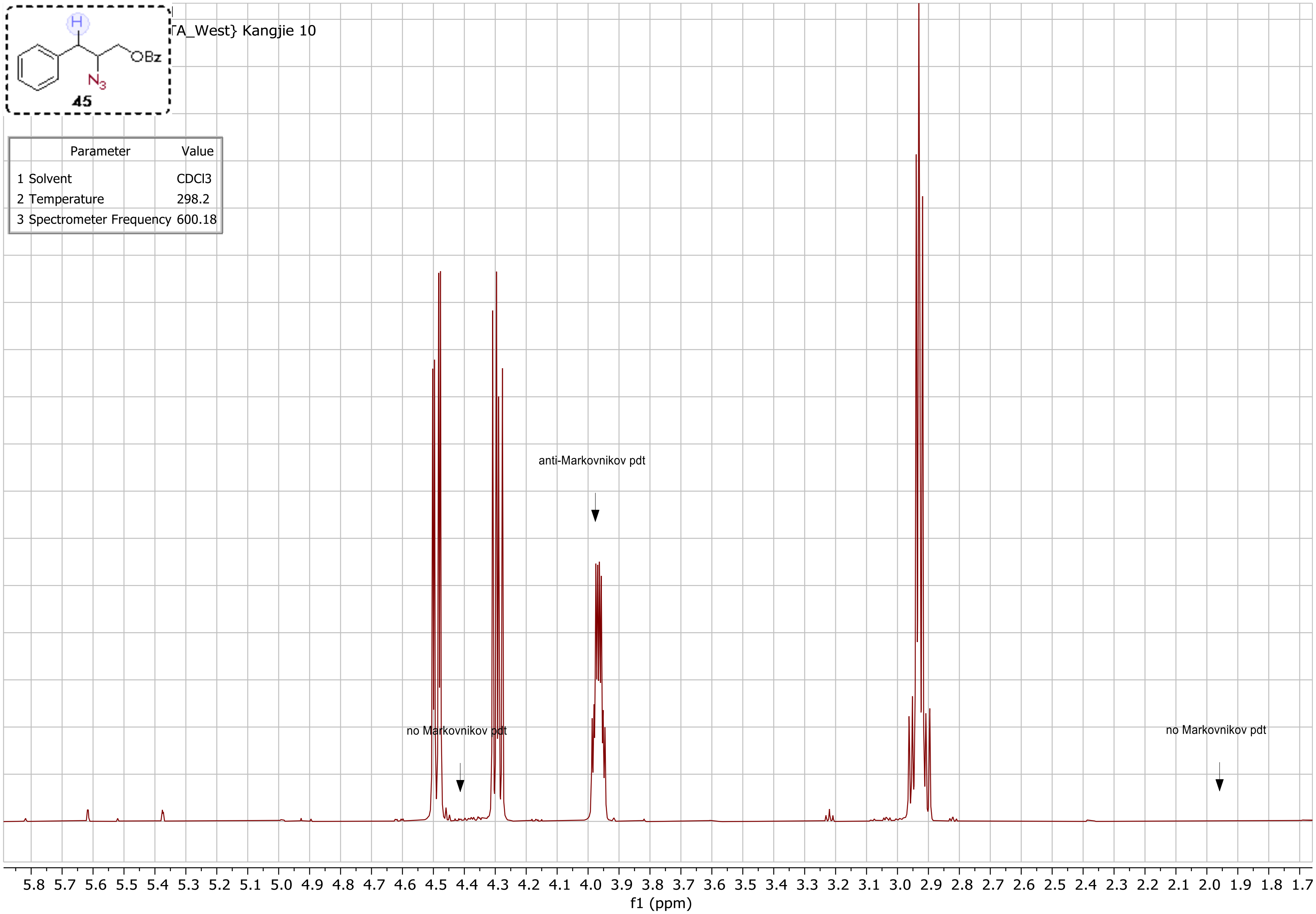

45
TA_West} Kangjie 10
Parameter Value
1 Solvent CDCl3
2 Temperature 298.2
3 Spectrometer Frequency 600.18
anti-Markovnikov pdt
no Markovnikov pdt
no Markovnikov pdt
5.8 5.7 5.6 5.5 5.4 5.3 5.2 5.1 5.0 4.9 4.8 4.7 4.6 4.5 4.4 4.3 4.2 4.1 4.0 3.9 3.8 3.7 3.6 3.5 3.4 3.3 3.2 3.1 3.0 2.9 2.8 2.7 2.6 2.5 2.4 2.3 2.2 2.1 2.0 1.9 1.8 1.7
f1 (ppm)

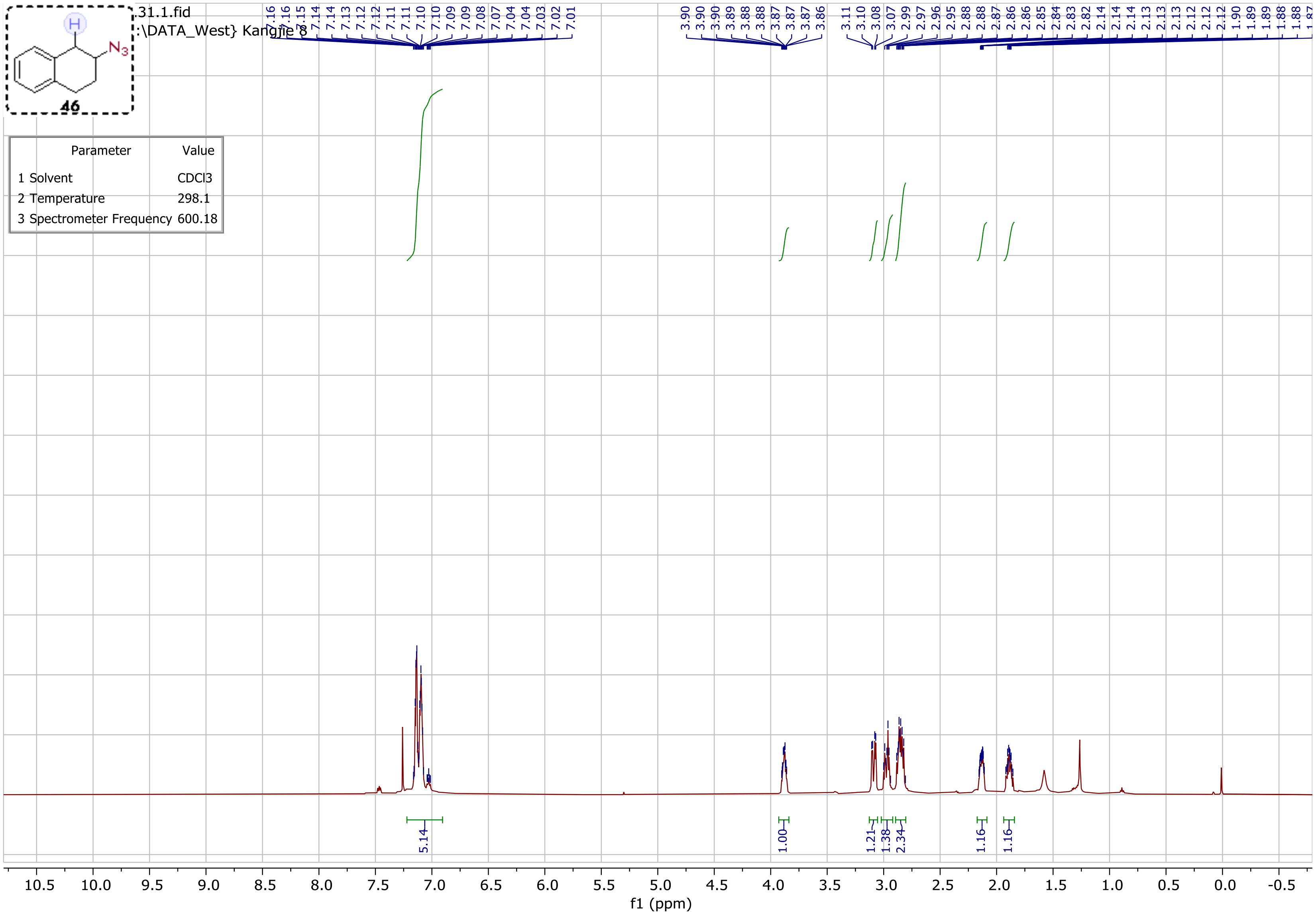
31.1.fid
:\DATA_West} Kangjie 8
46
N3
Parameter Value
1 Solvent CDCl3
2 Temperature 298.1
3 Spectrometer Frequency 600.18
5.14
1.00
1.21
1.38
2.34
1.16
1.16
f1 (ppm)

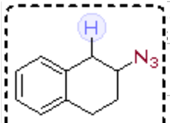

46

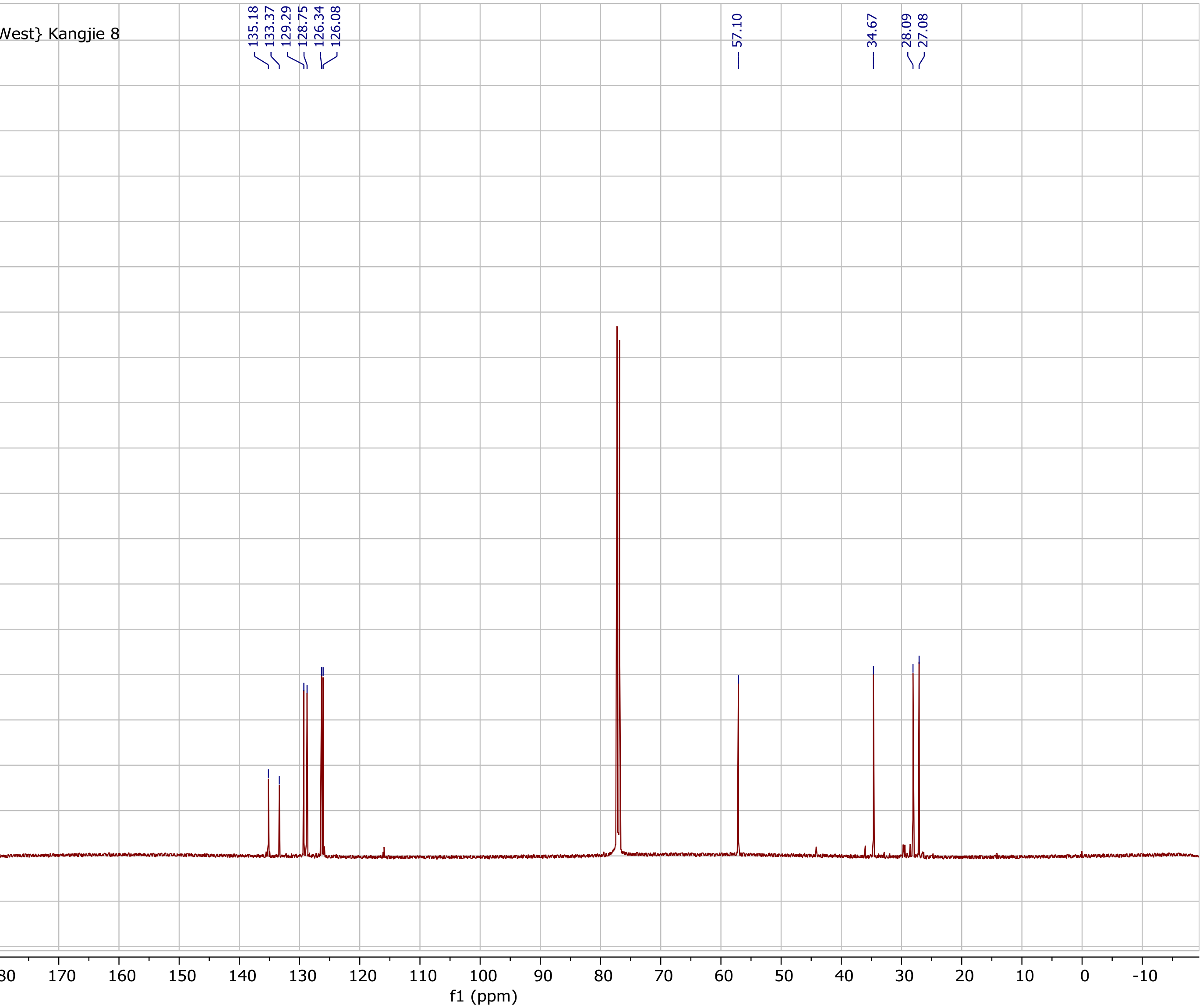

Parameter	Value
1 Solvent	CDCl3
2 Temperature	298.1
3 Spectrometer Frequency	150.93

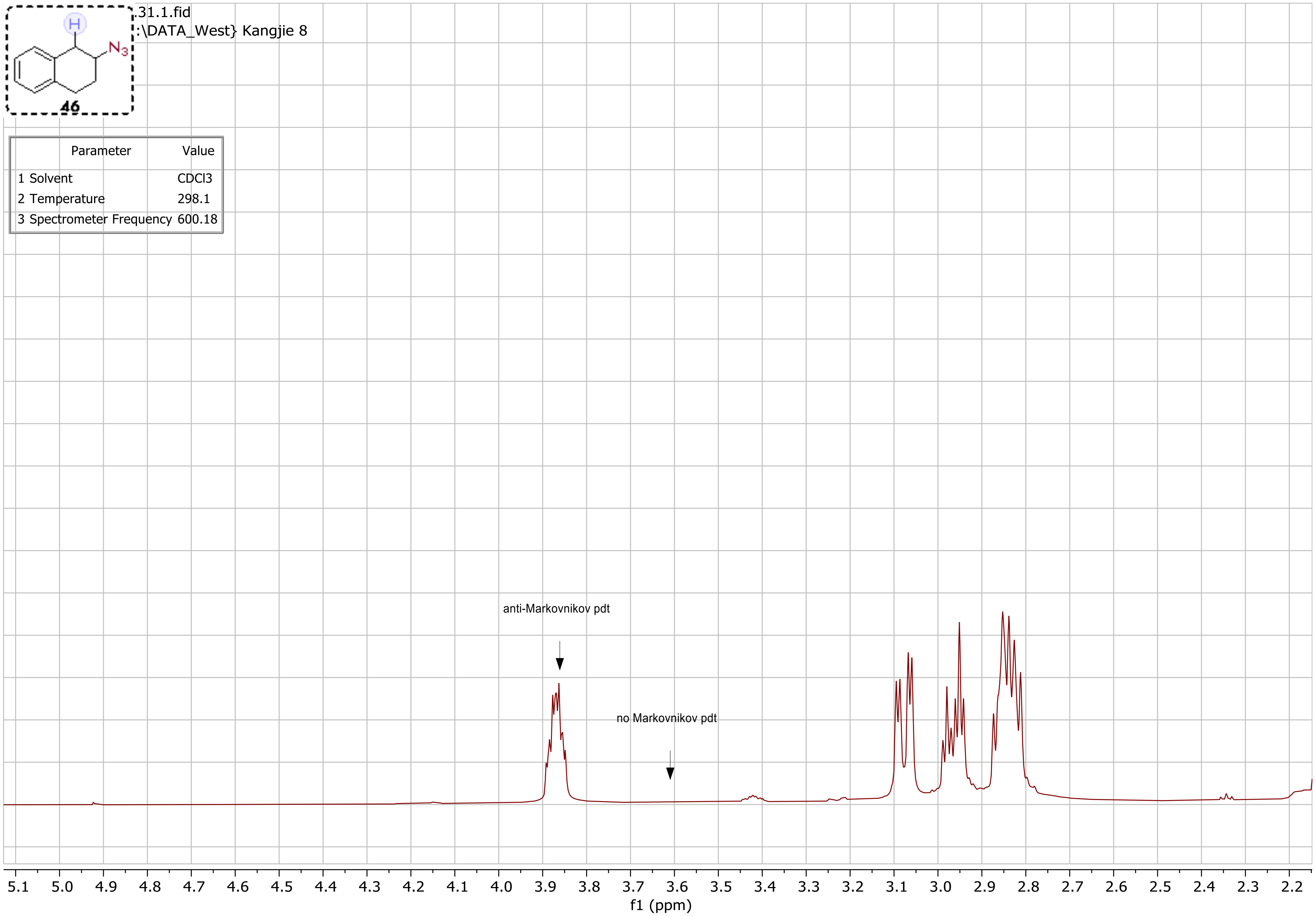
.31.1.fid
:\DATA_West} Kangjie 8
N3
46
Parameter Value
1 Solvent CDCl3
2 Temperature 298.1
3 Spectrometer Frequency 600.18
anti-Markovnikov pdt
no Markovnikov pdt
5.1 5.0 4.9 4.8 4.7 4.6 4.5 4.4 4.3 4.2 4.1 4.0 3.9 3.8 3.7 3.6 3.5 3.4 3.3 3.2 3.1 3.0 2.9 2.8 2.7 2.6 2.5 2.4 2.3 2.2
f1 (ppm)

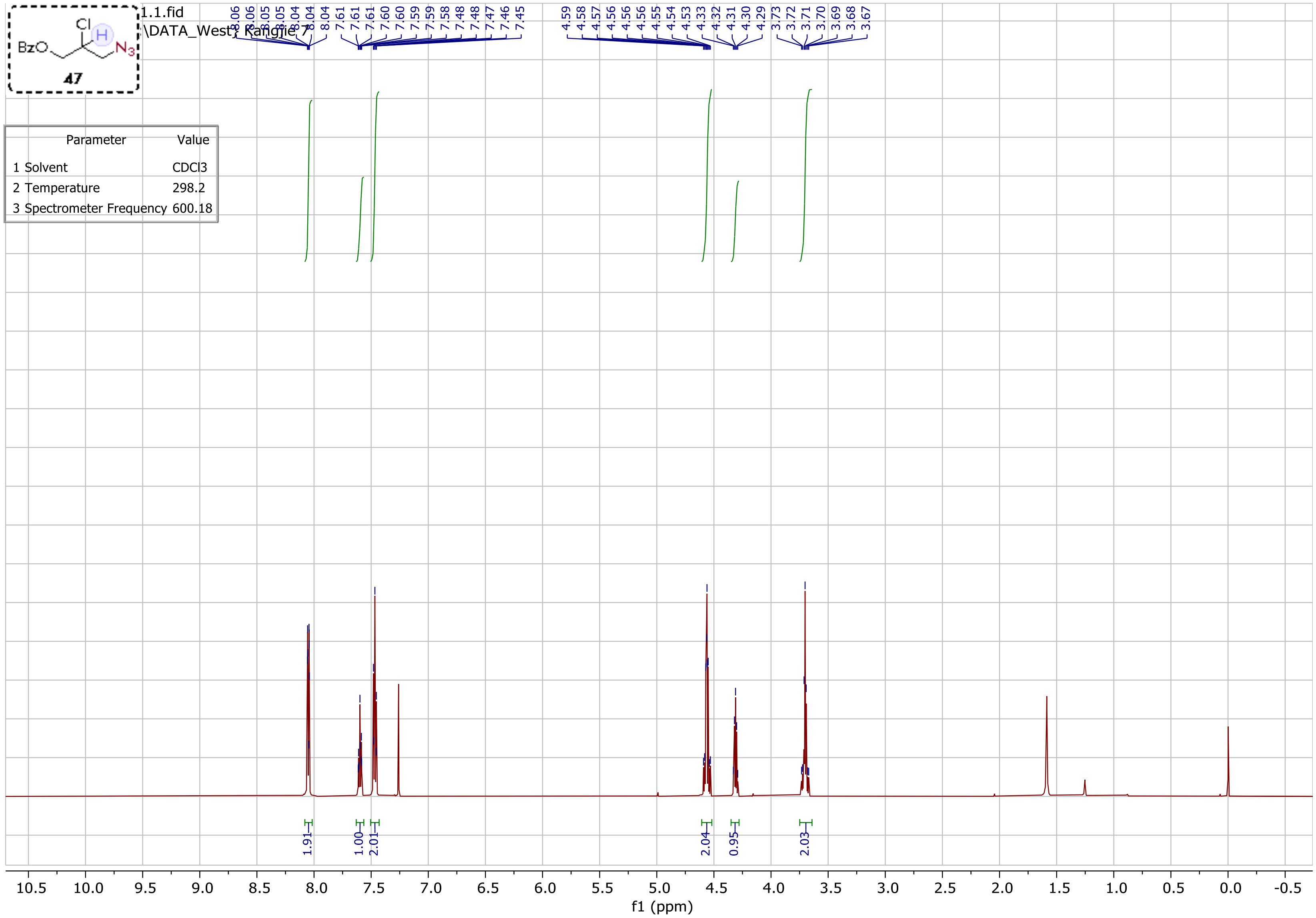
1.1.fid
\DATA_West; Kangjie 7
Cl
H
BzO
N3
47
Parameter Value
1 Solvent CDCl3
2 Temperature 298.2
3 Spectrometer Frequency 600.18
f1 (ppm)

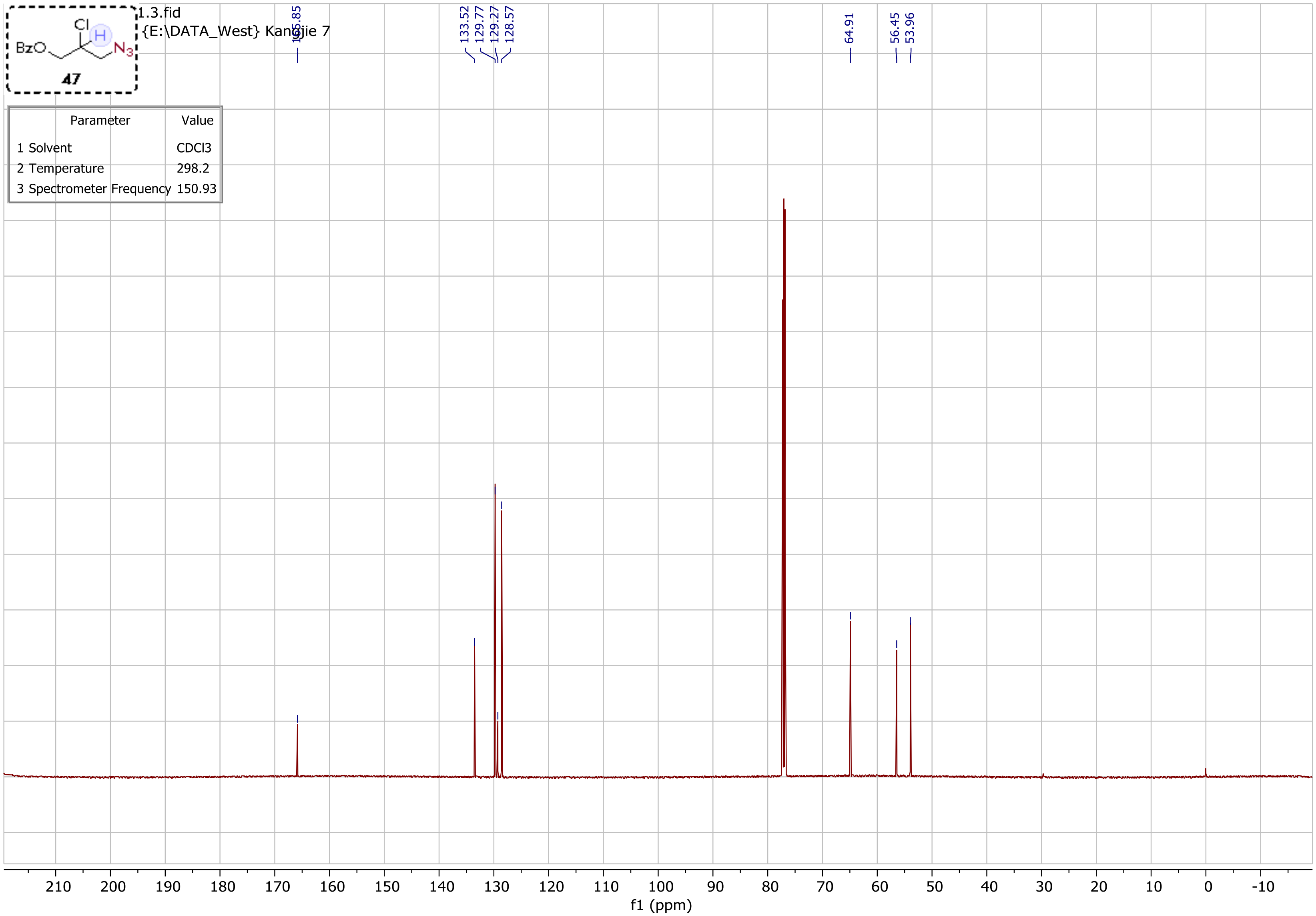

Cl
H
BzO
N3
47
1.3.fid
{E:\DATA_West} Kangjie 7
Parameter | Value
1 Solvent | CDCl3
2 Temperature | 298.2
3 Spectrometer Frequency | 150.93
133.52
129.77
129.27
128.57
64.91
56.45
53.96
210 200 190 180 170 160 150 140 130 120 110 100 90 80 70 60 50 40 30 20 10 0 -10
f1 (ppm)

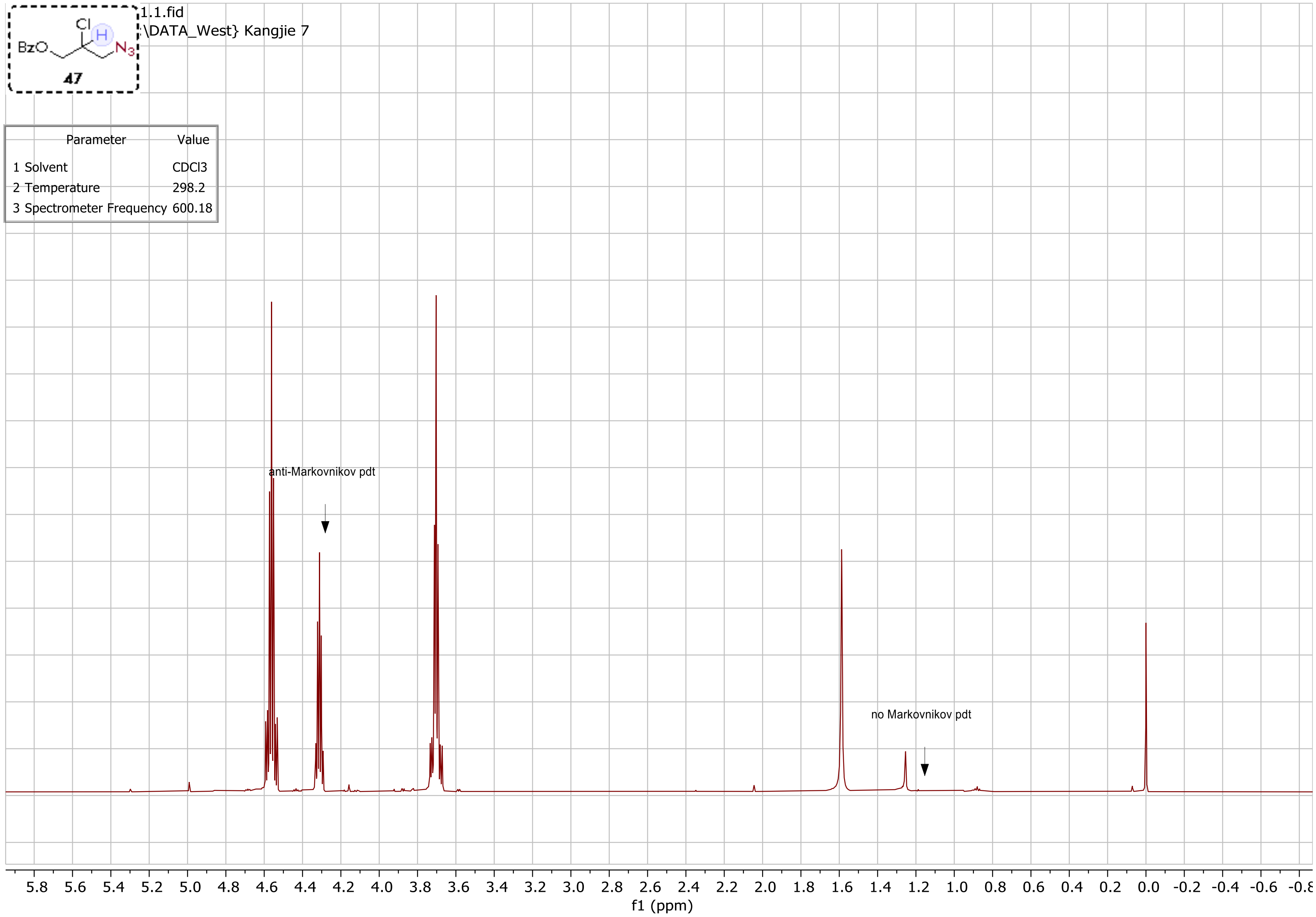
1.1.fid
\DATA_West} Kangjie 7
BzO
Cl
H
N3
47
Parameter Value
1 Solvent CDCl3
2 Temperature 298.2
3 Spectrometer Frequency 600.18
anti-Markovnikov pdt
no Markovnikov pdt
f1 (ppm)

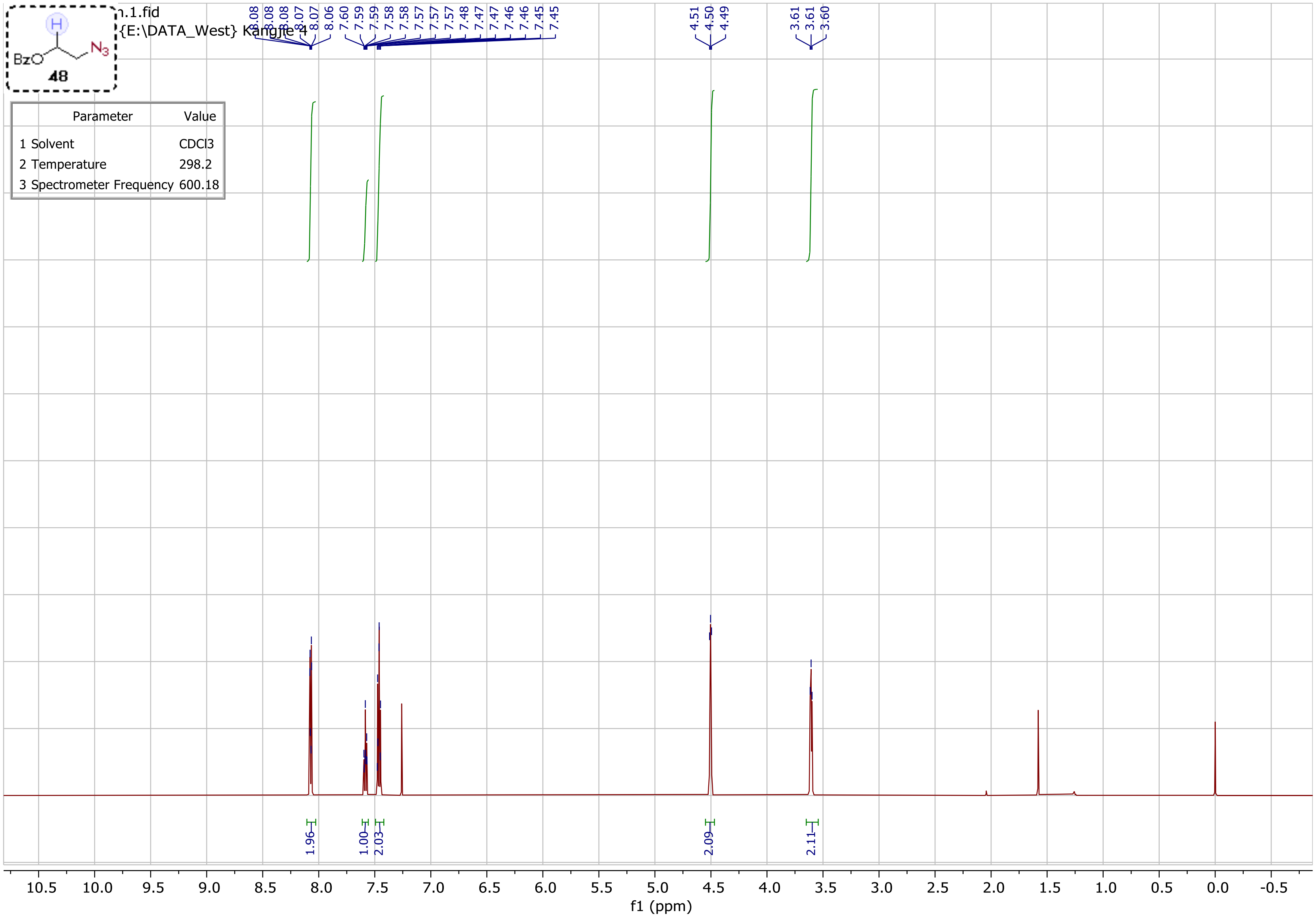
BzO N3
48
…n.1.fid
{E:\DATA_West}
Parameter Value
1 Solvent CDCl3
2 Temperature 298.2
3 Spectrometer Frequency 600.18
8.08 8.08 8.08 8.07 8.07 8.06 7.60 7.59 7.59 7.58 7.58 7.57 7.57 7.57 7.48 7.47 7.47 7.46 7.46 7.45 7.45
4.51 4.50 4.49
3.61 3.61 3.60
1.96 1.00 2.03 2.09 2.11
10.5 10.0 9.5 9.0 8.5 8.0 7.5 7.0 6.5 6.0 5.5 5.0 4.5 4.0 3.5 3.0 2.5 2.0 1.5 1.0 0.5 0.0 -0.5
f1 (ppm)

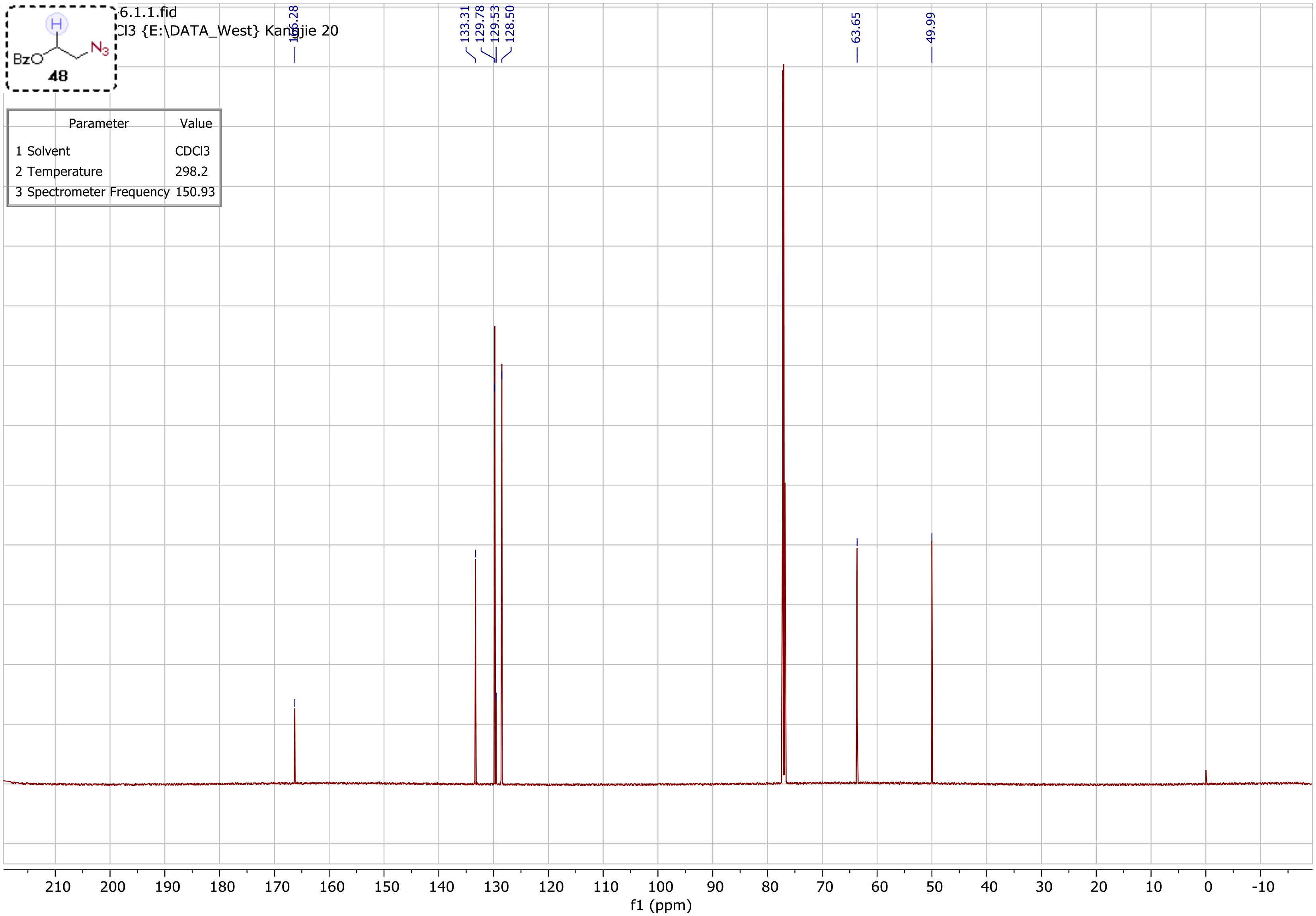

6.1.1.fid
Cl3 {E:\DATA_West} Kangjie 20
BzO
N_3
H
48
133.31
129.78
129.53
128.50
63.65
49.99
Parameter Value
1 Solvent CDCl3
2 Temperature 298.2
3 Spectrometer Frequency 150.93
210 200 190 180 170 160 150 140 130 120 110 100 90 80 70 60 50 40 30 20 10 0 -10
f1 (ppm)

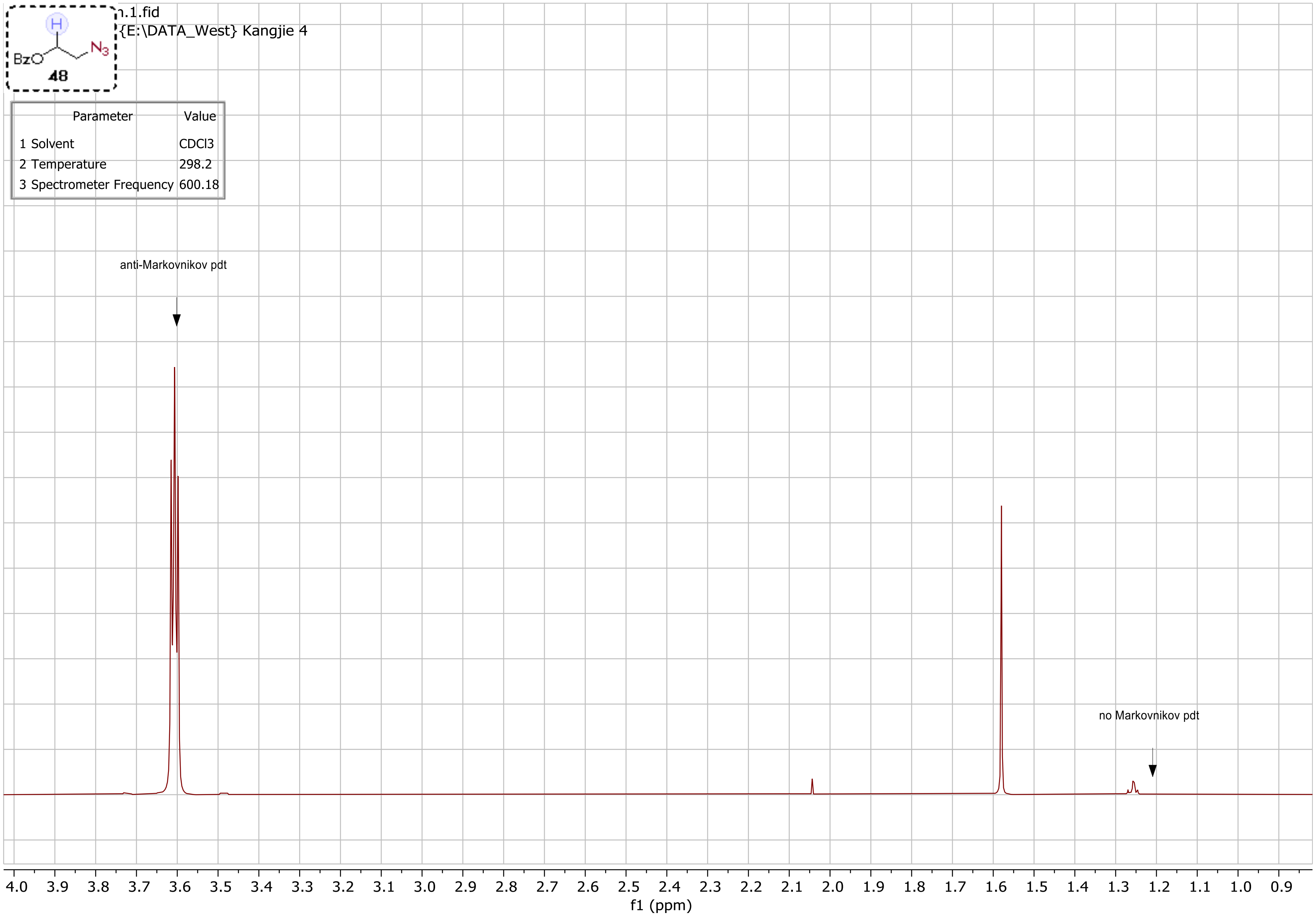

Parameter	Value
1 Solvent	CDCl3
2 Temperature	298.2
3 Spectrometer Frequency	600.18

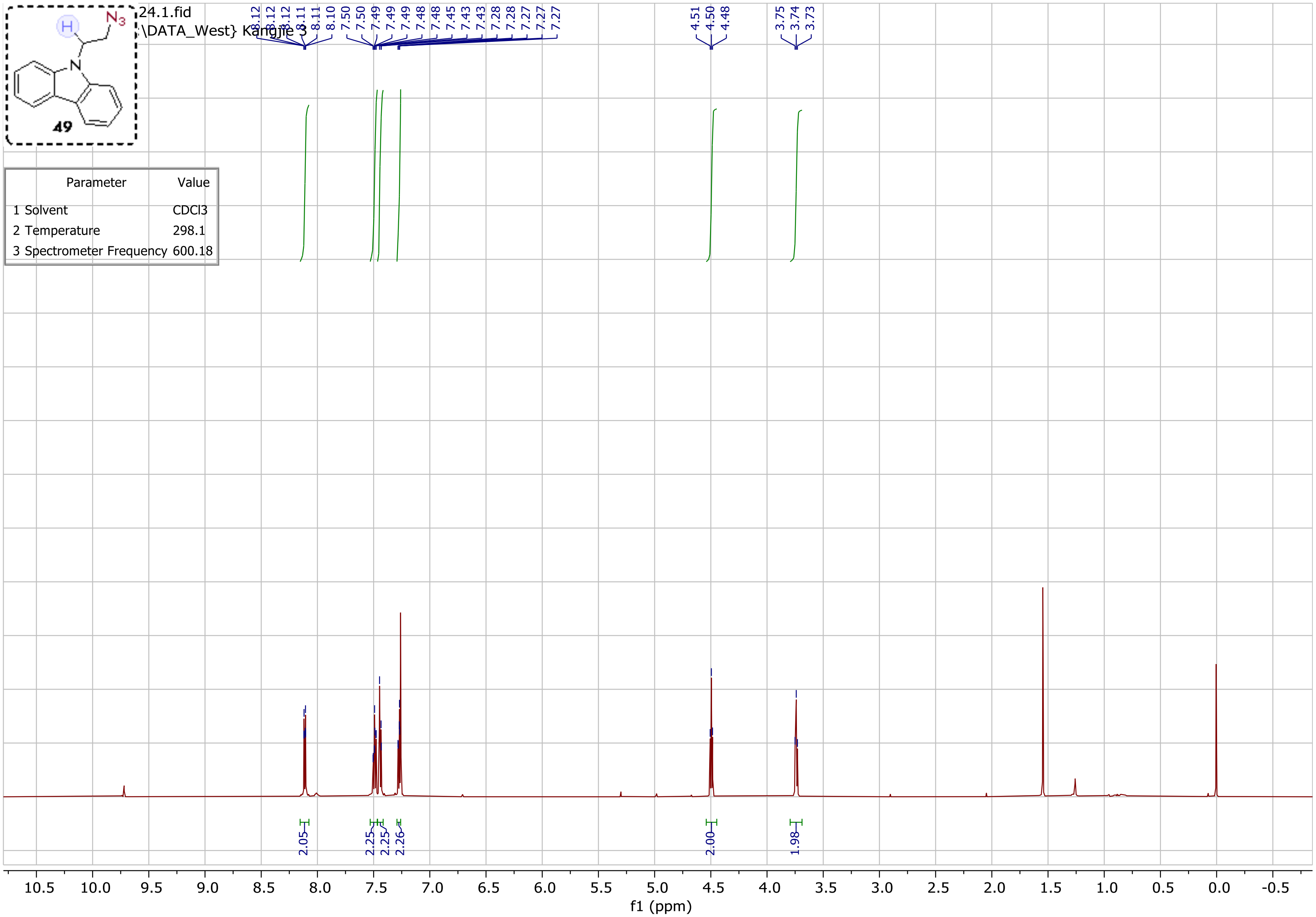

Parameter	Value
1 Solvent	CDCl3
2 Temperature	298.1
3 Spectrometer Frequency	600.18

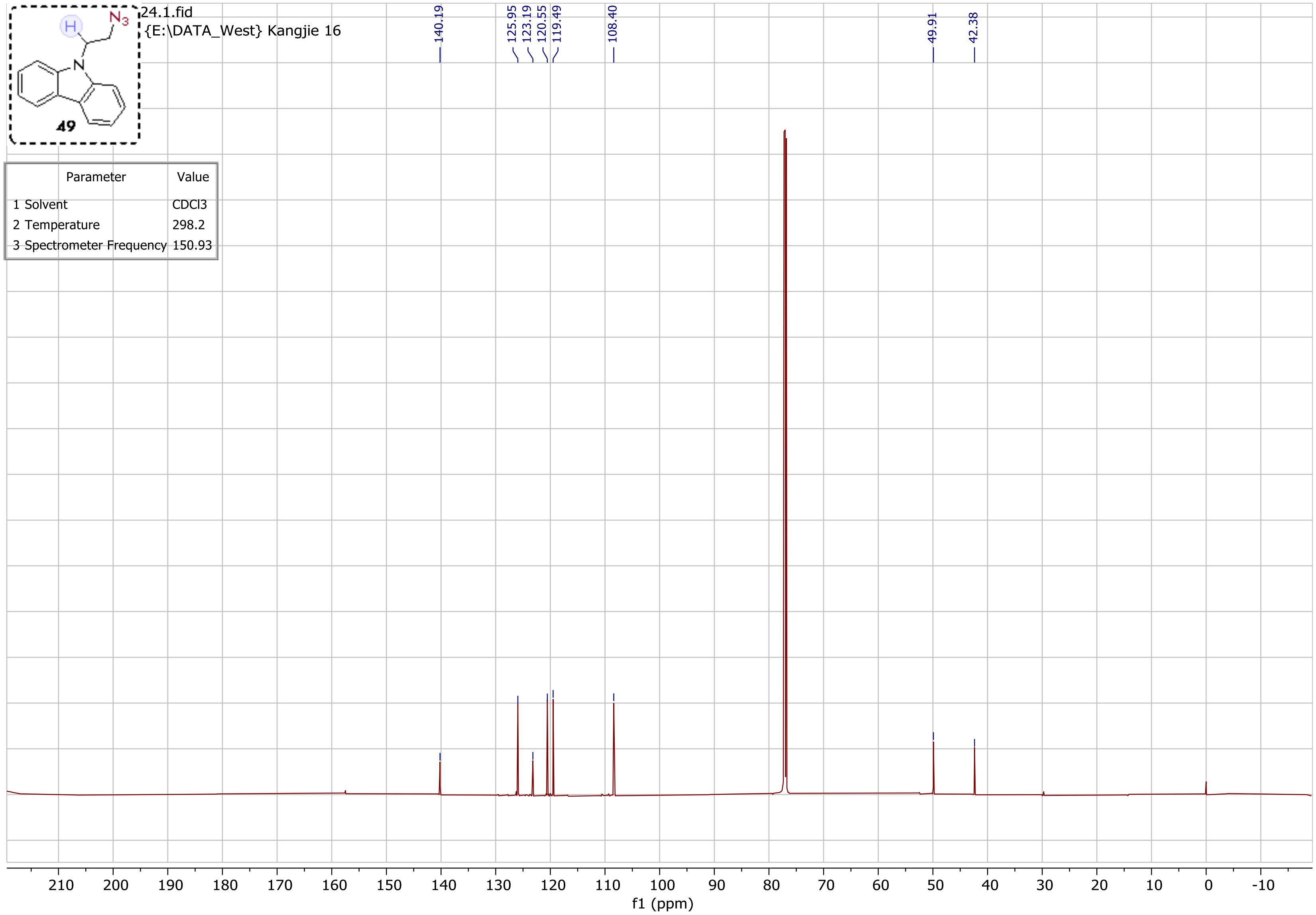

N3
H
N
49
24.1.fid
{E:\DATA_West} Kangjie 16
Parameter Value
1 Solvent CDCl3
2 Temperature 298.2
3 Spectrometer Frequency 150.93
140.19
125.95
123.19
120.55
119.49
108.40
49.91
42.38
210 200 190 180 170 160 150 140 130 120 110 100 90 80 70 60 50 40 30 20 10 0 -10
f1 (ppm)

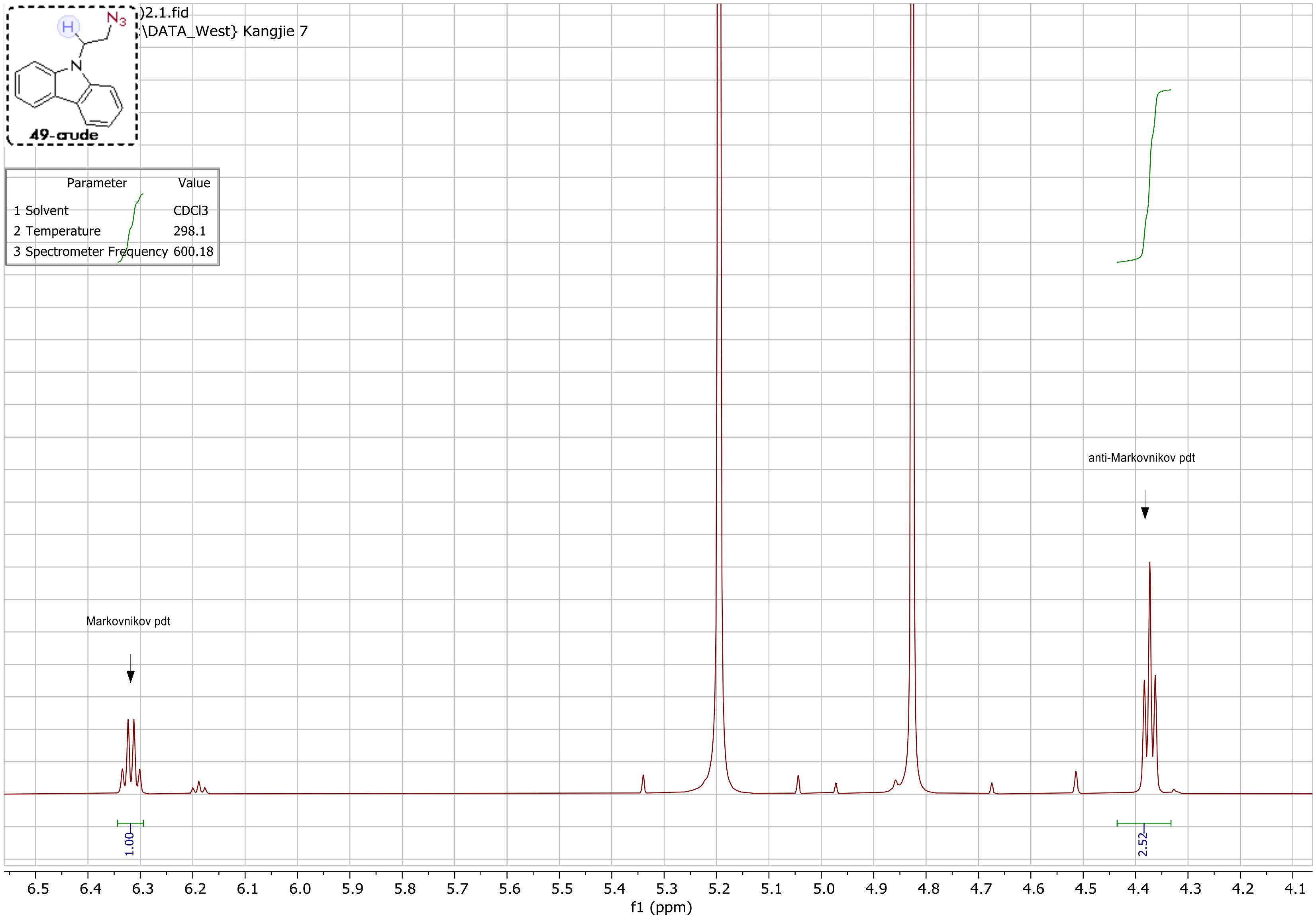

49-crude
)2.1.fid
\DATA_West} Kangjie 7
Parameter Value
1 Solvent CDCl3
2 Temperature 298.1
3 Spectrometer Frequency 600.18
Markovnikov pdt
anti-Markovnikov pdt
1.00
2.52
6.5 6.4 6.3 6.2 6.1 6.0 5.9 5.8 5.7 5.6 5.5 5.4 5.3 5.2 5.1 5.0 4.9 4.8 4.7 4.6 4.5 4.4 4.3 4.2 4.1
f1 (ppm)

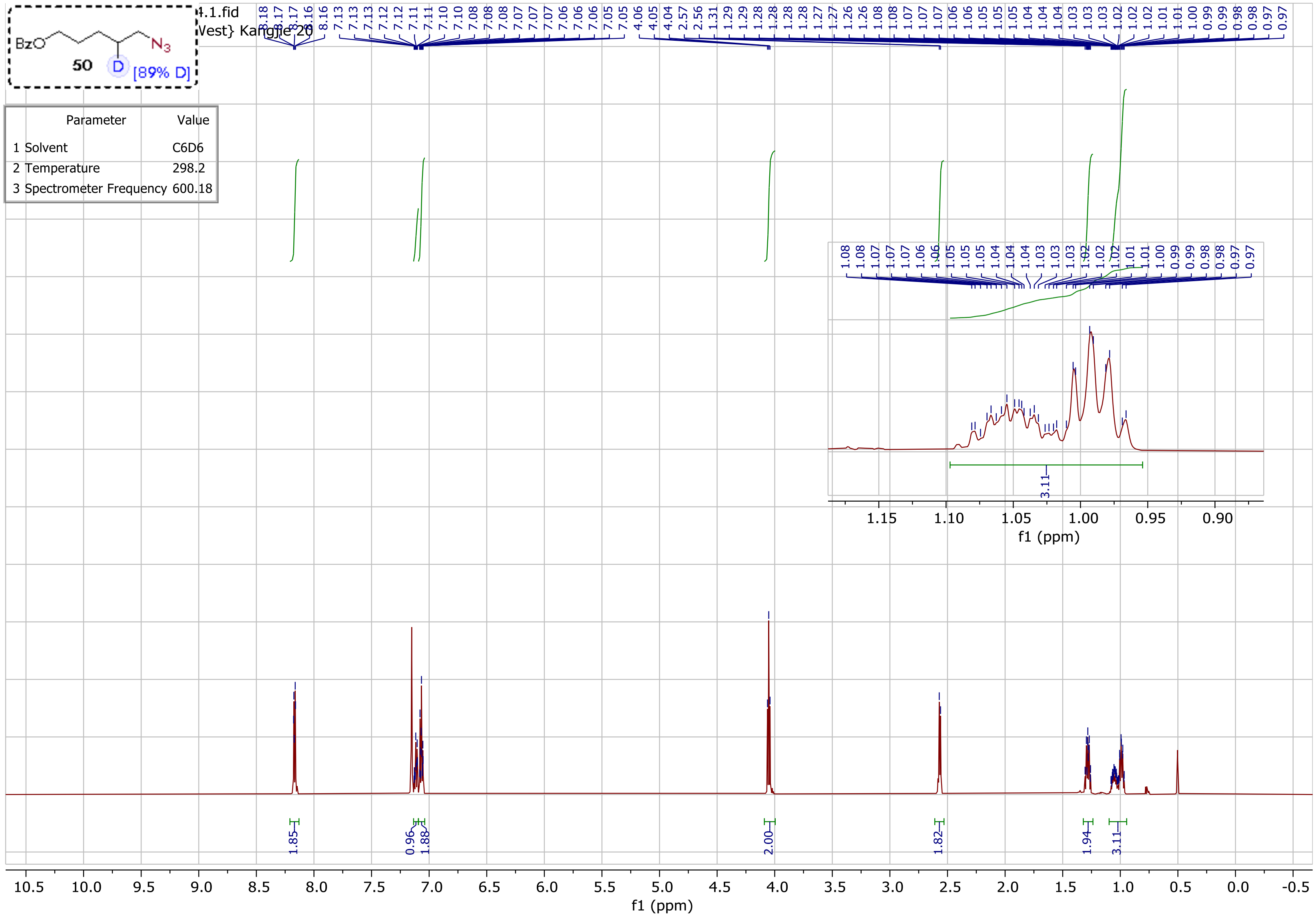

	Parameter	Value
1	Solvent	C6D6
2	Temperature	298.2
3	Spectrometer Frequency	600.18

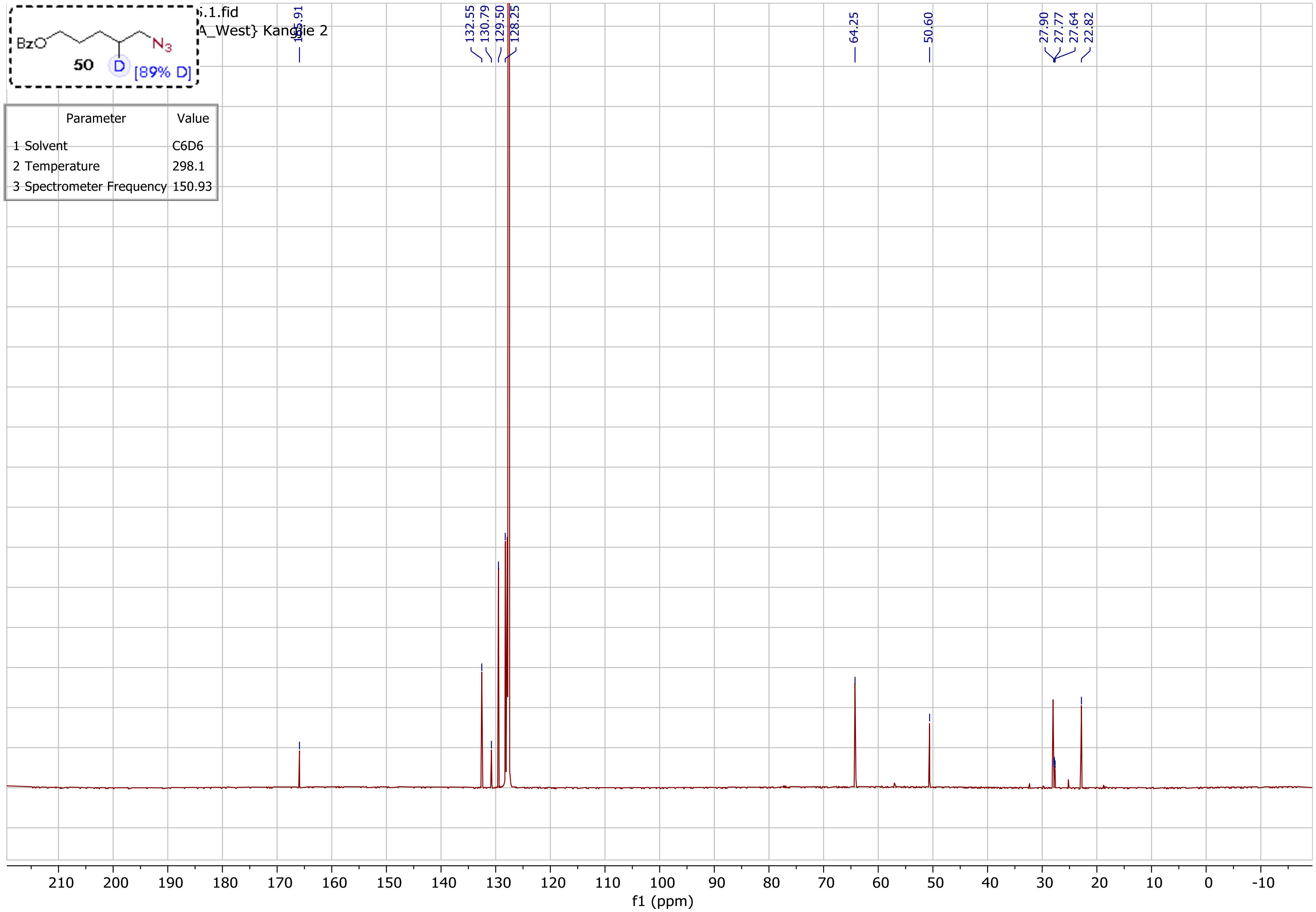

.1.fid
A_West} Kangjie 2
BzO
N3
50
D
[89% D]
Parameter | Value
1 Solvent | C6D6
2 Temperature | 298.1
3 Spectrometer Frequency | 150.93
132.55
130.79
129.50
128.25
64.25
50.60
27.90
27.77
27.64
22.82
f1 (ppm)

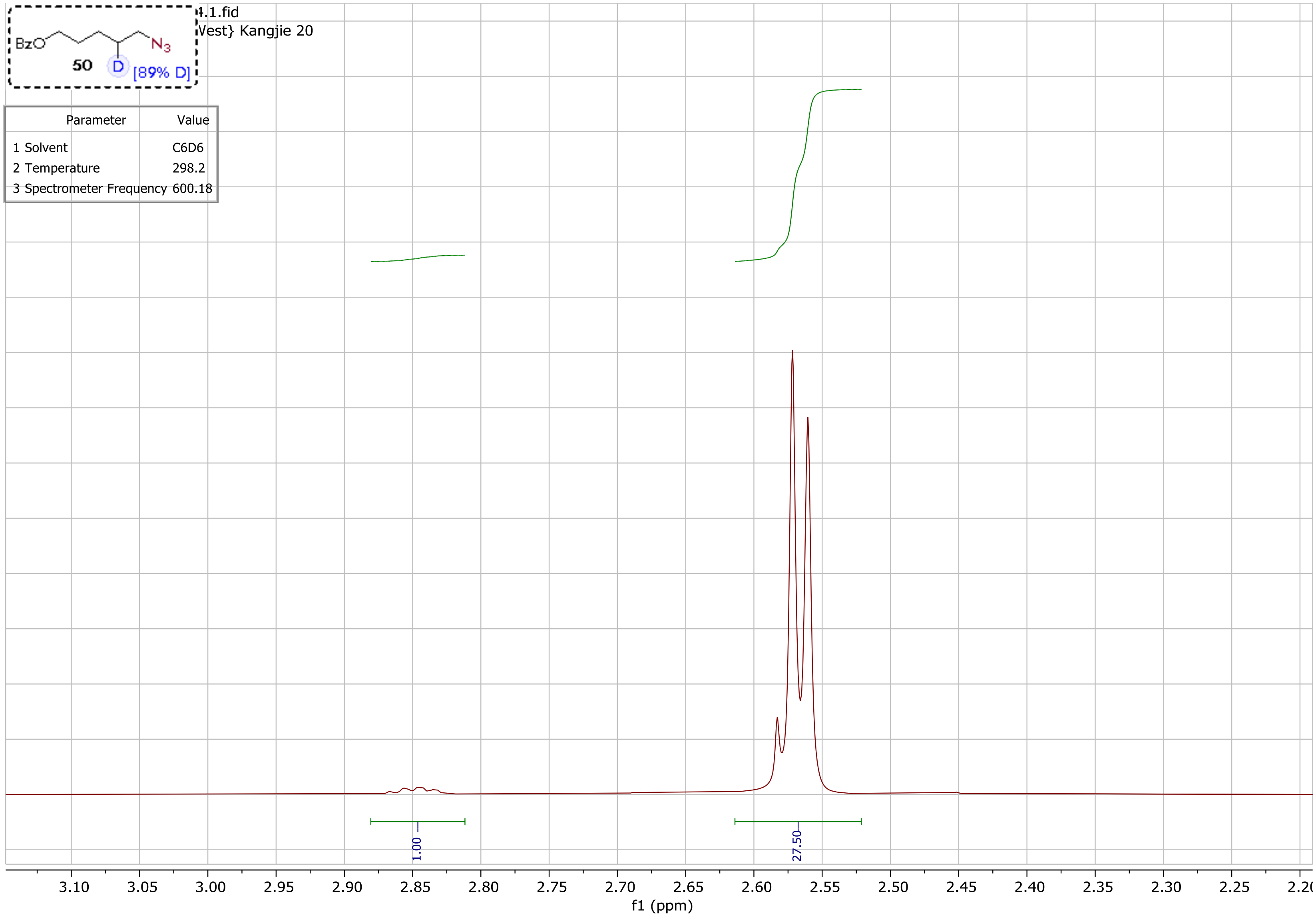

BzO
N_3
50
D
[89% D]
4.1.fid
Vest} Kangjie 20
Parameter Value
1 Solvent C6D6
2 Temperature 298.2
3 Spectrometer Frequency 600.18
1.00
27.50
3.10 3.05 3.00 2.95 2.90 2.85 2.80 2.75 2.70 2.65 2.60 2.55 2.50 2.45 2.40 2.35 2.30 2.25
f1 (ppm)

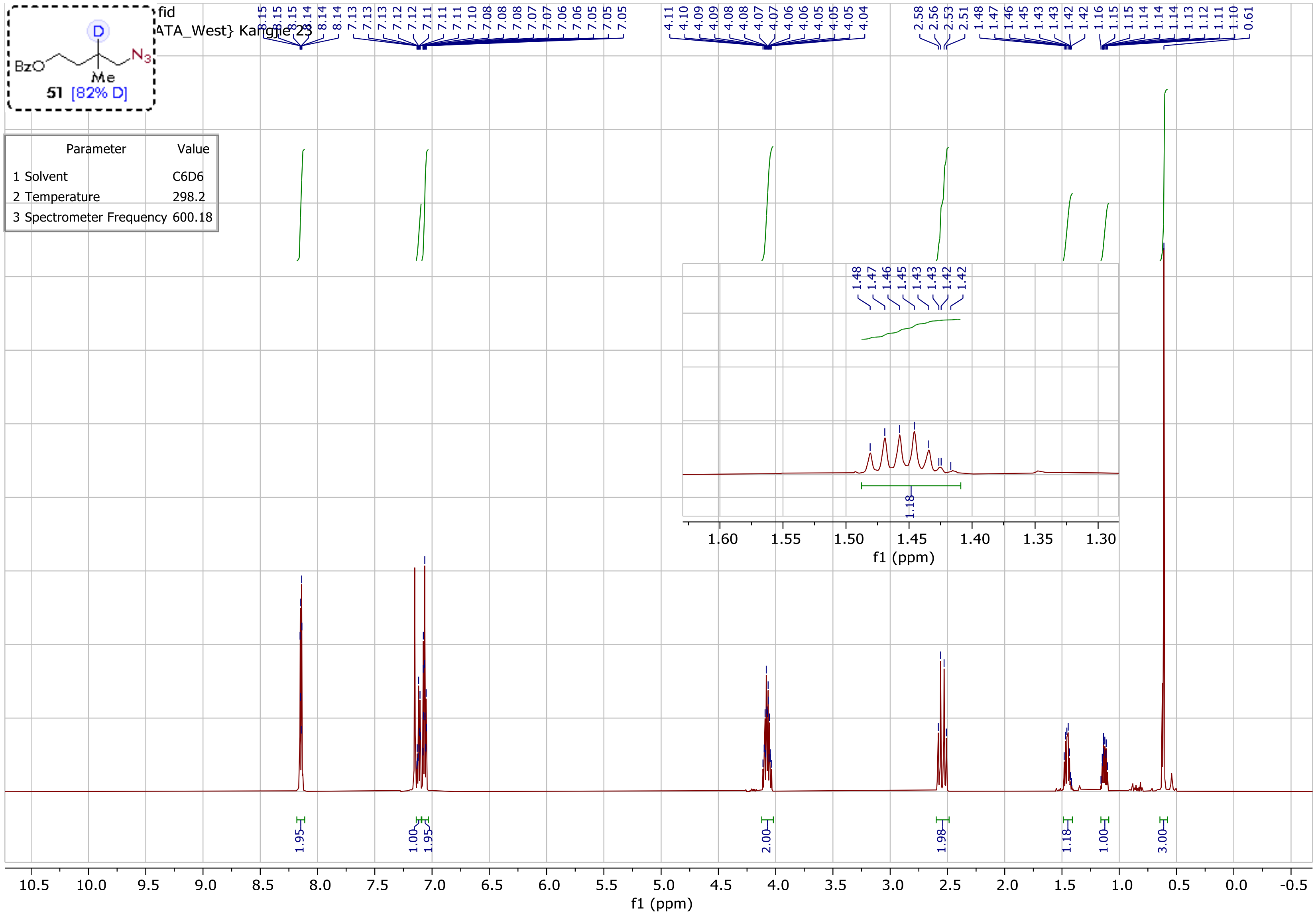

Parameter	Value
1 Solvent	C6D6
2 Temperature	298.2
3 Spectrometer Frequency	600.18

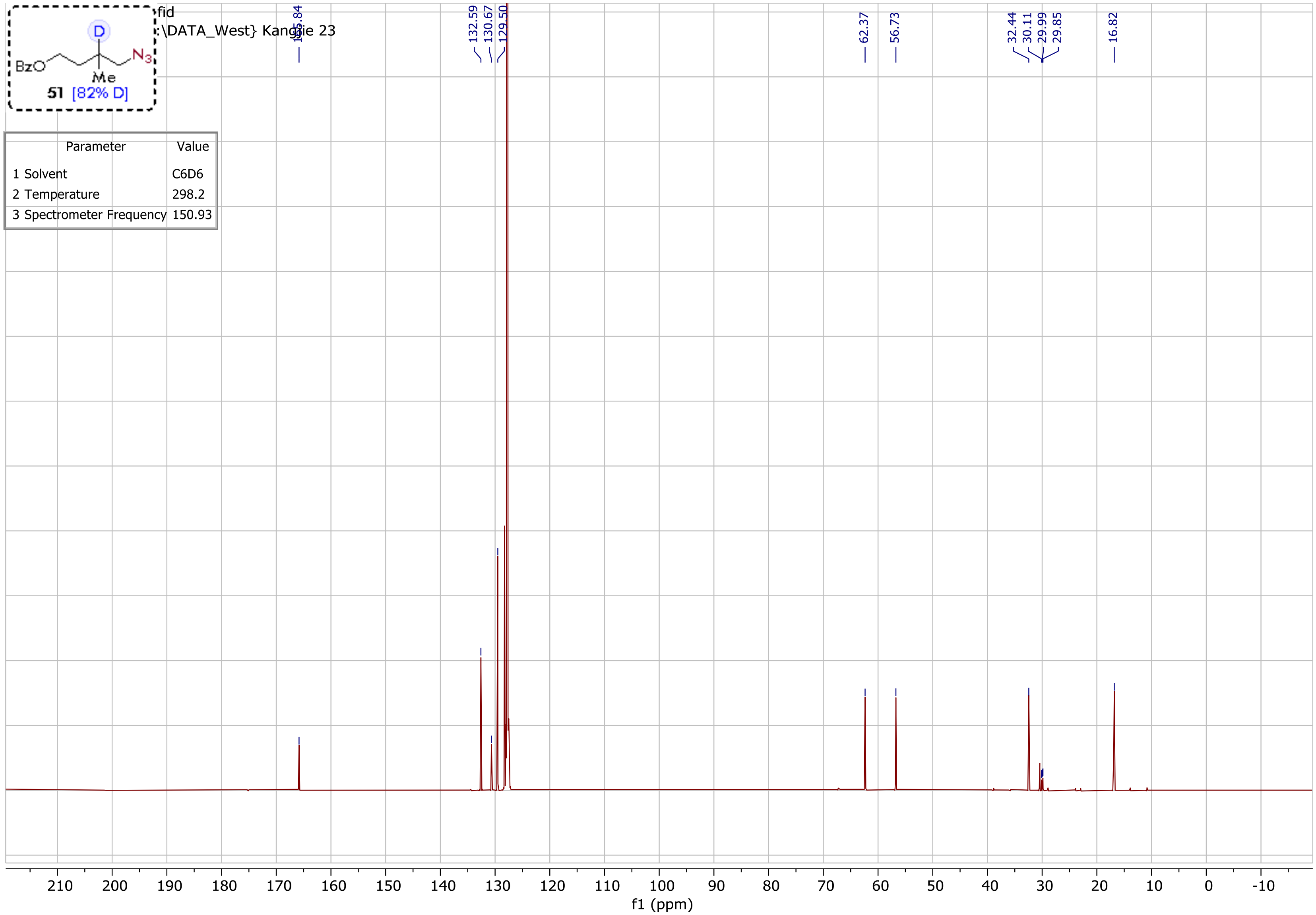
fid
:\DATA_West} Kangjie 23
BzO
D
N3
Me
51 [82% D]
Parameter Value
1 Solvent C6D6
2 Temperature 298.2
3 Spectrometer Frequency 150.93
132.59
130.67
129.50
62.37
56.73
32.44
30.11
29.99
29.85
16.82
210 200 190 180 170 160 150 140 130 120 110 100 90 80 70 60 50 40 30 20 10 0 -10
f1 (ppm)

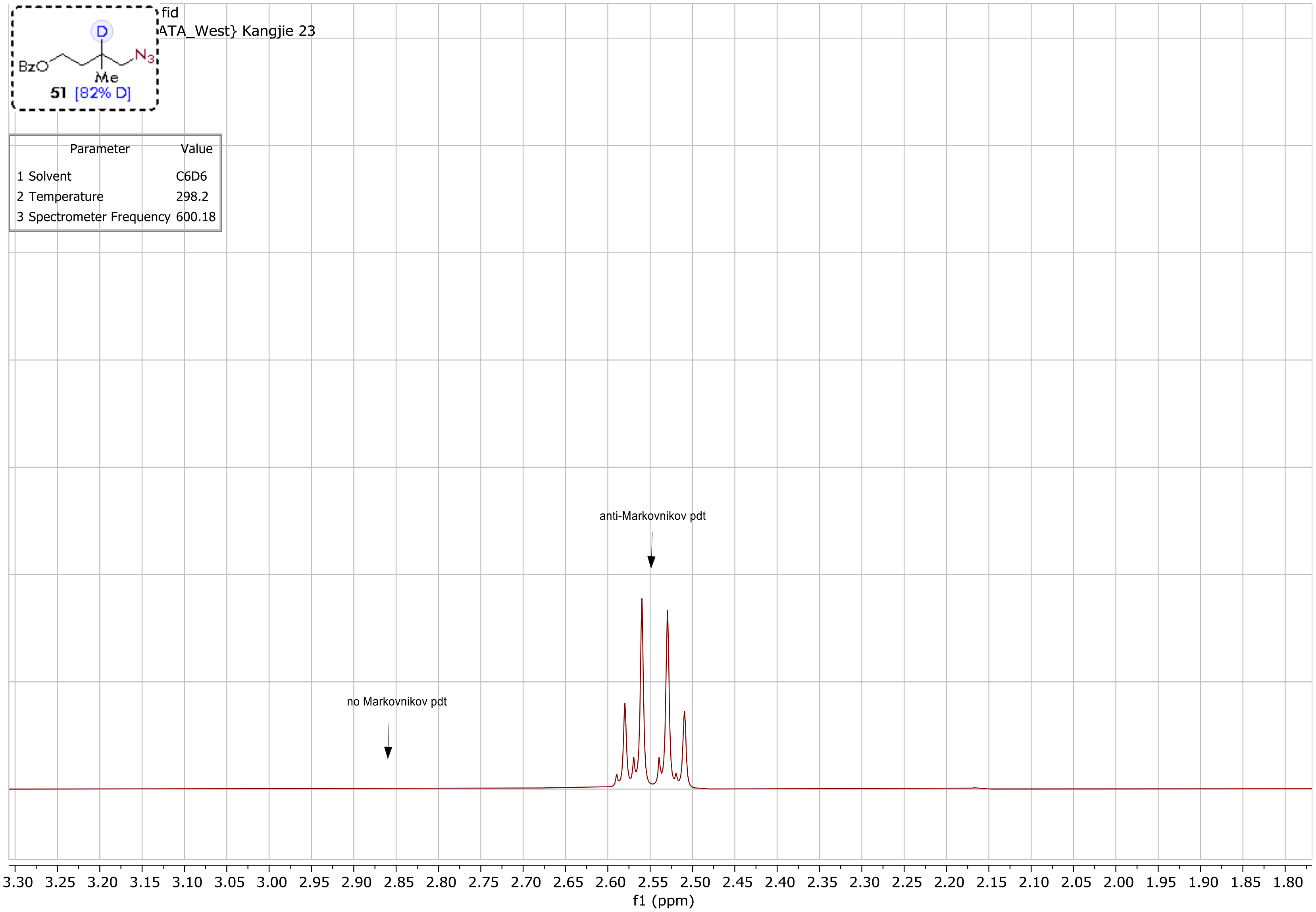
fid
ATA_West} Kangjie 23
D
BzO
N3
Me
51 [82% D]
Parameter Value
1 Solvent C6D6
2 Temperature 298.2
3 Spectrometer Frequency 600.18
anti-Markovnikov pdt
no Markovnikov pdt
3.30 3.25 3.20 3.15 3.10 3.05 3.00 2.95 2.90 2.85 2.80 2.75 2.70 2.65 2.60 2.55 2.50 2.45 2.40 2.35 2.30 2.25 2.20 2.15 2.10 2.05 2.00 1.95 1.90 1.85 1.80
f1 (ppm)

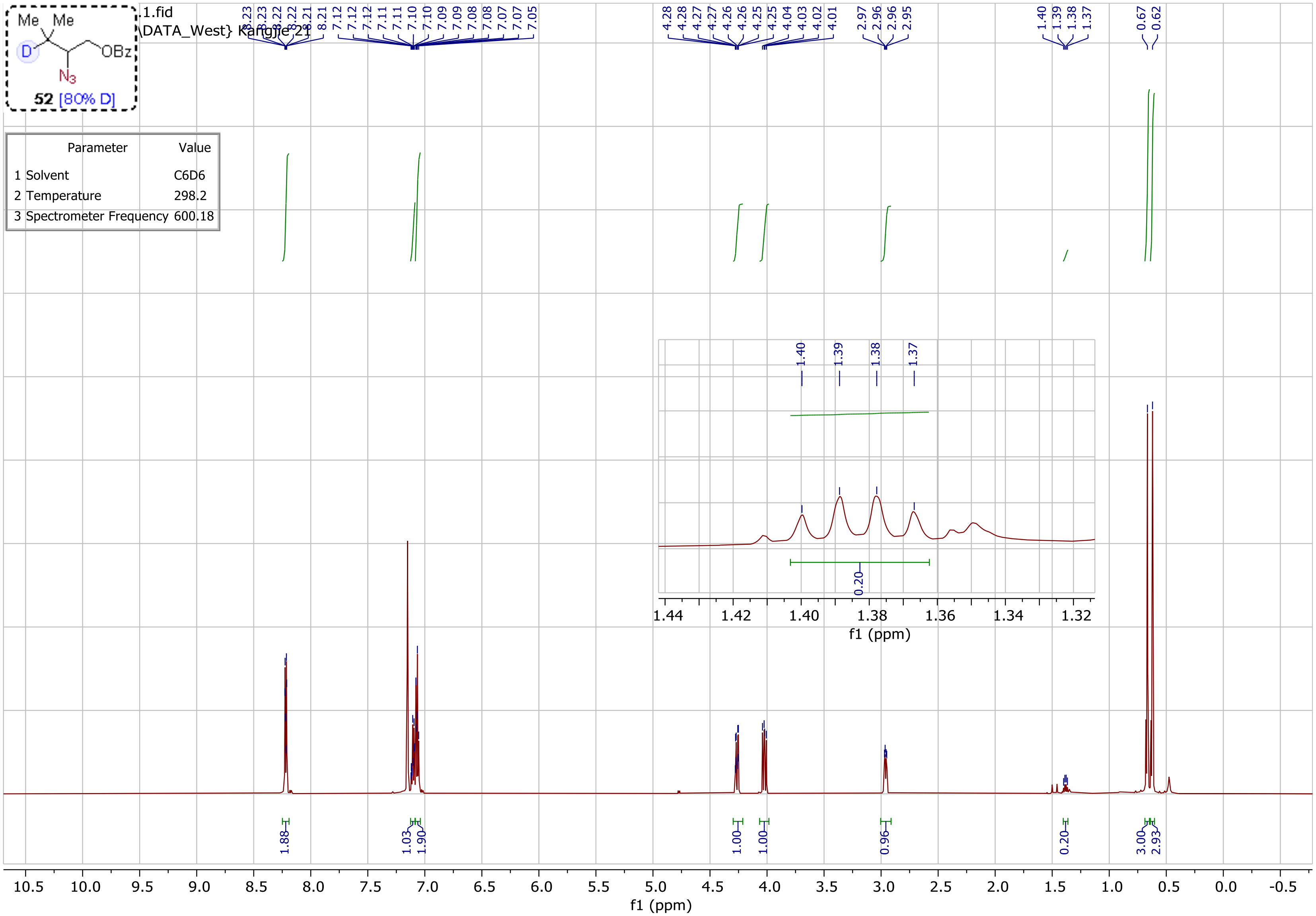
52 [80% D]
1.fid
\DATA_West} Kangjie 21
Parameter Value
1 Solvent C6D6
2 Temperature 298.2
3 Spectrometer Frequency 600.18
8.23 8.23 8.22 8.22 8.21 8.21
7.12 7.12 7.12 7.11 7.11 7.10 7.10 7.09 7.09 7.08 7.08 7.07 7.07 7.05
4.28 4.28 4.27 4.27 4.26 4.26 4.25 4.25 4.04 4.03 4.02 4.01
2.97 2.96 2.96 2.95
1.40 1.39 1.38 1.37
0.67 0.62
1.88 1.03 1.90 1.00 1.00 0.96 0.20 3.00 2.93
f1 (ppm)

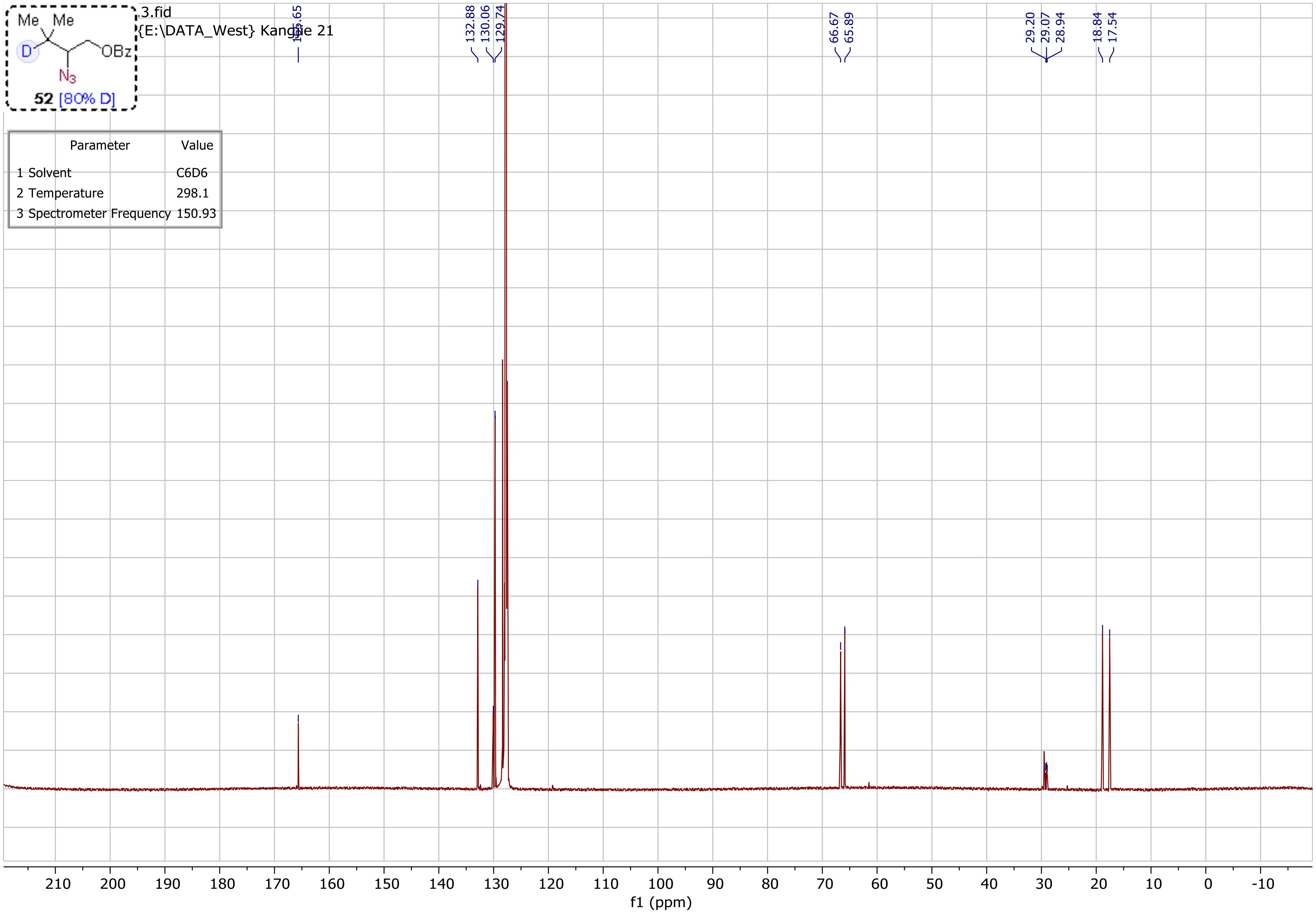

	Parameter	Value
1	Solvent	C6D6
2	Temperature	298.1
3	Spectrometer Frequency	150.93

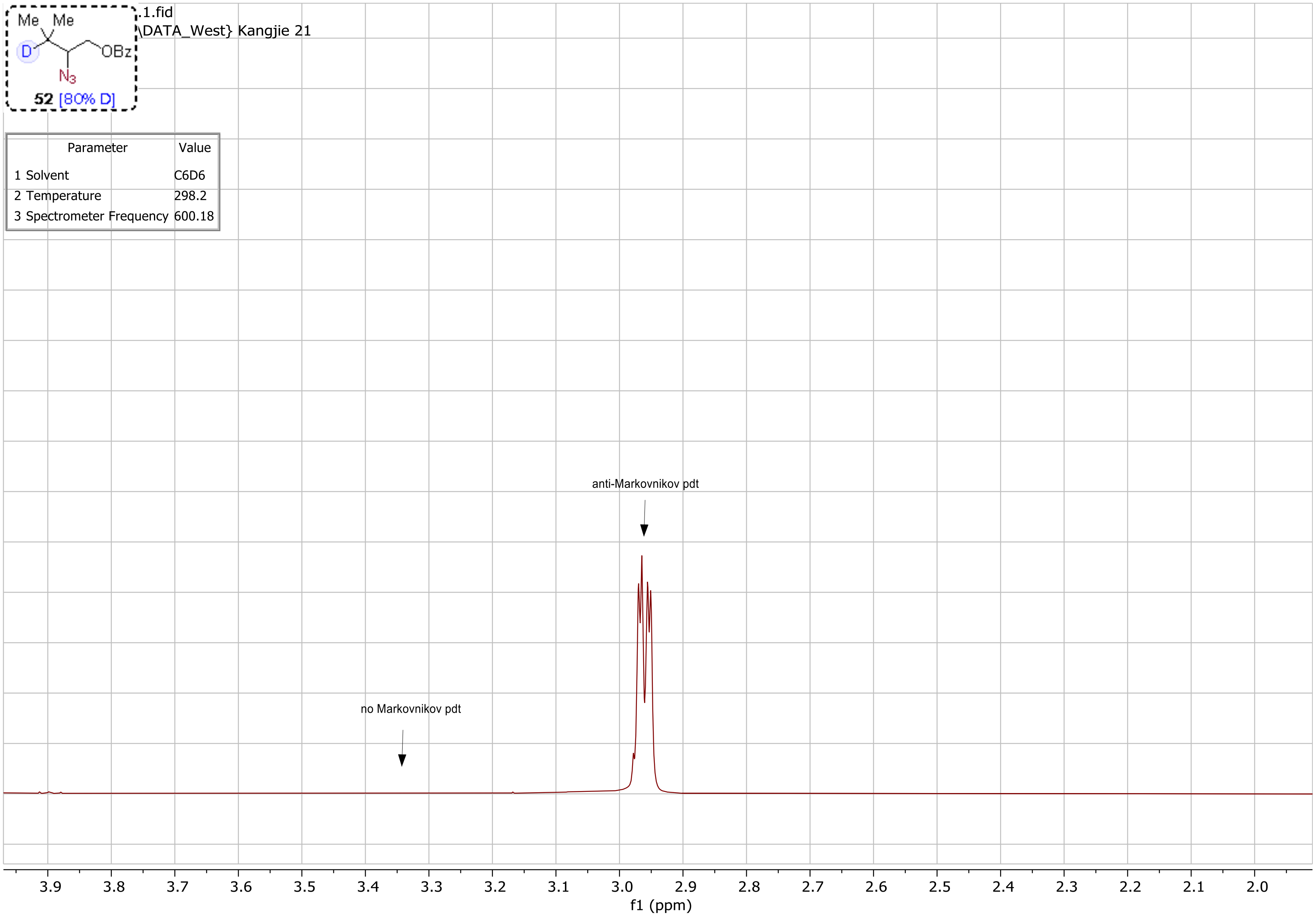

	Parameter	Value
1	Solvent	C6D6
2	Temperature	298.2
3	Spectrometer Frequency	600.18

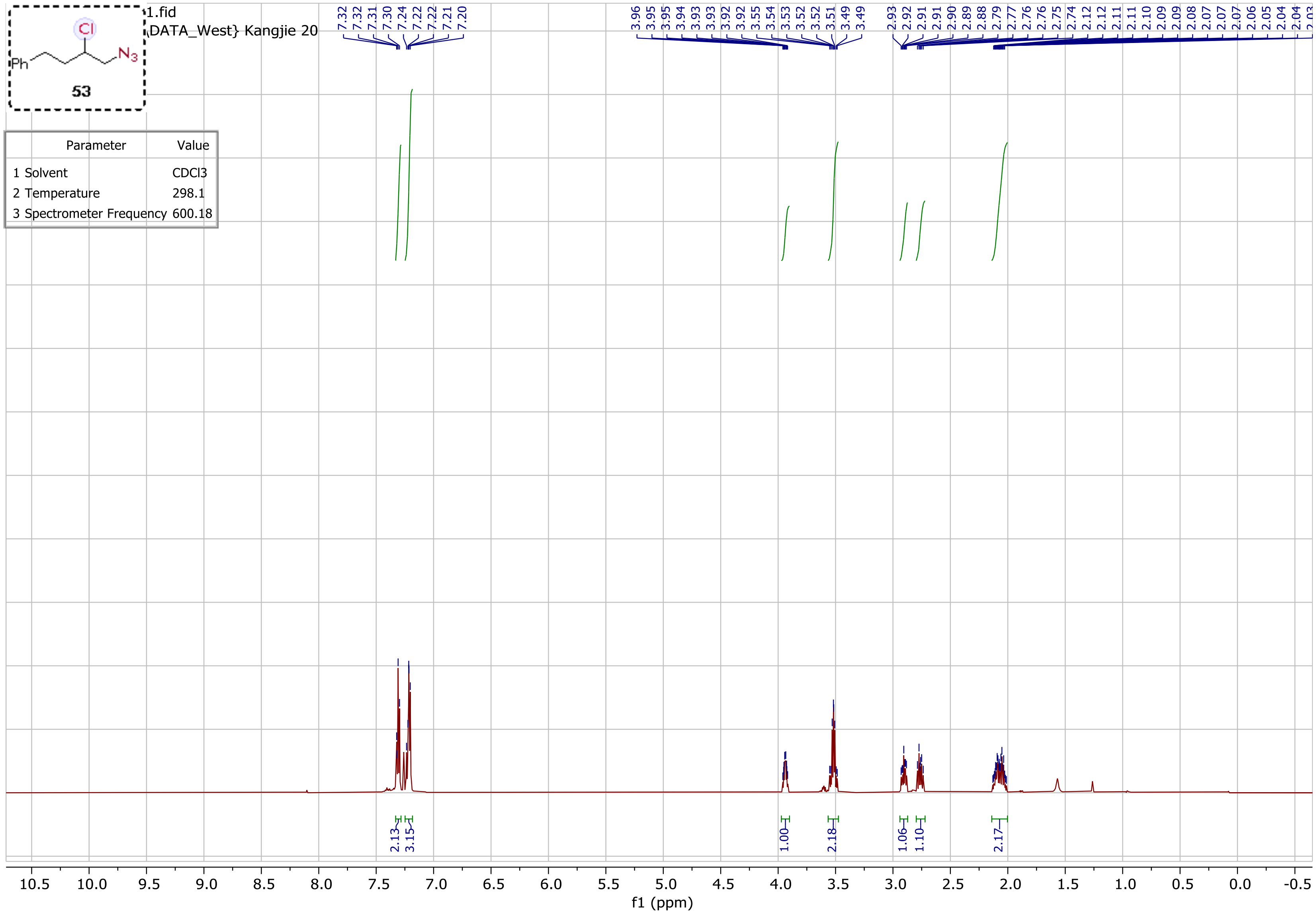

	Parameter	Value
1	Solvent	CDCl3
2	Temperature	298.1
3	Spectrometer Frequency	600.18

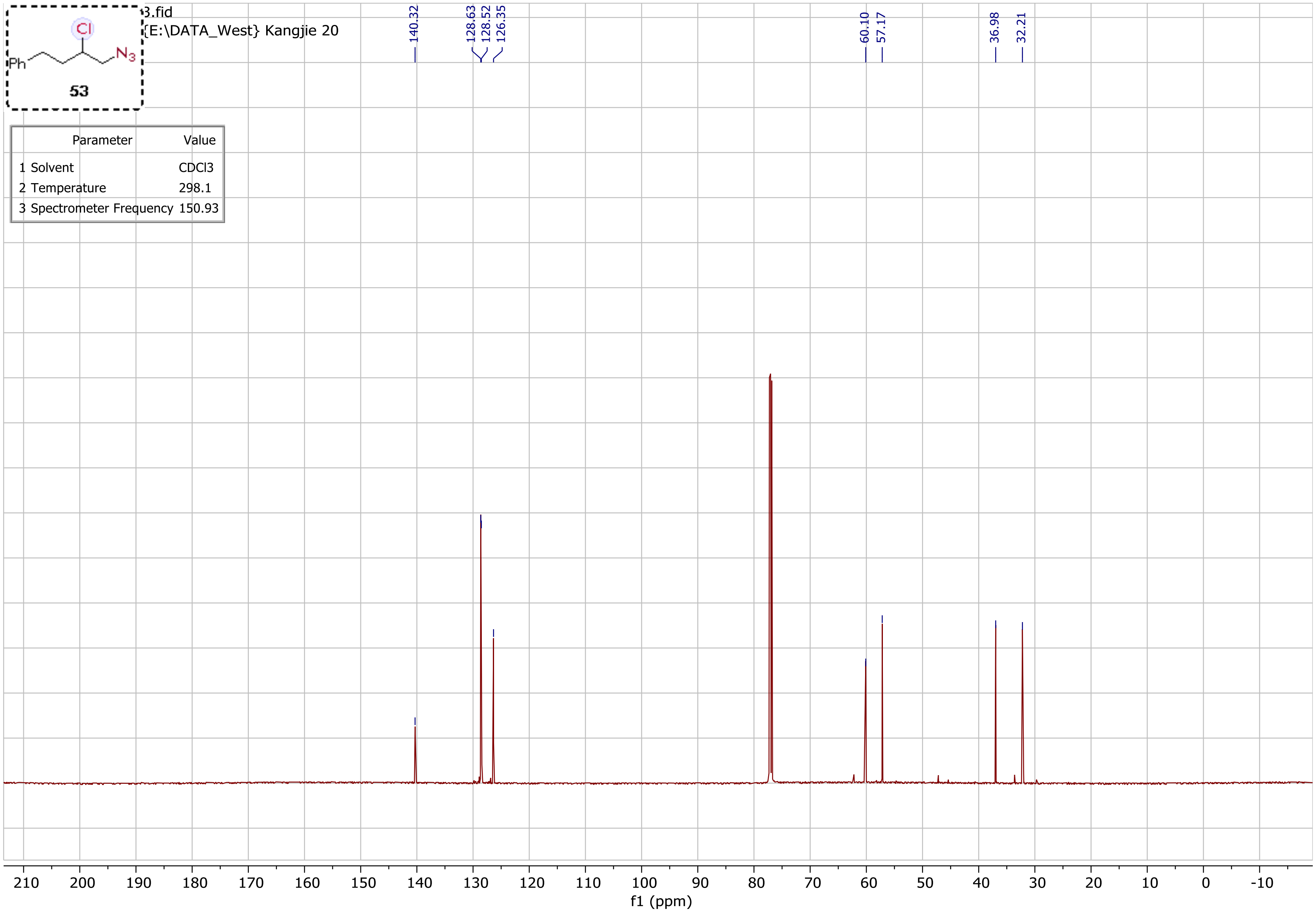

3.fid
{E:\DATA_West} Kangjie 20
Cl
Ph
N3
53
140.32
128.63
128.52
126.35
60.10
57.17
36.98
32.21
Parameter Value
1 Solvent CDCl3
2 Temperature 298.1
3 Spectrometer Frequency 150.93
210 200 190 180 170 160 150 140 130 120 110 100 90 80 70 60 50 40 30 20 10 0 -10
f1 (ppm)

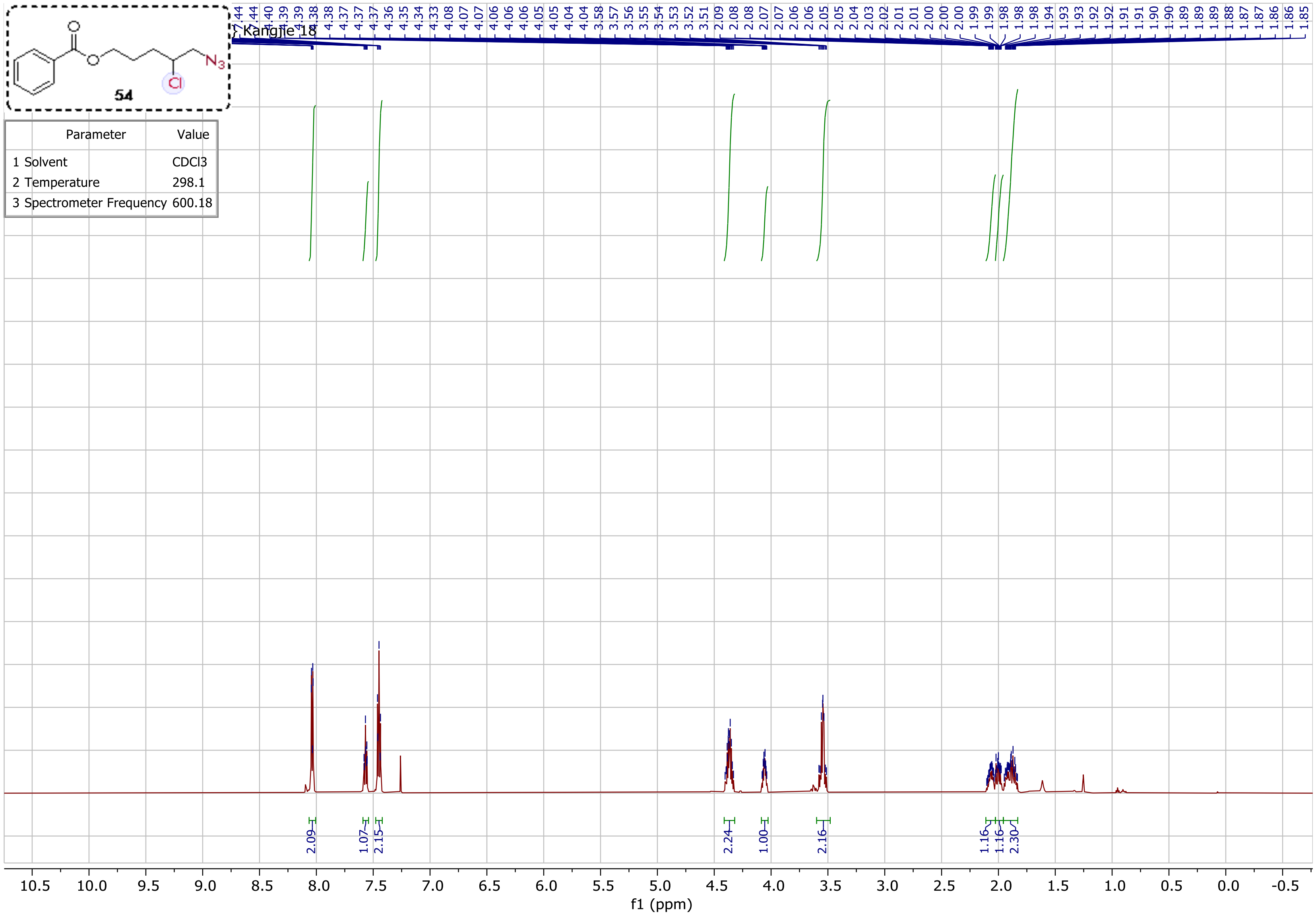

Kangjie 18
N3
Cl
54
Parameter | Value
1 Solvent | CDCl3
2 Temperature | 298.1
3 Spectrometer Frequency | 600.18
2.09
1.07
2.15
2.24
1.00
2.16
1.16
1.16
2.30
10.5 10.0 9.5 9.0 8.5 8.0 7.5 7.0 6.5 6.0 5.5 5.0 4.5 4.0 3.5 3.0 2.5 2.0 1.5 1.0 0.5 0.0 -0.5
f1 (ppm)

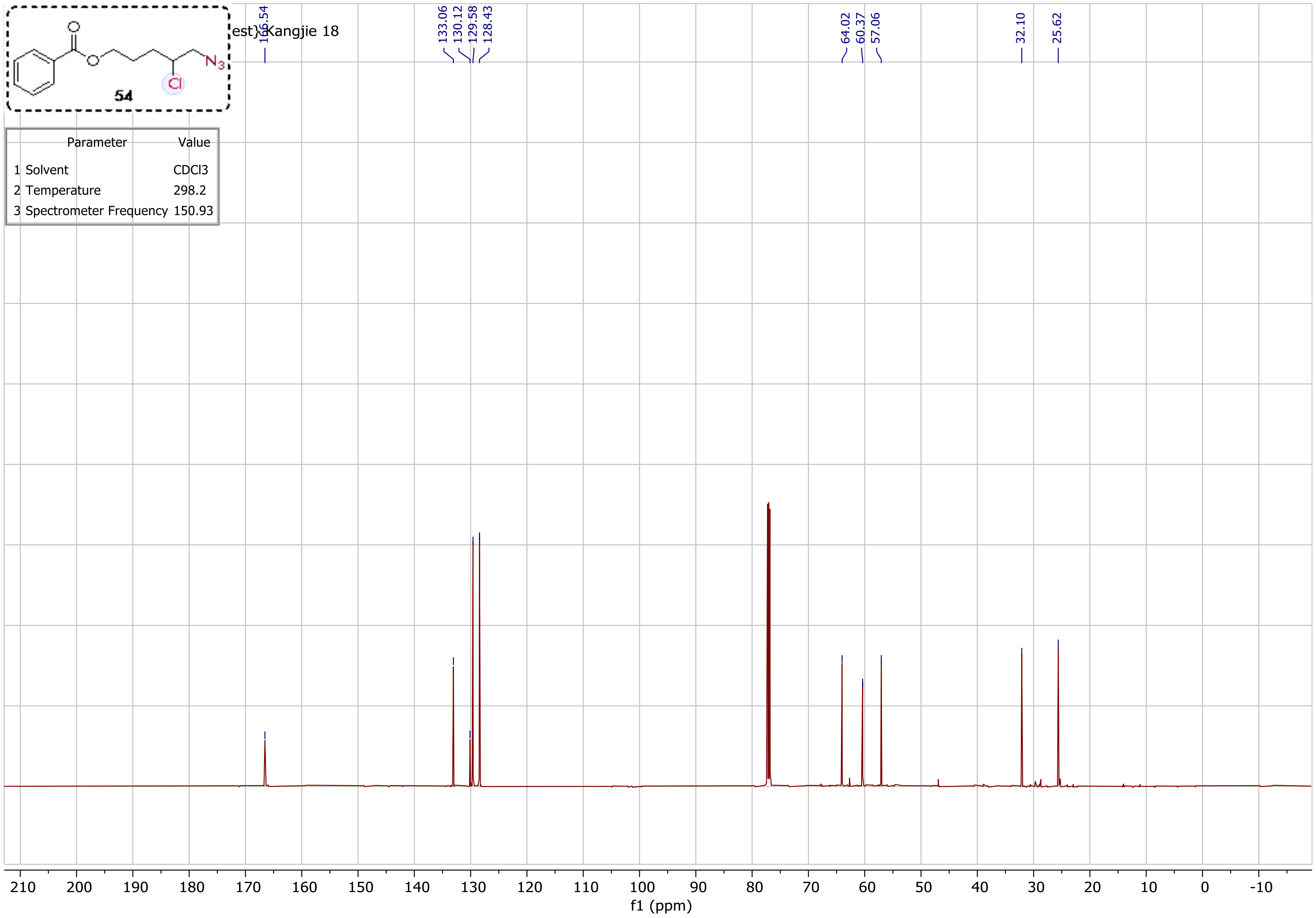

54
N3
Cl
est} Kangjie 18
Parameter Value
1 Solvent CDCl3
2 Temperature 298.2
3 Spectrometer Frequency 150.93
166.54
133.06
130.12
129.58
128.43
64.02
60.37
57.06
32.10
25.62
210 200 190 180 170 160 150 140 130 120 110 100 90 80 70 60 50 40 30 20 10 0 -10
f1 (ppm)

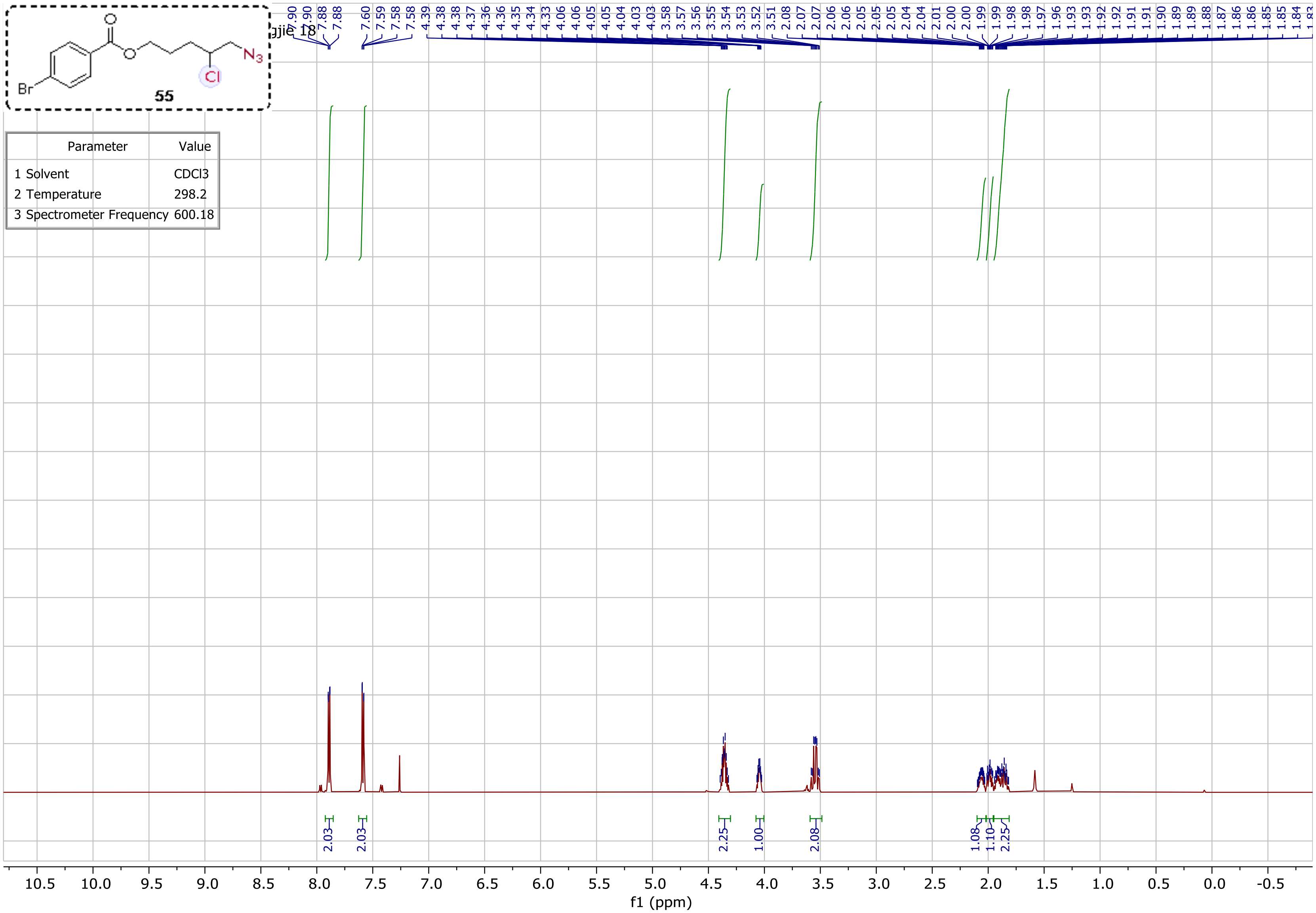

	Parameter	Value
1	Solvent	CDCl3
2	Temperature	298.2
3	Spectrometer Frequency	600.18

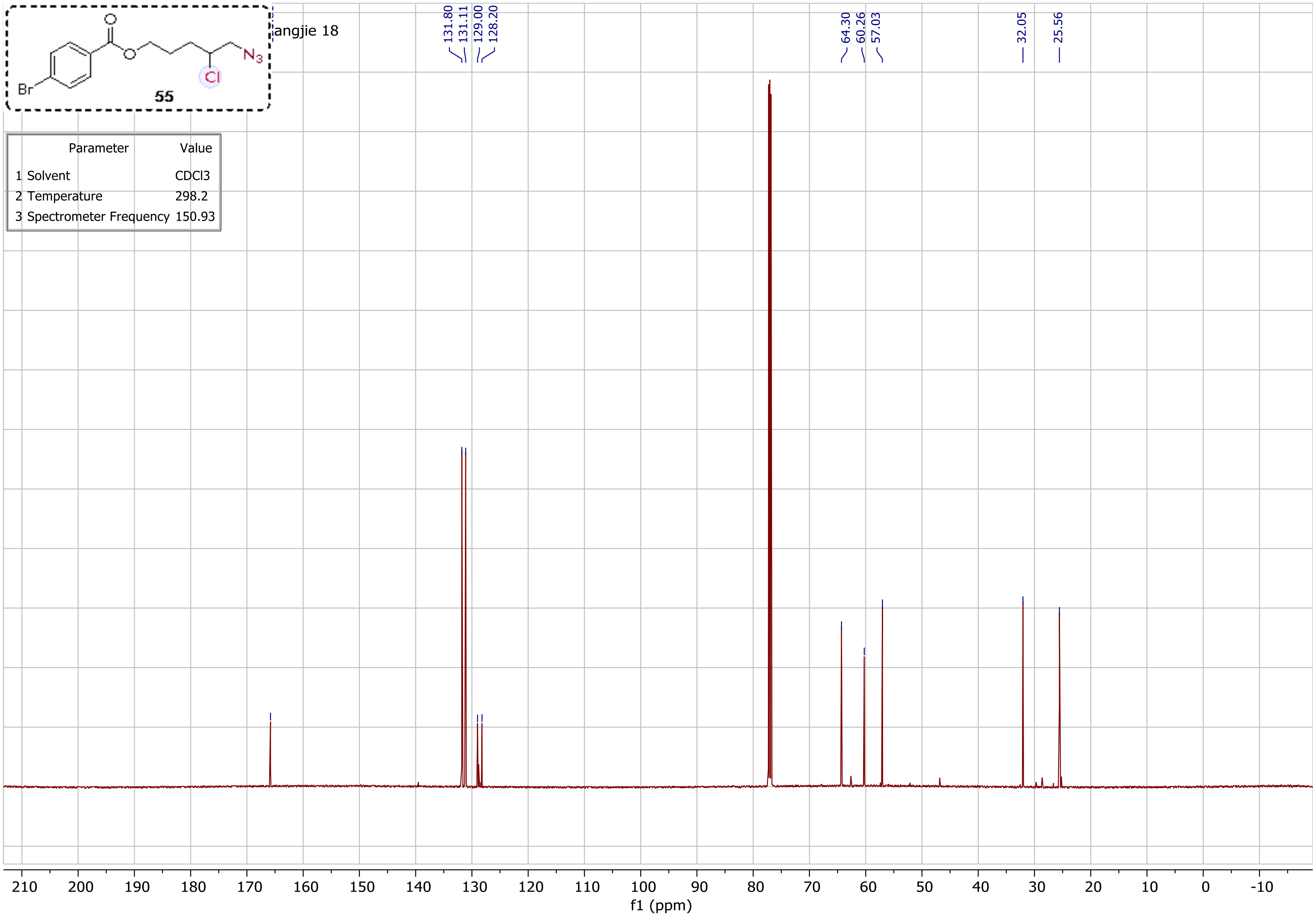

	Parameter	Value
1	Solvent	CDCl3
2	Temperature	298.2
3	Spectrometer Frequency	150.93

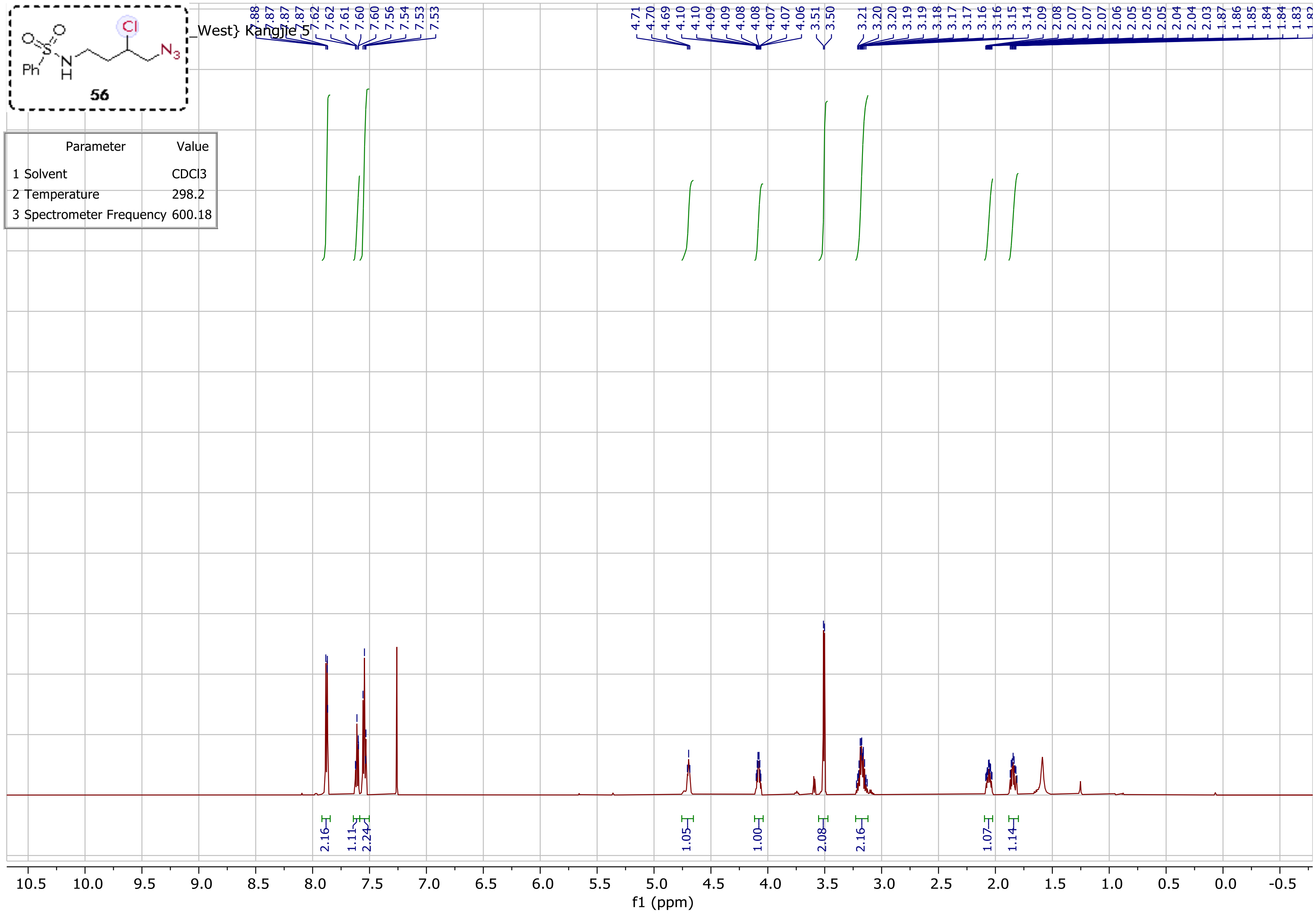

	Parameter	Value
1	Solvent	CDCl3
2	Temperature	298.2
3	Spectrometer Frequency	600.18

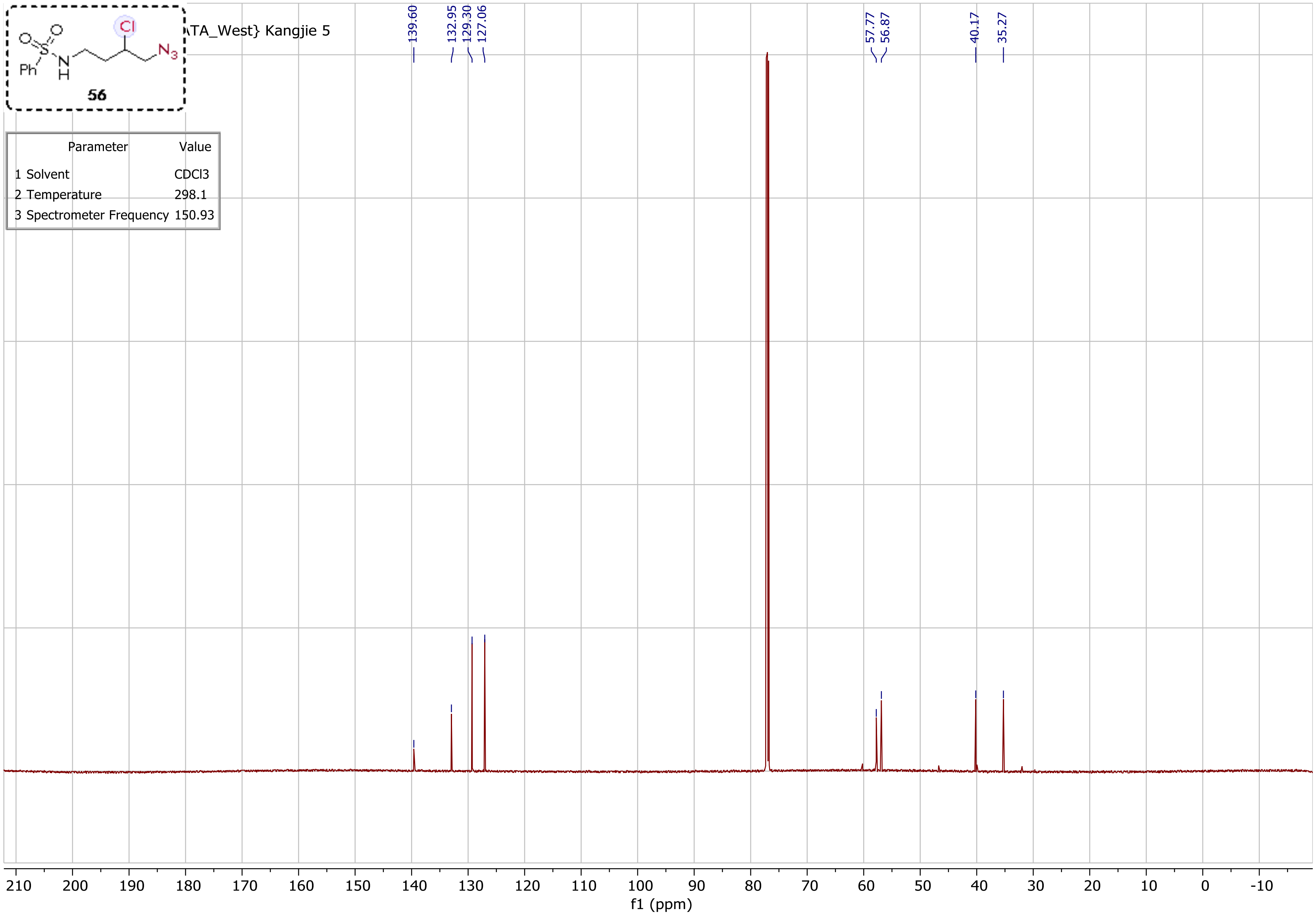

Parameter	Value
1 Solvent	CDCl3
2 Temperature	298.1
3 Spectrometer Frequency	150.93

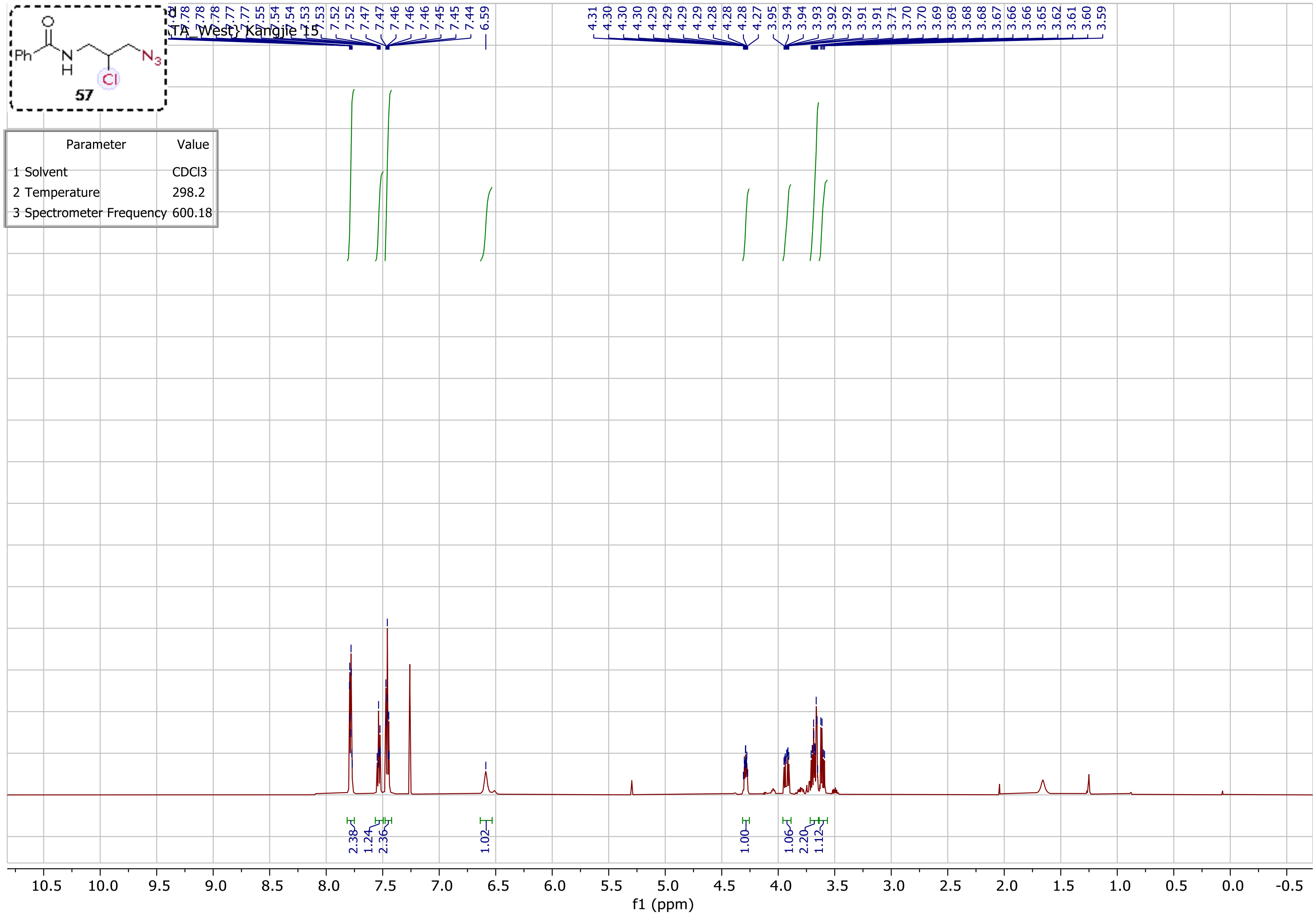

	Parameter	Value
1	Solvent	CDCl3
2	Temperature	298.2
3	Spectrometer Frequency	600.18

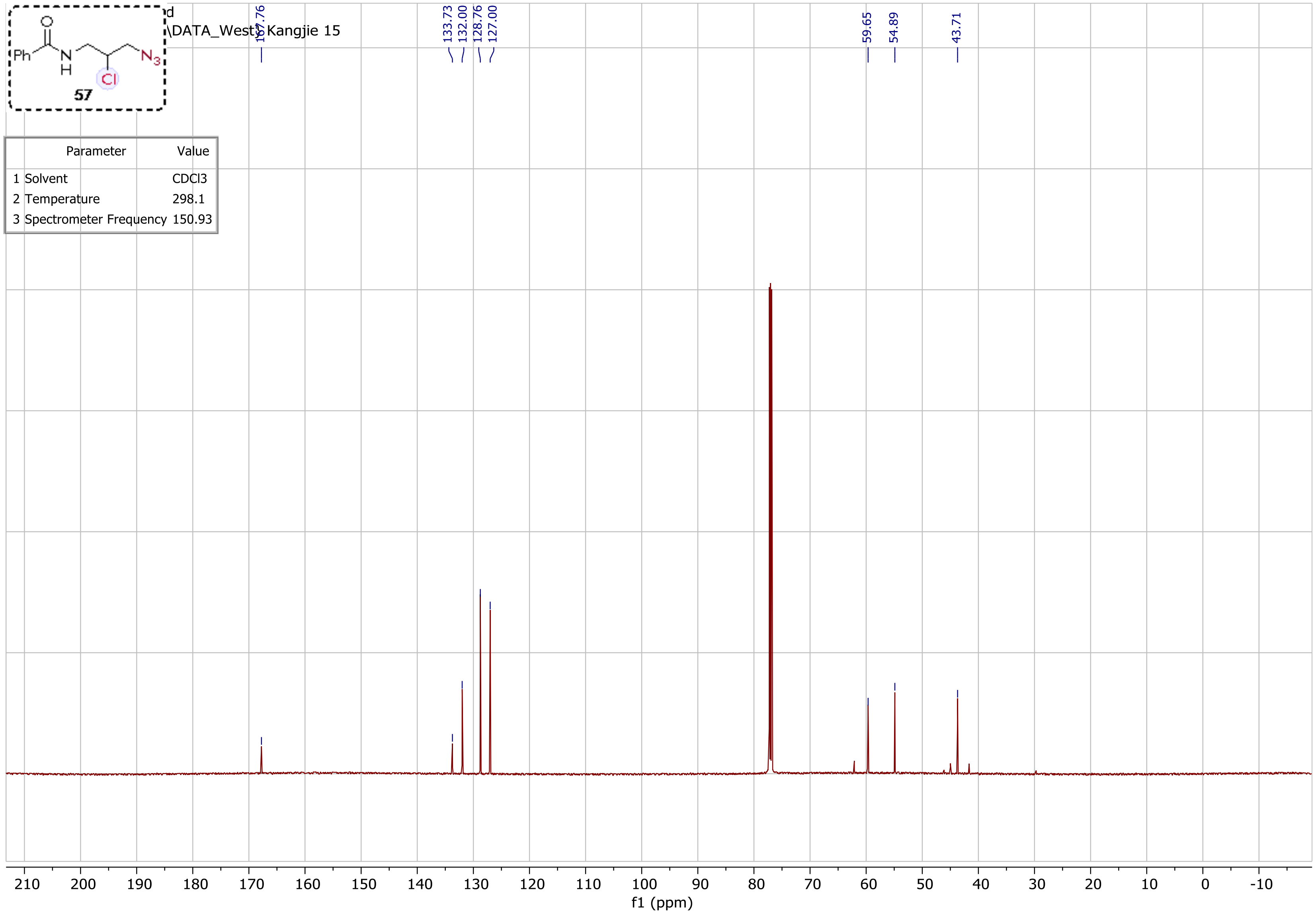

57
Ph
N
H
Cl
N3
\DATA_West\ Kangjie 15
133.73
132.00
128.76
127.00
59.65
54.89
43.71
Parameter Value
1 Solvent CDCl3
2 Temperature 298.1
3 Spectrometer Frequency 150.93
210 200 190 180 170 160 150 140 130 120 110 100 90 80 70 60 50 40 30 20 10 0 -10
f1 (ppm)

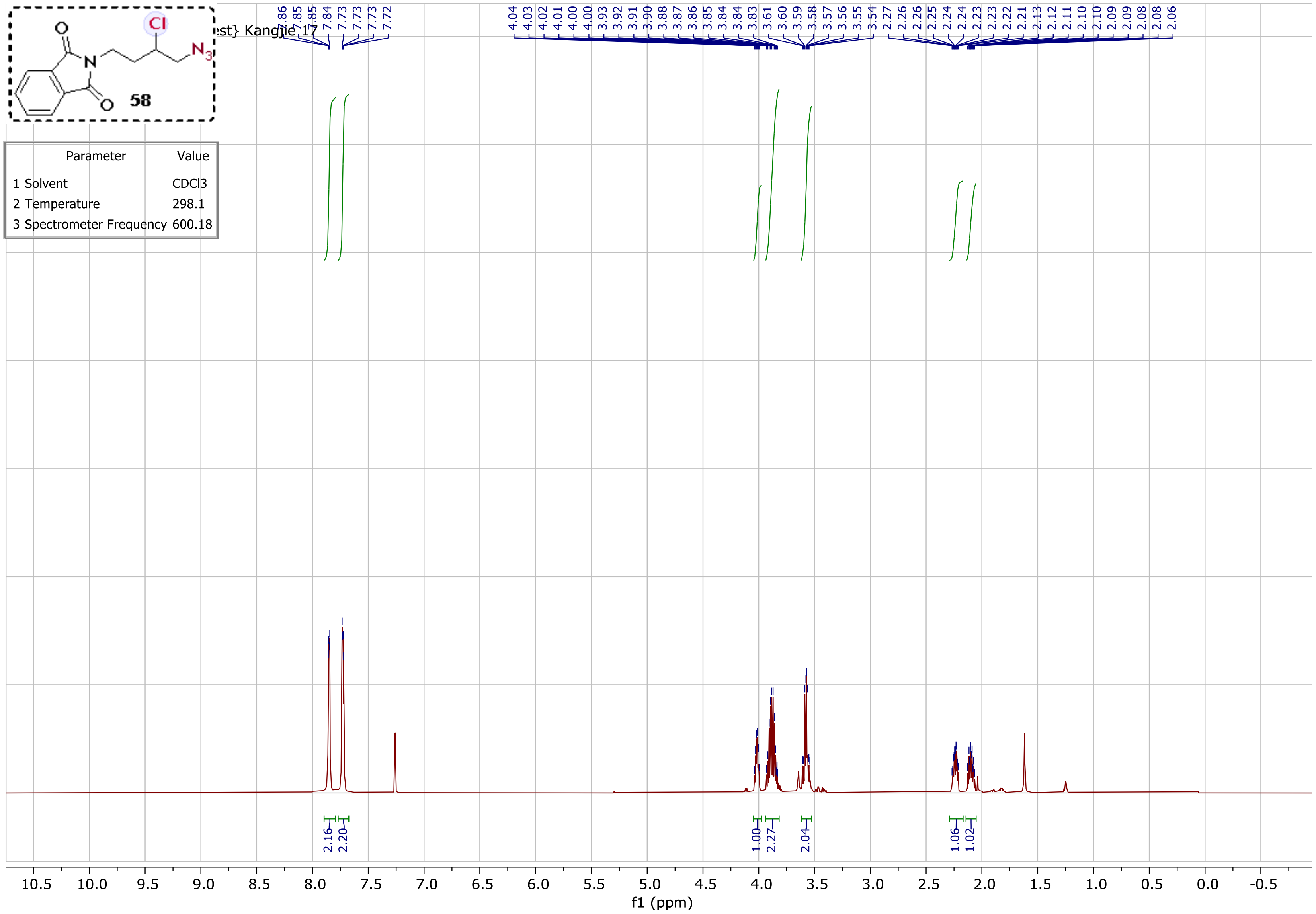

Parameter	Value
1 Solvent	CDCl3
2 Temperature	298.1
3 Spectrometer Frequency	600.18

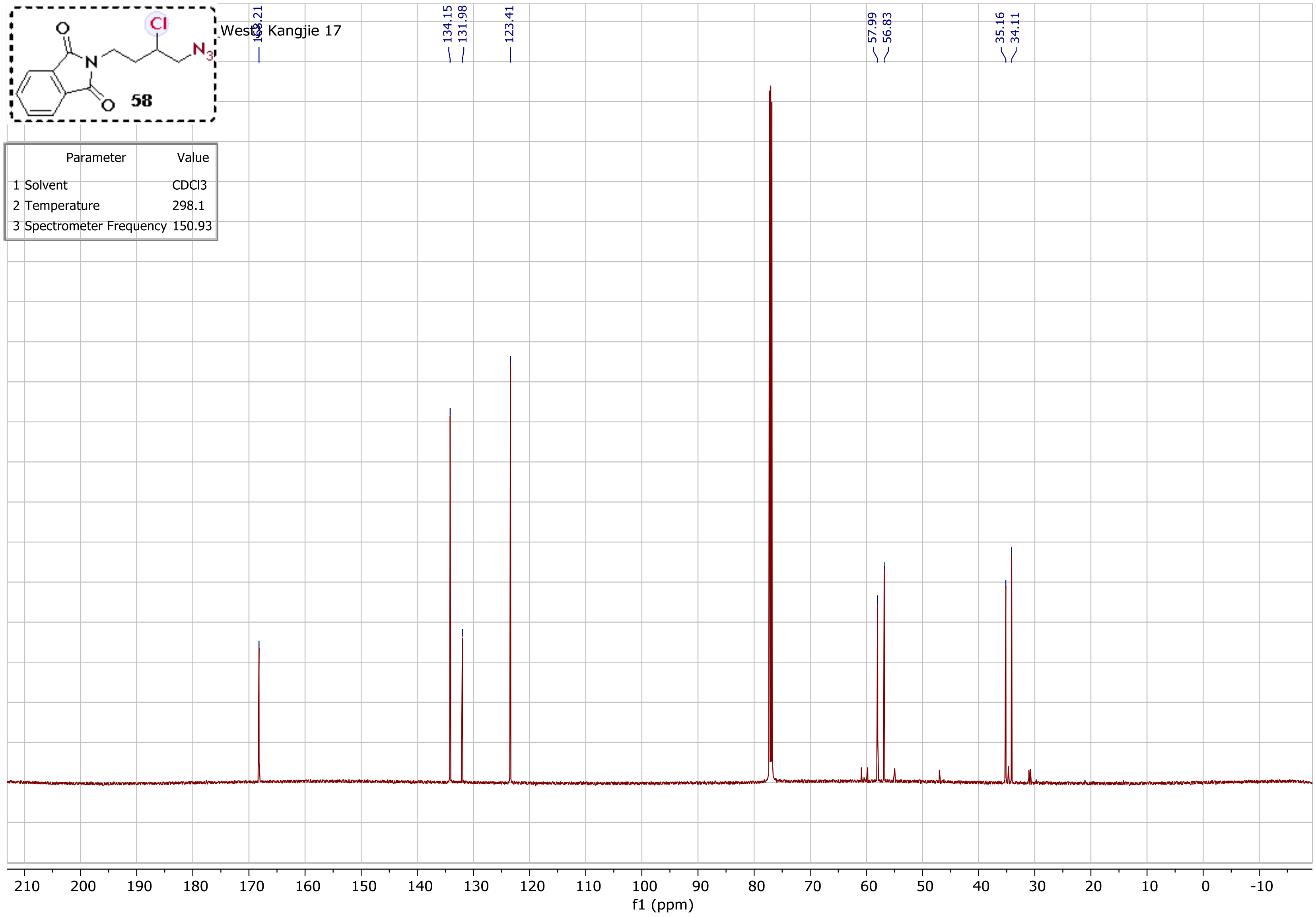

58
West Kangjie 17
168.21
134.15
131.98
123.41
57.99
56.83
35.16
34.11
Parameter Value
1 Solvent CDCl3
2 Temperature 298.1
3 Spectrometer Frequency 150.93
210 200 190 180 170 160 150 140 130 120 110 100 90 80 70 60 50 40 30 20 10 0 -10
f1 (ppm)

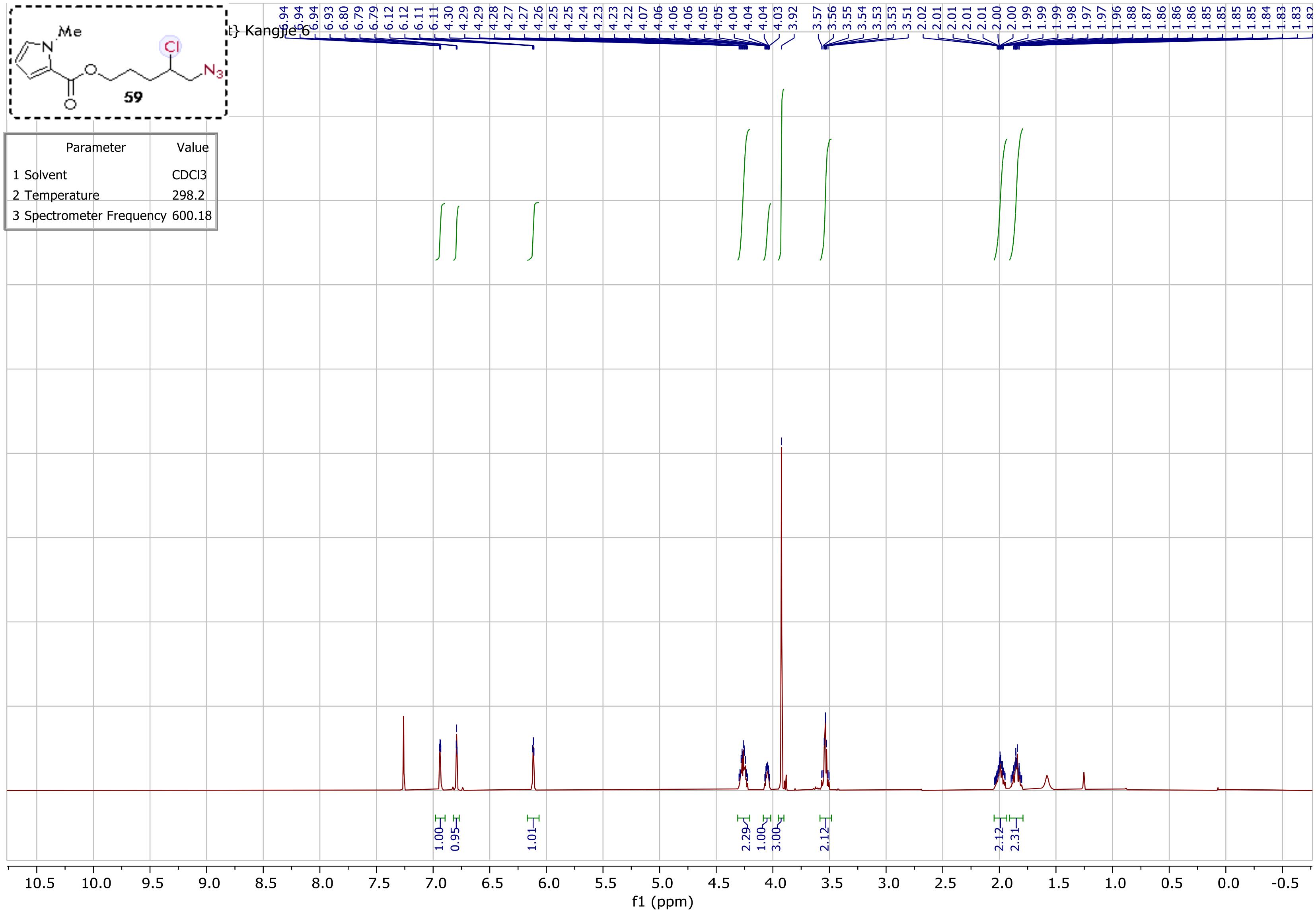

Parameter	Value
1 Solvent	CDCl3
2 Temperature	298.2
3 Spectrometer Frequency	600.18

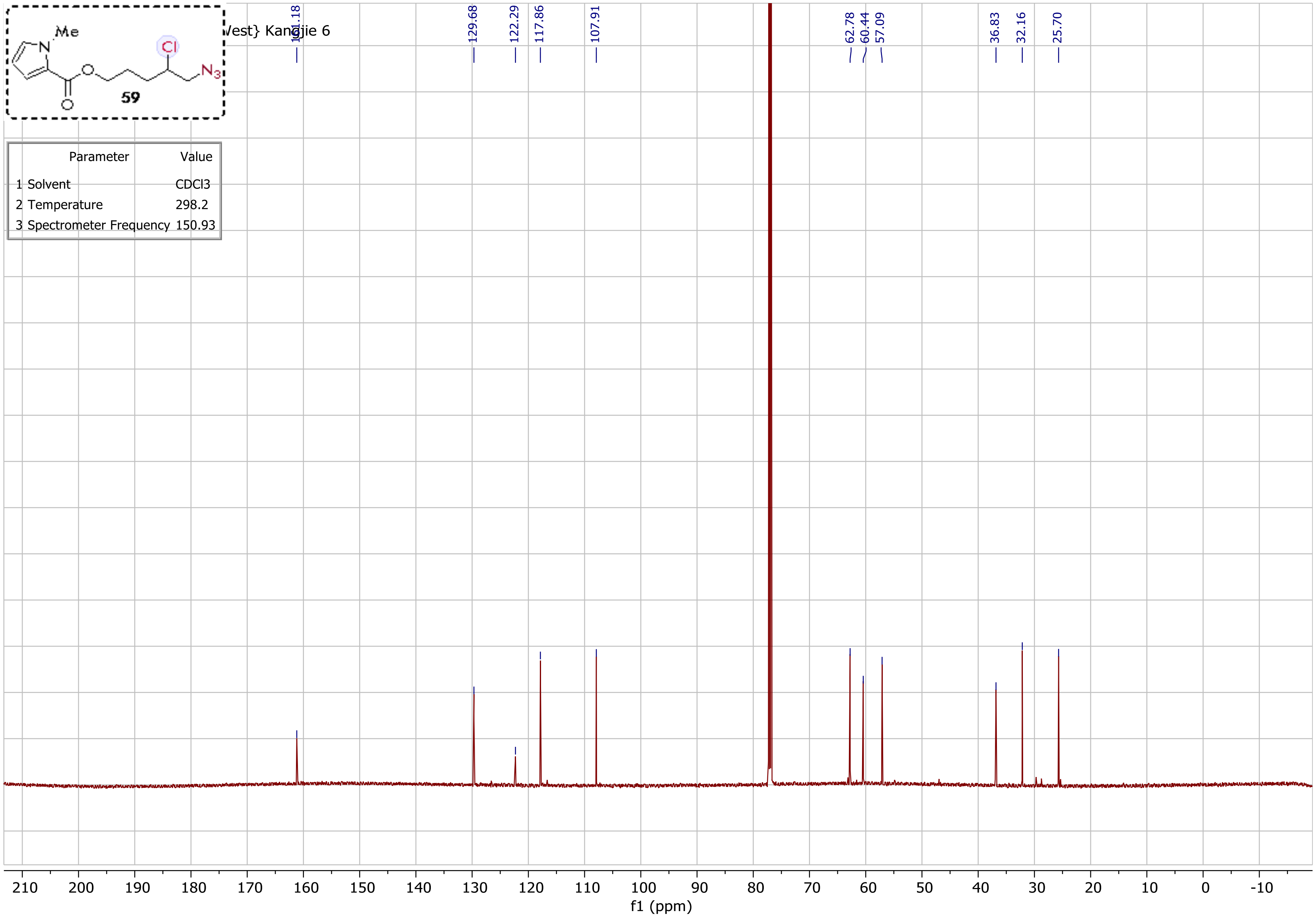
Me
N
O
O
Cl
N3
59
Kangjie 6
129.68
122.29
117.86
107.91
62.78
60.44
57.09
36.83
32.16
25.70
Parameter Value
1 Solvent CDCl3
2 Temperature 298.2
3 Spectrometer Frequency 150.93
210 200 190 180 170 160 150 140 130 120 110 100 90 80 70 60 50 40 30 20 10 0 -10
f1 (ppm)

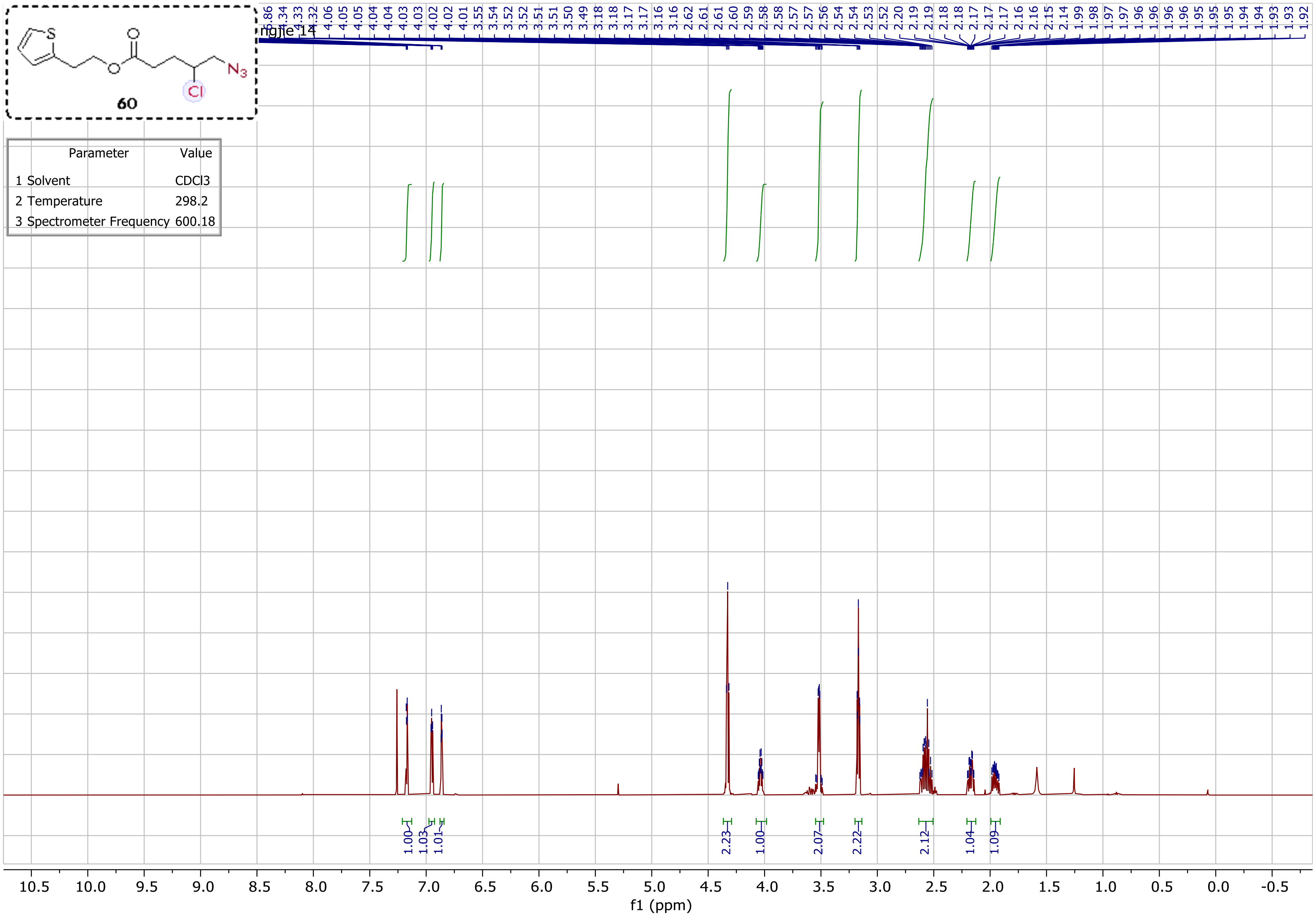
60
Parameter Value
1 Solvent CDCl3
2 Temperature 298.2
3 Spectrometer Frequency 600.18
1.00 1.03 1.01 2.23 1.00 2.07 2.22 2.12 1.04 1.09
f1 (ppm)

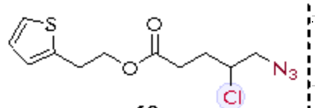

60

Kangjie 14

Parameter	Value
1 Solvent	CDCl3
2 Temperature	298.2
3 Spectrometer Frequency	150.93

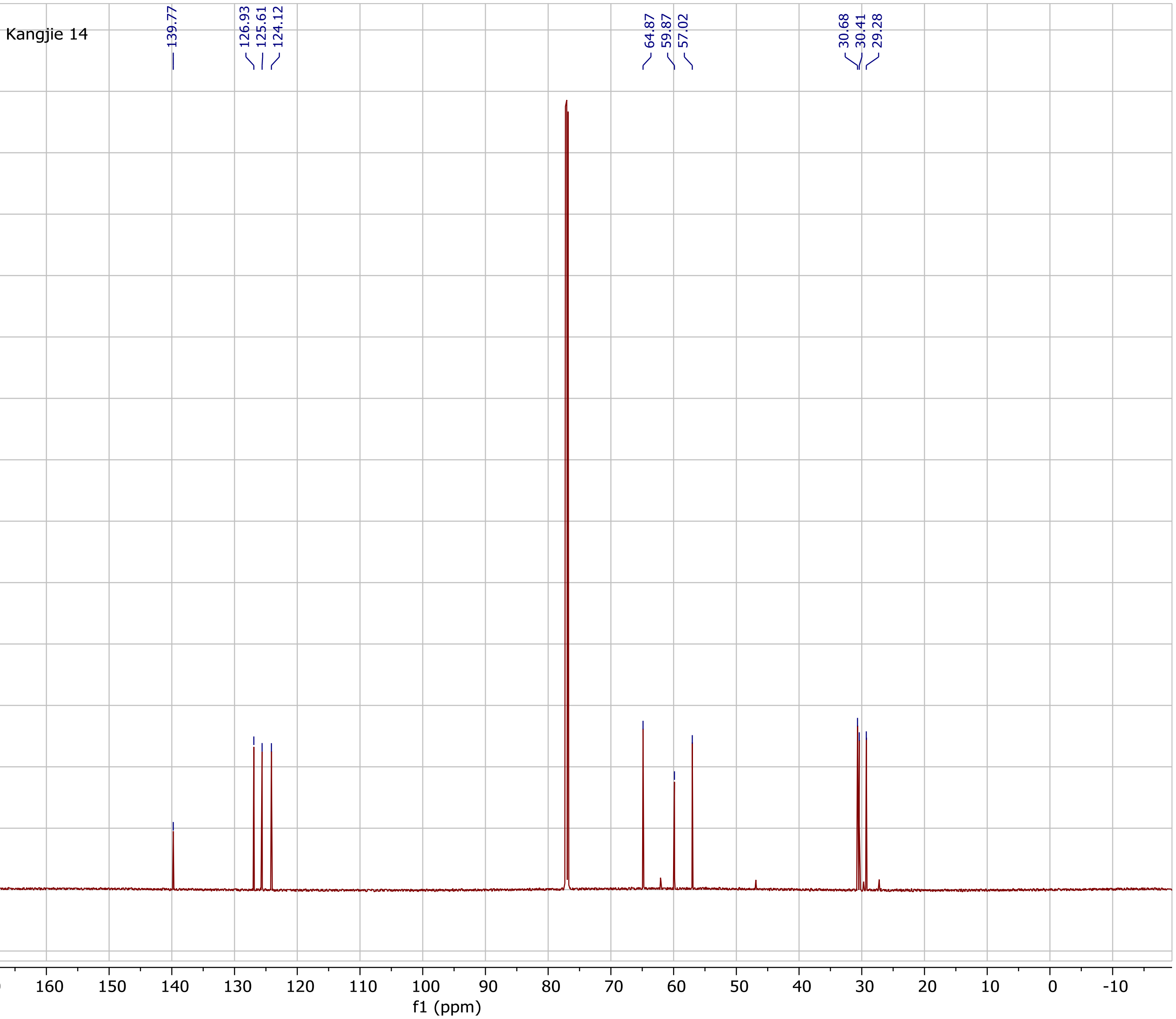

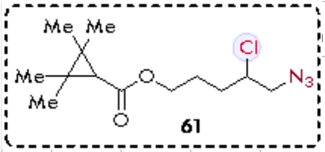

	Parameter	Value
1	Solvent	CDCl3
2	Temperature	298.1
3	Spectrometer Frequency	600.18

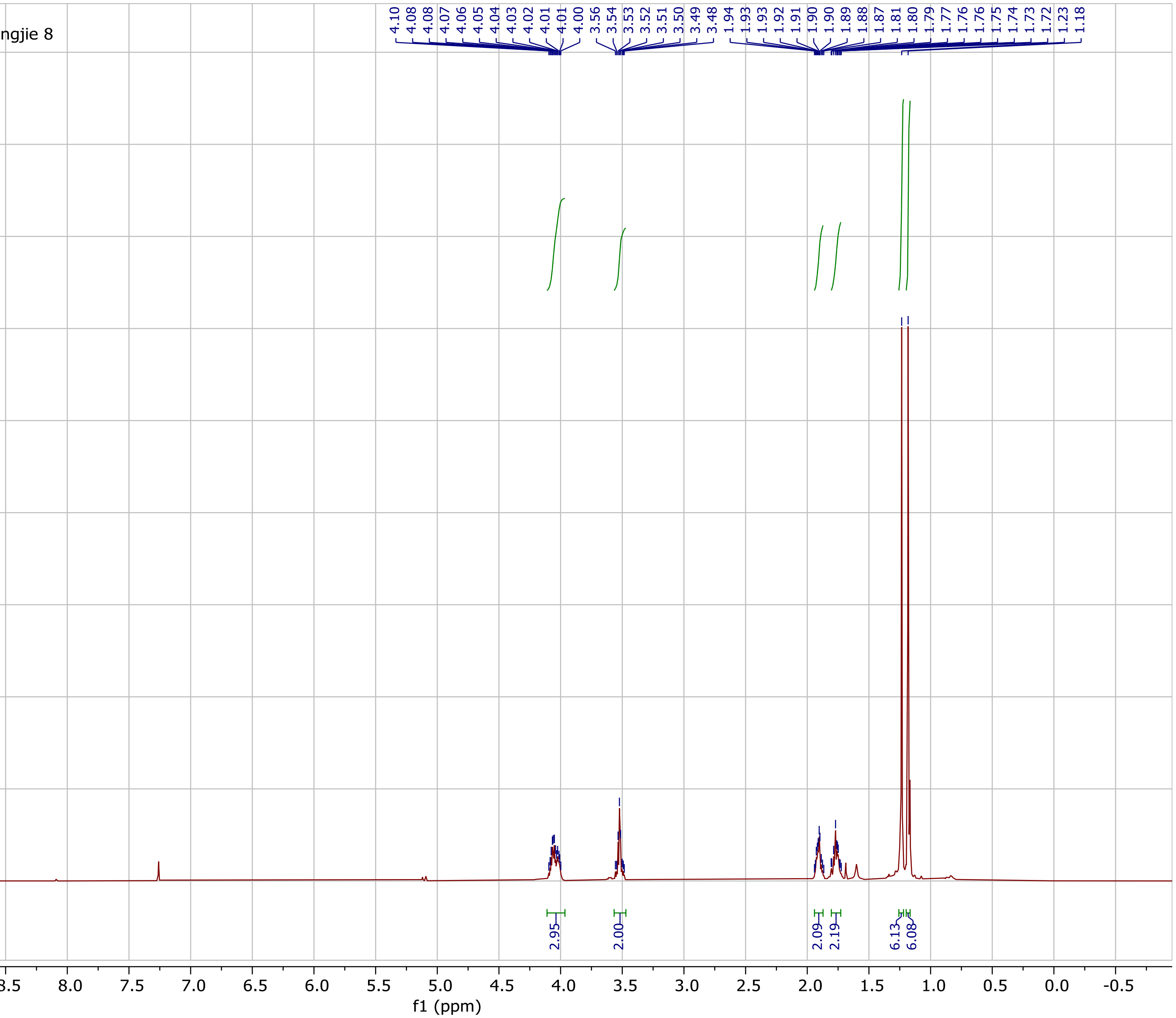

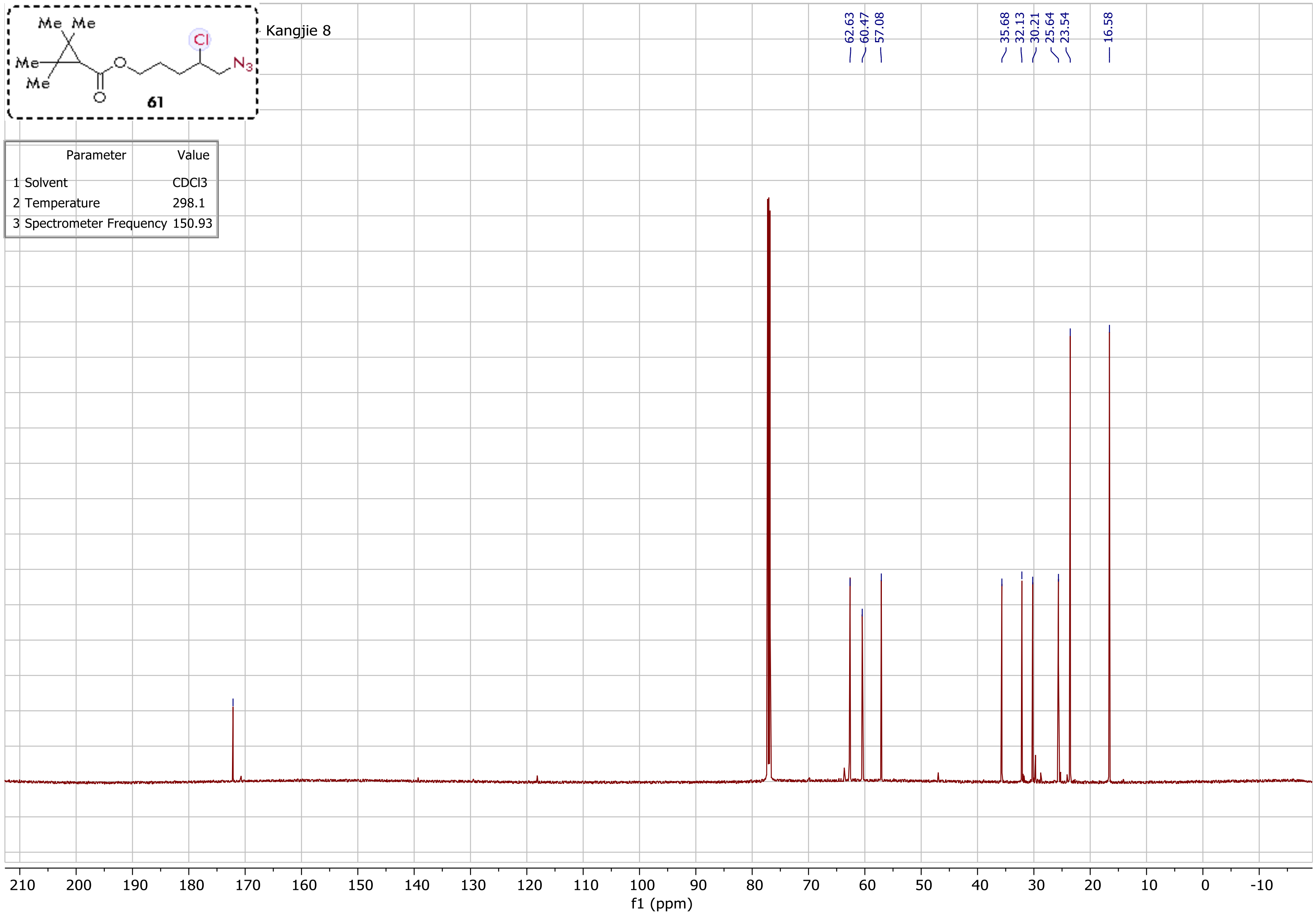

	Parameter	Value
1	Solvent	CDCl3
2	Temperature	298.1
3	Spectrometer Frequency	150.93

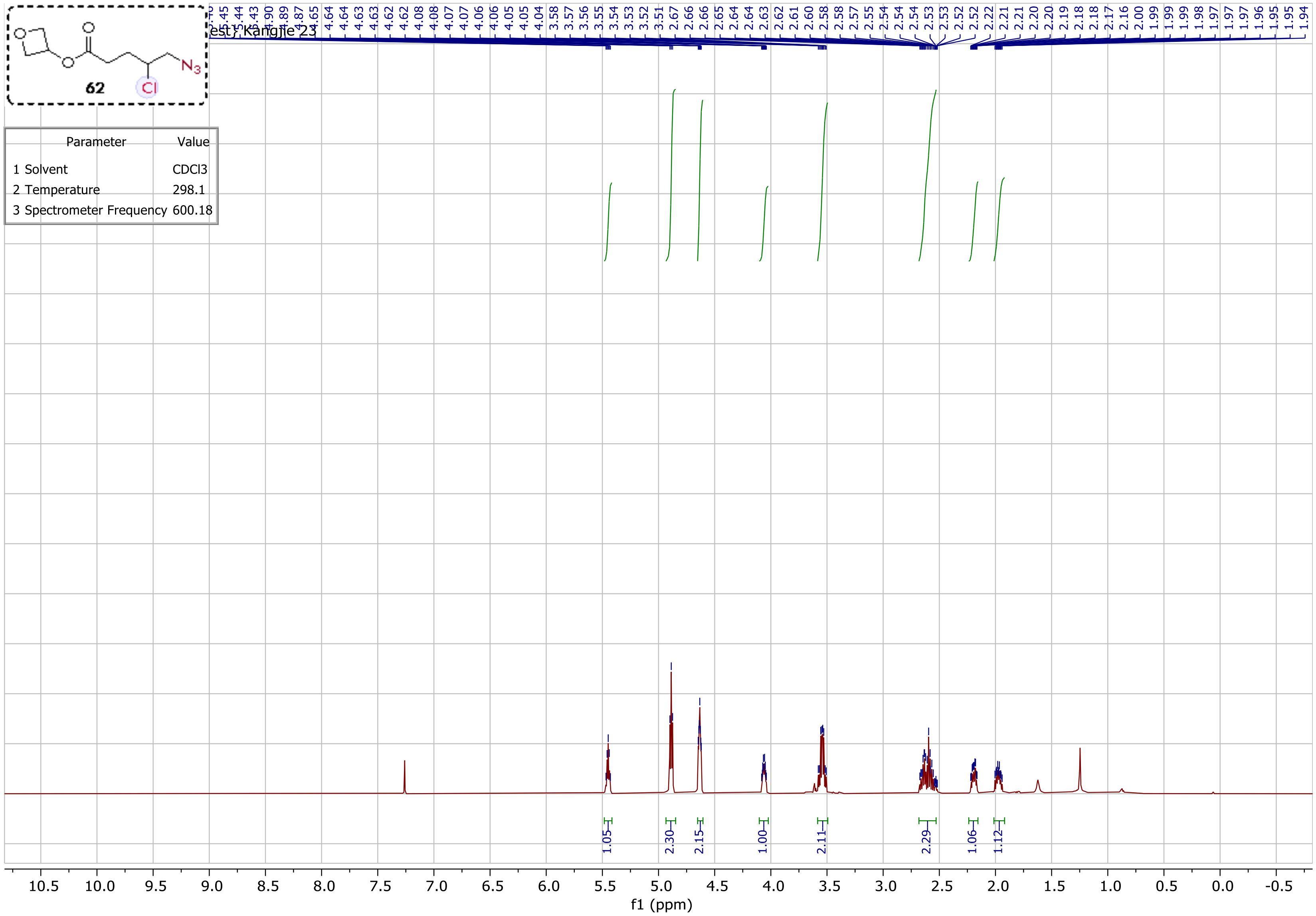

Parameter	Value
1 Solvent	CDCl3
2 Temperature	298.1
3 Spectrometer Frequency	600.18

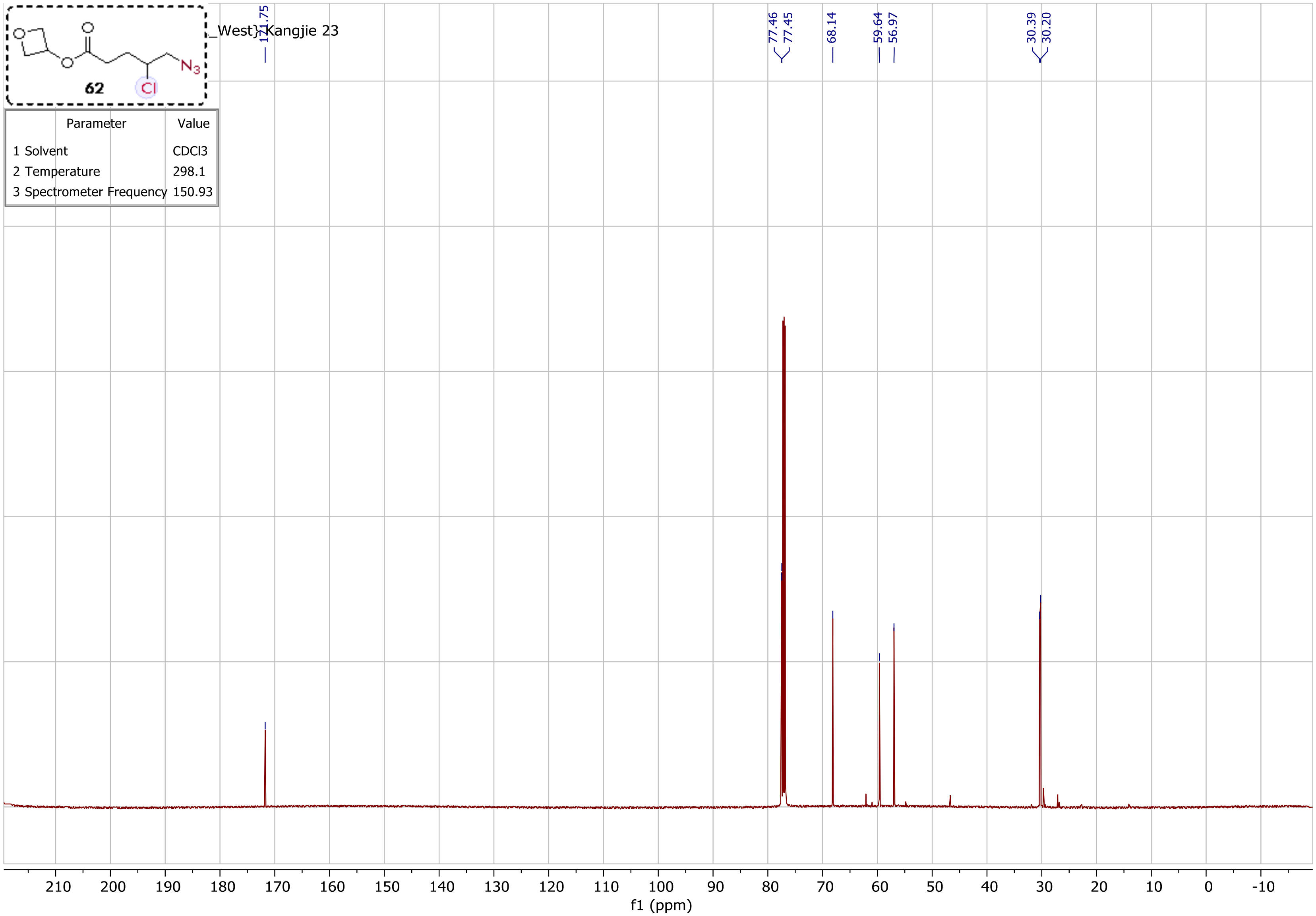
62
Cl
N3
_West}_Kangjie 23
171.75
77.46
77.45
68.14
59.64
56.97
30.39
30.20
Parameter Value
1 Solvent CDCl3
2 Temperature 298.1
3 Spectrometer Frequency 150.93
210 200 190 180 170 160 150 140 130 120 110 100 90 80 70 60 50 40 30 20 10 0 -10
f1 (ppm)

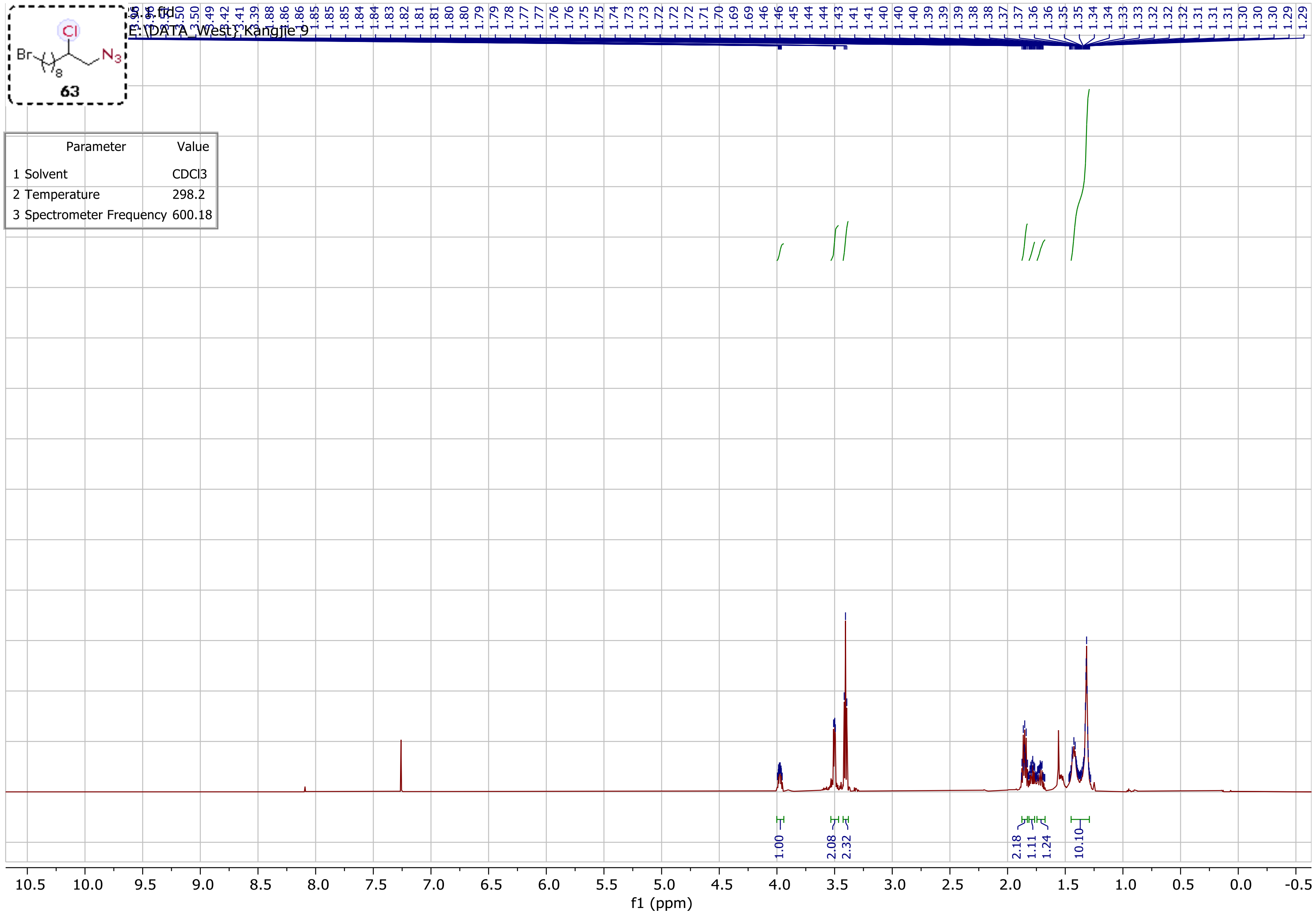

Br
Cl
N3
8
63
Parameter Value
1 Solvent CDCl3
2 Temperature 298.2
3 Spectrometer Frequency 600.18
1.00
2.08
2.32
2.18
1.11
1.24
10.10
10.5 10.0 9.5 9.0 8.5 8.0 7.5 7.0 6.5 6.0 5.5 5.0 4.5 4.0 3.5 3.0 2.5 2.0 1.5 1.0 0.5 0.0 -0.5
f1 (ppm)

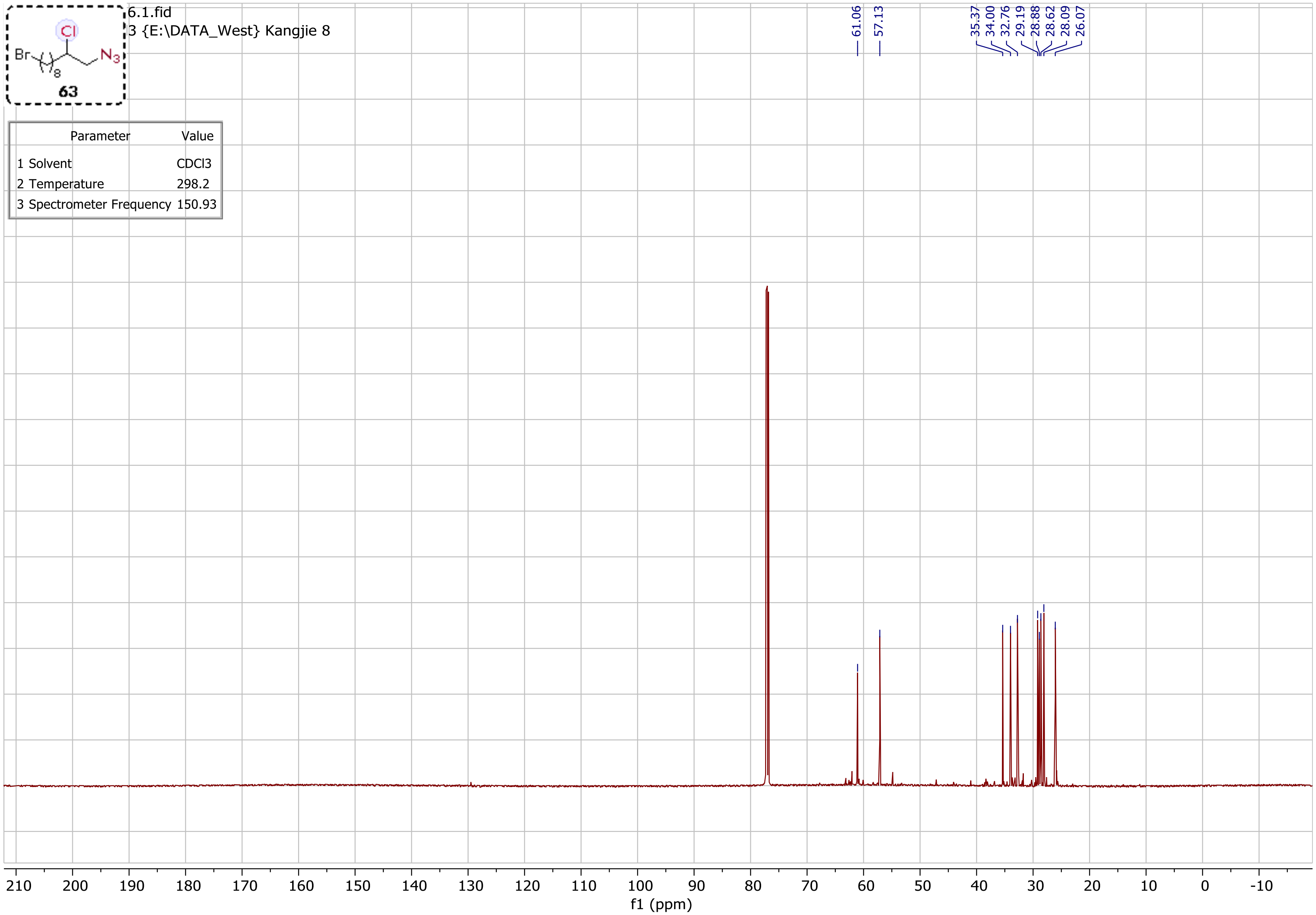

Parameter	Value
1 Solvent	CDCl3
2 Temperature	298.2
3 Spectrometer Frequency	150.93

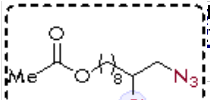

64

Parameter	Value
1 Solvent	CDCl3
2 Temperature	298.2
3 Spectrometer Frequency	600.18

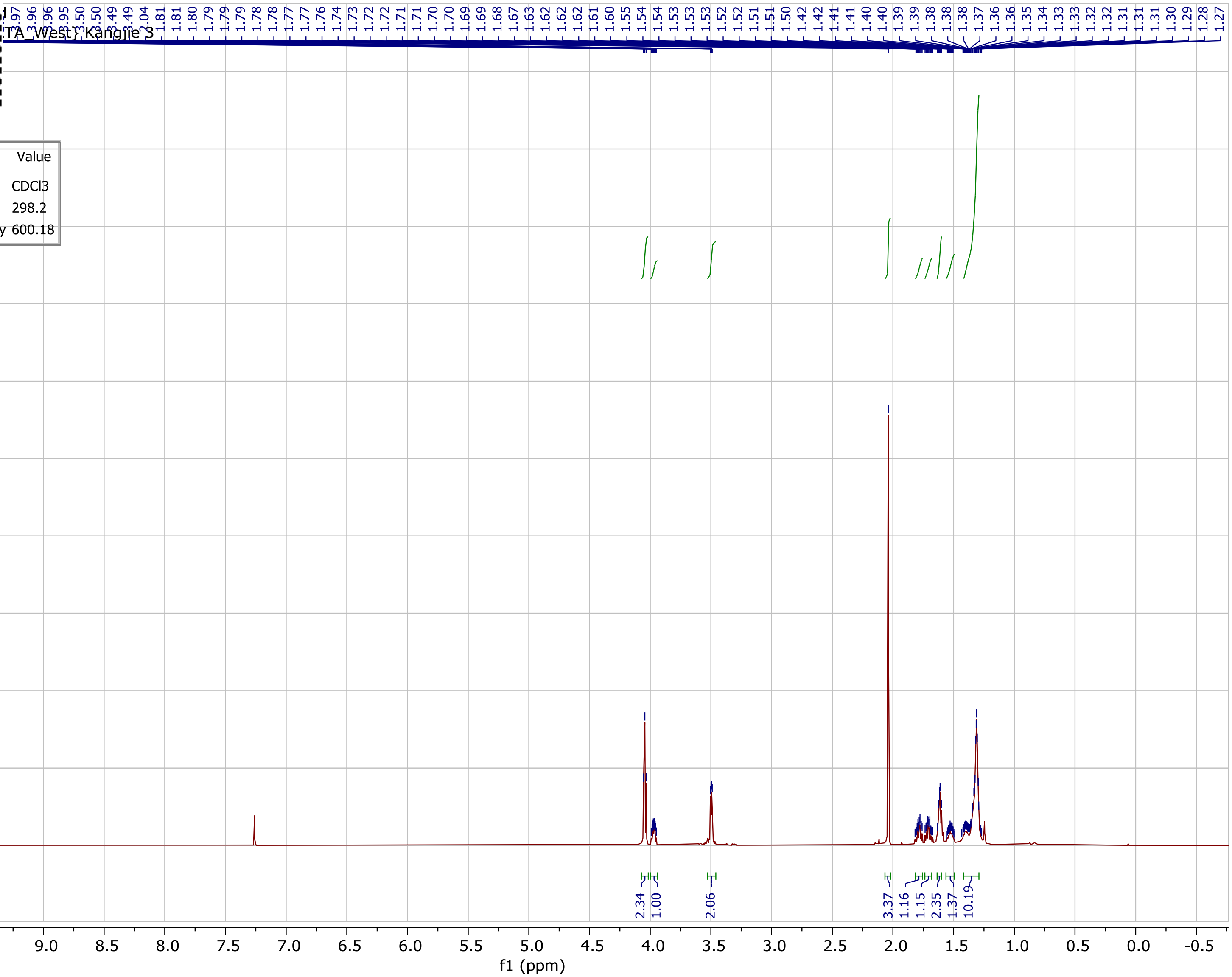

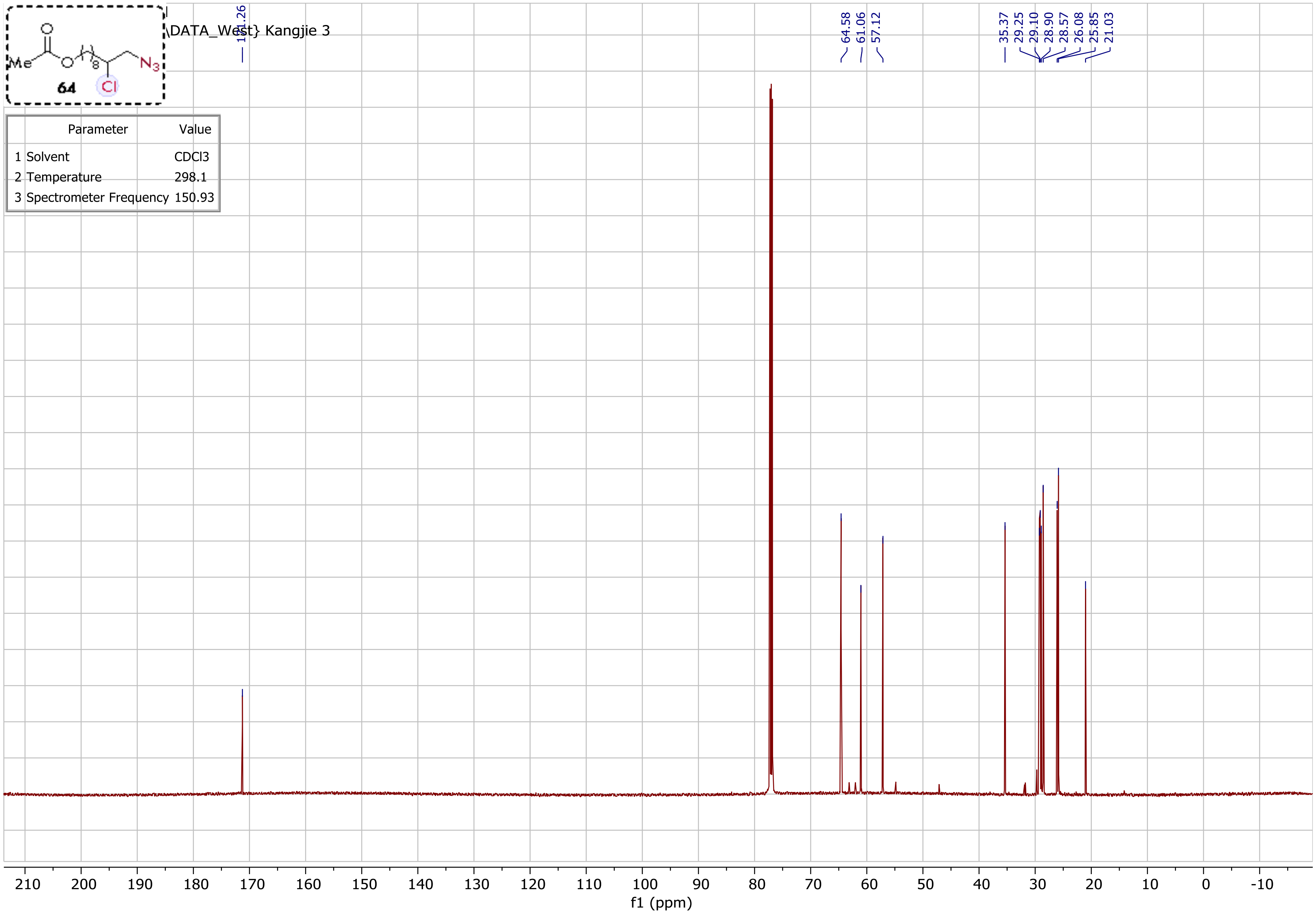

	Parameter	Value
1	Solvent	CDCl3
2	Temperature	298.1
3	Spectrometer Frequency	150.93

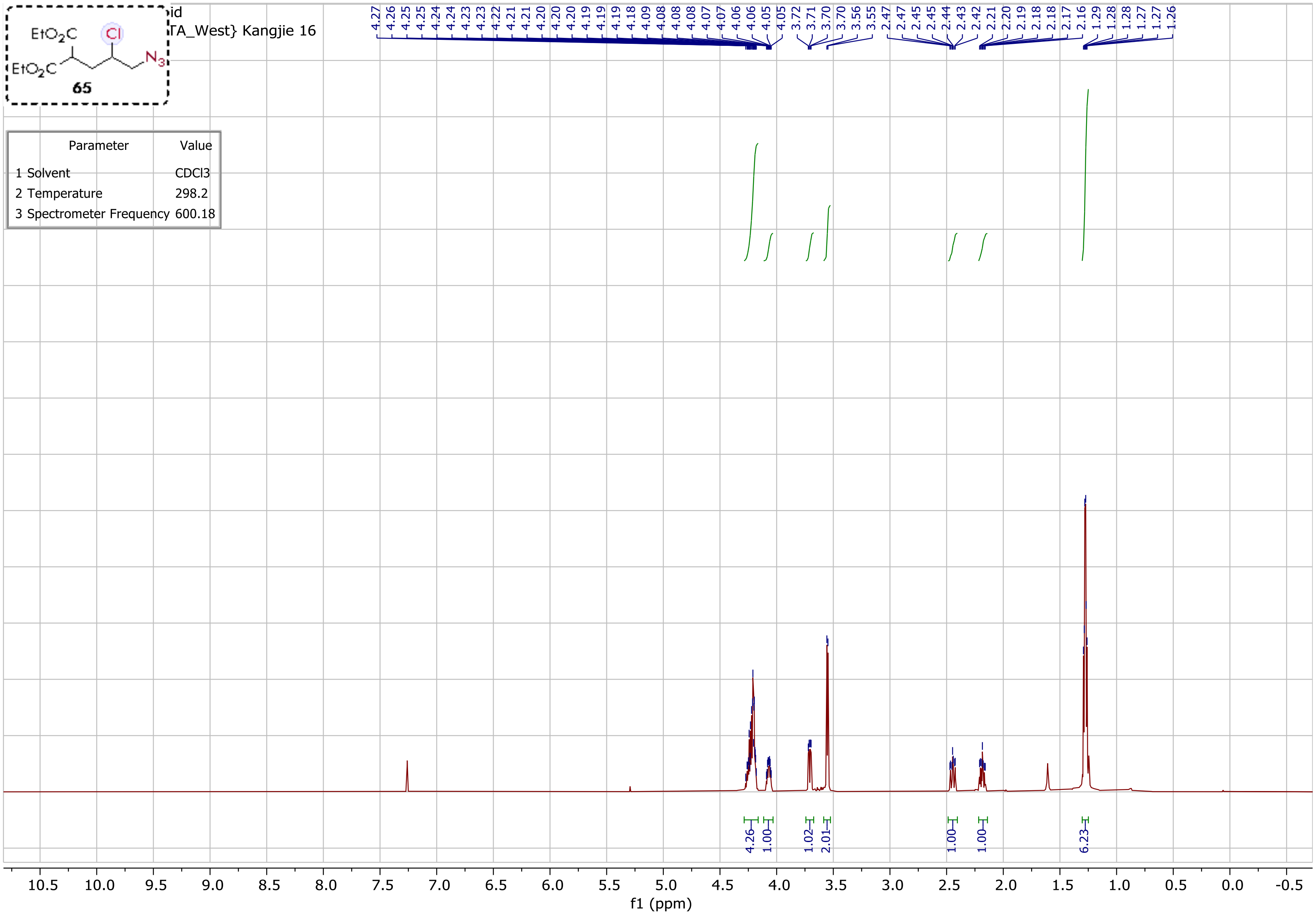

id
TA_West} Kangjie 16
EtO2C
Cl
EtO2C
N3
65
Parameter Value
1 Solvent CDCl3
2 Temperature 298.2
3 Spectrometer Frequency 600.18
4.27 4.26 4.25 4.25 4.24 4.24 4.23 4.23 4.22 4.21 4.21 4.20 4.20 4.20 4.19 4.19 4.19 4.18 4.09 4.08 4.08 4.08 4.07 4.07 4.06 4.05 4.05 3.72 3.71 3.70 3.70 3.56 3.55 2.47 2.47 2.45 2.45 2.44 2.43 2.42 2.21 2.21 2.20 2.19 2.18 2.18 2.17 2.16 1.29 1.28 1.28 1.27 1.27 1.26
4.26 1.00 1.02 2.01 1.00 1.00 6.23
10.5 10.0 9.5 9.0 8.5 8.0 7.5 7.0 6.5 6.0 5.5 5.0 4.5 4.0 3.5 3.0 2.5 2.0 1.5 1.0 0.5 0.0 -0.5
f1 (ppm)

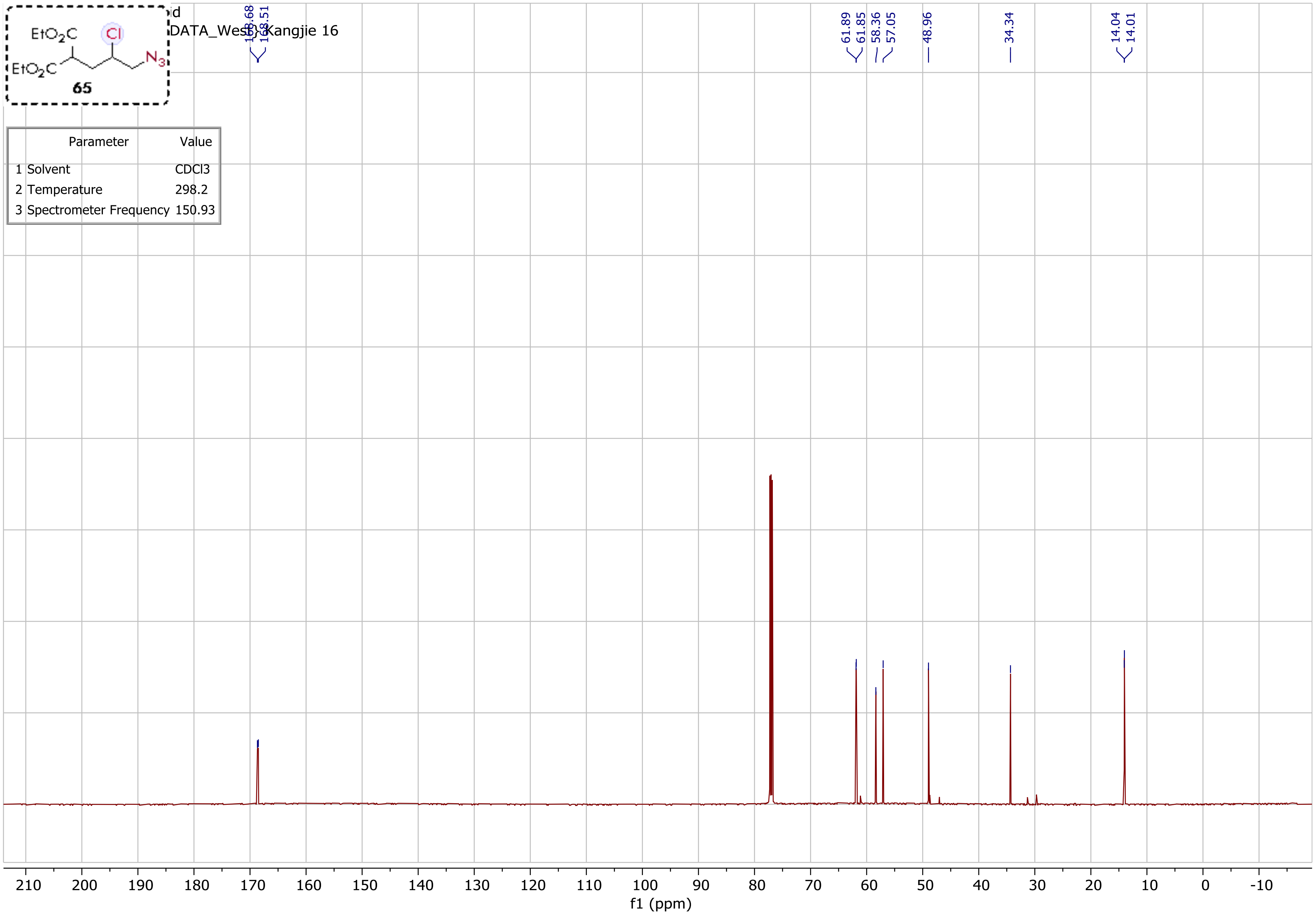

EtO2C
Cl
EtO2C
N3
65
DATA_West
Kangjie 16
Parameter Value
1 Solvent CDCl3
2 Temperature 298.2
3 Spectrometer Frequency 150.93
168.68
168.51
61.89
61.85
58.36
57.05
48.96
34.34
14.04
14.01
210 200 190 180 170 160 150 140 130 120 110 100 90 80 70 60 50 40 30 20 10 0 -10
f1 (ppm)

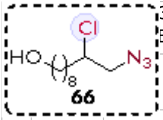

Parameter	Value
1 Solvent	CDCl3
2 Temperature	298.2
3 Spectrometer Frequency	600.18

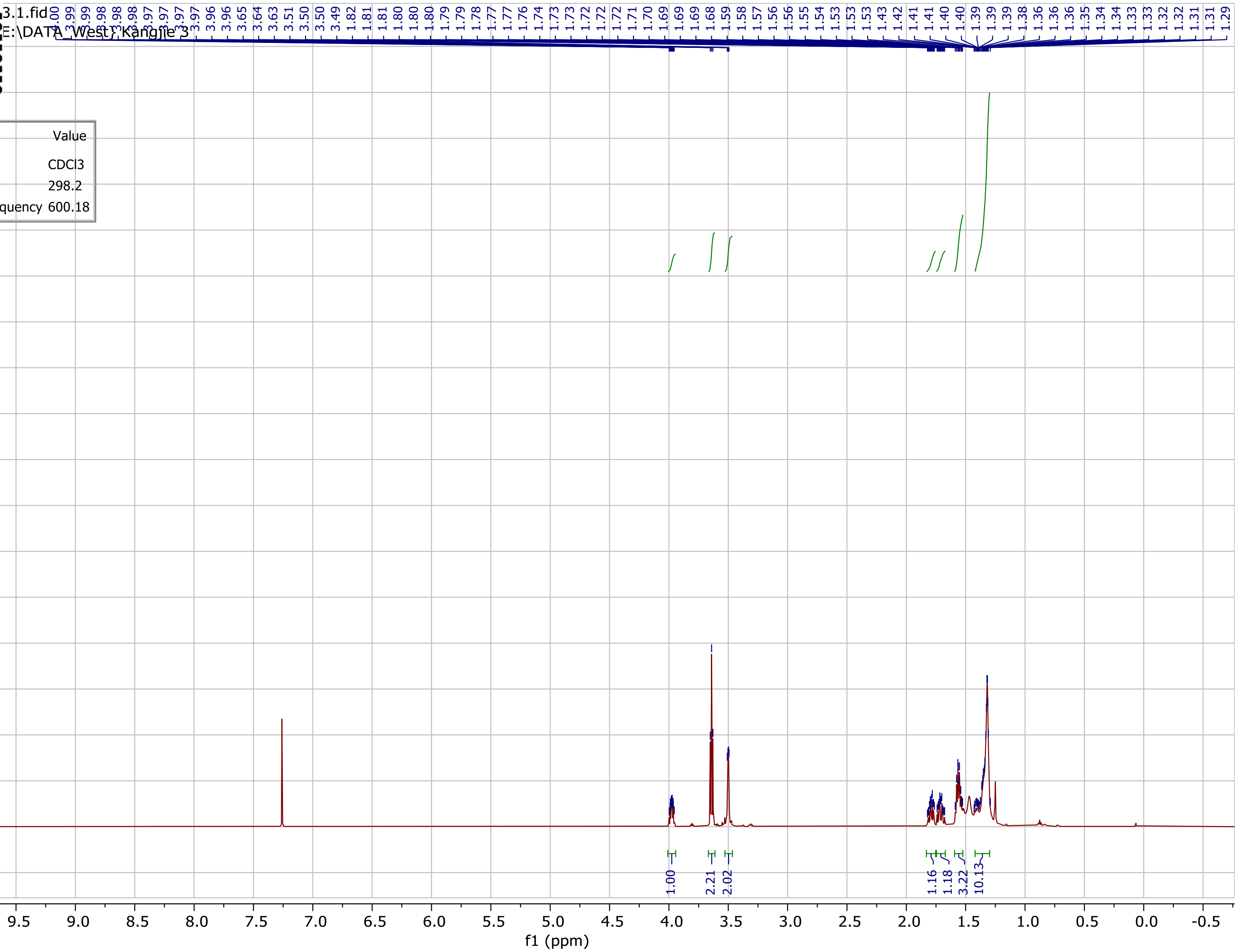

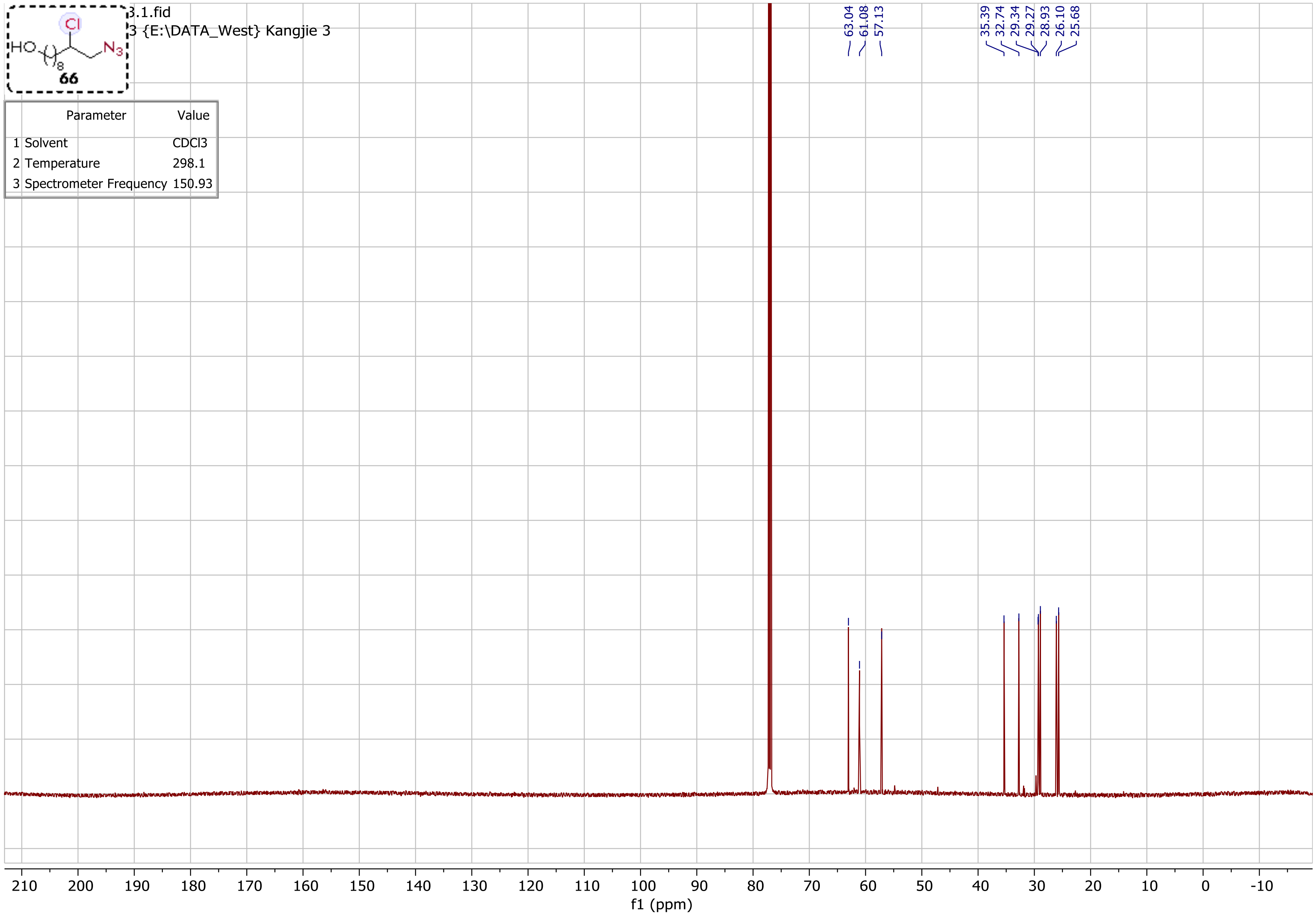

	Parameter	Value
1	Solvent	CDCl3
2	Temperature	298.1
3	Spectrometer Frequency	150.93

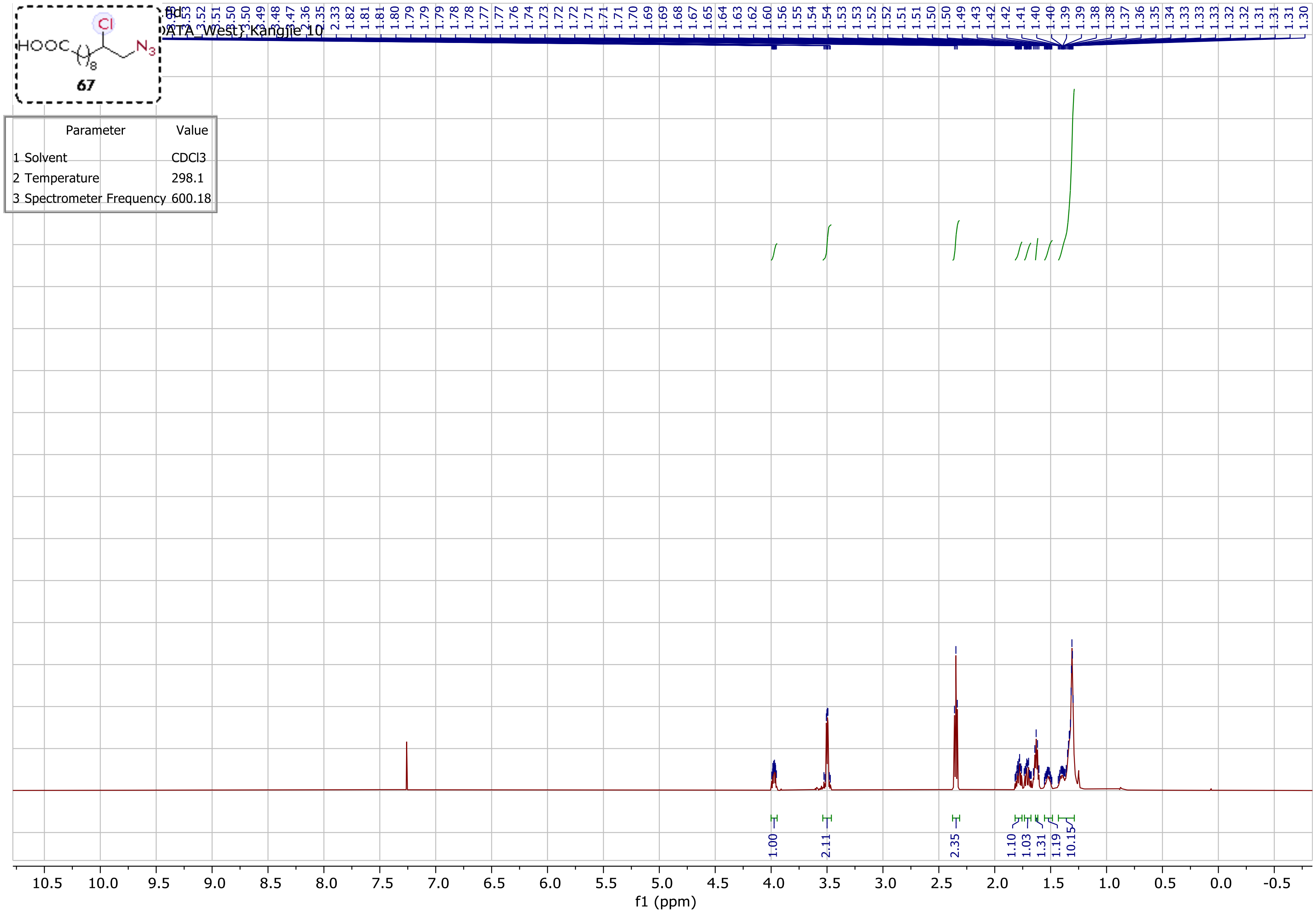

HOOC
Cl
N3
8
67
Parameter | Value
1 Solvent CDCl3
2 Temperature 298.1
3 Spectrometer Frequency 600.18
1.00
2.11
2.35
1.10
1.03
1.31
1.19
10.15
f1 (ppm)

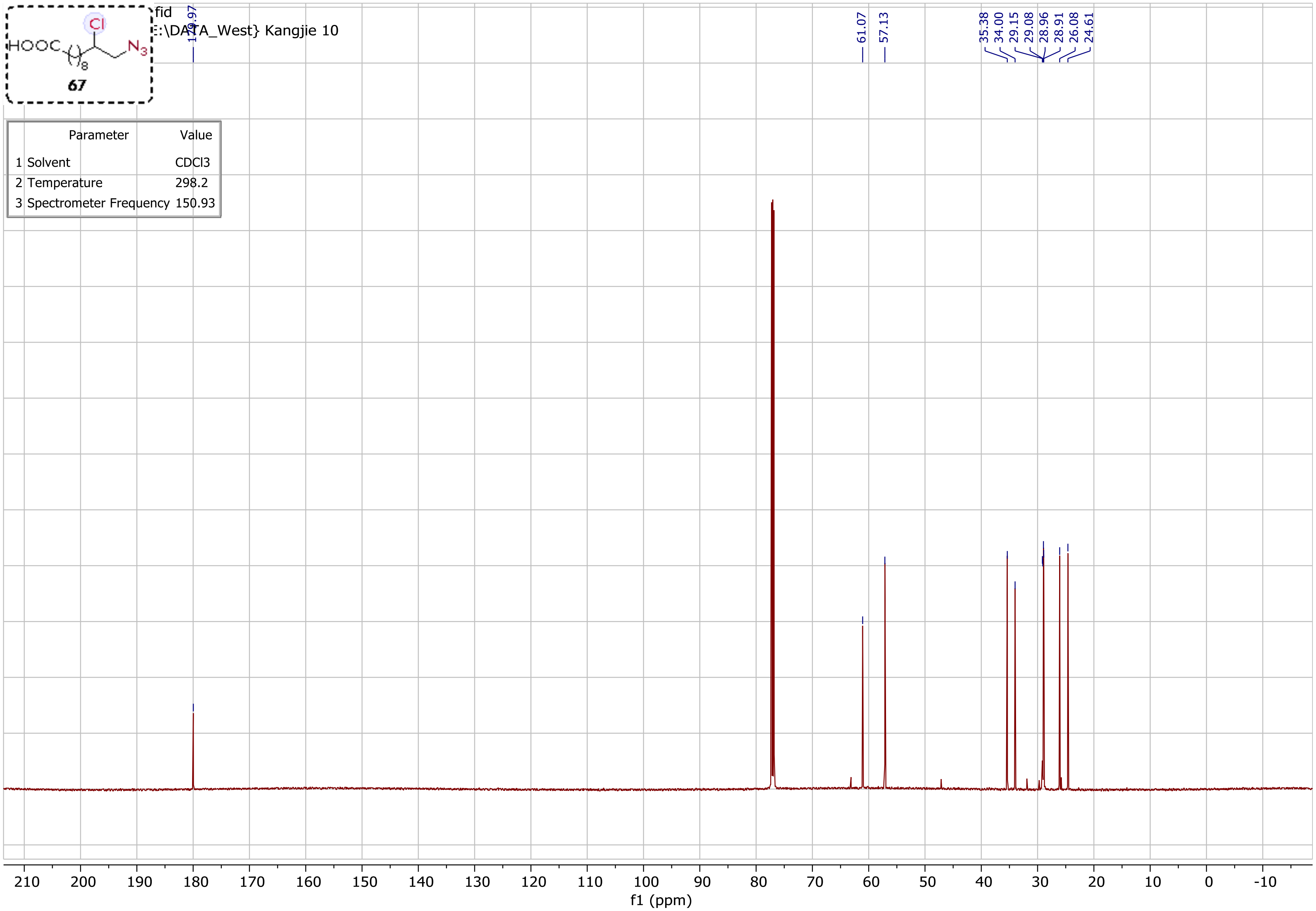

	Parameter	Value
1	Solvent	CDCl3
2	Temperature	298.2
3	Spectrometer Frequency	150.93

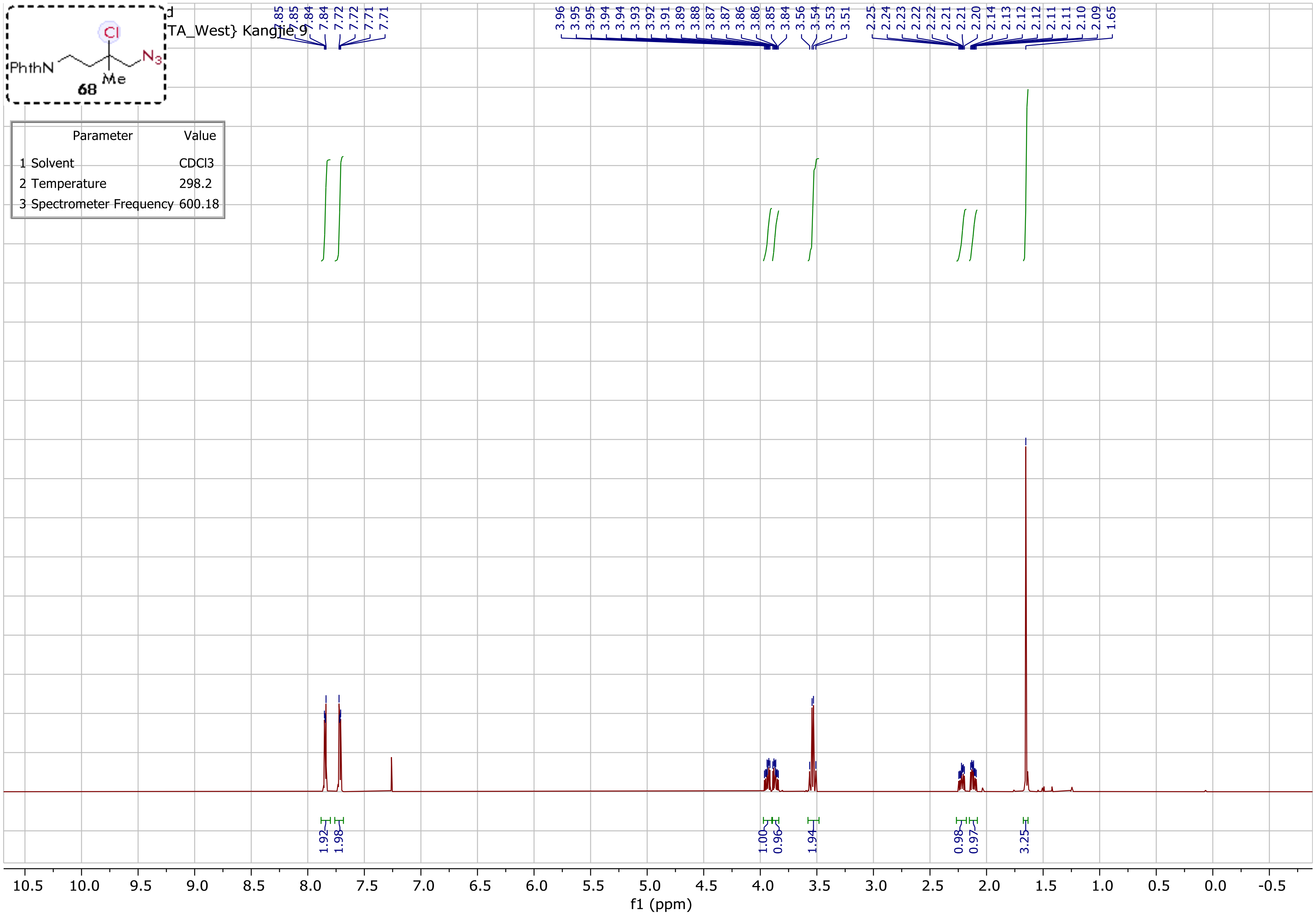

PhthN
Cl
N3
Me
68
TA_West} Kangjie 9
Parameter Value
1 Solvent CDCl3
2 Temperature 298.2
3 Spectrometer Frequency 600.18
f1 (ppm)

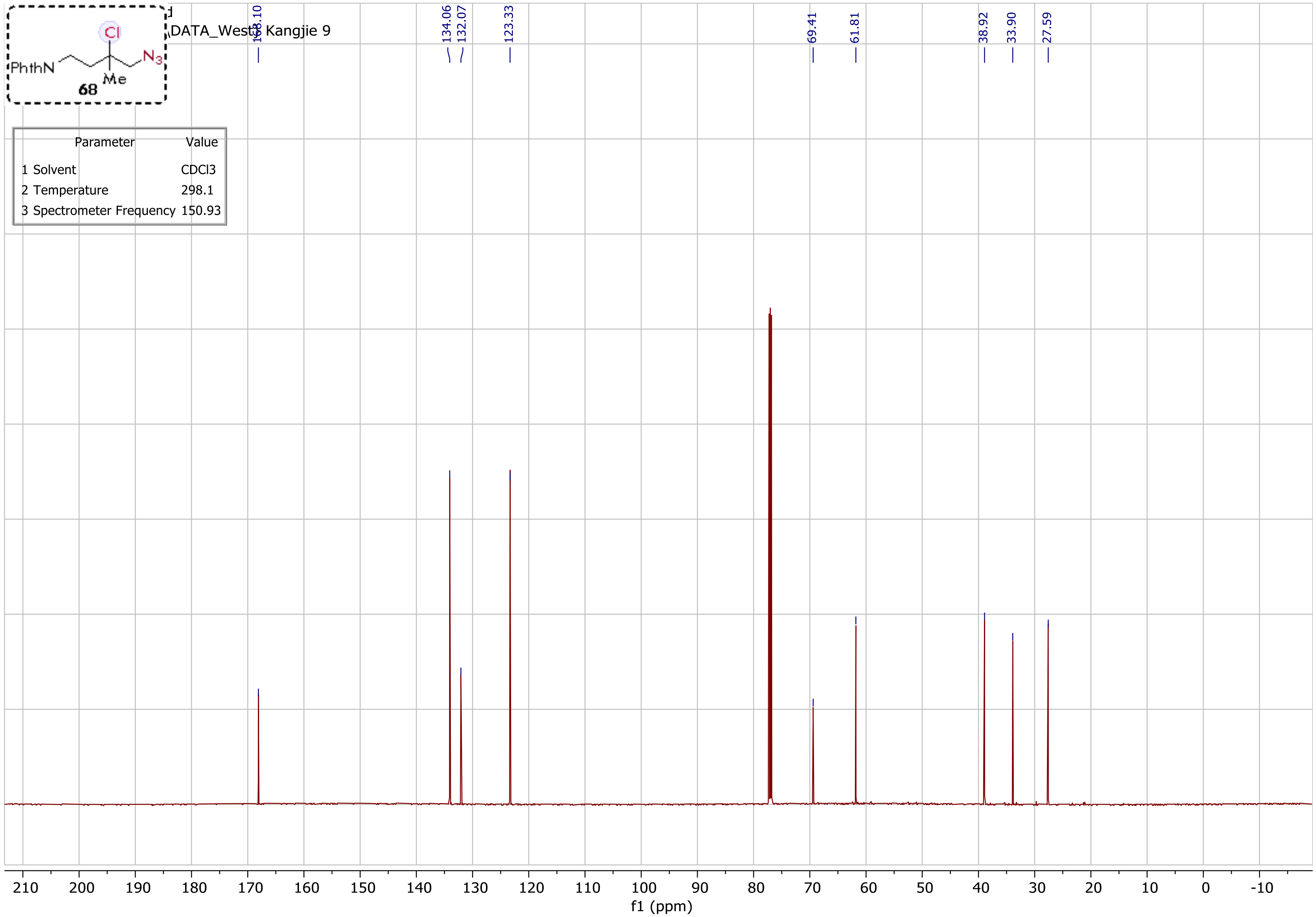

Parameter	Value
1 Solvent	CDCl3
2 Temperature	298.1
3 Spectrometer Frequency	150.93

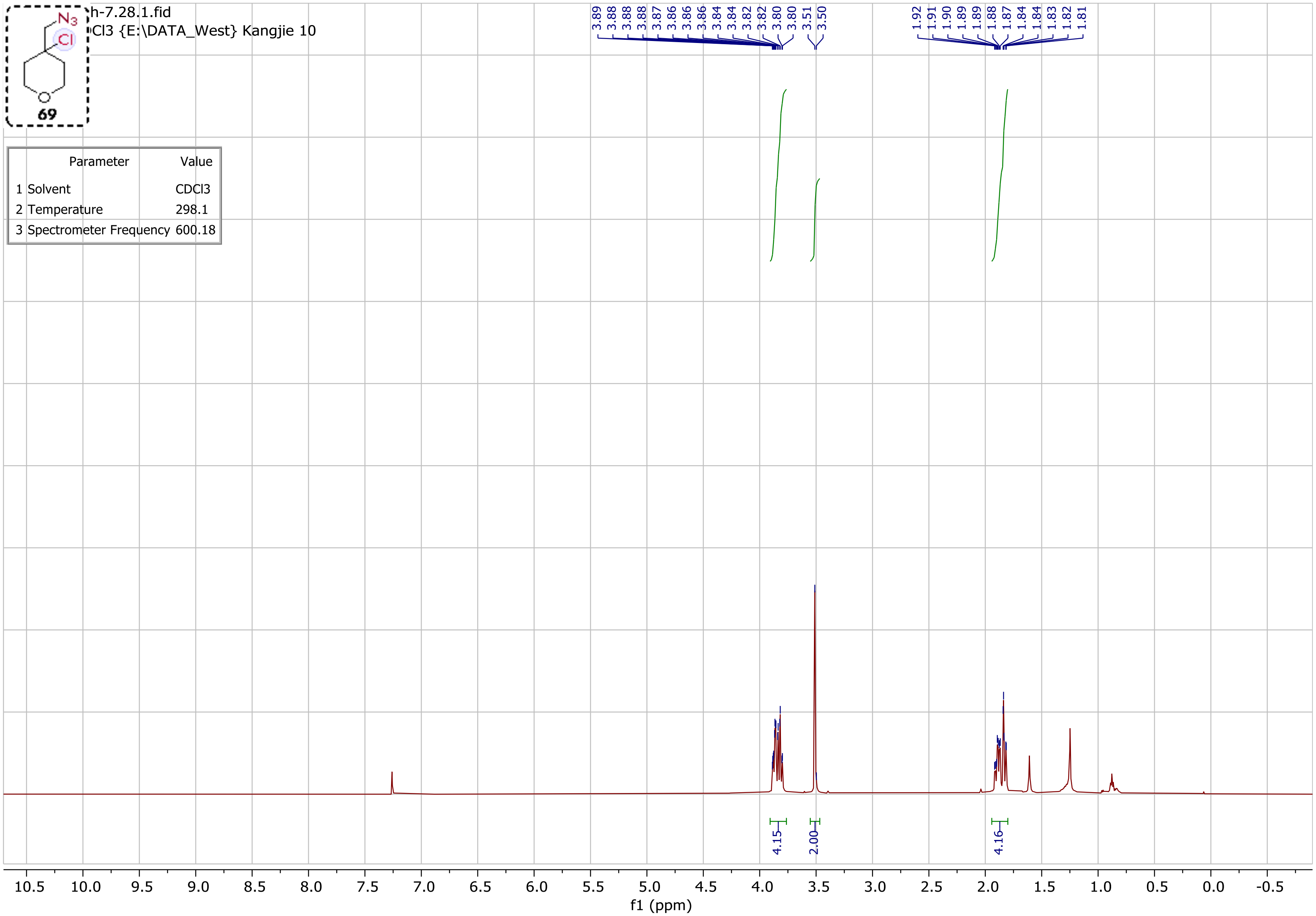
N3
Cl
69
h-7.28.1.fid
Cl3 {E:\DATA_West} Kangjie 10
Parameter Value
1 Solvent CDCl3
2 Temperature 298.1
3 Spectrometer Frequency 600.18
3.89 3.88 3.88 3.88 3.87 3.86 3.86 3.86 3.84 3.84 3.82 3.82 3.80 3.80 3.51 3.50
1.92 1.91 1.90 1.89 1.89 1.88 1.87 1.84 1.84 1.83 1.82 1.81
4.15
2.00
4.16
10.5 10.0 9.5 9.0 8.5 8.0 7.5 7.0 6.5 6.0 5.5 5.0 4.5 4.0 3.5 3.0 2.5 2.0 1.5 1.0 0.5 0.0 -0.5
f1 (ppm)

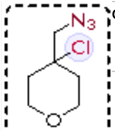

69

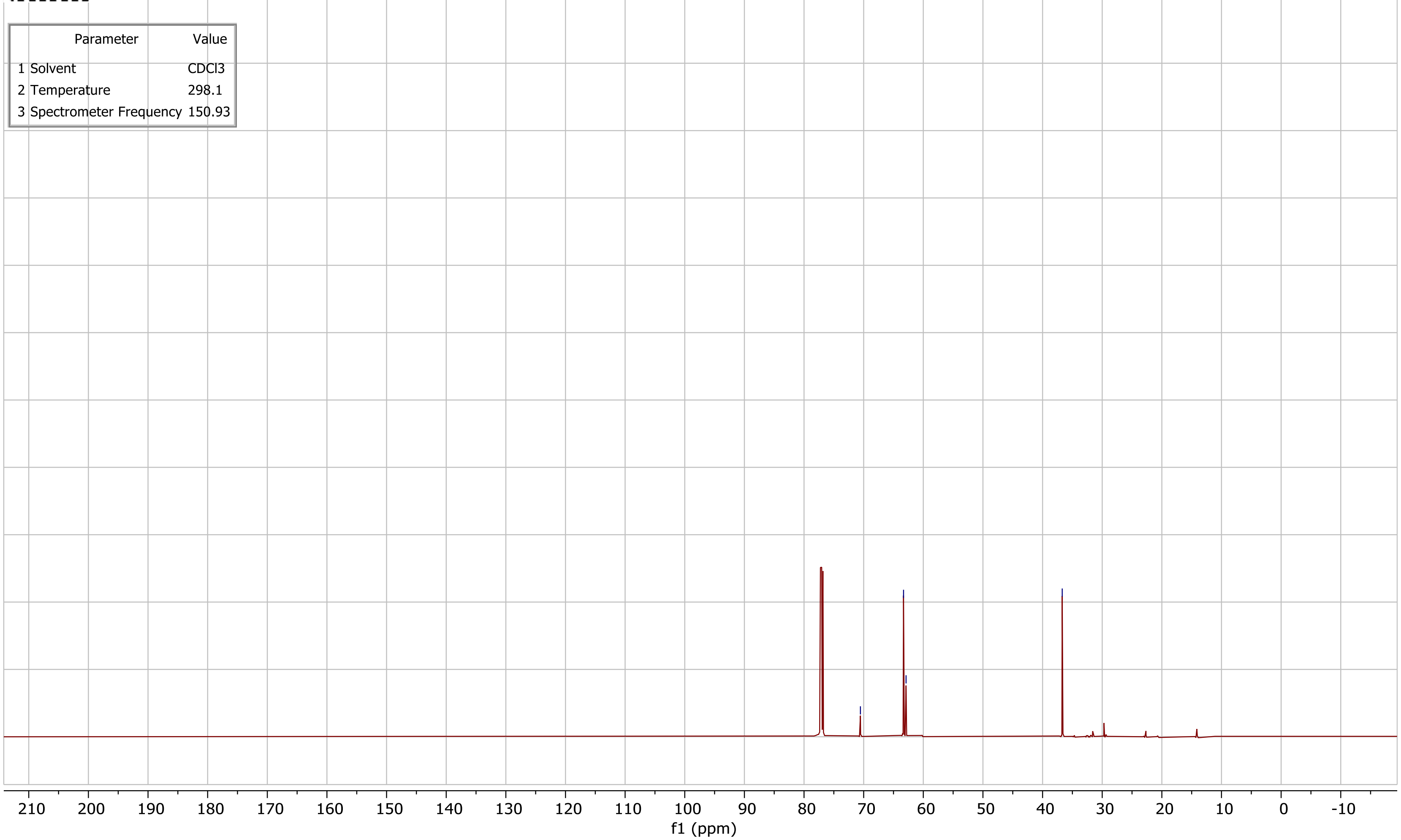

	Parameter	Value
1	Solvent	CDCl3
2	Temperature	298.1
3	Spectrometer Frequency	150.93

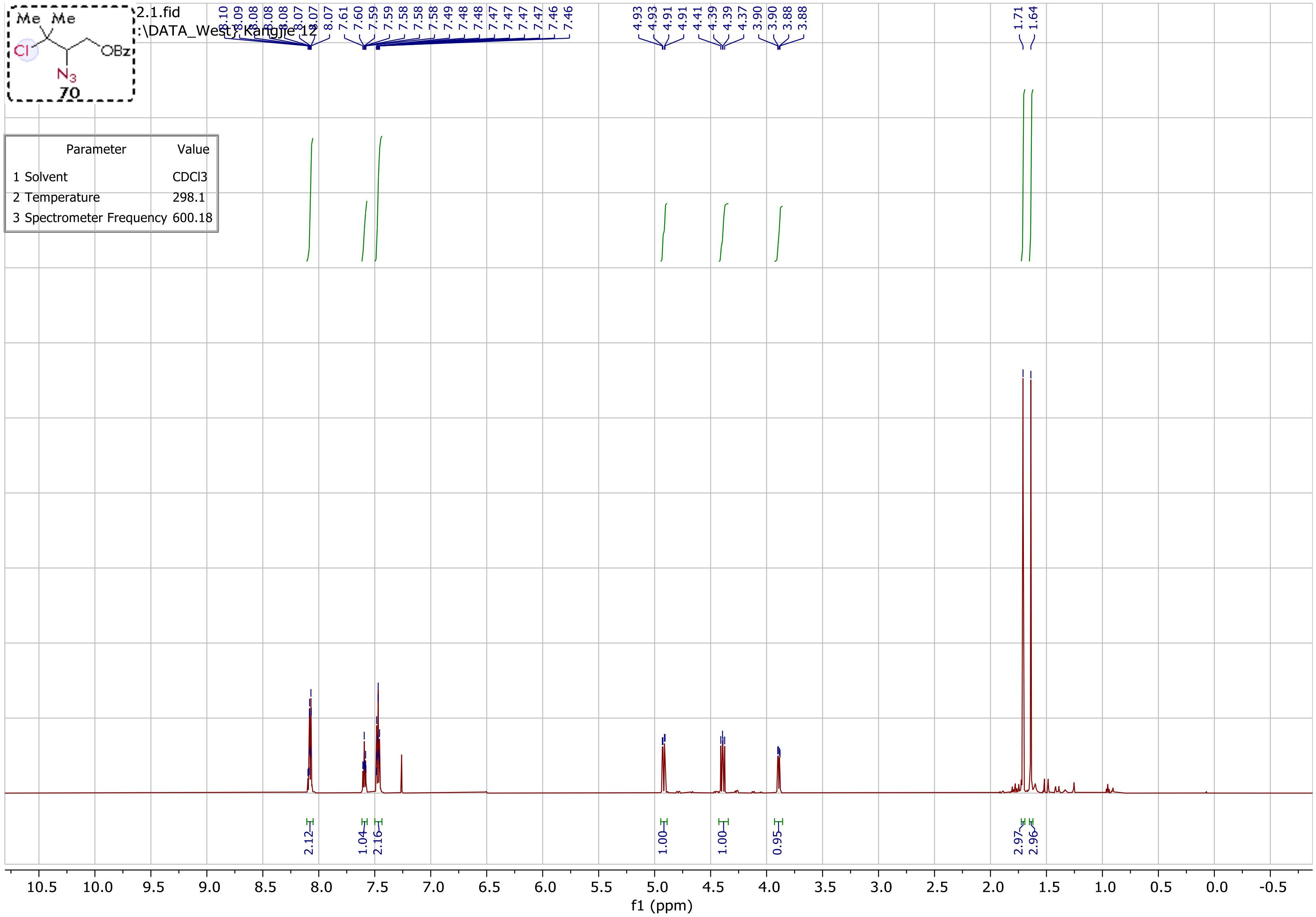
Me Me
Cl
OBz
N3
70
2.1.fid
:\DATA_West\Kangjie 12
Parameter Value
1 Solvent CDCl3
2 Temperature 298.1
3 Spectrometer Frequency 600.18
8.10 8.09 8.08 8.08 8.08 8.07 8.07 8.07 7.61 7.60 7.59 7.59 7.58 7.58 7.58 7.49 7.48 7.48 7.47 7.47 7.47 7.47 7.46 7.46
4.93 4.93 4.91 4.91 4.41 4.39 4.39 4.37 3.90 3.90 3.88 3.88
1.71 1.64
2.12 1.04 2.16 1.00 1.00 0.95 2.97 2.96
10.5 10.0 9.5 9.0 8.5 8.0 7.5 7.0 6.5 6.0 5.5 5.0 4.5 4.0 3.5 3.0 2.5 2.0 1.5 1.0 0.5 0.0 -0.5
f1 (ppm)

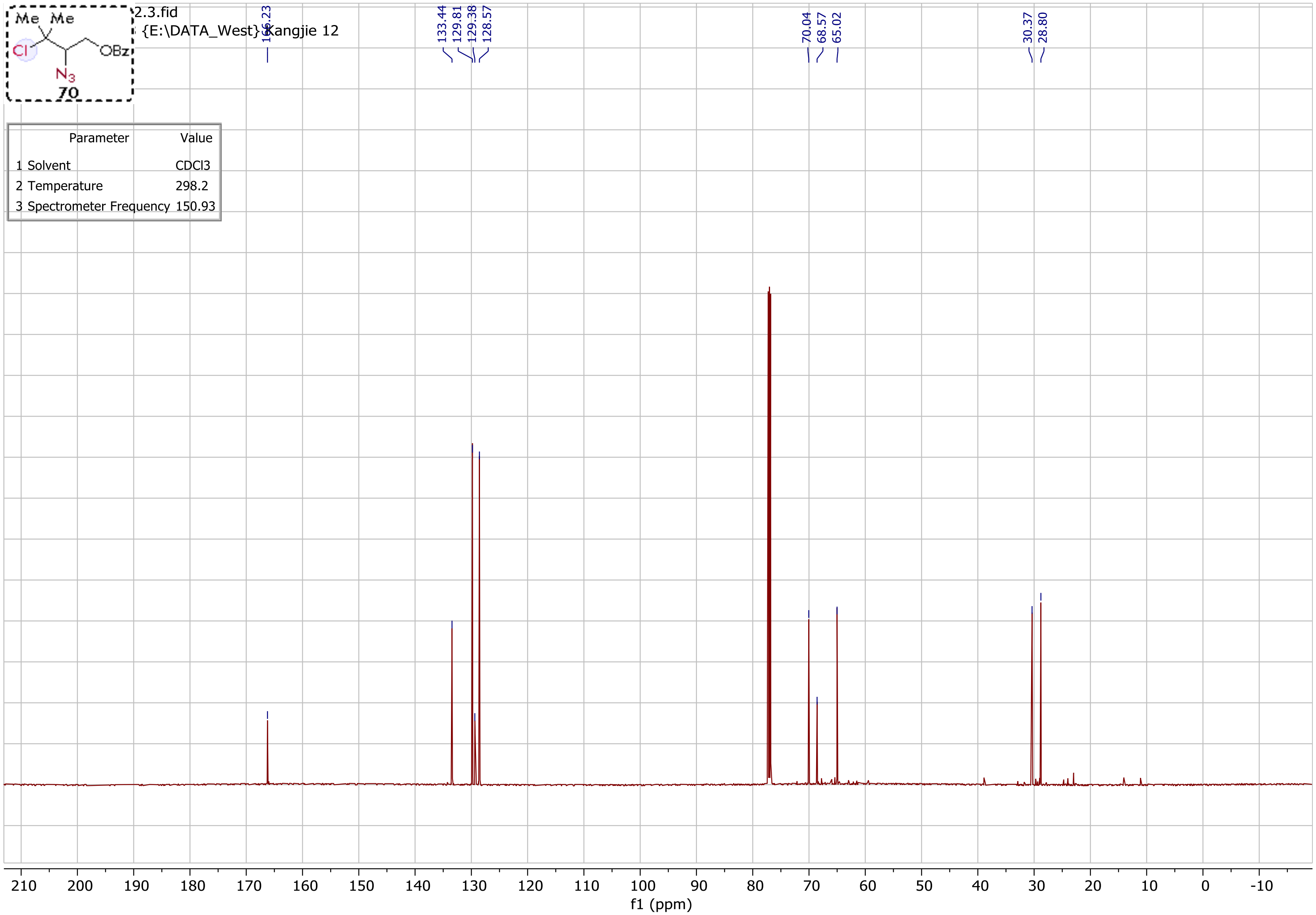

Me Me
Cl
OBz
N3
70
2.3.fid
{E:\DATA_West} Kangjie 12
Parameter Value
1 Solvent CDCl3
2 Temperature 298.2
3 Spectrometer Frequency 150.93
133.44
129.81
129.38
128.57
70.04
68.57
65.02
30.37
28.80
210 200 190 180 170 160 150 140 130 120 110 100 90 80 70 60 50 40 30 20 10 0 -10
f1 (ppm)

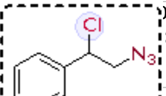

71

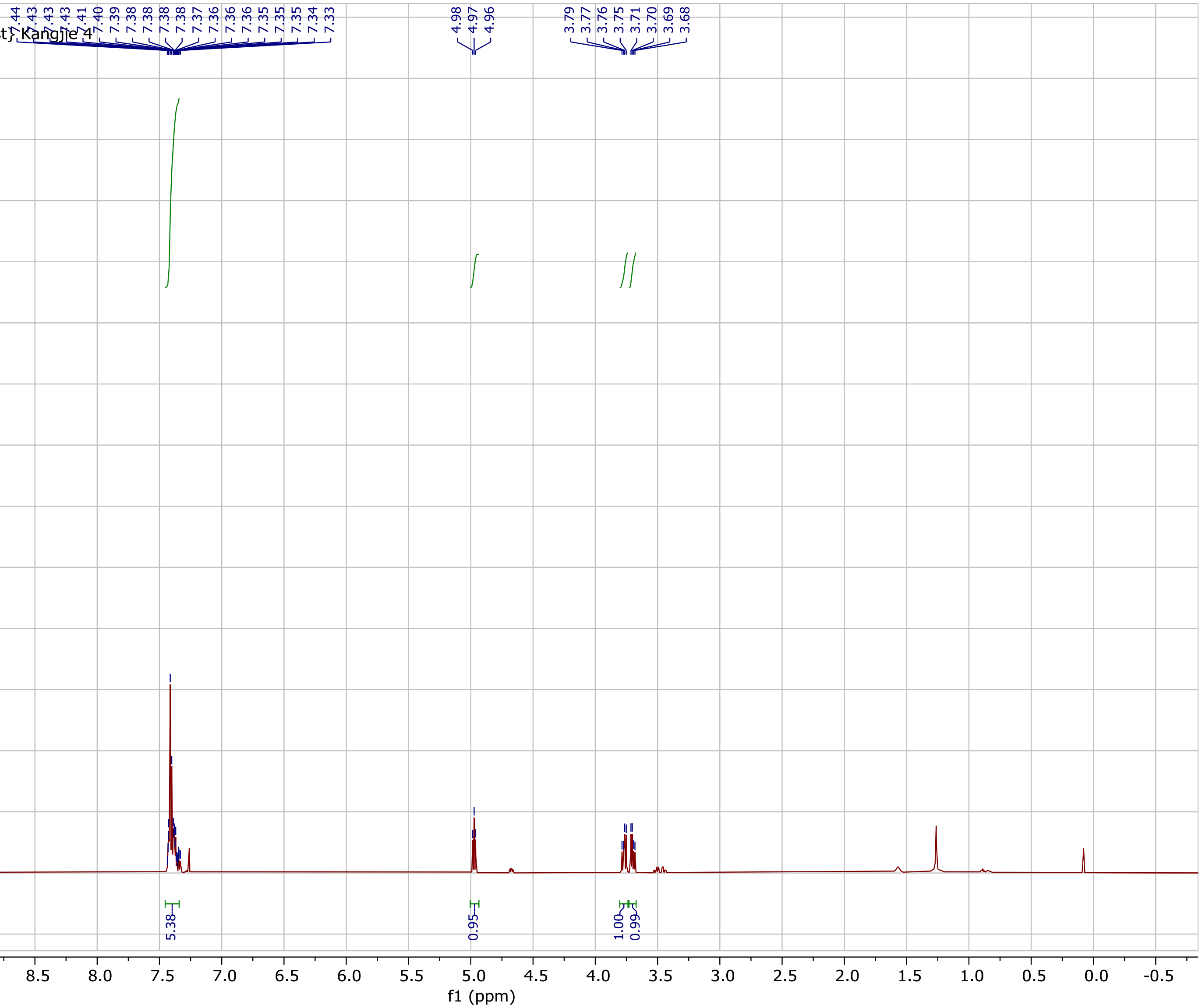

Parameter	Value
1 Solvent	CDCl3
2 Temperature	298.2
3 Spectrometer Frequency	600.18

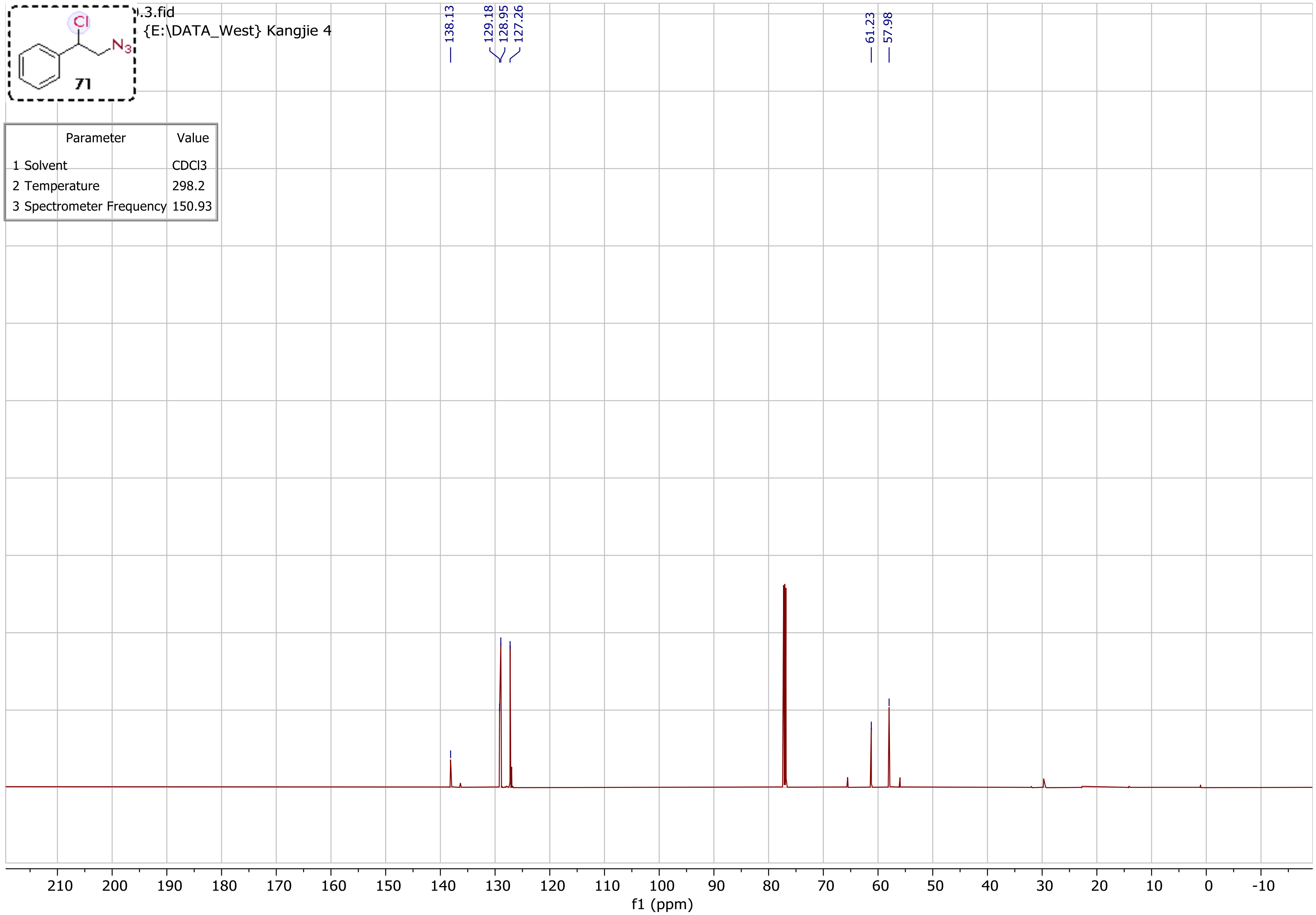

	Parameter	Value
1	Solvent	CDCl3
2	Temperature	298.2
3	Spectrometer Frequency	150.93

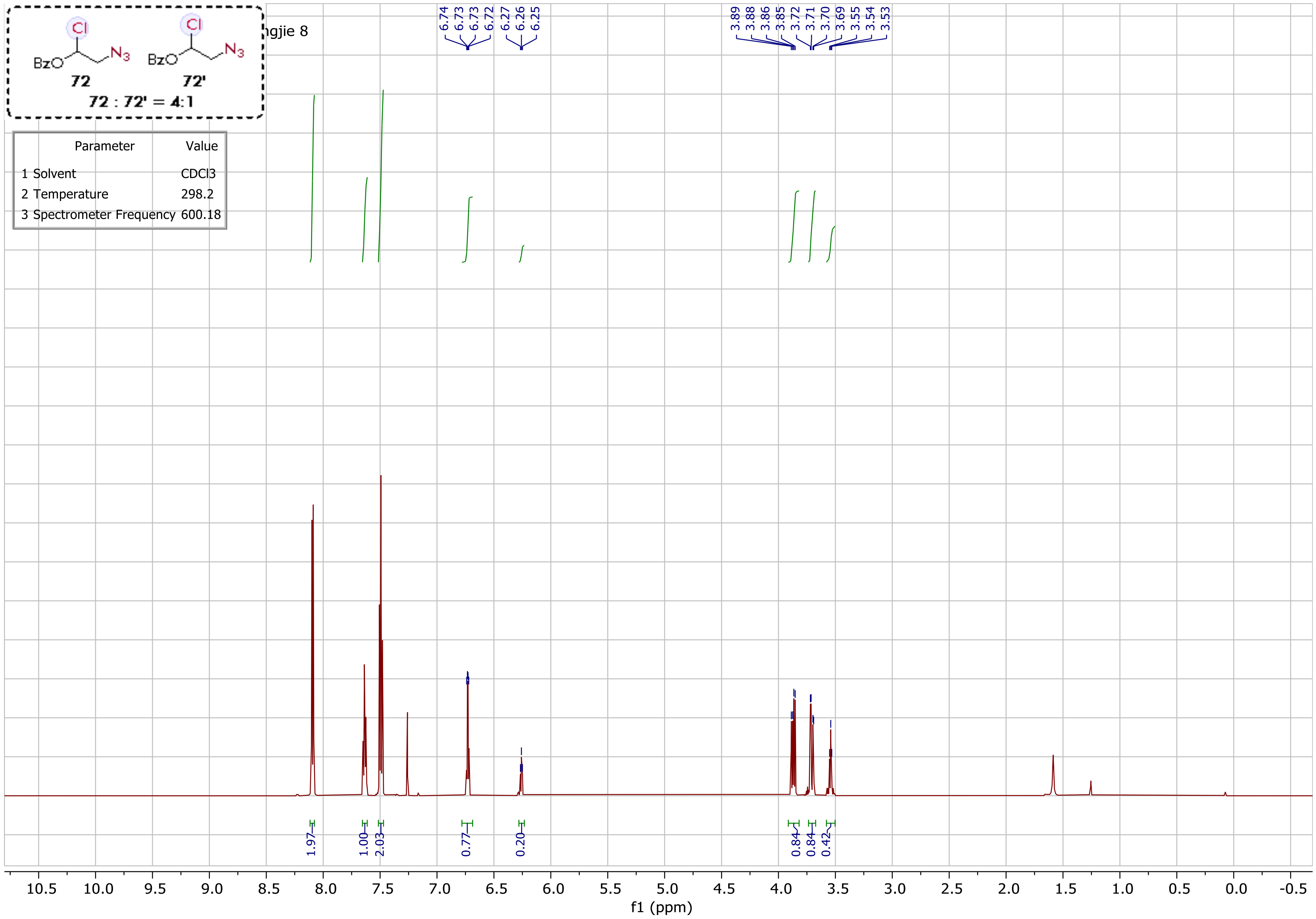

72
72'
72 : 72' = 4:1
Parameter Value
1 Solvent CDCl3
2 Temperature 298.2
3 Spectrometer Frequency 600.18
f1 (ppm)

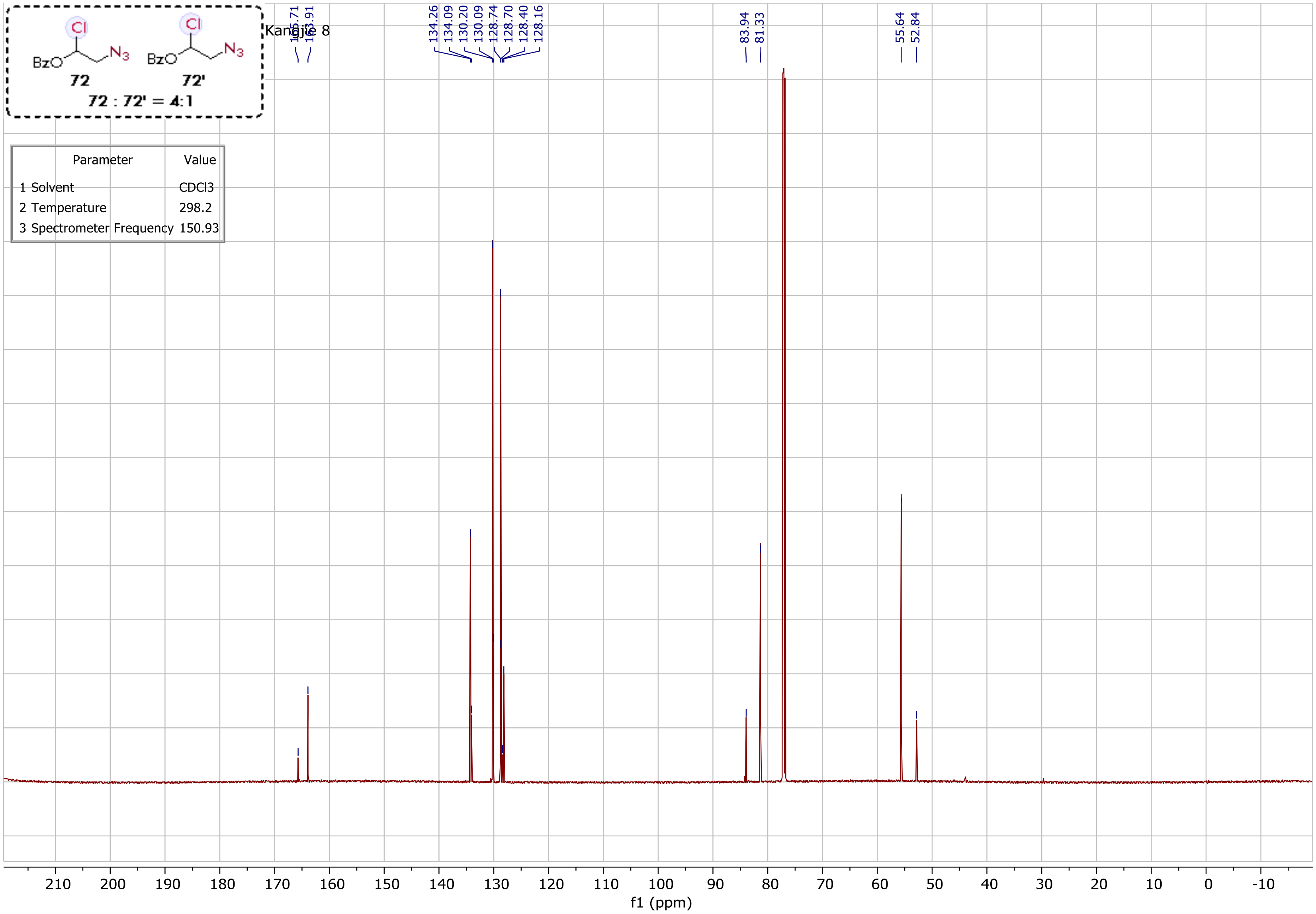
Cl
BzO
N3
72
72'
72 : 72' = 4:1
Kangjie 8
165.71
163.91
134.26
134.09
130.20
130.09
128.74
128.70
128.40
128.16
83.94
81.33
55.64
52.84
Parameter
Value
1 Solvent
CDCl3
2 Temperature
298.2
3 Spectrometer Frequency
150.93
210
200
190
180
170
160
150
140
130
120
110
100
90
80
70
60
50
40
30
20
10
0
-10
f1 (ppm)

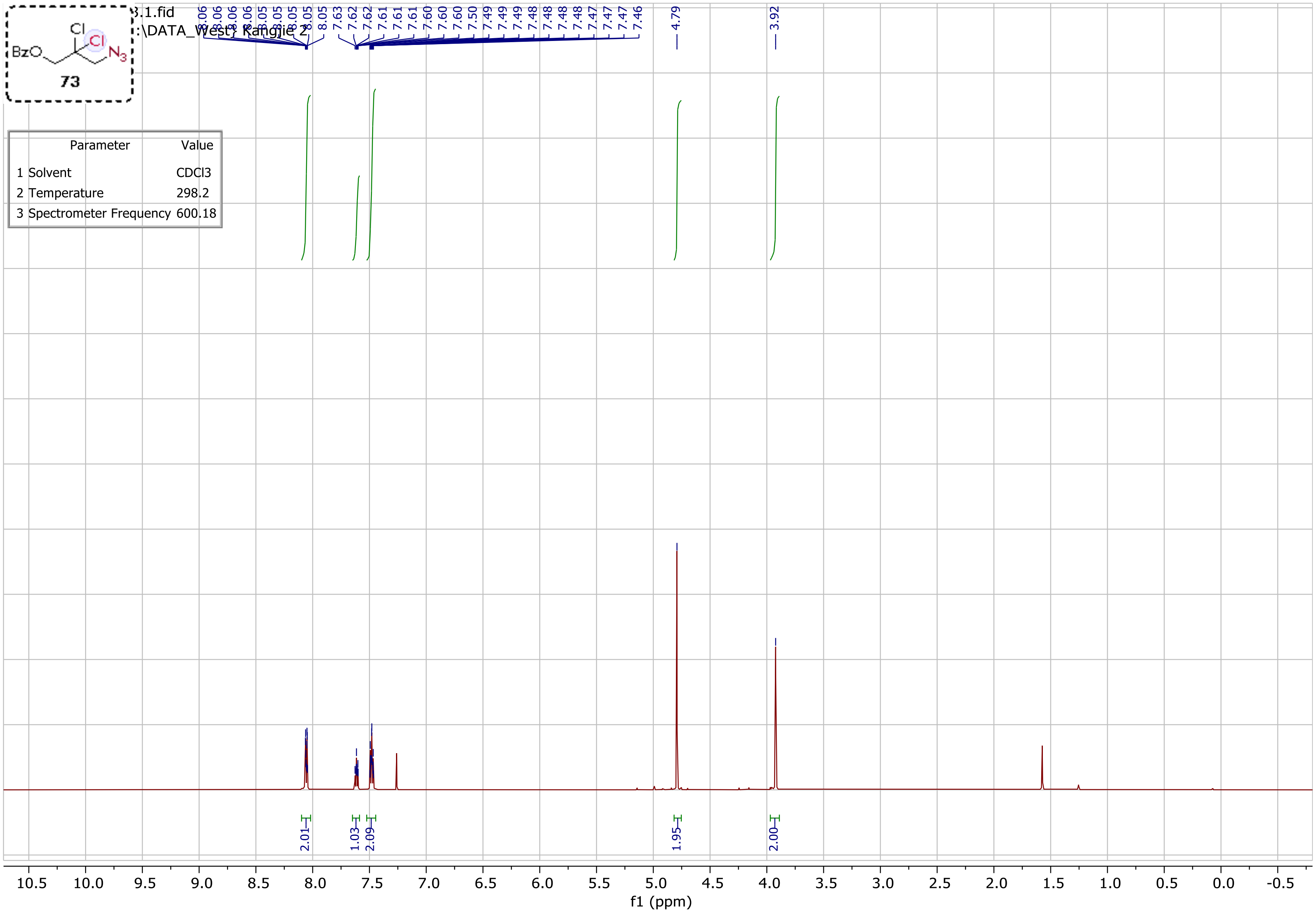
Cl
Cl
BzO
N3
73
3.1.fid
:\DATA_West\ Kangjie 2
8.06
8.06
8.06
8.06
8.05
8.05
8.05
8.05
8.05
7.63
7.62
7.62
7.61
7.61
7.61
7.60
7.60
7.60
7.50
7.49
7.49
7.49
7.48
7.48
7.48
7.48
7.47
7.47
7.47
7.46
4.79
3.92
Parameter Value
1 Solvent CDCl3
2 Temperature 298.2
3 Spectrometer Frequency 600.18
2.01
1.03
2.09
1.95
2.00
10.5 10.0 9.5 9.0 8.5 8.0 7.5 7.0 6.5 6.0 5.5 5.0 4.5 4.0 3.5 3.0 2.5 2.0 1.5 1.0 0.5 0.0 -0.5
f1 (ppm)

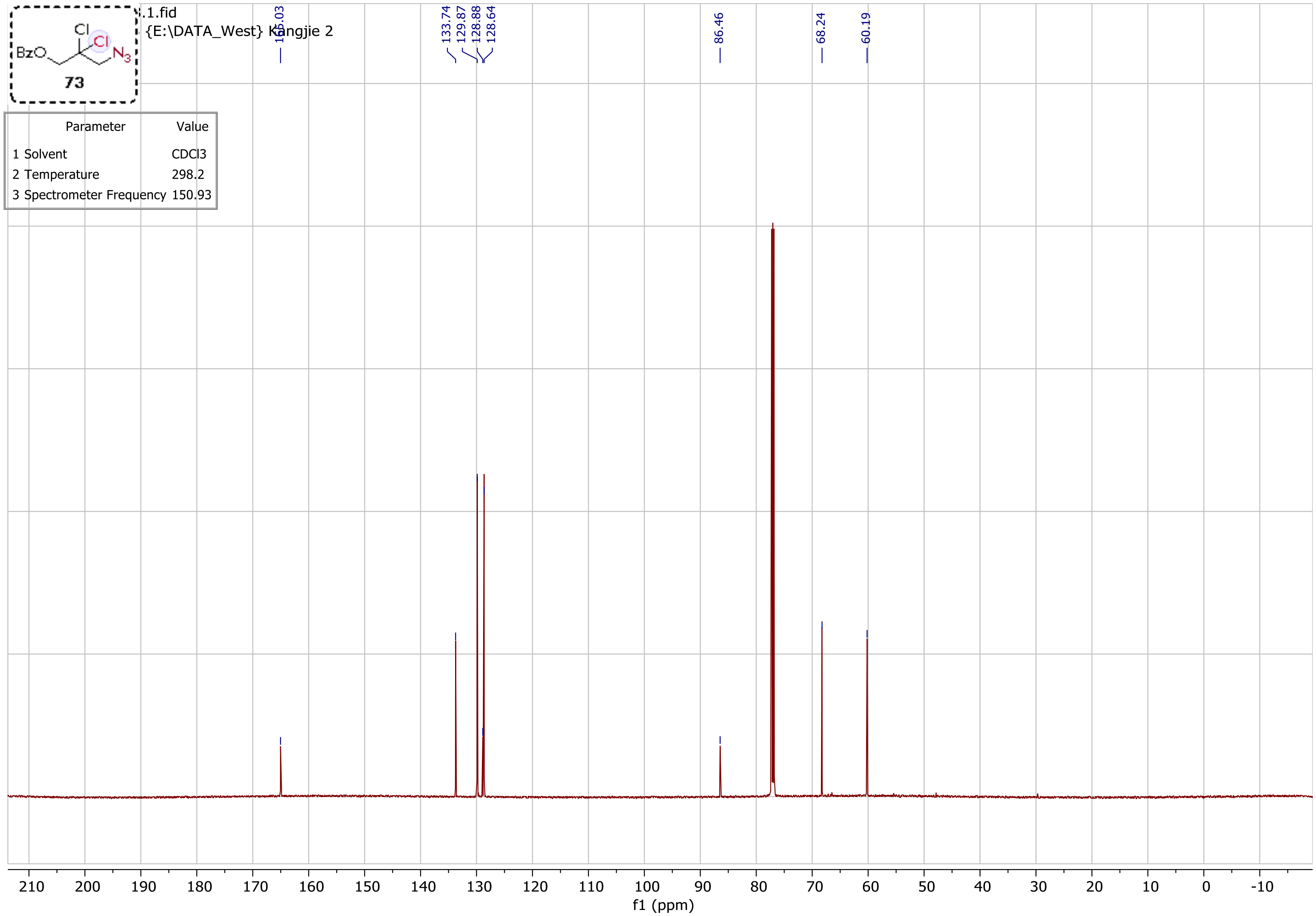
.1.fid
{E:\DATA_West} Kangjie 2
BzO
Cl
Cl
N3
73
Parameter Value
1 Solvent CDCl3
2 Temperature 298.2
3 Spectrometer Frequency 150.93
133.74
129.87
128.88
128.64
86.46
68.24
60.19
210 200 190 180 170 160 150 140 130 120 110 100 90 80 70 60 50 40 30 20 10 0 -10
f1 (ppm)

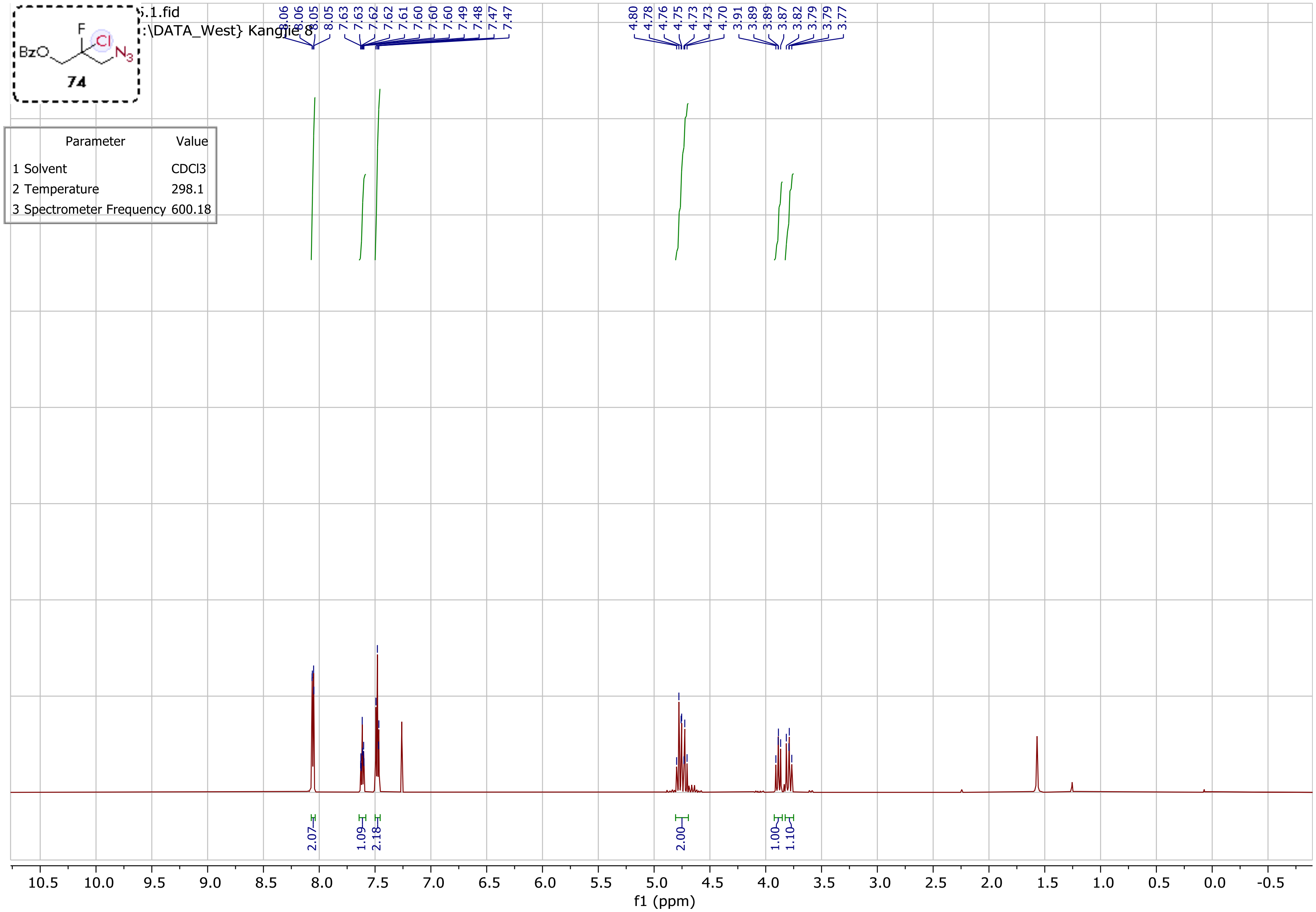
BzO
F
Cl
N3
74
5.1.fid
:\DATA_West} Kangjie 8
Parameter Value
1 Solvent CDCl3
2 Temperature 298.1
3 Spectrometer Frequency 600.18
8.06 8.06 8.05 8.05 7.63 7.63 7.62 7.62 7.61 7.60 7.60 7.60 7.49 7.48 7.47 7.47
4.80 4.78 4.76 4.75 4.73 4.73 4.70 3.91 3.89 3.89 3.87 3.82 3.79 3.79 3.77
2.07 1.09 2.18 2.00 1.00 1.10
10.5 10.0 9.5 9.0 8.5 8.0 7.5 7.0 6.5 6.0 5.5 5.0 4.5 4.0 3.5 3.0 2.5 2.0 1.5 1.0 0.5 0.0 -0.5
f1 (ppm)

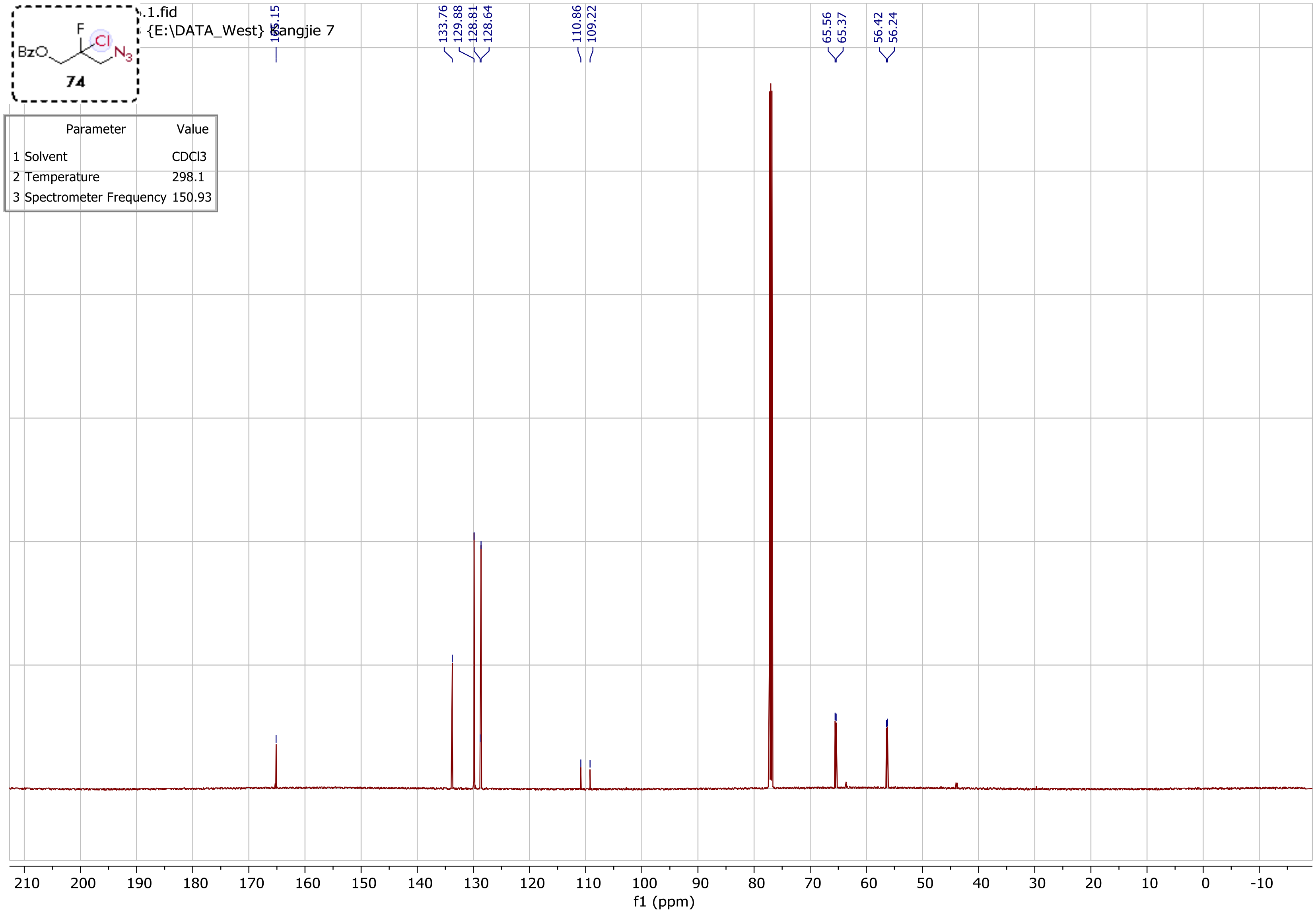
BzO
F
Cl
N3
74
.1.fid
{E:\DATA_West}
133.76
129.88
128.81
128.64
110.86
109.22
65.56
65.37
56.42
56.24
Parameter Value
1 Solvent CDCl3
2 Temperature 298.1
3 Spectrometer Frequency 150.93
210 200 190 180 170 160 150 140 130 120 110 100 90 80 70 60 50 40 30 20 10 0 -10
f1 (ppm)

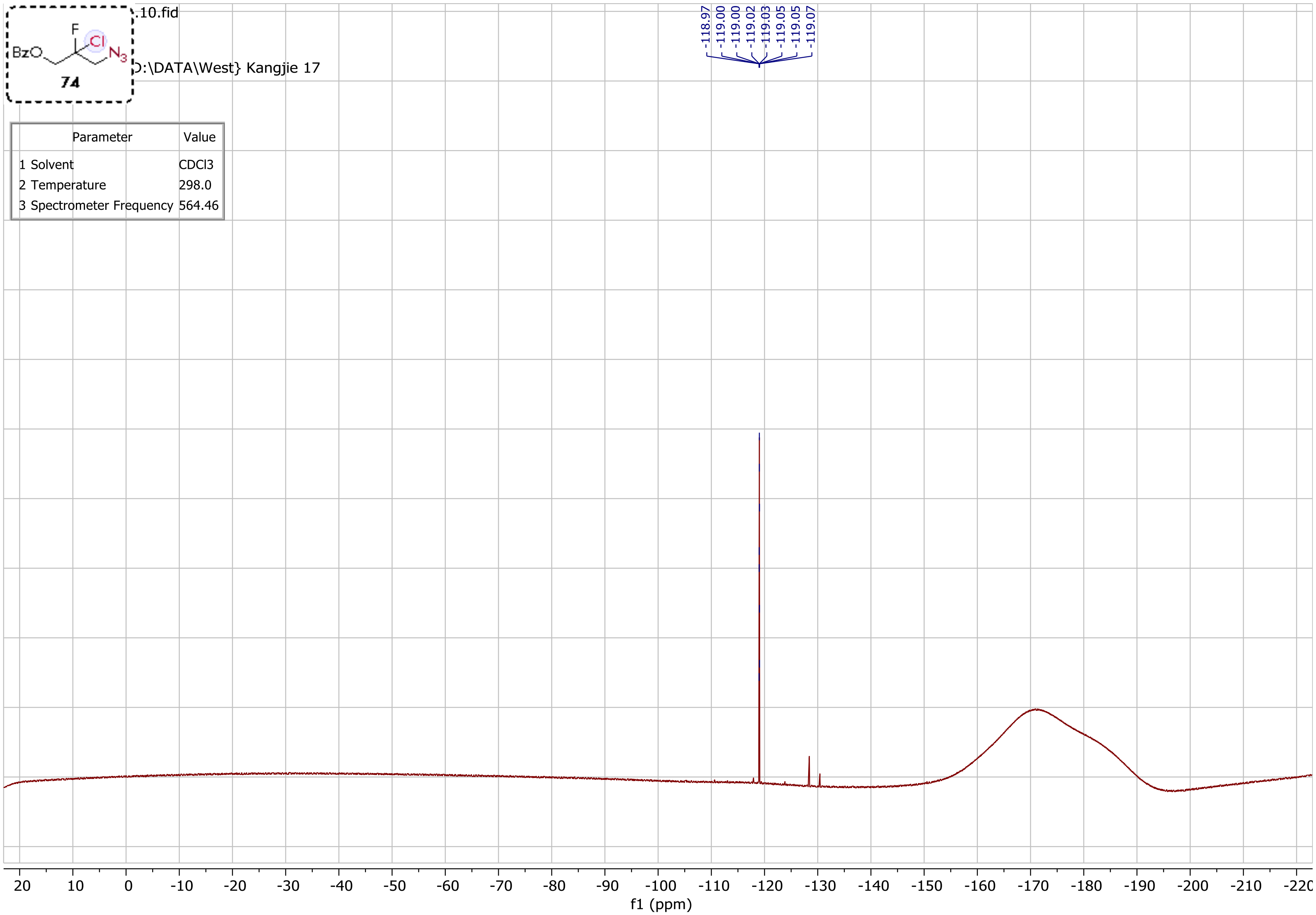
BzO
F
Cl
N3
74
.10.fid
D:\DATA\West} Kangjie 17
Parameter Value
1 Solvent CDCl3
2 Temperature 298.0
3 Spectrometer Frequency 564.46
-118.97
-119.00
-119.00
-119.02
-119.03
-119.05
-119.05
-119.07
20 10 0 -10 -20 -30 -40 -50 -60 -70 -80 -90 -100 -110 -120 -130 -140 -150 -160 -170 -180 -190 -200 -210 -220
f1 (ppm)

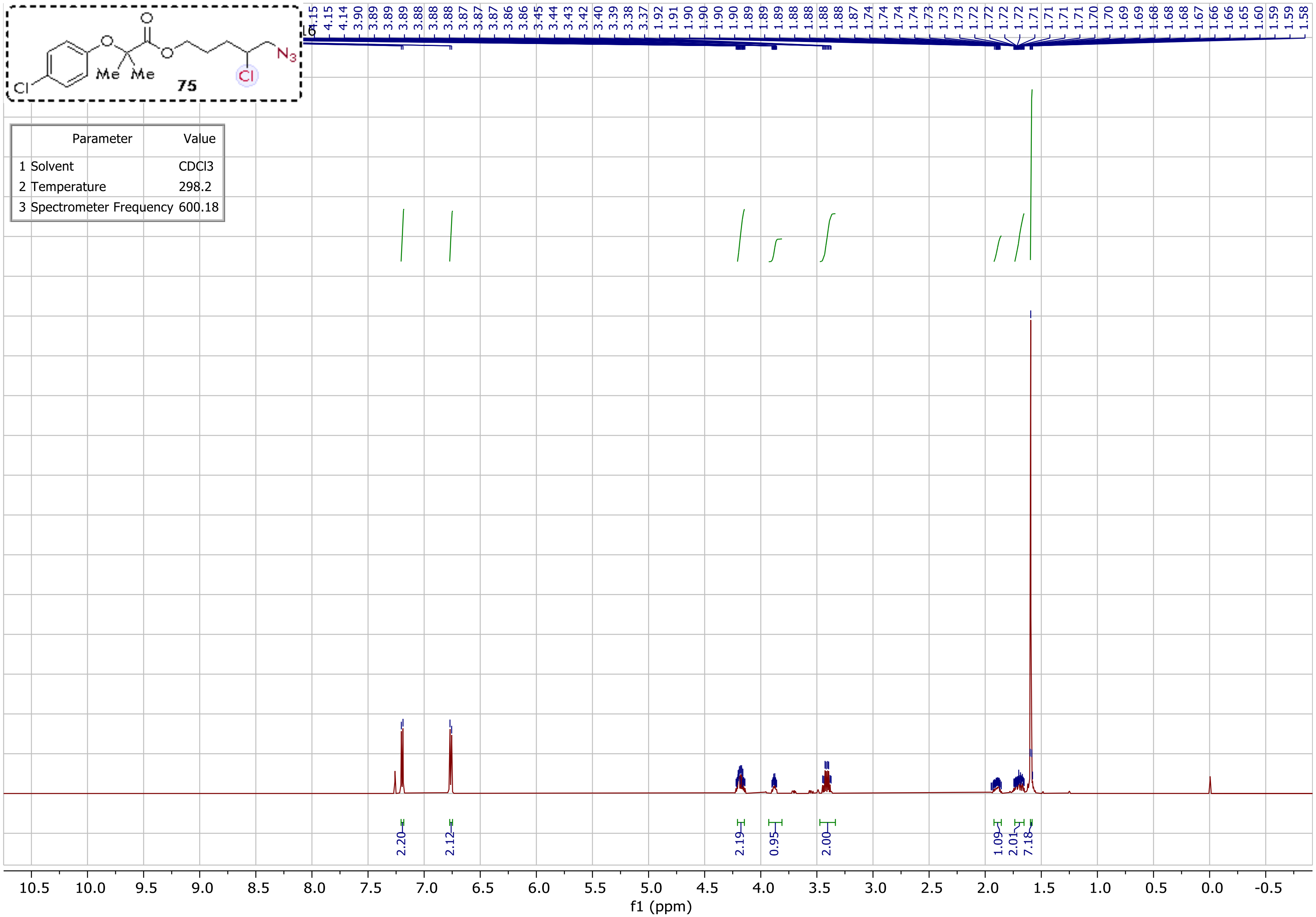

Parameter	Value
1 Solvent	CDCl3
2 Temperature	298.2
3 Spectrometer Frequency	600.18

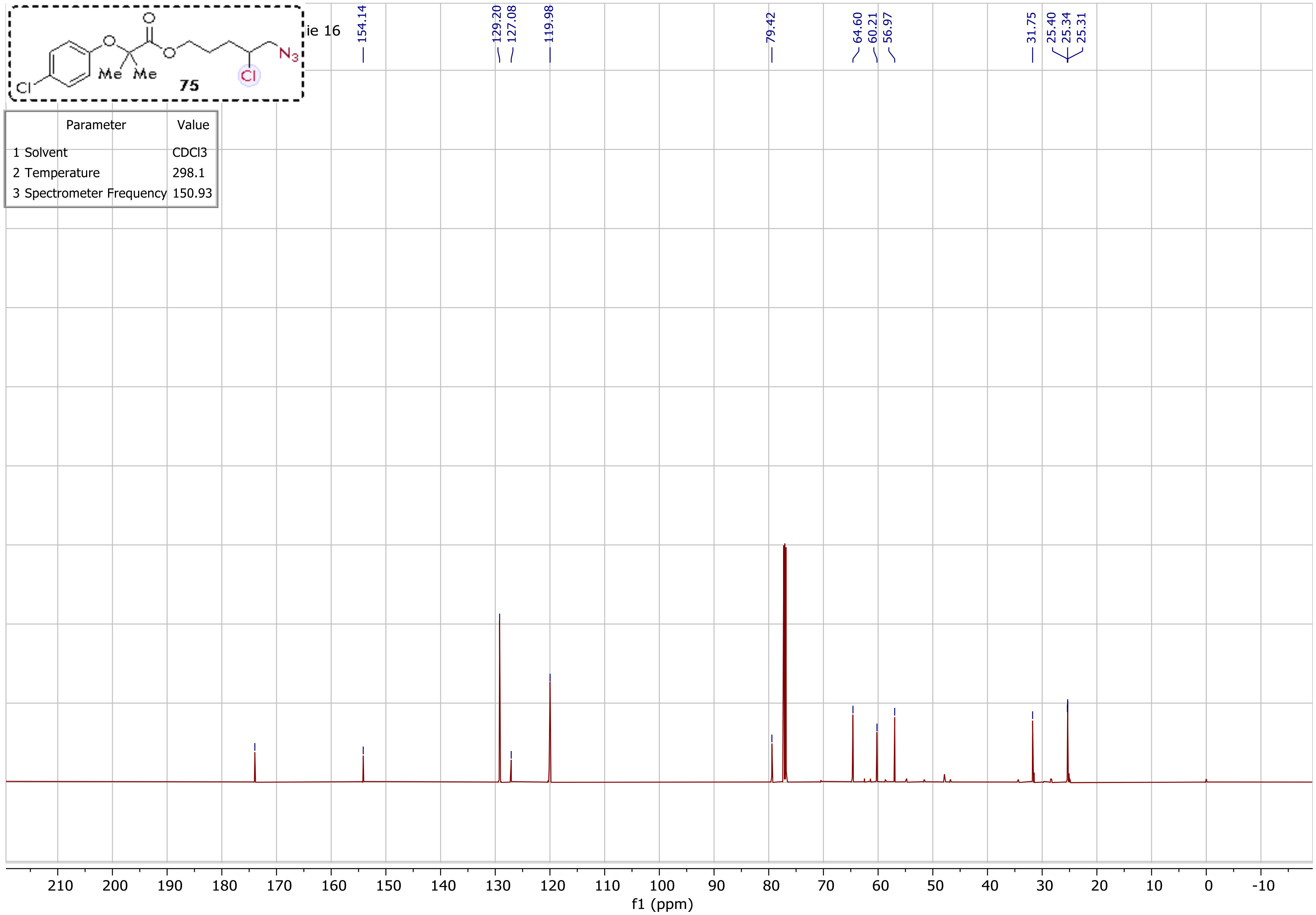

Parameter	Value
1 Solvent	CDCl3
2 Temperature	298.1
3 Spectrometer Frequency	150.93

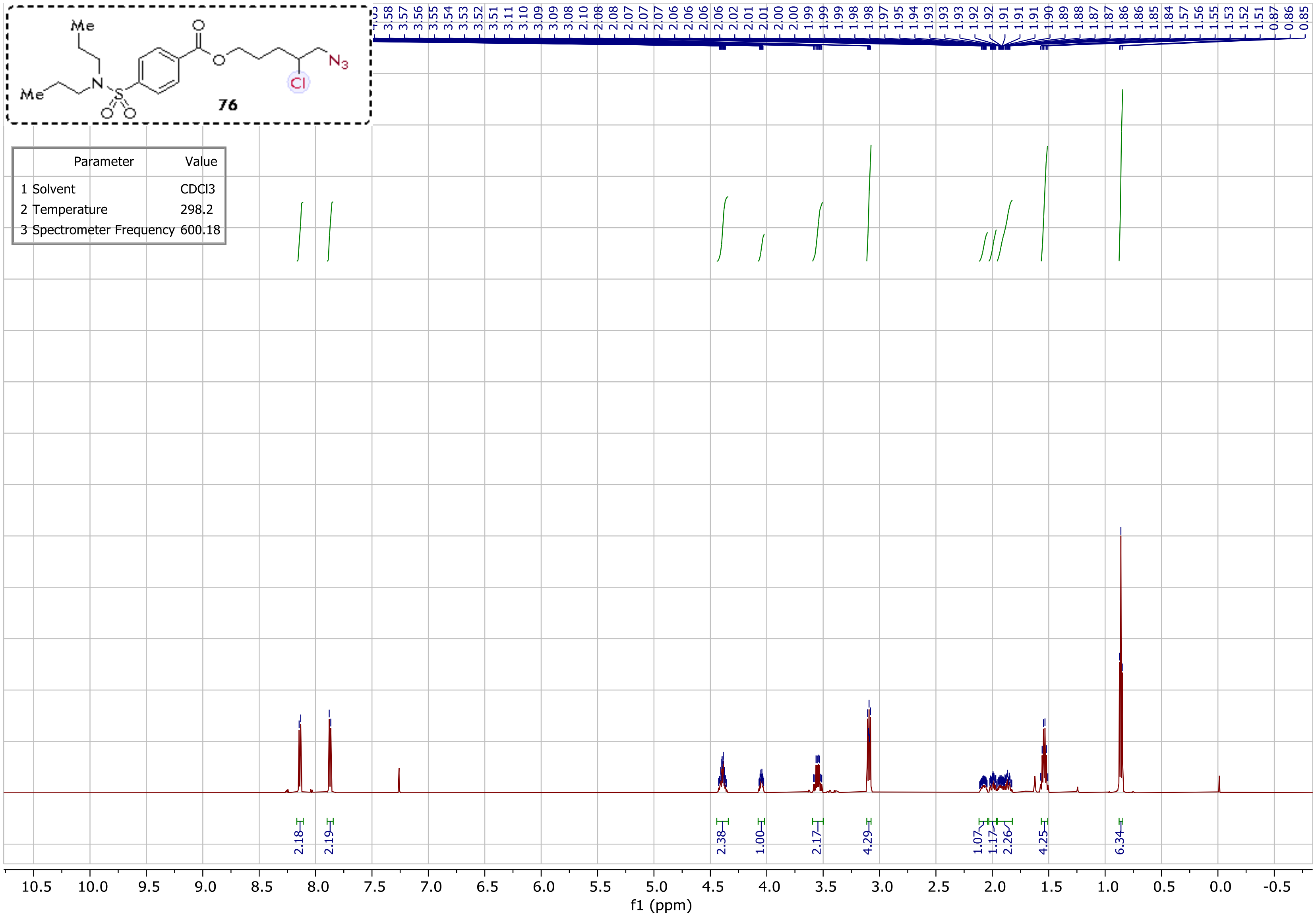

Parameter	Value
1 Solvent	CDCl3
2 Temperature	298.2
3 Spectrometer Frequency	600.18

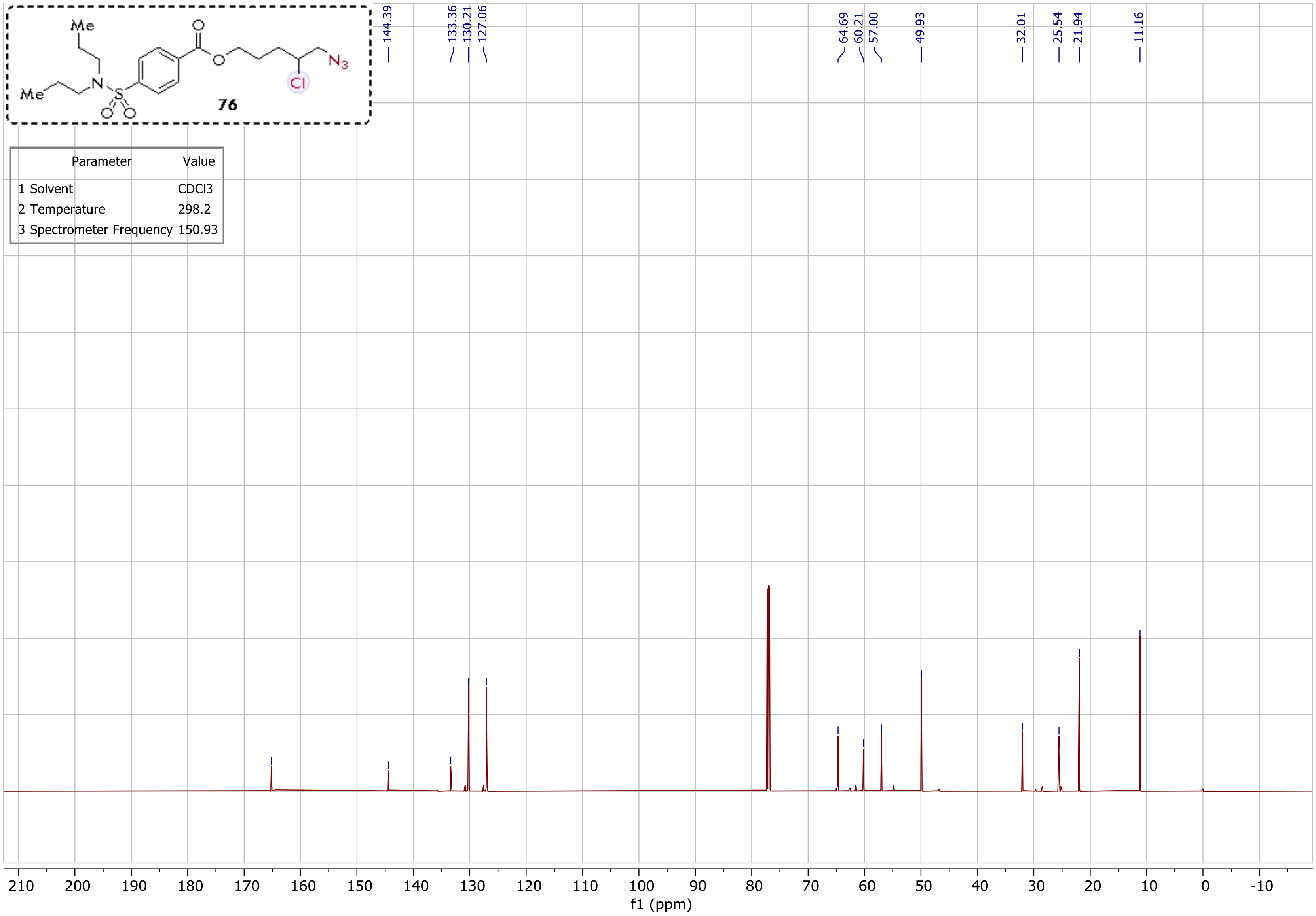

Me
Me
N
S
O
O
O
O
Cl
N3
76
Parameter Value
1 Solvent CDCl3
2 Temperature 298.2
3 Spectrometer Frequency 150.93
144.39
133.36
130.21
127.06
64.69
60.21
57.00
49.93
32.01
25.54
21.94
11.16
210 200 190 180 170 160 150 140 130 120 110 100 90 80 70 60 50 40 30 20 10 0 -10
f1 (ppm)

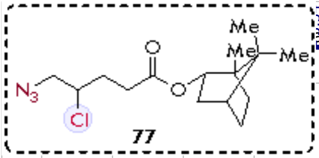

Parameter	Value
1 Solvent	CDCl3
2 Temperature	298.1
3 Spectrometer Frequency	600.18

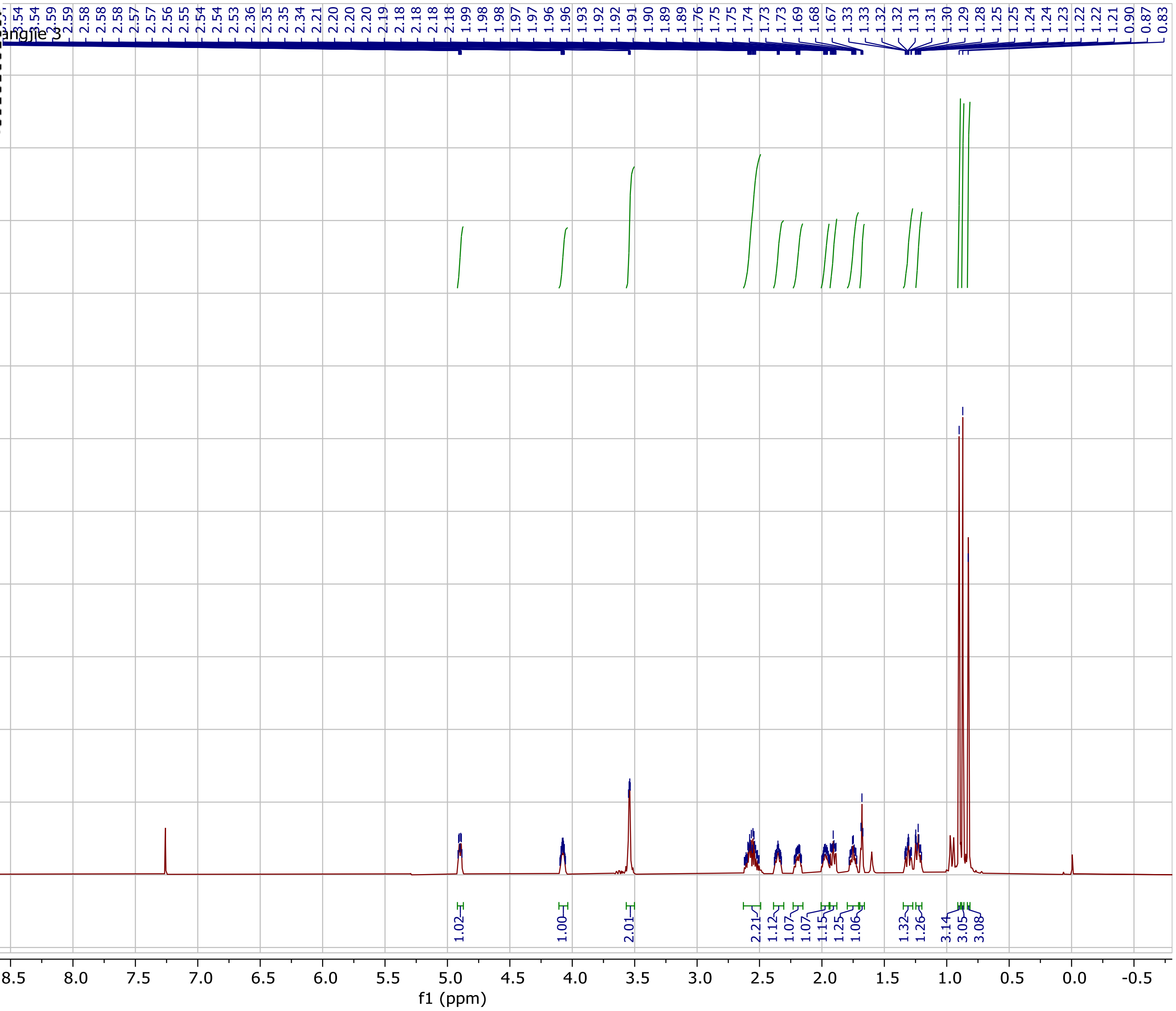

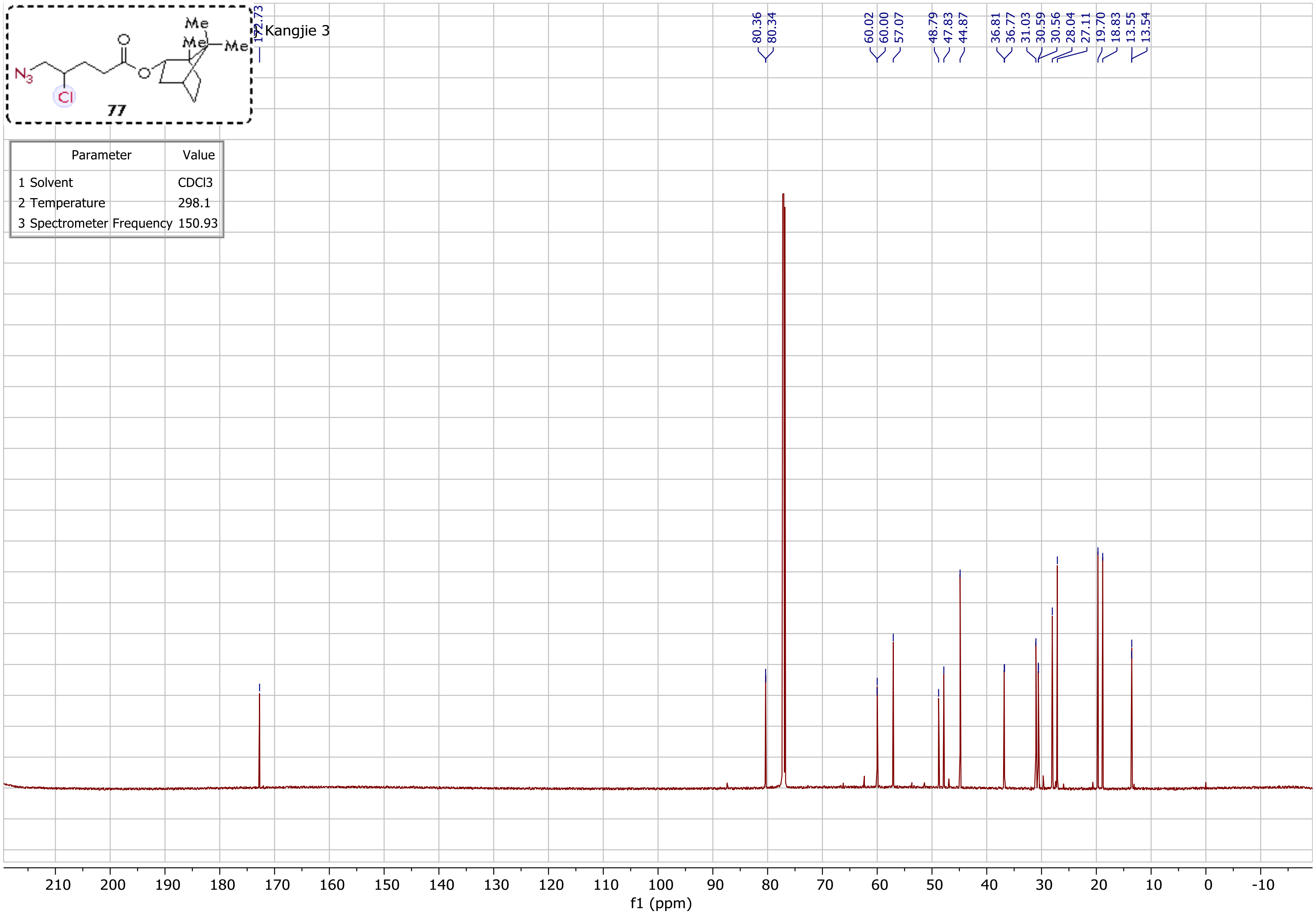

	Parameter	Value
1	Solvent	CDCl3
2	Temperature	298.1
3	Spectrometer Frequency	150.93

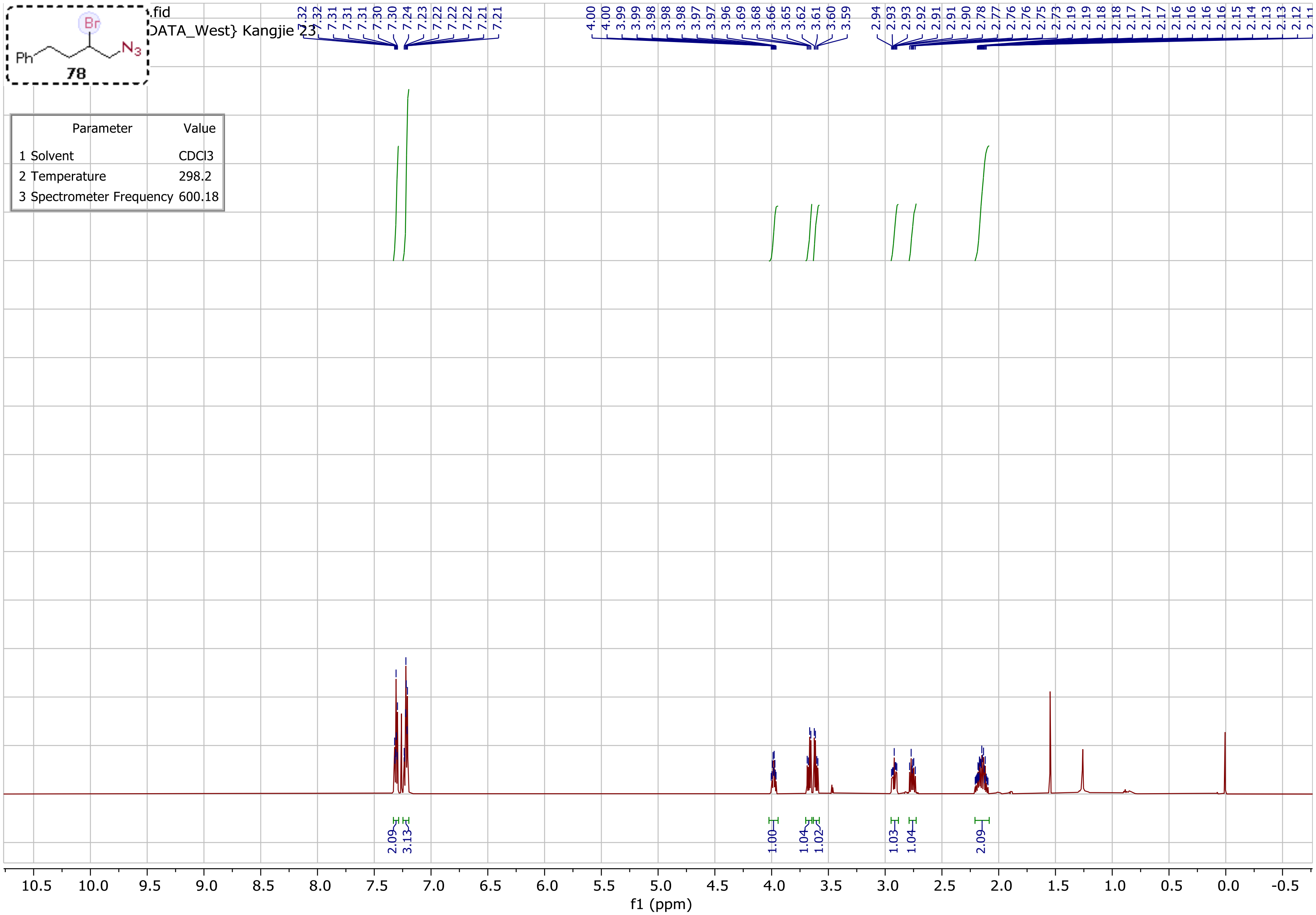

78
Ph
Br
N3
.fid
DATA_West} Kangjie 23
Parameter Value
1 Solvent CDCl3
2 Temperature 298.2
3 Spectrometer Frequency 600.18
7.32 7.32 7.31 7.31 7.31 7.30 7.24 7.23 7.22 7.22 7.22 7.21 7.21
4.00 4.00 3.99 3.99 3.98 3.98 3.97 3.97 3.96 3.69 3.68 3.66 3.65 3.62 3.61 3.60 3.59
2.94 2.93 2.93 2.92 2.91 2.90 2.78 2.77 2.76 2.76 2.75 2.73 2.19 2.19 2.18 2.18 2.17 2.17 2.17 2.16 2.16 2.16 2.15 2.14 2.13 2.12
2.09 3.13 1.00 1.04 1.02 1.03 1.04 2.09
10.5 10.0 9.5 9.0 8.5 8.0 7.5 7.0 6.5 6.0 5.5 5.0 4.5 4.0 3.5 3.0 2.5 2.0 1.5 1.0 0.5 0.0 -0.5
f1 (ppm)

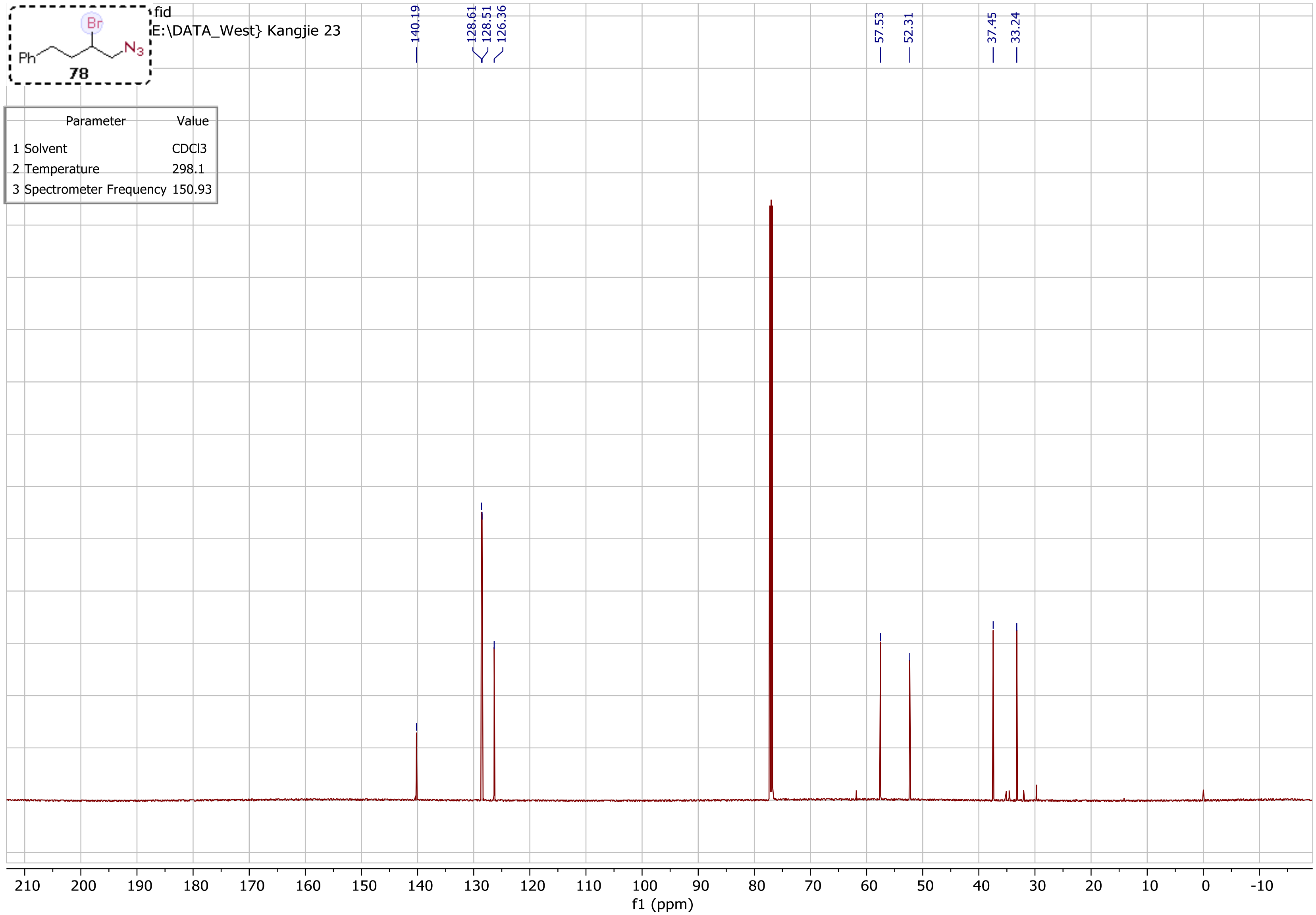

Br
Ph
N3
78
fid
E:\DATA_West} Kangjie 23
Parameter Value
1 Solvent CDCl3
2 Temperature 298.1
3 Spectrometer Frequency 150.93
140.19
128.61
128.51
126.36
57.53
52.31
37.45
33.24
210 200 190 180 170 160 150 140 130 120 110 100 90 80 70 60 50 40 30 20 10 0 -10
f1 (ppm)

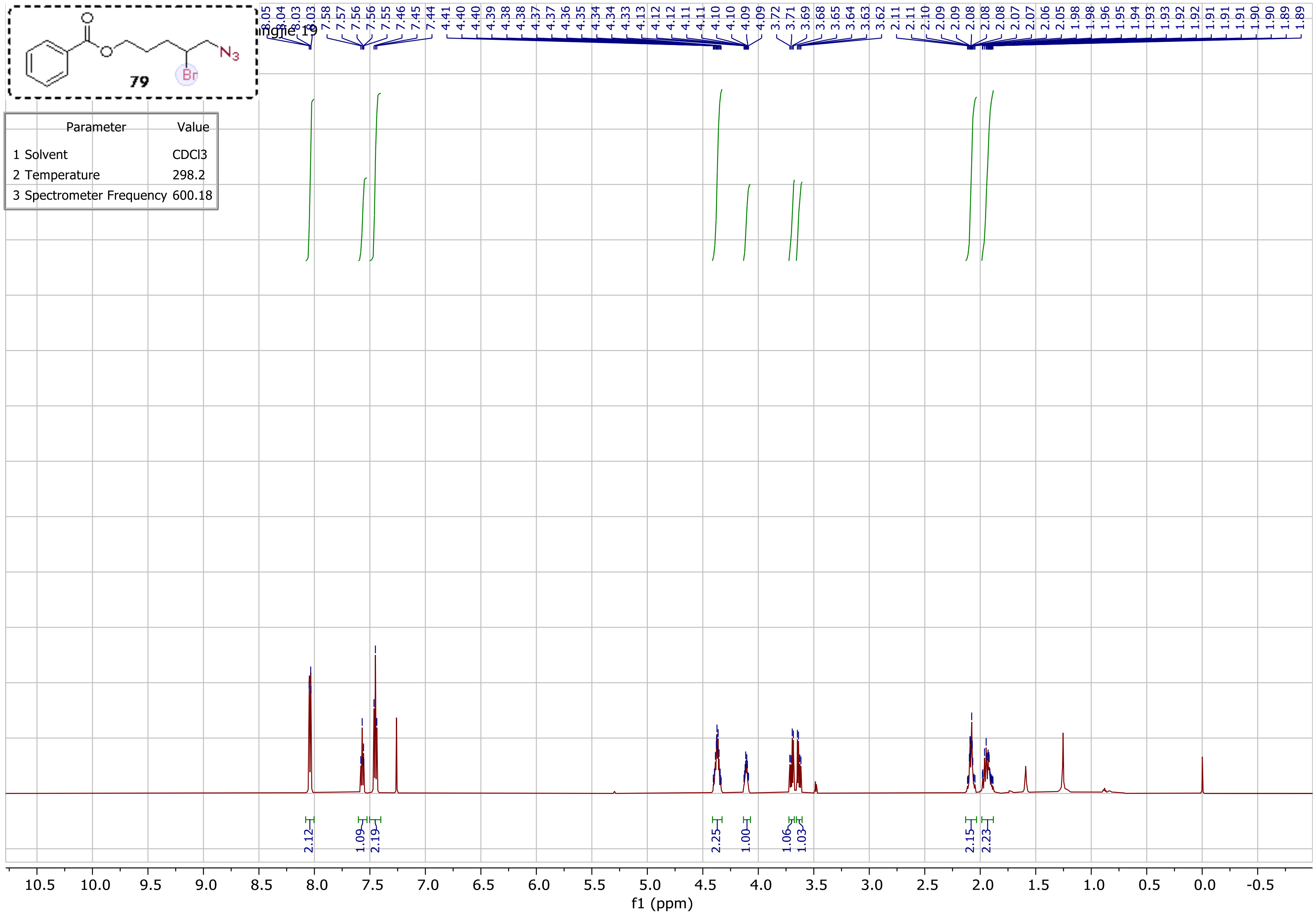

Parameter	Value
1 Solvent	CDCl3
2 Temperature	298.2
3 Spectrometer Frequency	600.18

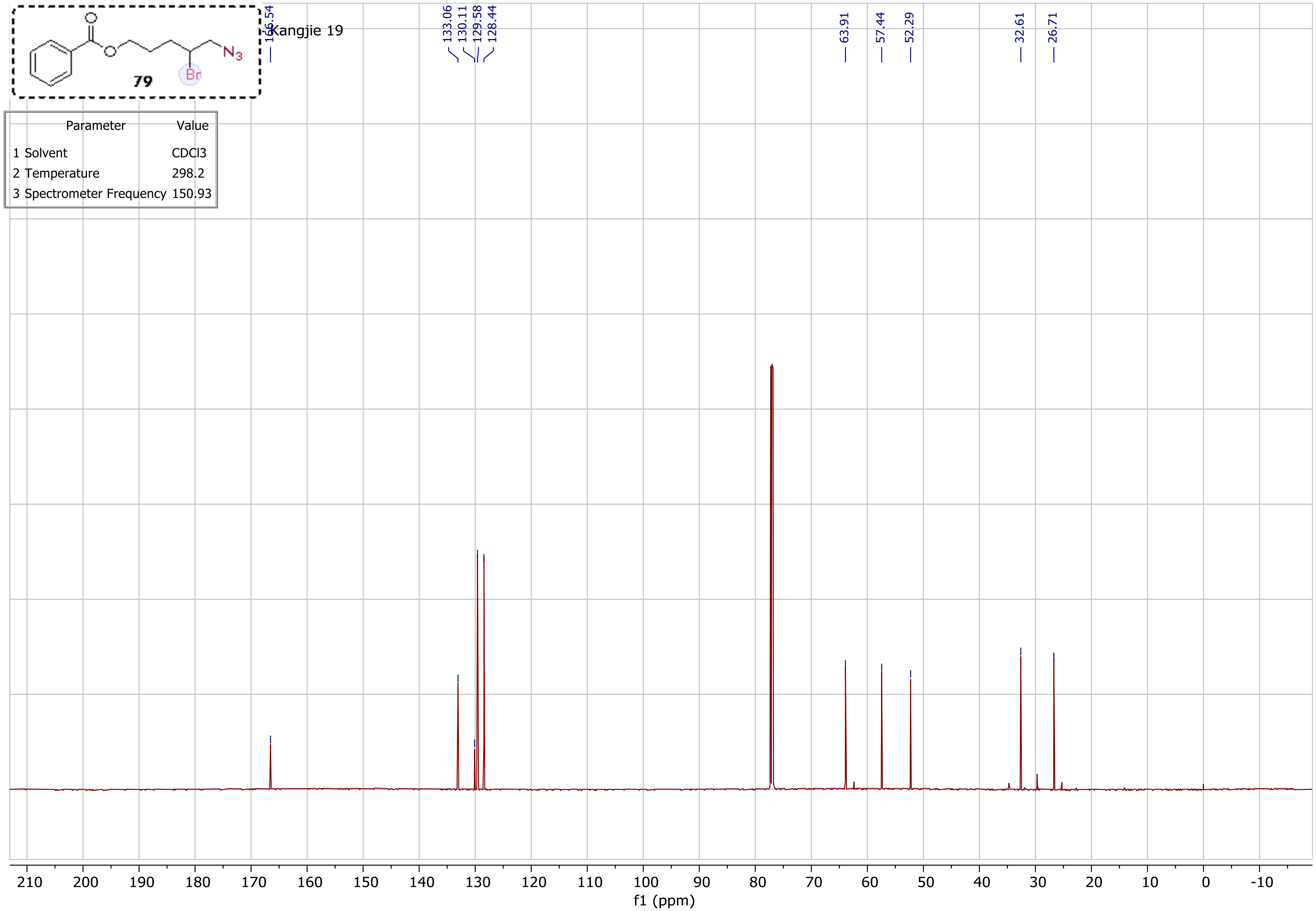

Parameter	Value
1 Solvent	CDCl3
2 Temperature	298.2
3 Spectrometer Frequency	150.93

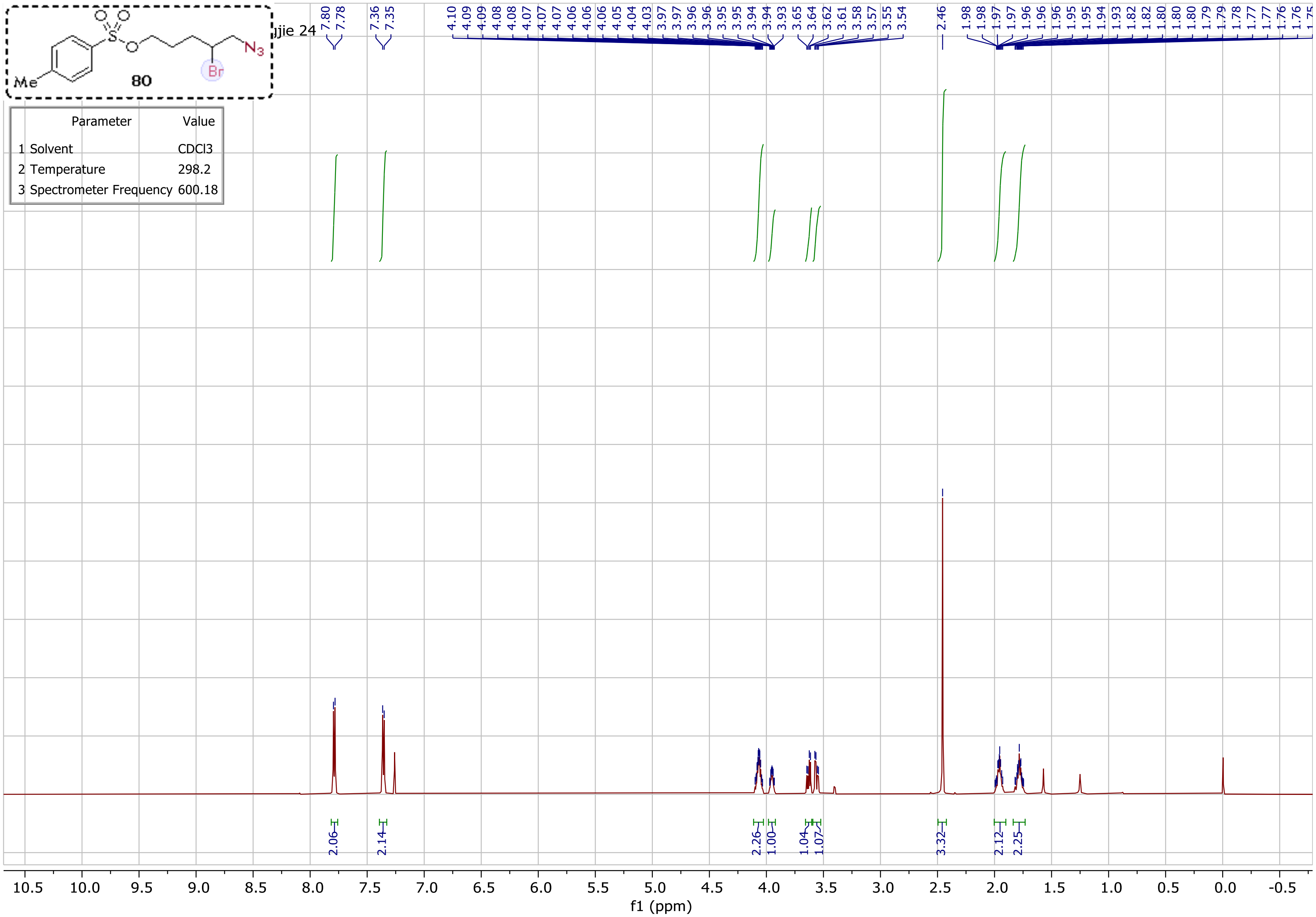
80
Me
Br
N3
Parameter Value
1 Solvent CDCl3
2 Temperature 298.2
3 Spectrometer Frequency 600.18
f1 (ppm)

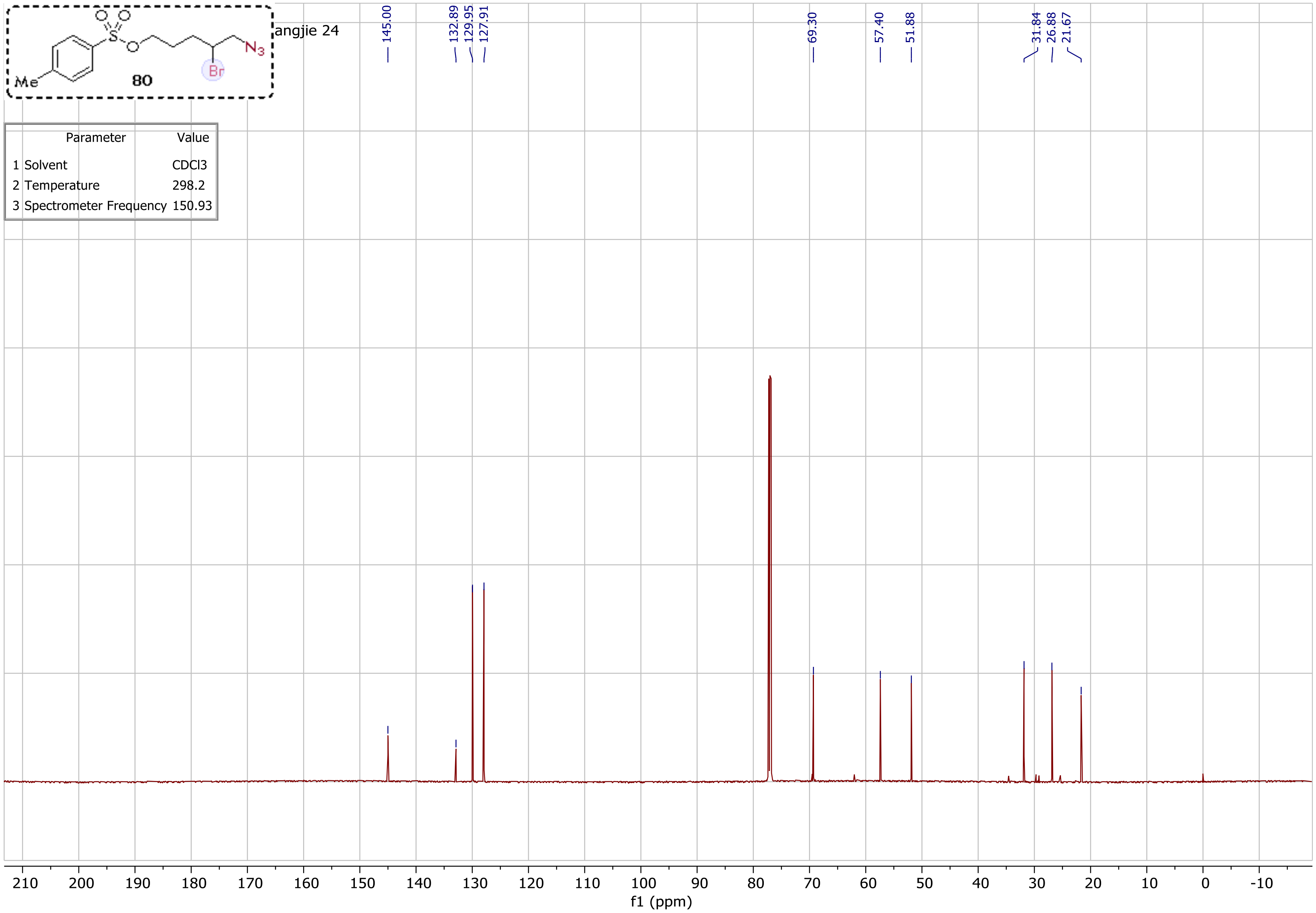

	Parameter	Value
1	Solvent	CDCl3
2	Temperature	298.2
3	Spectrometer Frequency	150.93

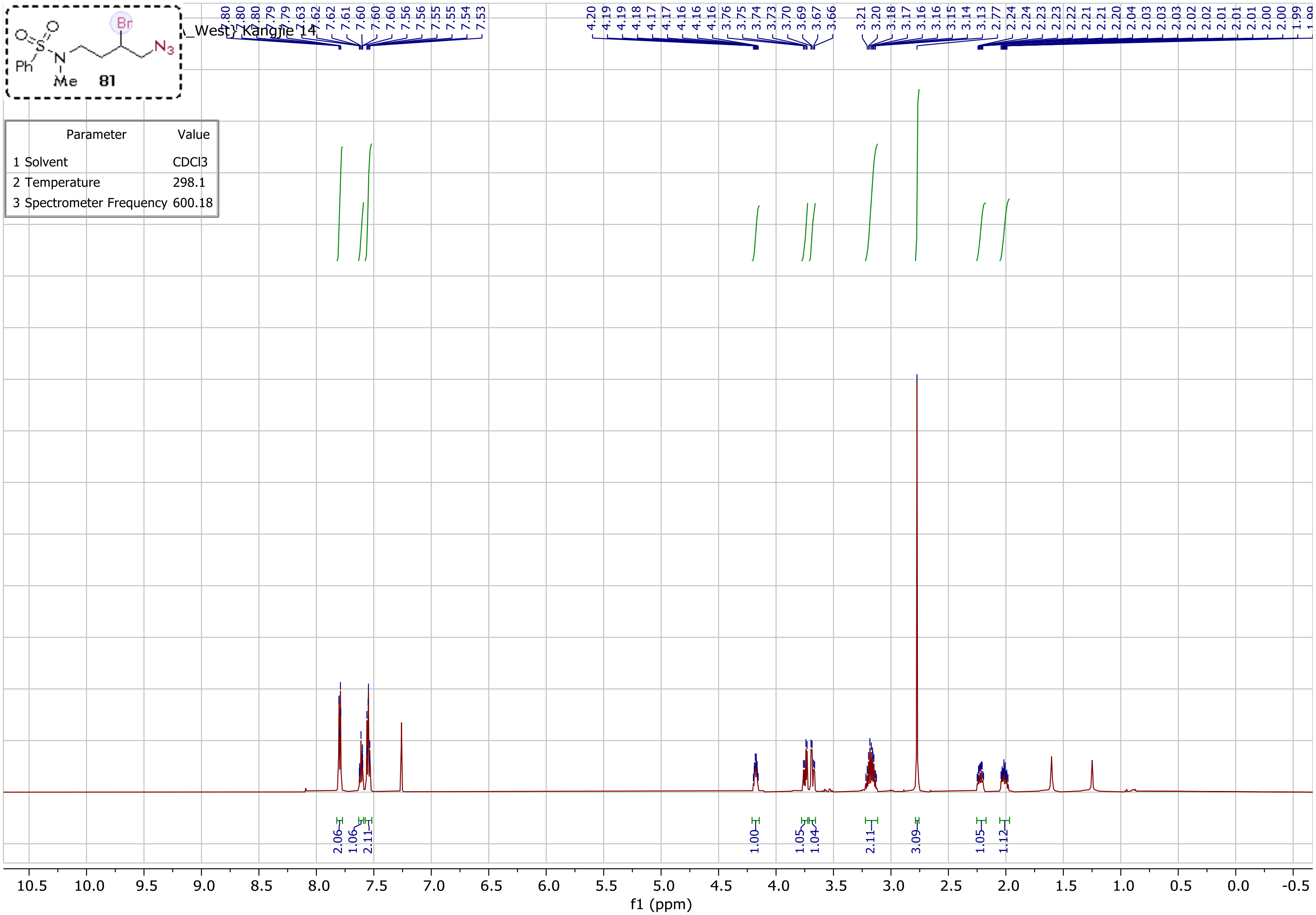
O=S(=O)(Ph)N(Me)CH2CH2CH(Br)CH2N3
81
_West} Kangjie 14
Parameter Value
1 Solvent CDCl3
2 Temperature 298.1
3 Spectrometer Frequency 600.18
2.06
1.06
2.11
1.00
1.05
1.04
2.11
3.09
1.05
1.12
f1 (ppm)

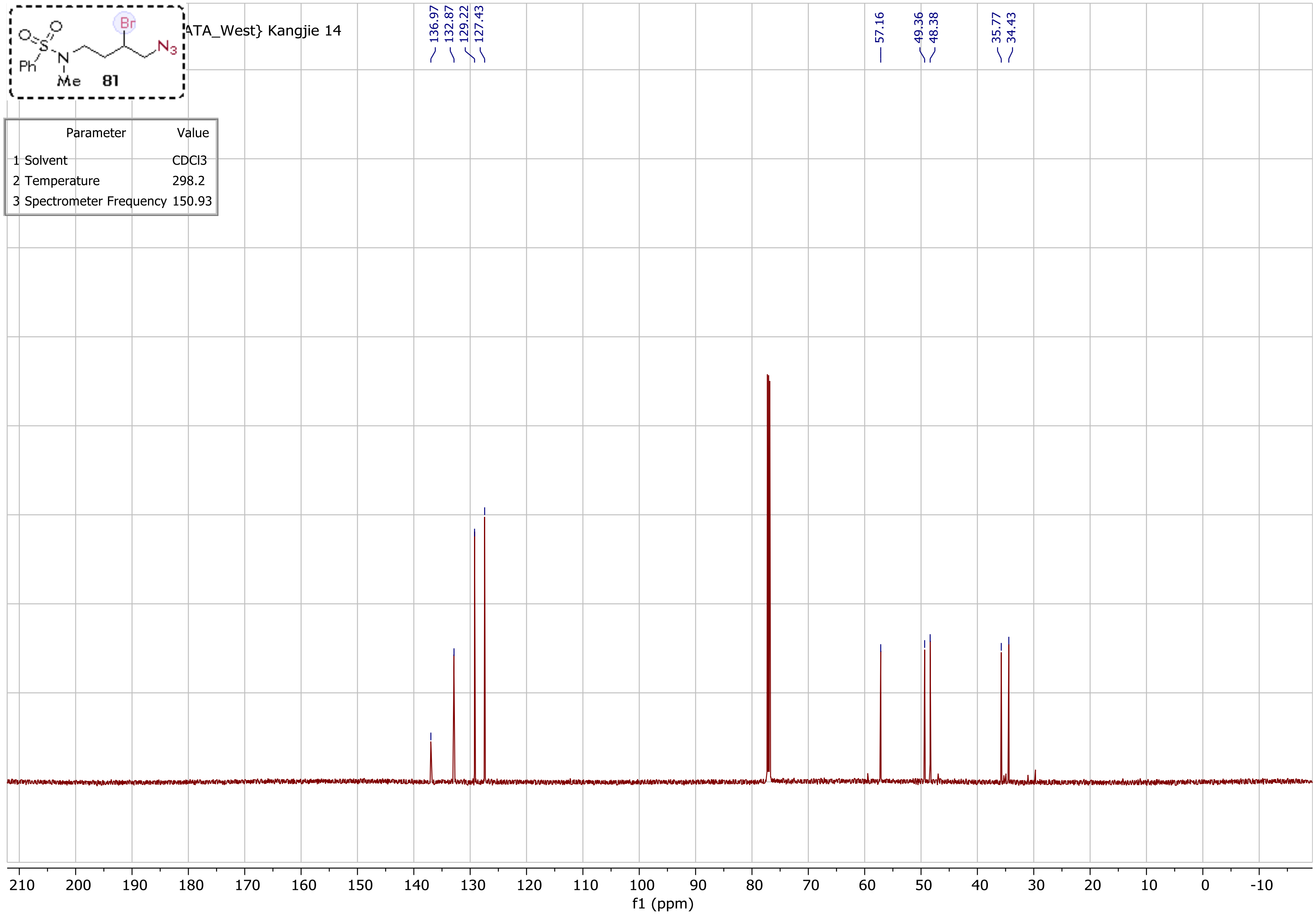
ATA_West} Kangjie 14
81
Parameter Value
1 Solvent CDCl3
2 Temperature 298.2
3 Spectrometer Frequency 150.93
136.97
132.87
129.22
127.43
57.16
49.36
48.38
35.77
34.43
210 200 190 180 170 160 150 140 130 120 110 100 90 80 70 60 50 40 30 20 10 0 -10
f1 (ppm)

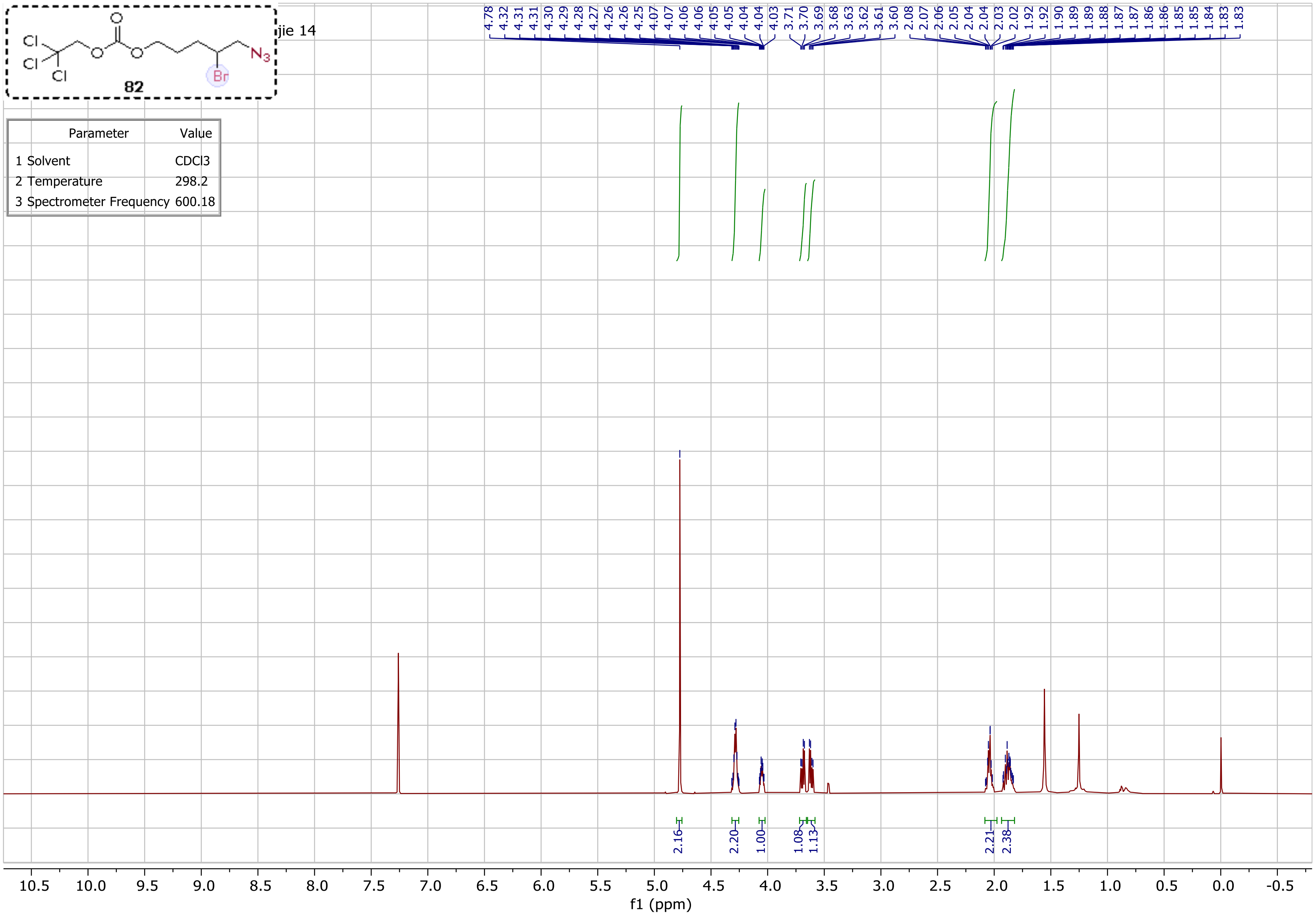

Parameter	Value
1 Solvent	CDCl3
2 Temperature	298.2
3 Spectrometer Frequency	600.18

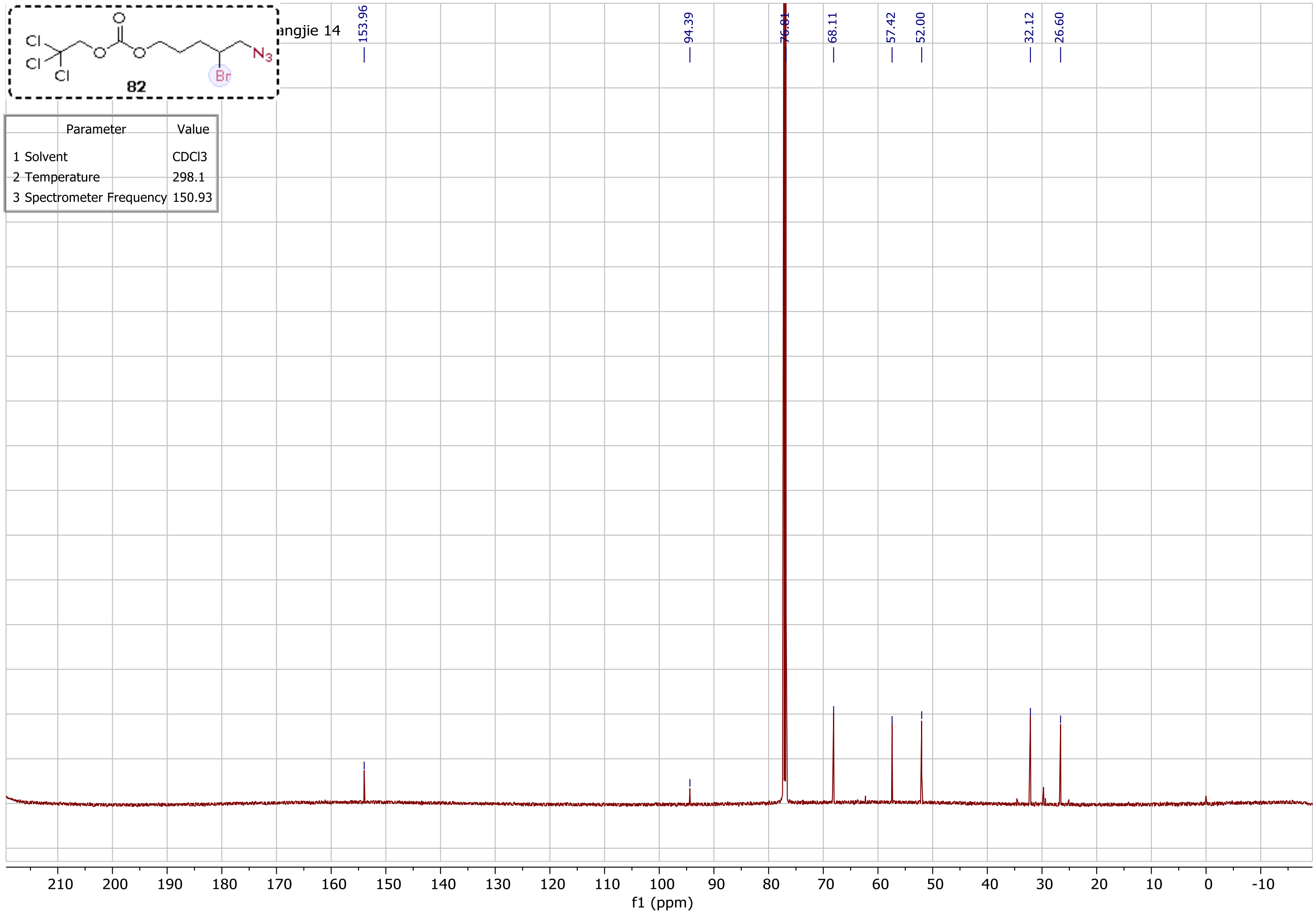

	Parameter	Value
1	Solvent	CDCl3
2	Temperature	298.1
3	Spectrometer Frequency	150.93

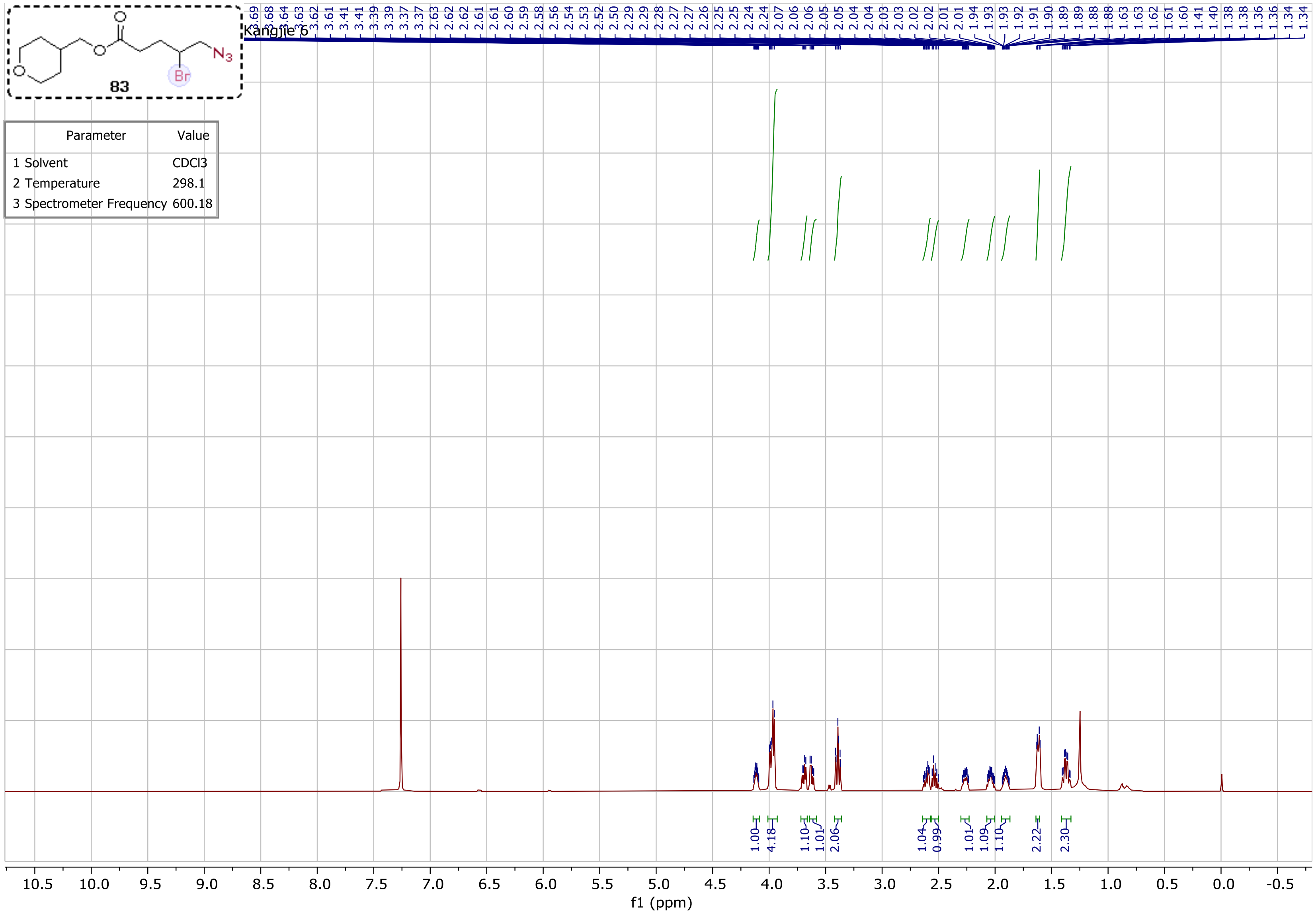
83
N3
Br
Kangjie 6
Parameter Value
1 Solvent CDCl3
2 Temperature 298.1
3 Spectrometer Frequency 600.18
3.69 3.68 3.64 3.63 3.62 3.61 3.41 3.41 3.39 3.39 3.37 3.37 2.63 2.62 2.62 2.61 2.61 2.60 2.59 2.58 2.56 2.54 2.53 2.52 2.50 2.29 2.29 2.28 2.27 2.27 2.26 2.25 2.25 2.24 2.24 2.07 2.06 2.06 2.05 2.05 2.04 2.04 2.03 2.03 2.02 2.02 2.01 2.01 1.94 1.93 1.93 1.92 1.91 1.90 1.89 1.89 1.88 1.88 1.63 1.63 1.62 1.61 1.60 1.41 1.40 1.38 1.38 1.36 1.36 1.34 1.34
1.00 4.18 1.10 1.01 2.06 1.04 0.99 1.01 1.09 1.10 2.22 2.30
10.5 10.0 9.5 9.0 8.5 8.0 7.5 7.0 6.5 6.0 5.5 5.0 4.5 4.0 3.5 3.0 2.5 2.0 1.5 1.0 0.5 0.0 -0.5
f1 (ppm)

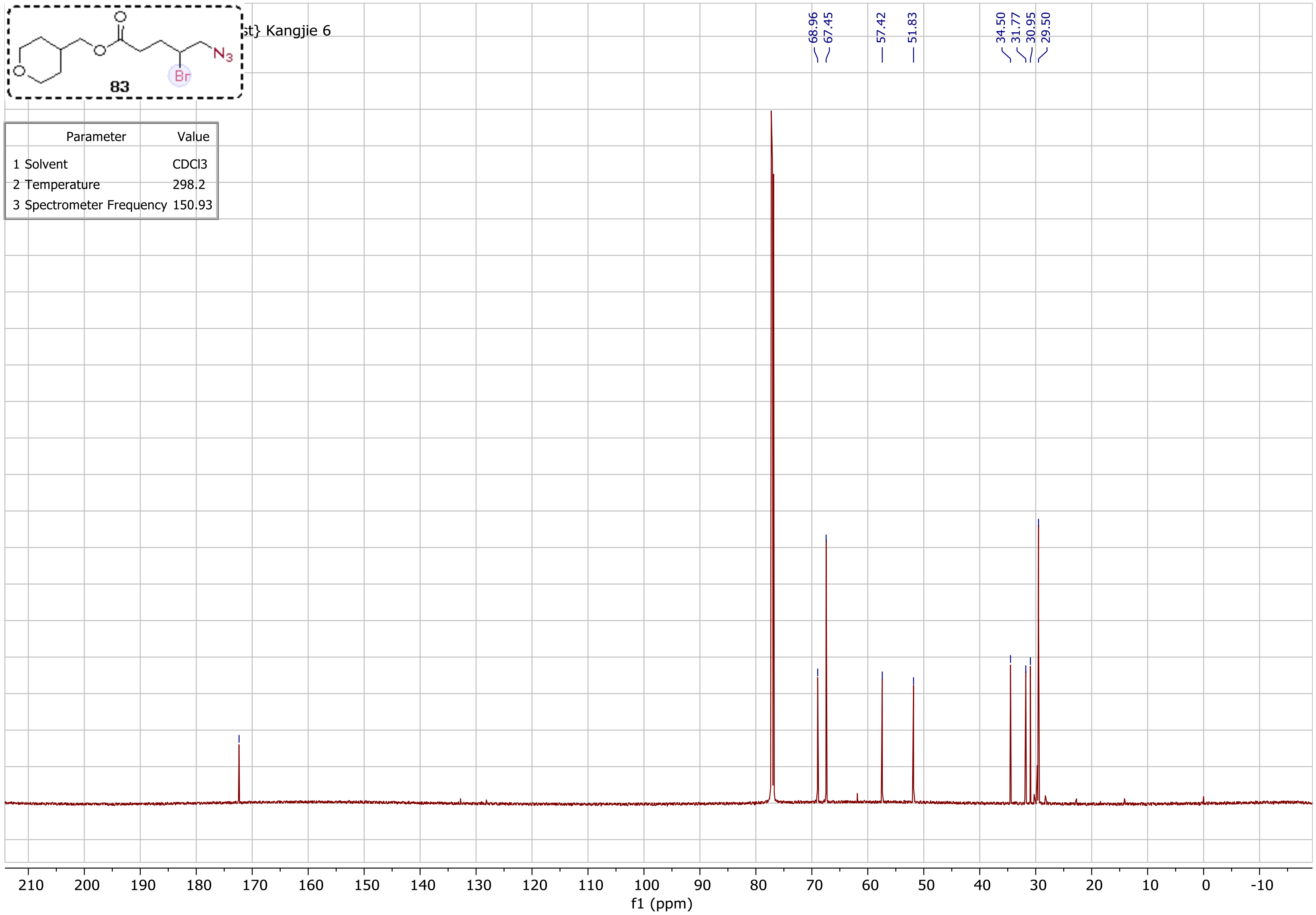

Parameter	Value
1 Solvent	CDCl3
2 Temperature	298.2
3 Spectrometer Frequency	150.93

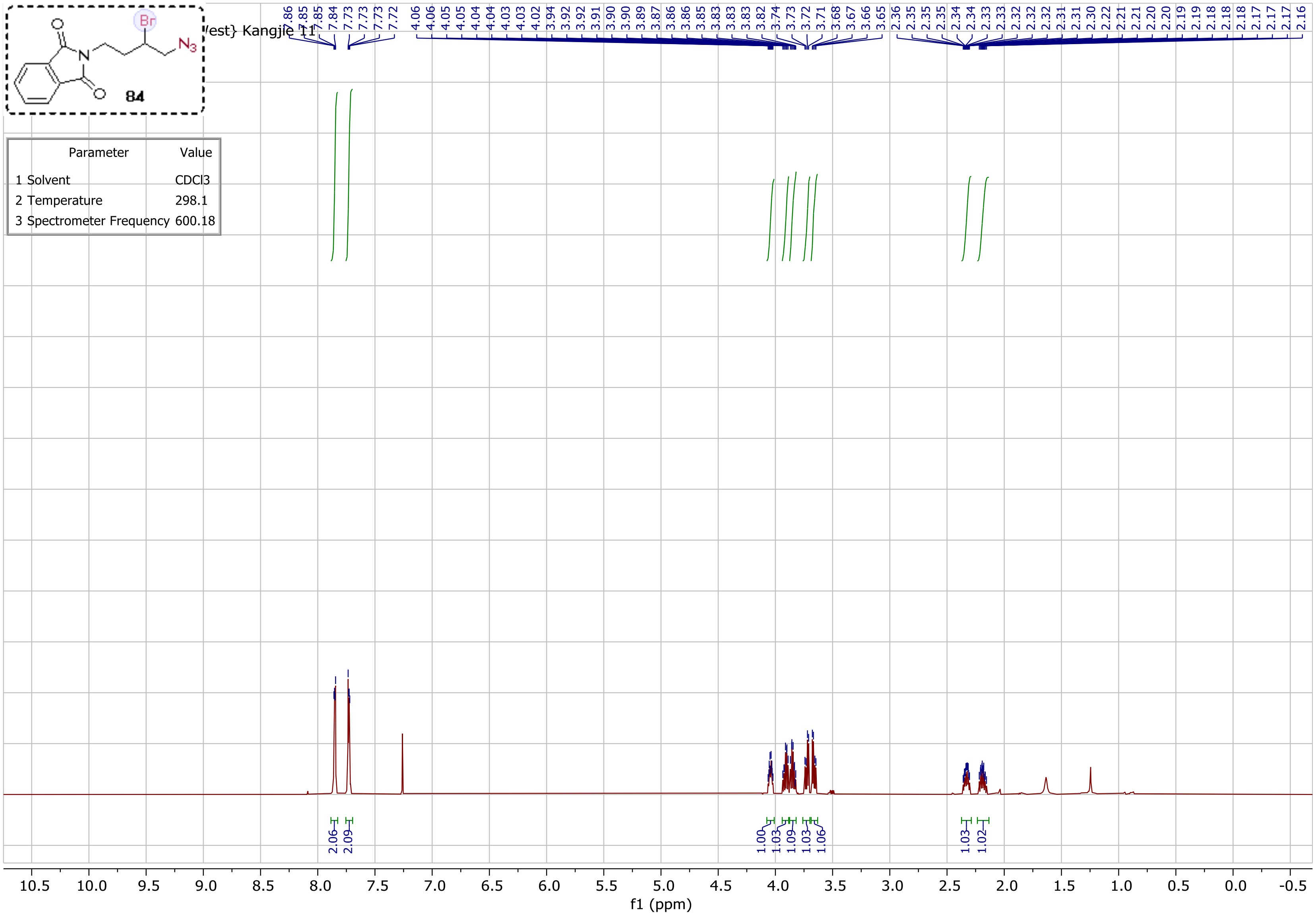

Parameter	Value
1 Solvent	CDCl3
2 Temperature	298.1
3 Spectrometer Frequency	600.18

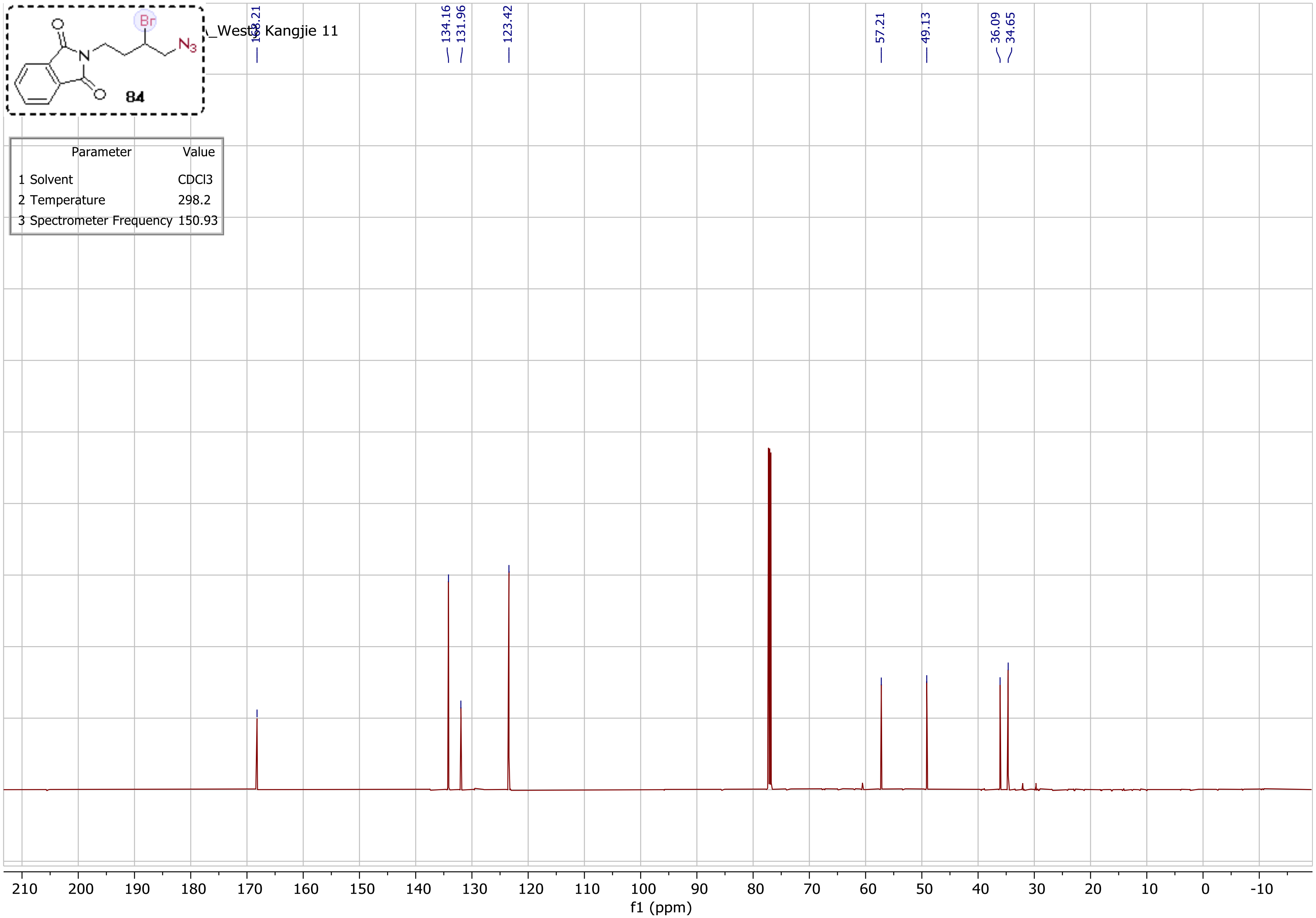

Br
N
N3
84
_West3 Kangjie 11
168.21
134.16
131.96
123.42
57.21
49.13
36.09
34.65
Parameter Value
1 Solvent CDCl3
2 Temperature 298.2
3 Spectrometer Frequency 150.93
210 200 190 180 170 160 150 140 130 120 110 100 90 80 70 60 50 40 30 20 10 0 -10
f1 (ppm)

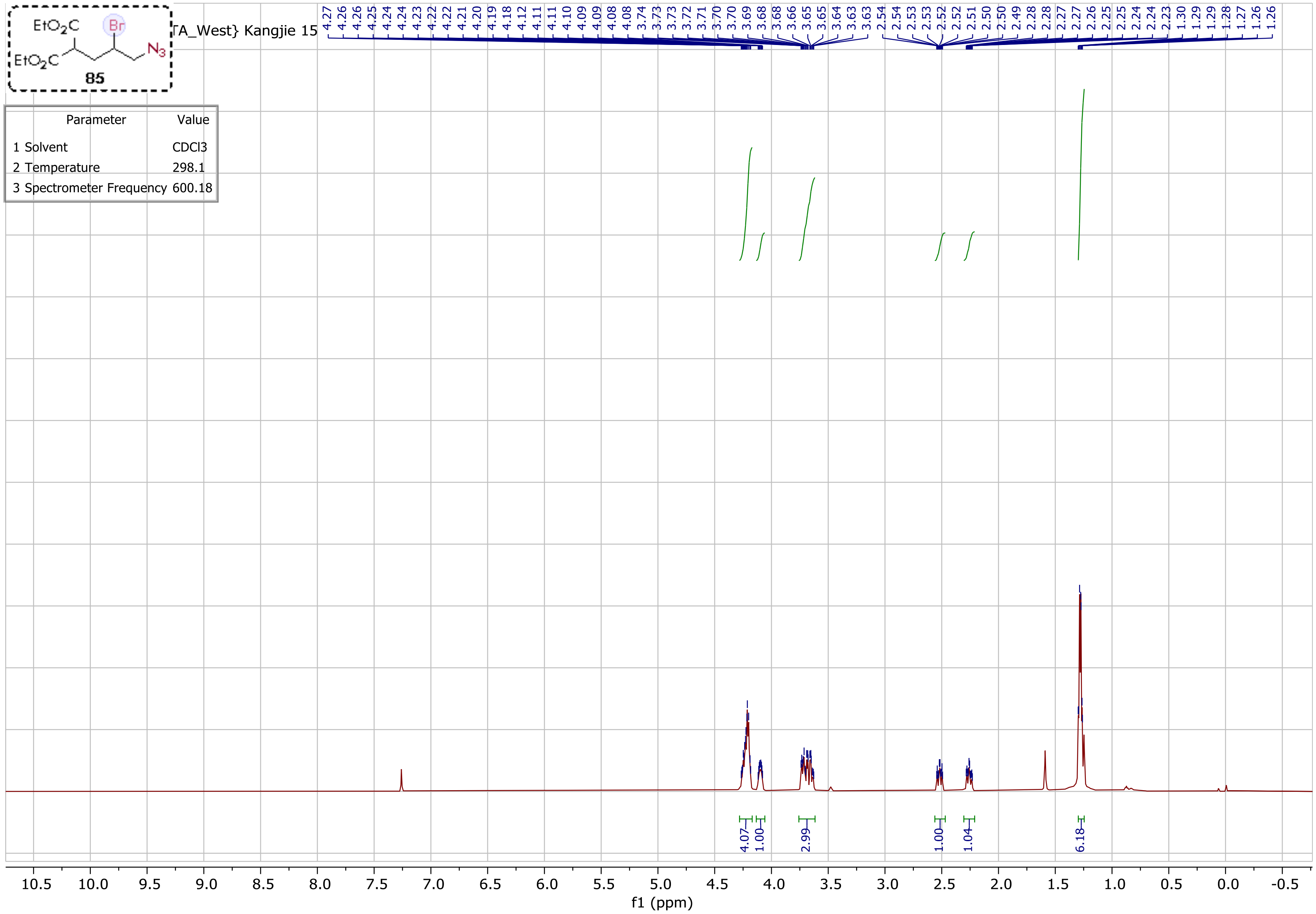

Parameter	Value
1 Solvent	CDCl3
2 Temperature	298.1
3 Spectrometer Frequency	600.18

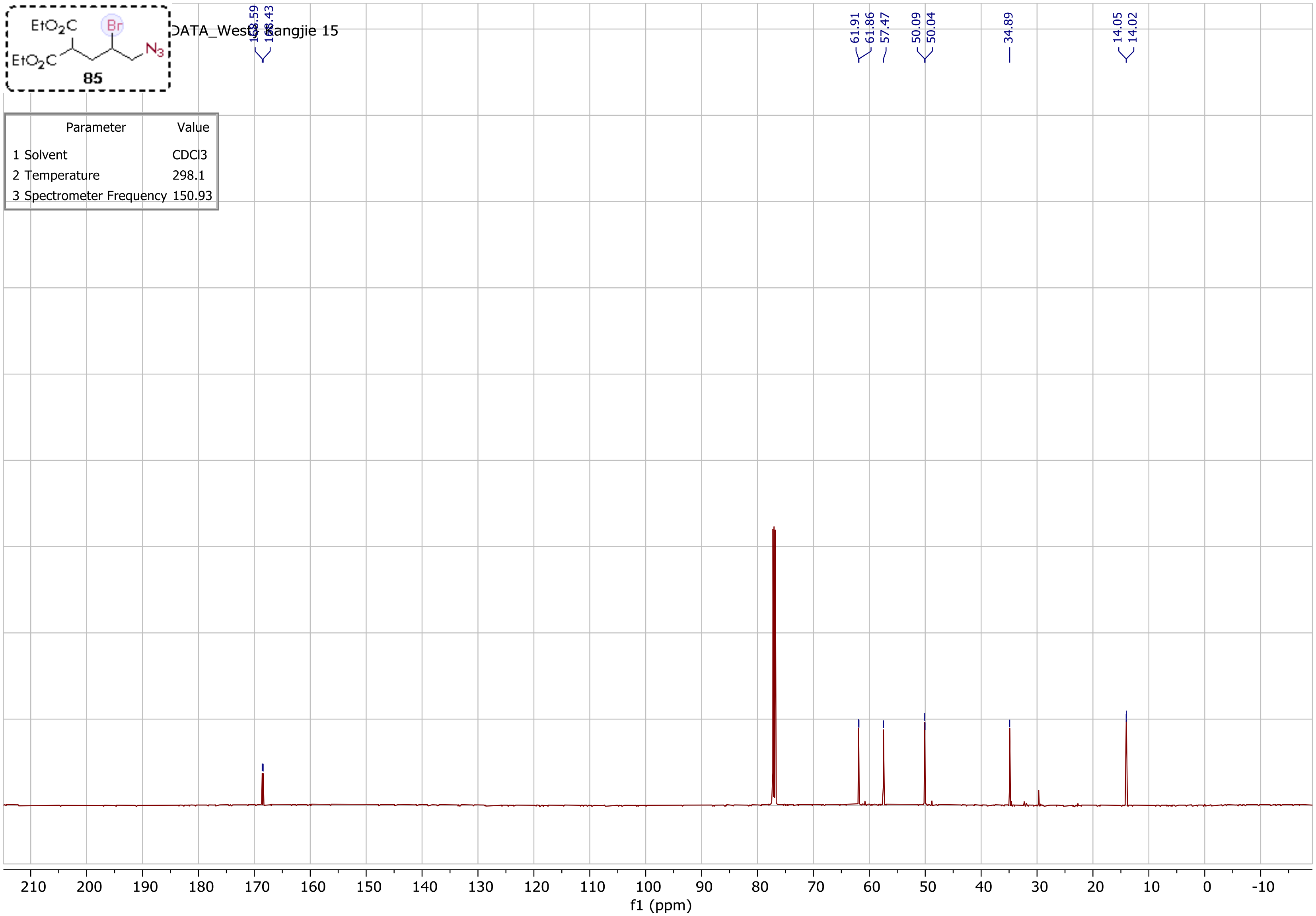
EtO2C
Br
N3
EtO2C
85
DATA_West3 Kangjie 15
168.59
168.43
61.91
61.86
57.47
50.09
50.04
34.89
14.05
14.02
Parameter Value
1 Solvent CDCl3
2 Temperature 298.1
3 Spectrometer Frequency 150.93
210 200 190 180 170 160 150 140 130 120 110 100 90 80 70 60 50 40 30 20 10 0 -10
f1 (ppm)

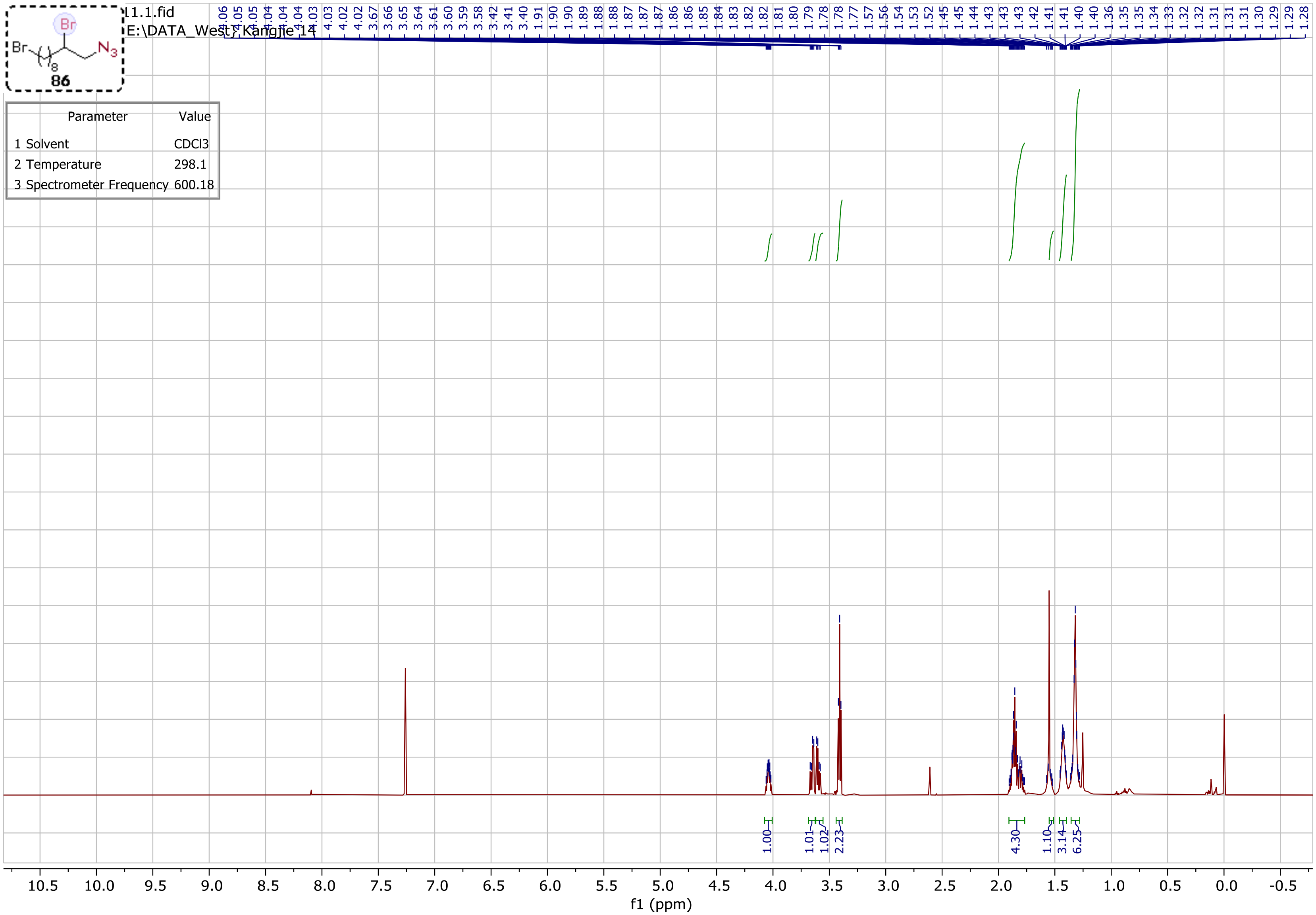
Br
Br
N3
8
86
11.1.fid
E:\DATA_West\Kangjie 14
Parameter Value
1 Solvent CDCl3
2 Temperature 298.1
3 Spectrometer Frequency 600.18
1.00
1.01
1.02
2.23
4.30
1.10
3.14
6.25
f1 (ppm)

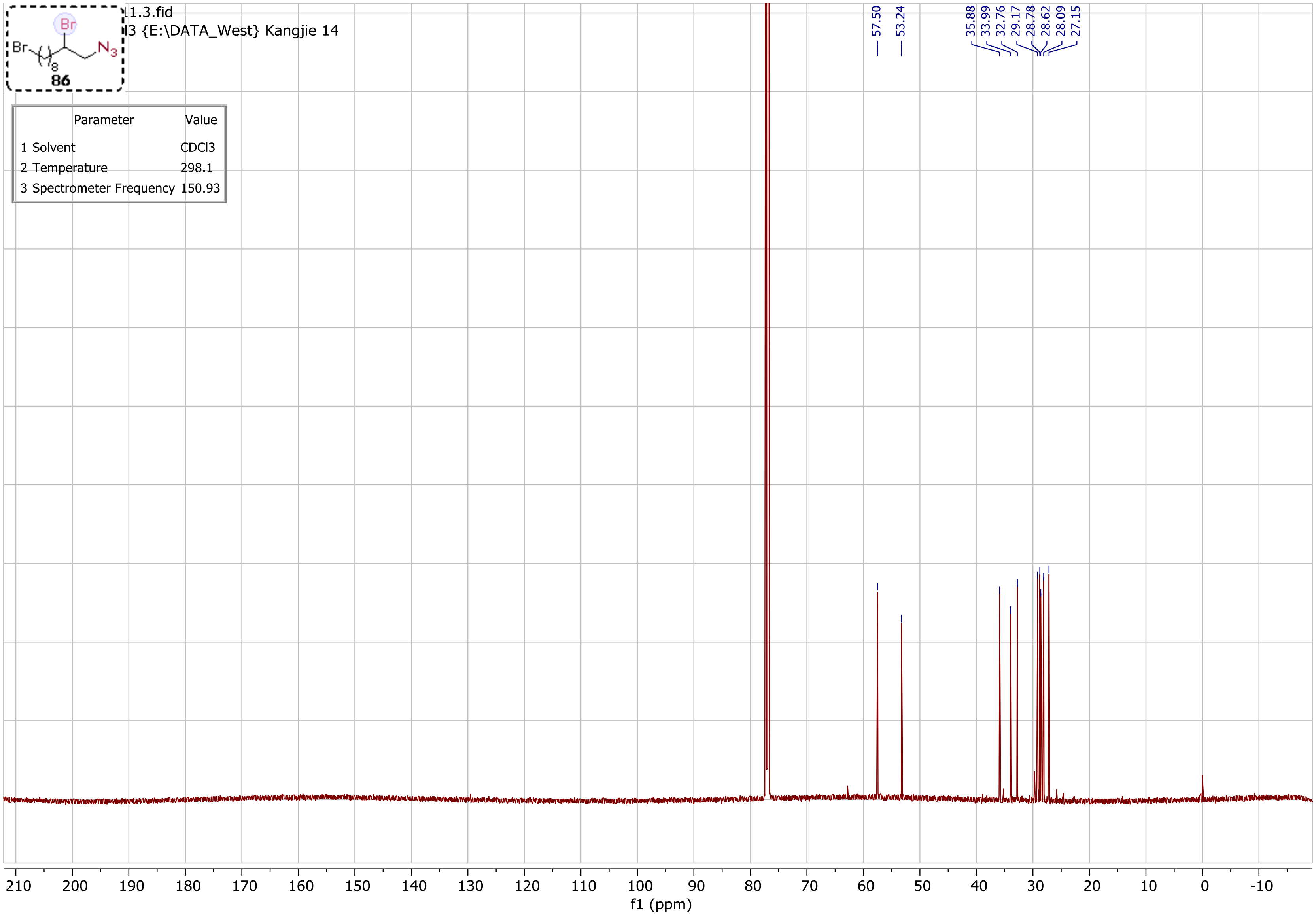

Parameter	Value
1 Solvent	CDCl3
2 Temperature	298.1
3 Spectrometer Frequency	150.93

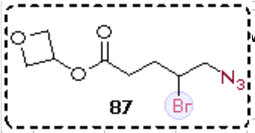

87

Parameter	Value
1 Solvent	CDCl3
2 Temperature	298.1
3 Spectrometer Frequency	600.18

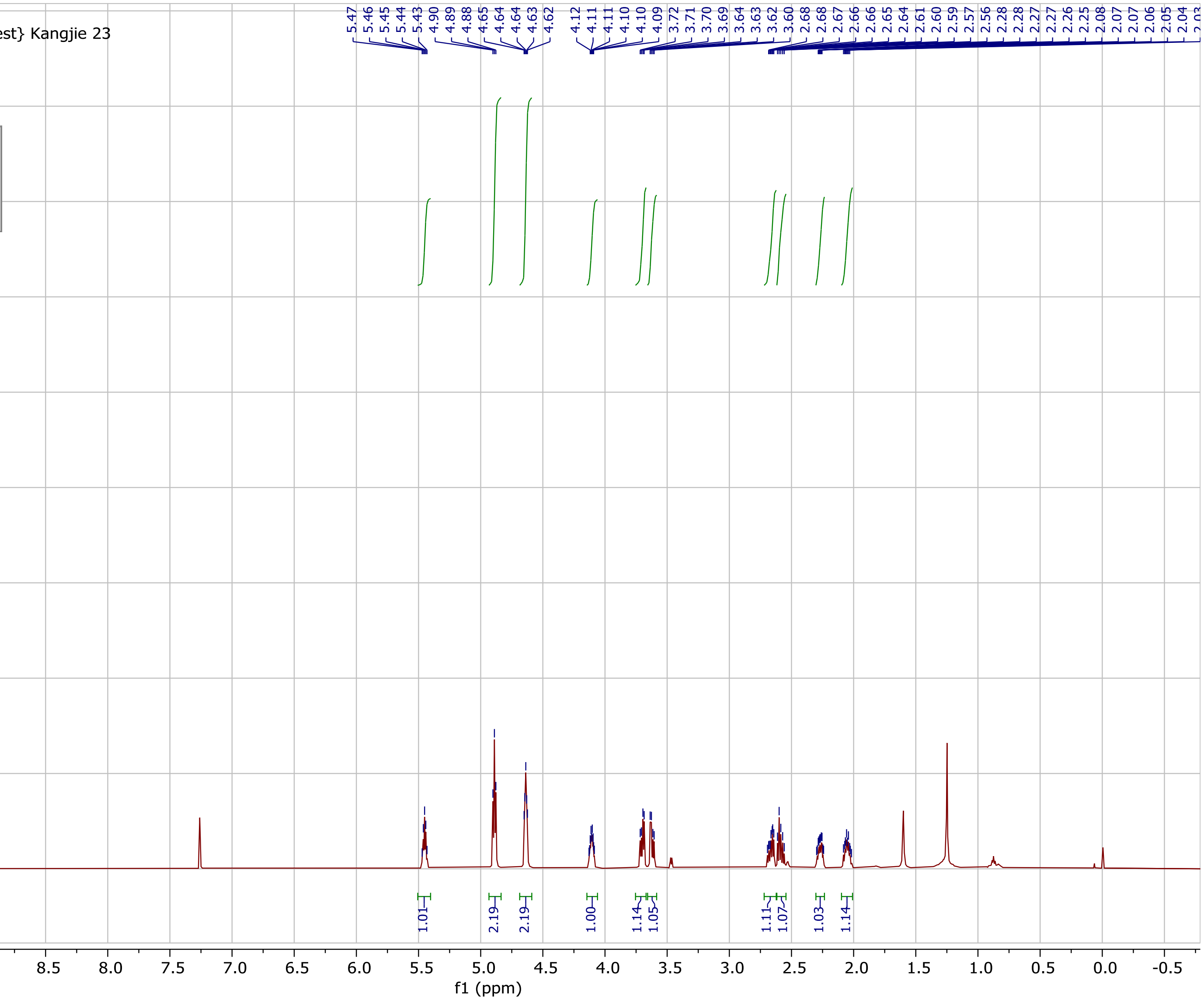

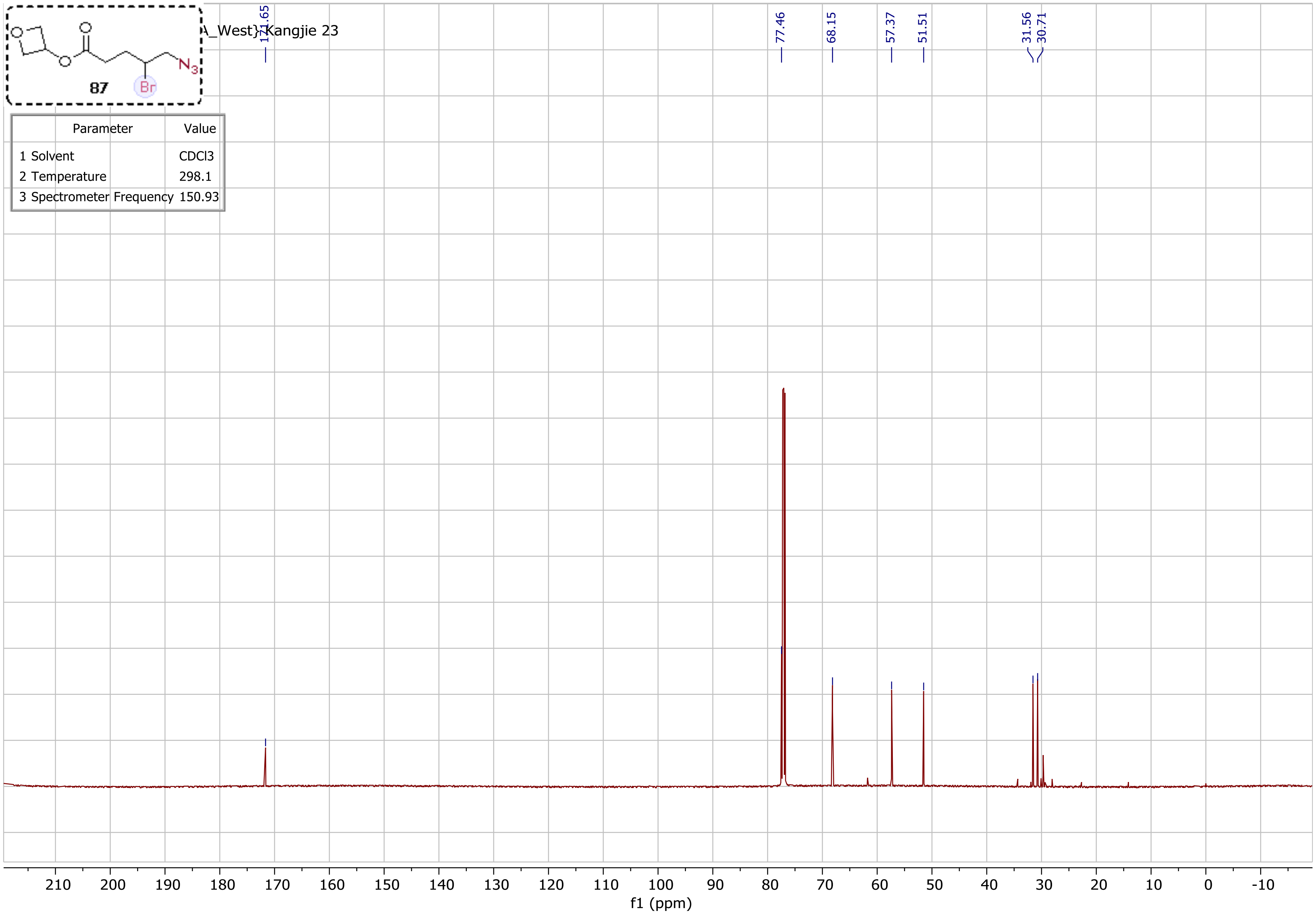

Parameter	Value
1 Solvent	CDCl3
2 Temperature	298.1
3 Spectrometer Frequency	150.93

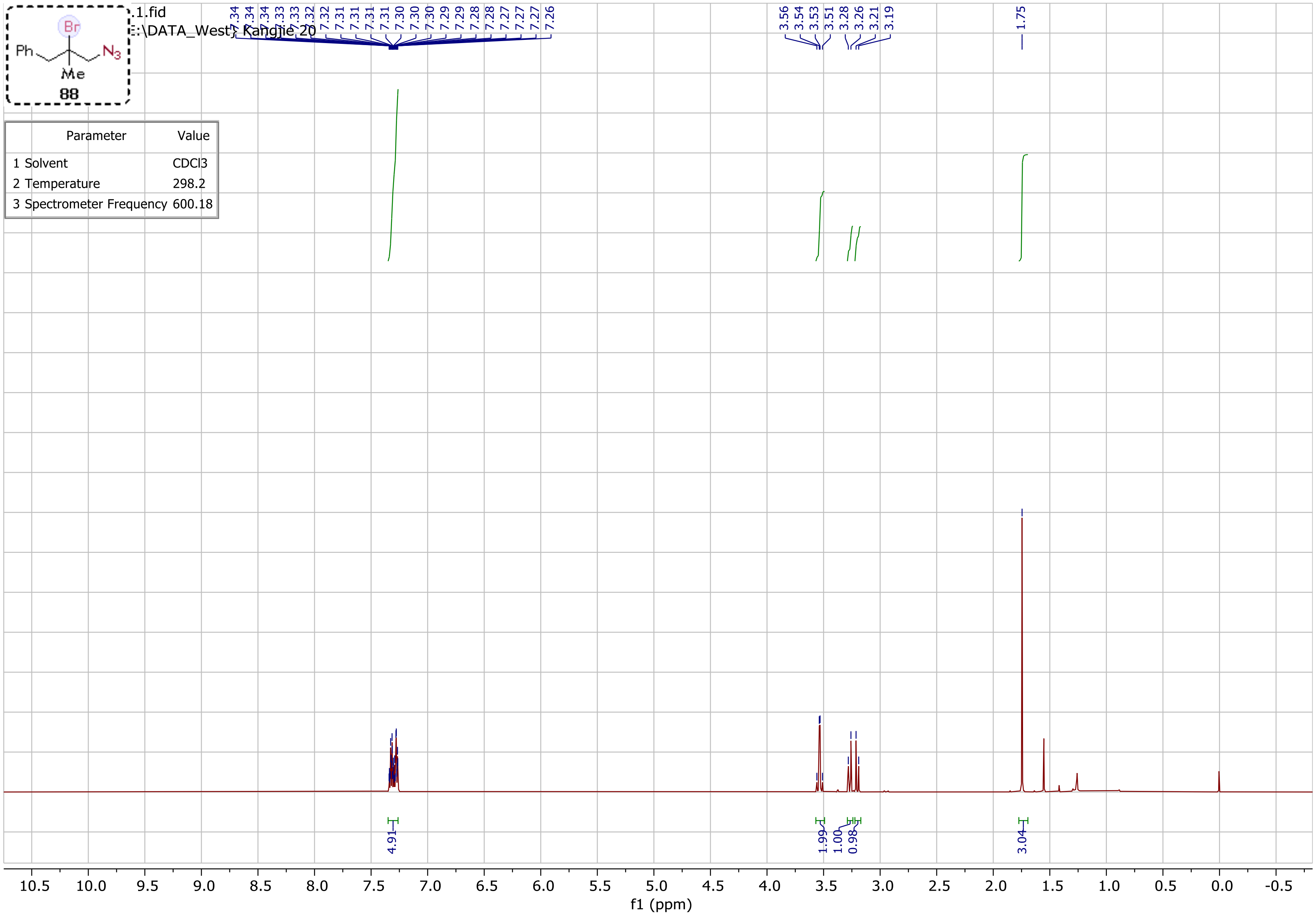

Br
Ph
N3
Me
88
.1.fid
E:\DATA_West} Kangjie 20
Parameter Value
1 Solvent CDCl3
2 Temperature 298.2
3 Spectrometer Frequency 600.18
7.34 7.34 7.34 7.33 7.33 7.32 7.32 7.31 7.31 7.31 7.30 7.30 7.29 7.29 7.28 7.28 7.27 7.27 7.27 7.26
3.56 3.54 3.53 3.51 3.28 3.26 3.21 3.19
1.75
4.91
1.99
1.00
0.98
3.04
10.5 10.0 9.5 9.0 8.5 8.0 7.5 7.0 6.5 6.0 5.5 5.0 4.5 4.0 3.5 3.0 2.5 2.0 1.5 1.0 0.5 0.0 -0.5
f1 (ppm)

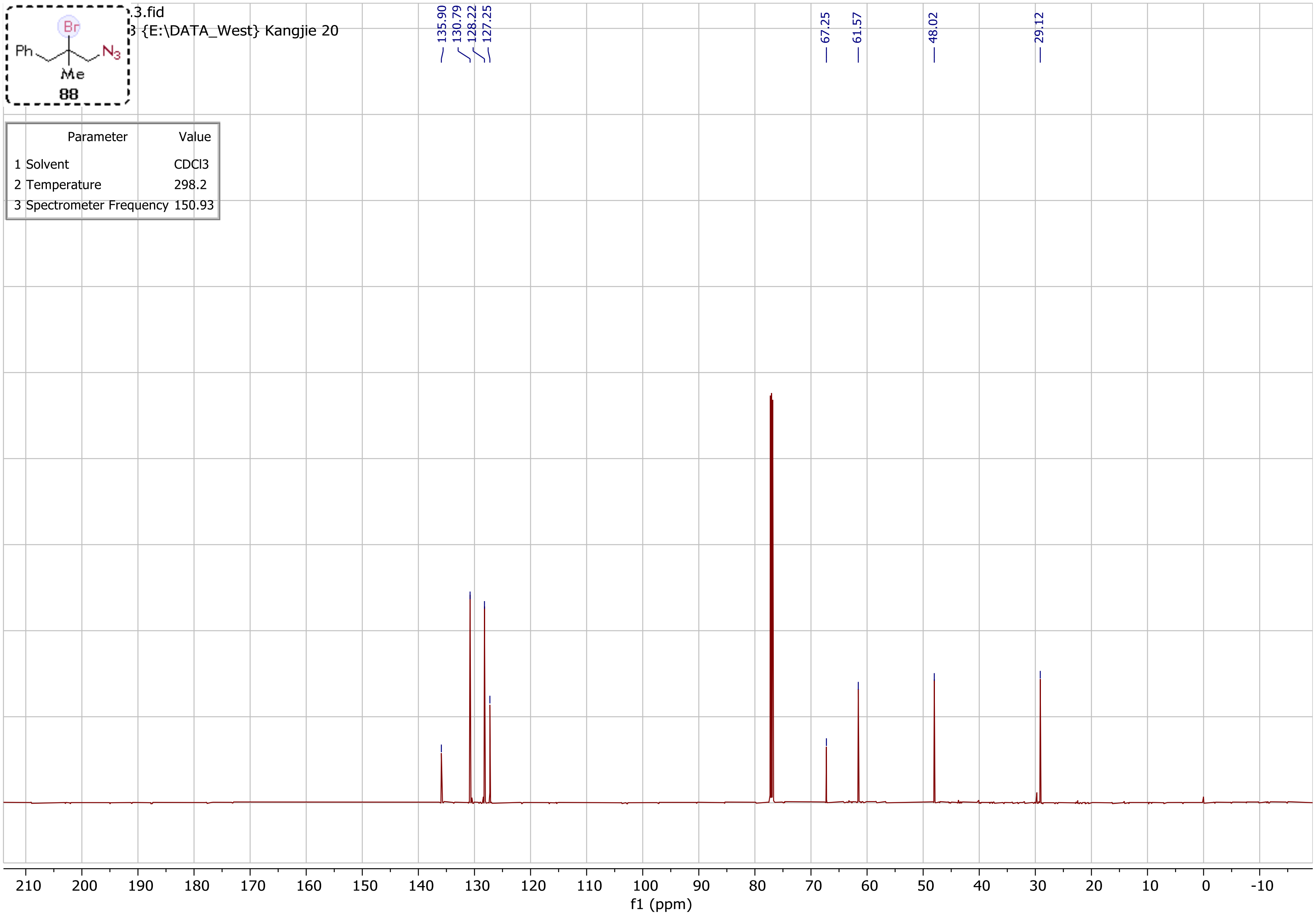

	Parameter	Value
1	Solvent	CDCl3
2	Temperature	298.2
3	Spectrometer Frequency	150.93

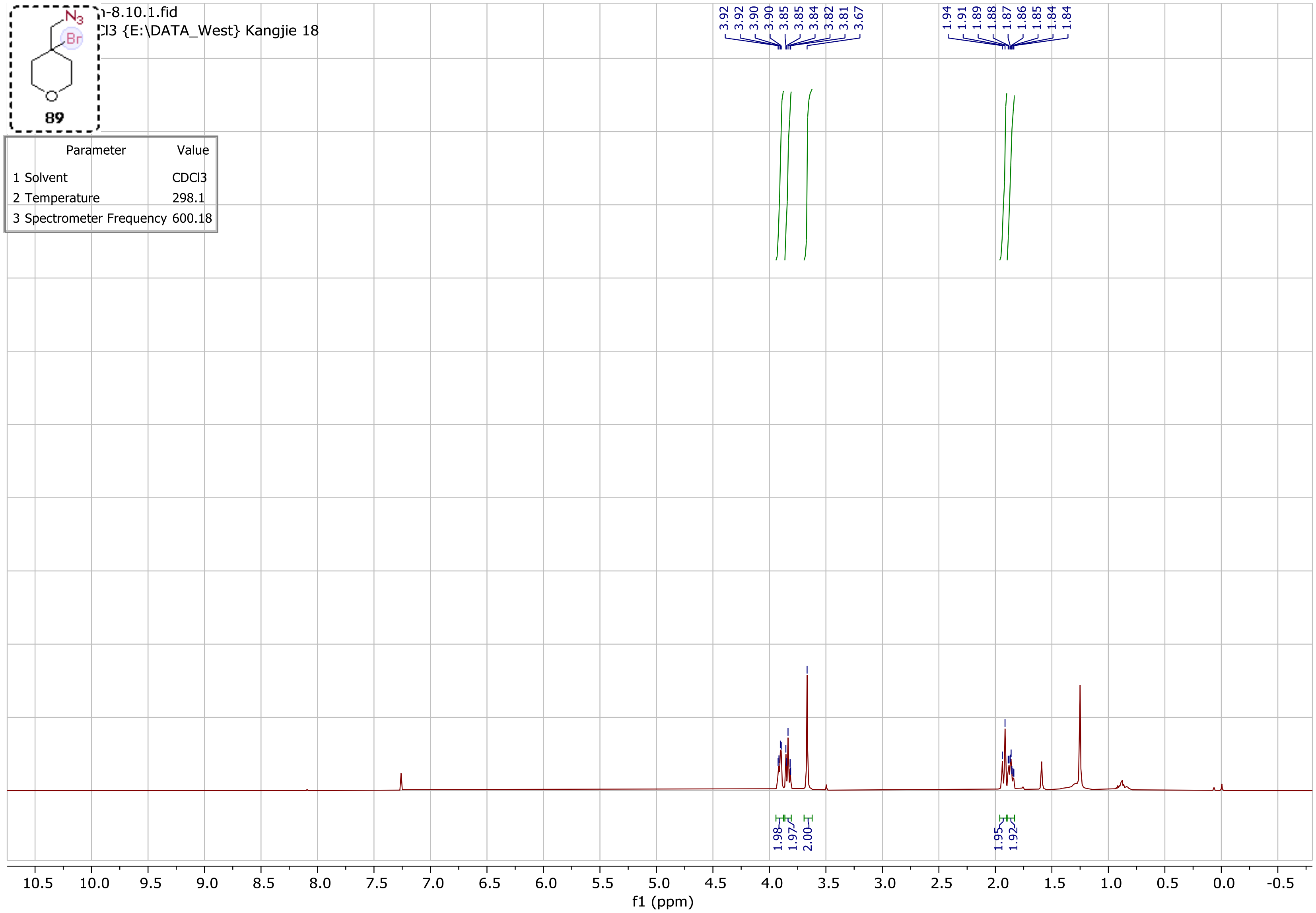

Parameter	Value
1 Solvent	CDCl3
2 Temperature	298.1
3 Spectrometer Frequency	600.18

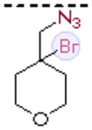

89

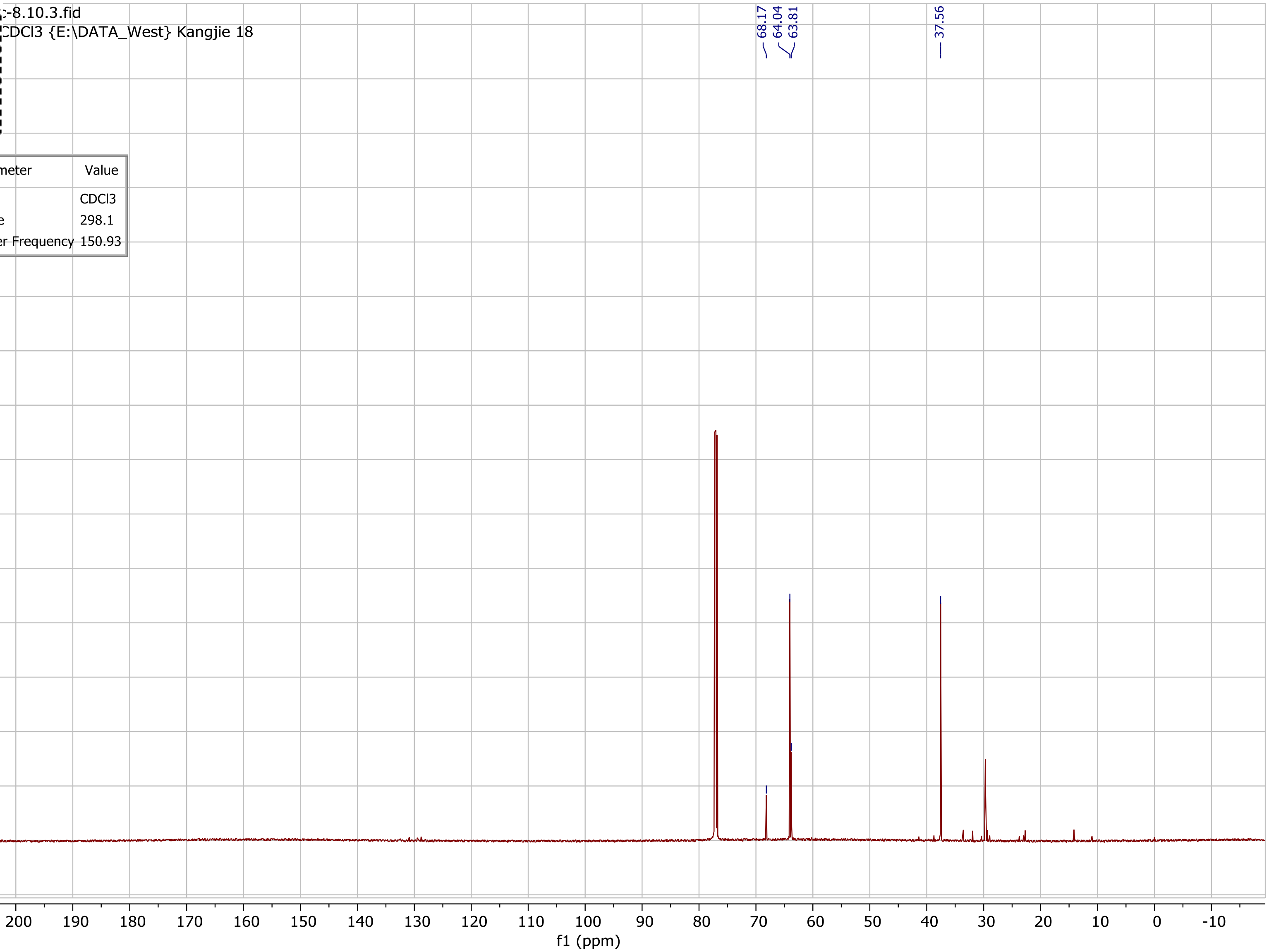

	Parameter	Value
1	Solvent	CDCl3
2	Temperature	298.1
3	Spectrometer Frequency	150.93

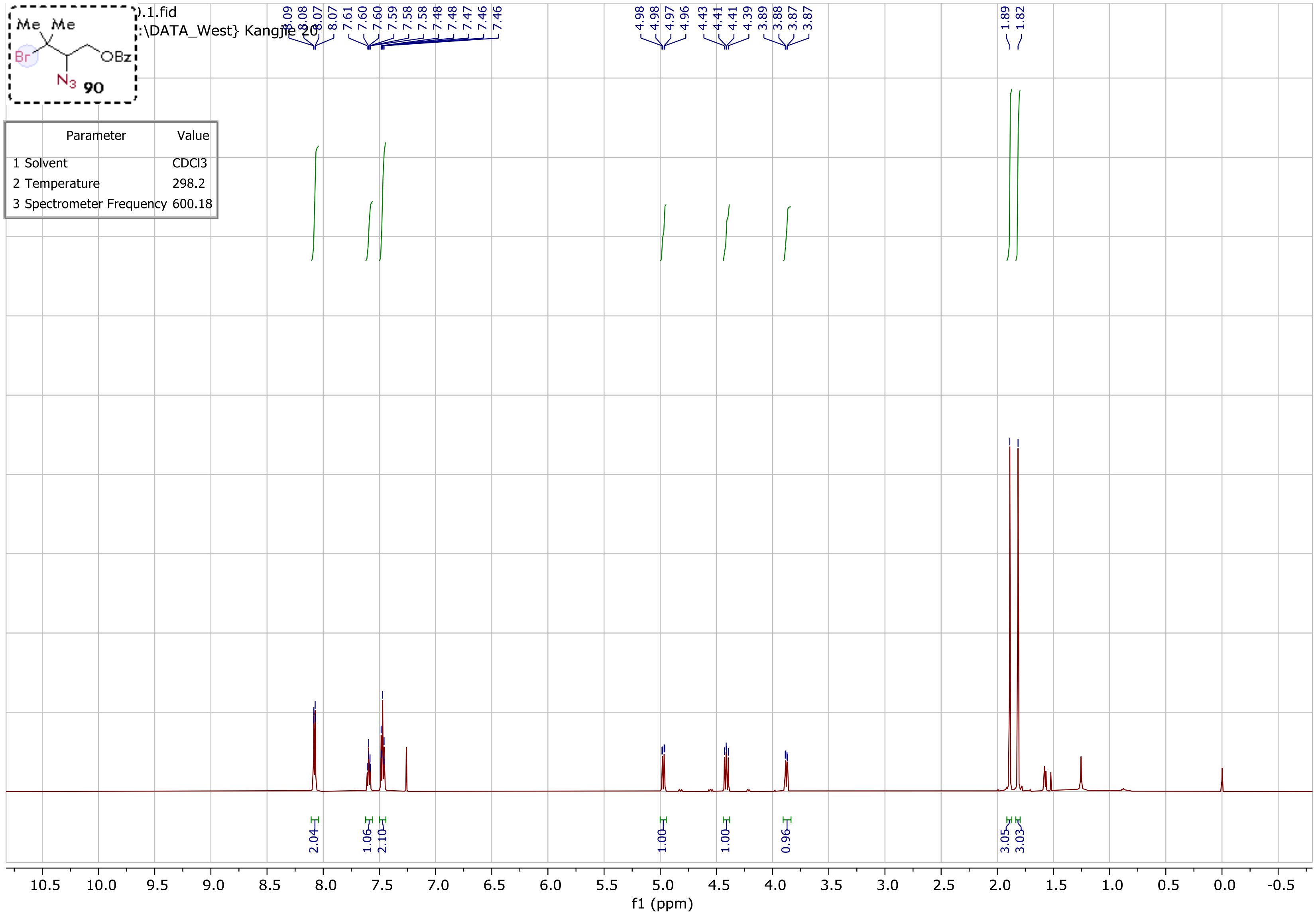

).1.fid
:\DATA_West} Kangjie 20
Me Me
Br
OBz
N3
90
Parameter Value
1 Solvent CDCl3
2 Temperature 298.2
3 Spectrometer Frequency 600.18
8.09 8.08 8.07 8.07 7.61 7.60 7.60 7.59 7.58 7.58 7.48 7.48 7.47 7.46 7.46
4.98 4.98 4.97 4.96 4.43 4.41 4.41 4.39 3.89 3.88 3.87 3.87
1.89 1.82
2.04 1.06 2.10 1.00 1.00 0.96 3.05 3.03
10.5 10.0 9.5 9.0 8.5 8.0 7.5 7.0 6.5 6.0 5.5 5.0 4.5 4.0 3.5 3.0 2.5 2.0 1.5 1.0 0.5 0.0 -0.5
f1 (ppm)

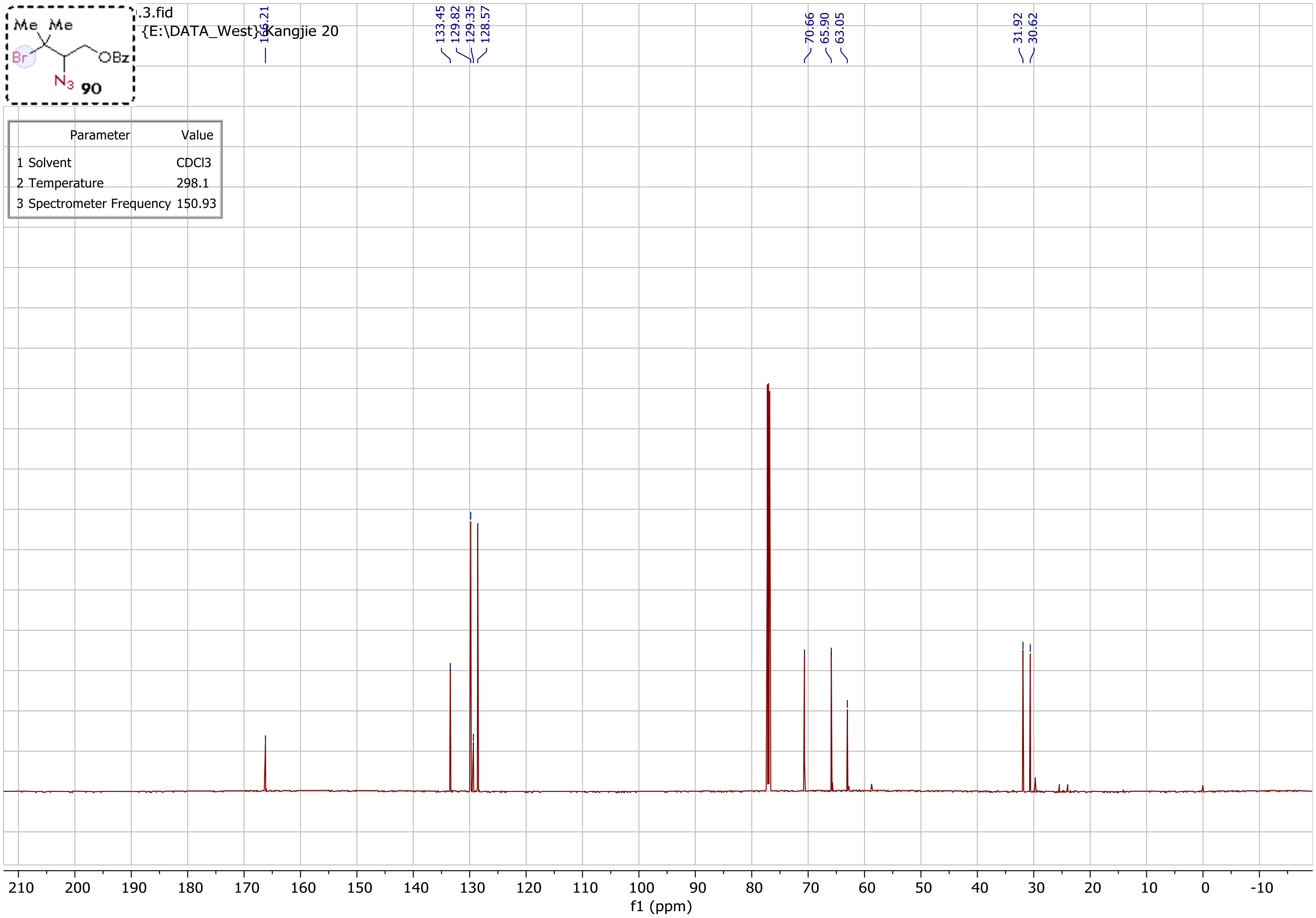

Me Me
Br
N3
OBz
90
.3.fid
{E:\DATA_West}
Parameter Value
1 Solvent CDCl3
2 Temperature 298.1
3 Spectrometer Frequency 150.93
133.45
129.82
129.35
128.57
70.66
65.90
63.05
31.92
30.62
210 200 190 180 170 160 150 140 130 120 110 100 90 80 70 60 50 40 30 20 10 0 -10
f1 (ppm)

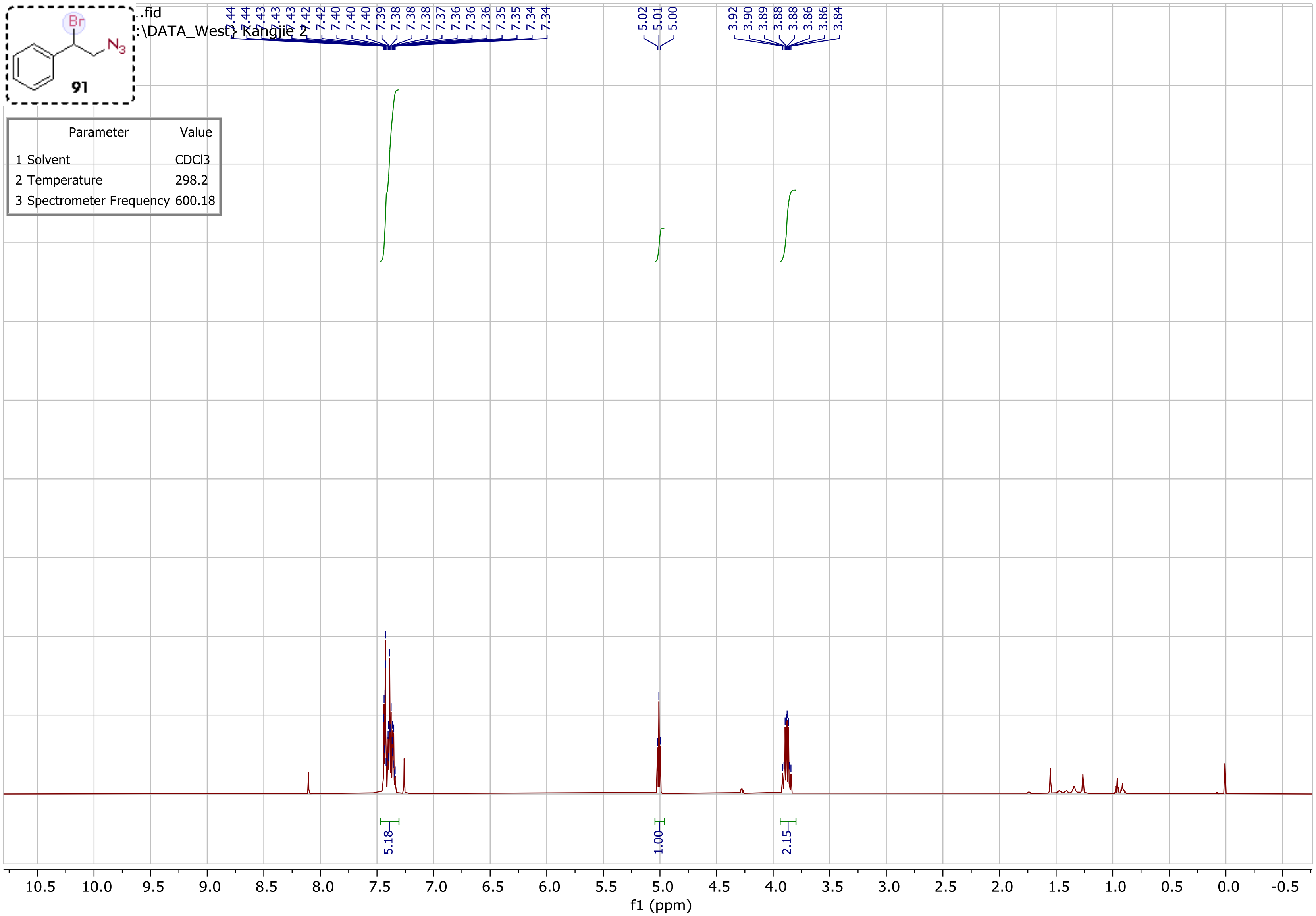
..fid
:\DATA_West} Kangjie 2
7.44 7.44 7.43 7.43 7.43 7.42 7.42 7.40 7.40 7.40 7.39 7.38 7.38 7.38 7.37 7.36 7.36 7.36 7.35 7.35 7.34 7.34
5.02 5.01 5.00
3.92 3.90 3.89 3.88 3.88 3.86 3.86 3.84
Br
N3
91
Parameter Value
1 Solvent CDCl3
2 Temperature 298.2
3 Spectrometer Frequency 600.18
5.18
1.00
2.15
10.5 10.0 9.5 9.0 8.5 8.0 7.5 7.0 6.5 6.0 5.5 5.0 4.5 4.0 3.5 3.0 2.5 2.0 1.5 1.0 0.5 0.0 -0.5
f1 (ppm)

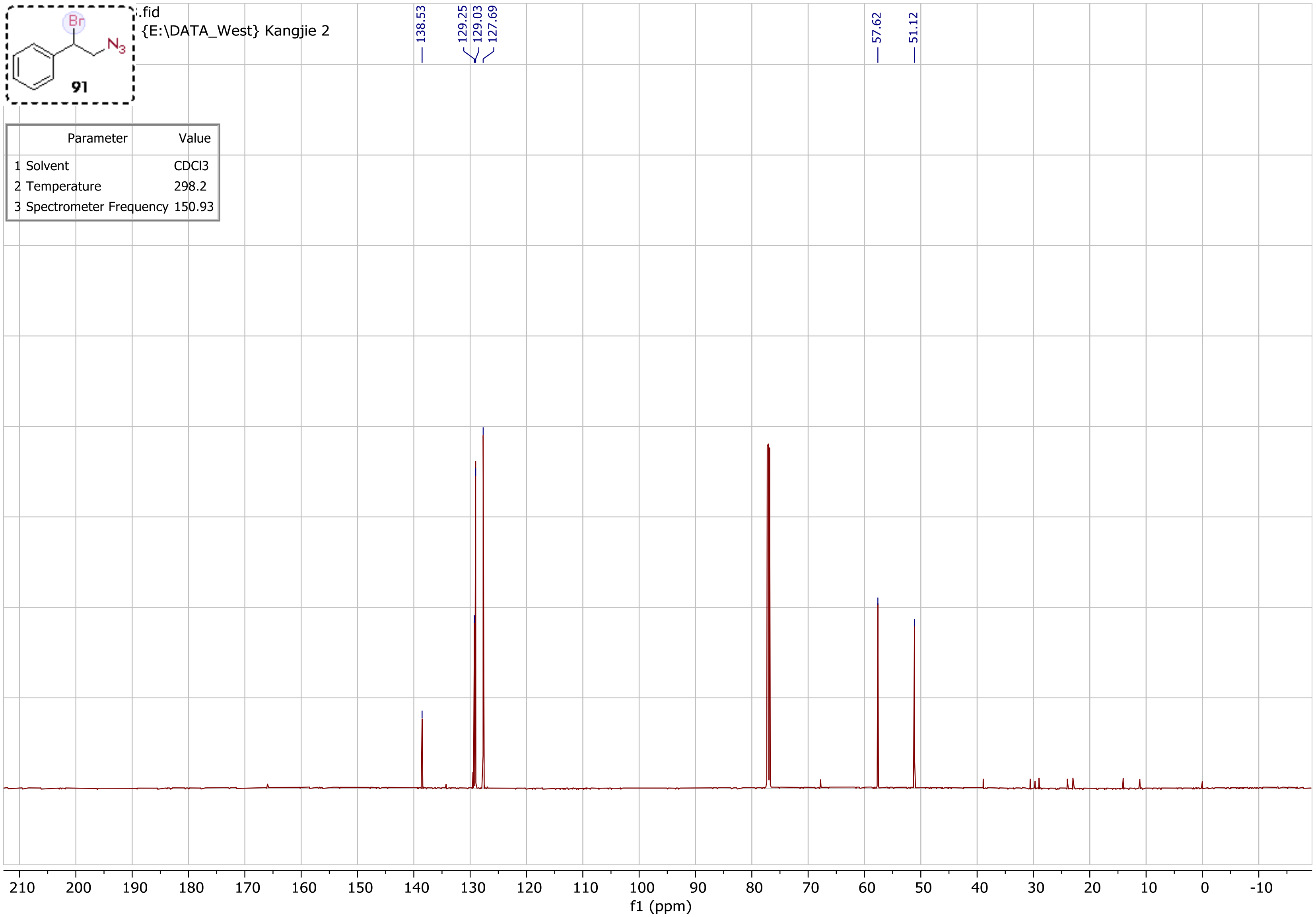
Br
N3
91
.fid
{E:\DATA_West} Kangjie 2
Parameter Value
1 Solvent CDCl3
2 Temperature 298.2
3 Spectrometer Frequency 150.93
138.53
129.25
129.03
127.69
57.62
51.12
210 200 190 180 170 160 150 140 130 120 110 100 90 80 70 60 50 40 30 20 10 0 -10
f1 (ppm)

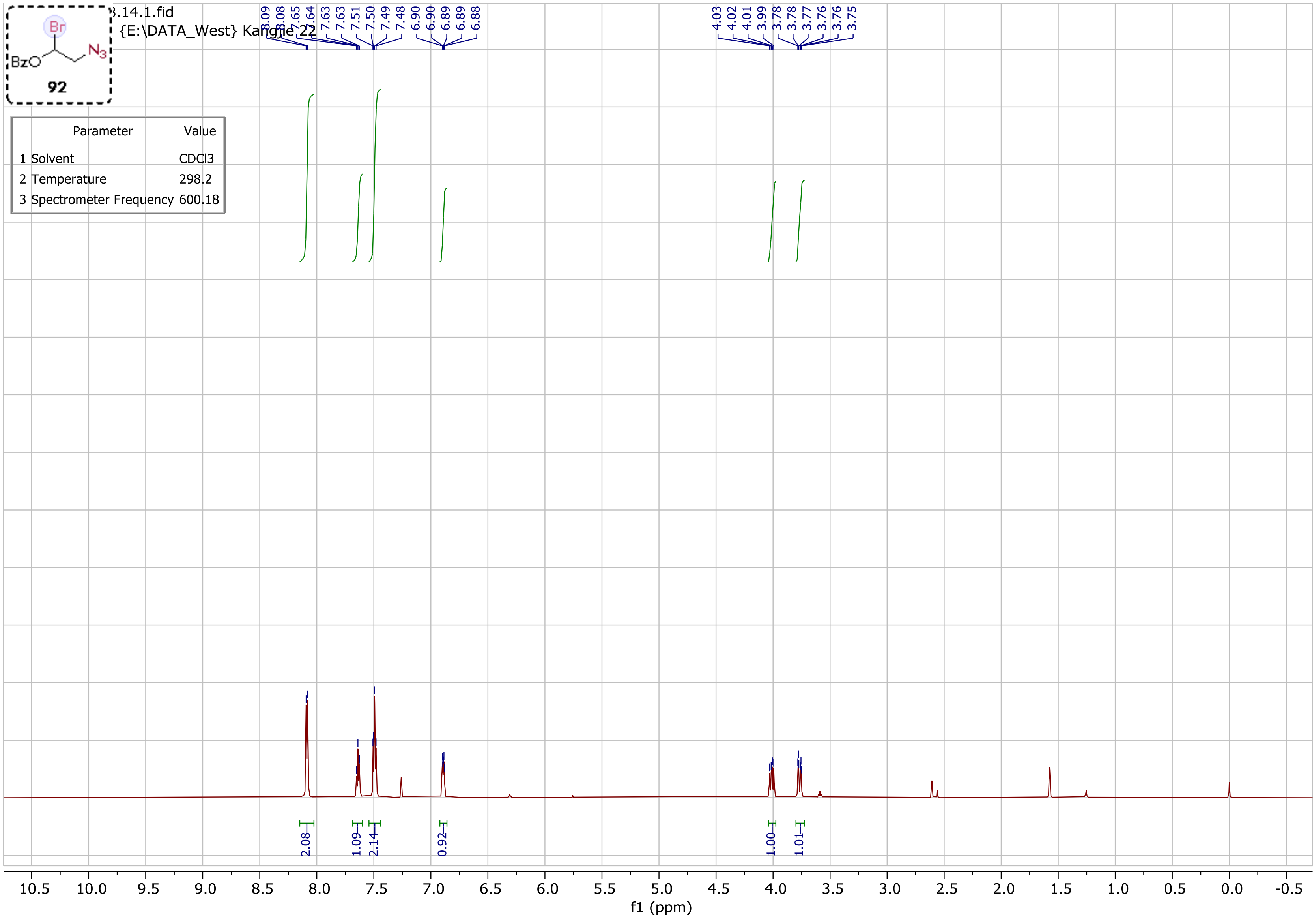

.14.1.fid
{E:\DATA_West} Kangjie 22
Br
BzO
N3
92
Parameter | Value
1 Solvent | CDCl3
2 Temperature | 298.2
3 Spectrometer Frequency | 600.18
8.09 8.08 7.65 7.64 7.63 7.63 7.51 7.50 7.49 7.48 6.90 6.90 6.89 6.89 6.88
4.03 4.02 4.01 3.99 3.78 3.78 3.77 3.76 3.76 3.75
2.08 1.09 2.14 0.92 1.00 1.01
10.5 10.0 9.5 9.0 8.5 8.0 7.5 7.0 6.5 6.0 5.5 5.0 4.5 4.0 3.5 3.0 2.5 2.0 1.5 1.0 0.5 0.0 -0.5
f1 (ppm)

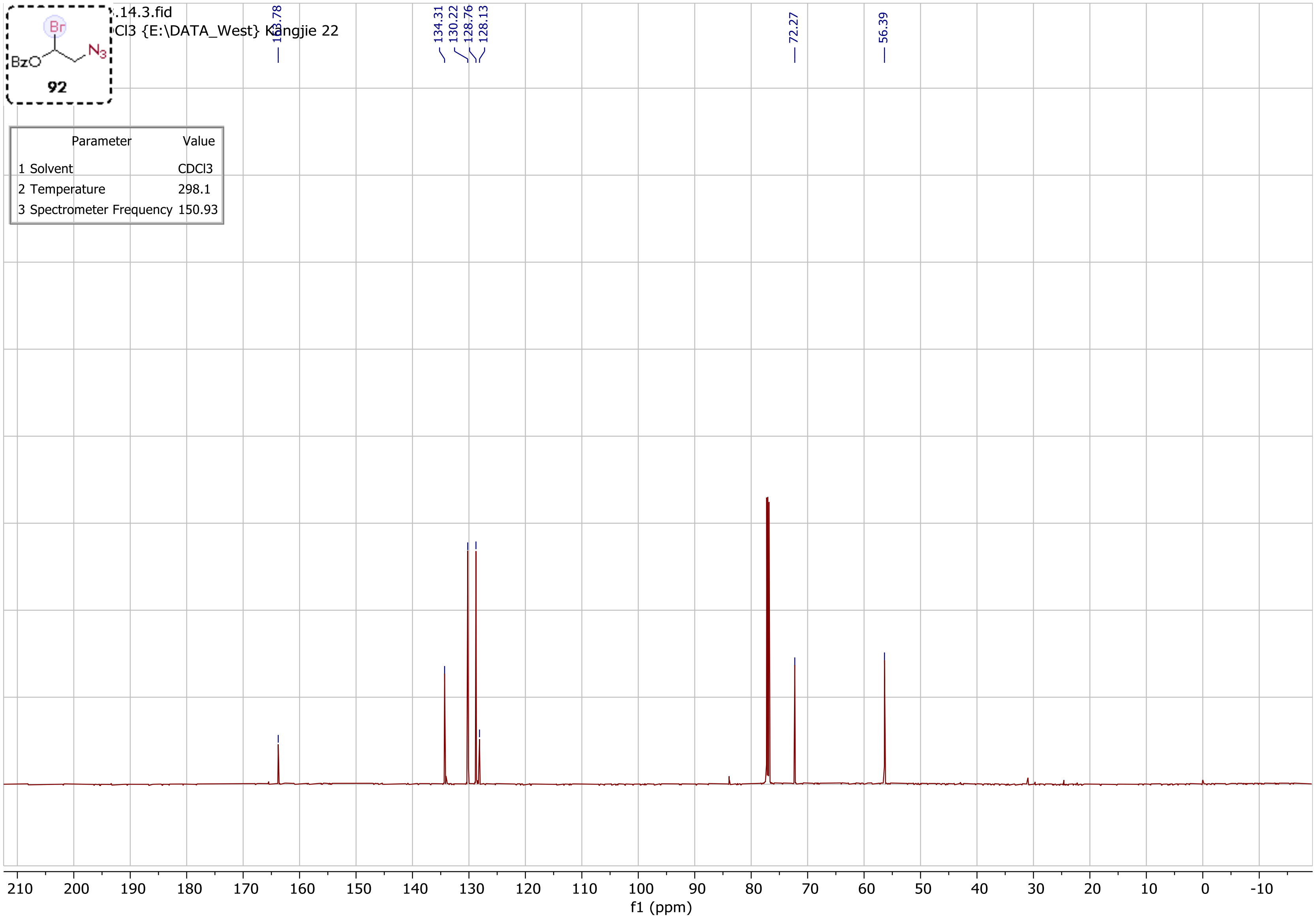

BzO
Br
N3
92
.14.3.fid
Cl3 {E:\DATA_West} Kangjie 22
Parameter Value
1 Solvent CDCl3
2 Temperature 298.1
3 Spectrometer Frequency 150.93
134.31
130.22
128.76
128.13
72.27
56.39
210 200 190 180 170 160 150 140 130 120 110 100 90 80 70 60 50 40 30 20 10 0 -10
f1 (ppm)

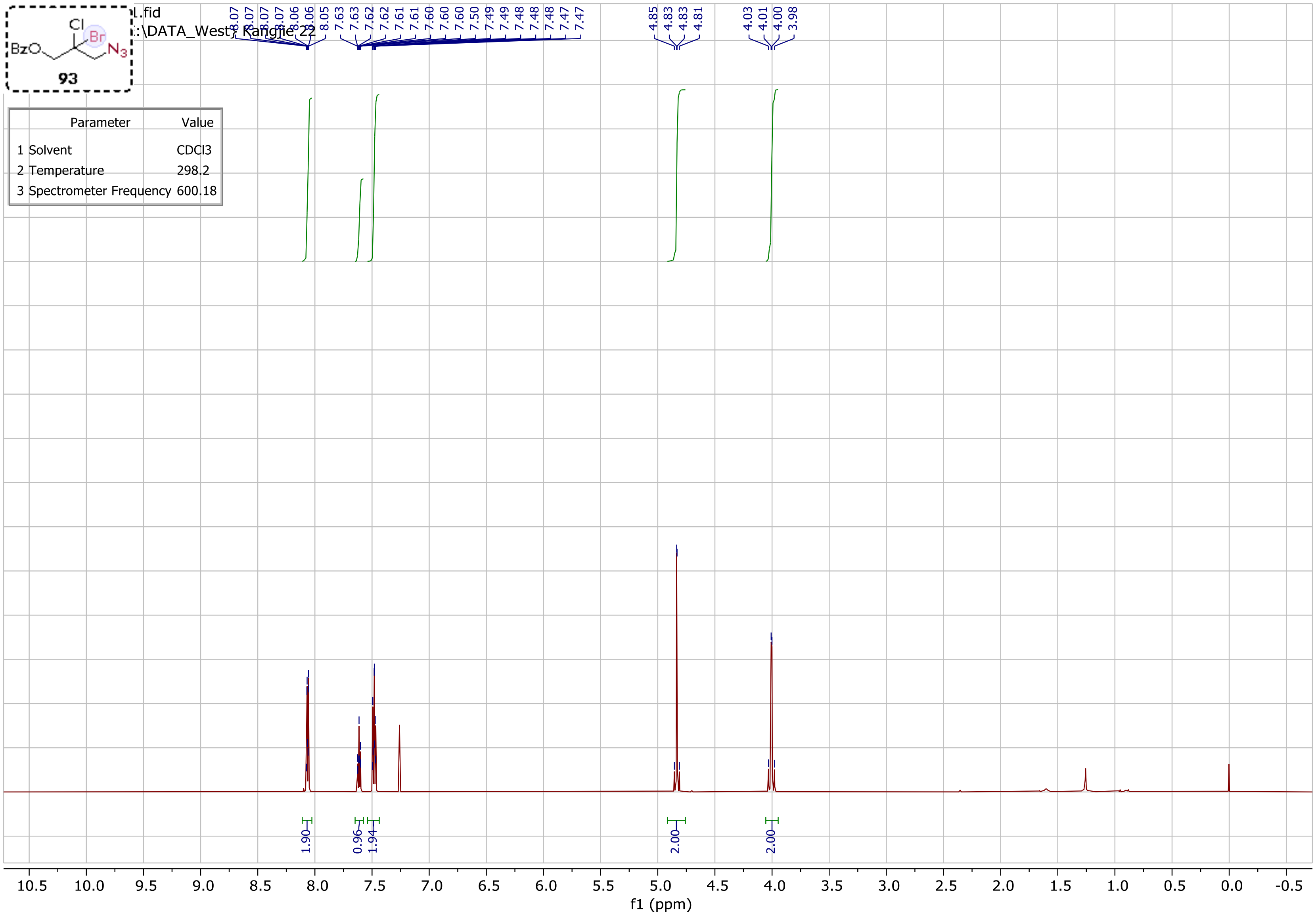
Cl
Br
BzO
N3
93
Parameter Value
1 Solvent CDCl3
2 Temperature 298.2
3 Spectrometer Frequency 600.18
8.07 8.07 8.07 8.07 8.06 8.06 8.05 7.63 7.63 7.62 7.62 7.61 7.61 7.60 7.60 7.60 7.50 7.49 7.49 7.48 7.48 7.48 7.47 7.47
4.85 4.83 4.83 4.81
4.03 4.01 4.00 3.98
1.90
0.96
1.94
2.00
2.00
10.5 10.0 9.5 9.0 8.5 8.0 7.5 7.0 6.5 6.0 5.5 5.0 4.5 4.0 3.5 3.0 2.5 2.0 1.5 1.0 0.5 0.0 -0.5
f1 (ppm)

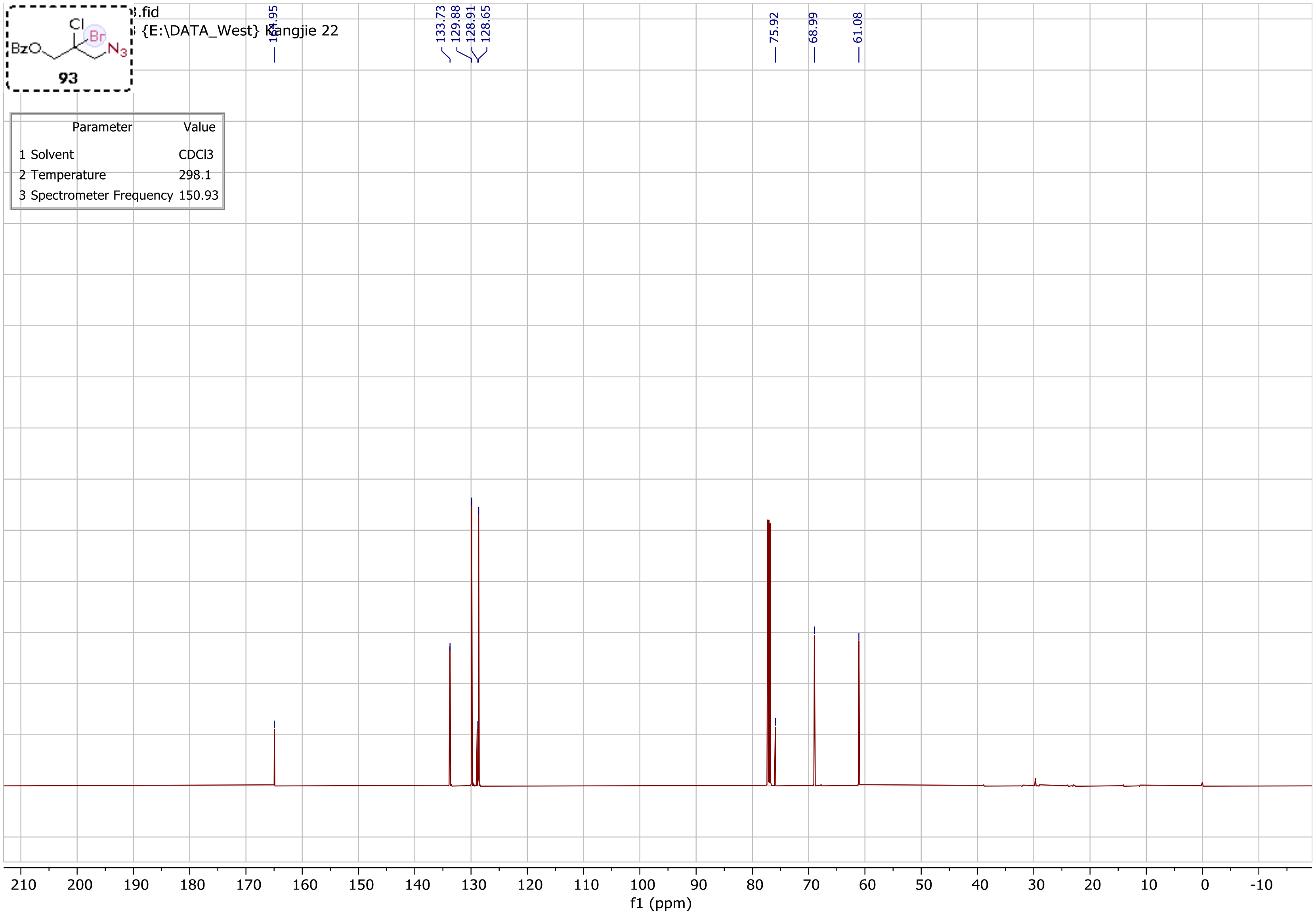

93
.fid
{E:\DATA_West} Kangjie 22
133.73
129.88
128.91
128.65
75.92
68.99
61.08
Parameter Value
1 Solvent CDCl3
2 Temperature 298.1
3 Spectrometer Frequency 150.93
210 200 190 180 170 160 150 140 130 120 110 100 90 80 70 60 50 40 30 20 10 0 -10
f1 (ppm)

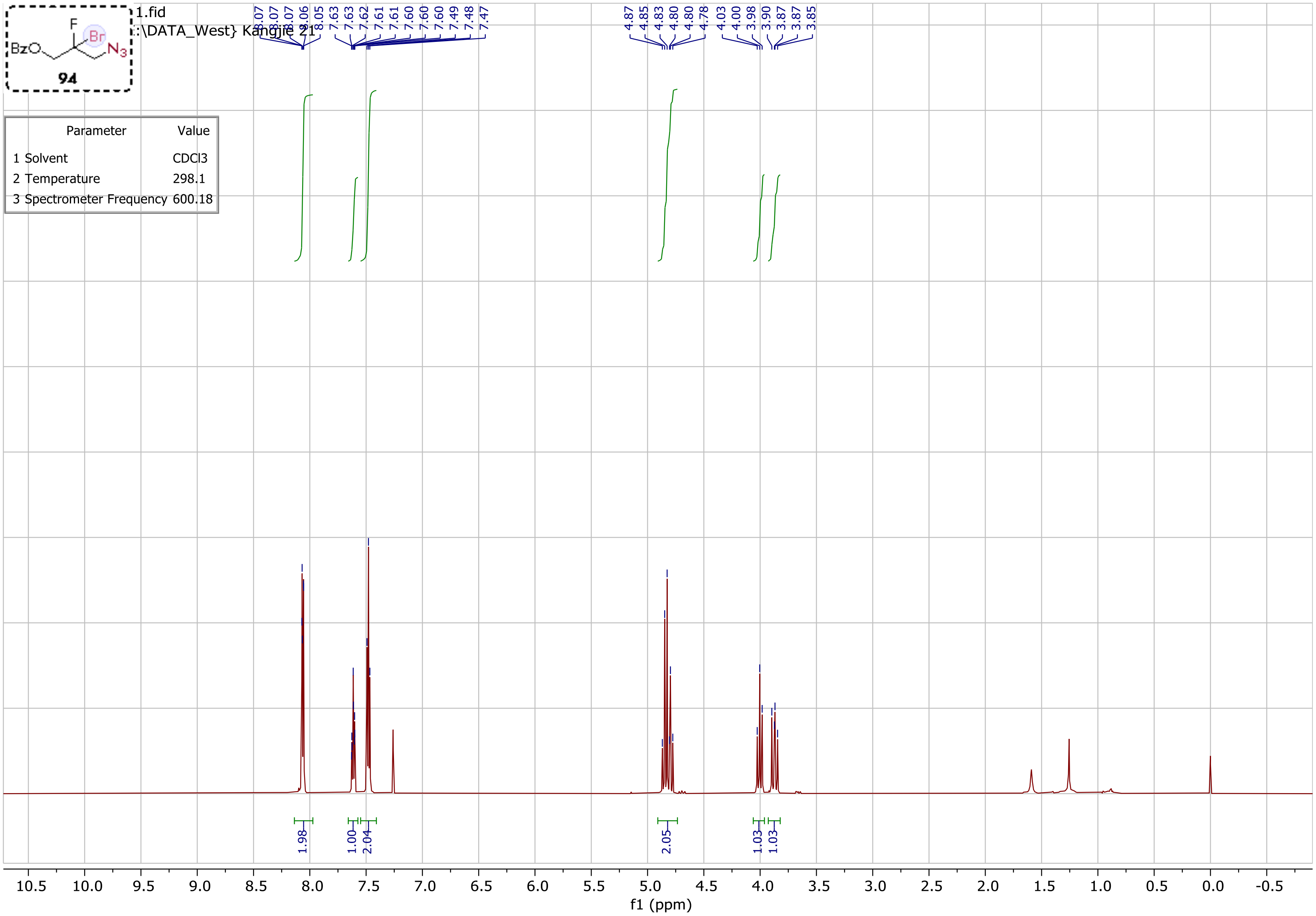

94
1.fid
:\DATA_West} Kangjie 21
Parameter Value
1 Solvent CDCl3
2 Temperature 298.1
3 Spectrometer Frequency 600.18
8.07 8.07 8.07 8.06 8.05 7.63 7.63 7.62 7.61 7.61 7.60 7.60 7.60 7.49 7.48 7.47
4.87 4.85 4.83 4.80 4.80 4.78 4.03 4.00 3.98 3.90 3.87 3.87 3.85
1.98 1.00 2.04 2.05 1.03 1.03
f1 (ppm)

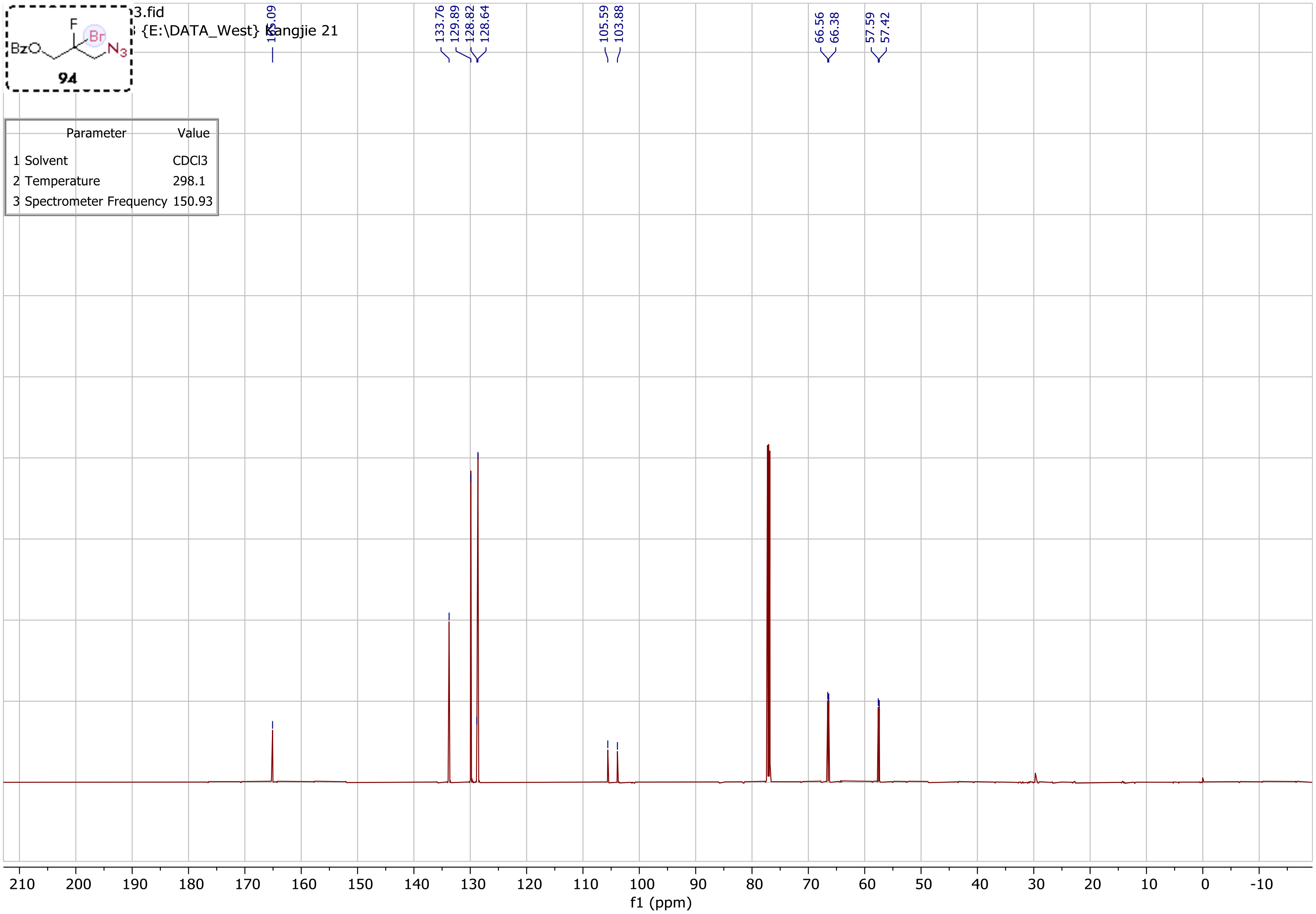

Parameter	Value
1 Solvent	CDCl3
2 Temperature	298.1
3 Spectrometer Frequency	150.93

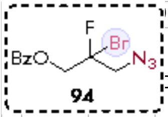

.10.fid

D:\DATA\West} Kangjie 23

	Parameter	Value
1	Solvent	CDCl3
2	Temperature	298.0
3	Spectrometer Frequency	564.46

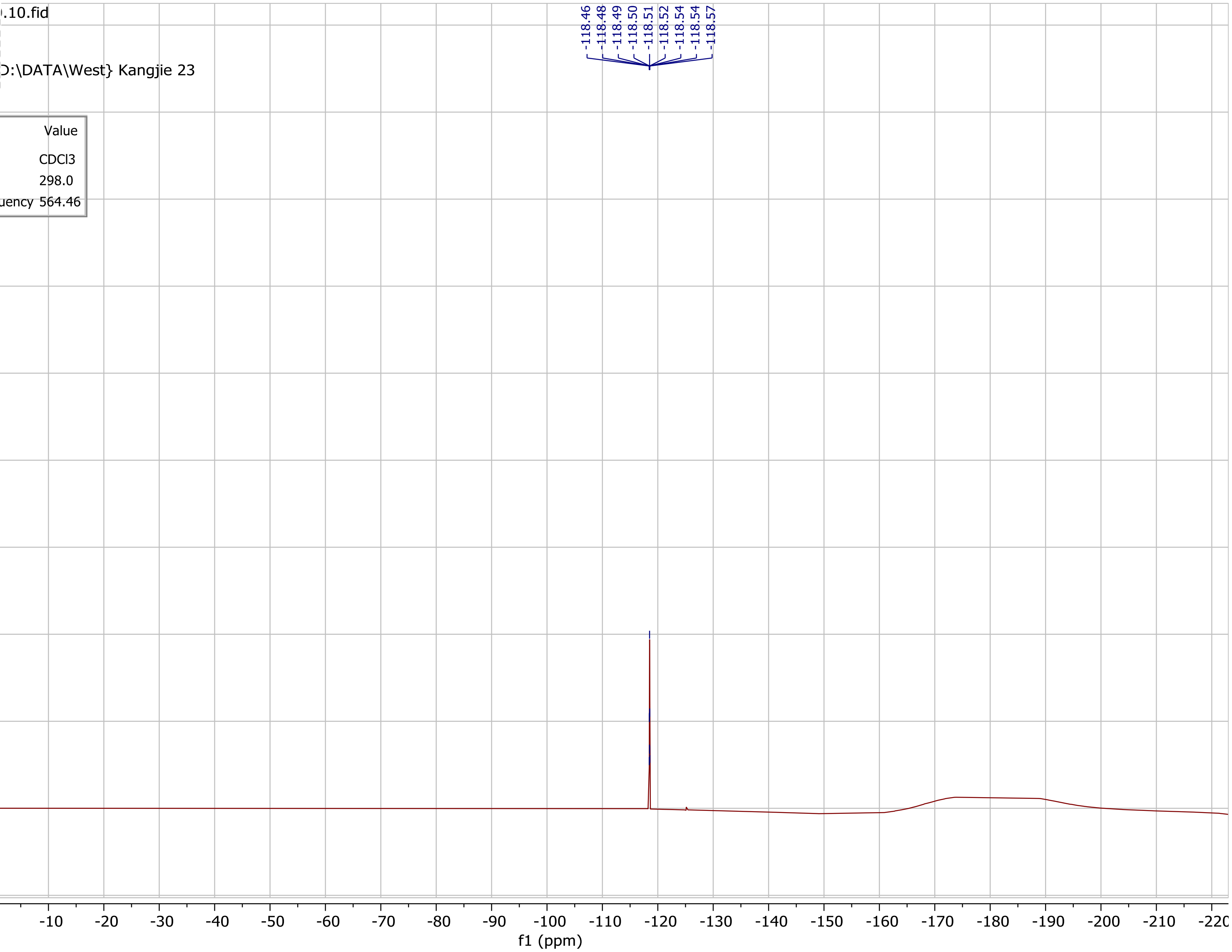

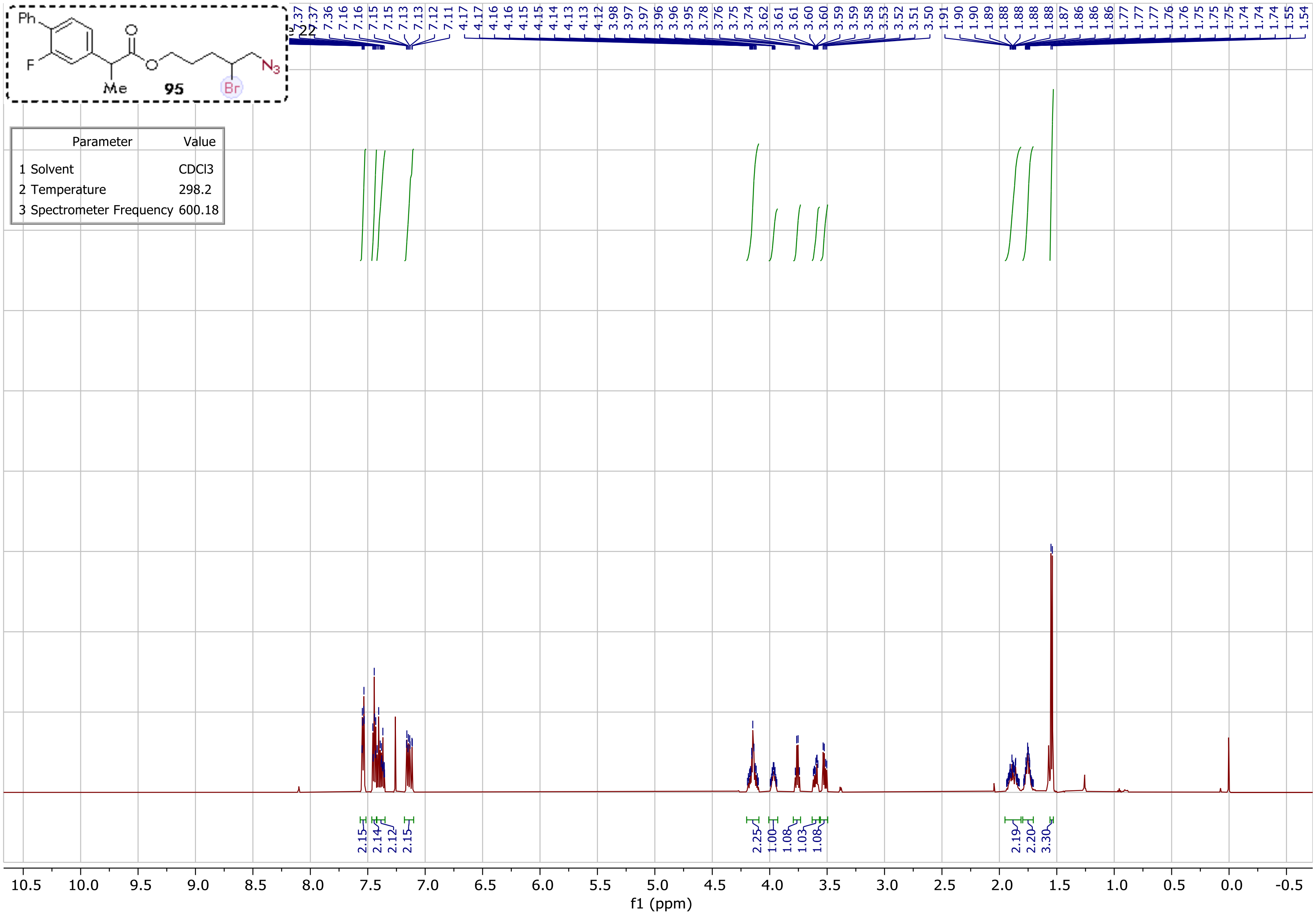
Ph
F
Me
95
Br
N3
Parameter Value
1 Solvent CDCl3
2 Temperature 298.2
3 Spectrometer Frequency 600.18
2.15
2.14
2.12
2.15
2.25
1.00
1.08
1.03
1.08
2.19
2.20
3.30
f1 (ppm)

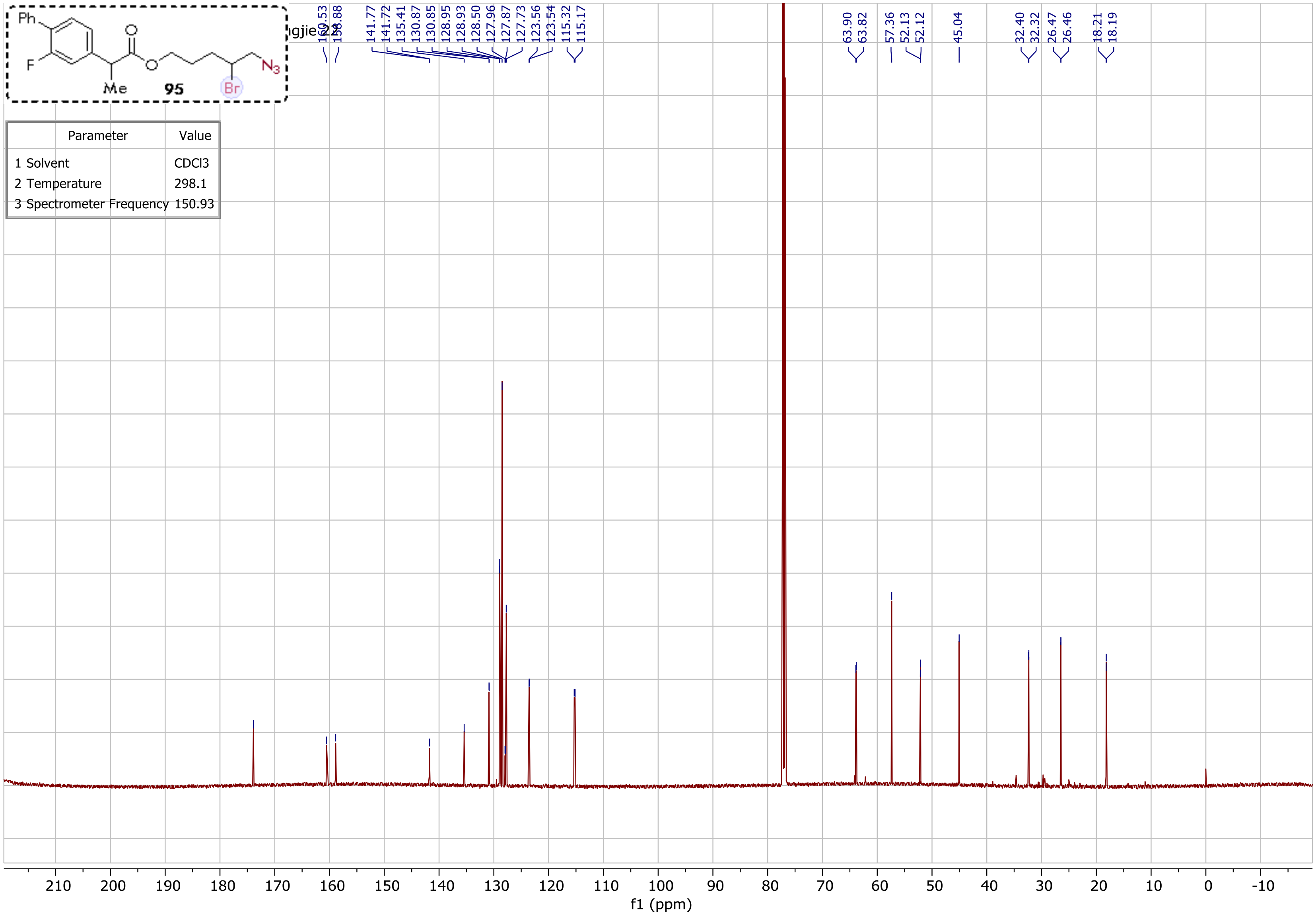

Parameter	Value
1 Solvent	CDCl3
2 Temperature	298.1
3 Spectrometer Frequency	150.93

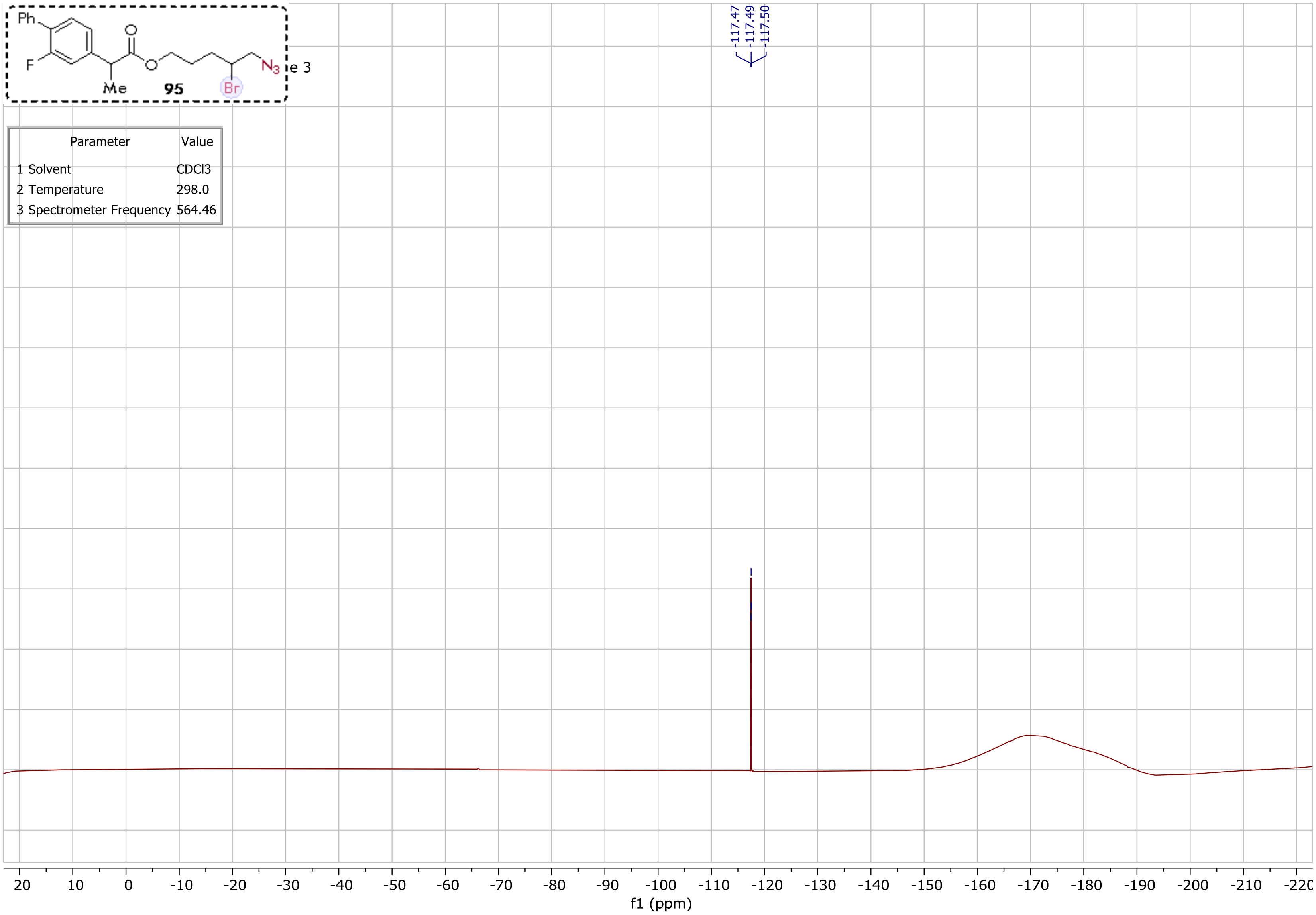

Parameter	Value
1 Solvent	CDCl3
2 Temperature	298.0
3 Spectrometer Frequency	564.46

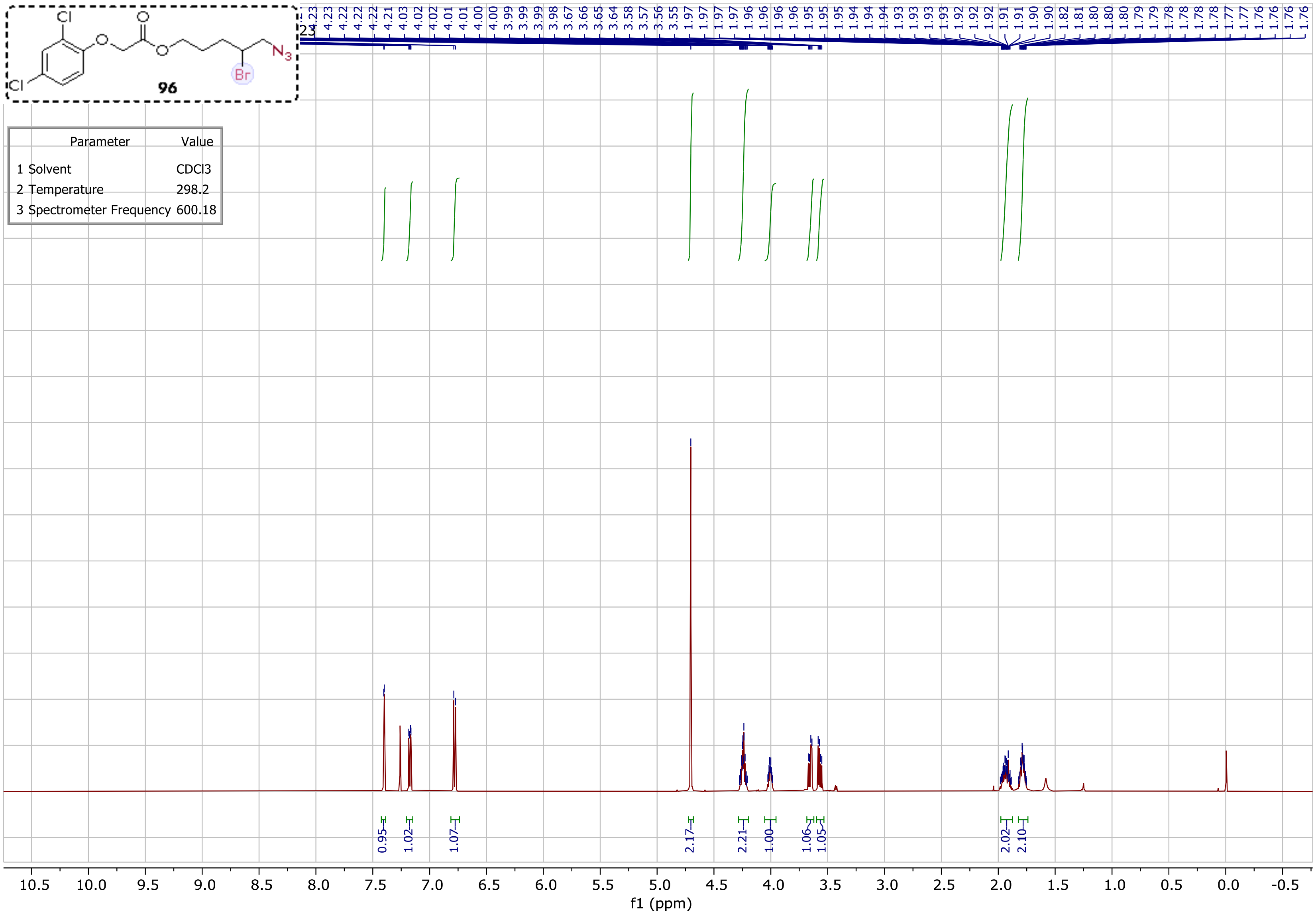

Cl
Cl
O
O
O
N3
Br
96
Parameter Value
1 Solvent CDCl3
2 Temperature 298.2
3 Spectrometer Frequency 600.18
0.95
1.02
1.07
2.17
2.21
1.00
1.06
1.05
2.02
2.10
10.5 10.0 9.5 9.0 8.5 8.0 7.5 7.0 6.5 6.0 5.5 5.0 4.5 4.0 3.5 3.0 2.5 2.0 1.5 1.0 0.5 0.0 -0.5
f1 (ppm)

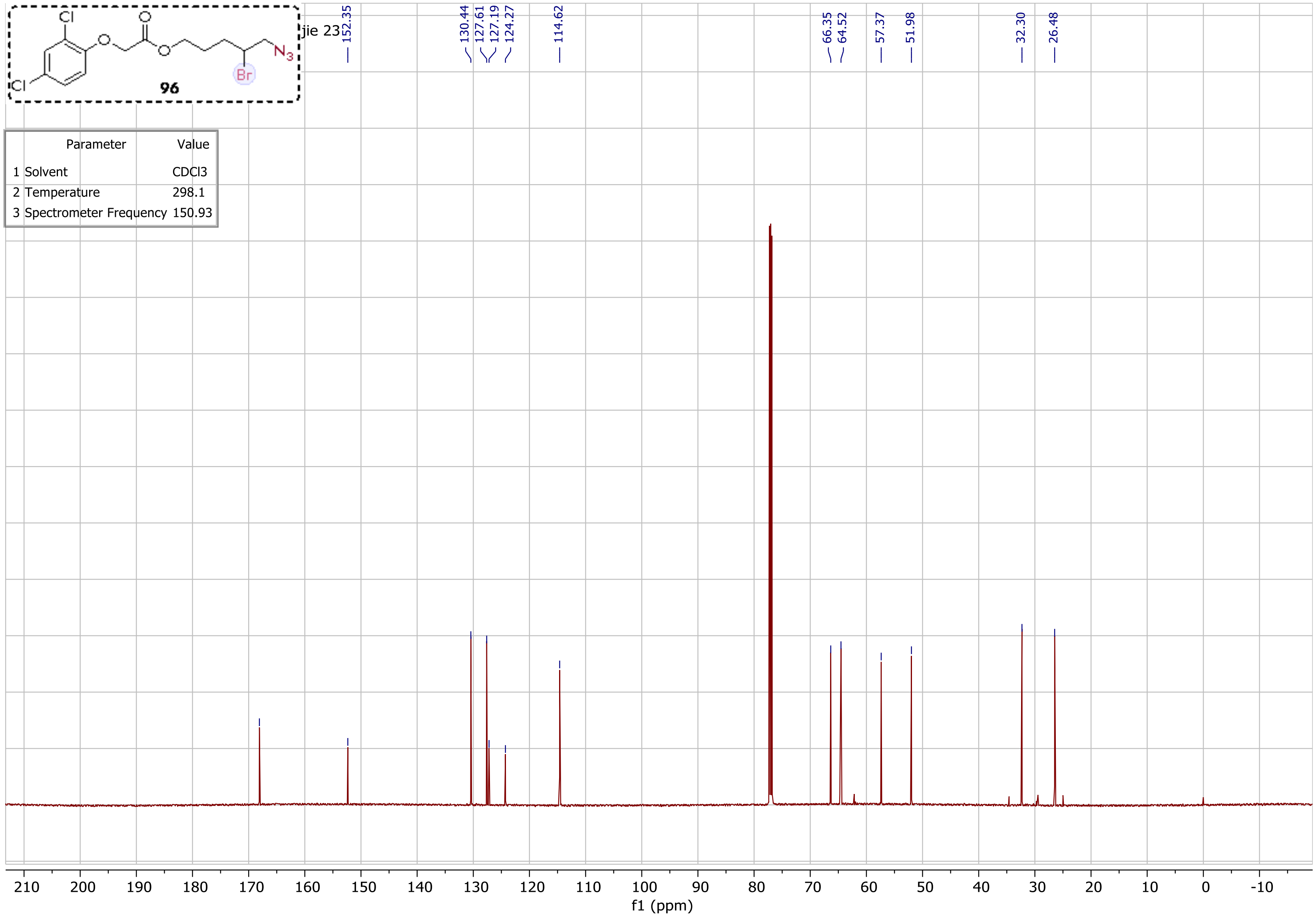

	Parameter	Value
1	Solvent	CDCl3
2	Temperature	298.1
3	Spectrometer Frequency	150.93

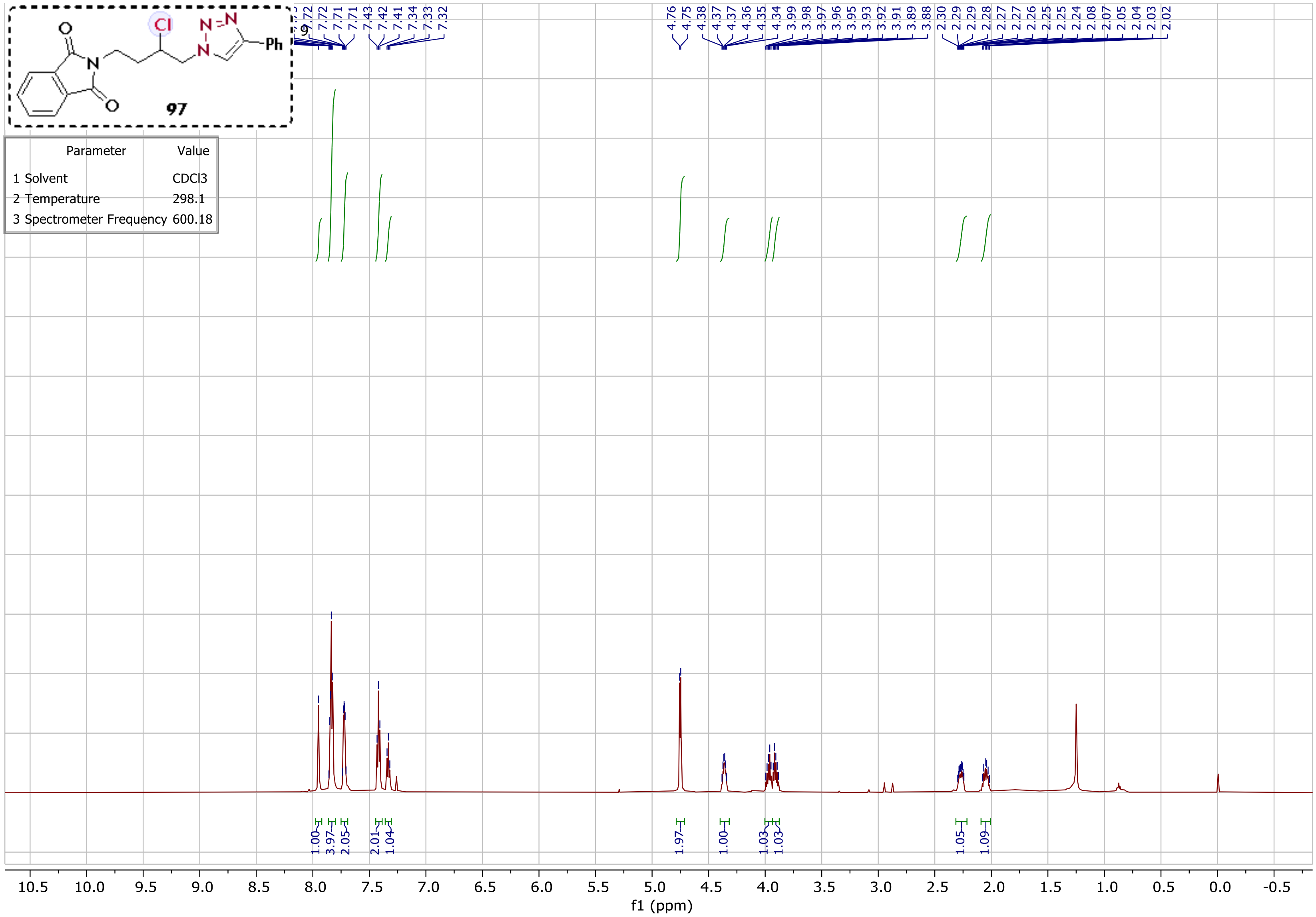
97
Cl
Ph
Parameter Value
1 Solvent CDCl3
2 Temperature 298.1
3 Spectrometer Frequency 600.18
7.72
7.72
7.71
7.71
7.43
7.42
7.41
7.34
7.33
7.32
4.76
4.75
4.38
4.37
4.37
4.36
4.35
4.34
3.99
3.98
3.97
3.96
3.95
3.93
3.92
3.91
3.89
3.88
2.30
2.29
2.29
2.28
2.27
2.27
2.26
2.25
2.25
2.24
2.08
2.07
2.05
2.04
2.03
2.02
1.00
3.97
2.05
2.01
1.04
1.97
1.00
1.03
1.03
1.05
1.09
10.5 10.0 9.5 9.0 8.5 8.0 7.5 7.0 6.5 6.0 5.5 5.0 4.5 4.0 3.5 3.0 2.5 2.0 1.5 1.0 0.5 0.0 -0.5
f1 (ppm)

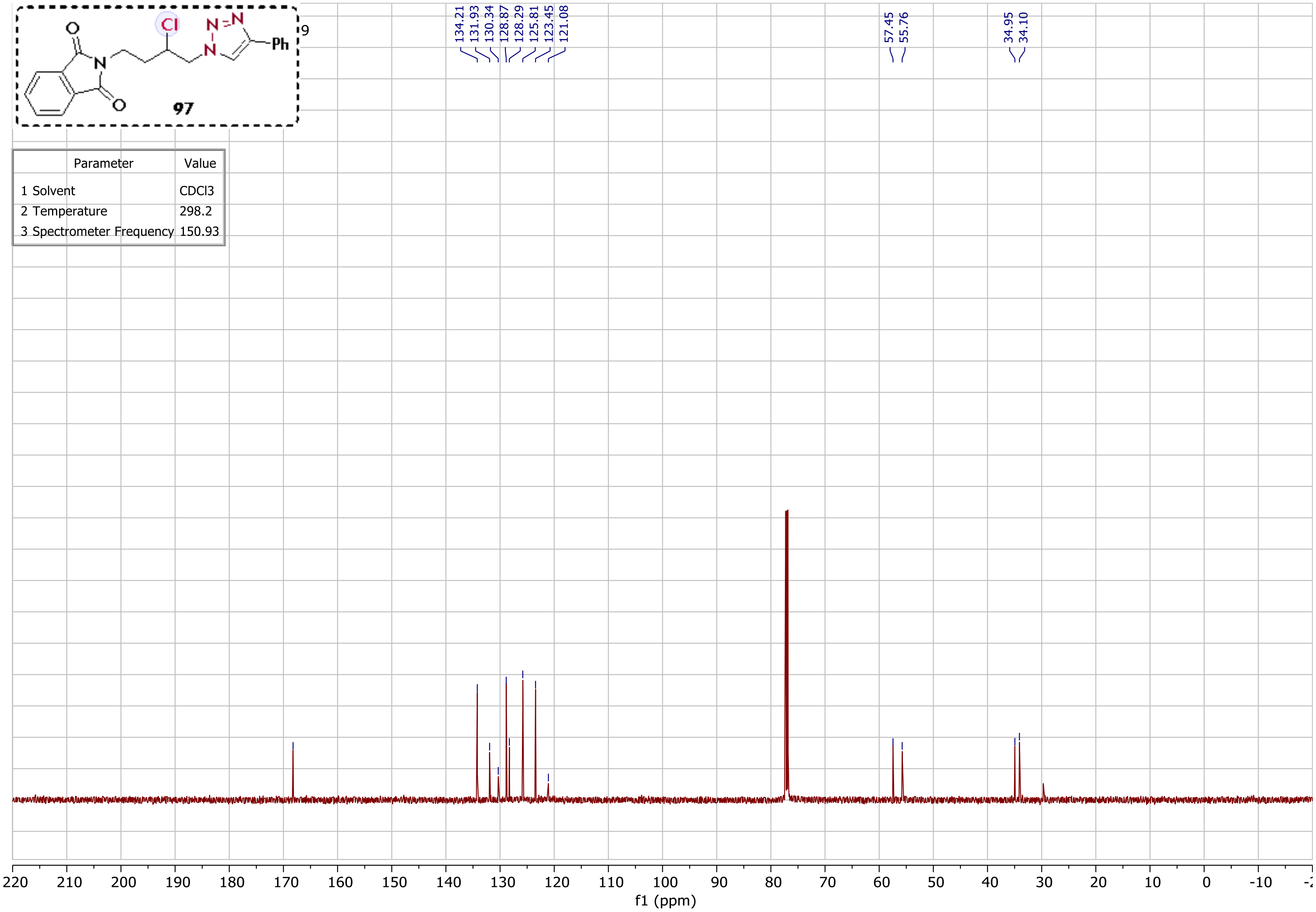

O
N
Cl
N=N
N
Ph
O
97
Parameter Value
1 Solvent CDCl3
2 Temperature 298.2
3 Spectrometer Frequency 150.93
134.21
131.93
130.34
128.87
128.29
125.81
123.45
121.08
57.45
55.76
34.95
34.10
220 210 200 190 180 170 160 150 140 130 120 110 100 90 80 70 60 50 40 30 20 10 0 -10
f1 (ppm)

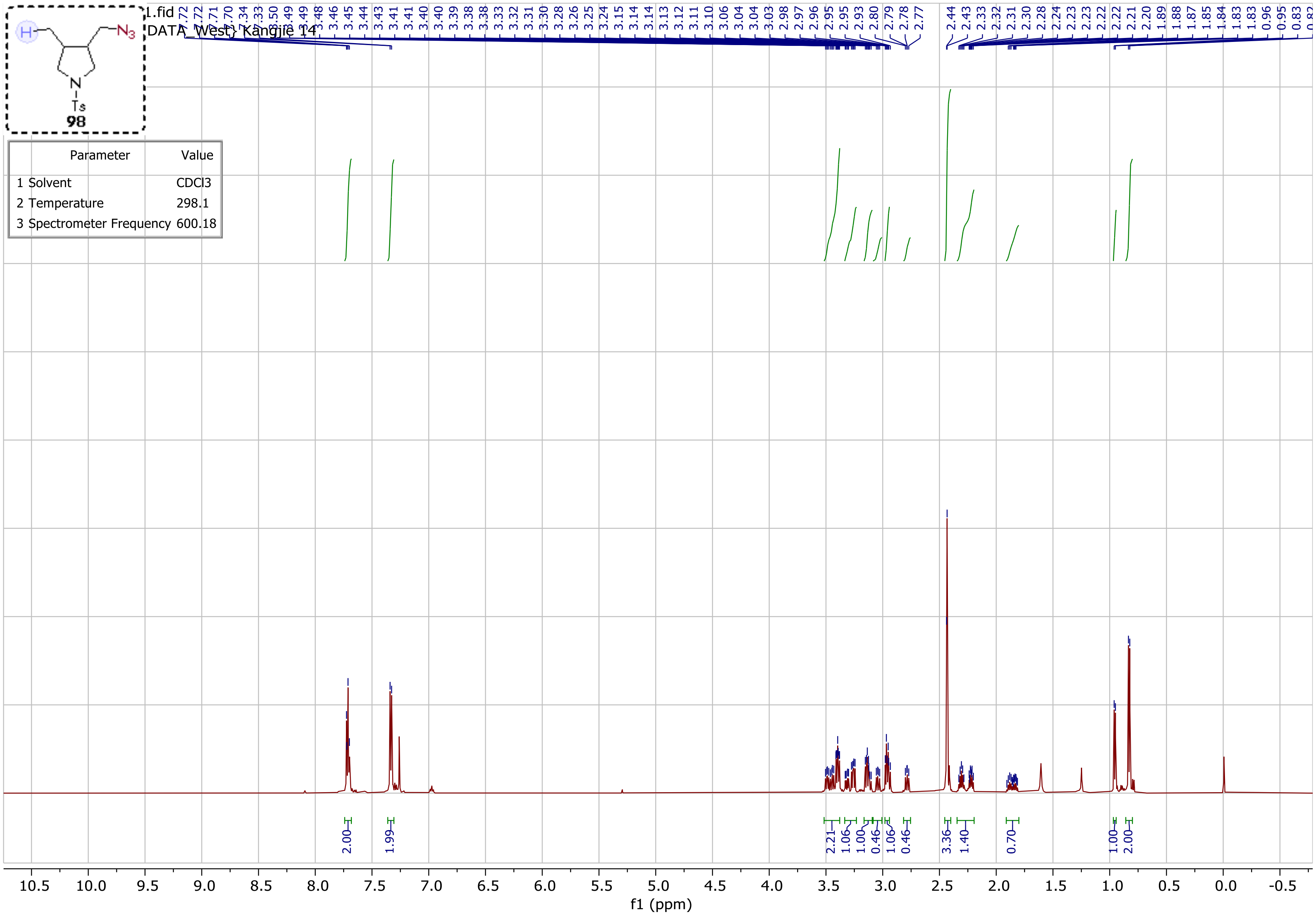
1.fid
DATA_West} Kangjie 14
N₃
Ts
98
Parameter Value
1 Solvent CDCl3
2 Temperature 298.1
3 Spectrometer Frequency 600.18
2.00
1.99
2.21
1.06
1.00
0.46
1.06
0.46
3.36
1.40
0.70
1.00
2.00
f1 (ppm)

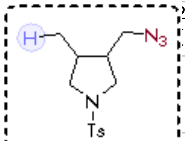

98

Parameter	Value
1 Solvent	CDCl3
2 Temperature	298.1
3 Spectrometer Frequency	150.93

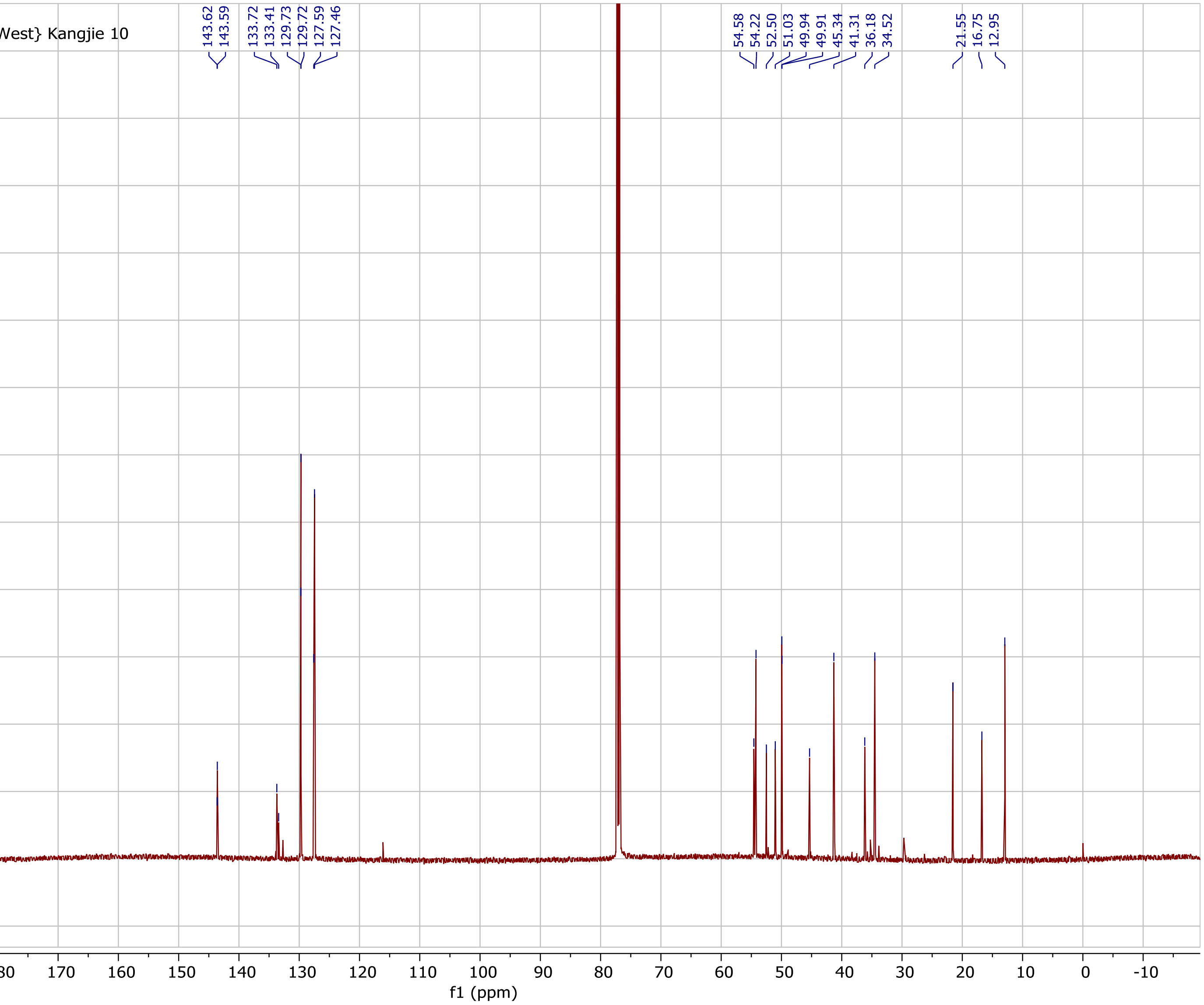

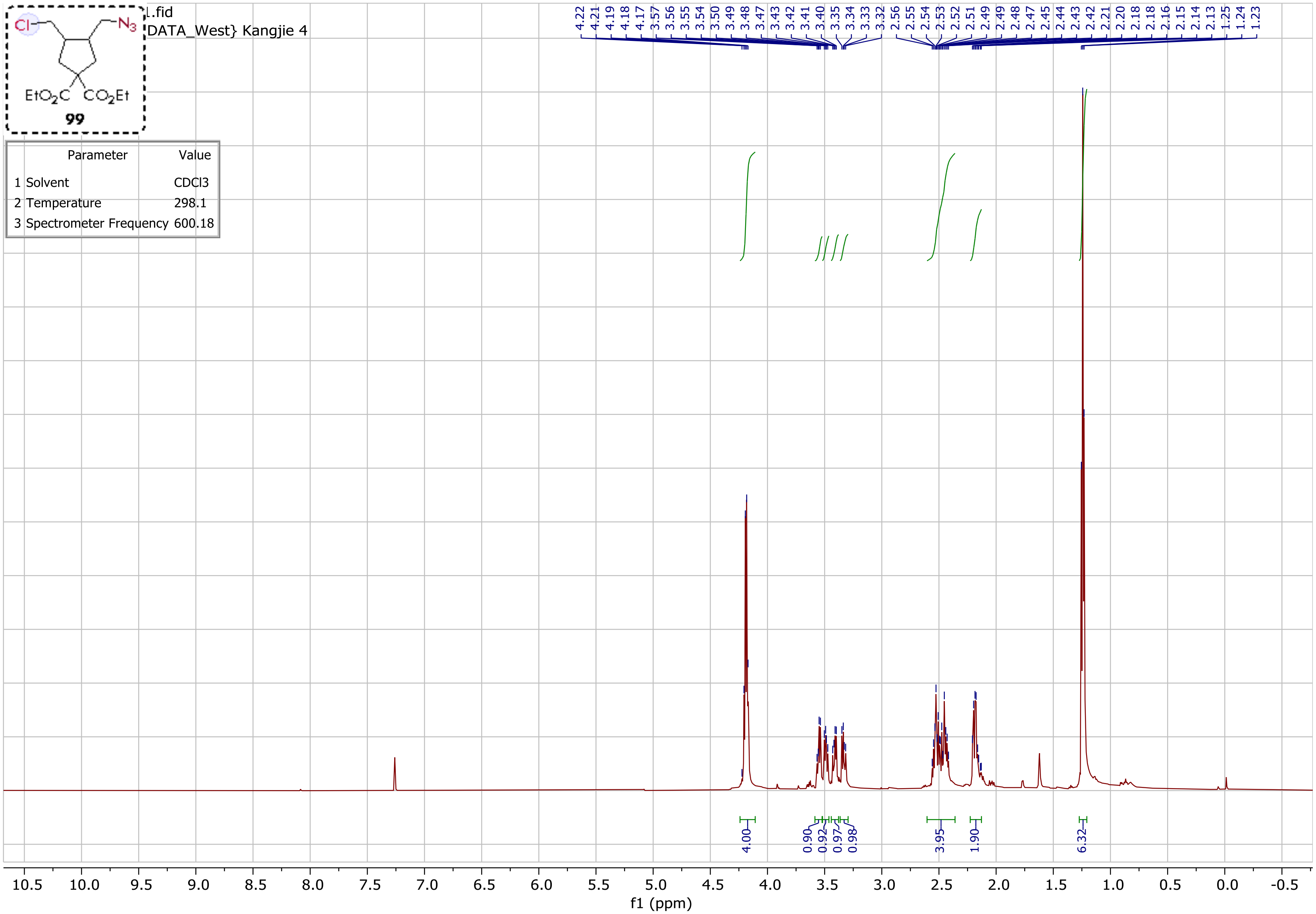
Cl
N3
EtO2C CO2Et
99
1.fid
DATA_West} Kangjie 4
Parameter Value
1 Solvent CDCl3
2 Temperature 298.1
3 Spectrometer Frequency 600.18
4.22 4.21 4.19 4.18 4.17 3.57 3.56 3.55 3.54 3.50 3.49 3.48 3.47 3.43 3.42 3.41 3.40 3.35 3.34 3.33 3.32 2.56 2.55 2.54 2.53 2.52 2.51 2.49 2.48 2.47 2.45 2.44 2.43 2.42 2.21 2.20 2.18 2.18 2.16 2.15 2.14 2.13 1.25 1.24 1.23
4.00 0.90 0.92 0.97 0.98 3.95 1.90 6.32
10.5 10.0 9.5 9.0 8.5 8.0 7.5 7.0 6.5 6.0 5.5 5.0 4.5 4.0 3.5 3.0 2.5 2.0 1.5 1.0 0.5 0.0 -0.5
f1 (ppm)

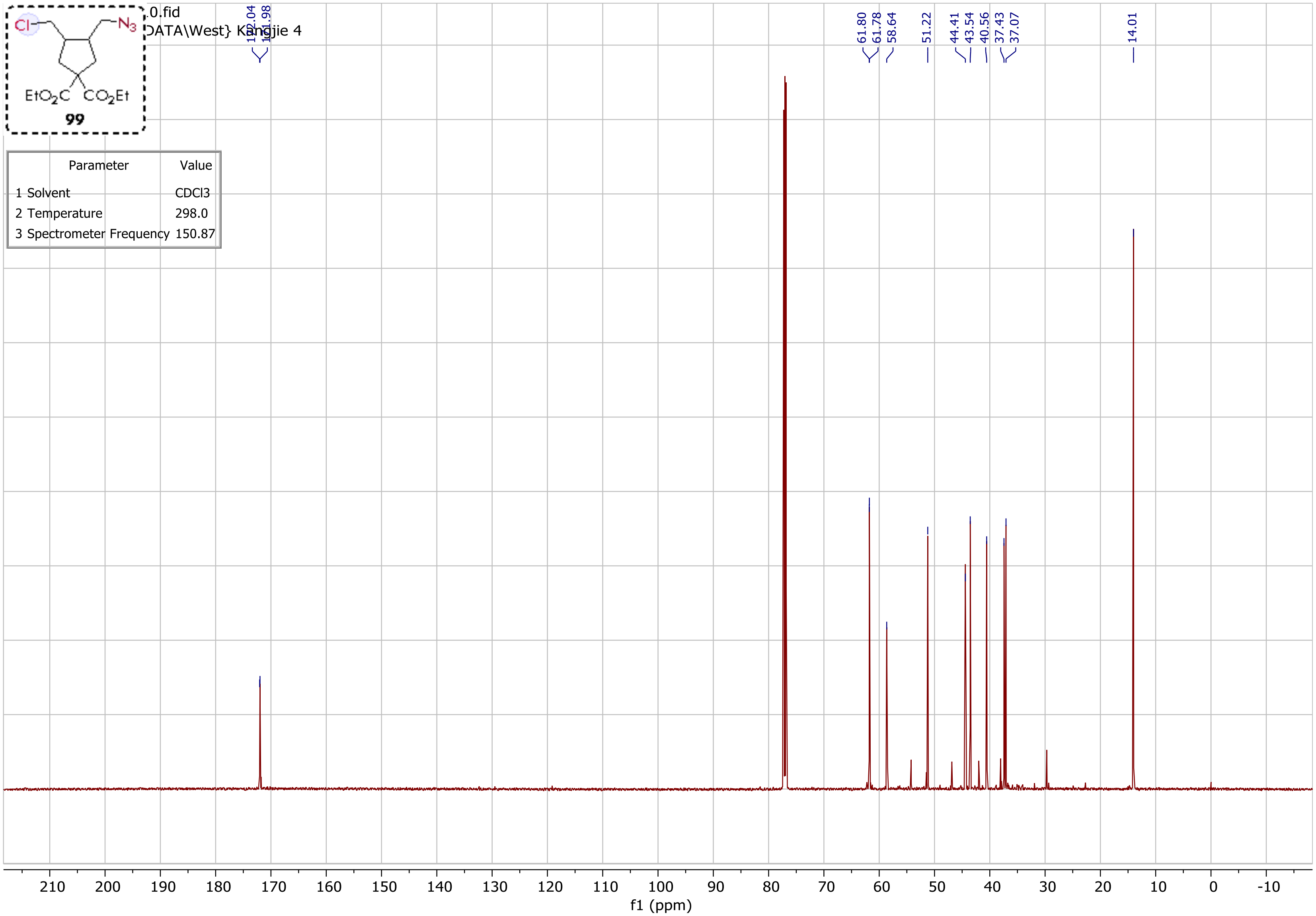

.0.fid
DATA\West} Kangjie 4
Cl
N_3
EtO_2C
CO_2Et
99
172.04
171.98
61.80
61.78
58.64
51.22
44.41
43.54
40.56
37.43
37.07
14.01
Parameter Value
1 Solvent CDCl3
2 Temperature 298.0
3 Spectrometer Frequency 150.87
210 200 190 180 170 160 150 140 130 120 110 100 90 80 70 60 50 40 30 20 10 0 -10
f1 (ppm)

IV. Supplemental References

[1] Nikitas, N. F.; Voutyritsa, E.; Gkizis, P. L.; Kokotos, C. G. *Eur. J. Org. Chem.* **2021**, *2021*, 96.

[2] Liu, J.; Lu, C.; Wu, S.; Wu, X.; Wu, Z.; Xue, X.-S.; Yu, J.; Zhu, C. *Angew. Chem. Int. Ed.* **2020**, *59*, 8195.

[3] Jiang, H.-J.; Liu, K.; Yu, J.; Zhang, L.; Gong, L.-Z. *Angew. Chem. Int. Ed.* **2017**, *56*, 11931.

[4] C. Almansa, J. Alfón, A. F. de Arriba, F. L. Cavalcanti, I. Escamilla, L. A. Gómez, et al. *J. Med. Chem.* **2003**, *46*, 3463.

[5] Aldrich, C.; Brody, S.; Cushman, M.; Fan, B.-Z.; Hiasa, H.; Liang, J.-H.; Lv, W.; Yang, Z.-Y. *Eur. J. Med. Chem.* **2020**, *193*, 112222.
